# Tuberculous Meningitis Alters the Proteomic Landscape of Brain-Derived Extracellular Vesicles

**DOI:** 10.3390/cells15191749

**Published:** 2026-09-25

**Authors:** Blanca V. Rodriguez, Nerketa N. L. Damiba, Harshita Tak, Nicole Beaubien, Daisy Puca, D. Brian Foster, Samarjit Das, Elizabeth W. Tucker

**Affiliations:** 1 Department of Anesthesiology and Critical Care Medicine, Johns Hopkins University School of Medicine, Baltimore, MD 21287, USA; bvr1222@gmail.com (B.V.R.); nbeaubien04@gmail.com (N.B.); sdas11@jhmi.edu (S.D.); 2 Center for Tuberculosis Research, Johns Hopkins University School of Medicine, Baltimore, MD 21287, USA; 3 Department of Medicine, Division of Cardiology, Johns Hopkins University School of Medicine, Baltimore, MD 21287, USA; dbrianfoster@jhmi.edu

**Keywords:** tuberculous meningitis, extracellular vesicles, neuroinflammation, proteomics, microglial activation

## Abstract

**Highlights:**

**What are the main findings?**

- This study provides the first proteomic characterization of brain-derived extracellular vesicles (EVs) in an in vivo model of tuberculous meningitis (TB meningitis).
- *M. tuberculosis* infection increases brain EV abundance and is associated with a proteomic shift enriched for microglial activation, host defense, and interferon signaling, including the excitotoxic enzyme glutaminase (GLS).
- Conversely, infection leads to a reduced representation of neuronal and neuroprotective EV-associated proteins involved in neuronal maintenance, synaptic signaling, and neuroprotection.

**What are the implications of the main findings?**

- These findings establish a foundational EV atlas for TB meningitis and nominate candidate proteins for future biomarker validation in accessible biofluids (plasma, CSF).
- The identified pathways, including EV-associated glutaminase, generate specific, testable hypotheses for mechanistic follow-up studies of EV-mediated host–pathogen interactions in the CNS.

**Abstract:**

Tuberculous meningitis (TB meningitis), the deadliest form of *Mycobacterium tuberculosis* infection, causes devastating mortality and severe neurological disability despite standard therapy. Brain injury and microglial activation are major determinants of outcome, yet the molecular mechanisms linking infection, inflammation, and neuronal injury remain poorly understood. Extracellular vesicles (EVs), critical mediators of cell-to-cell communication, have been investigated in pulmonary TB, but their role in TB meningitis remains unexplored. We used a young New Zealand White rabbit model of TB meningitis to isolate and characterize brain-derived EVs from infected and uninfected rabbits employing nanoflow cytometry, transmission electron microscopy, proteomic profiling by liquid chromatography-tandem mass spectrometry (LC-MS/MS), and bioinformatic analysis of pathways, cell-type enrichment, and protein-protein interaction networks (DAVID, Enrichr, and STRING databases). EV recovery from flash-frozen brain tissue proved feasible, enabling future studies using biobanked samples. *M. tuberculosis* infection increased EV release in brain tissue and induced a selective proteomic shift characterized by enrichment of proteins associated with TB host defense, microglial activation, and stress-response pathways, coupled with a reduced representation of neuronal and neuroprotective EV-associated proteins. These findings suggest that infection reprograms the brain EV proteome toward inflammatory and injury-associated pathways. Collectively, this study provides the first comprehensive proteomic characterization of brain-derived EVs in TB meningitis and establishes a foundational atlas of EV-associated proteins altered during infection. These findings identify candidate proteins for future biomarker validation in accessible biofluids (e.g., plasma and CSF) and provide a framework for generating hypotheses about EV-mediated host–pathogen interactions in the CNS during TB meningitis.

## 1. Introduction

Tuberculous meningitis (TB meningitis) is caused by *Mycobacterium tuberculosis* and is the most severe and life-threatening form of tuberculosis (TB) that disproportionately impacts children and immunocompromised individuals. It is characterized by high rates of mortality and life-long morbidity [1,2], with the majority of survivors experiencing neurological disability, including physical and neurodevelopmental deficits [2,3,4,5,6]. These poor outcomes occur despite appropriate antimicrobial and current standard-of-care host-directed therapy (i.e., corticosteroids), underscoring the need to improve our understanding of TB meningitis pathology. Brain injury and neuroinflammation are major determinants of outcomes. In patients, elevated brain injury biomarkers and cytokines in the cerebrospinal fluid (CSF) predict mortality [7,8], and glutamate excitotoxicity is increased, particularly in those with seizures [9,10]. Studies in cells, animals, and humans further demonstrate that microglia and astrocytes play critical roles in amplifying the inflammatory cascade that drives cytokine release and neuronal injury [11,12,13,14,15,16]. Despite these mechanistic insights, the precise molecular pathways linking infection, neuroinflammation, and neuronal damage in TB meningitis remain poorly understood.

Extracellular vesicles (EVs) are crucial mediators of intercellular communication under both physiological and pathological conditions, including infection and inflammation [17]. Their bioactive cargo (e.g., proteins, lipids, nucleic acids) can help maintain homeostasis or propagate disease-associated responses [17]. Among these cargo classes, EV-associated microRNAs (miRNAs) can influence gene expression in recipient cells and have been implicated in inflammatory signaling and host responses to infection [18]. In the central nervous system (CNS), brain-derived EVs are increasingly recognized as important regulators of neurodevelopment, neurodegenerative diseases, and neuroinflammation, with EV release often increased in response to pathological stimuli [19,20]. The proinflammatory role of EVs is well-documented across diverse disease states, ranging from infections (e.g., viral encephalitis) to neurodegenerative diseases (e.g., Alzheimer’s disease) [21,22]. Cell-specific EVs contribute to neuroinflammatory signaling in distinct ways: microglia-derived EVs propagate inflammatory signals to neighboring neurons and glia, astrocyte-derived EVs modulate synaptic function and glial activation, and neuron-derived EVs carry injury-associated cargo that may either protect against or exacerbate injury [19,20,22]. EV proteomics has emerged as a powerful approach to define CNS-specific responses to infection, as demonstrated in HIV-associated neurocognitive disorders, where CSF-derived EV proteomic profiles reflect synaptic injury and neuroinflammatory activation [23]. These findings highlight the potential of EVs not only as biomarkers but also as biologically active mediators of CNS pathology.

In TB, EV studies have largely focused on pulmonary disease. EVs derived from *M. tuberculosis* have been linked to active disease [24], whereas EVs derived from host immune cells activate macrophages and promote phagosome maturation [25,26]. However, whether EVs in the brain reflect and/or contribute to TB meningitis pathology remains unknown. The cellular origin, cargo, and function of brain-derived EVs in TB meningitis have not been defined, and no prior study has examined EVs directly in brain tissue from TB meningitis in either patients or experimental models. Defining the brain EV proteome in this setting could therefore provide mechanistic insights into neuroinflammatory signaling, brain injury, and disease-associated remodeling of the CNS microenvironment.

To address this knowledge gap, we optimized a workflow to isolate EVs from brain tissue of rabbits with and without TB meningitis and performed EV characterization, proteomic profiling, pathway analysis, and interaction analysis. The young rabbit model recapitulates key features of human TB meningitis, including robust microglial activation, heterogeneous CNS pathology (including tuberculomas and meningitis), and neuroinflammation-associated neurobehavioral deficits [11,12,16,27]. Importantly, it is the only TB meningitis model in a young animal undergoing active neurodevelopment, enabling investigation of infection-associated brain injury in a developmentally relevant context. To our knowledge, this study is the first to examine EVs in TB meningitis brain tissue and the first to define the proteomic composition of brain-derived EVs in this disease. Here, we show that *M. tuberculosis* infection alters the proteomic landscape of brain-derived EVs, establishing a foundational EV atlas for TB meningitis that identifies candidate proteins for future validation in accessible biofluids (e.g., plasma and CSF) and may help generate hypotheses about EV-mediated host–pathogen interactions during TB meningitis.

## 2. Materials and Methods

### 2.1. Animal Model

Male and female New Zealand White rabbits (Envigo Global Services Inc., Denver, PA, USA) were used for all rabbit studies. Rabbits were given at least two weeks to acclimatize to the animal biosafety level-3 (ABSL-3) housing facility before any intervention and were provided appropriate light/dark cycles and hay enrichment. All protocols were approved by the Johns Hopkins University Animal Care and Use (RB22M351, then replaced by RB25M291) and Biosafety committees. Rabbits were monitored for symptomatology (e.g., weight loss, motor deficits) and euthanized if a premorbid condition developed early per animal protocol.

#### 2.1.1. Young Rabbit Model of TB Meningitis

We utilized our young rabbit model of TB meningitis and healthy controls as previously described [11,12,27,28]. In brief, rabbits were bred in-house, and kits were delivered spontaneously. Genotyping for sex determination was performed using a 2 mm ear sample (Transnetyx, Memphis, TN, USA) prior to intervention. Young rabbits (kits) at postnatal days 4–8 received dexmedetomidine intramuscularly (IM) and topical lidocaine to the skull for analgesia and sedation prior to intraventricular injection of 20 µL of phosphate-buffered saline (PBS, uninfected) or *M. tuberculosis* H37Rv inoculum from frozen stock (i.e., infected) via the bregma (intersection of the coronal and sagittal suture). Rabbits were litter-matched and randomized to experimental groups (infected *n* = 12; uninfected *n* = 13) by weight and sex.

#### 2.1.2. Healthy Adult Rabbits

Healthy adult rabbits (female and male) used for in-house breeding were utilized to optimize EV isolation techniques prior to testing in young rabbits due to their smaller size. Supplementary Figure S1 represents 1 adult male rabbit.

### 2.2. Brain-Derived EV Isolation

#### 2.2.1. Sample Collection

Young rabbits with TB meningitis (i.e., infected) and age-matched healthy controls (uninfected) were followed for three weeks unless otherwise specified and euthanized with pentobarbital overdose (120 mg/kg; VetOne, Boise, ID, USA) for sample harvest. Three weeks were chosen due to the development of symptoms and lesions on gross pathology in infected rabbits in prior experiments [11,12]. Samples were stored at −80 °C until postmortem analysis unless otherwise specified.

#### 2.2.2. Brain Tissue Processing and EV Isolation

We optimized EV isolation protocols in two stages. First, we performed a pilot comparison of fresh and frozen brain hemispheres from a single adult rabbit (~4.5 g) to assess the technical feasibility of recovering EVs from frozen tissue. Second, we tested the protocol on smaller brain samples from one-week-old rabbits (~1 g) to ensure the method could be applied to smaller neonatal tissue volumes. Samples were weighed, and EV yield was corrected for tissue weight. This two-stage approach established a working protocol before application to the main study cohort.

For all EV isolations, brains were minced and incubated in 10 mL of EV-release media [29], composed of Neurobasal^TM^ medium (Gibco^TM^, Waltham, MA, USA, cat# 21103049), supplemented with 1% GlutaMAX^TM^ supplement (Gibco^TM^, cat# 35050038) and 1% Antibiotic-Antimycotic (100×) (Gibco^TM^, cat# 15240062) at 37 °C in a 5% CO_2_ environment for 24 h, except in the adult freshly-collected brain tissue, which was incubated for 16 h. The brain tissue supernatant was then clarified by differential centrifugation (300× *g* for 10 min, 3000× *g* for 10 min) and filtration through a 0.22 µm syringe filter (Millipore, Burlington, MA, USA, cat# SLGPR33RS) to remove *M. tuberculosis*, cellular debris, apoptotic bodies, and larger microvesicles. The resulting supernatant contained the released EVs and underwent further purification through additional centrifugation (500× *g* for 10 min, 3000× *g* for 20 min, and 12,000× *g* for 20 min) at room temperature (RT). The cleared supernatant was concentrated by centrifugation at 4000× *g* for 10 min at RT using Amicon Ultra-15 centrifugal filters (100 kDa MWCO) prior to size-exclusion chromatography (SEC) using an automatic fraction collector (Izon Science, Christchurch, New Zealand, cat# AFC-V2). The concentrated supernatant was fractionated into 0.5 mL fractions using Gen 2 qEVoriginal/35 nm SEC columns (Izon Science, cat# ICO-35). Fractions were collected, concentrated to 150–200 µL using Amicon Ultra-0.5 mL centrifugal filters (10 kDa MWCO; Millipore Sigma, cat# UFC501008) and stored at −20 °C until analysis. Because our isolation workflow enriches EVs but does not yield fully purified vesicle preparations, we refer to the isolated material as EV-enriched fractions; for readability, we use the term EVs throughout the manuscript unless otherwise specified. Figure 1 illustrates the EV isolation protocol from brain tissue.

### 2.3. EV Quantification by Nanoflow Cytometry (NanoFCM)

A NanoFCM Flow NanoAnalyzer (NanoFCM Co., Ltd., Xiamen, China) was used to measure EV concentration and size. Briefly, lasers were aligned and calibrated separately: fluorescent 250 nm silica nanoparticles at a concentration of 2.19 × 10^10^ (NanoFCM, cat# QS2503) were used for particle concentration, and a premixed silica nanosphere cocktail containing monodisperse nanoparticle populations of 68 nm, 91 nm, 113 nm, and 155 nm in diameter was used for size calibration (NanoFCM, cat# 516 M-Exo). PBS was used as the blank for background correction, and EVs were diluted 100-fold in PBS for particle quantification. Particle signal acquisition was performed for 1 min at a constant pressure of 1 kPa, with an event rate between 2000 and 12,000 events/min. The side-scattering of each sample was analyzed using the NanoFCM Professional Suite V2.0 software. All animals with successful EV isolation and quantification were included in the analysis.

### 2.4. Transmission Electron Microscopy (TEM)

A subset of the EV samples was further characterized using TEM to visualize the particles and confirm expected EV morphology in EV-enriched fractions. Samples (8 µL) were adsorbed to glow-discharged (Electron Microscopy Sciences [EMS] GloQube, Hatfield, PA, USA) ultra-thin (UL) carbon-coated 400 mesh copper grids (EMS, cat# CF400-Cu-UL), by flotation for 2 min. Grids were rinsed in 3 drops (1 min each) of Tris-buffered saline (TBS) buffer, negatively stained in 2 consecutive drops of 1% uranyl acetate (UA, aq.), and quickly aspirated. Grids were imaged on a Hitachi 7600 TEM operating at 80 kV with an AMT XR80 CCD (8 megapixels).

### 2.5. Bicinchoninic Acid (BCA) Assay

The protein concentration of EV samples was determined using a standard BCA protein assay (Thermo Fisher Scientific, Waltham, MA, USA, cat# 23225) or Pierce Dilution-Free Rapid Gold BCA Protein Assay Kit (Thermo Fisher Scientific, cat# A55860) as indicated in the manufacturer’s manual. Briefly, samples were lysed at a 1:1 ratio with RIPA buffer (Thermo Fisher Scientific, cat# 89900) supplemented with protease inhibitors (Thermo Fisher Scientific, cat# 78425) on ice for 15 min [30]. Ten microliters of diluted or undiluted samples were added to a 96-well plate, followed by 200 µL of the BCA/copper complex solution from the kit. Absorbance was measured at 564 nm for the standard BCA or 480 nm for the Pierce Dilution-Free Rapid Gold BCA in a microplate reader (Tecan Spark Multimode Microplate Reader (Tecan Austria GmbH, Grödig, Austria)), and protein concentration was determined against a bovine serum albumin (BSA) standard curve.

### 2.6. SDS-PAGE and Western Blot Analysis

EV samples were lysed 1:1 with ice-cold RIPA lysis buffer (Thermo Scientific, cat# 89900) containing 1× Halt protease inhibitor cocktail (Thermo Scientific, cat# 78430) for 15 min. To denature and reduce the protein in the samples, a 1:4 ratio of Laemmli buffer (Bio-Rad, Hercules, CA, USA, cat# 1610747) with dithiothreitol (DTT) was prepared and combined with the lysed EV samples at 95 °C for 5 min. BCA protein assay was used to quantify protein. The denatured protein (10–20 µg) was loaded onto pre-cast polyacrylamide gels, 4–20% Mini-PROTEAN^®^ TGX Stain-Free™ Protein Gels (Bio-Rad, cat# 4568093), with 1× Tris/Glycine/SDS (Bio-Rad, cat# 1610732) and subsequently transferred to nitrocellulose membranes using the Trans-Blot Turbo Mini 0.2 µm Nitrocellulose Transfer Packs (Bio-Rad, cat# 1704150). The membranes were blocked using a blocking buffer (SuperBlock T20 TBS Blocking Buffer, Thermo Scientific, cat# 37536) for 30 min at RT. The membranes were then incubated overnight at 4 °C with specific primary antibodies (Table 1) diluted in blocking buffer. The next day, membranes were washed three times in TBS containing 0.1% Tween-20 (TBS-T) for 10 min and then incubated with appropriate secondary antibodies (Table 2) at RT for 1 h. After the secondary antibody was removed with three washes of TBS-T, the membranes were incubated for 5 min with the following chemiluminescent substrates to visualize protein bands on the membranes: SuperSignal West Pico Maximum Sensitivity Substrate (Thermo Scientific, cat# 34580) or SuperSignal West Femto Maximum Sensitivity Substrate (Thermo Scientific, cat# 34095). The membranes were imaged with the ChemiDoc MP Touch imaging system (Bio-Rad) and analyzed using ImageJ (version 2.16.0/1.54p, NIH, Bethesda, MD, USA) software.

### 2.7. Dot Blot

Two microliters of EV lysate containing 100 ng of total protein were spotted onto nitrocellulose membranes. Separate membranes were prepared for each antibody tested. After air-drying for 15 min, the membranes were blocked in blocking buffer for 30 min at RT on a rocking platform. Primary antibodies (Table 1) were diluted in blocking buffer and incubated overnight at 4 °C with individual membranes. The membranes were then washed three times with TBS-T, 10 min each wash, and then incubated with secondary antibody (Table 2) for 1 h at RT. After washing, the signals were developed, imaged, and analyzed as described for Western blots.

### 2.8. Proteinase K Protection Assay

Proteinase K (PK) protection assay was used to distinguish surface-accessible EV proteins from proteins protected by the EV membrane, including intraluminal proteins. EV samples from three biological replicates per group (uninfected and infected) were normalized to a concentration of 100 ng/µL total protein and divided into three treatment groups: (1) untreated intact EVs (vehicle control); (2) EVs treated with PK (Thermo Fisher Scientific, cat# EO0491) alone; and (3) EVs pre-treated with Triton X-100 (Sigma-Aldrich, Darmstadt, Germany, cat# T8787-50ML) followed by PK. For permeabilization, EVs were incubated with 1% (*v*/*v*) Triton X-100 on ice for 15 min. Samples were then treated with PK (0.5 µg/mL final concentration) for 1 h at 37 °C. PK was inactivated by the addition of phenylmethylsulfonyl fluoride (PMSF; Cell Signaling Technology, Danvers, MA, USA, cat# 8553S) to a final concentration of 2 mM, followed by incubation at RT for 15 min. All reactions were adjusted to a final volume of 40 µL using PBS. A 1 µL aliquot from each sample (containing 100 ng EV protein) was then applied to a nitrocellulose membrane for dot blot analysis to determine if EV-associated proteins were digested by PK alone (i.e., surface-associated proteins) or required permeabilization with Triton X-100 (i.e., intraluminal or otherwise membrane-protected proteins).

### 2.9. Comparative Proteomics of Brain-Derived EVs

After EV isolation, a subset of EVs (*n* = 5 animals/group) was sent for comparative proteomic analysis, with sample identities blinded to the investigators performing the analysis. EVs were solubilized in 1% SDS and sonicated. Twenty micrograms of total protein per sample were digested with trypsin/lysC, desalted with a C18 column, and reconstituted in 0.1% formic acid. Liquid chromatography-tandem mass spectrometry (LC-MS/MS) analysis was conducted using a Thermo Orbitrap Exploris 240 Mass Spectrometer and a Thermo Vanquish Neo RSLCnano system (Poochon Scientific, Frederick, MD, USA). Peptide mixture from each sample was loaded onto a peptide trap cartridge at a flow rate of 5 µL/min. The trapped peptides were eluted onto a reversed-phase EasySpray column using a linear gradient of acetonitrile (3–36%) in 0.1% formic acid. The elution duration was 110 min at a flow rate of 0.3 µL/min. Eluted peptides from the EasySpray column were ionized and sprayed into the mass spectrometer, using a Nano-EasySpray Ion Source (Thermo) under the following settings: spray voltage, 1.6 kV; capillary temperature, 275 °C. All samples were processed and analyzed in a single continuous instrument run, so explicit batch correction was not required. Raw data files acquired from each sample were searched against *Oryctolagus cuniculus* and *M. tuberculosis* (strain ATCC 25618/H37Rv) protein sequence databases using the Proteome Discoverer 2.5 software (Thermo, San Jose, CA, USA) based on the SEQUEST algorithm. Carbamidomethylation (+57.021 Da) of cysteines was a fixed modification, and Oxidation Met and Deamidation Q/N-deamidated (+0.98402 Da) were set as dynamic modifications. The minimum peptide length was specified as five amino acids. The precursor mass tolerance was set to 15 ppm, whereas fragment mass tolerance was set to 0.05 Da. The maximum false peptide discovery rate was specified as 0.01. The resulting Proteome Discoverer Report contains all assembled proteins with peptide sequences and peptide spectrum match counts (PSM#) and MS1 peak area abundance. Protein quantification/normalization used the normalized spectral abundance factors (NSAFs) method to calculate the protein relative abundance [31,32]. The proteomic dataset is provided as a Supplementary Table S1.

### 2.10. Bioinformatics: Ortholog Mapping and Functional Enrichment Analysis

To reduce cross-species annotation bias, rabbit protein identifiers were mapped to human orthologs using UniProt (release 2026_01) and Ensembl BioMart (release 115; source species: *Oryctolagus cuniculus*) prior to all downstream enrichment and network analyses. BioMart outputs included rabbit and human gene symbols, Ensembl IDs, orthology type, orthology confidence scores, and percent identity. To maximize annotation reliability, only high-confidence one-to-one human orthologs were retained for primary analyses. Proteins lacking a human ortholog, exhibiting low-confidence orthology assignments, or mapping to one-to-many or many-to-many ortholog relationships were excluded. Because rabbit-specific pathway and functional annotation resources remain limited, all enrichment and network analyses were performed using the mapped human orthologs and are therefore interpreted as human ortholog-based functional summaries rather than direct rabbit-specific annotations. The rabbit-to-human ortholog mapping is provided as a Supplementary Table S2.

Subcellular localization enrichment was performed using the Database for Annotation, Visualization, and Integrated Discovery (DAVID; v2023q4), against the *Homo sapiens* proteome background and restricted to Gene Ontology Cellular Component (GO-CC) Direct terms. Enrichment significance was assessed using DAVID’s built-in Fisher’s exact test with Benjamini-Hochberg correction; terms with adjusted *p* < 0.05 were considered significantly enriched. The top enriched GO-CC terms were visualized as bar graphs in GraphPad Prism (version 11.0.1, Boston, MA, USA). To reduce figure complexity, broad parent-child terms were collapsed or omitted when more informative child terms were present.

Pathway and cell-type enrichment analyses were performed using Enrichr with mapped human ortholog gene lists. Cell-type enrichment used the CellMarker 2024 database. Pathway enrichment utilized Reactome Pathways 2024 for upregulated and downregulated proteins and WikiPathways 2024 for proteins detected exclusively in EVs from infected rabbits. Enriched terms were ranked by adjusted *p*-value, and the most significant pathways were visualized as bar graphs in GraphPad Prism. As above, broad categories lacking biological specificity were excluded when more informative child terms were available.

Protein association networks were constructed in Cytoscape (version 3.10.1) using the stringApp plugin (version 2.0.1) with human orthologs (reference species: *Homo sapiens*). Full STRING networks were generated using a minimum interaction confidence threshold of 0.40 with genome background enrichment enabled. Networks were clustered using the clusterMaker2 MCL algorithm (version 2.3.4; inflation parameter = 2.5). To facilitate biological interpretation, cluster-specific enrichment analyses were subsequently performed on individual network modules by interrogating the constituent nodes within each cluster. Enriched biological processes and pathways were categorized according to their respective cluster assignments.

To evaluate the overlap of brain EV proteins with known EV cargo, we compared all upregulated, downregulated, and infection-specific proteins (i.e., proteins detected exclusively in *M. tuberculosis*-infected samples) to the Vesiclepedia database (https://www.microvesicles.org/index.html) using FunRich (version 3.1.4). Because Vesiclepedia aggregates data generated using diverse EV isolation methods and biological sources, overlap with the database was interpreted as supportive, but not definitive, evidence of EV association. Venn diagrams were generated to visualize shared and unique protein subsets between our dataset and proteins cataloged in Vesiclepedia.

### 2.11. Statistical Analysis

All data were analyzed using Prism 11 software (GraphPad), except for the proteomic data as described below. Sample size was based on the number needed to detect differences in prior rabbit and proteomic studies. Data are expressed as mean ± SD, except for the proteomic data. Comparisons of parametric data were performed using *t*-tests for single comparisons and ordinary one-way analysis of variance (ANOVA) followed by Tukey’s multiple comparison tests for multiple comparisons. Mixed-effects analysis (REML) was used to compare rabbit weight (g) over time. *p*-values ≤ 0.05 were considered statistically significant. For proteomic analysis, empirical Bayesian-modified *t*-tests were performed in R (version 4.4.3, Vienna, Austria) using the linear modeling of microarrays (LIMMA) R package (version 3.60.6). To account for multiple comparisons in the proteomic dataset, *p*-values were adjusted using the Benjamini-Hochberg procedure to control the false discovery rate (FDR). Proteins were considered significantly differentially expressed at an FDR-adjusted *p* < 0.05. Principal component analysis (PCA) was performed on proteins with *p* < 0.05. Heatmaps showing log2-transformed, median-centered protein abundance values across individual samples were generated using the ComplexHeatmap R package (version 2.20.0), with row clustering based on Euclidean distance. Volcano plots highlight proteins passing the significance threshold and were labeled based on the top 50 *p*-values and top 20 absolute log_2_ fold changes. All visualizations were created in R using ggplot2 (version 3.5.2), ggrepel (version 0.9.6), and ComplexHeatmap packages. Functional relationships between proteins were collated with stringApp embedded in Cytoscape, using a string score of 0.4 as the evidence threshold for association. Figures and graphs were created with BioRender (accessed 2025, Toronto, ON, Canada), GraphPad, and CorelDRAW 2022 (Ottawa, ON, Canada).

## 3. Results

### 3.1. EV Recovery from Fresh and Flash-Frozen Adult Rabbit Brain Tissue

To our knowledge, EV isolation from rabbit brain tissue has not been previously reported. To address this gap, we tested whether the spontaneous “EV release” method, originally developed for mouse and human brain tissue [29], could be adapted for use in rabbits. This method was combined with standard EV purification steps including differential centrifugation, ultrafiltration, and size-exclusion chromatography (SEC), as illustrated in Figure 1.

Initial technical feasibility testing was performed using brain tissue from uninfected adult rabbits. Because brain tissue collection under biosafety level-3 (BSL-3) conditions limits the ability to process fresh samples immediately postmortem, we evaluated whether the protocol could be adapted for flash-frozen tissue without compromising EV integrity. A single adult rabbit brain was bisected; one hemisphere was processed fresh, while the other was flash-frozen for one week prior to EV isolation (Figure S1). This hemispheric comparison was designed as a technical feasibility assessment of flash-frozen, biobanked tissue rather than a biologically replicated experiment and should be interpreted accordingly. Following SEC, 13 fractions (F) were collected and analyzed using BCA protein assay and NanoFCM. In fresh tissue, EVs were enriched in fractions F1–F3, while in frozen tissue, enrichment extended through F1–F4. TEM confirmed abundant EV-like particles in fractions F1–F3 of fresh samples and F1–F5 of frozen samples. TEM also revealed the expected cup-shaped morphology, with diameters ranging from 50–200 nm. Mean particle size was comparable between conditions, and EV yield, normalized to tissue weight, was slightly higher in frozen samples. While our isolation protocol enriches for EVs, it does not yield complete purity. For clarity and readability, we refer to these EV-enriched fractions as ‘EVs’ throughout the manuscript and interpret all associated proteomic findings in the context of this limitation. Taken together, these findings demonstrate the technical feasibility of recovering EVs from flash-frozen rabbit brain tissue under these experimental conditions and support the use of our biobanked samples. However, additional biological replicates will be required to determine the reproducibility and equivalence of EV recovery between fresh and frozen tissue.

### 3.2. EV Recovery from Neonatal Rabbit Brain Tissue

Building on our results in adult rabbits, we next applied the optimized “EV release” method to brain tissue collected from one-week-old rabbits to assess EV yield from smaller samples. EVs were isolated from an uninfected rabbit and an infected littermate sacrificed 24 h post-infection (Figure S2). As in adults, EVs were enriched in fractions F1–F5 in both samples, as shown by NanoFCM analysis of all 13 fractions and TEM of pooled EV-enriched fractions, which confirmed the presence of vesicles with characteristic morphology and size (Figure S2A–D).

To further characterize EVs from one-week-old rabbits, we performed Western blot analysis on SEC fractions for the EV markers Hsp70 and Alix, along with GCPII, a protein associated with microglia and astrocytes. These proteins were enriched in fractions F1–F5, consistent with NanoFCM and TEM data (Figure S2E,F). We then pooled fractions F1–F5 from each sample to compare EV cargo between the uninfected and infected groups. Western blotting revealed similar GCPII levels in both groups, while Hsp70 and ATP1A3 appeared more abundant in EVs from the infected rabbit (Figure S2G). Overall, these findings confirmed that fractions F1–F5 were EV-enriched in both adult and neonatal rabbit brains, supporting their use in our subsequent 21-day post-infection study.

### 3.3. M. tuberculosis Infection Increases EV Release in Young Rabbit Brain Tissue

To determine whether *M. tuberculosis* infection alters brain EV abundance, we analyzed brain-derived EVs isolated from rabbits 21 days after infection. Consistent with previous studies, infected rabbits had lower body weights than uninfected rabbits (*p* = 0.027; Figure S3) [11,16,28]. Representative TEM images revealed typical cup-shaped EVs in both uninfected and infected rabbits (Figure 2A), and NanoFCM analysis showed similar size distributions and mean particle diameters across groups (Figure 2B,C). Despite these similarities in morphology and size, the total EV concentration was significantly higher in infected rabbits (Figure 2D). Due to uneven sex distributions, we found no differences in EV concentration based on sex (Figure S4). Although EV concentration increased significantly in infected brain tissue, total EV protein levels were comparable to uninfected samples and trended lower after normalization to absolute particle number determined by NanoFCM (Figure 2E,F). Interestingly, canonical EV markers such as Hsp70 and Flotillin-1 did not show significant differences between infected and uninfected samples (Figure S5). Together, these results suggest that the increase in EV concentration observed in infected brain tissue is not accompanied by a proportional increase in total EV protein content, but instead may reflect heterogeneous remodeling of EV cargo composition and/or altered vesicle secretion dynamics during infection.

### 3.4. M. tuberculosis Infection Remodels the Brain EV Proteome

To investigate the impact of *M. tuberculosis* infection on brain-derived EVs, we performed label-free quantitative proteomic analysis of EVs isolated from brain tissue from a subset of infected and uninfected rabbits. Because these samples are EV-enriched preparations rather than pure EVs, we refer to the proteins identified by proteomics as EV-associated proteins. Principal component analysis (PCA) revealed clear group separation, indicating distinct EV proteomic signatures between infected and uninfected samples (Figure 3A). Differential expression analysis identified proteins that were significantly enriched or depleted in brain-derived EVs during *M. tuberculosis* infection. A volcano plot showed proteins that were significantly upregulated or downregulated in EVs from infected compared to uninfected rabbits (*p* < 0.05, |log_2_FC| > 1; Figure 3B). Hierarchical clustering of the top 30 proteins meeting a FDR threshold of *p* < 0.05 revealed clear group-specific clustering, with several host response proteins elevated during infection (Figure 3C). In total, 321 proteins were significantly upregulated, 248 were downregulated, and 184 were exclusively detected (i.e., infection-specific) in brain-derived EVs from infected rabbits. A large proportion of the differentially expressed and infection-specific proteins overlapped with proteins cataloged in the Vesiclepedia database, supporting enrichment of known EV-associated proteins within the dataset (Figure 3D). In addition to canonical EV-associated proteins, the dataset also contained proteins previously reported in non-vesicular extracellular particles (NVEPs), lipoproteins, and other extracellular fractions, including ApoA1, ApoB, calnexin, and proteins associated with ribonucleoprotein-, exomere-, and supermere-enriched fractions (e.g., AGO1, YBX1, ENO1/ENO2, VPS26A) [33,34,35,36,37,38,39].

To evaluate the subcellular origin of EV cargo alterations, we performed cellular compartment enrichment analysis (DAVID) on upregulated, downregulated, and infection-specific proteins from infected rabbits (Figure 3E–G). All groups showed enrichment of extracellular exosome, cytoplasm, and cytosol terms, consistent with canonical EV-associated cargo. Upregulated proteins showed additional enrichment in components involved in protein turnover and trafficking, including proteasome accessory complexes, focal adhesion sites, and secretory granule lumens. In contrast, downregulated proteins were enriched in neuronal compartments, such as the glutamatergic synapse, axon, and neuromuscular junction, suggesting lower inclusion of neurostructural or signaling proteins. Infection-specific proteins were linked to a broader range of compartments, including the extracellular matrix, mitochondrial matrix, and nucleoplasm, suggesting an infection-associated diversification of EV-associated protein sources.

### 3.5. Host Immune and Stress-Response Proteins Are Enriched in Brain-Derived EVs During M. tuberculosis Infection

To investigate the biological relevance of proteins upregulated in brain-derived EVs from infected rabbits, we performed in silico enrichment and protein interaction network analyses. Enrichr CellMarker 2024 analysis revealed strong enrichment for markers of microglia, astrocytes, and immune cell populations, suggesting a shift in cell type–associated protein signatures during infection (Figure 4A). Reactome Pathways 2024 analysis of these upregulated proteins identified enrichment in broad metabolic, immune, and vesicle trafficking processes, including protein turnover, innate immune activation, and membrane transport (Figure 4B). STRING protein-protein interaction analysis clustered the upregulated proteins into five distinct functional groups (Figure 4C and Figure S6). Cluster-specific enrichment pointed to stress response, immune activation, and neurodegeneration-related pathways (Cluster 1), cytoskeletal remodeling and angiogenic signaling (Cluster 2), vesicle transport and phagocytic function (Cluster 3), Golgi-endoplasmic reticulum trafficking (Cluster 4), and cell-matrix adhesion and survival signaling (Cluster 5) (Figure 4D). Together, these results indicate that EVs from infected brain tissue are enriched in host proteins associated with immune and glial responses, stress adaptation, intracellular trafficking, and neurodegeneration-related pathways, consistent with infection-associated remodeling of the brain EV proteome.

Representative proteins underlying these enriched pathways have previously been implicated in both TB pathogenesis and neuroinflammatory disease. Cathepsin B (CTSB), a lysosomal protease that promotes inflammasome activation and IL-1β release, is elevated in the lungs of *M. tuberculosis*-infected mice and rabbits and in the plasma of patients with active TB [40], underscoring its systemic role in infection. Surfactant protein D (SFTPD), which binds to mycobacterial glycolipids [41] and facilitates bacterial clearance [42], and immunoglobulin M (IgM) heavy chain (IGHM) were both elevated in EVs from infected brain. Although SFTPD has traditionally been considered lung-specific, it has recently been detected in human CSF and CNS tissue under both healthy and pathological conditions [43,44], suggesting a role in CNS immune surveillance. Increased IGHM is consistent with early activation of humoral immunity during infection, and its presence in CSF has been associated with disease severity, poor prognosis, and treatment response in TB meningitis [45]. However, the interpretation of SFTPD and IGHM warrants caution, as both proteins may reflect infection-associated blood-brain barrier disruption and/or co-isolated NVEPs. Annexin A1 (ANXA1), an anti-inflammatory mediator that promotes resolution of inflammation and clearance of apoptotic cells [46], was also elevated during infection. In the CNS, annexin A1 is expressed by microglia and has been shown to reduce neuroinflammation [47] and ameliorate amyloid beta pathology in Alzheimer’s disease models [48], while its absence (i.e., annexin A1-deficient mice) leads to higher *M. tuberculosis* burden and disorganized granulomas [49]. Its presence in brain-derived EVs may reflect a host-protective response to limit tissue damage in TB meningitis.

### 3.6. Infection-Specific EV-Associated Proteins Are Enriched for Microglial and Interferon-Related Immune Signatures

To characterize infection-specific EV-associated proteins, we analyzed proteins exclusively detected in brain-derived EVs from infected rabbits. Enrichr CellMarker 2024 analysis revealed strong enrichment for microglial, monocyte, neutrophil, and myeloid-derived suppressor cell markers, indicating enrichment of proteins associated with innate immune cell markers (Figure 5A). WikiPathways 2024 analysis further revealed enrichment in TB-associated immunity, microglial phagocytosis, and type I interferon (IFN) signaling, as well as broader infection-response pathways (Figure 5B). STRING interaction network analysis grouped these proteins into five distinct clusters (Figure 5C and Figure S7). STRING enrichment analysis of each cluster highlighted innate immune defense and IFN-stimulated signaling (Cluster 1), mTORC1 signaling and autophagy (Cluster 2), metabolic remodeling (Cluster 3), splicing-related functions (Cluster 4), and heat shock protein binding and proteostasis (Cluster 5) (Figure 5D). These data suggest that EVs in the infected brain are enriched with proteins linked to microglial activation, IFN-driven immune signaling, and host defense, identifying these processes as candidates for future mechanistic studies of brain-derived EVs during *M. tuberculosis* infection.

Consistent with the enrichment analyses, several individual proteins uniquely detected in EVs from infected brains have established roles in neuroinflammation, microglial activation, and host defense. Glycoprotein nonmetastatic melanoma protein B (GPNMB), a marker of disease-associated microglial activation [50], galectin-3 (LGALS3), a known regulator of microglial activity and neuroinflammation [50,51], and lactotransferrin (LTF), which is released by activated microglia and has immunomodulatory and antimicrobial activity, were only detected in EV-enriched fractions from infected rabbits. The presence of these proteins is consistent with increased microglial activation during TB meningitis [11,12,13,16]. The IFN-stimulated proteins ISG15, OAS3, IFIT3, GBP1, and STAT1 were also enriched, consistent with the type I IFN bias in active TB [52,53,54]. Within this group, GBP1 was notable for its IFN-inducibility, anti-mycobacterial function, and emerging value as a TB biomarker [55]. Additionally, we detected gasdermin D (GSDMD), a protein cleaved during NLRP3 inflammasome activation to trigger pyroptosis and IL-1β release [56], suggesting a potential vesicle-associated mechanism for amplifying inflammasome signaling in the CNS. Finally, the neutrophil alarmins S100A8 and S100A9 were uniquely present, aligning with their elevated CSF levels in TB meningitis patients and their established role in neutrophil-driven inflammation [57].

### 3.7. M. tuberculosis Infection Reduces Neuronal and Neuroprotective EV-Associated Proteins

To gain insight into host processes that are downregulated during *M. tuberculosis* infection, we analyzed proteins significantly underrepresented in brain-derived EVs from infected rabbits. CellMarker 2024 enrichment revealed that these proteins were highly associated with neuronal, oligodendroglial, and astrocytic cell types, suggesting a reduction in proteins involved in neuronal maintenance, myelination, and glial homeostasis (Figure 6A). Pathway analysis using Enrichr Reactome Pathways 2024 indicated that downregulated proteins were involved in neurodevelopmental and neurodegenerative pathways, synaptic signaling, cell adhesion, oxidative stress responses, and metabolic regulation (Figure 6B). Protein-protein interaction analysis via STRING revealed five distinct functional clusters among downregulated proteins (Figure 6C and Figure S8). STRING enrichment analysis showed that downregulated proteins were predominantly associated with cell adhesion and synaptic organization (Cluster 1), neddylation and protein metabolism (Clusters 2–3), and nuclear import and translational control (Clusters 4–5) (Figure 6D). Together, these findings suggest that *M. tuberculosis* infection reduces EV-associated proteins linked to neuronal structure, synaptic function, and intracellular homeostatic pathways, consistent with disruption of these processes in the infected brain.

To provide biological context for these pathway-level decreases, we examined several representative proteins that were significantly reduced in EVs from infected brains and are known to support neuronal structure and neuroprotection. SORL1, an endosomal sorting receptor implicated in amyloid precursor trafficking and Alzheimer’s disease, was decreased, consistent with endosomal dysfunction seen in neurodegeneration [58]. Given that reduced PITRM1 activity has been linked to amyloid accumulation and Alzheimer’s-like pathology in animal models [59], this decrease is notable in the context of amyloid deposition observed in our rabbit model of TB meningitis [16]. In addition, reduced 3-hydroxy-3-methylglutaryl-CoA synthase 1 (HMG-CoA synthase 1; HMGCS1) suggests dysregulation of cholesterol biosynthesis and membrane lipid homeostasis, processes essential for neuronal maintenance, myelination, and repair [60]. Together, these changes highlight a depletion of EV-associated proteins involved in neuroprotection, potentially reflecting infection-induced brain injury [3,4].

### 3.8. Cell-Specific EV Protein Changes During Infection

Intrigued by the CellMarker 2024 cell-type enrichment data, we classified differentially expressed brain-derived EV proteins according to their cell type–associated annotations. This analysis revealed distinct patterns across neurons, astrocytes, and microglia (Figure 7A). While several proteins associated with microglia and astrocytes were elevated in EVs from infected rabbits, a substantial number of neuron-associated proteins, including NCAM1, SYT1, and GRIA1, were significantly reduced. These trends indicate reduced representation of neuron-associated proteins, alongside increased representation of several microglial- and astrocyte-associated proteins, during infection.

Next, we used Western blot analysis to validate selected candidates from the proteomic analysis, including cell type–associated proteins (Figure 7B–E and Figure S9). ATP1A3 (a neuronal ion transporter) and GFAP (an astrocytic intermediate filament protein) were detected at similar levels across both conditions, indicating no significant changes in their EV representation (Figure S9A,B). Likewise, the microglial marker TMEM119 remained stable between groups, as did GCPII, a glia-associated protein, suggesting that these glial EV proteins were preserved at this stage of infection (Figure S9C,D). Glutaminase (GLS) was also prioritized for validation because it catalyzes the conversion of glutamine to glutamate, a key pathway implicated in neuronal excitotoxicity during TB meningitis [9,10]. In homeostatic conditions, GLS is predominantly expressed in neurons, but it is upregulated in activated microglia during neuroinflammation [61]. Consistent with the proteomic findings, GLS was significantly increased in EVs from infected brains (*p* = 0.037; Figure 7B,C). Conversely, NCAM1, a neuronal adhesion molecule, showed a reduction in EVs from infected brains, aligning with the proteomic trend, although the change did not reach statistical significance (Figure 7D,E). Together, these findings suggest a potential remodeling of the EV-associated protein profile during *M. tuberculosis* infection, characterized by a relative loss of certain neurostructural proteins and gain of metabolism-associated or stress-related proteins.

### 3.9. Brain-Derived EVs Contain Both Surface-Associated and Internal Proteins

To evaluate the sub-vesicular localization and EV association of key candidates, we performed PK protection assays on selected proteins from brain-derived EVs (Figure 8A,B). In this assay, surface-associated EV proteins are degraded by PK, whereas proteins encapsulated within the vesicle lumen remain protected unless the membrane is permeabilized by detergent (i.e., Triton X-100). Dot blot analysis of EVs from uninfected rabbits revealed that the microglial markers CD11b and TMEM119, as well as the astrocyte marker GFAP and the metabolic enzyme GAPDH, were degraded following PK treatment even in the absence of detergent (Figure 8C). These results suggest that these proteins are predominantly surface-associated or loosely associated with the EV membrane. In contrast, ATP1A3, NCAM1, GCPII, and GLS were resistant to PK in the absence of detergent but were degraded upon permeabilization of the EV membrane with Triton X-100 (Figure 8D). These findings indicate that these proteins are predominantly localized within the EV lumen or protected by the EV membrane. A similar protection pattern was observed in EVs isolated from infected rabbit brain tissue, suggesting that infection does not alter sub-vesicular localization of these proteins. Together, these data demonstrate that brain-derived EVs contain a mixture of internal and surface-exposed protein markers and identify several surface-associated proteins as potential candidate targets for future cell-specific EV isolation and validation studies. The PK protection assay was used to define the sub-vesicular topology of selected representative targets, rather than to comprehensively validate the full untargeted proteomic dataset.

## 4. Discussion

EVs are increasingly recognized as active participants in infection, capable of transporting molecular cargo that can propagate inflammatory signaling and contribute to disease progression, encompassing both pathogen-derived and host-derived EVs [62]. This concept is well supported in pulmonary TB, where EVs from biofluids and immune cell cultures show infection-induced changes in EV cargo [63,64,65,66,67]. In TB meningitis, a proof-of-concept study demonstrated that urine-derived EVs can carry *M. tuberculosis* DNA detectable by PCR [68]. However, infection-associated changes in EV cargo within the CNS remain poorly defined. Here, we provide the first evidence that *M. tuberculosis* infection alters the proteomic composition of brain-derived EVs, revealing signatures consistent with key features of TB meningitis pathobiology. These data establish a foundation for future biomarker studies and mechanistic work on EV-mediated CNS host–pathogen interactions.

Methodologically, we extend the “EV-release” workflow to rabbit brain tissue. This approach has been shown to recover EV-enriched fractions with preserved morphology while minimizing cellular damage and intracellular contamination from mechanical or enzymatic dissociation [29]. Our pilot comparison of fresh and flash-frozen tissue from the same animal demonstrated the technical feasibility of recovering EVs from frozen tissue, consistent with prior reports [69,70]. This provides a practical framework for future biobanked tissue studies and is particularly advantageous in BSL-3 environments, where immediate processing is often impractical.

We found that TB meningitis drives both quantitative and qualitative changes in brain-derived EVs. While *M. tuberculosis* infection significantly increased EV concentration, EV size and morphology remained unchanged compared to uninfected controls. Notably, the increase in particle number was not matched by a proportional increase in total EV-associated protein, suggesting heterogeneous EV remodeling rather than uniform upregulation of EV protein. This discrepancy likely reflects shifts in EV subtype distribution, altered protein loading, or contributions from co-isolated NVEPs.

Proteomic analysis revealed a striking shift in cell type–associated EV signatures during *M. tuberculosis* infection. EVs from infected brains showed enrichment of microglia- and astrocyte-associated proteins, while neuron-associated proteins were depleted. These findings align with previous neuropathological characterization of this model, which demonstrated robust microglial activation by immunohistochemistry and PET imaging, increased microglial density, and evidence of neuronal injury, including amyloid deposition, white matter loss, and neurobehavioral deficits [11,12,16]. This enrichment of glial-associated EV protein signatures is consistent with known patterns in other neuroinflammatory conditions. In models of cerebral ischemia, Alzheimer’s disease, and viral encephalitis, proinflammatory stimuli increase EV release by microglia and astrocytes, with glial-derived EVs propagating disease-associated signaling [21,22,71,72,73,74,75]. Similarly, the depletion of neuron-associated proteins, also reported in Alzheimer’s disease and traumatic brain injury [76], suggests reduced neuronal EV contribution during infection. Collectively, these data suggest that *M. tuberculosis* infection remodels the brain EV landscape toward glial- and immune-associated protein signatures, consistent with known cellular and pathological changes in TB meningitis, consistent with the biological relevance of the EV proteomic signature.

However, proteomic enrichment does not definitively establish cellular origin. While our findings reveal cell type–associated patterns, in silico cell-type enrichment analyses are insufficient to establish lineage specificity, nor do proteomic patterns alone confirm activation of functional pathways. To address this limitation, we used PK protection assays to distinguish surface-associated from intraluminal proteins, thereby identifying candidate surface-accessible markers that may be useful for future cell-specific EV enrichment approaches. These findings provide a starting point for developing immunocapture approaches to isolate discrete EV subpopulations. Validating these cell-specific markers in peripheral biofluids (plasma or CSF) will be a critical next step for determining whether the brain-derived EV signatures identified here can be translated to clinically accessible samples.

In our study, TMEM119, CD11b, GFAP, and GAPDH were protease-sensitive, consistent with surface exposure. Although GAPDH is cytosolic, its protease accessibility is consistent with prior reports of noncanonical surface display or corona adsorption on EVs [77]. Conversely, ATP1A3, NCAM1, GCPII, and GLS were protease-resistant, consistent with intraluminal or membrane-protected localization. Notably, NCAM1 and ATP1A3 have been reported on EV surfaces in prior studies [78,79], but our findings suggest they are intraluminal in TB meningitis. These discrepancies may reflect differences in EV subtypes (e.g., endosome-derived small EVs or microvesicles), species- or brain region-specific effects, or infection-associated post-translational modifications.

At the proteomic level, infection was associated with broad remodeling of EV-associated proteins, characterized by enrichment of immune and stress-response pathways and depletion of neuronal and neuroprotective processes. Upregulated proteins were associated with innate immune activation, vesicle trafficking, metabolic regulation, and neurodegenerative processes. Infection-specific signatures were particularly enriched in proteins associated with microglial activation, IFN response, inflammasome activation, and host defense against TB. Conversely, downregulated proteins included those involved in synaptic function, axonal guidance, mitochondrial function, cholesterol metabolism, and neurodevelopment. Our previous work in this model demonstrated that cholesterol metabolism is dysregulated in the infected brain [80], but whether the altered EV-associated lipid signatures simply reflect these broader changes or contribute to their propagation remains to be determined.

Several apolipoproteins and plasma-associated proteins detected in these EV-enriched fractions warrant careful interpretation. Proteins such as ApoA1 and ApoB are commonly associated with lipoprotein co-isolation and may reflect the presence of NVEPs. However, not all detected apolipoproteins are necessarily contaminants. ApoE and clusterin (CLU/ApoJ) have well-established roles in CNS lipid transport and neuroinflammation and have been associated with CNS-derived EVs [75,81,82,83]. In the context of TB meningitis, where blood-brain barrier disruption and neuroinflammation are prominent features of disease, their detection may reflect underlying pathological processes rather than simple peripheral contamination. Similarly, the increased abundance of plasma-associated proteins (e.g., surfactant protein D and IgM heavy chain) in infected samples may be consistent with infection-associated barrier compromise. Therefore, although these proteins cannot be definitively assigned to EVs and may partially reflect co-isolated material, their presence may also provide biologically relevant insight into disease-associated changes in the CNS.

Comparative proteomics and Western blot validation identified GLS enrichment in EVs from infected brains, highlighting EV-associated GLS as a candidate link between altered glutamine metabolism, neuroinflammation, and excitotoxic injury. Beyond its established role in glutamate synthesis, GLS upregulation has been linked to microglial activation and altered EV cargo dynamics in other CNS diseases (e.g., Japanese encephalitis virus, Rett syndrome) [21,72,74,84,85,86]. Given prior evidence of glutamate excitotoxicity in TB meningitis [9,10,87], the enrichment of EV-associated GLS suggests that vesicle remodeling may intersect with altered CNS glutamate homeostasis during infection. However, these observations remain hypothesis-generating, as our data do not establish the cellular origin or a causative functional role for EV-associated GLS. Targeted functional validation is needed to determine whether EV-associated GLS directly contributes to excitotoxicity, inflammatory signaling, or metabolic reprogramming in TB meningitis.

Several limitations should be considered when interpreting these findings. Our animal model uses direct intracerebral inoculation, which provides reproducible experimental conditions but bypasses the natural hematogenous dissemination of *M. tuberculosis* to the brain. Additionally, the ex vivo tissue incubation may alter EV recovery or cargo composition relative to the in vivo state. Furthermore, our isolates represent EV-enriched fractions rather than fully purified EV populations, as SEC does not completely separate EVs from co-isolated NVEPs, lipoproteins, soluble protein complexes, or intracellular components released during tissue damage. However, because these conditions apply equally across both experimental groups, comparative analyses and identified relative differences remain valid. Beyond isolation parameters, in silico cell-type enrichment and proteomic abundance patterns reflect candidate protein signatures rather than definitive cellular lineage or active pathway signaling. Except for the selected proteins evaluated by PK protection assays, the precise topology and vesicular localization of individual proteomic candidates remain unconfirmed. Our proteomic discovery cohort was relatively small (*n* = 5 per group), and functional annotation relied on human ortholog mapping due to sparse *Oryctolagus cuniculus* databases, introducing potential cross-species annotation bias. Downstream validation was similarly constrained by a scarcity of validated, rabbit-specific antibodies for canonical EV markers. Finally, this study focused strictly on the proteomic atlas of brain tissue-derived fractions without evaluating vesicular microRNAs or biofluid compartments. Consequently, all findings regarding cell-of-origin, pathway activation, and candidate biomarkers must be considered hypothesis-generating. Future studies utilizing density-gradient purification, cell-specific immunoaffinity capture, transcriptomic profiling (e.g., miRNA-seq), and biofluid validation (e.g., CSF and plasma) in larger cohorts will be essential to establish causality and translational utility.

## 5. Conclusions

In summary, *M. tuberculosis* infection drives broad proteomic remodeling of brain-derived EVs across immune, metabolic, and neuronal pathways, providing a comprehensive proteomic resource for TB meningitis and a framework for future investigation into EV-mediated pathology in CNS infection. These EV-associated signatures align closely with the known pathobiology of TB meningitis, suggesting that EV-associated protein signatures reflect biologically relevant host–pathogen interactions within the CNS. Future studies will be essential to validate EV marker accessibility across species and disease contexts, as well as to determine whether the candidate surface-associated proteins identified here can be leveraged to isolate cell-specific EV subpopulations from accessible biofluids. Combining immunocapture approaches with single-vesicle technologies may enable more precise characterization of neuronal, microglial, and astrocytic EV populations and their associated cargo. Ultimately, functional studies will be required to determine whether the EV signatures identified here primarily reflect underlying CNS pathology, actively contribute to disease progression, or both. As the first characterization of brain-derived EVs in an in vivo model of TB meningitis, these findings establish a foundational atlas of infection-associated EV alterations and support continued investigation of EVs as both readouts of and potential modulators of CNS disease processes.

## Figures and Tables

**Figure 1:**
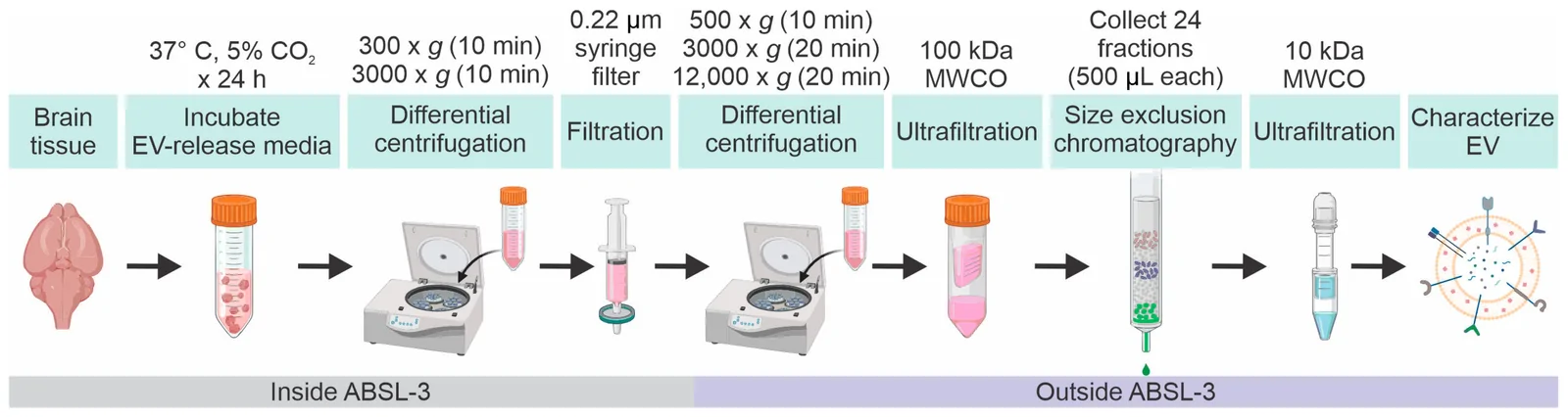
Workflow for the isolation of EVs from brain tissue. EV isolation steps for rabbit brain tissue performed inside and outside the ABSL-3 facility. Black arrows indicate sequential workflow steps and fluid transfers. The bottom bar indicates steps performed inside the ABSL-3 facility (grey) and outside the ABSL-3 facility (purple). The schematic was created using BioRender and CorelDRAW 2022.

**Figure 2:**
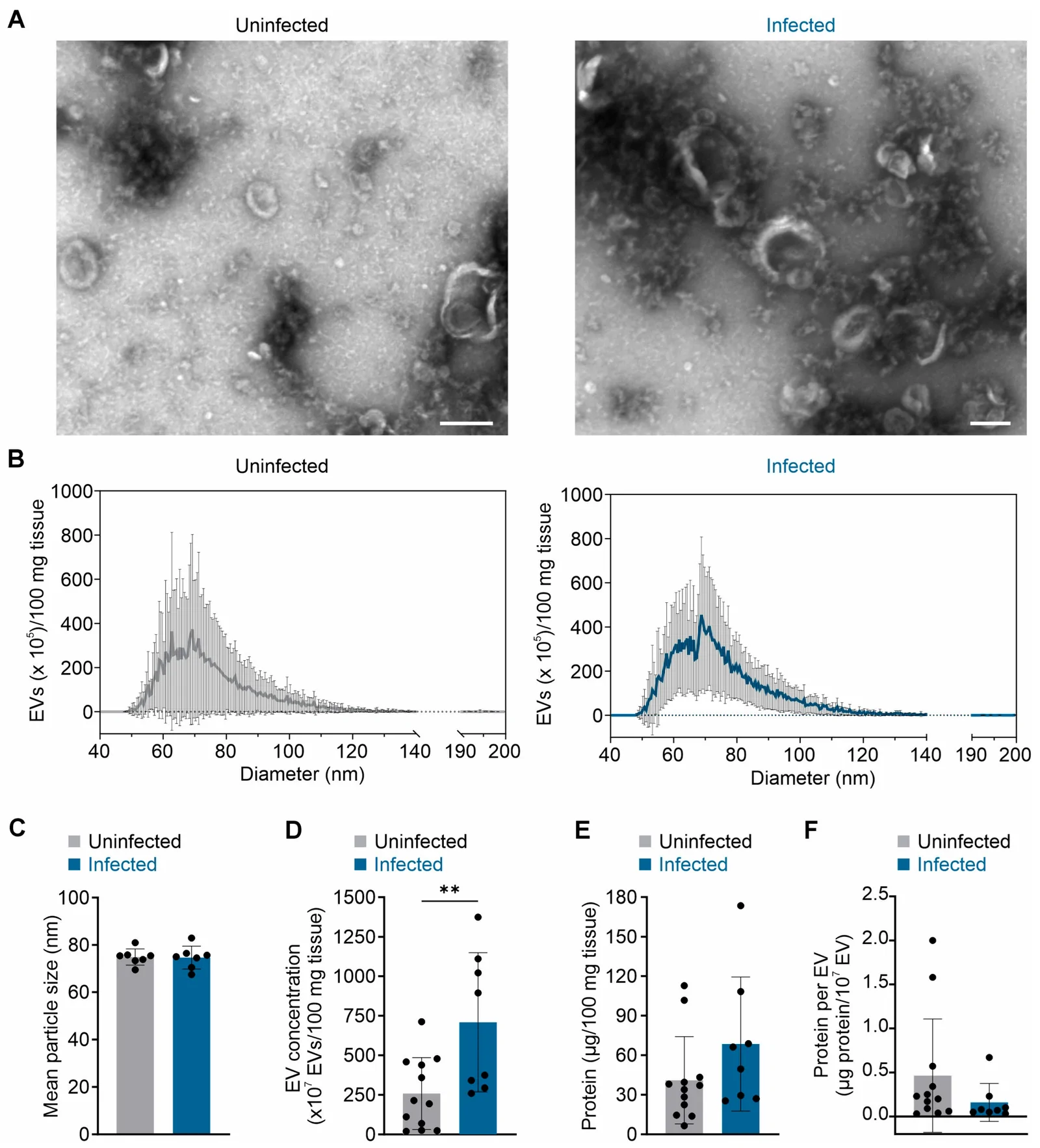
Characterization of brain-derived EVs from young rabbits with TB meningitis and age-matched healthy controls. EVs were isolated from brain tissue collected from young rabbits 21 days after injection with *M. tuberculosis* (infected) or PBS (uninfected). (**A**) Representative negative-stain transmission electron microscopy (TEM) images of brain-derived EVs from uninfected (left) and infected (right) rabbits. Scale bar = 200 nm. *n* = 1 animal per group. (**B**) EV size distribution determined by Nanoflow cytometry (NanoFCM) for uninfected (left) and infected (right) samples. *n* = 7 animals per group. (**C**) Mean EV particle size measured by NanoFCM. *n* = 7 animals per group. (**D**) EV concentration measured by NanoFCM and normalized to 100 mg of brain tissue. *n* = 8–12 animals per group. (**E**) Total protein concentration measured by BCA. *n* = 8–12 animals per group. (**F**) Protein concentration normalized to EV concentration measured by NanoFCM. *n* = 8–12 animals per group. Data are represented as mean ± standard deviation (SD). Statistical comparisons were made using an unpaired two-tailed *t*-test. ** *p* < 0.01.

**Figure 3:**
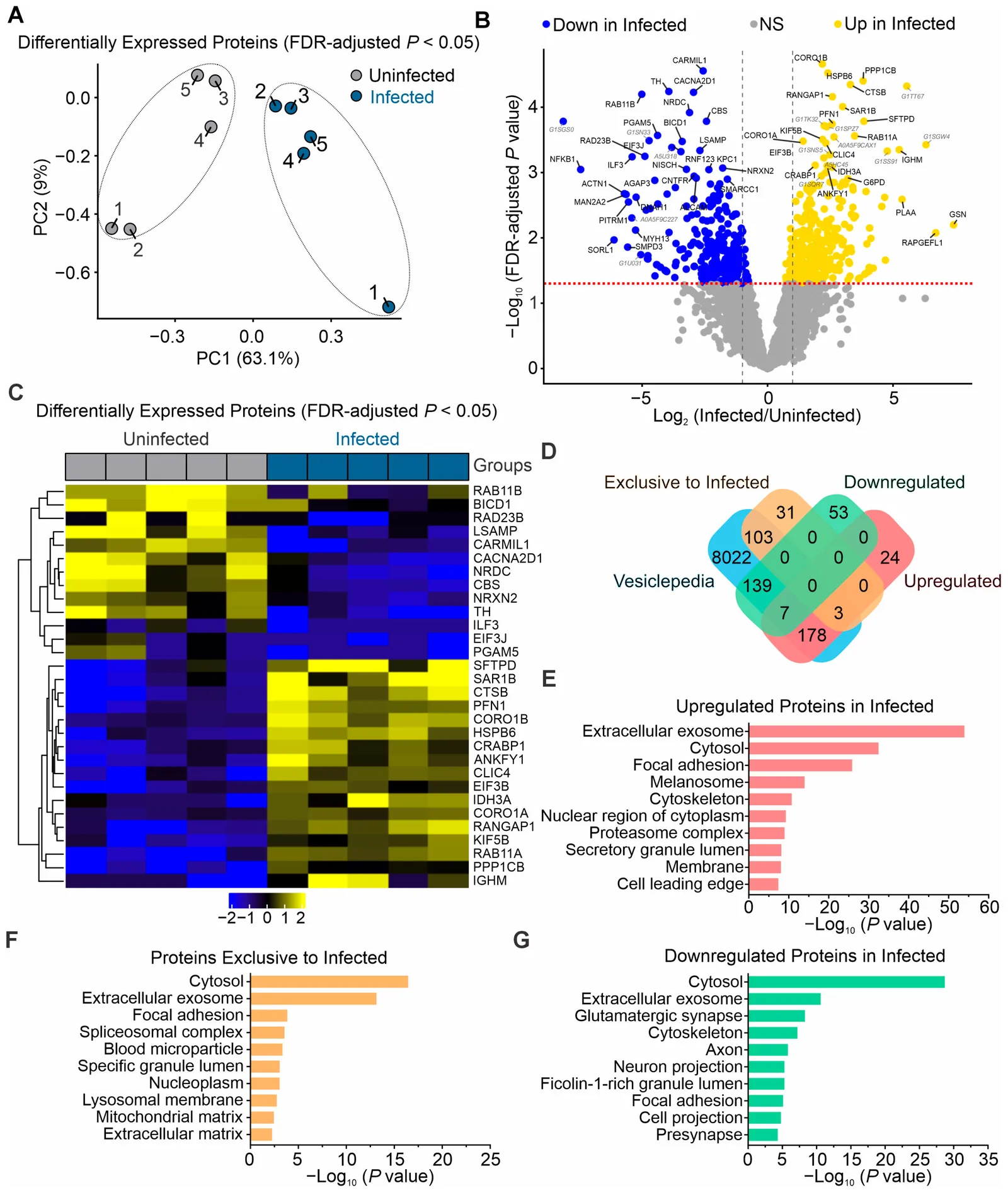
Protein profiling and differential expression analysis of brain-derived EVs. (**A**) Principal Component Analysis (PCA) of significantly differentially expressed proteins (FDR-adjusted *p* < 0.05), showing clear separation between uninfected control and *M. tuberculosis*-infected (i.e., infected) EV samples. Each dot represents an individual animal. (**B**) Volcano plot of differentially expressed proteins. Yellow and blue points denote proteins significantly upregulated or downregulated, respectively, in infected EVs compared to uninfected (FDR-adjusted *p* < 0.05 and |log_2_FC| > 1). Gray points denote proteins that did not meet significance thresholds (NS). Selected proteins with the largest fold changes and/or lowest adjusted *p*-values are labeled. (**C**) Heatmap of the top 30 significantly altered proteins (FDR-adjusted *p*-value < 0.05), showing log2-transformed, median-centered abundance values across individual samples. Rows represent proteins and columns represent individual animals. Proteins were hierarchically clustered based on expression patterns (left dendrogram). The color scale indicates relative abundance, with yellow representing higher abundance and blue representing lower abundance. (**D**) Venn diagram showing the overlap between proteins that were upregulated (red, corresponding to panel (**E**)), exclusively detected in infected (orange, corresponding to panel (**F**)), and downregulated (green, corresponding to panel (**G**)), in brain-derived EVs, and known EV proteins cataloged in the Vesiclepedia database (blue). Numbers within regions indicate protein counts. The analysis was performed using FunRich. (**E**–**G**) Cellular compartment enrichment analysis of upregulated, downregulated, and infection-specific proteins, respectively, using the Database for Annotation, Visualization and Integrated Discovery (DAVID). The top 10 enriched cellular compartment terms, ranked by FDR-adjusted *p*-value, are displayed. All analyses in this figure were derived from proteomic data obtained from *n* = 5 animals per group. All *p*-values were adjusted for multiple comparisons using the Benjamini-Hochberg procedure.

**Figure 4:**
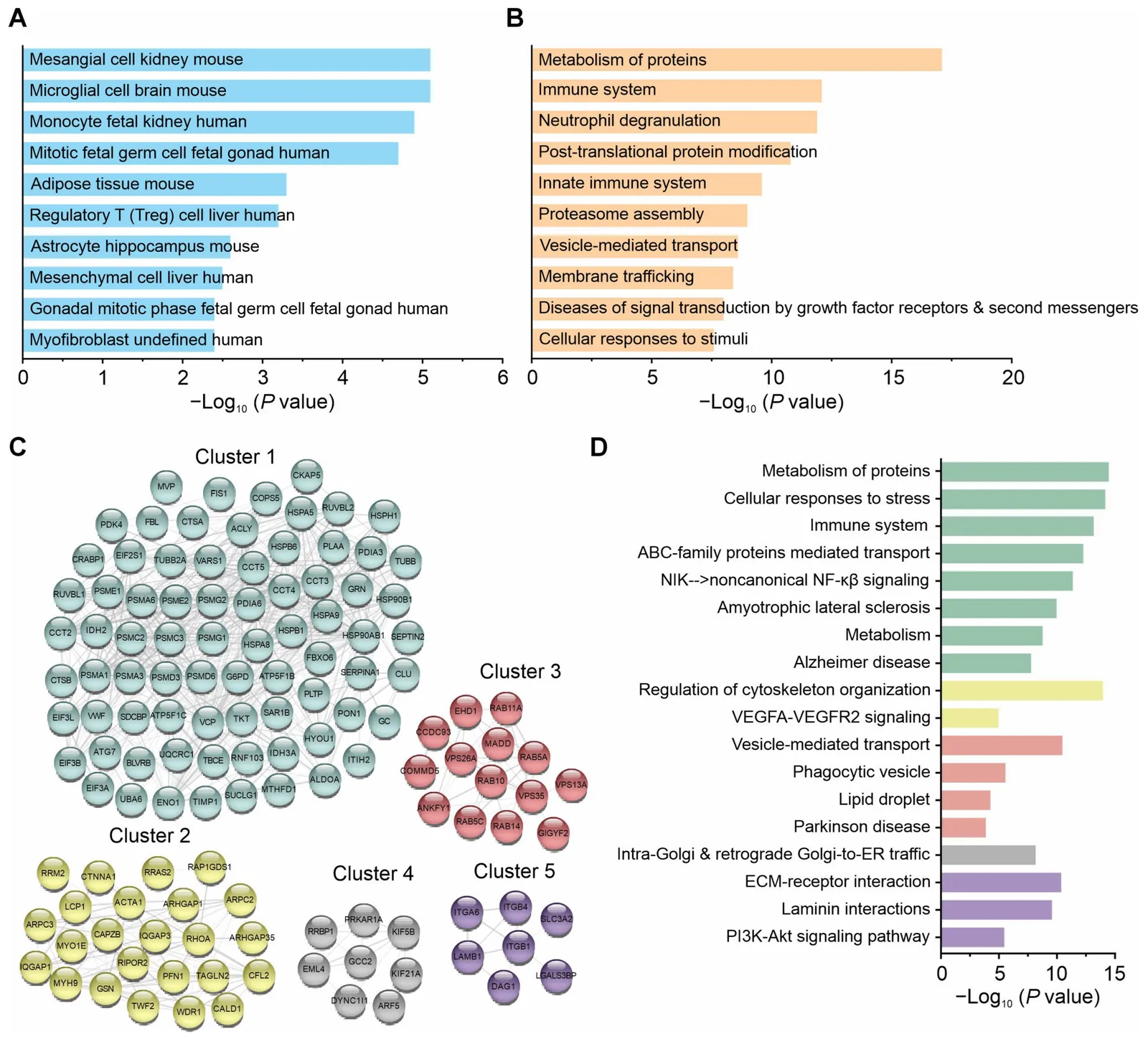
Cell-type marker enrichment and pathway analysis of proteins upregulated in brain-derived EVs during *M. tuberculosis* infection. All analyses were performed using proteomic data derived from *n* = 5 animals per group. Functional enrichment analyses were conducted after mapping rabbit proteins to human orthologs. (**A**) Top 10 significantly enriched cell-type markers identified using Enrichr CellMarker 2024 for proteins upregulated in brain-derived EVs during *M. tuberculosis* infection, ranked by adjusted *p*-value. (**B**) Top 10 significantly enriched pathways identified using Enrichr Reactome Pathways 2024 for proteins upregulated in infection, ranked by adjusted *p*-value. (**C**) STRING protein-protein interaction network clusters of proteins upregulated in infection. Nodes represent individual proteins, and edges indicate predicted functional associations. Background colors denote distinct protein-interaction clusters and correspond to the bar colors used in (**D**). An enlarged version is available in Figure S6. (**D**) STRING functional enrichment analysis of the clusters identified in (**C**), highlighting the major biological processes and pathways enriched within each cluster, ranked by adjusted *p*-value.

**Figure 5:**
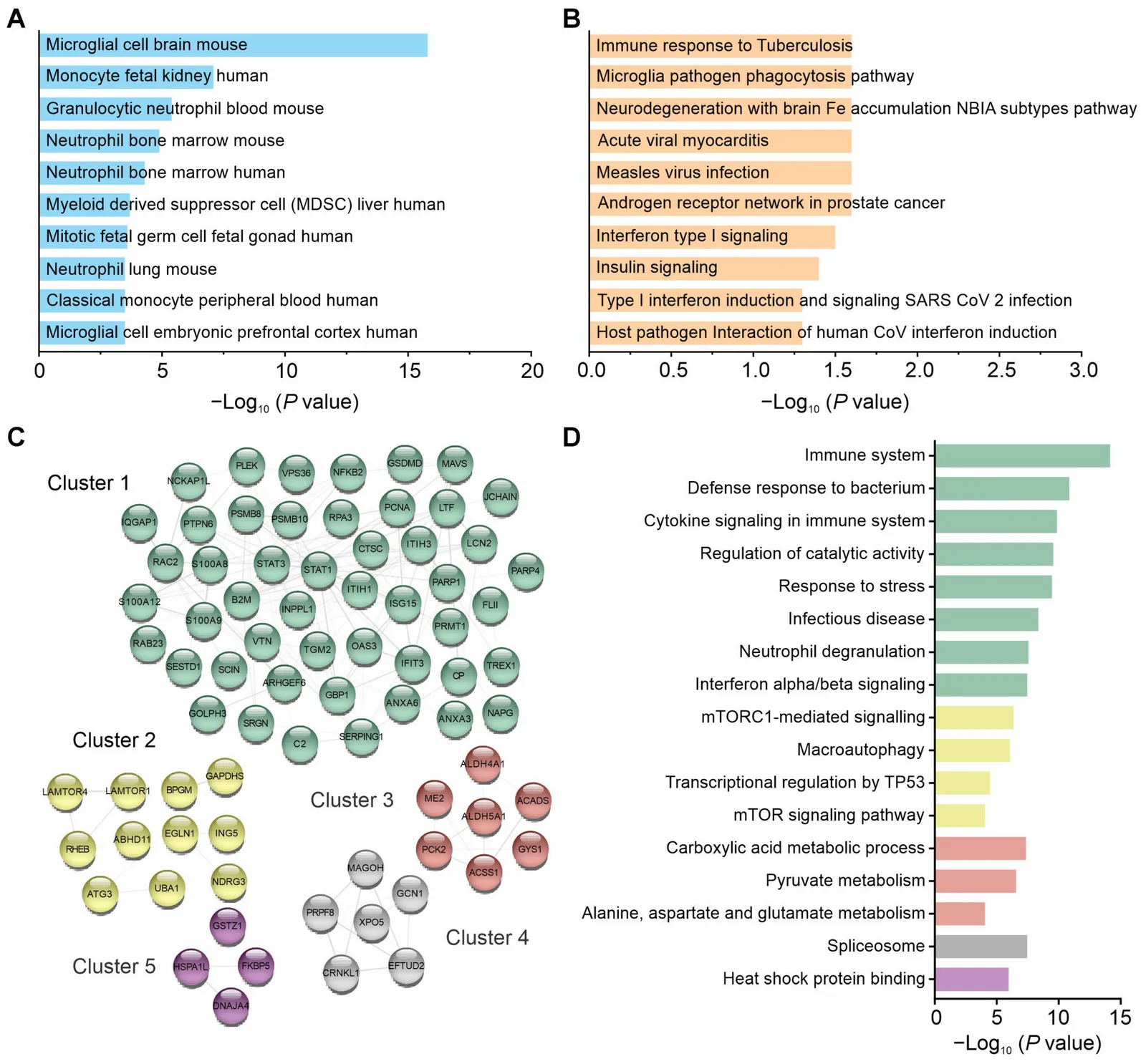
Cell-type marker enrichment and pathway analysis of proteins detected exclusively in brain-derived EVs during *M. tuberculosis* infection. All analyses were performed using proteomic data derived from *n* = 5 animals per group. Functional enrichment analyses were conducted after mapping rabbit proteins to human orthologs. (**A**) Top 10 significantly enriched cell-type markers identified using Enrichr CellMarker 2024 for proteins detected exclusively in brain-derived EVs during *M. tuberculosis* infection (infection-specific proteins), ranked by adjusted *p*-value. (**B**) Top 10 significantly enriched pathways identified using Enrichr WikiPathways 2024 for infection-specific proteins, ranked by adjusted *p*-value. (**C**) STRING protein-protein interaction network clusters of infection-specific proteins. Nodes represent individual proteins, and edges indicate predicted functional associations. Background colors denote distinct protein-interaction clusters and correspond to the bar colors used in (**D**). An enlarged version is available in Figure S7. (**D**) STRING functional enrichment analysis of the clusters identified in (**C**), highlighting the major biological processes and pathways enriched within each cluster, ranked by adjusted *p*-value.

**Figure 6:**
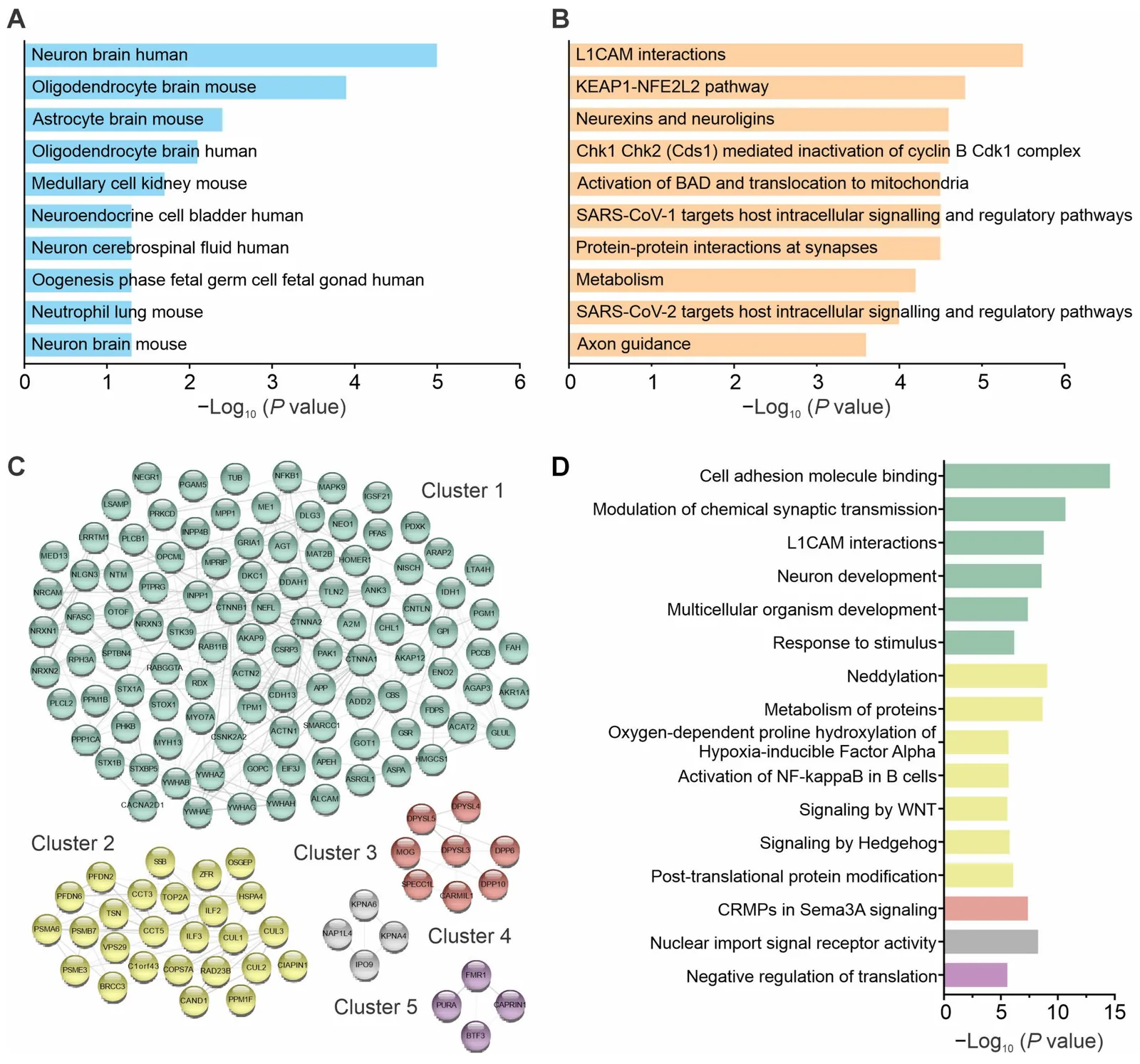
Cell-type marker enrichment and pathway analysis of proteins downregulated in brain-derived EVs during *M. tuberculosis* infection. All analyses were performed using proteomic data derived from *n* = 5 animals per group. Functional enrichment analyses were conducted after mapping rabbit proteins to human orthologs. (**A**) Top 10 significantly enriched cell-type markers identified using Enrichr CellMarker 2024 for proteins downregulated in brain-derived EVs during *M. tuberculosis* infection, ranked by adjusted *p*-value. (**B**) Top 10 significantly enriched pathways identified using Enrichr Reactome Pathways 2024 for proteins downregulated in infection, ranked by adjusted *p*-value. (**C**) STRING protein-protein interaction network clusters of proteins downregulated in infection. Nodes represent proteins, and edges indicate predicted functional interactions based on known associations. Background colors denote distinct protein-interaction clusters and correspond to the bar colors used in (**D**). An enlarged version is available in Figure S8. (**D**) STRING functional enrichment analysis of the clusters identified in (**C**), highlighting the major biological processes and pathways enriched within each cluster, ranked by adjusted *p*-value.

**Figure 7:**
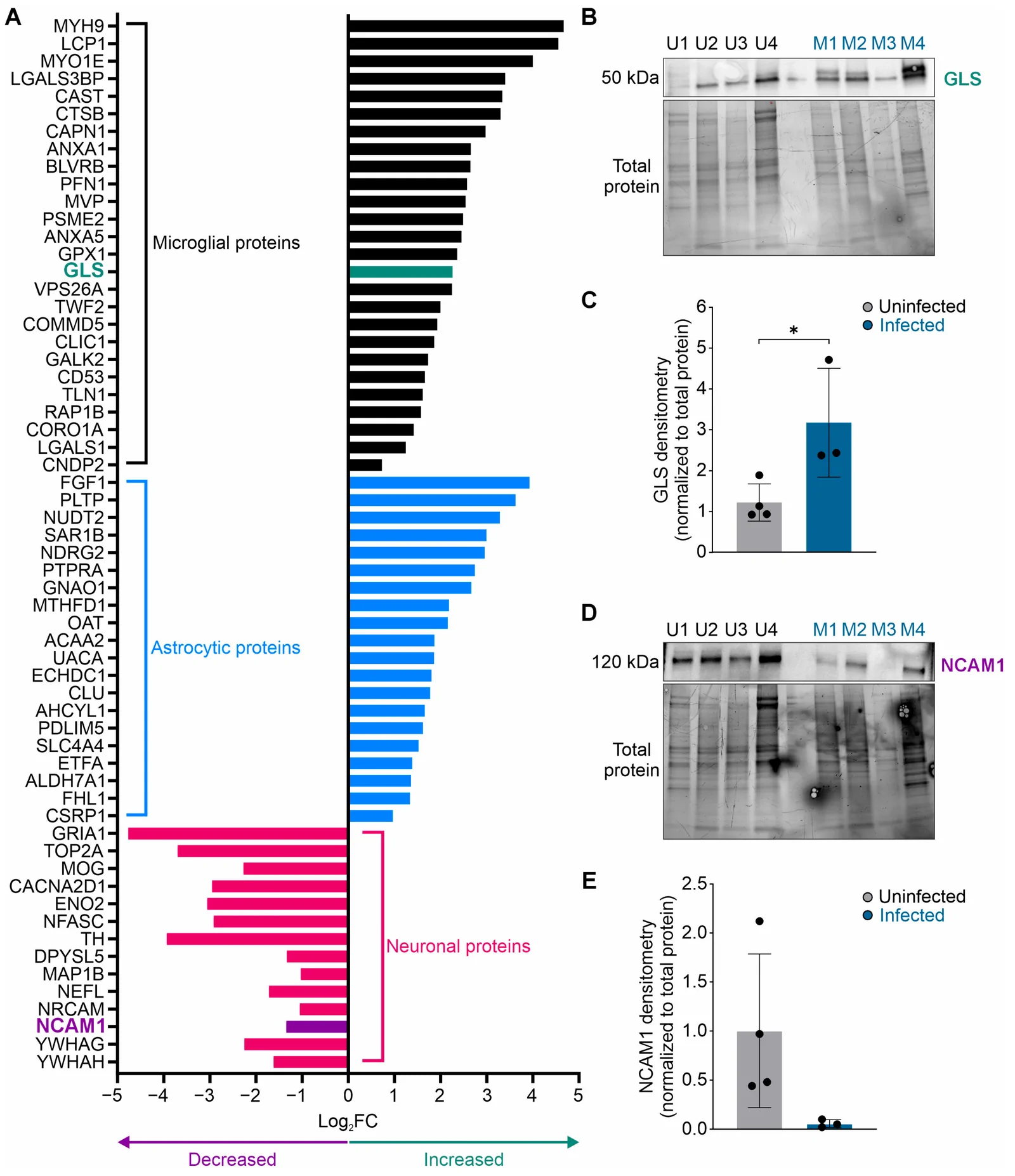
Differential expression and validation of cell type–associated proteins in brain-derived EVs. (**A**) Log2 fold change (Log2FC) of brain-derived EV proteins grouped according to cell type–associated protein enrichment based on Enrichr CellMarker 2024 annotations. This analysis was performed using proteomic data derived from *n* = 5 animals per group following rabbit-to-human ortholog mapping. Proteins associated with microglia (black), astrocytes (blue), and neurons (pink) are shown. Positive values indicate increased protein abundance in brain-derived EVs during *M. tuberculosis* infection relative to uninfected controls. GLS and NCAM1 are highlighted in teal and purple, respectively, to indicate the proteins selected for Western blot validation in panels (**B**–**E**), corresponding to increased and decreased protein abundance, respectively. (**B**–**E**) Western blot validation of selected proteins identified in the proteomic dataset and corresponding densitometry quantification of uninfected control (U#, gray) and infected samples (M#, blue). All protein levels were normalized to total protein loading. *n* = 3–4 animals per group (M3 was excluded from analysis because of inadequate protein loading). (**B**,**C**) GLS was significantly increased in brain-derived EVs from infected rabbits (*p* < 0.05). (**D**,**E**) NCAM1 was reduced during infection, although the difference did not reach statistical significance. Data are presented as mean ± SD from biologically independent samples. Statistical comparisons were made using an unpaired two-tailed *t*-test. * *p* < 0.05.

**Figure 8:**
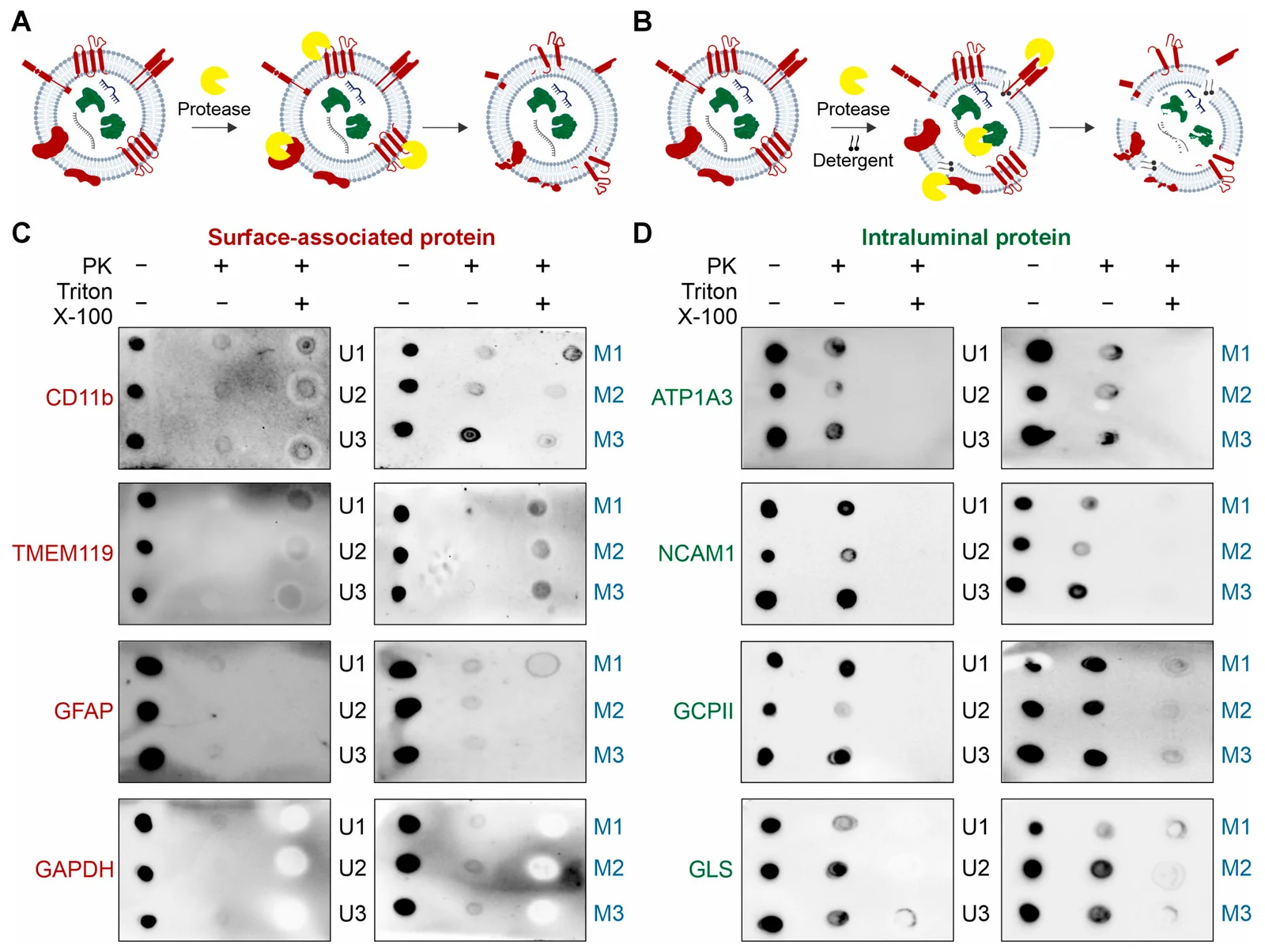
Proteinase K protection assay reveals differential localization of EV-associated proteins in brain-derived EVs. (**A**,**B**) Schematic of the proteinase K (PK) protection assay workflow. Arrows indicate the direction of the experimental workflow. Protein labels are shown in red for surface-associated proteins (**C**) and green for intraluminal or membrane-protected proteins (**D**). (**A**) Treatment of intact EVs with protease (PK) selectively digests surface-exposed proteins, while intraluminal proteins remain membrane-protected and intact. (**B**) Co-treatment with detergent (Triton X-100) permeabilizes EV membranes, enabling PK-mediated degradation of otherwise protected proteins. PK activity was inhibited with phenylmethylsulfonyl fluoride (PMSF) prior to downstream analysis. (**C**,**D**) Dot blot analysis of brain-derived EVs from uninfected control (U#, black) and *M. tuberculosis*-infected (M#, blue) rabbits treated with PK alone, PK plus Triton X-100, or left untreated. *n* = 3 animals per group; equal protein was loaded per dot. (**C**) CD11b, TMEM119, GFAP, and GAPDH were digested by PK in both the presence and absence of detergent, indicating that they are exposed to PK and likely surface-associated EV proteins. (**D**) ATP1A3, NCAM1, GCPII, and GLS were preserved following PK treatment alone but degraded upon detergent permeabilization, consistent with intraluminal or membrane-protected localization. The schematic was created using BioRender and CorelDRAW 2022.

**Table 1:** List of primary antibodies.

	Primary Antibody	Company	Catalog Number	Western Blot Dilution	Dot Blot Dilution
EV-positive markers	ALIX	Thermo Fisher Scientific	MA3-32773	[1:1000]	-
EV-positive markers	Flotillin-1	Thermo Fisher Scientific	MA5-35364	[1:1000]	-
EV-positive markers	Hsp70	Abcam (Waltham, MA, USA)	Ab5439	[1:1000]	-
EV-positive markers	GAPDH	Thermo Fisher Scientific	MA5-15738-HRP	-	[1:500]
Cell-specific markers	GFAP	Abcam	Ab4674	[1:2000]	[1:500]
Cell-specific markers	TMEM119	Thermo Fisher Scientific	Cat# 66948-1	[1:500]	[1:500]
Cell-specific markers	GCPII	GeneTex (Irvine, CA, USA)	GTX80151	[1:1000]	[1:500]
Cell-specific markers	NCAM1	Biolegend (San Diego, CA, USA)	Cat# 304602	[1:500]	[1:500]
Cell-specific markers	ATP1A3	Thermo Fisher Scientific	MA3-915	[1:1000]	[1:500]
Cell-specific markers	CD11b	Biolegend	Cat. 101212	-	[1:500]
Cell-specific markers	GLS	Thermo Fisher Scientific	6H5HK15	[1:500]	[1:500]

**Table 2:** List of secondary antibodies.

Secondary Antibody	Company	Catalog Number	Dilution
Goat anti-mouse	Thermo Fisher Scientific	Cat# 31431	[1:10,000]
Goat anti-chicken	Thermo Fisher Scientific	A16054	[1:20,000]
Donkey anti-goat	Thermo Fisher Scientific	PA1-28664	[1:10,000]
Goat anti-rabbit	Vector Labs (Newark, CA, USA)	BA-1000-1.5	[1:5000]

## Data Availability

All data are available in the main text or the Supplementary Materials. The mass spectrometry proteomics data have been deposited in the ProteomeXchange Consortium via the PRIDE [88] partner repository with the dataset identifier PXD071017 and 10.6019/PXD071017. We have submitted all relevant data from our experiments to the EV-TRACK knowledgebase (EV-TRACK ID: EV260011) [89].

## Supplementary Materials

The following supporting information can be downloaded at: https://www.mdpi.com/article/10.3390/cells15191749/s1, Table S1: Proteomic dataset of brain-derived EVs; Table S2: Rabbit-to-human ortholog mapping; Figure S1: Brain-derived EVs from healthy adult rabbits using fresh and flash-frozen tissue processing; Figure S2: Characterization of brain-derived EV isolation from 1-week-old rabbits; Figure S3: Body weight during infection incubation; Figure S4: Brain-derived EV concentration based on sex; Figure S5: Expression of canonical EV markers; Figure S6: Enlarged STRING protein-protein interaction network clusters of upregulated proteins in brain-derived EVs during *M. tuberculosis* infection; Figure S7: Enlarged STRING protein-protein interaction network clusters of infection-specific proteins; Figure S8: Enlarged STRING protein-protein interaction network clusters of downregulated proteins in brain-derived EVs during *M. tuberculosis* infection; Figure S9: Expression of cell type–associated proteins in brain-derived EVs.

## Abbreviations

The following abbreviations are used in this manuscript

TB	tuberculosis
TB meningitis	tuberculous meningitis
EV	extracellular vesicle
CNS	central nervous system
CSF	cerebrospinal fluid
GFAP	glial fibrillary acidic protein
LC-MS/MS	liquid chromatography-tandem mass spectrometry
NanoFCM	nanoflow cytometry
TEM	transmission electron microscopy
SEC	size-exclusion chromatography
BCA	bicinchoninic acid
PBS	phosphate-buffered saline
PK	proteinase K
PMSF	phenylmethylsulfonyl fluoride
BSL-3/ABSL-3	biosafety level-3/Animal biosafety level-3
DAVID	Database for Annotation, Visualization, and Integrated Discovery
GO-CC	Gene Ontology Cellular Component
PCA	principal component analysis
FDR	false discovery rate
NSAF	normalized spectral abundance factor
GLS	glutaminase
