# Supplementary material for: Tuberculous Meningitis Alters the Proteomic Landscape of Brain-Derived Extracellular Vesicles
Source: Cells. 2026 Sep 25;15(19):1749. doi: 10.3390/cells15191749 (PMC13664519; doi:10.3390/cells15191749)
Supplement: Supplementary file 1 [file cells-15-01749-s001.zip › cells-4546064-supplementary.pdf]

SUPPLEMENTAL MATERIAL

Tuberculous meningitis alters the proteomic landscape of brain-derived extracellular vesicles

Blanca V. Rodriguez[1,2], Nerketa N. L. Damiba[1,2], Harshita Tak[1], Nicole Beaubien[1], Daisy Puca[1], D. Brian Foster[3], Samarjit Das[1], Elizabeth W. Tucker[1,2*]

[1]Department of Anesthesiology and Critical Care Medicine, Johns Hopkins University School of Medicine, Baltimore, MD 21287, USA

[2]Center for Tuberculosis Research, Johns Hopkins University School of Medicine, Baltimore, MD 21287, USA

[3]Department of Medicine, Division of Cardiology, Johns Hopkins University School of Medicine, Baltimore, MD 21287, USA

*Corresponding author

Elizabeth W. Tucker

Email: etucker9@jh.edu

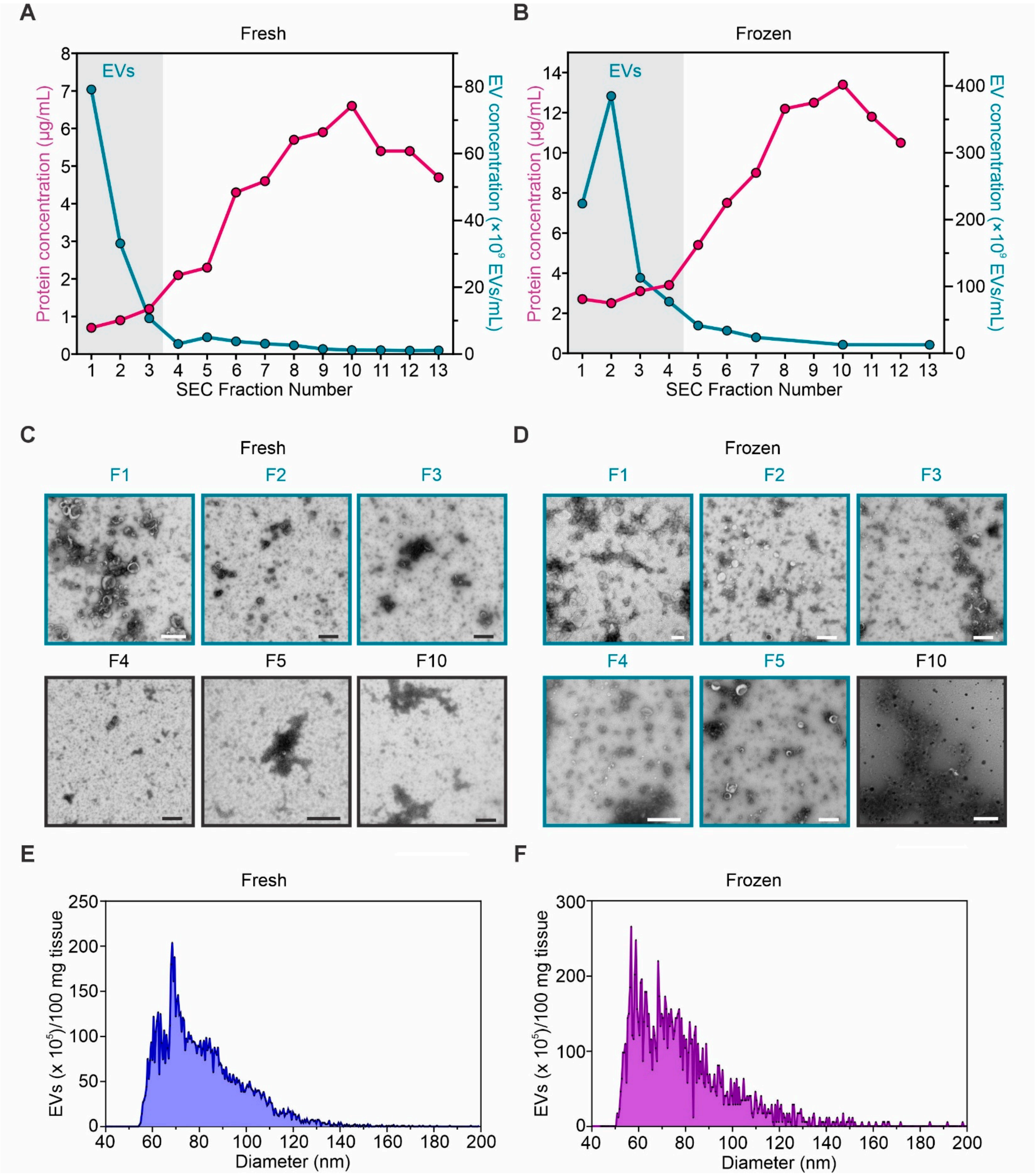

Figure S1. Brain-derived EVs from healthy adult rabbits using fresh and flash-frozen tissue processing. EVs were isolated from brain tissue from healthy adult rabbits for EV isolation protocol optimization. n = 1 animal per group. **A,C,E** Adult rabbit brain processed freshly at the time of euthanization. **B,D,F** Adult rabbit brain processed after flash freezing in -80°C. **A,B** Protein and EV concentration from first 13 size exclusion chromatography (SEC) fractions from the EV isolation from (**A**) freshly-processed and (**B**) flash-frozen adult rabbit brain. **C,D** Negative-stain TEM image visualized brain-derived EVs from adult rabbit brain with teal boxes indicating EV-rich fractions, corresponding to fraction 1-3 (F1-F3) from (**C**) freshly-processed and F1-F5 from (**D**) flash-frozen brain tissue. Scale bar = 500 nm. **E,F** Size distribution determined by Nanoflow cytometry

(NanoFCM) for (**E**) freshly-processed and (**F**) flash-frozen adult rabbit brain. EV quantification was normalized per 100 mg of brain tissue.

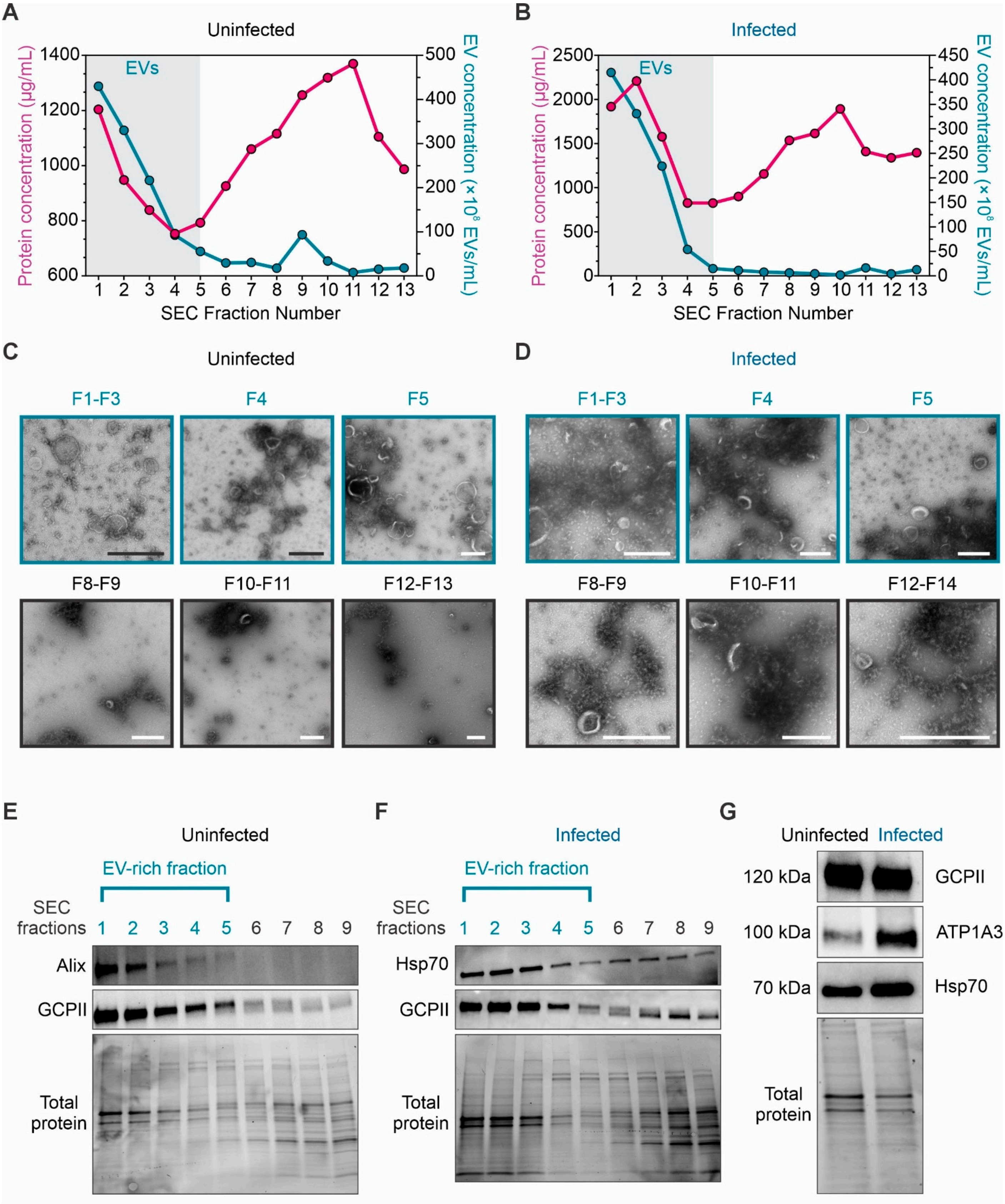

Figure S2. Characterization of brain-derived EV isolation from 1-week old rabbits. Brain-derived EVs were isolated from brain tissue from 1-week-old uninfected and infected rabbits (1-day post-injection of *M. tuberculosis*). *n* = 1 animal per group. **A,B** Protein concentration by BCA and EV concentration by NanoFCM from the first 13 size exclusion chromatography (SEC) fractions in (**A**) uninfected and (**B**) infected rabbits. EVs

are enriched in F1-F5. **C,D** Negative-stain TEM image visualized brain-derived EVs from several individual and pooled SEC fractions in (**C**) uninfected and (**D**) infected rabbits. Scale bar = 500 nm. **E,F** Western blots of common EV positive markers (Alix, Hsp70,) and the glial-associated protein, GCPII, in SEC fractions from (**E**) uninfected and (**F**) infected rabbits. Total protein loading is shown below each blot. In both groups, EV-associated markers were primarily detected in EV-rich fractions (F1–F5). **G** Pooled EV-enriched fractions (F1–F5) from each condition were analyzed by Western blot for GCPII (glial-associated protein), ATP1A3 (neuronal protein), and Hsp70 (EV-positive marker). GCPII levels appeared comparable between groups, while ATP1A3 and Hsp70 were more abundant in EVs from the *M. tuberculosis*-infected brain. Total protein staining confirms equal protein loading across conditions.

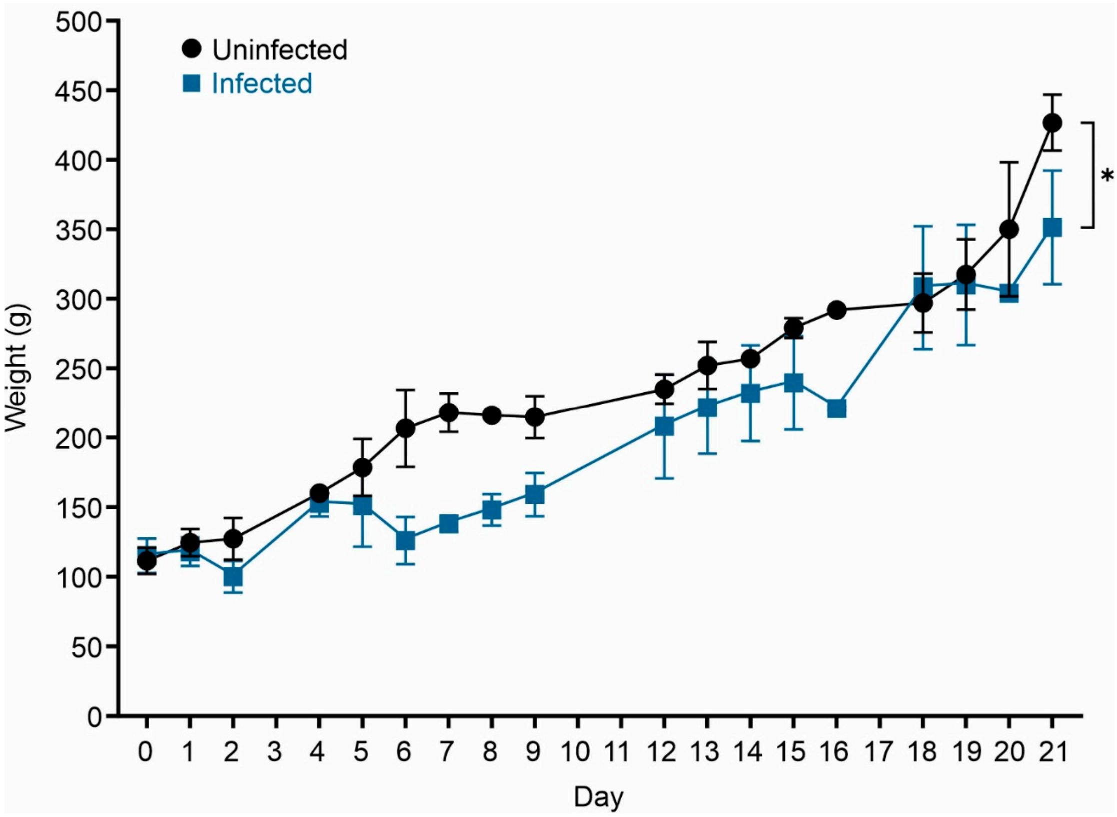

Figure S3. Body weight during infection incubation. Weight is represented in grams (g). n = 2-4 animals per group per time point. Data are presented as mean ± SD. Statistical comparisons were made using a mixed-effects model (REML). *$P < 0.05$.

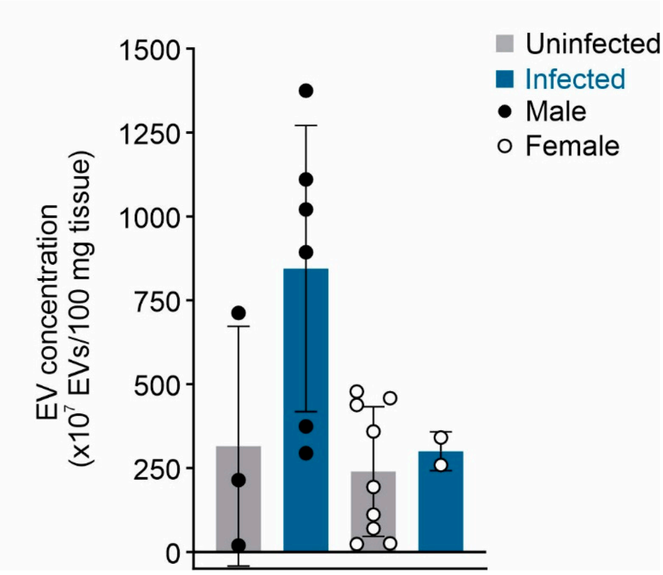

Figure S4. Brain-derived EV concentration based on sex. EVs were isolated from brain tissue from young rabbits with TB meningitis and age-matched healthy controls 3 weeks after injection, with *M. tuberculosis* (infected) or PBS (uninfected), respectively. EV concentration from **Figure 2D** is displayed based on infection group and sex (male [closed circle] and female [open circle]. *n* = 8-12 animals per group. Data are presented as mean ± SD from biologically independent samples.

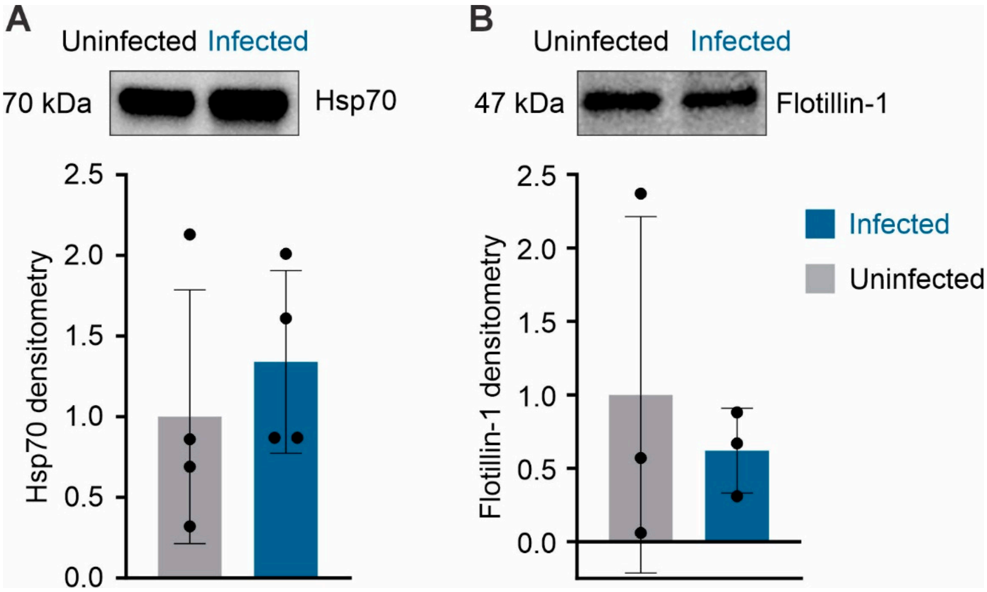

Figure S5. Expression of canonical EV markers. EVs were isolated from brain tissue from young rabbits with TB meningitis and age-matched healthy controls 3 weeks after injection, with *M. tuberculosis* (infected) or PBS (uninfected), respectively. **A,B** Western blot (top) and corresponding densitometry (bottom) of (**A**) Hsp70 and (**B**) Flotillin-1. *n* = 3-4 animals per group. Data are presented as mean ± SD from biologically independent samples. Statistical comparisons were made using an unpaired two-tailed t-test.

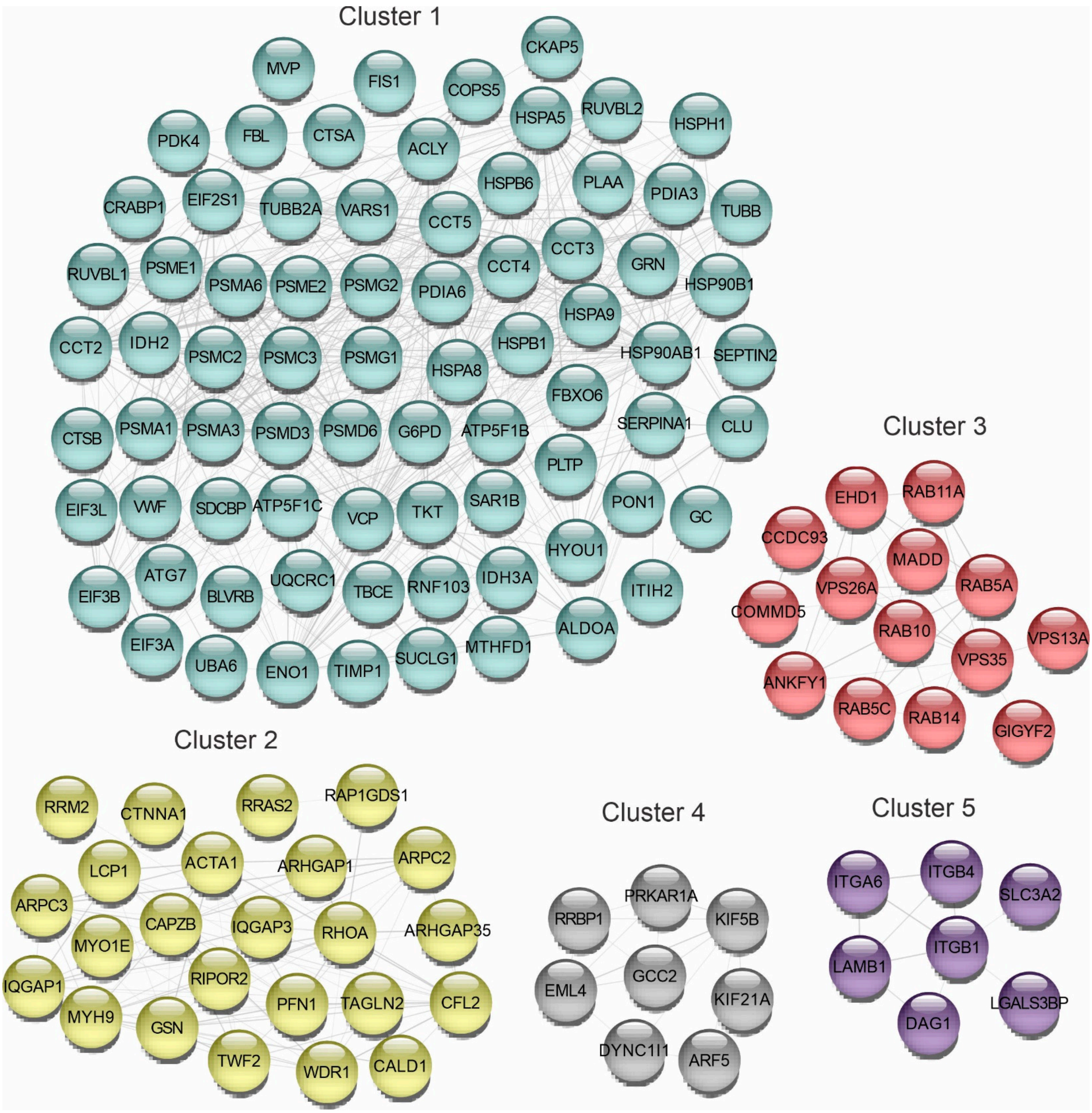

Figure S6. Enlarged STRING protein-protein interaction network clusters of upregulated proteins in brain-derived EVs during *M. tuberculosis* infection. Nodes represent individual proteins, and edges indicate predicted functional interactions based on known associations (with a minimum interaction score of 0.40). This network presents an expanded, high-resolution version of the cluster analysis shown in **Figure 4C**. All analyses were performed using proteomic data derived from *n* = 5 animals per group. Functional enrichment analyses were conducted after mapping rabbit proteins to human orthologs.

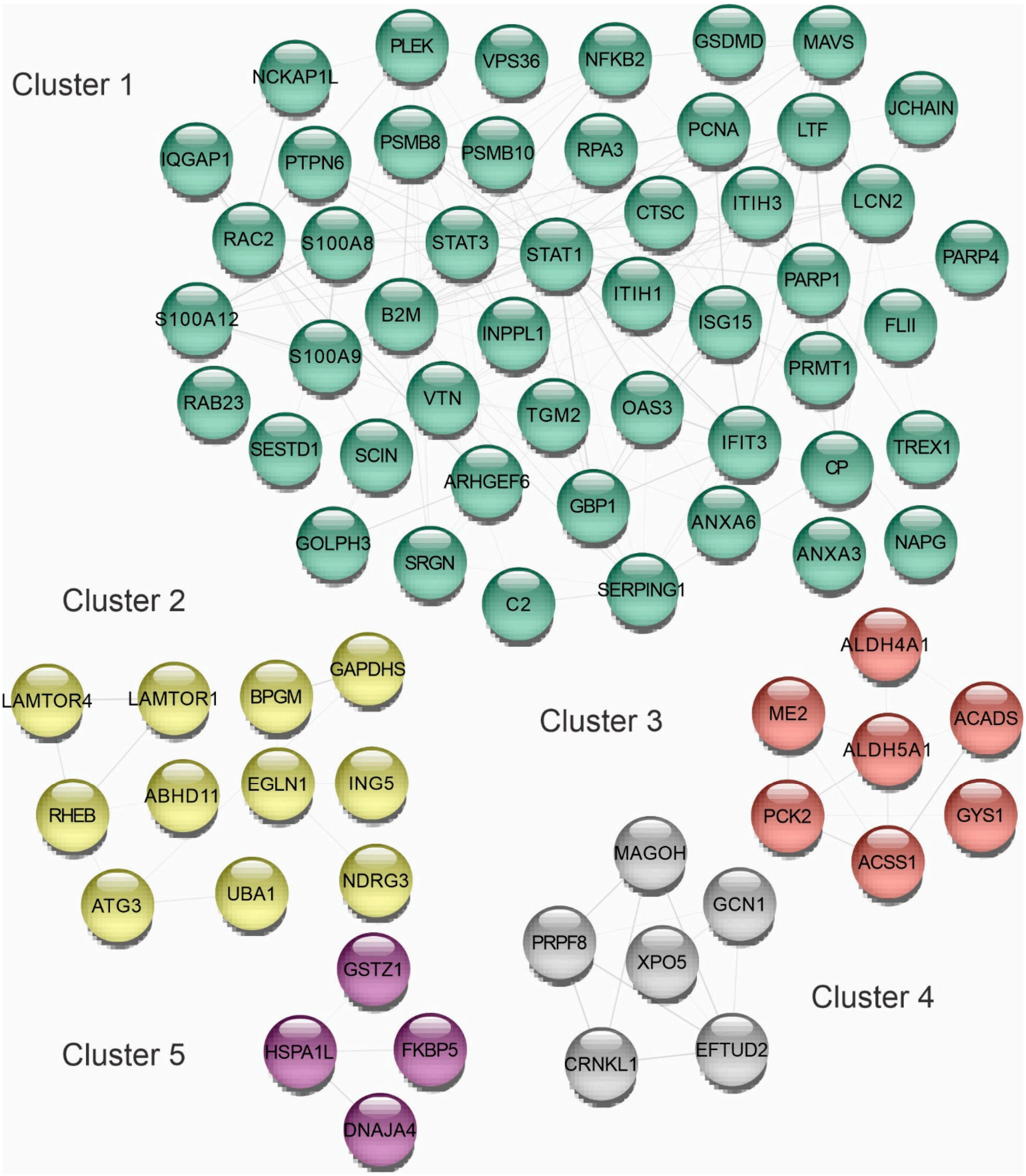

Figure S7. Enlarged STRING protein-protein interaction network clusters of infection-specific proteins. Nodes represent individual proteins, and edges indicate predicted functional interactions based on known associations (with a minimum interaction score of 0.40). This network presents an expanded, high-resolution version of the cluster analysis shown in **Figure 5C**, highlighting pathways uniquely activated during infection. All analyses were performed using proteomic data derived from *n* = 5 animals per group. Functional enrichment analyses were conducted after mapping rabbit proteins to human orthologs.

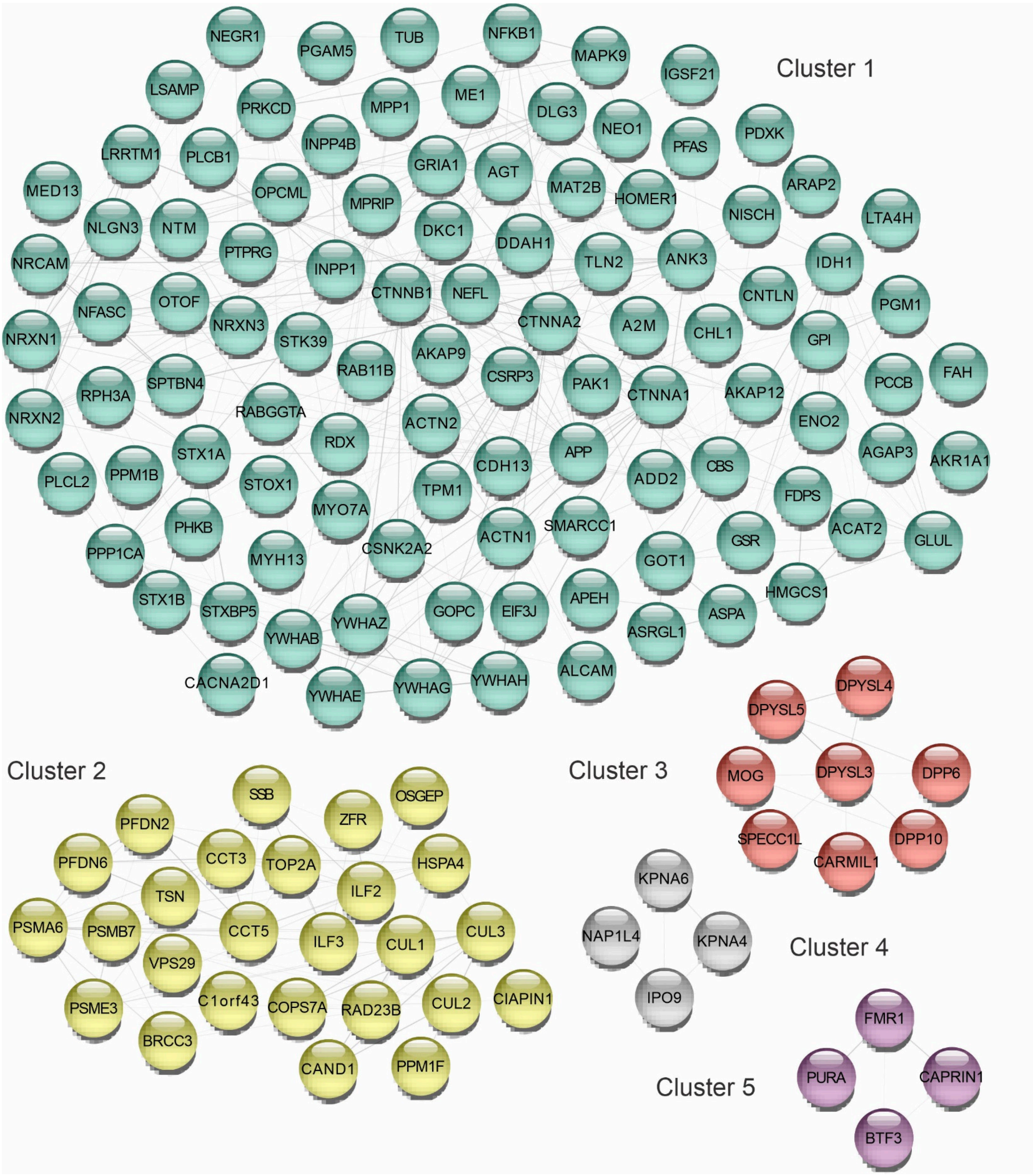

Figure S8. Enlarged STRING protein-protein interaction network clusters of downregulated proteins in brain-derived EVs during *M. tuberculosis* infection. Nodes represent individual proteins, and edges indicate predicted functional interactions based on known associations (with a minimum interaction score of 0.40). This network presents an expanded, high-resolution version of the cluster analysis shown in **Figure 6C**, illustrating pathways downregulated or depleted during infection. All analyses were performed using proteomic data derived from *n* = 5 animals per group. Functional enrichment analyses were conducted after mapping rabbit proteins to human orthologs.

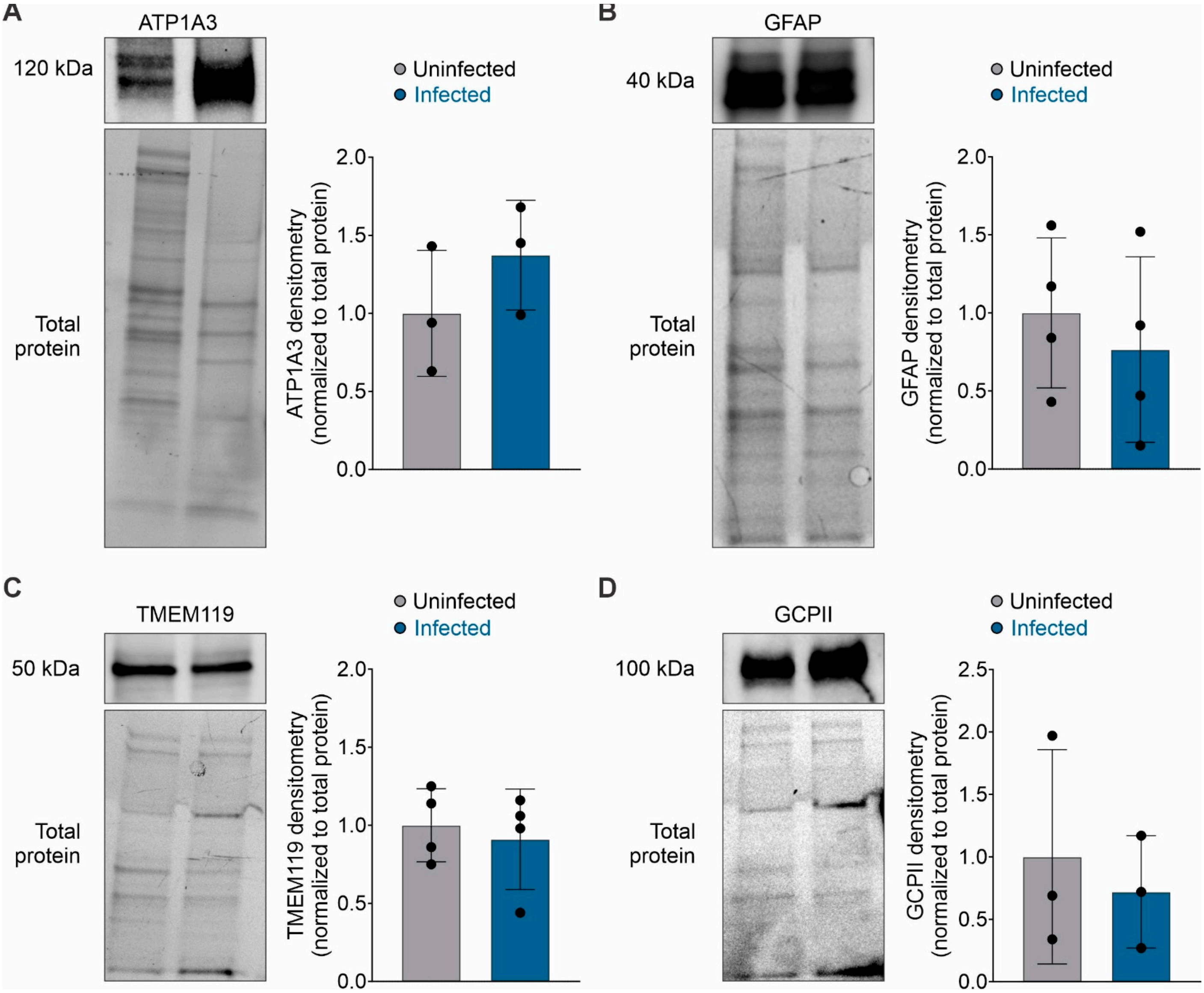

Figure S9. Expression of cell type-associated proteins in brain-derived EVs. A-D Representative Western blot (left) and corresponding densitometry (right) of (**A**) ATP1A3 (neuronal), (**B**) GFAP (astrocytic), (**C**) TMEM119 (microglial), and (**D**) GCPII (glial). All protein levels were normalized to total protein loading. *n* = 3-4 animals per group. Data are presented as mean ± SD from biologically independent samples. Statistical comparisons were made using an unpaired two-tailed t-test.
