# Supplementary material for: Tuberculous Meningitis Alters the Proteomic Landscape of Brain-Derived Extracellular Vesicles
Source: Cells. 2026 Sep 25;15(19):1749. doi: 10.3390/cells15191749 (PMC13664519; doi:10.3390/cells15191749)
Supplement: Supplementary file 1 [file cells-15-01749-s001.zip › Supplementary Table S1.pdf]

| Column1 | accession | description | genes | species | con1 | con2 | con3 | con4 | con5 | mtub1 | mtub2 | mtub3 | mtub4 | mtub5 | log2FC | t.ord | t.mod | p.ord | p.mod | q.ord | q.mod | df.r | df.0 | s2.0 | s2 | s2.post | diffexpressed | Enrichment | label |
|---|---|---|---|---|---|---|---|---|---|---|---|---|---|---|---|---|---|---|---|---|---|---|---|---|---|---|---|---|---|
| 2780 | A0A5F9C0T5 | #N/A | GSN | RABBIT | #N/A | #N/A | #N/A | #N/A | -3.71278 | #N/A | #N/A | #N/A | #N/A | 3.69939 | 7.412171 | #N/A | 7.05048 | #N/A | 0.00632 | #N/A | 0.07369 | 0 | 2.93229 | 0.55262 | #N/A | 0.552617 | Significant | Up in Mtub | GSN |
| 2835 | A0A5F9CTY3 | #N/A | RAPGEFL1 | RABBIT | #N/A | #N/A | #N/A | #N/A | -3.3577 | 3.34432 | #N/A | #N/A | #N/A | #N/A | 6.702019 | #N/A | 6.37498 | #N/A | 0.00837 | #N/A | 0.08441 | 0 | 2.93229 | 0.55262 | #N/A | 0.552617 | Significant | Up in Mtub | RAPGEFL1 |
| 2603 | G1SGW4 | #N/A | #N/A | RABBIT | #N/A | #N/A | #N/A | -6.05568 | #N/A | #N/A | 1.37904 | -0.5663 | 0 | 0.22395 | 6.314848 | 6.91082 | 7.22611 | 0.00621 | 0.00037 | 0.07337 | 0.02381 | 3 | 2.93229 | 0.55262 | 0.66797 | 0.610952 | Significant | Up in Mtub | #N/A |
| 2614 | G1TB13 | #N/A | PMVK | RABBIT | #N/A | #N/A | #N/A | -3.69901 | #N/A | 6.14057 | #N/A | 5.43625 | -1.24365 | 0 | 6.2823 | 1.49975 | 2.06952 | 0.23064 | 0.08448 | 0.2895 | 0.18737 | 3 | 2.93229 | 0.55262 | 14.0375 | 7.372019 | Not Significant | NS | #N/A |
| 1872 | G1TT67 | #N/A | #N/A | RABBIT | #N/A | -5.21419 | -5.45915 | #N/A | #N/A | 0.78601 | 0 | -1.3952 | 1.08456 | 0.58724 | 5.549194 | 7.51984 | 7.95792 | 0.00066 | 4.8E-05 | 0.0401 | 0.01186 | 5 | 2.93229 | 0.55262 | 0.77794 | 0.694645 | Significant | Up in Mtub | #N/A |
| 2488 | G1SQ01 | #N/A | AKR1A1 | RABBIT | #N/A | #N/A | #N/A | -6.45643 | #N/A | 0.35183 | -0.35183 | -6.66097 | 0.76585 | 0.51386 | 5.380174 | 1.55947 | 2.0123 | 0.1939 | 0.08448 | 0.26472 | 0.18737 | 4 | 2.93229 | 0.55262 | 9.91878 | 5.956987 | Not Significant | NS | #N/A |
| 1903 | A0A5F9CLQ3 | #N/A | PLAA | RABBIT | #N/A | #N/A | -0.02298 | #N/A | -0.01338 | 5.34116 | #N/A | #N/A | #N/A | #N/A | 5.359344 | 644.666 | 6.81659 | 0.00099 | 0.00258 | 0.04382 | 0.05283 | 1 | 2.93229 | 0.55262 | 4.6E-05 | 0.412095 | Significant | Up in Mtub | PLAA |
| 1310 | A0A5F9C411 | Immunoglobulin heavy constant mu OS=Oryctolagu | IGHM | RABBIT | #N/A | #N/A | #N/A | -5.42344 | -3.44584 | 0 | 2.26772 | 1.84387 | -0.59147 | 0.54097 | 5.246862 | 5.00756 | 5.74185 | 0.00408 | 0.00045 | 0.06755 | 0.02567 | 5 | 2.93229 | 0.55262 | 1.56837 | 1.192882 | Significant | Up in Mtub | IGHM |
| 1814 | G1SS91 | #N/A | #N/A | RABBIT | #N/A | -3.16607 | #N/A | -5.66703 | #N/A | #N/A | 1.03742 | 0.38359 | -0.13569 | 0.13569 | 4.771803 | 5.59255 | 6.18417 | 0.00502 | 0.00047 | 0.07293 | 0.02567 | 4 | 2.93229 | 0.55262 | 0.9707 | 0.793855 | Significant | Up in Mtub | #N/A |
| 2656 | A0A5F9CXP3 | #N/A | LMO7 | RABBIT | #N/A | #N/A | #N/A | #N/A | -2.3549 | #N/A | #N/A | #N/A | #N/A | 2.34151 | 4.696407 | #N/A | 4.46723 | #N/A | 0.02193 | #N/A | 0.11057 | 0 | 2.93229 | 0.55262 | #N/A | 0.552617 | Significant | Up in Mtub | LMO7 |
| 740 | G1SL68 | Myosin-9 OS=Oryctolagus cuniculus OX=9986 GN= | MYH9 | RABBIT | -5.6174 | -5.57948 | -0.10769 | -5.0872 | -0.29858 | 0.27834 | 2.93347 | 1.18262 | 1.50057 | 0.75215 | 4.667498 | 3.43094 | 3.92572 | 0.00894 | 0.0024 | 0.09045 | 0.05138 | 8 | 2.93229 | 0.55262 | 4.62681 | 3.534017 | Significant | Up in Mtub | MYH9 |
| 1727 | G1TET2 | #N/A | LCP1 | RABBIT | #N/A | -2.79984 | #N/A | -5.15785 | #N/A | -1.42976 | 2.72135 | -0.06437 | 1.07571 | 0.56528 | 4.552486 | 3.50277 | 4.14248 | 0.01723 | 0.0033 | 0.10978 | 0.05702 | 5 | 2.93229 | 0.55262 | 2.41311 | 1.725351 | Significant | Up in Mtub | LCP1 |
| 2675 | G1SSD7 | #N/A | #N/A | RABBIT | #N/A | #N/A | #N/A | -2.13573 | #N/A | 2.07809 | #N/A | #N/A | #N/A | #N/A | 4.213828 | #N/A | 4.0082 | #N/A | 0.02907 | #N/A | 0.12009 | 0 | 2.93229 | 0.55262 | #N/A | 0.552617 | Significant | Up in Mtub | #N/A |
| 112 | A0A5F9D4G4 | Rap1 GTPase-GDP dissociation stimulator 1 OS=Or | RAP1GDS1 | RABBIT | #N/A | -0.38792 | -1.52957 | -0.05764 | -0.78101 | -1.80919 | 4.60066 | 4.59721 | 5.21712 | 4.5505 | 4.120291 | 2.71465 | 3.1629 | 0.03 | 0.01019 | 0.12757 | 0.09149 | 7 | 2.93229 | 0.55262 | 5.11937 | 3.771137 | Significant | Up in Mtub | RAP1GDS1 |
| 2698 | U3KM44 | #N/A | #N/A | RABBIT | #N/A | #N/A | #N/A | -3.9819 | #N/A | #N/A | 0.17181 | #N/A | 0 | #N/A | 4.067807 | 27.3387 | 5.15054 | 0.02328 | 0.00706 | 0.11776 | 0.07947 | 1 | 2.93229 | 0.55262 | 0.01476 | 0.415837 | Significant | Up in Mtub | #N/A |
| 2649 | G1SK20 | #N/A | #N/A | RABBIT | #N/A | #N/A | #N/A | -3.0191 | #N/A | #N/A | 2.0625 | #N/A | 0 | #N/A | 4.050355 | 2.26761 | 3.3877 | 0.26441 | 0.02833 | 0.30603 | 0.11898 | 1 | 2.93229 | 0.55262 | 2.12696 | 0.95298 | Significant | Up in Mtub | #N/A |
| 2386 | G1SSY9 | #N/A | RNF17 | RABBIT | #N/A | #N/A | -1.93577 | #N/A | #N/A | #N/A | 2.10642 | #N/A | #N/A | #N/A | 4.042198 | #N/A | 3.84495 | #N/A | 0.03232 | #N/A | 0.12495 | 0 | 2.93229 | 0.55262 | #N/A | 0.552617 | Significant | Up in Mtub | RNF17 |
| 1604 | G1TAB8 | Myosin IE OS=Oryctolagus cuniculus OX=9986 GN= | MYO1E | RABBIT | -2.02617 | #N/A | #N/A | #N/A | #N/A | #N/A | #N/A | 1.97347 | #N/A | #N/A | 3.999643 | #N/A | 3.80447 | #N/A | 0.0332 | #N/A | 0.12595 | 0 | 2.93229 | 0.55262 | #N/A | 0.552617 | Significant | Up in Mtub | MYO1E |
| 2179 | U3KMZ9 | #N/A | #N/A | RABBIT | #N/A | #N/A | -3.1625 | #N/A | #N/A | 2.12284 | #N/A | -0.06437 | 1.48123 | -0.35486 | 3.958713 | 2.95931 | 3.5459 | 0.05958 | 0.01236 | 0.15524 | 0.09506 | 3 | 2.93229 | 0.55262 | 1.43158 | 0.997116 | Significant | Up in Mtub | #N/A |
| 2337 | B7NZB1 | #N/A | FGF1 FGF2 | RABBIT | #N/A | #N/A | #N/A | #N/A | -3.70319 | 1.24259 | -0.29623 | -0.66438 | 0.29623 | 0.52716 | 3.924261 | 4.83652 | 4.82908 | 0.00842 | 0.00195 | 0.08795 | 0.04926 | 4 | 2.93229 | 0.55262 | 0.54862 | 0.550308 | Significant | Up in Mtub | FGF1 FGF2B |
| 1873 | G1SJM9 | #N/A | IQGAP3 | RABBIT | #N/A | -1.84081 | #N/A | #N/A | #N/A | #N/A | #N/A | #N/A | #N/A | 2.03863 | 3.879447 | #N/A | 3.69014 | #N/A | 0.03586 | #N/A | 0.12868 | 0 | 2.93229 | 0.55262 | #N/A | 0.552617 | Significant | Up in Mtub | IQGAP3 |
| 1417 | G1TK26 | Surfactant protein D OS=Oryctolagus cuniculus OX | SFTPD | RABBIT | -2.90523 | -1.84891 | -0.72176 | #N/A | -1.11314 | 0.89241 | 2.52339 | 2.99329 | #N/A | 2.36132 | 3.839858 | 5.81391 | 6.19884 | 0.00114 | 0.00016 | 0.04885 | 0.0188 | 6 | 2.93229 | 0.55262 | 0.87241 | 0.767432 | Significant | Up in Mtub | SFTPD |
| 322 | P62143 | Serine/threonine-protein phosphatase PP1-beta cat | PPP1CB | RABBIT | -4.69559 | #N/A | -2.53853 | #N/A | -3.65218 | 0.72163 | 0.01811 | -0.08248 | 0.15552 | 0.0778 | 3.806883 | 7.73255 | 7.47218 | 0.00025 | 4E-05 | 0.03578 | 0.01186 | 6 | 2.93229 | 0.55262 | 0.45446 | 0.486683 | Significant | Up in Mtub | PPP1CB |
| 1471 | G1TD19 | Protein-serine/threonine kinase OS=Oryctolagus cu | PDK4 | RABBIT | -1.83015 | #N/A | #N/A | #N/A | #N/A | 1.84181 | #N/A | #N/A | #N/A | #N/A | 3.671954 | #N/A | 3.49277 | #N/A | 0.04113 | #N/A | 0.13433 | 0 | 2.93229 | 0.55262 | #N/A | 0.552617 | Significant | Up in Mtub | PDK4 |
| 1859 | A0A5F9CJ45 | #N/A | PLTP | RABBIT | #N/A | -1.71414 | #N/A | #N/A | #N/A | 1.91196 | #N/A | #N/A | #N/A | #N/A | 3.626091 | #N/A | 3.44915 | #N/A | 0.04243 | #N/A | 0.13674 | 0 | 2.93229 | 0.55262 | #N/A | 0.552617 | Significant | Up in Mtub | PLTP |
| 2560 | G1T7I8 | #N/A | RIC8A | RABBIT | #N/A | #N/A | #N/A | -3.38126 | #N/A | 0.68864 | #N/A | -0.78574 | 0 | 0.58687 | 3.503702 | 4.62713 | 4.40936 | 0.01901 | 0.00465 | 0.11259 | 0.06565 | 3 | 2.93229 | 0.55262 | 0.45869 | 0.505118 | Significant | Up in Mtub | RIC8A |
| 839 | P62493 | Ras-related protein Rab-11A OS=Oryctolagus cunic | RAB11A RA | RABBIT | -2.46816 | -1.51536 | -4.15536 | -4.23045 | -0.6387 | 0.90818 | 0.8105 | 0.56095 | 0.93606 | 1.13591 | 3.471924 | 4.83951 | 5.25855 | 0.00129 | 0.00027 | 0.04885 | 0.02295 | 8 | 2.93229 | 0.55262 | 1.2867 | 1.089804 | Significant | Up in Mtub | RAB11A RAB11 |
| 709 | A0A5F9CSP5 | IQ motif containing GTPase activating protein 1 OS= | IQGAP1 | RABBIT | -0.89965 | 0.19782 | #N/A | 0.69898 | -0.06169 | #N/A | #N/A | #N/A | 3.42104 | #N/A | 3.437169 | 4.60024 | 4.35203 | 0.01931 | 0.00494 | 0.11273 | 0.06702 | 3 | 2.93229 | 0.55262 | 0.44661 | 0.499009 | Significant | Up in Mtub | IQGAP1 |
| 1879 | G1STS3 | #N/A | MMS19 | RABBIT | #N/A | #N/A | -2.82075 | -0.8614 | -3.78516 | 1.0525 | -0.68487 | 1.59554 | 1.94293 | 0.68487 | 3.407297 | 3.89925 | 4.36378 | 0.00799 | 0.00185 | 0.08529 | 0.04725 | 6 | 2.93229 | 0.55262 | 1.43172 | 1.14313 | Significant | Up in Mtub | MMS19 |
| 2562 | G1TFU9 | #N/A | LGALS3BP | RABBIT | #N/A | #N/A | #N/A | -3.307 | -2.06788 | -0.47806 | 2.42371 | 1.28891 | 0 | 0.33079 | 3.400512 | 3.68003 | 4.12005 | 0.01429 | 0.00341 | 0.09894 | 0.05724 | 5 | 2.93229 | 0.55262 | 1.2198 | 0.973165 | Significant | Up in Mtub | LGALS3BP |
| 2119 | Q9BGH4 | #N/A | #N/A | RABBIT | #N/A | #N/A | #N/A | -3.35274 | #N/A | -0.69817 | 0.23319 | 0.02089 | 0.72339 | -0.08526 | 3.391554 | 5.99937 | 4.97412 | 0.00388 | 0.00166 | 0.06755 | 0.04595 | 4 | 2.93229 | 0.55262 | 0.26632 | 0.387421 | Significant | Up in Mtub | #N/A |
| 1935 | G1SE49 | #N/A | VPS51 | RABBIT | #N/A | #N/A | -1.68908 | -3.16299 | #N/A | 1.83561 | 1.78222 | -0.06437 | -0.61219 | 1.65091 | 3.344473 | 3.5009 | 3.94626 | 0.01727 | 0.00433 | 0.10978 | 0.06392 | 5 | 2.93229 | 0.55262 | 1.30376 | 1.02609 | Significant | Up in Mtub | VPS51 |
| 1126 | P08855 | Calpastatin OS=Oryctolagus cuniculus OX=9986 G | CAST | RABBIT | #N/A | #N/A | #N/A | -3.0454 | #N/A | #N/A | 0.58817 | #N/A | 0 | #N/A | 3.339484 | 6.55615 | 4.03755 | 0.09636 | 0.01618 | 0.1866 | 0.10049 | 1 | 2.93229 | 0.55262 | 0.17297 | 0.456071 | Significant | Up in Mtub | CAST |
| 1012 | U3KPI0 | 40S ribosomal protein S15a OS=Oryctolagus cunicu | #N/A | RABBIT | -0.02992 | 0.19782 | #N/A | #N/A | #N/A | #N/A | 3.40358 | #N/A | #N/A | #N/A | 3.319629 | 16.8312 | 4.18893 | 0.03778 | 0.01432 | 0.13845 | 0.09913 | 1 | 2.93229 | 0.55262 | 0.02593 | 0.418679 | Significant | Up in Mtub | #N/A |
| 1958 | A0A5F9DV86 | #N/A | WDR7 | RABBIT | #N/A | #N/A | #N/A | -0.05764 | -4.80559 | 2.51697 | -1.39538 | #N/A | #N/A | 1.52867 | 3.315034 | 1.42257 | 1.92235 | 0.25001 | 0.10348 | 0.2994 | 0.20254 | 3 | 2.93229 | 0.55262 | 6.51642 | 3.568556 | Not Significant | NS | #N/A |
| 1546 | A0A5F9D948 | phenylalanine--tRNA ligase OS=Oryctolagus cunicu | FARSB | RABBIT | -2.39928 | 0.19782 | #N/A | #N/A | #N/A | #N/A | 2.20037 | #N/A | #N/A | #N/A | 3.301104 | 1.46771 | 2.39199 | 0.38075 | 0.07611 | 0.36082 | 0.1805 | 1 | 2.93229 | 0.55262 | 3.37246 | 1.269717 | Not Significant | NS | #N/A |
| 1323 | A0A5F9C4V2 | Cathepsin B OS=Oryctolagus cuniculus OX=9986 G | CTSB | RABBIT | -3.11215 | -2.04044 | -1.35302 | -0.75726 | -1.13733 | 2.53051 | 1.69422 | 0.63526 | 1.33168 | 1.90506 | 3.299386 | 6.34822 | 6.50879 | 0.00022 | 4.5E-05 | 0.03578 | 0.01186 | 8 | 2.93229 | 0.55262 | 0.67531 | 0.6424 | Significant | Up in Mtub | CTSB |
| 873 | G1TD91 | Cleavage and polyadenylation specificity factor sub | NUDT21 | RABBIT | -0.43004 | -0.0967 | -1.08748 | 0.60387 | 0.09487 | 5.90133 | 5.63429 | 5.45364 | -1.37889 | -0.10826 | 3.28352 | 2.04134 | 2.3498 | 0.07552 | 0.03863 | 0.16901 | 0.13043 | 8 | 2.93229 | 0.55262 | 6.46825 | 4.881542 | Significant | Up in Mtub | NUDT21 |
| 1805 | P53789 | #N/A | GC DBP | RABBIT | #N/A | -0.62692 | #N/A | -0.58862 | -1.62213 | 0.53098 | 3.78055 | 2.65698 | #N/A | #N/A | 3.268729 | 3.23283 | 3.78519 | 0.03189 | 0.00697 | 0.13025 | 0.07947 | 4 | 2.93229 | 0.55262 | 1.5335 | 1.118596 | Significant | Up in Mtub | GC DBP |
| 1161 | F5XVB8 | von Willebrand factor OS=Oryctolagus cuniculus O | VWF | RABBIT | #N/A | #N/A | #N/A | -1.10687 | -0.01338 | #N/A | 2.70356 | #N/A | #N/A | #N/A | 3.263682 | 3.44639 | 3.54794 | 0.17978 | 0.02453 | 0.25448 | 0.11483 | 1 | 2.93229 | 0.55262 | 0.59785 | 0.564121 | Significant | Up in Mtub | VWF |
| 1887 | G1TB18 | #N/A | ARHGAP1 | RABBIT | #N/A | -2.58346 | -0.98615 | -0.7624 | #N/A | 0.67866 | -0.67866 | 2.67303 | 5.14064 | 1.12539 | 3.231815 | 2.3243 | 2.73586 | 0.0591 | 0.02316 | 0.15506 | 0.11217 | 6 | 2.93229 | 0.55262 | 3.62503 | 2.616417 | Significant | Up in Mtub | ARHGAP1 |
| 1060 | G1SW41 | Drebrin like OS=Oryctolagus cuniculus OX=9986 PE | #N/A | RABBIT | 0.01166 | #N/A | #N/A | #N/A | -0.2323 | #N/A | #N/A | #N/A | 3.11819 | #N/A | 3.228512 | 15.2806 | 4.06923 | 0.0416 | 0.01577 | 0.14427 | 0.09934 | 1 | 2.93229 | 0.55262 | 0.02976 | 0.419652 | Significant | Up in Mtub | #N/A |
| 2885 | G1TF51 | #N/A | ZEB2 | RABBIT | #N/A | #N/A | #N/A | #N/A | -1.61269 | #N/A | #N/A | #N/A | #N/A | 1.59931 | 3.211998 | #N/A | 3.05526 | #N/A | 0.05687 | #N/A | 0.15828 | 0 | 2.93229 | 0.55262 | #N/A | 0.552617 | Not Significant | NS | #N/A |
| 2573 | A0A5F9CFJ4 | #N/A | PSMG2 | RABBIT | #N/A | #N/A | #N/A | -3.51325 | #N/A | -2.77429 | 0.90653 | 0.05476 | 0.39453 | -0.11913 | 3.205734 | 2.0416 | 2.45641 | 0.11074 | 0.04402 | 0.1981 | 0.14088 | 4 | 2.93229 | 0.55262 | 2.05462 | 1.41929 | Significant | Up in Mtub | PSMG2 |
| 1406 | B7NZR0 | Glucose-6-phosphate 1-dehydrogenase OS=Oryctc | G6PD | RABBIT | -2.65047 | -2.9061 | -0.52907 | -0.41692 | -4.50681 | 1.83077 | 0.6677 | 0.29491 | 1.3363 | 0.86229 | 3.200268 | 3.90625 | 4.31344 | 0.0045 | 0.00125 | 0.06905 | 0.04432 | 8 | 2.93229 | 0.55262 | 1.678 | 1.376149 | Significant | Up in Mtub | G6PD |
| 1539 | G1T7N4 | Protein kinase C OS=Oryctolagus cuniculus OX=99 | PRKCI | RABBIT | -2.7584 | #N/A | #N/A | -1.54436 | #N/A | 1.55608 | #N/A | -0.89374 | 0.82937 | 2.64751 | 3.186187 | 2.71024 | 3.23046 | 0.05352 | 0.01464 | 0.15354 | 0.09934 | 4 | 2.93229 | 0.55262 | 1.84274 | 1.297034 | Significant | Up in Mtub | PRKCI |
| 15 | A0A5F9DHA9 | 14-3-3 protein epsilon OS=Oryctolagus cuniculus C | YWHAE | RABBIT | -1.91752 | 0.19782 | #N/A | #N/A | #N/A | #N/A | 0.3994 | #N/A | -0.92307 | 7.49674 | 3.184202 | 0.91875 | 1.26841 | 0.42598 | 0.25215 | 0.38072 | 0.3189 | 3 | 2.93229 | 0.55262 | 14.414 | 7.562435 | Not Significant | NS | #N/A |
| 2712 | G1SL62 | #N/A | ICE2 | RABBIT | #N/A | #N/A | #N/A | -1.82754 | #N/A | #N/A | #N/A | -0.06437 | #N/A | 2.74468 | 3.167693 | 1.30213 | 2.17398 | 0.41693 | 0.09656 | 0.37794 | 0.19688 | 1 | 2.93229 | 0.55262 | 3.94536 | 1.415409 | Not Significant | NS | #N/A |
| 2541 | A0A5F9CP00 | #N/A | SRPK1 | RABBIT | #N/A | #N/A | #N/A | -3.15029 | #N/A | 0.01544 | #N/A | -0.06437 | #N/A | #N/A | 3.125831 | 45.228 | 3.97192 | 0.01407 | 0.01707 | 0.09799 | 0.10198 | 1 | 2.93229 | 0.55262 | 0.00318 | 0.412893 | Significant | Up in Mtub | SRPK1 |
| 2822 | A0A5F9DGV6 | #N/A | TOPBP1 | RABBIT | #N/A | #N/A | #N/A | #N/A | -1.59723 | #N/A | #N/A | 1.51948 | #N/A | #N/A | 3.116701 | #N/A | 2.96461 | #N/A | 0.06104 | #N/A | 0.16251 | 0 | 2.93229 | 0.55262 | #N/A | 0.552617 | Not Significant | NS | #N/A |
| 991 | G1SCT5 | Coronin OS=Oryctolagus cuniculus OX=9986 GN=C | CORO7 | RABBIT | -1.94289 | -1.8999 | -3.55488 | 0.1986 | -1.14391 | 2.47901 | 1.48225 | -0.32061 | 2.58699 | 0.90371 | 3.094865 | 3.80493 | 4.19827 | 0.0052 | 0.00151 | 0.07293 | 0.04508 | 8 | 2.93229 | 0.55262 | 1.65398 | 1.35857 | Significant | Up in Mtub | CORO7 |
| 1293 | G1TER3 | B-cell receptor-associated protein OS=Oryctolagus | BCAP31 | RABBIT | #N/A | -2.67982 | #N/A | -3.37505 | -0.97146 | 0.73785 | 1.91971 | 0.40875 | -0.47311 | 1.15233 | 3.091216 | 4.16318 | 4.52303 | 0.00592 | 0.00147 | 0.07337 | 0.04508 | 6 | 2.93229 | 0.55262 | 1.03373 | 0.875793 | Significant | Up in Mtub | BCAP31 |
| 307 | G1SEF9 | Desmin OS=Oryctolagus cuniculus OX=9986 GN=D | DES | RABBIT | #N/A | -3.80728 | -0.08231 | -1.64388 | -2.37279 | #N/A | 2.87181 | 0.32278 | 0.25296 | 0.93427 | 3.072018 | 3.11173 | 3.55821 | 0.0208 | 0.00621 | 0.11673 | 0.07298 | 6 | 2.93229 | 0.55262 | 1.94928 | 1.490784 | Significant | Up in Mtub | DES |
| 2200 | A0A5F9D815 | #N/A | ITIH2 | RABBIT | #N/A | #N/A | -1.56584 | -1.03091 | #N/A | 0.97327 | 2.47429 | #N/A | #N/A | #N/A | 3.022158 | 3.79315 | 3.94812 | 0.06301 | 0.01117 | 0.15802 | 0.09296 | 2 | 2.93229 | 0.55262 | 0.6348 | 0.58594 | Significant | Up in Mtub | ITIH2 |
| 470 | A0A5F9D376 | Exportin-2 OS=Oryctolagus cuniculus OX=9986 GN | CSE1L | RABBIT | -3.50272 | -3.85245 | -3.25695 | -1.47418 | -0.08932 | 0.58547 | 1.74396 | 0.36936 | 0.09138 | 0.07593 | 3.008345 | 3.86098 | 4.23941 | 0.0048 | 0.00141 | 0.07239 | 0.04508 | 8 | 2.93229 | 0.55262 | 1.51775 | 1.258878 | Significant | Up in Mtub | CSE1L |
| 1376 | G1SUS6 | Small COPII coat GTPase SAR1B OS=Oryctolagus c | SAR1B | RABBIT | -1.99182 | -1.7453 | -0.87876 | -0.05764 | -1.24136 | 2.07215 | #N/A | 0.89872 | 2.12086 | 2.14693 | 2.992641 | 6.3619 | 6.2487 | 0.00038 | 9.8E-05 | 0.03578 | 0.01529 | 7 | 2.93229 | 0.55262 | 0.49173 | 0.509702 | Significant | Up in Mtub | SAR1B |
| 1976 | G1SG15 | #N/A | RPGRIP1L | RABBIT | #N/A | 0.10595 | -4.03573 | #N/A | 0.07848 | #N/A | #N/A | #N/A | #N/A | 1.70235 | 2.986112 | 1.08507 | 1.59408 | 0.39127 | 0.1726 | 0.36581 | 0.26481 | 2 | 2.93229 | 0.55262 | 5.68016 | 2.631793 | Not Significant | NS | #N/A |
| 2067 | B7NZA6 | #N/A | PSMG1 | RABBIT | #N/A | #N/A | #N/A | -2.87046 | #N/A | -0.1336 | -0.38967 | 0.07156 | 0.1336 | 0.86032 | 2.9789 | 5.81751 | 4.53332 | 0.00435 | 0.00276 | 0.0684 | 0.05341 | 4 | 2.93229 | 0.55262 | 0.2185 | 0.35983 | Significant | Up in Mtub | PSMG1 |
| 660 | G1SJ23 | Calpain-1 catalytic subunit OS=Oryctolagus cunicu | CAPN1 | RABBIT | -2.2954 | -0.916 | -4.68602 | 1.05618 | -3.15504 | 1.50133 | -1.28565 | 1.40176 | 1.82638 | 1.43541 | 2.975099 | 2.62668 | 2.97796 | 0.03033 | 0.01264 | 0.12782 | 0.09521 | 8 | 2.93229 | 0.55262 | 3.20723 | 2.495202 | Significant | Up in Mtub | CAPN1 |
| 1343 | A0A5F9D713 | Protein NDRG2 OS=Oryctolagus cuniculus OX=998 | NDRG2 | RABBIT | -4.4058 | #N/A | -0.78307 | #N/A | #N/A | -0.11163 | #N/A | 1.02171 | 0.11163 | 0.4225 | 2.955486 | 2.52828 | 3.01048 | 0.06478 | 0.01988 | 0.1592 | 0.10889 | 4 | 2.93229 | 0.55262 | 1.822 | 1.285062 | Significant | Up in Mtub | NDRG2 |
| 82 | G8ZF10 | Alpha-globin 1 OS=Oryctolagus cuniculus OX=998 | HBA1 | RABBIT | -3.82943 | #N/A | -10.6757 | 1.3583 | 1.8139 | #N/A | #N/A | -1.48031 | 1.65513 | #N/A | 2.920657 | 0.65335 | 0.85365 | 0.54918 | 0.42181 | 0.43219 | 0.4086 | 4 | 2.93229 | 0.55262 | 26.6444 | 15.60785 | Not Significant | NS | #N/A |
| 1179 | A0A5F9CT68 | Cytoskeleton associated protein 5 OS=Oryctolagus | CKAP5 | RABBIT | -3.06177 | -2.65648 | -1.17324 | 0.31281 | -3.37637 | 1.26997 | -0.37045 | 1.59093 | 1.0774 | 1.01225 | 2.90703 | 3.7917 | 4.15532 | 0.0053 | 0.00162 | 0.07293 | 0.04595 | 8 | 2.93229 | 0.55262 | 1.4695 | 1.223573 | Significant | Up in Mtub | CKAP5 |
| 1240 | A0A5F9DNN3 | Basic leucine zipper and W2 domains 1 OS=Oryctol | #N/A | RABBIT | -5.17191 | -4.43809 | -1.85739 | 0.51908 | -1.49681 | 0.83995 | -0.24841 | 0.18405 | 0.34365 | 0.96845 | 2.906563 | 2.74557 | 3.09946 | 0.02523 | 0.01018 | 0.12061 | 0.09149 | 8 | 2.93229 | 0.55262 | 2.80179 | 2.19851 | Significant | Up in Mtub | #N/A |
| 2230 | G1TIS5 | #N/A | ANXA1 | RABBIT | #N/A | #N/A | -1.44948 | #N/A | #N/A | 0 | 2.90424 | #N/A | #N/A | #N/A | 2.901597 | 1.15365 | 1.94443 | 0.45466 | 0.12497 | 0.39384 | 0.22412 | 1 | 2.93229 | 0.55262 | 4.21731 | 1.484566 | Not Significant | NS | #N/A |
| 1153 | A0A5F9DSP2 | Coatomer subunit beta OS=Oryctolagus cuniculus | COPB1 | RABBIT | -3.1203 | -1.51374 | #N/A | #N/A | -0.01338 | #N/A | 1.69307 | 0.97617 | #N/A | #N/A | 2.883757 | 2.42633 | 2.97123 | 0.09364 | 0.02526 | 0.18402 | 0.11552 | 3 | 2.93229 | 0.55262 | 1.69511 | 1.130383 | Significant | Up in Mtub | COPB1 |
| 630 | G1TMN0 | Histone H2A OS=Oryctolagus cuniculus OX=9986 ( | H2AC7 | RABBIT | 0.09125 | 0.71935 | 1.15638 | -7.55616 | -8.5426 | -0.25836 | 2.46341 | 0.88891 | -0.07959 | -2.74417 | 2.880396 | 1.24857 | 1.44858 | 0.24712 | 0.17552 | 0.29795 | 0.26731 | 8 | 2.93229 | 0.55262 | 13.3051 | 9.884585 | Not Significant | NS | #N/A |
| 1694 | P29562 | #N/A | EIF4A1 DD | RABBIT | #N/A | -3.68064 | -1.3104 | -0.4247 | -1.57426 | 2.9439 | 0.44368 | -0.06437 | 1.0119 | 1.32453 | 2.87943 | 3.43236 | 3.81588 | 0.01095 | 0.00344 | 0.09732 | 0.05724 | 7 | 2.93229 | 0.55262 | 1.56392 | 1.265353 | Significant | Up in Mtub | EIF4A1 DDX2A EIF4A |
| 587 | A0A5F9C2B4 | Dynamin-1-like protein OS=Oryctolagus cuniculus ( | DNM1L | RABBIT | -4.77411 | -6.50627 | -0.44913 | 0.30938 | -1.29835 | 0.61504 | -0.14894 | 0.08457 | 0.62335 | 0.41632 | 2.861765 | 2.15657 | 2.46494 | 0.06313 | 0.03152 | 0.15805 | 0.12426 | 8 | 2.93229 | 0.55262 | 4.40231 | 3.369737 | Significant | Up in Mtub | DNM1L |
| 2018 | G1TCW2 | #N/A | #N/A | RABBIT | #N/A | -2.85075 | #N/A | #N/A | -2.89044 | 0.72462 | 0.36229 | -1.57286 | 0 | 0.3451 | 2.84242 | 4.2164 | 4.33738 | 0.00836 | 0.00254 | 0.08791 | 0.05283 | 5 | 2.93229 | 0.55262 | 0.64922 | 0.613512 | Significant | Up in Mtub | #N/A |
| 2704 | A0A5F9CI34 | #N/A | SENP5 | RABBIT | #N/A | #N/A | #N/A | -1.42668 | #N/A | #N/A | 1.36904 | #N/A | #N/A | #N/A | 2.795714 | #N/A | 2.65929 | #N/A | 0.07826 | #N/A | 0.18205 | 0 | 2.93229 | 0.55262 | #N/A | 0.552617 | Not Significant | NS | #N/A |
| 2009 | G1SN68 | #N/A | QARS1 | RABBIT | #N/A | -4.59626 | -2.90379 | 0.30624 | -2.53602 | 1.18937 | 0 | -0.07477 | 0.18399 | 0.47108 | 2.786388 | 2.99489 | 3.3704 | 0.02009 | 0.00718 | 0.11473 | 0.07947 | 7 | 2.93229 | 0.55262 | 1.92357 | 1.518827 | Significant | Up in Mtub | QARS1 |
| 2296 | P08507 | #N/A | GPD1 | RABBIT | #N/A | #N/A | -2.07506 | -0.88164 | -1.87022 | 2.03738 | 0.824 | 1.7519 | -1.36721 | 2.43129 | 2.744443 | 2.90403 | 3.28806 | 0.02719 | 0.0095 | 0.124 | 0.08953 | 6 | 2.93229 | 0.55262 | 1.67458 | 1.306263 | Significant | Up in Mtub | GPD1 |
| 694 | G1TXJ3 | Receptor-type tyrosine-protein phosphatase alpha ( | PTPRA | RABBIT | -3.63363 | #N/A | #N/A | -0.86885 | -0.55467 | 2.73937 | 0.15077 | -0.06941 | 2.46556 | 0.00505 | 2.743983 | 2.48185 | 2.86413 | 0.04769 | 0.01879 | 0.14885 | 0.106 | 6 | 2.93229 | 0.55262 | 2.29199 | 1.720992 | Significant | Up in Mtub | PTPRA |
| 2017 | G1TM35 | #N/A | FIS1 | RABBIT | #N/A | #N/A | -2.54098 | #N/A | #N/A | 1.01614 | -0.53539 | 0.48479 | -0.5755 | 0.53539 | 2.726063 | 3.51867 | 3.44317 | 0.02448 | 0.01096 | 0.11931 | 0.09208 | 4 | 2.93229 | 0.55262 | 0.50019 | 0.522364 | Significant | Up in Mtub | FIS1 |
| 2582 | A0A5F9DHZ9 | #N/A | FRMD4A | RABBIT | #N/A | #N/A | #N/A | -1.37844 | #N/A | #N/A | 1.3208 | #N/A | #N/A | #N/A | 2.699237 | #N/A | 2.56752 | #N/A | 0.08459 | #N/A | 0.18738 | 0 | 2.93229 | 0.55262 | #N/A | 0.552617 | Not Significant | NS | #N/A |
| 2485 | B7NZM1 | #N/A | RA_m003_ | RABBIT | #N/A | #N/A | #N/A | -0.57787 | -3.99928 | 0.52023 | 0.71396 | 1.07913 | -0.68842 | #N/A | 2.6948 | 2.25571 | 2.69638 | 0.08709 | 0.03108 | 0.17659 | 0.12366 | 4 | 2.93229 | 0.55262 | 1.90294 | 1.33177 | Significant | Up in Mtub | RA_m003_ism4E071r |
| 1934 | G1TXC4 | #N/A | ANKFN1 | RABBIT | #N/A | -4.76136 | -0.22119 | #N/A | #N/A | 0.1732 | #N/A | -0.17375 | 0.69137 | 0.10939 | 2.691327 | 1.90039 | 2.33142 | 0.13018 | 0.05285 | 0.21499 | 0.15205 | 4 | 2.93229 | 0.55262 | 2.67415 | 1.776764 | Not Significant | NS | #N/A |
| 2004 | A0A5F9DL43 | #N/A | #N/A | RABBIT | #N/A | -1.92391 | 0.17065 | #N/A | #N/A | #N/A | 1.81264 | #N/A | #N/A | #N/A | 2.689271 | 1.48256 | 2.22957 | 0.37778 | 0.09081 | 0.35939 | 0.19199 | 1 | 2.93229 | 0.55262 | 2.19359 | 0.969924 | Not Significant | NS | #N/A |
| 1218 | G1U7U8 | Guanine nucleotide-binding protein G(o) subunit al | #N/A | RABBIT | -5.00916 | -2.54578 | #N/A | -0.78802 | -0.5305 | 0.87867 | 0.59277 | 0.44583 | 0 | 0.32161 | 2.666137 | 2.89277 | 3.25215 | 0.02322 | 0.00876 | 0.11776 | 0.0849 | 7 | 2.93229 | 0.55262 | 1.88766 | 1.49352 | Significant | Up in Mtub | #N/A |
| 2183 | G1SI29 | #N/A | TUFM | RABBIT | #N/A | #N/A | -0.5069 | -3.50143 | #N/A | 0.60442 | #N/A | 1.36015 | 1.23979 | -0.60442 | 2.654151 | 2.33153 | 2.76256 | 0.08012 | 0.02827 | 0.1731 | 0.11898 | 4 | 2.93229 | 0.55262 | 1.72786 | 1.230744 | Significant | Up in Mtub | TUFM |
| 2759 | P51662 | #N/A | ANXA1 AN | RABBIT | #N/A | #N/A | #N/A | -3.1623 | -1.80959 | 0.63612 | 0.06565 | -0.51533 | 0 | 0.64933 | 2.653097 | 5.18334 | 4.77946 | 0.00352 | 0.00142 | 0.06676 | 0.04508 | 5 | 2.93229 | 0.55262 | 0.37427 | 0.440201 | Significant | Up in Mtub | ANXA1 ANX1 |
| 1245 | A0A5F9CAX1 | Biliverdin reductase B OS=Oryctolagus cuniculus O | #N/A | RABBIT | -2.22995 | -1.41952 | -0.04781 | -1.61242 | -2.23156 | 1.79663 | 0.83283 | 0.15409 | 0.96535 | 1.94523 | 2.647078 | 5.10754 | 5.23331 | 0.00092 | 0.00029 | 0.04345 | 0.02295 | 8 | 2.93229 | 0.55262 | 0.67151 | 0.639617 | Significant | Up in Mtub | #N/A |
| 2398 | A0A5F9CQX8 | #N/A | MAGI1 | RABBIT | #N/A | #N/A | #N/A | -1.34814 | #N/A | #N/A | #N/A | #N/A | #N/A | 1.2905 | 2.638635 | #N/A | 2.50988 | #N/A | 0.08889 | #N/A | 0.1902 | 0 | 2.93229 | 0.55262 | #N/A | 0.552617 | Not Significant | NS | #N/A |
| 2138 | U3KMU7 | #N/A | EHD1 | RABBIT | #N/A | #N/A | -0.97019 | -1.85171 | -2.59528 | 2.60857 | #N/A | -0.06437 | 0.53718 | 0.24635 | 2.637658 | 3.23076 | 3.59169 | 0.02319 | 0.00717 | 0.11776 | 0.07947 | 5 | 2.93229 | 0.55262 | 1.14264 | 0.924532 | Significant | Up in Mtub | EHD1 |
| 2330 | A0A5F9C953 | #N/A | SDCBP | RABBIT | #N/A | #N/A | -0.06342 | -0.13037 | 0.05935 | 2.56487 | #N/A | #N/A | #N/A | #N/A | 2.609678 | 23.4885 | 3.92066 | 0.00181 | 0.01148 | 0.05406 | 0.09305 | 2 | 2.93229 | 0.55262 | 0.00926 | 0.33229 | Significant | Up in Mtub | SDCBP |
| 2851 | G1TIF8 | #N/A | APOD | RABBIT | #N/A | #N/A | #N/A | #N/A | -1.30887 | 1.29549 | #N/A | #N/A | #N/A | #N/A | 2.604363 | #N/A | 2.47728 | #N/A | 0.09144 | #N/A | 0.19284 | 0 | 2.93229 | 0.55262 | #N/A | 0.552617 | Not Significant | NS | #N/A |
| 1992 | G1TBI4 | #N/A | BROX | RABBIT | #N/A | #N/A | #N/A | -2.71578 | #N/A | -1.88561 | -0.30417 | 0.82916 | 0.30417 | 0.48431 | 2.601355 | 2.21511 | 2.50748 | 0.09111 | 0.04087 | 0.18149 | 0.13388 | 4 | 2.93229 | 0.55262 | 1.14928 | 0.896899 | Significant | Up in Mtub | BROX |
| 1253 | G1TP30 | Ran GTPase activating protein 1 OS=Oryctolagus cu | RANGAP1 | RABBIT | -0.64777 | -2.45358 | -1.96514 | -1.17821 | -1.08042 | 0.69522 | 1.08685 | 0.59507 | 1.41852 | 1.79336 | 2.582827 | 6.53405 | 6.19842 | 0.00018 | 6.9E-05 | 0.03578 | 0.01186 | 8 | 2.93229 | 0.55262 | 0.39063 | 0.434078 | Significant | Up in Mtub | RANGAP1 |
| 1523 | G1SF78 | Vacuolar protein sorting 13 homolog A OS=Oryctola | VPS13A | RABBIT | 0.21881 | #N/A | -0.21884 | #N/A | -0.22053 | #N/A | #N/A | 2.5047 | #N/A | #N/A | 2.578218 | 8.81954 | 3.74997 | 0.01261 | 0.01363 | 0.09752 | 0.09774 | 2 | 2.93229 | 0.55262 | 0.06409 | 0.354524 | Significant | Up in Mtub | VPS13A |
| 1043 | G1SER8 | Profilin OS=Oryctolagus cuniculus OX=9986 GN=PF | PFN1 | RABBIT | -3.03261 | -1.62367 | -0.70065 | -0.85313 | -1.1529 | 1.81688 | 1.20221 | 0.77345 | 0.79549 | 0.91837 | 2.573872 | 5.55418 | 5.53247 | 0.00054 | 0.00018 | 0.03578 | 0.01896 | 8 | 2.93229 | 0.55262 | 0.53688 | 0.541099 | Significant | Up in Mtub | PFN1 |

| | | | | | | | | | | | | | | | | | | | | | | | | | | | | | |
|---|---|---|---|---|---|---|---|---|---|---|---|---|---|---|---|---|---|---|---|---|---|---|---|---|---|---|---|---|---|
| 1766 | G1TA59 | #N/A | IDH3A | RABBIT | #N/A | -0.83288 | -0.79203 | -0.82041 | -2.43294 | 0.76277 | #N/A | 2.26805 | 1.15664 | 1.2002 | 2.566477 | 4.96116 | 4.93491 | 0.00255 | 0.00083 | 0.05406 | 0.036 | 6 | 2.93229 | 0.55262 | 0.53523 | 0.540936 | Significant | Up in Mtub | IDH3A |
| 298 | G1SVM1 | Major vault protein OS=Oryctolagus cuniculus OX=9 | MVP | RABBIT | -2.32196 | -3.06526 | 0.0362 | -4.10988 | 0.19391 | -0.07515 | 1.84413 | 0.55743 | 0.07515 | 1.03327 | 2.540365 | 2.75762 | 3.07995 | 0.02477 | 0.01054 | 0.11969 | 0.09154 | 8 | 2.93229 | 0.55262 | 2.1216 | 1.700763 | Significant | Up in Mtub | MVP |
| 1574 | G1TE90 | Atlastin-1 OS=Oryctolagus cuniculus OX=9986 GN= | ATL1 | RABBIT | -4.45692 | -2.47297 | -0.8608 | -0.02447 | -2.25149 | 0.34764 | 1.23437 | 0.07943 | 0.93363 | -0.03316 | 2.525713 | 3.16533 | 3.48512 | 0.01329 | 0.00515 | 0.09752 | 0.06702 | 8 | 2.93229 | 0.55262 | 1.59174 | 1.313022 | Significant | Up in Mtub | ATL1 |
| 1581 | G1T7G9 | WD repeat domain 13 OS=Oryctolagus cuniculus O | WDR13 | RABBIT | -3.27565 | -4.11555 | -0.09653 | 0.20955 | -0.61925 | 1.51967 | -0.38674 | 1.01847 | 1.31361 | 1.23768 | 2.520025 | 2.65922 | 2.9772 | 0.02884 | 0.01266 | 0.12702 | 0.09521 | 8 | 2.93229 | 0.55262 | 2.24513 | 1.791159 | Significant | Up in Mtub | WDR13 |
| 1135 | A0A5F9CDU4 | MAP kinase-activating death domain protein OS=Or | MADD | RABBIT | #N/A | #N/A | #N/A | -2.22904 | #N/A | 0.06121 | #N/A | 0.81765 | -0.06121 | #N/A | 2.501589 | 4.55113 | 3.34122 | 0.04504 | 0.02095 | 0.14686 | 0.10939 | 2 | 2.93229 | 0.55262 | 0.2266 | 0.420419 | Significant | Up in Mtub | MADD |
| 2518 | G1T1R9 | #N/A | LRGUK | RABBIT | #N/A | #N/A | #N/A | -1.85108 | #N/A | 1.9782 | #N/A | -0.80856 | 0.7442 | #N/A | 2.489026 | 1.54364 | 2.03751 | 0.26266 | 0.09795 | 0.30553 | 0.19757 | 2 | 2.93229 | 0.55262 | 1.94997 | 1.119232 | Not Significant | NS | #N/A |
| 1177 | G1SIH1 | Proteasome activator complex subunit 2 OS=Oryctc | PSME2 | RABBIT | -0.39041 | 0.07783 | #N/A | -1.58607 | -1.56019 | 0 | 2.27073 | 2.05024 | 1.86152 | 1.93082 | 2.487372 | 4.18114 | 4.3777 | 0.00413 | 0.0014 | 0.06755 | 0.04508 | 7 | 2.93229 | 0.55262 | 0.78646 | 0.717426 | Significant | Up in Mtub | PSME2 |
| 145 | G1SNS5 | Rab GDP dissociation inhibitor OS=Oryctolagus cun | #N/A | RABBIT | -1.07043 | -0.97138 | -0.1751 | -0.17298 | -0.24985 | 2.63535 | 2.01369 | 0.05098 | 2.56757 | 2.51287 | 2.484042 | 4.69572 | 4.8339 | 0.00155 | 0.00053 | 0.05271 | 0.02771 | 8 | 2.93229 | 0.55262 | 0.69961 | 0.660181 | Significant | Up in Mtub | #N/A |
| 1329 | A0A5F9C6U9 | Vesicle trafficking 1 OS=Oryctolagus cuniculus OX= | VTA1 | RABBIT | #N/A | #N/A | -2.97955 | 0.02228 | -2.1567 | 1.54484 | 0.85796 | #N/A | -0.07992 | #N/A | 2.478953 | 2.45004 | 2.86907 | 0.07044 | 0.02428 | 0.16349 | 0.11483 | 4 | 2.93229 | 0.55262 | 1.53561 | 1.119812 | Significant | Up in Mtub | VTA1 |
| 647 | G1U0J2 | RRM domain-containing protein OS=Oryctolagus cu | #N/A | RABBIT | -2.31794 | #N/A | #N/A | #N/A | #N/A | -0.54208 | -0.21733 | 0.15296 | 0.45746 | 0.94518 | 2.477179 | 3.90652 | 3.46021 | 0.01745 | 0.01071 | 0.1103 | 0.09154 | 4 | 2.93229 | 0.55262 | 0.33508 | 0.427098 | Significant | Up in Mtub | #N/A |
| 1826 | A0A1Y1BBR2 | #N/A | #N/A | RABBIT | #N/A | -0.50635 | -1.30901 | -0.93168 | -0.01338 | -2.34242 | 4.44156 | 1.83542 | 1.92616 | 3.06746 | 2.475744 | 1.88994 | 2.18588 | 0.10068 | 0.05389 | 0.18996 | 0.15373 | 7 | 2.93229 | 0.55262 | 3.81331 | 2.850664 | Not Significant | NS | #N/A |
| 1199 | G1TP15 | Proteasome 26S subunit, non-ATPase 3 OS=Orycto | PSMD3 | RABBIT | -5.068 | -2.73425 | 0.15059 | -0.17707 | -2.27599 | 0.02006 | 0.27168 | 0.66623 | 0.10469 | 1.17731 | 2.468936 | 2.54074 | 2.85033 | 0.03467 | 0.01588 | 0.13411 | 0.09934 | 8 | 2.93229 | 0.55262 | 2.36068 | 1.87572 | Significant | Up in Mtub | PSMD3 |
| 1255 | G1TED6 | Annexin OS=Oryctolagus cuniculus OX=9986 GN=A | ANXA5 | RABBIT | -5.21544 | -3.91725 | -0.01208 | -1.05619 | 0.30755 | 0.50224 | 1.00524 | 0.04969 | -0.11405 | 0.93075 | 2.453457 | 2.18582 | 2.4768 | 0.06031 | 0.03087 | 0.15554 | 0.12309 | 8 | 2.93229 | 0.55262 | 3.14969 | 2.453094 | Significant | Up in Mtub | ANXA5 |
| 1254 | A0A5F9CTM9 | AHNAK nucleoprotein OS=Oryctolagus cuniculus O | #N/A | RABBIT | -1.11218 | -1.19152 | #N/A | #N/A | -1.96116 | 0 | 2.33285 | 1.63182 | 0.11011 | #N/A | 2.440316 | 3.40369 | 3.6657 | 0.01917 | 0.00644 | 0.11259 | 0.07464 | 5 | 2.93229 | 0.55262 | 0.8812 | 0.759735 | Significant | Up in Mtub | #N/A |
| 1477 | G1SZP0 | Target of myb1 membrane trafficking protein OS=Or | TOM1 | RABBIT | #N/A | -3.96986 | #N/A | -0.58348 | #N/A | 0 | -0.90656 | 0.25731 | 0.50293 | 0.93192 | 2.433791 | 2.35639 | 2.69521 | 0.06504 | 0.02749 | 0.15932 | 0.11898 | 5 | 2.93229 | 0.55262 | 1.52396 | 1.16489 | Significant | Up in Mtub | TOM1 |
| 1197 | A0A5F9DS41 | Adenosine deaminase RNA specific OS=Oryctolagu | ADAR | RABBIT | -3.05964 | -2.81359 | -1.84522 | 0.39334 | -1.86784 | -0.11252 | 0.97973 | 1.14353 | 0.80284 | 0.11252 | 2.423807 | 3.68579 | 3.95413 | 0.00617 | 0.00228 | 0.07337 | 0.05019 | 8 | 2.93229 | 0.55262 | 1.08112 | 0.939366 | Significant | Up in Mtub | ADAR |
| 1001 | G1TGW1 | Eukaryotic translation initiation factor 5 OS=Oryctol | #N/A | RABBIT | -3.93068 | -3.22646 | -0.41915 | 0.27055 | -1.39345 | 1.66156 | -0.26545 | 0.20108 | 0.969 | 0.81722 | 2.416518 | 2.78067 | 3.08909 | 0.0239 | 0.01037 | 0.11927 | 0.09154 | 8 | 2.93229 | 0.55262 | 1.88809 | 1.529885 | Significant | Up in Mtub | #N/A |
| 2514 | P12530 | #N/A | ALOX15 | RABBIT | #N/A | #N/A | #N/A | -4.74779 | 0.05371 | 0.19513 | 0.65994 | -0.53282 | #N/A | -0.06709 | 2.41083 | 1.58925 | 1.96639 | 0.18721 | 0.09037 | 0.26045 | 0.19156 | 4 | 2.93229 | 0.55262 | 3.06823 | 2.004152 | Not Significant | NS | #N/A |
| 2501 | A0A5F9CDP0 | #N/A | LANCL2 | RABBIT | #N/A | #N/A | #N/A | -2.18527 | #N/A | 1.06706 | -0.38377 | -0.11899 | 0.05463 | 0.50841 | 2.410741 | 3.84942 | 3.38633 | 0.01831 | 0.01183 | 0.11171 | 0.0939 | 4 | 2.93229 | 0.55262 | 0.32684 | 0.422339 | Significant | Up in Mtub | LANCL2 |
| 1592 | G1U1F6 | Heat shock protein family B (small) member 6 OS=( | HSPB6 | RABBIT | -1.91294 | -0.48827 | -0.80694 | -1.16547 | -1.29222 | 1.65196 | 1.09925 | 1.32198 | 1.5975 | 0.68609 | 2.404527 | 8.05403 | 6.81428 | 4.2E-05 | 3E-05 | 0.02014 | 0.01186 | 8 | 2.93229 | 0.55262 | 0.22283 | 0.311286 | Significant | Up in Mtub | HSPB6 |
| 1378 | G1SMB8 | Prune exopolyphosphatase 1 OS=Oryctolagus cuni | PRUNE1 | RABBIT | -0.7768 | -1.06918 | -0.24774 | -1.167 | 0.40501 | 0.72288 | -0.73278 | 7.59821 | 0.93393 | 0.60651 | 2.396893 | 1.59672 | 1.83387 | 0.149 | 0.09401 | 0.23045 | 0.19607 | 8 | 2.93229 | 0.55262 | 5.63355 | 4.270726 | Not Significant | NS | #N/A |
| 1813 | A0A5F9DM87 | #N/A | RUVBL2 | RABBIT | #N/A | -2.31564 | -0.23538 | -1.16403 | -4.42717 | 0 | 0.27214 | 0.30372 | 0.28088 | 0.93967 | 2.394836 | 2.94576 | 3.2613 | 0.02154 | 0.00863 | 0.11776 | 0.0849 | 7 | 2.93229 | 0.55262 | 1.46874 | 1.198275 | Significant | Up in Mtub | RUVBL2 |
| 481 | A0A5F9CMJ6 | ADP-ribosylation factor OS=Oryctolagus cuniculus | #N/A | RABBIT | -4.05976 | -3.64507 | 0.15271 | -0.0397 | -1.17334 | 1.13493 | -0.4429 | 0.47407 | 0.97492 | 1.02307 | 2.385847 | 2.55 | 2.85186 | 0.03417 | 0.01583 | 0.13313 | 0.09934 | 8 | 2.93229 | 0.55262 | 2.1885 | 1.749719 | Significant | Up in Mtub | #N/A |
| 680 | G1T6T5 | Cysteine and histidine-rich domain-containing prote | CHORDC1 | RABBIT | -2.39894 | -3.7871 | -1.02349 | 0.65797 | -1.37516 | 1.16806 | -0.68876 | 0.62439 | 1.00921 | 1.87178 | 2.38228 | 2.80006 | 3.10412 | 0.0232 | 0.0101 | 0.11776 | 0.09149 | 8 | 2.93229 | 0.55262 | 1.80964 | 1.472478 | Significant | Up in Mtub | CHORDC1 |
| 1029 | G1T888 | Ankyrin repeat and FYVE domain containing 1 OS=O | ANKFY1 | RABBIT | -1.8951 | -1.4483 | -1.19983 | -0.34478 | -1.36207 | 2.89177 | 1.10336 | 0.22278 | 0.94139 | 0.46355 | 2.374586 | 4.44911 | 4.58912 | 0.00214 | 0.00079 | 0.05406 | 0.0357 | 8 | 2.93229 | 0.55262 | 0.71215 | 0.669356 | Significant | Up in Mtub | ANKFY1 |
| 996 | A0A5F9CH20 | 26S proteasome regulatory subunit 6B OS=Oryctola | #N/A | RABBIT | -3.30533 | -2.31594 | -2.09443 | 0.49573 | -1.71765 | #N/A | 0 | 0.56582 | 0.66118 | 1.10695 | 2.37101 | 3.20446 | 3.4987 | 0.01497 | 0.0058 | 0.10089 | 0.07001 | 7 | 2.93229 | 0.55262 | 1.21659 | 1.020566 | Significant | Up in Mtub | #N/A |
| 1274 | G1TWP4 | Valine--tRNA ligase OS=Oryctolagus cuniculus OX= | VARS1 | RABBIT | -3.75754 | -1.6546 | -2.18831 | 0.56438 | -2.702 | 0.77451 | 0.3864 | 0.50456 | -0.3864 | 0.79867 | 2.363161 | 3.15419 | 3.44684 | 0.01351 | 0.00551 | 0.09752 | 0.06816 | 8 | 2.93229 | 0.55262 | 1.4033 | 1.175126 | Significant | Up in Mtub | VARS1 |
| 601 | G1SPZ7 | Glutathione peroxidase OS=Oryctolagus cuniculus | #N/A | RABBIT | -1.63673 | -1.93995 | -0.91189 | -0.49271 | -0.84591 | 2.06903 | 0.60776 | 0.37071 | 1.68548 | 1.24702 | 2.361439 | 5.66728 | 5.47043 | 0.00047 | 0.0002 | 0.03578 | 0.01896 | 8 | 2.93229 | 0.55262 | 0.43405 | 0.465855 | Significant | Up in Mtub | #N/A |
| 2180 | A0A5F9CGG2 | #N/A | RRBP1 | RABBIT | #N/A | #N/A | -1.41508 | -2.18795 | #N/A | #N/A | #N/A | 0.60844 | 0 | 1.04418 | 2.352388 | 4.84465 | 3.99422 | 0.01678 | 0.00733 | 0.10856 | 0.08009 | 3 | 2.93229 | 0.55262 | 0.28293 | 0.416233 | Significant | Up in Mtub | RRBP1 |
| 2811 | A0A5F9DQ30 | #N/A | LRSAM1 | RABBIT | #N/A | #N/A | #N/A | #N/A | -1.18016 | 1.16678 | #N/A | #N/A | #N/A | #N/A | 2.34694 | #N/A | 2.23241 | #N/A | 0.1138 | #N/A | 0.21301 | 0 | 2.93229 | 0.55262 | #N/A | 0.552617 | Not Significant | NS | #N/A |
| 1570 | A0A5F9C5U2 | F-box protein 6 OS=Oryctolagus cuniculus OX=998 | FBXO6 | RABBIT | -2.27069 | -2.63001 | -3.48089 | 0.78963 | -1.96746 | 0.64465 | 0.8778 | -0.2134 | 0.71397 | 0.14903 | 2.346295 | 3.13152 | 3.42209 | 0.01398 | 0.00575 | 0.09783 | 0.06994 | 8 | 2.93229 | 0.55262 | 1.40344 | 1.175231 | Significant | Up in Mtub | FBXO6 |
| 2303 | G1U2C8 | #N/A | MFHAS1 | RABBIT | #N/A | #N/A | -1.08518 | #N/A | #N/A | #N/A | 1.25583 | #N/A | #N/A | #N/A | 2.341013 | #N/A | 2.22678 | #N/A | 0.11439 | #N/A | 0.21343 | 0 | 2.93229 | 0.55262 | #N/A | 0.552617 | Not Significant | NS | #N/A |
| 1698 | A0A5F9C865 | #N/A | RELCH | RABBIT | #N/A | -0.25299 | #N/A | -0.3604 | #N/A | #N/A | #N/A | 0.2384 | 3.82601 | #N/A | 2.338903 | 1.30329 | 1.82946 | 0.32232 | 0.12766 | 0.33441 | 0.22682 | 2 | 2.93229 | 0.55262 | 3.22062 | 1.634468 | Not Significant | NS | #N/A |
| 2447 | O46426 | #N/A | #N/A | RABBIT | #N/A | #N/A | #N/A | #N/A | -2.2215 | #N/A | 0.39077 | 0.3308 | -0.39077 | #N/A | 2.331763 | 4.64285 | 3.17217 | 0.04339 | 0.02523 | 0.14581 | 0.11552 | 2 | 2.93229 | 0.55262 | 0.18917 | 0.405244 | Significant | Up in Mtub | #N/A |
| 813 | A0A5F9DNN9 | Chloride intracellular channel protein OS=Oryctola | CLIC4 | RABBIT | -1.39974 | -2.15959 | -0.24885 | -0.94876 | -2.56582 | 1.49585 | 0.4195 | 0.71333 | 0.86815 | 0.80076 | 2.324068 | 5.15349 | 5.09446 | 0.00087 | 0.00035 | 0.04345 | 0.02333 | 8 | 2.93229 | 0.55262 | 0.50843 | 0.520285 | Significant | Up in Mtub | CLIC4 |
| 892 | A0A5F9DN55 | Ubiquitin carboxyl-terminal hydrolase 7 OS=Oryctol | USP7 | RABBIT | #N/A | -3.12726 | 0.17065 | #N/A | -0.24572 | #N/A | 0.84806 | 1.64282 | #N/A | #N/A | 2.312884 | 1.687 | 2.13082 | 0.19019 | 0.07765 | 0.26255 | 0.18205 | 3 | 2.93229 | 0.55262 | 2.25559 | 1.413821 | Not Significant | NS | #N/A |
| 1565 | A5HC45 | Cathepsin D (Fragment) OS=Oryctolagus cuniculus | #N/A | RABBIT | #N/A | -1.13774 | -3.15475 | -0.98274 | -2.33871 | 1.03831 | 0.39817 | -0.06437 | 0.57318 | 0.07604 | 2.30775 | 4.58132 | 4.59488 | 0.00254 | 0.00101 | 0.05406 | 0.03918 | 7 | 2.93229 | 0.55262 | 0.56388 | 0.560552 | Significant | Up in Mtub | #N/A |
| 540 | G1U4K3 | ADP-ribosylation factor OS=Oryctolagus cuniculus | ARF5 | RABBIT | -3.21109 | -2.27285 | -0.20993 | 0.32294 | -1.00018 | 1.19996 | -1.43732 | 0.38215 | 4.10834 | 0.89061 | 2.30297 | 2.07908 | 2.35395 | 0.07122 | 0.03835 | 0.16445 | 0.12996 | 8 | 2.93229 | 0.55262 | 3.06741 | 2.392889 | Significant | Up in Mtub | ARF5 |
| 1482 | A0A5F9DQK5 | Eukaryotic translation initiation factor 4 gamma 3 O | EIF4G3 | RABBIT | -3.96886 | #N/A | -2.78723 | -0.02282 | #N/A | 1.01338 | 0.1986 | -2.42588 | -0.03482 | 1.42612 | 2.295117 | 1.85708 | 2.16604 | 0.11268 | 0.05871 | 0.20012 | 0.16071 | 6 | 2.93229 | 0.55262 | 2.86386 | 2.105124 | Not Significant | NS | #N/A |
| 879 | G1U8C4 | Proteasome activator complex subunit 1 OS=Oryctc | PSME1 | RABBIT | 0.01166 | -0.00581 | #N/A | -0.7723 | -0.44326 | -0.60134 | 3.7763 | 3.05413 | 3.14166 | 0.57952 | 2.292479 | 2.35175 | 2.65937 | 0.05096 | 0.02406 | 0.15026 | 0.11457 | 7 | 2.93229 | 0.55262 | 2.11162 | 1.651358 | Significant | Up in Mtub | PSME1 |
| 1164 | P27170 | Serum paraoxonase/arylesterase 1 OS=Oryctolagus | PON1 PON | RABBIT | -2.03438 | -1.91017 | #N/A | -0.68985 | 0.40205 | 2.89189 | 2.08291 | -0.39383 | #N/A | 0.32946 | 2.285694 | 2.40003 | 2.73203 | 0.05329 | 0.0233 | 0.15348 | 0.11255 | 6 | 2.93229 | 0.55262 | 1.81398 | 1.399899 | Significant | Up in Mtub | PON1 PON |
| 1691 | G1TEQ0 | #N/A | #N/A | RABBIT | #N/A | -0.29217 | #N/A | -0.50618 | #N/A | #N/A | #N/A | #N/A | 3.32304 | 0.44854 | 2.284964 | 1.58543 | 2.11173 | 0.25375 | 0.08919 | 0.30091 | 0.1902 | 2 | 2.93229 | 0.55262 | 2.07713 | 1.170794 | Not Significant | NS | #N/A |
| 2646 | G1SVZ8 | #N/A | QNG1 | RABBIT | #N/A | #N/A | #N/A | -1.16358 | #N/A | #N/A | #N/A | #N/A | 1.10594 | #N/A | 2.269515 | #N/A | 2.15877 | #N/A | 0.12179 | #N/A | 0.22013 | 0 | 2.93229 | 0.55262 | #N/A | 0.552617 | Not Significant | NS | #N/A |
| 2857 | G1SVK5 | #N/A | S100A4 | RABBIT | #N/A | #N/A | #N/A | #N/A | -1.96826 | -0.62305 | 1.76824 | -1.22258 | 0.92025 | 0.62305 | 2.261438 | 1.7122 | 1.99335 | 0.16202 | 0.08686 | 0.24125 | 0.18876 | 4 | 2.93229 | 0.55262 | 1.45371 | 1.072557 | Not Significant | NS | #N/A |
| 1847 | G1SHK8 | #N/A | GLS | RABBIT | #N/A | -2.20992 | -2.0281 | -0.78955 | #N/A | 0.71218 | #N/A | 1.48627 | 0.14037 | 0 | 2.260564 | 4.1338 | 4.07548 | 0.00905 | 0.00362 | 0.09065 | 0.05856 | 5 | 2.93229 | 0.55262 | 0.51265 | 0.527422 | Significant | Up in Mtub | GLS |
| 767 | G1SQA8 | ATP synthase subunit beta OS=Oryctolagus cunicul | ATP5F1B | RABBIT | -4.85488 | -0.83661 | 0.04297 | 0.07004 | -0.2288 | 1.2671 | -0.13048 | 1.81264 | 1.67299 | 0.86821 | 2.259546 | 2.25865 | 2.53954 | 0.05384 | 0.02762 | 0.15365 | 0.11898 | 8 | 2.93229 | 0.55262 | 2.50199 | 1.979121 | Significant | Up in Mtub | ATP5F1B |
| 2072 | A0A5F9CH88 | #N/A | RUVBL1 | RABBIT | #N/A | -4.1288 | -0.21625 | -0.06204 | -0.90697 | 0.15487 | 0 | 0.35945 | 1.9702 | 2.15955 | 2.257328 | 2.28017 | 2.58231 | 0.05662 | 0.02745 | 0.15449 | 0.11898 | 7 | 2.93229 | 0.55262 | 2.17792 | 1.698084 | Significant | Up in Mtub | RUVBL1 |
| 883 | G1TK32 | Phosphofructokinase domain-containing protein OS | #N/A | RABBIT | #N/A | -2.937 | -1.64329 | -1.64411 | -1.64748 | 0.16217 | 0.19639 | 0.15493 | 0.92647 | 0 | 2.255965 | 6.66057 | 5.74372 | 0.00029 | 0.00019 | 0.03578 | 0.01896 | 7 | 2.93229 | 0.55262 | 0.25494 | 0.342819 | Significant | Up in Mtub | #N/A |
| 2589 | G1TXV6 | #N/A | SENP8 | RABBIT | #N/A | #N/A | #N/A | -1.63392 | #N/A | 2.20811 | -1.74442 | -1.37471 | 2.68894 | 1.31035 | 2.251575 | 1.00149 | 1.25925 | 0.37326 | 0.24868 | 0.35712 | 0.31654 | 4 | 2.93229 | 0.55262 | 4.21212 | 2.664189 | Not Significant | NS | #N/A |
| 1224 | G1TM00 | VPS26, retromer complex component A OS=Orycto | VPS26A | RABBIT | #N/A | -1.85485 | -1.27025 | #N/A | #N/A | 0.36467 | 2.42853 | -0.15575 | 0.78695 | 0 | 2.247427 | 2.83153 | 3.05808 | 0.03661 | 0.01579 | 0.13586 | 0.09934 | 5 | 2.93229 | 0.55262 | 0.89998 | 0.771569 | Significant | Up in Mtub | VPS26A |
| 2613 | G1TD24 | #N/A | ARHGAP35 | RABBIT | #N/A | #N/A | #N/A | -1.85762 | #N/A | 0.65512 | #N/A | -0.06437 | -0.25765 | 1.22168 | 2.246319 | 2.95393 | 2.82141 | 0.05984 | 0.03067 | 0.1554 | 0.12309 | 3 | 2.93229 | 0.55262 | 0.46263 | 0.507109 | Significant | Up in Mtub | ARHGAP35 |
| 2783 | A0A5F9CCU3 | #N/A | LAP3 | RABBIT | #N/A | #N/A | #N/A | #N/A | -1.92874 | -0.55862 | 1.11431 | -0.44913 | 0.38476 | 1.08913 | 2.244832 | 2.5466 | 2.62942 | 0.06353 | 0.03424 | 0.15826 | 0.1262 | 4 | 2.93229 | 0.55262 | 0.64754 | 0.607388 | Significant | Up in Mtub | LAP3 |
| 686 | A0A4P1LX85 | Eukaryotic translation initiation factor 3 subunit B O | EIF3B EIF3 | RABBIT | -2.77416 | -2.05643 | -1.2525 | #N/A | -0.74329 | 0.81903 | 0.88463 | 0.63944 | 0 | 0.34744 | 2.244701 | 5.17086 | 4.94292 | 0.00129 | 0.0006 | 0.04885 | 0.02841 | 7 | 2.93229 | 0.55262 | 0.41877 | 0.458287 | Significant | Up in Mtub | EIF3B EIF3S9 |
| 2281 | A0A5F9C965 | #N/A | NAA15 | RABBIT | #N/A | #N/A | -2.02185 | #N/A | #N/A | #N/A | -0.52002 | 0.42927 | 0 | 0.95657 | 2.238301 | 3.18903 | 2.91262 | 0.04975 | 0.02724 | 0.14967 | 0.11898 | 3 | 2.93229 | 0.55262 | 0.3941 | 0.472455 | Significant | Up in Mtub | NAA15 |
| 2732 | G1T4V2 | #N/A | MTARC1 | RABBIT | #N/A | #N/A | #N/A | -1.99795 | -1.26122 | 1.78346 | -0.68914 | 0.62477 | 0.71399 | #N/A | 2.237858 | 2.82483 | 3.05258 | 0.04759 | 0.01874 | 0.14885 | 0.106 | 4 | 2.93229 | 0.55262 | 0.83679 | 0.71659 | Significant | Up in Mtub | MTARC1 |
| 1127 | A0A5F9CB44 | AP2 associated kinase 1 OS=Oryctolagus cuniculus | AAK1 | RABBIT | -3.62973 | #N/A | -0.92646 | 0.37435 | -2.78607 | #N/A | -0.05638 | -0.00798 | 0.75711 | 1.27641 | 2.234267 | 2.33002 | 2.65467 | 0.05864 | 0.02644 | 0.15505 | 0.11767 | 6 | 2.93229 | 0.55262 | 1.83899 | 1.416703 | Significant | Up in Mtub | AAK1 |
| 1833 | A0A5F9DQX8 | #N/A | #N/A | RABBIT | #N/A | -6.08129 | -2.77773 | 0.48953 | -0.39906 | 0.83585 | -1.51078 | -0.06437 | 0.44428 | 0.50135 | 2.233403 | 1.62732 | 1.88695 | 0.14769 | 0.08871 | 0.2295 | 0.19011 | 7 | 2.93229 | 0.55262 | 4.18577 | 3.113161 | Not Significant | NS | #N/A |
| 1967 | A0A5F9D3I3 | #N/A | SUCLG1 | RABBIT | #N/A | -2.58428 | -1.61894 | -0.48319 | #N/A | 1.42493 | #N/A | 1.00464 | 0.23951 | 0 | 2.22941 | 3.47487 | 3.62313 | 0.01776 | 0.00685 | 0.11065 | 0.07882 | 5 | 2.93229 | 0.55262 | 0.70565 | 0.649076 | Significant | Up in Mtub | SUCLG1 |
| 2806 | A0A5F9D479 | #N/A | #N/A | RABBIT | #N/A | #N/A | #N/A | #N/A | -1.11209 | 1.09871 | #N/A | #N/A | #N/A | #N/A | 2.210794 | #N/A | 2.10291 | #N/A | 0.12831 | #N/A | 0.22682 | 0 | 2.93229 | 0.55262 | #N/A | 0.552617 | Not Significant | NS | #N/A |
| 19 | P29751 | Actin, cytoplasmic 1 OS=Oryctolagus cuniculus OX | ACTB | RABBIT | -0.30389 | -3.5518 | -3.20147 | -2.99852 | 0.30217 | 1.07775 | 0.98847 | 0.76028 | -2.66731 | 1.11284 | 2.205108 | 2.02315 | 2.2883 | 0.07768 | 0.04304 | 0.17105 | 0.13846 | 8 | 2.93229 | 0.55262 | 2.9699 | 2.321533 | Significant | Up in Mtub | ACTB |
| 1743 | A0A5F9DPZ5 | #N/A | TBCD | RABBIT | #N/A | -0.18443 | #N/A | #N/A | -0.01338 | #N/A | #N/A | #N/A | 2.09923 | #N/A | 2.198133 | 14.8391 | 2.78333 | 0.04284 | 0.05061 | 0.14581 | 0.15092 | 1 | 2.93229 | 0.55262 | 0.01463 | 0.415804 | Not Significant | NS | #N/A |
| 1731 | G1TDU0 | #N/A | KIF5B | RABBIT | #N/A | -2.64443 | -1.1312 | -1.65511 | -1.38748 | 0.28406 | 0 | 0.53596 | 0.47808 | 1.14472 | 2.193122 | 6.07322 | 5.39385 | 0.0005 | 0.00031 | 0.03578 | 0.02295 | 7 | 2.93229 | 0.55262 | 0.28978 | 0.36738 | Significant | Up in Mtub | KIF5B |
| 928 | G1SE67 | Sepiapterin reductase OS=Oryctolagus cuniculus O | SPR | RABBIT | -4.79702 | -2.59914 | 1.91717 | -0.66426 | -1.53829 | 0.76637 | 1.77351 | 0.17901 | 0.75768 | -0.24338 | 2.182949 | 1.88733 | 2.14234 | 0.09582 | 0.05553 | 0.1866 | 0.15633 | 8 | 2.93229 | 0.55262 | 3.34452 | 2.595665 | Not Significant | NS | #N/A |
| 973 | Q9XS70 | Coronin-1B OS=Oryctolagus cuniculus OX=9986 GI | CORO1B | RABBIT | -1.1205 | -0.78781 | -0.554 | -0.86864 | -0.76329 | 1.92632 | 1.44203 | 0.66029 | 1.45717 | 1.3336 | 2.182729 | 9.78705 | 7.05622 | 1E-05 | 2.2E-05 | 0.00984 | 0.01186 | 8 | 2.93229 | 0.55262 | 0.12435 | 0.239219 | Significant | Up in Mtub | CORO1B |
| 2053 | G1T1U7 | #N/A | MTHFD1 | RABBIT | #N/A | -2.7976 | -0.30312 | -0.05764 | -1.36509 | 2.18401 | -0.54836 | 0.62453 | 1.34698 | 1.64673 | 2.181638 | 2.84676 | 3.12515 | 0.0248 | 0.01087 | 0.11969 | 0.09177 | 7 | 2.93229 | 0.55262 | 1.30512 | 1.08296 | Significant | Up in Mtub | MTHFD1 |
| 2605 | G1T457 | #N/A | IGFBP1 | RABBIT | #N/A | #N/A | #N/A | -1.11686 | #N/A | 1.05922 | #N/A | #N/A | #N/A | #N/A | 2.176079 | #N/A | 2.06989 | #N/A | 0.13235 | #N/A | 0.23203 | 0 | 2.93229 | 0.55262 | #N/A | 0.552617 | Not Significant | NS | #N/A |
| 1649 | G1T877 | Autophagy related 2B OS=Oryctolagus cuniculus O: | ATG2B | RABBIT | -2.75455 | #N/A | #N/A | -3.64419 | 0.53128 | #N/A | #N/A | -0.06437 | 0.50012 | #N/A | 2.173697 | 1.31521 | 1.71365 | 0.27993 | 0.13799 | 0.31436 | 0.23706 | 3 | 2.93229 | 0.55262 | 3.27786 | 1.930791 | Not Significant | NS | #N/A |
| 1564 | G1SF30 | Ribosomal protein L15 OS=Oryctolagus cuniculus ( | #N/A | RABBIT | -4.41411 | -3.09974 | 0.36011 | -0.83434 | #N/A | -0.15312 | #N/A | 0.50786 | 0.18408 | 0.15312 | 2.170004 | 1.99587 | 2.30702 | 0.09296 | 0.04667 | 0.18402 | 0.14576 | 6 | 2.93229 | 0.55262 | 2.3642 | 1.769497 | Significant | Up in Mtub | #N/A |
| 1169 | G1SN70 | Serpin family B member 9 OS=Oryctolagus cunicul | SERPINB9 | RABBIT | -1.984 | -2.37861 | #N/A | -0.09142 | #N/A | 0.03378 | 0.87475 | -0.32996 | 2.6934 | 0.15251 | 2.169575 | 2.45348 | 2.7509 | 0.04956 | 0.0226 | 0.14957 | 0.11217 | 6 | 2.93229 | 0.55262 | 1.46618 | 1.166275 | Significant | Up in Mtub | SERPINB9 |
| 1392 | G1T030 | Cilia and flagella associated protein 44 OS=Oryctol | CFAP44 | RABBIT | -2.00853 | #N/A | #N/A | #N/A | #N/A | 0.38185 | #N/A | -0.06437 | #N/A | #N/A | 2.167278 | 5.60835 | 2.67565 | 0.11233 | 0.05648 | 0.20006 | 0.15773 | 1 | 2.93229 | 0.55262 | 0.09956 | 0.437401 | Not Significant | NS | #N/A |
| 2663 | A0A5F9C487 | #N/A | OAT | RABBIT | #N/A | #N/A | #N/A | -1.97721 | #N/A | 0.39429 | -0.68682 | 0.85436 | 0.72564 | -0.39429 | 2.155843 | 2.87096 | 2.76968 | 0.04542 | 0.02798 | 0.14686 | 0.11898 | 4 | 2.93229 | 0.55262 | 0.46989 | 0.504885 | Significant | Up in Mtub | OAT |
| 2080 | G1SHF9 | #N/A | UFC1 | RABBIT | #N/A | -0.97715 | #N/A | #N/A | #N/A | 1.17497 | #N/A | #N/A | #N/A | #N/A | 2.152127 | #N/A | 2.04711 | #N/A | 0.13524 | #N/A | 0.23421 | 0 | 2.93229 | 0.55262 | #N/A | 0.552617 | Not Significant | NS | #N/A |
| 1292 | G1SQR7 | Nuclear transport factor 2 OS=Oryctolagus cunicul | #N/A | RABBIT | -2.18146 | 0.15261 | -1.92999 | -1.25702 | -2.08194 | 1.46916 | 0.04521 | 0.30508 | 0.76114 | 0.86695 | 2.149069 | 4.30794 | 4.37401 | 0.00259 | 0.00113 | 0.05406 | 0.04239 | 8 | 2.93229 | 0.55262 | 0.62216 | 0.603506 | Significant | Up in Mtub | #N/A |
| 2064 | U3KPQ3 | #N/A | SENP2 | RABBIT | #N/A | -2.13724 | #N/A | #N/A | #N/A | 0 | #N/A | #N/A | 0.01582 | #N/A | 2.145148 | 156.549 | 2.72836 | 0.00407 | 0.05352 | 0.06755 | 0.15308 | 1 | 2.93229 | 0.55262 | 0.00013 | 0.412115 | Not Significant | NS | #N/A |
| 2206 | G1T466 | #N/A | UBA6 | RABBIT | #N/A | #N/A | -2.16989 | -2.13448 | #N/A | 0.65223 | -1.60751 | -0.06437 | 0.37578 | 0.59694 | 2.142798 | 3.05618 | 3.18417 | 0.02822 | 0.01306 | 0.12583 | 0.09653 | 5 | 2.93229 | 0.55262 | 0.70227 | 0.646951 | Significant | Up in Mtub | UBA6 |
| 2319 | A0A5F9DNU2 | #N/A | #N/A | RABBIT | #N/A | #N/A | #N/A | -1.0979 | #N/A | #N/A | #N/A | #N/A | #N/A | 1.04026 | 2.138155 | #N/A | 2.03382 | #N/A | 0.13695 | #N/A | 0.23575 | 0 | 2.93229 | 0.55262 | #N/A | 0.552617 | Not Significant | NS | #N/A |
| 2273 | A0A5F9CWY3 | #N/A | SMAP1 | RABBIT | #N/A | #N/A | -1.95813 | #N/A | #N/A | 0.689 | -0.36421 | -0.56023 | 0.76854 | 0.36421 | 2.13759 | 3.2042 | 2.91619 | 0.03277 | 0.02271 | 0.132 | 0.11217 | 4 | 2.93229 | 0.55262 | 0.37087 | 0.44775 | Significant | Up in Mtub | SMAP1 |
| 1606 | G1TKE3 | Transgelin OS=Oryctolagus cuniculus OX=9986 GN | TAGLN2 | RABBIT | #N/A | #N/A | #N/A | -2.26243 | #N/A | 0.12694 | -0.06032 | -1.2456 | 0.06032 | 0.43308 | 2.125311 | 3.00403 | 2.81673 | 0.03978 | 0.02616 | 0.14119 | 0.117 | 4 | 2.93229 | 0.55262 | 0.41712 | 0.474431 | Significant | Up in Mtub | TAGLN2 |
| 1181 | G1TLQ8 | Proteasome 26S subunit, ATPase 3 OS=Oryctolagus | PSMC3 | RABBIT | -2.69854 | -1.87063 | -0.09308 | 0.61201 | -3.65456 | 0.66099 | 0.16754 | -0.17109 | 0.10672 | 2.14246 | 2.122283 | 2.36881 | 2.63913 | 0.04533 | 0.02314 | 0.14686 | 0.11217 | 8 | 2.93229 | 0.55262 | 2.00671 | 1.616689 | Significant | Up in Mtub | PSMC3 |
| 1272 | G1TGG4 | Spermatogenesis associated 2 OS=Oryctolagus cu | SPATA2 | RABBIT | -0.92662 | #N/A | #N/A | -1.62547 | #N/A | 1.24986 | #N/A | -0.06437 | 1.33225 | #N/A | 2.115292 | 3.3076 | 3.2092 | 0.04547 | 0.01868 | 0.14686 | 0.106 | 3 | 2.93229 | 0.55262 | 0.49079 | 0.52135 | Significant | Up in Mtub | SPATA2 |
| 1524 | G1TEK3 | GRIP and coiled-coil domain containing 2 OS=Oryct | GCC2 | RABBIT | #N/A | #N/A | -0.37513 | -1.85229 | -0.14699 | #N/A | 0 | 2.44655 | 1.54027 | 1.2796 | 2.108075 | 2.82421 | 3.07376 | 0.03693 | 0.01542 | 0.13648 | 0.09934 | 5 | 2.93229 | 0.55262 | 0.95513 | 0.806336 | Significant | Up in Mtub | GCC2 |
| 1880 | A0A5F9DCQ3 | #N/A | EFTUD2 | RABBIT | #N/A | #N/A | #N/A | -1.08027 | #N/A | 1.02263 | #N/A | #N/A | #N/A | #N/A | 2.102893 | #N/A | 2.00028 | #N/A | 0.1414 | #N/A | 0.24075 | 0 | 2.93229 | 0.55262 | #N/A | 0.552617 | Not Significant | NS | #N/A |
| 1354 | G1SY30 | U5 small nuclear ribonucleoprotein 200 kDa helica: | SNRNP200 | RABBIT | #N/A | -0.36755 | -0.06665 | -2.887 | #N/A | #N/A | 0.2373 | 2.04233 | #N/A | 0.69599 | 2.098941 | 2.00768 | 2.36675 | 0.1151 | 0.05019 | 0.20204 | 0.15072 | 4 | 2.93229 | 0.55262 | 1.63947 | 1.179743 | Not Significant | NS | #N/A |
| 1900 | A0A5F9CCT1 | #N/A | GAS2 | RABBIT | #N/A | -0.94609 | #N/A | #N/A | #N/A | #N/A | 1.14391 | #N/A | #N/A | #N/A | 2.090007 | #N/A | 1.98802 | #N/A | 0.14307 | #N/A | 0.24239 | 0 | 2.93229 | 0.55262 | #N/A | 0.552617 | Not Significant | NS | #N/A |
| 930 | A0A5F9DKZ2 | Erythrocyte membrane protein band 4.1 like 2 OS=( | EPB41L2 | RABBIT | -2.66612 | -0.08247 | -0.22428 | -1.85713 | -0.36653 | 2.43621 | 1.50326 | 0.45059 | 0.53915 | 0.2803 | 2.081209 | 3.15785 | 3.38893 | 0.01344 | 0.0061 | 0.09752 | 0.07277 | 8 | 2.93229 | 0.55262 | 1.0859 | 0.94286 | Significant | Up in Mtub | EPB41L2 |
| 1969 | A0A5F9DT66 | #N/A | LRRCC1 | RABBIT | #N/A | 0.19782 | #N/A | #N/A | -1.65374 | #N/A | 1.34849 | #N/A | #N/A | #N/A | 2.076452 | 1.29495 | 1.84111 | 0.41863 | 0.14065 | 0.3785 | 0.23971 | 1 | 2.93229 | 0.55262 | 1.71414 | 0.847998 | Not Significant | NS | #N/A |
| 1360 | G1U1X6 | 5'-nucleotidase OS=Oryctolagus cuniculus OX=998 | NT5C3A | RABBIT | -1.72058 | #N/A | #N/A | #N/A | #N/A | #N/A | #N/A | 0.21697 | 1.13116 | -0.28134 | 2.076183 | 2.50987 | 2.45447 | 0.12878 | 0.0583 | 0.21348 | 0.16044 | 2 | 2.93229 | 0.55262 | 0.5132 | 0.536635 | Not Significant | NS | #N/A |
| 1610 | G1SZ38 | Ring finger protein 103 OS=Oryctolagus cuniculus ( | RNF103 | RABBIT | -2.76585 | -0.96313 | #N/A | #N/A | #N/A | #N/A | 0.33575 | -0.06437 | 0.36319 | #N/A | 2.076011 | 2.98658 | 3.02182 | 0.05829 | 0.02367 | 0.15468 | 0.11338 | 3 | 2.93229 | 0.55262 | 0.57982 | 0.566374 | Significant | Up in Mtub | RNF103 |
| 1119 | G1SL45 | Fucose kinase OS=Oryctolagus cuniculus OX=9986 | FCSK | RABBIT | -1.36921 | #N/A | -1.73482 | -0.21371 | 0.14269 | #N/A | 1.85916 | -1.82593 | 3.00168 | 2.06755 | 2.069379 | 1.79232 | 2.08386 | 0.12326 | 0.06708 | 0.2103 | 0.1709 | 6 | 2.93229 | 0.55262 | 2.66611 | 1.972296 | Not Significant | NS | #N/A |
| 707 | Q01971 | Ras-related protein Rab-2A OS=Oryctolagus cunicu | RAB2A RAI | RABBIT | -4.33777 | -0.636 | -1.89664 | 0.04297 | -2.56542 | -0.05797 | 0.51552 | 0.11529 | 0.30821 | 0.05797 | 2.066375 | 2.67208 | 2.93137 | 0.02827 | 0.01374 | 0.12583 | 0.09774 | 8 | 2.93229 | 0.55262 | 1.49506 | 1.242277 | Significant | Up in Mtub | RAB2A RAB2 |
| 558 | G1T5V3 | Ras-related protein Rab-14 OS=Oryctolagus cunicu | RAB14 | RABBIT | -3.34277 | -3.11091 | -0.68733 | -1.07701 | 0.80955 | 0.68614 | 0.80962 | -0.02271 | 1.44667 | -0.04165 | 2.057308 | 2.48326 | 2.74539 | 0.03792 | 0.01914 | 0.13861 | 0.10762 | 8 | 2.93229 | 0.55262 | 1.71591 | 1.403885 | Significant | Up in Mtub | RAB14 |

| | | | | | | | | | | | | | | | | | | | | | | | | | | | | | | |
|---|---|---|---|---|---|---|---|---|---|---|---|---|---|---|---|---|---|---|---|---|---|---|---|---|---|---|---|---|---|---|
| 2554 | A0A5F9DSF0 | #N/A | VPS35L | RABBIT | #N/A | #N/A | #N/A | -2.23763 | #N/A | 0.79548 | 0.20221 | -2.14044 | -0.20221 | 0.43569 | 2.055772 | 1.62719 | 1.87548 | 0.17903 | 0.10326 | 0.25448 | 0.20233 | 4 | 2.93229 | 0.55262 | 1.33013 | 1.001251 | Not Significant | NS | #N/A |
| 966 | G1T550 | small monomeric GTPase OS=Oryctolagus cuniculu | RAB5C | RABBIT | #N/A | #N/A | -1.97208 | -0.49708 | -1.00546 | 2.97634 | 0.34989 | -0.41425 | 0.97618 | 0.59283 | 2.054405 | 2.50546 | 2.77423 | 0.04619 | 0.02175 | 0.1481 | 0.11035 | 6 | 2.93229 | 0.55262 | 1.26067 | 1.028228 | Significant | Up in Mtub | RAB5C |
| 2198 | G1SJ66 | #N/A | FARP1 | RABBIT | #N/A | #N/A | -1.53589 | #N/A | #N/A | 1.74416 | #N/A | -0.06437 | -0.44488 | 0.82319 | 2.050418 | 1.87736 | 2.1096 | 0.1571 | 0.07994 | 0.23659 | 0.18325 | 3 | 2.93229 | 0.55262 | 0.95429 | 0.755745 | Not Significant | NS | #N/A |
| 1381 | G1SYV0 | 26S proteasome regulatory subunit 7 OS=Oryctolag | PSMC2 | RABBIT | -4.32375 | -2.87821 | -0.09111 | 0.85215 | -1.40621 | 1.12599 | -0.54306 | 0.6792 | 0.26176 | 0.87246 | 2.048697 | 2.09748 | 2.354 | 0.06921 | 0.03835 | 0.16349 | 0.12996 | 8 | 2.93229 | 0.55262 | 2.38507 | 1.893568 | Significant | Up in Mtub | PSMC2 |
| 2147 | A0A5F9DDP5 | #N/A | USP14 | RABBIT | #N/A | #N/A | -1.59661 | #N/A | #N/A | 0.89143 | #N/A | #N/A | #N/A | 0 | 2.042323 | 2.64549 | 2.32792 | 0.23007 | 0.08157 | 0.2895 | 0.18476 | 1 | 2.93229 | 0.55262 | 0.39732 | 0.513125 | Not Significant | NS | #N/A |
| 2415 | A0A5F9CH05 | #N/A | VPS25 | RABBIT | #N/A | #N/A | #N/A | -1.93297 | #N/A | 1.1462 | -1.49091 | -0.7408 | 0.94738 | 0.67643 | 2.040633 | 1.60587 | 1.85341 | 0.18357 | 0.10665 | 0.25793 | 0.20568 | 4 | 2.93229 | 0.55262 | 1.34563 | 1.010194 | Not Significant | NS | #N/A |
| 546 | G1TCS8 | Ras-related protein Rab-1A OS=Oryctolagus cunicu | RAB1A | RABBIT | -2.87116 | -1.96269 | -0.87773 | -0.22329 | -1.70721 | 1.19502 | 0.16565 | 0.12005 | 0.44238 | 0.62675 | 2.038388 | 4.11465 | 4.17057 | 0.00337 | 0.00158 | 0.06482 | 0.04595 | 8 | 2.93229 | 0.55262 | 0.61355 | 0.597205 | Significant | Up in Mtub | RAB1A |
| 256 | A0A5F9DP68 | Proteasome 20S subunit alpha 6 OS=Oryctolagus c | PSMA6 | RABBIT | 0.07157 | -1.22234 | -0.0154 | -1.40314 | -0.01338 | #N/A | 3.33361 | 2.3106 | 1.73613 | -1.31496 | 2.032884 | 2.1328 | 2.40632 | 0.07038 | 0.03707 | 0.16349 | 0.12876 | 7 | 2.93229 | 0.55262 | 2.01889 | 1.586009 | Significant | Up in Mtub | PSMA6 |
| 2354 | A0A5F9CMS3 | #N/A | RRAS2 | RABBIT | #N/A | #N/A | -0.83155 | -0.8913 | -1.47369 | 2.23425 | 0.41358 | 2.14061 | 0.44679 | -0.41358 | 2.029844 | 2.84723 | 3.06653 | 0.02929 | 0.01355 | 0.12708 | 0.09774 | 6 | 2.93229 | 0.55262 | 0.95297 | 0.821545 | Significant | Up in Mtub | RRAS2 |
| 2543 | G1SRA9 | #N/A | ILK | RABBIT | #N/A | #N/A | #N/A | -1.9447 | #N/A | 1.03593 | -0.86731 | -0.18556 | 0.26765 | 0.12119 | 2.019082 | 2.66144 | 2.57969 | 0.05631 | 0.03679 | 0.15432 | 0.12876 | 4 | 2.93229 | 0.55262 | 0.47962 | 0.510494 | Significant | Up in Mtub | ILK |
| 1289 | G1SLK2 | 26S proteasome regulatory subunit 8 OS=Oryctolag | #N/A | RABBIT | -3.62646 | -1.34938 | 0.55004 | -0.12419 | -3.01346 | 0.06655 | 0.43254 | 0.95069 | -0.12161 | 1.13585 | 2.005494 | 2.38717 | 2.643 | 0.04405 | 0.02298 | 0.14581 | 0.11217 | 8 | 2.93229 | 0.55262 | 1.76448 | 1.439428 | Significant | Up in Mtub | #N/A |
| 1424 | A0A5F9C2S6 | Phosphatidylinositol 3-kinase regulatory subunit alp | PIK3R1 | RABBIT | #N/A | #N/A | #N/A | -0.90553 | -1.37067 | 0.69076 | -1.11549 | -0.06437 | 0.89032 | 3.90317 | 1.998975 | 1.41997 | 1.69418 | 0.21485 | 0.129 | 0.27765 | 0.22709 | 5 | 2.93229 | 0.55262 | 2.83112 | 1.98884 | Not Significant | NS | #N/A |
| 1480 | G1TX74 | Twinfilin actin binding protein 2 OS=Oryctolagus cu | TWF2 | RABBIT | #N/A | -2.25568 | #N/A | -0.11811 | -2.11012 | 0.06047 | 1.55632 | -0.19529 | 0.57645 | 0.51595 | 1.997417 | 3.10639 | 3.26404 | 0.02094 | 0.00987 | 0.11689 | 0.09102 | 6 | 2.93229 | 0.55262 | 0.77522 | 0.702146 | Significant | Up in Mtub | TWF2 |
| 1143 | G1TC28 | Kinesin family member 21A OS=Oryctolagus cunicu | KIF21A | RABBIT | -0.00079 | -2.34483 | #N/A | -1.35819 | -0.06072 | 1.26837 | 0 | 2.01749 | 0.48117 | 1.4944 | 1.993419 | 3.10514 | 3.30463 | 0.0172 | 0.00802 | 0.10978 | 0.08337 | 7 | 2.93229 | 0.55262 | 0.91585 | 0.808611 | Significant | Up in Mtub | KIF21A |
| 2705 | G1SJ57 | #N/A | SBDS | RABBIT | #N/A | #N/A | #N/A | -1.76267 | #N/A | 0.67021 | #N/A | #N/A | -0.50077 | 0.50077 | 1.986075 | 2.71784 | 2.45479 | 0.11291 | 0.05827 | 0.20012 | 0.16044 | 2 | 2.93229 | 0.55262 | 0.4005 | 0.490935 | Not Significant | NS | #N/A |
| 1562 | A0A5F9CH75 | EMAP like 4 OS=Oryctolagus cuniculus OX=9986 GI | EML4 | RABBIT | -2.20734 | -0.62048 | #N/A | -0.75139 | #N/A | 1.45283 | 1.50504 | -0.60984 | 1.05335 | 0.54548 | 1.982442 | 3.10329 | 3.25526 | 0.02103 | 0.01001 | 0.11691 | 0.09132 | 6 | 2.93229 | 0.55262 | 0.76517 | 0.695392 | Significant | Up in Mtub | EML4 |
| 2286 | A0A5F9DHI7 | #N/A | OGT | RABBIT | #N/A | #N/A | -2.74036 | 0.01551 | -1.86977 | 0.84796 | -0.64219 | 0.57754 | -0.07314 | 1.52045 | 1.977665 | 2.55202 | 2.79644 | 0.04337 | 0.02098 | 0.14581 | 0.10939 | 6 | 2.93229 | 0.55262 | 1.126 | 0.937768 | Significant | Up in Mtub | OGT |
| 1011 | G1T2I5 | Reticulon OS=Oryctolagus cuniculus OX=9986 GN= | RTN4 | RABBIT | -5.26656 | -0.48032 | 0.00339 | -0.8237 | -0.75279 | 0.16726 | 0.68466 | 1.01699 | 0.35396 | 0.31243 | 1.971054 | 2.02415 | 2.27117 | 0.07756 | 0.04436 | 0.17105 | 0.14118 | 8 | 2.93229 | 0.55262 | 2.37056 | 1.882948 | Significant | Up in Mtub | RTN4 |
| 1905 | G1TXF8 | #N/A | ADSS2 | RABBIT | #N/A | -3.60032 | -2.94206 | 1.50842 | #N/A | 0.1827 | -1.28924 | -0.24706 | 0.90042 | 1.89848 | 1.967047 | 1.43292 | 1.68513 | 0.20186 | 0.1265 | 0.2713 | 0.2257 | 6 | 2.93229 | 0.55262 | 3.53337 | 2.554847 | Not Significant | NS | #N/A |
| 1773 | G1T336 | #N/A | DDX3X | RABBIT | #N/A | -1.75669 | -0.79842 | 0.61047 | -2.17746 | 1.61274 | -0.33439 | 0.18819 | 0 | 3.15375 | 1.954585 | 2.13691 | 2.40038 | 0.06995 | 0.03745 | 0.16349 | 0.12876 | 7 | 2.93229 | 0.55262 | 1.8592 | 1.473457 | Significant | Up in Mtub | DDX3X |
| 1380 | A0A5F9CZR3 | Tubulin-specific chaperone E OS=Oryctolagus cuni | TBCE | RABBIT | -3.20225 | -1.36407 | 0.36021 | -1.11523 | -0.13822 | 1.98626 | 0.12483 | -0.35228 | 0.60923 | 1.94382 | 1.954284 | 2.51643 | 2.76201 | 0.03601 | 0.01859 | 0.13507 | 0.106 | 8 | 2.93229 | 0.55262 | 1.5078 | 1.251602 | Significant | Up in Mtub | TBCE |
| 2010 | G1SKT4 | #N/A | #N/A | RABBIT | #N/A | #N/A | -1.68788 | -0.98345 | #N/A | 1.2886 | #N/A | 1.16573 | -0.61548 | 0.61548 | 1.949248 | 2.83618 | 2.91268 | 0.04705 | 0.02282 | 0.14885 | 0.11217 | 4 | 2.93229 | 0.55262 | 0.6298 | 0.597154 | Significant | Up in Mtub | #N/A |
| 2026 | G1TDN4 | #N/A | PRKAR1A | RABBIT | #N/A | #N/A | -0.97842 | 0.08322 | -3.79651 | 0.87988 | -0.44293 | -0.20522 | 0.45947 | 1.19952 | 1.942048 | 2.06111 | 2.3327 | 0.08493 | 0.04476 | 0.17453 | 0.1421 | 6 | 2.93229 | 0.55262 | 1.66464 | 1.299583 | Significant | Up in Mtub | PRKAR1A |
| 2355 | A0A5F9CHR8 | #N/A | ATG7 | RABBIT | #N/A | #N/A | -1.61462 | #N/A | #N/A | 0.91711 | 0.01687 | -0.23605 | -0.01687 | 0.93967 | 1.938769 | 3.15968 | 2.74803 | 0.03419 | 0.02886 | 0.13313 | 0.12009 | 4 | 2.93229 | 0.55262 | 0.31375 | 0.414788 | Significant | Up in Mtub | ATG7 |
| 896 | T2HVY9 | 3alpha/17beta/20alpha-hydroxysteroid dehydroger | AKR1C32 | RABBIT | #N/A | 0.16353 | -0.03965 | -0.75019 | -1.0905 | 2.15725 | 1.13326 | -0.06437 | 2.06593 | 2.24392 | 1.936402 | 3.43946 | 3.55435 | 0.01085 | 0.00529 | 0.09732 | 0.06762 | 7 | 2.93229 | 0.55262 | 0.70437 | 0.659566 | Significant | Up in Mtub | AKR1C32 |
| 1946 | Q9N0V3 | #N/A | ABCB11 B! | RABBIT | #N/A | 0.88252 | #N/A | -0.05764 | -0.80389 | #N/A | -0.37661 | 4.25307 | #N/A | #N/A | 1.931236 | 1.05144 | 1.3888 | 0.37027 | 0.21479 | 0.35549 | 0.29234 | 3 | 2.93229 | 0.55262 | 4.04841 | 2.320463 | Not Significant | NS | #N/A |
| 970 | A0A5F9DF76 | Phosphofurin acidic cluster sorting protein 1 OS=Or | PACS1 | RABBIT | -3.02719 | -2.55632 | -1.82908 | 0.65794 | -0.82448 | 0.60658 | -0.00465 | -0.05972 | 0.77782 | 0.73483 | 1.926798 | 2.81951 | 3.04259 | 0.02251 | 0.01127 | 0.11776 | 0.09305 | 8 | 2.93229 | 0.55262 | 1.16753 | 1.002593 | Significant | Up in Mtub | PACS1 |
| 2744 | G1SR33 | #N/A | COMMD5 | RABBIT | #N/A | #N/A | #N/A | -1.77856 | #N/A | -0.08778 | 0.04588 | 0.20485 | -0.04588 | 0.62318 | 1.926612 | 6.09883 | 3.31347 | 0.00366 | 0.01306 | 0.06691 | 0.09653 | 4 | 2.93229 | 0.55262 | 0.08316 | 0.281735 | Significant | Up in Mtub | COMMD5 |
| 1064 | G1SGB5 | Splicing factor proline and glutamine rich OS=Oryct | SFPQ | RABBIT | -4.29525 | -0.77775 | 0.03305 | 0.88836 | -1.17834 | 0.99905 | 0.1376 | 1.49494 | -0.14414 | 1.80066 | 1.923607 | 2.00612 | 2.24816 | 0.07976 | 0.04618 | 0.17277 | 0.14461 | 8 | 2.93229 | 0.55262 | 2.29858 | 1.830277 | Significant | Up in Mtub | SFPQ |
| 1032 | A0A5F9D9X0 | Uroporphyrinogen decarboxylase OS=Oryctolagus c | UROD | RABBIT | -1.16723 | #N/A | #N/A | -1.37856 | #N/A | #N/A | 1.17889 | 1.81732 | -2.22846 | 1.83128 | 1.92265 | 1.31821 | 1.62332 | 0.25785 | 0.14897 | 0.30277 | 0.24821 | 4 | 2.93229 | 0.55262 | 2.83641 | 1.87039 | Not Significant | NS | #N/A |
| 39 | A0A5F9CMV1 | Tubulin beta chain OS=Oryctolagus cuniculus OX=9 | #N/A | RABBIT | -2.05271 | -6.96347 | 0.06234 | 0.39729 | -0.61467 | 0.71964 | -0.92222 | 0.04395 | 0.22688 | 0.37364 | 1.922622 | 1.39586 | 1.59798 | 0.20028 | 0.13853 | 0.27115 | 0.23727 | 8 | 2.93229 | 0.55262 | 4.74289 | 3.618961 | Not Significant | NS | #N/A |
| 1464 | G1SWG3 | Histamine N-methyltransferase OS=Oryctolagus cu | HNMT | RABBIT | #N/A | -1.95206 | #N/A | -1.95066 | #N/A | 0.63112 | 0.9068 | -2.58588 | 0.89698 | 0 | 1.921164 | 1.74003 | 2.01238 | 0.14234 | 0.07929 | 0.22477 | 0.18311 | 5 | 2.93229 | 0.55262 | 1.74148 | 1.301998 | Not Significant | NS | #N/A |
| 1780 | G1SCY3 | #N/A | UBR4 | RABBIT | #N/A | #N/A | -2.08777 | -0.04444 | -4.36579 | -0.0132 | -2.45229 | 0.52086 | 0.10681 | 0.57968 | 1.91437 | 1.62246 | 1.88453 | 0.15583 | 0.09239 | 0.23612 | 0.19439 | 6 | 2.93229 | 0.55262 | 2.61038 | 1.934855 | Not Significant | NS | #N/A |
| 1628 | A0AAG1VTQ6 | Receptor for activated C kinase 1 OS=Oryctolagus c | RACK1 | RABBIT | #N/A | -3.0886 | -0.02348 | 0.31131 | -4.35349 | 0.27086 | 0.73105 | -0.05238 | 0 | -0.33146 | 1.912178 | 1.86015 | 2.11401 | 0.10519 | 0.06082 | 0.19468 | 0.16242 | 7 | 2.93229 | 0.55262 | 2.34828 | 1.818147 | Not Significant | NS | #N/A |
| 641 | U3KPH8 | Huntingtin OS=Oryctolagus cuniculus OX=9986 GN | HTT | RABBIT | -1.63337 | -3.16731 | -0.46805 | -0.15752 | 0.0865 | 0.10871 | -0.50321 | 1.35992 | 1.87526 | 1.37071 | 1.910228 | 2.55137 | 2.78785 | 0.0341 | 0.01775 | 0.13313 | 0.10373 | 8 | 2.93229 | 0.55262 | 1.4014 | 1.17374 | Significant | Up in Mtub | HTT |
| 1875 | G1U276 | #N/A | SUCLA2 | RABBIT | #N/A | -3.44986 | -1.41406 | 0.18463 | -4.35914 | 0 | -3.87489 | 1.21805 | 0.46245 | 0.40399 | 1.901527 | 1.39875 | 1.62109 | 0.2046 | 0.13627 | 0.27183 | 0.23506 | 7 | 2.93229 | 0.55262 | 4.10691 | 3.057583 | Not Significant | NS | #N/A |
| 977 | G1T2I4 | proline--tRNA ligase OS=Oryctolagus cuniculus OX= | EPRS1 | RABBIT | -4.01403 | -2.40745 | -0.61993 | 0.41129 | -1.08496 | 0.59311 | -0.06052 | -0.00385 | 0.37837 | 0.86738 | 1.897916 | 2.41468 | 2.65406 | 0.0422 | 0.02253 | 0.14488 | 0.11217 | 8 | 2.93229 | 0.55262 | 1.54445 | 1.278417 | Significant | Up in Mtub | EPRS1 |
| 999 | G1SDA2 | Cellular retinoic acid-binding protein 1 OS=Oryctola | CRABP1 | RABBIT | -1.60954 | -1.64368 | -0.81612 | -0.351 | -0.56213 | 1.5537 | 1.50479 | 0.24278 | 0.91227 | 0.29336 | 1.897873 | 4.8794 | 4.60304 | 0.00123 | 0.00077 | 0.04885 | 0.0357 | 8 | 2.93229 | 0.55262 | 0.37822 | 0.424995 | Significant | Up in Mtub | CRABP1 |
| 1798 | G1SZR5 | #N/A | MAPK10 | RABBIT | #N/A | -2.95686 | -0.34575 | -1.51548 | #N/A | 0.45333 | #N/A | -0.06437 | 0.70868 | 0.06043 | 1.895548 | 2.84633 | 3.00192 | 0.03598 | 0.01719 | 0.13507 | 0.10198 | 5 | 2.93229 | 0.55262 | 0.7603 | 0.683525 | Significant | Up in Mtub | MAPK10 |
| 1005 | G1U115 | 26S proteasome non-ATPase regulatory subunit 6 C | PSMD6 | RABBIT | -4.06382 | -1.93644 | -0.36658 | 0.19234 | -1.76863 | 0.11383 | 0.09401 | 0.59293 | -0.09401 | 0.81502 | 1.892981 | 2.4933 | 2.72914 | 0.03733 | 0.01971 | 0.13749 | 0.10842 | 8 | 2.93229 | 0.55262 | 1.44106 | 1.202761 | Significant | Up in Mtub | PSMD6 |
| 1528 | A0A5F9DNQ1 | Calponin-homology (CH) domain-containing protei | #N/A | RABBIT | #N/A | #N/A | #N/A | -0.67257 | #N/A | #N/A | 2.80092 | -0.40574 | 2.4796 | 0 | 1.891267 | 1.0221 | 1.31359 | 0.38196 | 0.2375 | 0.36126 | 0.30755 | 3 | 2.93229 | 0.55262 | 2.73911 | 1.658342 | Not Significant | NS | #N/A |
| 1763 | G1THZ6 | #N/A | IGHM | RABBIT | #N/A | -1.7279 | #N/A | #N/A | -2.01198 | -4.25364 | 2.21425 | 1.92043 | 0 | 0.15103 | 1.876352 | 0.96964 | 1.18591 | 0.37676 | 0.26996 | 0.35889 | 0.32977 | 5 | 2.93229 | 0.55262 | 5.34948 | 3.576252 | Not Significant | NS | #N/A |
| 1787 | A0A5F9CLC5 | #N/A | ROCK1 | RABBIT | #N/A | -0.83885 | #N/A | #N/A | #N/A | #N/A | 1.03667 | #N/A | #N/A | #N/A | 1.875528 | #N/A | 1.78401 | #N/A | 0.17454 | #N/A | 0.26652 | 0 | 2.93229 | 0.55262 | #N/A | 0.552617 | Not Significant | NS | #N/A |
| 2853 | A0A5F9CUA6 | #N/A | SYK | RABBIT | #N/A | #N/A | #N/A | #N/A | -0.93915 | 0.92577 | #N/A | #N/A | #N/A | #N/A | 1.864925 | #N/A | 1.77392 | #N/A | 0.17629 | #N/A | 0.26749 | 0 | 2.93229 | 0.55262 | #N/A | 0.552617 | Not Significant | NS | #N/A |
| 994 | G1TBU9 | Acetyl-CoA acyltransferase 2 OS=Oryctolagus cuni | ACAA2 | RABBIT | -1.21089 | -1.90509 | 0.75928 | -0.64626 | -2.87389 | 0.69639 | -1.36511 | 1.34185 | 1.60495 | 1.16335 | 1.863656 | 2.29826 | 2.535 | 0.05061 | 0.02784 | 0.15026 | 0.11898 | 8 | 2.93229 | 0.55262 | 1.64389 | 1.351188 | Significant | Up in Mtub | ACAA2 |
| 1191 | Q95MF9 | Chloride intracellular channel protein 1 OS=Oryctol | CLIC1 | RABBIT | #N/A | -2.24183 | -0.82619 | -1.96517 | -0.01338 | 0.76421 | 0.74566 | -0.08354 | 0.4207 | 1.15083 | 1.861214 | 3.64563 | 3.67059 | 0.00823 | 0.00436 | 0.08715 | 0.06392 | 7 | 2.93229 | 0.55262 | 0.57921 | 0.571358 | Significant | Up in Mtub | CLIC1 |
| 1450 | G1T658 | Uveal autoantigen with coiled-coil domains and ank | UACA | RABBIT | -0.99559 | -0.75247 | #N/A | #N/A | #N/A | #N/A | #N/A | 0.88593 | #N/A | 1.08468 | 1.859333 | 11.8422 | 3.19564 | 0.00706 | 0.02458 | 0.07875 | 0.11483 | 2 | 2.93229 | 0.55262 | 0.02465 | 0.338532 | Significant | Up in Mtub | UACA |
| 1869 | A0A5F9DFY5 | #N/A | PPP1R21 | RABBIT | #N/A | -0.99665 | 0.46675 | #N/A | -0.30948 | #N/A | #N/A | 1.57294 | #N/A | #N/A | 1.852735 | 2.19151 | 2.17165 | 0.15976 | 0.08272 | 0.23886 | 0.18564 | 2 | 2.93229 | 0.55262 | 0.53604 | 0.545896 | Not Significant | NS | #N/A |
| 960 | A0A5F9C1C5 | A2ML1 protein OS=Oryctolagus cuniculus OX=9986 | #N/A | RABBIT | -2.06746 | #N/A | 2.10954 | #N/A | -1.27915 | 0 | #N/A | #N/A | #N/A | 2.87908 | 1.851896 | 0.93924 | 1.25037 | 0.4169 | 0.25822 | 0.37794 | 0.32364 | 3 | 2.93229 | 0.55262 | 4.66508 | 2.632318 | Not Significant | NS | #N/A |
| 1965 | A0A5F9CLD9 | #N/A | DYNC1LI2 | RABBIT | #N/A | -1.61569 | #N/A | -0.05764 | #N/A | #N/A | #N/A | #N/A | #N/A | 1.01024 | 1.846905 | 1.36877 | 1.77626 | 0.40168 | 0.15157 | 0.3719 | 0.25099 | 1 | 2.93229 | 0.55262 | 1.21377 | 0.720751 | Not Significant | NS | #N/A |
| 1777 | A0A5F9D5D7 | #N/A | HADH | RABBIT | #N/A | -4.27428 | -0.52356 | -1.43579 | -0.01338 | 0.63314 | -0.48632 | 0.5124 | 0.49725 | 0.24042 | 1.84113 | 2.12622 | 2.37321 | 0.07107 | 0.03922 | 0.16442 | 0.13135 | 7 | 2.93229 | 0.55262 | 1.66625 | 1.337472 | Significant | Up in Mtub | HADH |
| 2458 | A0A5F9DF32 | #N/A | ALOX15B | RABBIT | #N/A | #N/A | -0.90565 | #N/A | #N/A | 1.87001 | #N/A | #N/A | #N/A | 0 | 1.840656 | 1.13658 | 1.6237 | 0.45936 | 0.18098 | 0.39628 | 0.27126 | 1 | 2.93229 | 0.55262 | 1.74847 | 0.856727 | Not Significant | NS | #N/A |
| 1543 | A0A5F9D5N1 | adenine phosphoribosyltransferase OS=Oryctolagu | #N/A | RABBIT | -3.49301 | -1.84507 | -0.23551 | 0.30066 | -0.99358 | 1.07779 | -0.32171 | 0.25735 | 0.87141 | 1.01948 | 1.834162 | 2.55242 | 2.77405 | 0.03405 | 0.01819 | 0.13313 | 0.10478 | 8 | 2.93229 | 0.55262 | 1.29096 | 1.092918 | Significant | Up in Mtub | #N/A |
| 1112 | G1T752 | Malignant T-cell-amplified sequence OS=Oryctolag | #N/A | RABBIT | -4.65823 | -0.81337 | -0.36735 | 0.38516 | -0.6388 | 1.42148 | -0.4428 | 0.43065 | 1.17794 | 0.47139 | 1.830252 | 1.94176 | 2.17298 | 0.08811 | 0.05265 | 0.17722 | 0.15178 | 8 | 2.93229 | 0.55262 | 2.22111 | 1.773584 | Not Significant | NS | #N/A |
| 2446 | G1T448 | #N/A | RGS14 | RABBIT | #N/A | #N/A | 0.14256 | -0.05764 | -0.48353 | 1.973 | -0.64096 | 4.15629 | 1.24855 | #N/A | 1.817092 | 1.53603 | 1.81593 | 0.18513 | 0.10724 | 0.25954 | 0.20635 | 5 | 2.93229 | 0.55262 | 2.39905 | 1.716489 | Not Significant | NS | #N/A |
| 2045 | A0A5F9C0Z0 | #N/A | SNX27 | RABBIT | #N/A | -1.78852 | -2.40442 | 0.46738 | -1.1113 | 1.63881 | -0.24099 | -0.06437 | 1.52289 | 0.15254 | 1.81099 | 2.54699 | 2.76261 | 0.03827 | 0.02016 | 0.1391 | 0.10938 | 7 | 2.93229 | 0.55262 | 1.12348 | 0.954947 | Significant | Up in Mtub | SNX27 |
| 2888 | G1TCE1 | #N/A | #N/A | RABBIT | #N/A | #N/A | #N/A | #N/A | -1.41254 | 0.99204 | -0.66512 | #N/A | 1.25456 | 0 | 1.807909 | 1.81733 | 1.97034 | 0.16677 | 0.09685 | 0.24583 | 0.19688 | 3 | 2.93229 | 0.55262 | 0.79173 | 0.673539 | Not Significant | NS | #N/A |
| 1326 | G1SQD4 | TBC1 domain family member 24 OS=Oryctolagus cu | TBC1D24 | RABBIT | -1.38909 | #N/A | -1.29565 | -0.46702 | -2.39891 | 0.85046 | 0.29807 | 0.74804 | 0 | 0.19106 | 1.805195 | 4.58129 | 4.22168 | 0.00254 | 0.00179 | 0.05406 | 0.04684 | 7 | 2.93229 | 0.55262 | 0.34503 | 0.406318 | Significant | Up in Mtub | TBC1D24 |
| 688 | A0A5F9CKD8 | Ethylmalonyl-CoA decarboxylase OS=Oryctolagus c | ECHDC1 | RABBIT | -3.03081 | -1.69328 | 0.18585 | -0.69221 | -0.02858 | 1.2324 | -0.32763 | 0.40113 | 0.71819 | 1.73643 | 1.803912 | 2.61385 | 2.82473 | 0.03094 | 0.01662 | 0.12928 | 0.10198 | 8 | 2.93229 | 0.55262 | 1.19072 | 1.019568 | Significant | Up in Mtub | ECHDC1 |
| 1747 | A0A5F9DK06 | #N/A | MBP | RABBIT | #N/A | -1.45303 | -0.15381 | -2.5184 | -0.43093 | 1.06708 | #N/A | 0.8118 | 0.32446 | 0.45492 | 1.803604 | 3.19809 | 3.2693 | 0.01864 | 0.00979 | 0.11219 | 0.09075 | 6 | 2.93229 | 0.55262 | 0.63611 | 0.6087 | Significant | Up in Mtub | MBP |
| 1744 | G1SFU0 | #N/A | #N/A | RABBIT | #N/A | -2.47474 | -0.57506 | -1.40827 | -1.56783 | 0.66803 | 0 | -0.00245 | 0.26491 | 0.51161 | 1.794894 | 4.79381 | 4.32513 | 0.00198 | 0.00152 | 0.05406 | 0.04508 | 7 | 2.93229 | 0.55262 | 0.31153 | 0.382707 | Significant | Up in Mtub | #N/A |
| 2192 | G1SMG5 | #N/A | GSPT1 | RABBIT | #N/A | #N/A | -0.85237 | -0.96099 | -3.09417 | 0.09894 | #N/A | 0.10231 | 0 | 0.42231 | 1.79173 | 2.88878 | 2.97939 | 0.03424 | 0.01779 | 0.13313 | 0.10373 | 5 | 2.93229 | 0.55262 | 0.65947 | 0.619973 | Significant | Up in Mtub | GSPT1 |
| 1914 | A0A5F9CA59 | #N/A | #N/A | RABBIT | #N/A | 1.78877 | -1.17173 | #N/A | -2.14527 | #N/A | #N/A | 1.27801 | #N/A | #N/A | 1.787418 | 0.75549 | 1.08622 | 0.52881 | 0.32759 | 0.42603 | 0.36005 | 2 | 2.93229 | 0.55262 | 4.19816 | 2.030854 | Not Significant | NS | #N/A |
| 1268 | G1SFE0 | Proteasome 26S subunit, non-ATPase 14 OS=Oryct | PSMD14 | RABBIT | -4.02393 | -1.79653 | -0.19648 | -0.05764 | #N/A | 0.00852 | -0.17972 | 0.41766 | 0.27807 | 0.80766 | 1.785081 | 2.14058 | 2.37792 | 0.06958 | 0.03891 | 0.16349 | 0.13081 | 7 | 2.93229 | 0.55262 | 1.5454 | 1.252306 | Significant | Up in Mtub | PSMD14 |
| 763 | Q9XSC5 | Clusterin OS=Oryctolagus cuniculus OX=9986 GN= | CLU ApoJ | RABBIT | -1.3322 | -2.38227 | -0.40406 | 0.08371 | -0.63456 | 1.1636 | 1.36124 | 1.35208 | 0.45709 | -0.14135 | 1.772406 | 3.40767 | 3.49446 | 0.00925 | 0.00506 | 0.09144 | 0.06702 | 8 | 2.93229 | 0.55262 | 0.67632 | 0.64314 | Significant | Up in Mtub | CLU ApoJ |
| 975 | G1TRK3 | Morphine-6-dehydrogenase OS=Oryctolagus cunic | AKR1C26 | RABBIT | -2.30763 | -1.59765 | #N/A | 1.087 | -1.14421 | -1.44206 | 1.13082 | #N/A | 2.14373 | 1.28565 | 1.770156 | 1.6616 | 1.91655 | 0.14766 | 0.08778 | 0.2295 | 0.1893 | 6 | 2.93229 | 0.55262 | 2.26986 | 1.706126 | Not Significant | NS | #N/A |
| 1762 | G1T7I4 | #N/A | COPA | RABBIT | #N/A | -3.37191 | -1.30151 | -0.00474 | #N/A | 0.72634 | 0.03933 | -0.45078 | -0.03933 | 0.75759 | 1.766017 | 2.26107 | 2.48137 | 0.06445 | 0.0351 | 0.15893 | 0.12696 | 6 | 2.93229 | 0.55262 | 1.14383 | 0.94975 | Significant | Up in Mtub | COPA |
| 1311 | A0A5F9DTM2 | YTH domain-containing family protein OS=Oryctola | YTHDF3 | RABBIT | -1.35746 | #N/A | -0.68187 | -0.28488 | -0.97911 | 3.04511 | 0 | 0.84304 | 0.47644 | 0.32863 | 1.764474 | 2.72279 | 2.90318 | 0.02965 | 0.01585 | 0.12757 | 0.09934 | 7 | 2.93229 | 0.55262 | 0.93323 | 0.820863 | Significant | Up in Mtub | YTHDF3 |
| 2414 | G1SL95 | #N/A | PMM2 | RABBIT | #N/A | #N/A | #N/A | -0.17132 | -0.04741 | 2.58122 | #N/A | -0.06437 | 2.43896 | #N/A | 1.761303 | 1.58662 | 1.90948 | 0.21079 | 0.10534 | 0.27497 | 0.20407 | 3 | 2.93229 | 0.55262 | 1.47878 | 1.020985 | Not Significant | NS | #N/A |
| 862 | G1TMX4 | Hippocalcin-like protein 4 OS=Oryctolagus cuniculu | HPCAL4 | RABBIT | -3.04767 | -2.97964 | #N/A | 0.63602 | -0.10797 | 0 | -0.84169 | 0.91919 | 0.79105 | 1.03932 | 1.75639 | 1.88164 | 2.11831 | 0.10192 | 0.06038 | 0.19155 | 0.16242 | 7 | 2.93229 | 0.55262 | 1.93622 | 1.527743 | Not Significant | NS | #N/A |
| 1850 | A0A5F9DRQ6 | #N/A | COMT | RABBIT | #N/A | -1.68774 | #N/A | -0.74458 | #N/A | 0.83866 | 0 | -0.67204 | 1.10353 | 1.42957 | 1.756106 | 2.54404 | 2.63742 | 0.05164 | 0.03005 | 0.15041 | 0.12239 | 5 | 2.93229 | 0.55262 | 0.6807 | 0.633353 | Significant | Up in Mtub | COMT |
| 2054 | G1TT75 | #N/A | PGRMC1 | RABBIT | #N/A | -1.64542 | #N/A | #N/A | -1.53066 | -0.23666 | 0.33199 | 0.22824 | 0 | 0.50139 | 1.753031 | 8.03289 | 4.21448 | 0.00048 | 0.00299 | 0.03578 | 0.05579 | 5 | 2.93229 | 0.55262 | 0.06804 | 0.247168 | Significant | Up in Mtub | PGRMC1 |
| 2275 | G1TBW2 | #N/A | IDH3G | RABBIT | #N/A | #N/A | -1.51209 | #N/A | #N/A | -0.33206 | #N/A | 0.7175 | 0.33206 | #N/A | 1.751254 | 2.85665 | 2.27909 | 0.10381 | 0.07234 | 0.19361 | 0.17519 | 2 | 2.93229 | 0.55262 | 0.28187 | 0.442831 | Not Significant | NS | #N/A |
| 2522 | G1SM72 | #N/A | KIN | RABBIT | #N/A | #N/A | #N/A | -0.79992 | #N/A | 1.96492 | #N/A | -0.06437 | #N/A | #N/A | 1.750192 | 0.99589 | 1.47731 | 0.50131 | 0.21484 | 0.41436 | 0.29234 | 1 | 2.93229 | 0.55262 | 2.05899 | 0.935695 | Not Significant | NS | #N/A |
| 263 | P68105 | Elongation factor 1-alpha 1 OS=Oryctolagus cunicu | EEF1A1 EE | RABBIT | -2.78041 | -1.42146 | -0.17408 | -0.17746 | -1.11183 | 1.00692 | 1.2945 | 0.15656 | 0.11982 | 0.50609 | 1.749826 | 3.27406 | 3.37811 | 0.01129 | 0.00621 | 0.09732 | 0.07298 | 8 | 2.93229 | 0.55262 | 0.71409 | 0.670782 | Significant | Up in Mtub | EEF1A1 EEF1A EF1A |
| 2714 | A0A5F9C153 | #N/A | ATP5PO | RABBIT | #N/A | #N/A | #N/A | -1.59869 | #N/A | -0.26331 | #N/A | 0.19895 | #N/A | 0.51273 | 1.74815 | 3.8781 | 2.42322 | 0.06052 | 0.06057 | 0.15579 | 0.16242 | 2 | 2.93229 | 0.55262 | 0.1524 | 0.390332 | Not Significant | NS | #N/A |
| 2679 | A0A5F9D1J2 | #N/A | MOB1A | RABBIT | #N/A | #N/A | #N/A | -0.89824 | #N/A | 0.8406 | #N/A | #N/A | #N/A | #N/A | 1.738844 | #N/A | 1.65399 | #N/A | 0.19879 | #N/A | 0.28306 | 0 | 2.93229 | 0.55262 | #N/A | 0.552617 | Not Significant | NS | #N/A |
| 1271 | G1TAJ8 | Synapsin ATP-binding domain-containing protein OS | #N/A | RABBIT | -4.00557 | -1.1932 | -0.20025 | 0.3248 | -3.22524 | 0.37361 | -1.82141 | 0.69779 | 0.76801 | 0.3709 | 1.737675 | 1.78842 | 2.00632 | 0.11151 | 0.0702 | 0.19884 | 0.17293 | 8 | 2.93229 | 0.55262 | 2.36013 | 1.875317 | Not Significant | NS | #N/A |
| 2508 | Q28695 | #N/A | #N/A | RABBIT | #N/A | #N/A | #N/A | -1.01515 | #N/A | #N/A | 0 | #N/A | 1.44301 | #N/A | 1.736657 | 1.38968 | 1.72354 | 0.39709 | 0.16112 | 0.36958 | 0.25578 | 1 | 2.93229 | 0.55262 | 1.04114 | 0.67685 | Not Significant | NS | #N/A |
| 2455 | P9WIJ1 | #N/A | #N/A | MT | #N/A | #N/A | -0.78242 | #N/A | #N/A | #N/A | #N/A | #N/A | #N/A | 0.95307 | 1.735491 | #N/A | 1.6508 | #N/A | 0.19944 | #N/A | 0.28306 | 0 | 2.93229 | 0.55262 | #N/A | 0.552617 | Not Significant | NS | #N/A |
| 1825 | A0A5F9DDI5 | #N/A | PPP6R3 | RABBIT | #N/A | -1.66625 | -1.096 | 0.36148 | -1.10651 | 0.07751 | -0.87849 | 3.69009 | 1.40363 | 0 | 1.735369 | 1.77096 | 2.00364 | 0.11987 | 0.07314 | 0.20742 | 0.17612 | 7 | 2.93229 | 0.55262 | 2.13379 | 1.666987 | Not Significant | NS | #N/A |
| 2251 | A0A5F9CL23 | #N/A | GALK2 | RABBIT | #N/A | #N/A | -1.06068 | -1.62489 | #N/A | #N/A | -0.16152 | 0.09715 | 0.6759 | 0.94366 | 1.731587 | 4.13035 | 3.29168 | 0.01449 | 0.01346 | 0.09905 | 0.09774 | 4 | 2.93229 | 0.55262 | 0.23434 | 0.36897 | Significant | Up in Mtub | GALK2 |
| 1124 | G1SZ12 | Large ribosomal subunit protein eL14 OS=Oryctolag | RPL14 | RABBIT | -4.25586 | -3.20667 | 0.65624 | -1.78665 | 0.71779 | -0.16636 | 0.16636 | 0.67134 | 0.29215 | -0.22758 | 1.722212 | 1.6943 | 1.90725 | 0.12866 | 0.08309 | 0.21348 | 0.18576 | 8 | 2.93229 | 0.55262 | 2.58304 | 2.038436 | Not Significant | NS | #N/A |
| 1141 | G1SSE2 | V-type proton ATPase proteolipid subunit OS=Oryct | #N/A | RABBIT | -2.87616 | -3.44402 | 0.08101 | -1.22598 | 0.47292 | 0.08964 | 0.28978 | 0.93777 | 0.4984 | -0.19877 | 1.72181 | 2.15016 | 2.36834 | 0.06376 | 0.03739 | 0.15847 | 0.12876 | 8 | 2.93229 | 0.55262 | 1.60313 | 1.32136 | Significant | Up in Mtub | #N/A |
| 1447 | A0A5F9D4Y7 | GCS light chain OS=Oryctolagus cuniculus OX=998 | GCLM | RABBIT | -0.39435 | #N/A | 0.17065 | #N/A | -0.25419 | #N/A | 1.11757 | #N/A | #N/A | 1.99504 | 1.715602 | 4.35723 | 3.10124 | 0.02234 | 0.0214 | 0.11776 | 0.11031 | 3 | 2.93229 | 0.55262 | 0.18603 | 0.367233 | Significant | Up in Mtub | GCLM |
| 1370 | G1TAD9 | DUF4592 domain-containing protein OS=Oryctolag | #N/A | RABBIT | -0.85164 | #N/A | #N/A | #N/A | #N/A | #N/A | #N/A | #N/A | #N/A | 0.8633 | 1.714939 | #N/A | 1.63125 | #N/A | 0.20342 | #N/A | 0.28553 | 0 | 2.93229 | 0.55262 | #N/A | 0.552617 | Not Significant | NS | #N/A |
| 2482 | B7NZP9 | #N/A | FLNA | RABBIT | #N/A | #N/A | #N/A | -1.2751 | -0.90089 | -0.14946 | 2.14508 | -0.06437 | 0.45638 | 0.72761 | 1.71104 | 2.44622 | 2.54674 | 0.05821 | 0.03458 | 0.15468 | 0.1262 | 5 | 2.93229 | 0.55262 | 0.69892 | 0.64484 | Significant | Up in Mtub | FLNA |
| 1285 | G1SQK3 | Bis(5'-nucleosyl)-tetraphosphatase [asymmetrical] | NUDT2 | RABBIT | -3.01176 | -2.54682 | -0.42571 | 0.76329 | -0.76717 | 1.15305 | -0.75123 | 0.53199 | 0.79082 | 0.84219 | 1.711 | 2.21522 | 2.42982 | 0.05761 | 0.03355 | 0.15468 | 0.1262 | 8 | 2.93229 | 0.55262 | 1.49145 | 1.239633 | Significant | Up in Mtub | NUDT2 |
| 2519 | G1SGY2 | #N/A | PTPN12 | RABBIT | #N/A | #N/A | #N/A | -1.61309 | #N/A | 0.28986 | #N/A | #N/A | -0.14511 | 0.14511 | 1.709714 | 6.68458 | 2.5084 | 0.02166 | 0.0546 | 0.11776 | 0.15447 | 2 | 2.93229 | 0.55262 | 0.04906 | 0.34843 | Not Significant | NS | #N/A |
| 1065 | G1TH33 | Electron transfer flavoprotein subunit beta OS=Oryc | ETFB | RABBIT | -2.62038 | -1.00943 | 0.20317 | -0.58396 | -0.0459 | 1.5519 | -0.5546 | 1.27659 | 1.27591 | 0.93946 | 1.709156 | 2.7361 | 2.91055 | 0.0256 | 0.01426 | 0.12149 | 0.09913 | 8 | 2.93229 | 0.55262 | 0.97553 | 0.862092 | Significant | Up in Mtub | ETFB |
| 1090 | G1SRP8 | Prostaglandin reductase 2 OS=Oryctolagus cunicul | PTGR2 | RABBIT | -3.63964 | -1.42703 | -1.13013 | -0.08168 | 0.01065 | 0.68491 | -0.14619 | 0.40717 | 1.07789 | 0.23905 | 1.706129 | 2.46755 | 2.66735 | 0.03886 | 0.022 | 0.1391 | 0.11062 | 8 | 2.93229 | 0.55262 | 1.19518 | 1.02283 | Significant | Up in Mtub | PTGR2 |

| | | | | | | | | | | | | | | | | | | | | | | | | | | | | | |
|---|---|---|---|---|---|---|---|---|---|---|---|---|---|---|---|---|---|---|---|---|---|---|---|---|---|---|---|---|---|
| 23 | A0A5F9D363 | Pyruvate kinase OS=Oryctolagus cuniculus OX=998 | #N/A | RABBIT | -1.9042 | -0.50863 | -0.53512 | -0.1813 | -2.09492 | 1.36544 | 0.12367 | 0.13009 | 0.98555 | 0.64787 | 1.695356 | 3.65376 | 3.64075 | 0.00646 | 0.00392 | 0.07556 | 0.06094 | 8 | 2.93229 | 0.55262 | 0.53825 | 0.542102 | Significant | Up in Mtub | #N/A |
| 2850 | G1T1I4 | #N/A | ZBTB48 | RABBIT | #N/A | #N/A | #N/A | #N/A | -0.85111 | #N/A | 0.83772 | #N/A | #N/A | #N/A | 1.688829 | #N/A | 1.60642 | #N/A | 0.20861 | #N/A | 0.28822 | 0 | 2.93229 | 0.55262 | #N/A | 0.552617 | Not Significant | NS | #N/A |
| 1347 | A0A5F9DCX9 | Sorting nexin-1 OS=Oryctolagus cuniculus OX=998 | SNX1 | RABBIT | #N/A | -0.92051 | -0.855 | -0.53993 | -1.6189 | 0 | 0.11624 | 1.12225 | 1.17597 | 1.10521 | 1.687519 | 4.68209 | 4.15479 | 0.00226 | 0.00199 | 0.05406 | 0.04954 | 7 | 2.93229 | 0.55262 | 0.28867 | 0.366596 | Significant | Up in Mtub | SNX1 |
| 1264 | G1SR17 | AP-3 complex subunit beta OS=Oryctolagus cunicu | AP3B2 | RABBIT | -1.59693 | -1.81536 | 0.04538 | 0.06763 | -0.27164 | 1.90513 | -0.24797 | 0.78253 | 1.03691 | 1.38449 | 1.686399 | 3.09309 | 3.20526 | 0.01482 | 0.00844 | 0.10079 | 0.08441 | 8 | 2.93229 | 0.55262 | 0.74315 | 0.692043 | Significant | Up in Mtub | AP3B2 |
| 2264 | G1TTN9 | #N/A | RCC2 | RABBIT | #N/A | #N/A | -1.04788 | -1.08207 | -1.40583 | 0.94504 | -0.31398 | 0.69547 | 0.31398 | 0.87426 | 1.681549 | 5.25598 | 4.13333 | 0.00191 | 0.00259 | 0.05406 | 0.05283 | 6 | 2.93229 | 0.55262 | 0.19192 | 0.310327 | Significant | Up in Mtub | RCC2 |
| 971 | G1SVE5 | WD repeat domain 82 OS=Oryctolagus cuniculus O | WDR82 | RABBIT | 0.53066 | -1.36767 | -1.76607 | -0.31559 | -3.76288 | 1.00655 | -1.22472 | 0.19358 | 0.98118 | 0.72825 | 1.673278 | 1.99446 | 2.20788 | 0.08121 | 0.04955 | 0.1731 | 0.14997 | 8 | 2.93229 | 0.55262 | 1.75965 | 1.435898 | Significant | Up in Mtub | WDR82 |
| 974 | G1T6D4 | Proteasome 26S subunit, non-ATPase 12 OS=Oryct | PSMD12 | RABBIT | -2.99083 | -1.67546 | -0.51708 | -0.07179 | -1.32954 | 0.36817 | 0.01415 | 0.11632 | 0.42294 | 0.8568 | 1.672615 | 3.175 | 3.26538 | 0.0131 | 0.00759 | 0.09752 | 0.08098 | 8 | 2.93229 | 0.55262 | 0.69381 | 0.655942 | Significant | Up in Mtub | PSMD12 |
| 866 | G1SEE0 | RB binding protein 4, chromatin remodeling factor C | RBBP4 | RABBIT | -0.98185 | 1.30523 | -2.5053 | -0.60036 | -1.36028 | 1.42705 | 1.06201 | -0.14577 | 0.08141 | 1.77485 | 1.668423 | 2.2985 | 2.50147 | 0.05059 | 0.02955 | 0.15026 | 0.12149 | 8 | 2.93229 | 0.55262 | 1.31724 | 1.11215 | Significant | Up in Mtub | RBBP4 |
| 2707 | G1SY28 | #N/A | #N/A | RABBIT | #N/A | #N/A | #N/A | -1.48955 | #N/A | -0.01288 | #N/A | -0.05149 | #N/A | 0.59116 | 1.66515 | 4.00124 | 2.33563 | 0.05716 | 0.06745 | 0.15449 | 0.17128 | 2 | 2.93229 | 0.55262 | 0.12989 | 0.381205 | Not Significant | NS | #N/A |
| 1462 | A0A5F9DE32 | 26S proteasome non-ATPase regulatory subunit 5 C | PSMD5 | RABBIT | -0.76817 | 1.49405 | -0.57955 | -0.61932 | -0.48624 | 2.13777 | 0.47286 | 1.80032 | #N/A | #N/A | 1.662163 | 2.45824 | 2.61552 | 0.04924 | 0.02819 | 0.14906 | 0.11898 | 6 | 2.93229 | 0.55262 | 0.85724 | 0.757237 | Significant | Up in Mtub | PSMD5 |
| 2451 | G1T7D2 | #N/A | CD53 | RABBIT | #N/A | #N/A | -0.83157 | -0.77605 | -0.36851 | 1.77393 | 2.62033 | 0.23871 | -0.30307 | 0.67586 | 1.659862 | 2.32479 | 2.50468 | 0.05906 | 0.03379 | 0.15506 | 0.1262 | 6 | 2.93229 | 0.55262 | 0.95582 | 0.823457 | Significant | Up in Mtub | CD53 |
| 2000 | G1SPT2 | #N/A | DDX6 | RABBIT | #N/A | #N/A | -1.51438 | 0.13932 | -2.9474 | 0.60075 | -0.48469 | -0.26133 | 0.36191 | 0.87691 | 1.659527 | 2.25427 | 2.44474 | 0.06506 | 0.03727 | 0.15932 | 0.12876 | 6 | 2.93229 | 0.55262 | 1.01615 | 0.863985 | Significant | Up in Mtub | DDX6 |
| 591 | G1TKR2 | Synapsin I OS=Oryctolagus cuniculus OX=9986 GN | SYN1 | RABBIT | -2.43469 | -0.75058 | -0.1354 | 0.08881 | -1.49429 | 1.38138 | -0.14645 | 0.89702 | 0.73763 | 0.69311 | 1.657766 | 3.16032 | 3.24712 | 0.01339 | 0.00783 | 0.09752 | 0.08248 | 8 | 2.93229 | 0.55262 | 0.6879 | 0.651612 | Significant | Up in Mtub | SYN1 |
| 1422 | G1SWU8 | Chromosome 5 open reading frame 51 OS=Oryctola | RIMOC1 | RABBIT | #N/A | #N/A | #N/A | -0.94666 | #N/A | #N/A | 2.13066 | #N/A | -0.07889 | 0.07889 | 1.656878 | 1.16406 | 1.47632 | 0.36448 | 0.20067 | 0.35393 | 0.28387 | 2 | 2.93229 | 0.55262 | 1.51947 | 0.944666 | Not Significant | NS | #N/A |
| 700 | G1SXT1 | Adenosylhomocysteinase like 1 OS=Oryctolagus cu | AHCYL1 | RABBIT | -2.6856 | -0.99941 | 1.02307 | -1.76978 | -0.20794 | 1.75208 | 0.19456 | -0.83739 | 1.1066 | 1.42852 | 1.656807 | 2.09189 | 2.30129 | 0.06981 | 0.04207 | 0.16349 | 0.13611 | 8 | 2.93229 | 0.55262 | 1.56822 | 1.295815 | Significant | Up in Mtub | AHCYL1 |
| 1046 | A0A5F9DC17 | Dmx like 2 OS=Oryctolagus cuniculus OX=9986 GN | DMXL2 | RABBIT | -2.80992 | -0.85607 | 0.45947 | -0.76017 | -0.32304 | 1.10746 | -0.28882 | 1.13037 | 1.33706 | 0.69682 | 1.654523 | 2.69649 | 2.85947 | 0.02722 | 0.01562 | 0.124 | 0.09934 | 8 | 2.93229 | 0.55262 | 0.94121 | 0.836981 | Significant | Up in Mtub | DMXL2 |
| 978 | G1SVT4 | Proteasome 26S subunit, non-ATPase 7 OS=Orycto | PSMD7 | RABBIT | -3.30245 | -1.67804 | -0.50946 | 1.2072 | -1.79951 | -0.06612 | 0.06612 | 0.62496 | 0.3233 | 1.23503 | 1.65311 | 2.10334 | 2.31184 | 0.06858 | 0.0413 | 0.16349 | 0.13462 | 8 | 2.93229 | 0.55262 | 1.54427 | 1.278284 | Significant | Up in Mtub | PSMD7 |
| 2440 | A0A5F9D4W1 | #N/A | #N/A | RABBIT | #N/A | #N/A | -2.44186 | 0.52776 | -2.35852 | 1.34064 | -0.33898 | -0.10931 | 0.04494 | 0.20394 | 1.652455 | 2.03389 | 2.24839 | 0.08819 | 0.05135 | 0.17722 | 0.15166 | 6 | 2.93229 | 0.55262 | 1.23767 | 1.012781 | Not Significant | NS | #N/A |
| 1801 | A0A5S8H7S6 | #N/A | SPTBN2 | RABBIT | #N/A | -0.84467 | #N/A | -0.00498 | -0.49843 | #N/A | #N/A | -0.11702 | 3.13115 | 0.59347 | 1.651895 | 1.62662 | 1.90628 | 0.17915 | 0.0987 | 0.25448 | 0.19816 | 4 | 2.93229 | 0.55262 | 1.54697 | 1.126369 | Not Significant | NS | #N/A |
| 1815 | A0A5F9CUH2 | #N/A | #N/A | RABBIT | #N/A | -2.05717 | #N/A | -0.44141 | #N/A | 1.42074 | 0.08942 | 0.74733 | 0 | -0.25207 | 1.650375 | 2.48539 | 2.54375 | 0.05547 | 0.03474 | 0.15432 | 0.1262 | 5 | 2.93229 | 0.55262 | 0.62991 | 0.601336 | Significant | Up in Mtub | #N/A |
| 1853 | G1SY50 | #N/A | NDUFV2 | RABBIT | #N/A | #N/A | #N/A | -0.67363 | -0.56886 | #N/A | #N/A | 1.49629 | 0.55547 | #N/A | 1.647122 | 3.47997 | 2.54345 | 0.07358 | 0.05233 | 0.1668 | 0.15178 | 2 | 2.93229 | 0.55262 | 0.22403 | 0.419377 | Not Significant | NS | #N/A |
| 635 | A0A5F9CPB0 | dynamin GTPase OS=Oryctolagus cuniculus OX=99 | DNM3 | RABBIT | -4.30227 | -2.18175 | 0.58295 | -0.32497 | -0.88931 | 0.84087 | -0.58335 | 0.20297 | 0.33435 | 0.32038 | 1.646115 | 1.87383 | 2.08382 | 0.09783 | 0.06145 | 0.18746 | 0.16292 | 8 | 2.93229 | 0.55262 | 1.92931 | 1.560048 | Not Significant | NS | #N/A |
| 2287 | A5U0I3 | #N/A | #N/A | MT | #N/A | #N/A | -0.73689 | #N/A | #N/A | 0.90754 | #N/A | #N/A | #N/A | #N/A | 1.644426 | #N/A | 1.56418 | #N/A | 0.21779 | #N/A | 0.29542 | 0 | 2.93229 | 0.55262 | #N/A | 0.552617 | Not Significant | NS | #N/A |
| 1865 | G1TST9 | #N/A | CHD4 | RABBIT | #N/A | 0.19782 | #N/A | -1.49972 | -1.4278 | 1.19238 | 0.03918 | -0.58671 | #N/A | 2.29136 | 1.643954 | 1.85863 | 2.10091 | 0.12219 | 0.06913 | 0.20979 | 0.17249 | 5 | 2.93229 | 0.55262 | 1.34114 | 1.049652 | Not Significant | NS | #N/A |
| 1053 | G1SVA3 | 26S proteasome non-ATPase regulatory subunit 11 | PSMD11 | RABBIT | -3.33281 | -1.89947 | -0.41013 | 0.6295 | -1.60063 | 0.26078 | 0.36976 | 0.1745 | -0.23886 | 1.02558 | 1.641059 | 2.32879 | 2.52418 | 0.04825 | 0.02838 | 0.14885 | 0.11898 | 8 | 2.93229 | 0.55262 | 1.24145 | 1.05669 | Significant | Up in Mtub | PSMD11 |
| 1258 | G1TTY7 | Large ribosomal subunit protein eL20 OS=Oryctolag | RPL18A | RABBIT | -3.35964 | -3.0198 | 0.14163 | -0.59036 | 0.37256 | 0.15043 | 1.22342 | 0.43406 | 0.02902 | -0.09373 | 1.639757 | 1.98206 | 2.19096 | 0.08278 | 0.05103 | 0.17453 | 0.15114 | 8 | 2.93229 | 0.55262 | 1.71105 | 1.400335 | Not Significant | NS | #N/A |
| 2373 | B7NZI5 | #N/A | EIF6 ITGB4 | RABBIT | #N/A | #N/A | #N/A | -1.289 | #N/A | 0.11811 | #N/A | #N/A | -0.11811 | 1.04817 | 1.638392 | 2.30122 | 2.04227 | 0.14802 | 0.09736 | 0.22977 | 0.19688 | 2 | 2.93229 | 0.55262 | 0.38017 | 0.482692 | Not Significant | NS | #N/A |
| 404 | G1TXQ6 | ADP-ribosylation factor OS=Oryctolagus cuniculus | #N/A | RABBIT | -1.67843 | -1.41194 | -0.24117 | 0.2479 | -0.64651 | 0.89678 | 1.65787 | 0.26282 | -0.30554 | 1.9467 | 1.637755 | 2.96686 | 3.08255 | 0.01796 | 0.01049 | 0.11098 | 0.09154 | 8 | 2.93229 | 0.55262 | 0.7618 | 0.705696 | Significant | Up in Mtub | #N/A |
| 2580 | G1T4P0 | #N/A | KRT32 | RABBIT | #N/A | #N/A | #N/A | -0.87616 | #N/A | #N/A | #N/A | 0.75415 | #N/A | #N/A | 1.630307 | #N/A | 1.55075 | #N/A | 0.2208 | #N/A | 0.2975 | 0 | 2.93229 | 0.55262 | #N/A | 0.552617 | Not Significant | NS | #N/A |
| 2159 | A0A5F9DEV8 | #N/A | ICE2 | RABBIT | #N/A | #N/A | -1.33911 | #N/A | -0.82921 | 0.12543 | 1.51298 | #N/A | 0 | #N/A | 1.630293 | 2.4926 | 2.44676 | 0.08828 | 0.05047 | 0.17722 | 0.15075 | 3 | 2.93229 | 0.55262 | 0.51334 | 0.532756 | Not Significant | NS | #N/A |
| 905 | A0A0D3RIY6 | Dihydropteridine reductase OS=Oryctolagus cunicu | #N/A | RABBIT | -1.74412 | -2.43778 | -0.25788 | 0.04076 | -0.20564 | 1.81599 | -0.0984 | 0.28487 | 1.07782 | 0.45337 | 1.627662 | 2.72928 | 2.87934 | 0.02587 | 0.01508 | 0.12204 | 0.09934 | 8 | 2.93229 | 0.55262 | 0.88915 | 0.798882 | Significant | Up in Mtub | #N/A |
| 241 | Q8MI17 | Aldehyde dehydrogenase 1A1 OS=Oryctolagus cuni | ALDH1A1 | RABBIT | 0.24048 | 0.16313 | -0.21388 | -3.2105 | -2.73296 | -2.13145 | 1.35 | -0.02967 | 1.53967 | 1.63713 | 1.623881 | 1.56318 | 1.76238 | 0.15664 | 0.1059 | 0.23659 | 0.20493 | 8 | 2.93229 | 0.55262 | 2.69793 | 2.122505 | Not Significant | NS | #N/A |
| 2685 | B7NZB3 | #N/A | ARHGAP26 | RABBIT | #N/A | #N/A | #N/A | -1.33544 | #N/A | 0.56884 | #N/A | #N/A | #N/A | 0 | 1.619864 | 3.28821 | 1.9646 | 0.18795 | 0.12213 | 0.26074 | 0.22013 | 1 | 2.93229 | 0.55262 | 0.16179 | 0.453227 | Not Significant | NS | #N/A |
| 1910 | A0A5F9CXH1 | #N/A | PDLIM5 | RABBIT | #N/A | -1.95228 | -0.43447 | -0.6984 | -1.69906 | 1.11763 | 0.6604 | 0.18454 | 0.1506 | 0 | 1.618687 | 4.03655 | 3.74571 | 0.00495 | 0.00386 | 0.07293 | 0.06065 | 7 | 2.93229 | 0.55262 | 0.35735 | 0.414997 | Significant | Up in Mtub | PDLIM5 |
| 1086 | G1SQV5 | Eukaryotic translation initiation factor 3 subunit D C | EIF3D EIF3 | RABBIT | #N/A | -1.94425 | -0.77874 | -0.09371 | -1.03336 | 0 | 0.67597 | 0.67498 | 0.68791 | 1.23762 | 1.61781 | 4.01328 | 3.73177 | 0.0051 | 0.00394 | 0.07293 | 0.06094 | 7 | 2.93229 | 0.55262 | 0.36111 | 0.417651 | Significant | Up in Mtub | EIF3D EIF3S7 |
| 1007 | A0A5F9DDJ5 | Alpha-mannosidase OS=Oryctolagus cuniculus OX | #N/A | RABBIT | 0.44757 | -0.94274 | -1.26783 | -0.66108 | -0.43215 | #N/A | 2.91935 | 0.85933 | 0 | 0.40442 | 1.617022 | 2.45681 | 2.62757 | 0.04367 | 0.0254 | 0.14581 | 0.11552 | 7 | 2.93229 | 0.55262 | 0.96267 | 0.841611 | Significant | Up in Mtub | #N/A |
| 116 | A0A5F9CYF1 | Talin 1 OS=Oryctolagus cuniculus OX=9986 GN=TLI | TLN1 | RABBIT | -0.64821 | -1.38376 | -1.20854 | 0.08672 | -0.6146 | -0.14436 | 1.83307 | 0.84521 | 0.88855 | 0.88847 | 1.615866 | 3.97916 | 3.80904 | 0.00407 | 0.00293 | 0.06755 | 0.05519 | 8 | 2.93229 | 0.55262 | 0.41226 | 0.449904 | Significant | Up in Mtub | TLN1 |
| 1118 | A0A5F9DHC7 | Alpha-1-B glycoprotein OS=Oryctolagus cuniculus ( | A1BG | RABBIT | #N/A | -0.39481 | #N/A | -0.03325 | -3.99167 | -2.13195 | 1.32858 | 0.86806 | -0.02439 | 0.66721 | 1.614744 | 1.31402 | 1.53188 | 0.23684 | 0.16017 | 0.29252 | 0.25503 | 6 | 2.93229 | 0.55262 | 2.83143 | 2.083344 | Not Significant | NS | #N/A |
| 1774 | G1SQL0 | #N/A | PSMC1 | RABBIT | #N/A | -3.74175 | -1.97541 | 0.28247 | -0.72864 | 0.84121 | -0.66498 | -0.04847 | 0 | 0.23948 | 1.614278 | 1.99357 | 2.20591 | 0.08644 | 0.0521 | 0.17578 | 0.15166 | 7 | 2.93229 | 0.55262 | 1.45708 | 1.190057 | Not Significant | NS | #N/A |
| 1955 | A0A5F9C3F3 | #N/A | OCRL | RABBIT | #N/A | -0.87302 | #N/A | -0.94611 | -0.7445 | 1.77167 | 0.2414 | 0.97621 | 1.04224 | -0.2414 | 1.612566 | 3.46083 | 3.27403 | 0.01345 | 0.00972 | 0.09752 | 0.09056 | 6 | 2.93229 | 0.55262 | 0.40708 | 0.454854 | Significant | Up in Mtub | OCRL |
| 1096 | G1U567 | Large ribosomal subunit protein eL21 OS=Oryctolag | #N/A | RABBIT | -3.41779 | -4.16074 | 0.23802 | -0.39015 | 0.86573 | -0.0903 | 0.48382 | -0.13174 | 0.3521 | 0.58319 | 1.612402 | 1.57493 | 1.7738 | 0.15392 | 0.10392 | 0.23444 | 0.20293 | 8 | 2.93229 | 0.55262 | 2.62038 | 2.065758 | Not Significant | NS | #N/A |
| 1416 | G1T8T5 | Complement C1q B chain OS=Oryctolagus cuniculu | C1QB | RABBIT | -2.26348 | -2.00528 | #N/A | -0.28454 | #N/A | -2.51074 | 1.89546 | 0.59599 | 0.2629 | 0.2269 | 1.611871 | 1.5205 | 1.74665 | 0.1792 | 0.1149 | 0.25448 | 0.2139 | 6 | 2.93229 | 0.55262 | 2.10712 | 1.596807 | Not Significant | NS | #N/A |
| 1944 | G1SGE9 | #N/A | LDHD | RABBIT | #N/A | -0.70308 | #N/A | #N/A | #N/A | #N/A | 0.9009 | #N/A | #N/A | #N/A | 1.603974 | #N/A | 1.5257 | #N/A | 0.22655 | #N/A | 0.30062 | 0 | 2.93229 | 0.55262 | #N/A | 0.552617 | Not Significant | NS | #N/A |
| 1000 | A0A5F9CPX8 | non-specific serine/threonine protein kinase OS=Or | DCLK2 | RABBIT | -1.94539 | -2.14626 | -0.03177 | 0.52203 | -1.60811 | 1.28649 | 0 | -0.0432 | 1.00325 | #N/A | 1.603534 | 2.35299 | 2.53309 | 0.05086 | 0.02986 | 0.15026 | 0.12188 | 7 | 2.93229 | 0.55262 | 1.03206 | 0.890515 | Significant | Up in Mtub | DCLK2 |
| 1839 | G1SET0 | #N/A | COPB2 | RABBIT | #N/A | #N/A | -1.16505 | -0.97446 | #N/A | 0.78306 | 2.19464 | -0.8847 | 0 | 0.56447 | 1.601247 | 1.88822 | 2.07362 | 0.11763 | 0.07212 | 0.20536 | 0.17493 | 5 | 2.93229 | 0.55262 | 1.02733 | 0.851848 | Not Significant | NS | #N/A |
| 987 | A0AAA9WV72 | Small ribosomal subunit protein uS13 OS=Oryctola | #N/A | RABBIT | -3.78077 | -3.15681 | 0.08344 | 0.01863 | -0.3741 | -0.05916 | 0.43381 | 0.15416 | 0.05916 | 0.20282 | 1.600081 | 1.90255 | 2.10667 | 0.0936 | 0.05907 | 0.18402 | 0.16127 | 8 | 2.93229 | 0.55262 | 1.76828 | 1.442213 | Not Significant | NS | #N/A |
| 1594 | G1SKM5 | NPL4 homolog, ubiquitin recognition factor OS=Ory | NPLOC4 | RABBIT | -2.43054 | -2.50615 | -0.9675 | 0.86109 | -0.9408 | 1.20357 | -0.07509 | 0.01072 | 0.78698 | 0.09007 | 1.60003 | 2.40419 | 2.58404 | 0.0429 | 0.02552 | 0.14581 | 0.11552 | 8 | 2.93229 | 0.55262 | 1.10728 | 0.95851 | Significant | Up in Mtub | NPLOC4 |
| 2875 | G1SW97 | #N/A | IVD | RABBIT | #N/A | #N/A | #N/A | #N/A | -1.61464 | 0.70156 | #N/A | 0.54591 | 0 | -1.32207 | 1.595992 | 1.5524 | 1.70526 | 0.21838 | 0.13959 | 0.28083 | 0.23886 | 3 | 2.93229 | 0.55262 | 0.84556 | 0.700763 | Not Significant | NS | #N/A |
| 1799 | A0A5F9CKB9 | #N/A | PLP1 | RABBIT | #N/A | -1.36553 | #N/A | #N/A | #N/A | #N/A | #N/A | -0.12548 | 0.06112 | 0.75502 | 1.595747 | 2.97855 | 2.14309 | 0.09665 | 0.08574 | 0.1866 | 0.18814 | 2 | 2.93229 | 0.55262 | 0.21527 | 0.415825 | Not Significant | NS | #N/A |
| 2639 | A0A5F9CFL2 | #N/A | ZNF423 | RABBIT | #N/A | #N/A | #N/A | -0.8254 | #N/A | #N/A | 0.76777 | #N/A | #N/A | #N/A | 1.593169 | #N/A | 1.51543 | #N/A | 0.22895 | #N/A | 0.30149 | 0 | 2.93229 | 0.55262 | #N/A | 0.552617 | Not Significant | NS | #N/A |
| 2165 | G1SDA4 | #N/A | COPB1 | RABBIT | #N/A | #N/A | -1.77379 | -0.076 | #N/A | 1.13454 | #N/A | #N/A | 0 | 0.86887 | 1.592698 | 2.06312 | 2.18978 | 0.1311 | 0.07161 | 0.21539 | 0.1745 | 3 | 2.93229 | 0.55262 | 0.71515 | 0.634813 | Not Significant | NS | #N/A |
| 809 | P83468 | 14 kDa phosphohistidine phosphatase OS=Oryctola | PHPT1 PHF | RABBIT | -0.30302 | -1.23267 | 0.00531 | -0.47189 | -0.06746 | 1.38672 | 0.05407 | 0.51232 | 1.68622 | 2.20112 | 1.582036 | 3.5216 | 3.47744 | 0.00783 | 0.00522 | 0.08478 | 0.06727 | 8 | 2.93229 | 0.55262 | 0.50454 | 0.517433 | Significant | Up in Mtub | PHPT1 PHP14 |
| 2791 | A0A5F9D525 | #N/A | #N/A | RABBIT | #N/A | #N/A | #N/A | #N/A | -1.45712 | 0.56543 | 0.1363 | -0.20067 | -0.20068 | 0.29336 | 1.575872 | 4.36543 | 2.6423 | 0.01201 | 0.03361 | 0.09752 | 0.1262 | 4 | 2.93229 | 0.55262 | 0.10859 | 0.296411 | Significant | Up in Mtub | #N/A |
| 951 | G1T823 | ADF-H domain-containing protein OS=Oryctolagus ( | #N/A | RABBIT | -1.92071 | -0.25846 | -0.79013 | -0.78532 | -0.32532 | 0.31194 | 1.26358 | 0.47712 | 0.94052 | 0.79102 | 1.572825 | 4.59675 | 4.13099 | 0.00176 | 0.00169 | 0.05406 | 0.04595 | 8 | 2.93229 | 0.55262 | 0.29268 | 0.362404 | Significant | Up in Mtub | #N/A |
| 562 | P80912 | Adenosine 5'-monophosphoramidase HINT1 OS=Or | HINT1 HIN | RABBIT | -1.88257 | -0.18361 | -0.5427 | -0.35256 | -1.18987 | 0.88746 | 1.12156 | 0.23056 | 0.63564 | 0.83483 | 1.572275 | 4.52704 | 4.09305 | 0.00193 | 0.0018 | 0.05406 | 0.04684 | 8 | 2.93229 | 0.55262 | 0.30156 | 0.368896 | Significant | Up in Mtub | HINT1 HINT |
| 1044 | G1TRM4 | Small ribosomal subunit protein uS17 OS=Oryctola | RPS11 | RABBIT | -3.12517 | -3.77125 | 0.52371 | -0.52426 | -0.49432 | -0.04116 | 0.29636 | 0.06062 | 0.07994 | 0.04116 | 1.565642 | 1.87672 | 2.07633 | 0.0974 | 0.06225 | 0.18714 | 0.16394 | 8 | 2.93229 | 0.55262 | 1.73992 | 1.421455 | Not Significant | NS | #N/A |
| 730 | G1SYI2 | Guanine nucleotide-binding protein G(I)/G(S)/G(T) s | GNB1 | RABBIT | -4.79776 | -1.67317 | -0.06155 | -0.2179 | -0.00465 | 0.60804 | 0.04925 | 0.3877 | -0.00873 | 0.03096 | 1.564449 | 1.69494 | 1.89338 | 0.12853 | 0.08506 | 0.21348 | 0.18794 | 8 | 2.93229 | 0.55262 | 2.12986 | 1.706811 | Not Significant | NS | #N/A |
| 324 | G1SXQ0 | Glutathione S-transferase OS=Oryctolagus cuniculu | GSTM3 | RABBIT | -1.84742 | -0.96738 | -0.28451 | -0.16843 | -0.59748 | 1.59943 | 0.11079 | 0.59622 | 0.59873 | 1.04116 | 1.562311 | 3.98061 | 3.76692 | 0.00406 | 0.00315 | 0.06755 | 0.05686 | 8 | 2.93229 | 0.55262 | 0.3851 | 0.430033 | Significant | Up in Mtub | GSTM3 |
| 1544 | G1SLD5 | Hypoxia up-regulated protein 1 OS=Oryctolagus cur | HYOU1 | RABBIT | #N/A | -0.83362 | #N/A | -1.16864 | #N/A | 1.82232 | #N/A | 0.41935 | 0.3122 | -0.3122 | 1.561547 | 2.28467 | 2.34121 | 0.08435 | 0.0521 | 0.17453 | 0.15166 | 4 | 2.93229 | 0.55262 | 0.62287 | 0.593156 | Not Significant | NS | #N/A |
| 836 | A0A5F9C713 | Lysine--tRNA ligase OS=Oryctolagus cuniculus OX= | KARS1 | RABBIT | -1.48995 | -0.34893 | -1.13471 | -0.04872 | -0.719 | 1.77463 | 0.85056 | -0.07328 | 0.54981 | 0.93623 | 1.555849 | 3.92344 | 3.72597 | 0.0044 | 0.00338 | 0.0684 | 0.05724 | 8 | 2.93229 | 0.55262 | 0.39313 | 0.435911 | Significant | Up in Mtub | KARS1 |
| 1071 | G1SPW0 | Immunoglobulin superfamily member 10 OS=Oryct | IGSF10 | RABBIT | -3.22801 | -1.14047 | 0.33178 | -0.21877 | -0.57134 | 1.6599 | -0.4774 | 0.51286 | 0.42661 | 0.81629 | 1.553014 | 2.20587 | 2.39065 | 0.05846 | 0.03595 | 0.15484 | 0.12868 | 8 | 2.93229 | 0.55262 | 1.23917 | 1.055019 | Significant | Up in Mtub | IGSF10 |
| 1441 | A0A5F9CDJ0 | THO complex 5 OS=Oryctolagus cuniculus OX=998 | THOC5 | RABBIT | -0.488 | #N/A | #N/A | #N/A | #N/A | #N/A | #N/A | #N/A | 0 | 2.11834 | 1.547166 | 0.84336 | 1.27435 | 0.55397 | 0.27264 | 0.43432 | 0.33035 | 1 | 2.93229 | 0.55262 | 2.24368 | 0.982663 | Not Significant | NS | #N/A |
| 2657 | G1SD73 | #N/A | LYPLAL1 | RABBIT | #N/A | #N/A | #N/A | -0.89889 | #N/A | 1.28954 | #N/A | #N/A | 0 | #N/A | 1.54366 | 1.38225 | 1.59617 | 0.39871 | 0.1869 | 0.36992 | 0.27558 | 1 | 2.93229 | 0.55262 | 0.83146 | 0.623527 | Not Significant | NS | #N/A |
| 1769 | A0A5F9CNH0 | #N/A | NRBP1 | RABBIT | #N/A | -0.69878 | #N/A | #N/A | #N/A | #N/A | 1.68901 | #N/A | 0 | #N/A | 1.543282 | 1.05508 | 1.43153 | 0.48294 | 0.22671 | 0.40677 | 0.30062 | 1 | 2.93229 | 0.55262 | 1.42637 | 0.774817 | Not Significant | NS | #N/A |
| 1994 | G1SNP4 | #N/A | THRAP3 | RABBIT | #N/A | -1.55612 | -0.45133 | -1.09005 | #N/A | -0.07663 | 0.45245 | 0.70321 | 0.07663 | 1.38641 | 1.540911 | 3.69555 | 3.33457 | 0.01014 | 0.00883 | 0.09529 | 0.08502 | 6 | 2.93229 | 0.55262 | 0.32599 | 0.400384 | Significant | Up in Mtub | THRAP3 |
| 359 | G1TBC1 | Endoplasmin OS=Oryctolagus cuniculus OX=9986 ( | HSP90B1 | RABBIT | -2.26602 | -0.29454 | #N/A | -0.29443 | #N/A | -0.77668 | 0.75724 | 1.33847 | 0.23679 | 1.38982 | 1.540795 | 2.14372 | 2.31296 | 0.07576 | 0.04622 | 0.16912 | 0.14461 | 6 | 2.93229 | 0.55262 | 0.96862 | 0.832057 | Significant | Up in Mtub | HSP90B1 |
| 365 | G1SCP8 | Moesin OS=Oryctolagus cuniculus OX=9986 PE=4 S | #N/A | RABBIT | 0.19027 | 1.36509 | -0.93297 | -2.00837 | -0.69404 | #N/A | 4.16368 | -0.06437 | -0.32714 | 0.72458 | 1.54019 | 1.3814 | 1.58059 | 0.20964 | 0.14526 | 0.27454 | 0.24441 | 7 | 2.93229 | 0.55262 | 2.76247 | 2.110062 | Not Significant | NS | #N/A |
| 1079 | G1SN16 | DNA repair nuclease/redox regulator APEX1 OS=Ory | APEX1 | RABBIT | -6.51261 | -0.56224 | 1.536 | -0.26596 | -1.35609 | 0.59186 | -0.90282 | 0.19269 | 0.20832 | 0.40661 | 1.53151 | 1.10954 | 1.27031 | 0.29943 | 0.23034 | 0.32408 | 0.30263 | 8 | 2.93229 | 0.55262 | 4.76318 | 3.633809 | Not Significant | NS | #N/A |
| 390 | P00569 | Adenylate kinase isoenzyme 1 OS=Oryctolagus cun | AK1 | RABBIT | -2.41182 | -2.04886 | 2.8005 | -0.73436 | -0.83224 | 0.02236 | 1.14113 | 2.62758 | -0.02236 | 0.61795 | 1.522691 | 1.46023 | 1.64674 | 0.18235 | 0.12802 | 0.25737 | 0.22682 | 8 | 2.93229 | 0.55262 | 2.71846 | 2.137532 | Not Significant | NS | #N/A |
| 1110 | G1SR51 | Anion exchange protein OS=Oryctolagus cuniculus | SLC4A4 | RABBIT | -1.0262 | -0.94055 | -0.20329 | -0.16599 | -0.19083 | 1.65126 | 0.24274 | 0.04399 | 1.53156 | 1.60811 | 1.520904 | 3.72803 | 3.57406 | 0.0058 | 0.00441 | 0.07337 | 0.06401 | 8 | 2.93229 | 0.55262 | 0.41609 | 0.452709 | Significant | Up in Mtub | SLC4A4 |
| 494 | P29678 | Dual specificity mitogen-activated protein kinase ki | MAP2K1 M | RABBIT | -2.20195 | -1.83106 | -0.02544 | 0.31371 | -1.0647 | 1.27778 | -0.37147 | 0.13172 | 0.87567 | 0.87162 | 1.518953 | 2.65001 | 2.77455 | 0.02926 | 0.01817 | 0.12708 | 0.10478 | 8 | 2.93229 | 0.55262 | 0.82136 | 0.749276 | Significant | Up in Mtub | MAP2K1 MEK1 PRKMK1 |
| 1988 | A0A5F9DSW5 | #N/A | NADK2 | RABBIT | #N/A | #N/A | #N/A | -1.18449 | #N/A | 2.30951 | -2.21045 | -0.06437 | 1.30215 | #N/A | 1.518703 | 0.69461 | 0.91433 | 0.53726 | 0.39618 | 0.42838 | 0.39935 | 3 | 2.93229 | 0.55262 | 3.8243 | 2.207128 | Not Significant | NS | #N/A |
| 1885 | G1SK61 | #N/A | PLXNA1 | RABBIT | #N/A | -0.38629 | #N/A | #N/A | #N/A | 2.26026 | 0 | #N/A | #N/A | #N/A | 1.51642 | 0.7747 | 1.20165 | 0.58039 | 0.29683 | 0.44328 | 0.34543 | 1 | 2.93229 | 0.55262 | 2.55438 | 1.061675 | Not Significant | NS | #N/A |
| 1159 | A0A5F9D0K3 | FRY like transcription coactivator OS=Oryctolagus c | FRYL | RABBIT | -0.35403 | 0.15422 | #N/A | 0.29563 | 0.03021 | #N/A | -0.75229 | 3.84772 | #N/A | #N/A | 1.516211 | 1.06482 | 1.30726 | 0.34697 | 0.23281 | 0.34583 | 0.30378 | 4 | 2.93229 | 0.55262 | 2.70338 | 1.793628 | Not Significant | NS | #N/A |
| 1842 | A0A5F9C6N9 | #N/A | ACSF2 | RABBIT | #N/A | -1.03549 | 0.93131 | #N/A | -0.77405 | #N/A | #N/A | #N/A | #N/A | 1.21989 | 1.512627 | 1.22646 | 1.47278 | 0.34482 | 0.20158 | 0.34542 | 0.28418 | 2 | 2.93229 | 0.55262 | 1.14082 | 0.79113 | Not Significant | NS | #N/A |
| 349 | U3KMH9 | Malate dehydrogenase OS=Oryctolagus cuniculus ( | MDH2 | RABBIT | -2.19285 | -0.46443 | #N/A | -0.05764 | -0.65206 | 1.32898 | -0.30911 | 1.12113 | 0.7646 | 0.42882 | 1.50863 | 2.87538 | 2.91729 | 0.02381 | 0.01548 | 0.11922 | 0.09934 | 7 | 2.93229 | 0.55262 | 0.61174 | 0.594282 | Significant | Up in Mtub | MDH2 |
| 2258 | A0A5F9DIN9 | #N/A | CLUH | RABBIT | #N/A | #N/A | -0.78318 | #N/A | -0.01338 | #N/A | 1.27599 | -0.14297 | 2.18985 | #N/A | 1.505907 | 1.63342 | 1.85722 | 0.20089 | 0.11322 | 0.27128 | 0.21238 | 3 | 2.93229 | 0.55262 | 1.01995 | 0.788952 | Not Significant | NS | #N/A |
| 1836 | A0A5F9DNF1 | #N/A | FRMD6 | RABBIT | #N/A | -1.16847 | 0.17065 | #N/A | #N/A | #N/A | 1.00684 | #N/A | #N/A | #N/A | 1.505752 | 1.29839 | 1.53668 | 0.41781 | 0.20038 | 0.37849 | 0.28371 | 1 | 2.93229 | 0.55262 | 0.89662 | 0.640098 | Not Significant | NS | #N/A |
| 1439 | G1SYK3 | mitogen-activated protein kinase kinase OS=Orycto | MAP2K4 | RABBIT | -0.99952 | -1.05517 | -1.04214 | 1.58774 | -1.80663 | 1.78264 | -1.68694 | 0.94682 | 1.57866 | 1.59013 | 1.505407 | 1.72806 | 1.92018 | 0.12224 | 0.08129 | 0.20979 | 0.18476 | 8 | 2.93229 | 0.55262 | 1.89728 | 1.536608 | Not Significant | NS | #N/A |
| 2550 | A0A3B0IP62 | #N/A | ADIB | RABBIT | #N/A | #N/A | #N/A | -1.59568 | #N/A | -2.60563 | 1.70126 | 0.42753 | 0.12027 | -0.12027 | 1.500316 | 0.87279 | 1.06474 | 0.43204 | 0.32267 | 0.3831 | 0.35646 | 4 | 2.93229 | 0.55262 | 2.46246 | 1.654615 | Not Significant | NS | #N/A |
| 1603 | G1SDF3 | Uncharacterized protein OS=Oryctolagus cuniculus | #N/A | RABBIT | #N/A | 0.50579 | -0.32052 | #N/A | -0.32135 | 1.45248 | #N/A | #N/A | #N/A | #N/A | 1.497843 | 2.71766 | 1.9994 | 0.11292 | 0.1028 | 0.20012 | 0.20213 | 2 | 2.93229 | 0.55262 | 0.22783 | 0.420917 | Not Significant | NS | #N/A |
| 1925 | G1U8B3 | #N/A | #N/A | RABBIT | #N/A | -0.16722 | -0.16443 | #N/A | #N/A | 0.33508 | -0.44356 | #N/A | 1.57761 | 3.85694 | 1.497338 | 1.063 | 1.30317 | 0.3477 | 0.23413 | 0.34597 | 0.3048 | 4 | 2.93229 | 0.55262 | 2.64553 | 1.760249 | Not Significant | NS | #N/A |
| 1529 | B8K1B7 | Trifunctional purine biosynthetic protein adenosine- | GART | RABBIT | #N/A | -3.66615 | -0.929 | -0.19968 | -0.42917 | 0.15269 | 0.07016 | -0.06437 | 0.36378 | 0.4165 | 1.493755 | 2.10046 | 2.27835 | 0.07382 | 0.04608 | 0.16683 | 0.14461 | 7 | 2.93229 | 0.55262 | 1.12387 | 0.955224 | Significant | Up in Mtub | GART |
| 1579 | A0A5F9DNV0 | Uncharacterized protein OS=Oryctolagus cuniculus | #N/A | RABBIT | 0.34928 | #N/A | -1.34373 | #N/A | -0.351 | 1.04326 | #N/A | #N/A | #N/A | #N/A | 1.491739 | 1.5186 | 1.63807 | 0.26819 | 0.16313 | 0.30684 | 0.25811 | 2 | 2.93229 | 0.55262 | 0.7237 | 0.621989 | Not Significant | NS | #N/A |
| 623 | G1SSA2 | 26S proteasome non-ATPase regulatory subunit 2 C | PSMD2 | RABBIT | -3.50833 | -1.37227 | -0.36727 | 0.2164 | -0.82993 | 0.2993 | -0.21036 | 0.36456 | 0.21036 | 0.91865 | 1.488784 | 2.23873 | 2.40592 | 0.05554 | 0.03499 | 0.15432 | 0.12684 | 8 | 2.93229 | 0.55262 | 1.10561 | 0.957281 | Significant | Up in Mtub | PSMD2 |
| 1248 | G1SUU2 | Anion-transporting ATPase-like domain-containing p | #N/A | RABBIT | -2.15158 | -0.90723 | 0.17065 | 0.02895 | -0.10882 | 1.31226 | 0.46929 | #N/A | #N/A | #N/A | 1.484382 | 1.96222 | 2.09147 | 0.10698 | 0.07015 | 0.19677 | 0.17293 | 5 | 2.93229 | 0.55262 | 0.81752 | 0.719595 | Not Significant | NS | #N/A |
| 1309 | A0A5F9CDT9 | Cytoplasmic linker associated protein 2 OS=Orycto | CLASP2 | RABBIT | #N/A | -3.66029 | -1.26043 | 0.16944 | -0.16673 | 0.83926 | -0.4422 | 0.41406 | #N/A | 0.15335 | 1.470621 | 1.62251 | 1.83462 | 0.15582 | 0.10001 | 0.23612 | 0.20009 | 6 | 2.93229 | 0.55262 | 1.64308 | 1.285104 | Not Significant | NS | #N/A |
| 1057 | G1U1H1 | Cell division protein kinase 5 OS=Oryctolagus cunic | CDK5 | RABBIT | -3.05803 | -4.2699 | 2.41661 | -0.03008 | -1.90781 | 0.79882 | -0.85867 | -0.09192 | 0.30855 | 0.34421 | 1.47004 | 1.216 | 1.38366 | 0.25864 | 0.19406 | 0.30309 | 0.27937 | 8 | 2.93229 | 0.55262 | 3.65366 | 2.821891 | Not Significant | NS | #N/A |
| 1518 | G1TU00 | Ribosomal protein L26 like 1 OS=Oryctolagus cunic | RPL26L1 | RABBIT | -4.7706 | -2.14342 | 0.43169 | -1.19489 | 0.66383 | 0.07145 | -0.27206 | 0.47519 | -0.07145 | 0.12013 | 1.467331 | 1.47199 | 1.6546 | 0.17924 | 0.1264 | 0.25448 | 0.2257 | 8 | 2.93229 | 0.55262 | 2.48421 | 1.966114 | Not Significant | NS | #N/A |
| 777 | G1T568 | Transportin 1 OS=Oryctolagus cuniculus OX=9986 ( | TNPO1 | RABBIT | -2.04741 | -0.86526 | 0.09039 | -1.68101 | -0.60834 | 0.08026 | 0.71592 | 0.31973 | 0.61361 | 0.49202 | 1.466633 | 3.68295 | 3.50257 | 0.00619 | 0.00499 | 0.07337 | 0.06702 | 8 | 2.93229 | 0.55262 | 0.39645 | 0.438339 | Significant | Up in Mtub | TNPO1 |
| 1273 | A0A5F9DKP4 | Peptidyl-prolyl cis-trans isomerase OS=Oryctolagus | #N/A | RABBIT | -1.10569 | -1.25657 | -1.12239 | 1.50825 | -1.06116 | 1.62584 | 0.10348 | -0.16785 | 1.50647 | 1.18215 | 1.457529 | 2.2553 | 2.41276 | 0.05412 | 0.03457 | 0.15365 | 0.1262 | 8 | 2.93229 | 0.55262 | 1.04416 | 0.912318 | Significant | Up in Mtub | #N/A |

| | | | | | | | | | | | | | | | | | | | | | | | | | | | | | |
|---|---|---|---|---|---|---|---|---|---|---|---|---|---|---|---|---|---|---|---|---|---|---|---|---|---|---|---|---|---|
| 469 | A0A5F9C3T0 | D-3-phosphoglycerate dehydrogenase OS=Oryctola | PHGDH | RABBIT | -2.06471 | -1.40101 | #N/A | 0.06595 | #N/A | #N/A | #N/A | -0.07644 | 0.01207 | 1.03511 | 1.456839 | 2.0136 | 2.15298 | 0.11433 | 0.06869 | 0.20093 | 0.17193 | 4 | 2.93229 | 0.55262 | 0.78518 | 0.686808 | Not Significant | NS | #N/A |
| 1828 | G1SLL8 | #N/A | WDFY1 | RABBIT | #N/A | -3.5439 | #N/A | 0.08501 | -0.93156 | 0.31961 | -0.14265 | 0.21986 | 0.73188 | -1.1649 | 1.456241 | 1.62164 | 1.82253 | 0.15601 | 0.10195 | 0.23614 | 0.20137 | 6 | 2.93229 | 0.55262 | 1.51202 | 1.197068 | Not Significant | NS | #N/A |
| 1385 | G1T9Y5 | COMM domain containing 9 OS=Oryctolagus cunicu | COMMD9 | RABBIT | #N/A | #N/A | -1.41295 | #N/A | #N/A | 0.21963 | -0.21418 | 0.14981 | -0.32736 | 0.36949 | 1.452424 | 4.46672 | 2.48537 | 0.0111 | 0.0422 | 0.09732 | 0.13627 | 4 | 2.93229 | 0.55262 | 0.08811 | 0.284592 | Significant | Up in Mtub | COMMD9 |
| 2625 | A0A5F9DHL5 | #N/A | QSER1 | RABBIT | #N/A | #N/A | #N/A | -0.78443 | #N/A | #N/A | #N/A | 0.66243 | #N/A | #N/A | 1.446862 | #N/A | 1.37626 | #N/A | 0.26443 | #N/A | 0.32681 | 0 | 2.93229 | 0.55262 | #N/A | 0.552617 | Not Significant | NS | #N/A |
| 1758 | A0A5F9CJM7 | #N/A | ABR | RABBIT | #N/A | -1.55273 | 0.04426 | 1.89599 | -0.01338 | #N/A | #N/A | 1.53854 | #N/A | #N/A | 1.445001 | 0.91597 | 1.14238 | 0.42723 | 0.2973 | 0.38091 | 0.34543 | 3 | 2.93229 | 0.55262 | 1.99096 | 1.279996 | Not Significant | NS | #N/A |
| 1597 | G1SCX2 | DIRAS family GTPase 2 OS=Oryctolagus cuniculus ( | DIRAS2 | RABBIT | #N/A | -2.20142 | -1.00967 | 0.74632 | -2.41233 | 1.31529 | -1.69221 | -0.06437 | 0.78938 | 0.75745 | 1.440386 | 1.6506 | 1.84407 | 0.1428 | 0.09517 | 0.22477 | 0.19659 | 7 | 2.93229 | 0.55262 | 1.69223 | 1.355784 | Not Significant | NS | #N/A |
| 794 | G1T3V2 | Heat shock protein beta-1 OS=Oryctolagus cunicul | HSPB1 | RABBIT | -1.32391 | -1.45231 | 0.21509 | -0.30368 | -0.81164 | 1.04555 | -0.04444 | 0.20414 | 1.55895 | 0.70232 | 1.428591 | 3.36116 | 3.26468 | 0.00991 | 0.00759 | 0.09414 | 0.08098 | 8 | 2.93229 | 0.55262 | 0.45162 | 0.478712 | Significant | Up in Mtub | HSPB1 |
| 938 | A0A5F9CJC1 | Elongin-C OS=Oryctolagus cuniculus OX=9986 GN= | ELOC | RABBIT | -0.54009 | -3.05432 | -0.51931 | -0.15306 | -0.15489 | 0.59518 | 0.09542 | 0.3559 | 0.94635 | 0.72716 | 1.428335 | 2.51281 | 2.62653 | 0.03621 | 0.02366 | 0.13507 | 0.11338 | 8 | 2.93229 | 0.55262 | 0.80776 | 0.739324 | Significant | Up in Mtub | ELOC |
| 2270 | A0A5F9DPL9 | #N/A | ASAP1 | RABBIT | #N/A | #N/A | 0.03652 | -0.53371 | #N/A | 2.56951 | #N/A | #N/A | 0.96714 | 0 | 1.427479 | 1.44135 | 1.67805 | 0.24514 | 0.14491 | 0.29719 | 0.24406 | 3 | 2.93229 | 0.55262 | 1.17701 | 0.868378 | Not Significant | NS | #N/A |
| 2352 | A0A5F9C6H4 | #N/A | FSD1L | RABBIT | #N/A | #N/A | 0.17065 | #N/A | #N/A | #N/A | #N/A | #N/A | -0.18709 | 3.37304 | 1.422323 | 0.46132 | 0.81636 | 0.72484 | 0.46086 | 0.49085 | 0.42527 | 1 | 2.93229 | 0.55262 | 6.33727 | 2.023682 | Not Significant | NS | #N/A |
| 1970 | G1SRD0 | #N/A | CASP3 | RABBIT | #N/A | #N/A | #N/A | -0.34663 | -1.6009 | #N/A | -0.40017 | 0.53171 | 0.28899 | 1.36587 | 1.420362 | 2.1259 | 2.1588 | 0.10067 | 0.06811 | 0.18996 | 0.17147 | 4 | 2.93229 | 0.55262 | 0.59519 | 0.57718 | Not Significant | NS | #N/A |
| 1128 | U3KNK4 | Mannose-1-phosphate guanyltransferase alpha OS | GMPPA | RABBIT | #N/A | #N/A | -0.70644 | -0.90913 | -0.95652 | 1.58216 | 0.03294 | -0.09731 | 0.95107 | 0.34332 | 1.4198 | 3.37598 | 3.05803 | 0.01493 | 0.01373 | 0.10089 | 0.09774 | 6 | 2.93229 | 0.55262 | 0.33163 | 0.404177 | Significant | Up in Mtub | GMPPA |
| 309 | A0A5F9DEN1 | Coronin OS=Oryctolagus cuniculus OX=9986 GN=C | CORO1A | RABBIT | -0.62005 | -0.97436 | -0.81779 | -0.40742 | -0.59777 | 0.76581 | 0.80797 | 0.28541 | 0.88366 | 0.91639 | 1.415325 | 9.39815 | 5.13778 | 1.3E-05 | 0.00033 | 0.00984 | 0.02295 | 8 | 2.93229 | 0.55262 | 0.0567 | 0.189715 | Significant | Up in Mtub | CORO1A |
| 2282 | A0A5F9CT46 | #N/A | RAB21 | RABBIT | #N/A | #N/A | -1.63737 | -0.31534 | #N/A | 1.29385 | 0 | 0.52734 | -0.02923 | 0.40247 | 1.415241 | 2.6575 | 2.49495 | 0.04502 | 0.03747 | 0.14686 | 0.12876 | 5 | 2.93229 | 0.55262 | 0.40515 | 0.459663 | Significant | Up in Mtub | RAB21 |
| 1162 | A0A5F9DJZ1 | Transportin 3 OS=Oryctolagus cuniculus OX=9986 ( | TNPO3 | RABBIT | -1.47563 | -0.23056 | -1.21112 | 0.18835 | -0.01338 | #N/A | #N/A | 0.62499 | #N/A | 1.0999 | 1.410915 | 2.46413 | 2.38607 | 0.05694 | 0.04438 | 0.15449 | 0.14118 | 5 | 2.93229 | 0.55262 | 0.46836 | 0.499504 | Significant | Up in Mtub | TNPO3 |
| 2762 | G1TUZ4 | #N/A | STK33 | RABBIT | #N/A | #N/A | #N/A | -0.73293 | #N/A | 0.67529 | #N/A | #N/A | #N/A | #N/A | 1.408227 | #N/A | 1.33951 | #N/A | 0.27476 | #N/A | 0.33144 | 0 | 2.93229 | 0.55262 | #N/A | 0.552617 | Not Significant | NS | #N/A |
| 705 | A0A5F9C573 | Aldo-keto reductase family 7 like (gene/pseudogene | AKR7L | RABBIT | -1.46945 | -1.98177 | -0.60269 | 0.13481 | -0.58217 | 1.21451 | -0.19244 | 0.36866 | 0.87145 | 0.26661 | 1.406012 | 3.16207 | 3.11318 | 0.01335 | 0.00994 | 0.09752 | 0.09112 | 8 | 2.93229 | 0.55262 | 0.49428 | 0.50993 | Significant | Up in Mtub | AKR7L |
| 949 | G1SL57 | Uncharacterized protein OS=Oryctolagus cuniculus | #N/A | RABBIT | -1.27377 | #N/A | 0.44974 | -3.10746 | -0.68751 | 0.43757 | 0.91087 | 0.19274 | 0 | -0.30825 | 1.401332 | 2.0223 | 2.18366 | 0.08285 | 0.05409 | 0.17453 | 0.15379 | 7 | 2.93229 | 0.55262 | 1.06703 | 0.915162 | Not Significant | NS | #N/A |
| 2500 | A0A5F9D512 | #N/A | SNX2 | RABBIT | #N/A | #N/A | #N/A | -1.2312 | #N/A | 0.94018 | 0.00492 | -0.94042 | -0.00492 | 0.8491 | 1.400973 | 1.67023 | 1.69091 | 0.17019 | 0.13512 | 0.24886 | 0.23421 | 4 | 2.93229 | 0.55262 | 0.58631 | 0.572056 | Not Significant | NS | #N/A |
| 2777 | A0A5F9CPS8 | #N/A | MACF1 | RABBIT | #N/A | #N/A | #N/A | #N/A | -0.01338 | 1.35839 | #N/A | -1.01482 | -1.43989 | 6.63299 | 1.39755 | 0.33697 | 0.46482 | 0.75833 | 0.65863 | 0.50158 | 0.49906 | 3 | 2.93229 | 0.55262 | 13.7604 | 7.231891 | Not Significant | NS | #N/A |
| 1600 | G1SL38 | Twinfilin-1 OS=Oryctolagus cuniculus OX=9986 GN | TWF1 | RABBIT | #N/A | -1.92109 | -0.76322 | -0.22971 | -0.46178 | 0.9431 | 0.06628 | -0.06437 | 1.06448 | 0.7388 | 1.393609 | 3.31066 | 3.12954 | 0.01293 | 0.01078 | 0.09752 | 0.09154 | 7 | 2.93229 | 0.55262 | 0.39377 | 0.440665 | Significant | Up in Mtub | TWF1 |
| 2332 | A0A5F9C4U7 | #N/A | TPST1 | RABBIT | #N/A | #N/A | 0.96114 | #N/A | -3.78614 | -0.58634 | 0.50802 | #N/A | 0.23876 | -0.23876 | 1.392923 | 0.92929 | 1.14819 | 0.40534 | 0.28896 | 0.37324 | 0.33957 | 4 | 2.93229 | 0.55262 | 2.99567 | 1.962284 | Not Significant | NS | #N/A |
| 2672 | A0A5F9C6E6 | #N/A | IL1RAP | RABBIT | #N/A | #N/A | #N/A | #N/A | -0.88972 | #N/A | #N/A | #N/A | 0 | 1.0061 | 1.392766 | 1.59848 | 1.54638 | 0.35589 | 0.19812 | 0.35042 | 0.28262 | 1 | 2.93229 | 0.55262 | 0.50612 | 0.540792 | Not Significant | NS | #N/A |
| 1334 | A0AAA9WYR1 | Small ribosomal subunit protein uS11 OS=Oryctola | #N/A | RABBIT | -3.13666 | -1.1096 | 0.07576 | -0.04997 | -0.4 | -0.00766 | 0.54078 | 0.56712 | 0.42271 | 0.81666 | 1.392014 | 2.29884 | 2.43202 | 0.05056 | 0.03342 | 0.15026 | 0.1262 | 8 | 2.93229 | 0.55262 | 0.91666 | 0.819019 | Significant | Up in Mtub | #N/A |
| 1418 | A0A5F9CCF5 | Argonaute RISC catalytic component 2 OS=Oryctol | AGO2 | RABBIT | -0.54645 | #N/A | #N/A | #N/A | #N/A | #N/A | #N/A | 1.68978 | 0 | #N/A | 1.391345 | 0.95077 | 1.29032 | 0.51606 | 0.26759 | 0.42132 | 0.32868 | 1 | 2.93229 | 0.55262 | 1.42768 | 0.775151 | Not Significant | NS | #N/A |
| 555 | A0A5F9C8S6 | Sulfurtransferase OS=Oryctolagus cuniculus OX=9 | #N/A | RABBIT | -1.77973 | -2.6486 | -0.63456 | -0.56717 | 1.06246 | 1.21432 | -0.11318 | 0.04882 | 1.11535 | 0.12285 | 1.391149 | 2.02155 | 2.18373 | 0.07787 | 0.05168 | 0.17122 | 0.15166 | 8 | 2.93229 | 0.55262 | 1.18391 | 1.014586 | Not Significant | NS | #N/A |
| 2094 | G1STX3 | #N/A | SCFD1 | RABBIT | #N/A | #N/A | #N/A | -1.38057 | #N/A | 0.01372 | -0.39332 | 0.41473 | 0.0244 | -0.01372 | 1.389732 | 4.43538 | 2.39342 | 0.01137 | 0.04827 | 0.09732 | 0.14779 | 4 | 2.93229 | 0.55262 | 0.08181 | 0.280958 | Significant | Up in Mtub | SCFD1 |
| 929 | A0A5F9DSD7 | Electron transfer flavoprotein subunit alpha OS=Or | ETFA | RABBIT | -1.80793 | #N/A | 0.31029 | -1.27028 | -0.98864 | 1.22969 | 0 | 0.40186 | 0.67026 | -0.06015 | 1.387474 | 2.90459 | 2.86683 | 0.02284 | 0.01687 | 0.11776 | 0.10198 | 7 | 2.93229 | 0.55262 | 0.50707 | 0.520516 | Significant | Up in Mtub | ETFA |
| 1202 | G1SM51 | 26S proteasome non-ATPase regulatory subunit 13 | #N/A | RABBIT | -3.34378 | -1.25116 | -0.38084 | 0.5258 | -1.13181 | 0.18282 | 0.28227 | 0.25115 | -0.18282 | 0.81965 | 1.386969 | 2.09897 | 2.25349 | 0.06905 | 0.04575 | 0.16349 | 0.14393 | 8 | 2.93229 | 0.55262 | 1.09159 | 0.947026 | Significant | Up in Mtub | #N/A |
| 1496 | A0A5F9CIV0 | Vasorin OS=Oryctolagus cuniculus OX=9986 GN=V | VASN | RABBIT | -0.25546 | #N/A | #N/A | #N/A | -0.01338 | #N/A | #N/A | #N/A | #N/A | 1.25232 | 1.386746 | 6.61473 | 1.7481 | 0.09552 | 0.15659 | 0.1866 | 0.25377 | 1 | 2.93229 | 0.55262 | 0.0293 | 0.419535 | Not Significant | NS | #N/A |
| 852 | G1SKF7 | Large ribosomal subunit protein eL6 OS=Oryctolagu | RPL6 | RABBIT | -3.62838 | -2.48259 | 0.40644 | -0.72464 | 0.23656 | -0.13893 | 0.04065 | 0.71122 | 0.1663 | -0.04065 | 1.386241 | 1.72929 | 1.905 | 0.12201 | 0.08341 | 0.20979 | 0.18597 | 8 | 2.93229 | 0.55262 | 1.6065 | 1.323822 | Not Significant | NS | #N/A |
| 1041 | A0A5F9D3Y5 | non-specific serine/threonine protein kinase OS=Or | CIT | RABBIT | 0.01166 | #N/A | -0.4805 | #N/A | #N/A | -0.05206 | #N/A | 1.48636 | 2.01944 | #N/A | 1.385666 | 1.68481 | 1.83584 | 0.19061 | 0.11661 | 0.26255 | 0.21451 | 3 | 2.93229 | 0.55262 | 0.81171 | 0.68364 | Not Significant | NS | #N/A |
| 1436 | A0A5F9CVV7 | Serine/threonine-protein phosphatase OS=Oryctola | PPP6C | RABBIT | -1.09332 | -0.47285 | -0.01062 | 0.12364 | -0.9663 | 1.51948 | 0.548 | #N/A | #N/A | 0.63728 | 1.385479 | 3.489 | 3.07751 | 0.013 | 0.01331 | 0.09752 | 0.09752 | 6 | 2.93229 | 0.55262 | 0.29566 | 0.380016 | Significant | Up in Mtub | PPP6C |
| 523 | A0A5F9CZT7 | RRM domain-containing protein OS=Oryctolagus cu | #N/A | RABBIT | -2.02214 | 0.19782 | 0.02399 | -0.06814 | -0.05851 | 0.56634 | 0.64663 | 1.06342 | #N/A | 1.71849 | 1.384116 | 2.65511 | 2.68903 | 0.0327 | 0.02286 | 0.132 | 0.11217 | 7 | 2.93229 | 0.55262 | 0.60391 | 0.588764 | Significant | Up in Mtub | #N/A |
| 1369 | B7NZD2 | 26S proteasome non-ATPase regulatory subunit 4 C | PSMD4 | RABBIT | -1.80189 | -1.06739 | -0.67581 | 0.14813 | -1.36229 | 0.68558 | 0.52975 | -0.27013 | 0.58197 | 0.61319 | 1.37992 | 3.67971 | 3.42632 | 0.00622 | 0.00571 | 0.07337 | 0.06991 | 8 | 2.93229 | 0.55262 | 0.35158 | 0.4055 | Significant | Up in Mtub | PSMD4 |
| 564 | A0A5F9DKF6 | 40S ribosomal protein S4 OS=Oryctolagus cuniculu | #N/A | RABBIT | -3.04402 | -2.17421 | -0.02189 | -0.18593 | -0.22405 | 0.203 | 0.1283 | 0.46642 | 0.14463 | 0.30715 | 1.379919 | 2.21362 | 2.35392 | 0.05775 | 0.03835 | 0.15468 | 0.12996 | 8 | 2.93229 | 0.55262 | 0.9715 | 0.859143 | Significant | Up in Mtub | #N/A |
| 848 | A0A5F9DQ12 | DnaJ heat shock protein family (Hsp40) member C6 | DNAJC6 | RABBIT | -1.76888 | -2.36628 | -0.89307 | 0.28902 | -0.35562 | 1.2098 | 0.43244 | 0.54144 | 0.34223 | -0.72864 | 1.378418 | 2.41975 | 2.53035 | 0.04187 | 0.02807 | 0.14462 | 0.11898 | 8 | 2.93229 | 0.55262 | 0.81126 | 0.741889 | Significant | Up in Mtub | DNAJC6 |
| 891 | A0A5F9DI63 | AT-hook containing transcription factor 1 OS=Oryct | AHCTF1 | RABBIT | -1.13662 | #N/A | 0.58142 | -0.02952 | -4.04445 | #N/A | -0.02812 | #N/A | 0.45732 | #N/A | 1.371892 | 0.88732 | 1.10029 | 0.42504 | 0.30794 | 0.38058 | 0.35174 | 4 | 2.93229 | 0.55262 | 3.18723 | 2.072816 | Not Significant | NS | #N/A |
| 2358 | G1TKT9 | #N/A | CCDC30 | RABBIT | #N/A | #N/A | #N/A | -0.05764 | -1.81314 | 1.93302 | -1.79129 | -1.11824 | 1.73563 | 1.41509 | 1.370232 | 0.98559 | 1.17439 | 0.3696 | 0.27429 | 0.35531 | 0.33142 | 5 | 2.93229 | 0.55262 | 2.76121 | 1.944773 | Not Significant | NS | #N/A |
| 791 | A0A5F9DIR6 | Heat shock protein family A (Hsp70) member 12A O | HSPA12A | RABBIT | -3.5111 | -1.6048 | -0.11378 | 0.33644 | -1.5348 | 0.65615 | -1.23916 | 0.22007 | 0.3061 | 0.45558 | 1.365353 | 1.80806 | 1.97777 | 0.10821 | 0.07371 | 0.1968 | 0.17651 | 8 | 2.93229 | 0.55262 | 1.42562 | 1.191459 | Not Significant | NS | #N/A |
| 1658 | G1STB6 | #N/A | ACTC1 | RABBIT | #N/A | -1.16304 | #N/A | -0.50106 | #N/A | 0.44342 | #N/A | #N/A | 0.62145 | #N/A | 1.364479 | 3.98097 | 2.22471 | 0.05769 | 0.07741 | 0.15468 | 0.18205 | 2 | 2.93229 | 0.55262 | 0.11748 | 0.376172 | Not Significant | NS | #N/A |
| 2346 | P9WJQ7 | #N/A | #N/A | MT | #N/A | #N/A | #N/A | #N/A | -1.30706 | #N/A | #N/A | 0.11331 | #N/A | 0 | 1.363715 | 13.8976 | 1.73112 | 0.04573 | 0.15971 | 0.14728 | 0.25503 | 1 | 2.93229 | 0.55262 | 0.00642 | 0.413716 | Not Significant | NS | #N/A |
| 2687 | G1T0C6 | #N/A | GREB1L | RABBIT | #N/A | #N/A | #N/A | -0.71042 | #N/A | #N/A | #N/A | #N/A | 0.65279 | #N/A | 1.36321 | #N/A | 1.29669 | #N/A | 0.28735 | #N/A | 0.33837 | 0 | 2.93229 | 0.55262 | #N/A | 0.552617 | Not Significant | NS | #N/A |
| 2699 | A0A5F9C4H1 | #N/A | AIFM1 | RABBIT | #N/A | #N/A | #N/A | -0.38644 | #N/A | #N/A | #N/A | -0.2301 | 0.16574 | 2.98783 | 1.360934 | 0.67164 | 0.93848 | 0.571 | 0.39164 | 0.43989 | 0.396 | 2 | 2.93229 | 0.55262 | 3.07934 | 1.577182 | Not Significant | NS | #N/A |
| 1722 | G1SYS5 | #N/A | ALDH7A1 | RABBIT | #N/A | -0.61599 | -0.03694 | -0.05764 | -0.5476 | 1.12956 | -0.56322 | 1.75161 | 1.25111 | 1.65893 | 1.360139 | 2.75175 | 2.74452 | 0.02843 | 0.02079 | 0.12598 | 0.10939 | 7 | 2.93229 | 0.55262 | 0.54292 | 0.545783 | Significant | Up in Mtub | ALDH7A1 |
| 1919 | G1SDJ0 | #N/A | #N/A | RABBIT | #N/A | -2.13535 | 0.17065 | #N/A | #N/A | #N/A | 0.37745 | #N/A | #N/A | #N/A | 1.359797 | 0.6809 | 1.06431 | 0.61943 | 0.34813 | 0.45663 | 0.37235 | 1 | 2.93229 | 0.55262 | 2.65882 | 1.088236 | Not Significant | NS | #N/A |
| 2006 | G1TJ15 | #N/A | #N/A | RABBIT | #N/A | #N/A | #N/A | -0.70719 | #N/A | #N/A | #N/A | #N/A | #N/A | 0.64955 | 1.356742 | #N/A | 1.29054 | #N/A | 0.28921 | #N/A | 0.33963 | 0 | 2.93229 | 0.55262 | #N/A | 0.552617 | Not Significant | NS | #N/A |
| 1551 | G1SDG8 | Nucleolar and coiled-body phosphoprotein 1 OS=O | NOLC1 | RABBIT | -1.46538 | #N/A | #N/A | -0.03177 | -0.03925 | #N/A | #N/A | #N/A | #N/A | 0.83583 | 1.347963 | 1.41406 | 1.50097 | 0.29293 | 0.19445 | 0.32101 | 0.27969 | 2 | 2.93229 | 0.55262 | 0.68153 | 0.604888 | Not Significant | NS | #N/A |
| 1419 | A0A5F9C9E7 | THO complex subunit 2 OS=Oryctolagus cuniculus | THOC2 | RABBIT | -1.13156 | #N/A | #N/A | #N/A | #N/A | #N/A | 0.1233 | -0.06437 | 1.36232 | -0.55997 | 1.346883 | 1.47419 | 1.54135 | 0.23687 | 0.17472 | 0.29252 | 0.26652 | 3 | 2.93229 | 0.55262 | 0.6678 | 0.610864 | Not Significant | NS | #N/A |
| 770 | A0A5F9DSA1 | Pyruvate dehydrogenase E1 component subunit alp | PDHA1 | RABBIT | -1.28157 | -1.99151 | 0.27154 | -0.25456 | 0.08961 | 1.06549 | -0.24384 | 1.10016 | 1.74621 | -0.10089 | 1.346725 | 2.33212 | 2.4453 | 0.048 | 0.03264 | 0.14885 | 0.12548 | 8 | 2.93229 | 0.55262 | 0.83368 | 0.758289 | Significant | Up in Mtub | PDHA1 |
| 2058 | G1SGR9 | #N/A | GTF2I | RABBIT | #N/A | #N/A | #N/A | -0.70053 | #N/A | #N/A | #N/A | #N/A | #N/A | 0.64289 | 1.343428 | #N/A | 1.27787 | #N/A | 0.29307 | #N/A | 0.34299 | 0 | 2.93229 | 0.55262 | #N/A | 0.552617 | Not Significant | NS | #N/A |
| 1270 | G1TME7 | Cystatin-B OS=Oryctolagus cuniculus OX=9986 GN | CSTB | RABBIT | -0.04722 | -0.99439 | -0.81544 | 0.18319 | -0.73941 | -0.94934 | 2.56944 | -0.00548 | 2.27745 | 0.39173 | 1.339413 | 1.87145 | 2.03283 | 0.09819 | 0.06708 | 0.18746 | 0.1709 | 8 | 2.93229 | 0.55262 | 1.2806 | 1.085339 | Not Significant | NS | #N/A |
| 859 | A0A5F9DLR9 | Four and a half LIM domains 1 OS=Oryctolagus cuni | FHL1 | RABBIT | -1.84608 | -1.27808 | -0.94498 | -0.50723 | -0.14461 | 1.11132 | 0.13123 | 0.09466 | 0.47141 | 0.15372 | 1.336662 | 3.78667 | 3.44583 | 0.00534 | 0.00552 | 0.07293 | 0.06816 | 8 | 2.93229 | 0.55262 | 0.31151 | 0.376179 | Significant | Up in Mtub | FHL1 |
| 1822 | G1TDU6 | #N/A | NEB | RABBIT | #N/A | -0.59941 | #N/A | #N/A | #N/A | #N/A | #N/A | 0.73286 | #N/A | #N/A | 1.332269 | #N/A | 1.26726 | #N/A | 0.29635 | #N/A | 0.34518 | 0 | 2.93229 | 0.55262 | #N/A | 0.552617 | Not Significant | NS | #N/A |
| 1183 | G1TRV3 | Large ribosomal subunit protein uL22 OS=Oryctolag | #N/A | RABBIT | -4.77656 | -1.48976 | 0.23868 | -0.19955 | 0.22216 | 0.22591 | -0.2467 | 0.37007 | -0.06803 | 0.37083 | 1.331421 | 1.39284 | 1.5605 | 0.20115 | 0.1471 | 0.2713 | 0.24654 | 8 | 2.93229 | 0.55262 | 2.28437 | 1.819873 | Not Significant | NS | #N/A |
| 2298 | G1TWP8 | #N/A | AK5 | RABBIT | #N/A | #N/A | #N/A | -0.16575 | -1.59847 | -0.67317 | #N/A | 1.81482 | 0.10811 | 0.54591 | 1.331027 | 1.4864 | 1.66639 | 0.21137 | 0.13999 | 0.27499 | 0.23907 | 4 | 2.93229 | 0.55262 | 1.06916 | 0.850669 | Not Significant | NS | #N/A |
| 718 | A0A5F9D693 | NEDD8-activating enzyme E1 catalytic subunit OS= | UBA3 | RABBIT | -1.20958 | -0.86992 | -0.72378 | 0.50215 | -1.30844 | 0.52911 | 0.24803 | 0.02505 | 2.32974 | -0.08942 | 1.330415 | 2.42325 | 2.51485 | 0.04164 | 0.02886 | 0.14427 | 0.12009 | 8 | 2.93229 | 0.55262 | 0.75356 | 0.699664 | Significant | Up in Mtub | UBA3 |
| 2435 | A0A5F9DNK1 | #N/A | NIN | RABBIT | #N/A | #N/A | -0.57961 | #N/A | #N/A | 0.75026 | #N/A | #N/A | #N/A | #N/A | 1.329871 | #N/A | 1.26498 | #N/A | 0.29706 | #N/A | 0.34543 | 0 | 2.93229 | 0.55262 | #N/A | 0.552617 | Not Significant | NS | #N/A |
| 2495 | G1THL2 | #N/A | #N/A | RABBIT | #N/A | #N/A | #N/A | -0.18944 | #N/A | #N/A | 2.3445 | -0.06437 | #N/A | #N/A | 1.329508 | 0.63731 | 1.01231 | 0.63878 | 0.36956 | 0.46486 | 0.38394 | 1 | 2.93229 | 0.55262 | 2.90131 | 1.149901 | Not Significant | NS | #N/A |
| 2038 | A5U5I9 | #N/A | #N/A | MT | #N/A | -1.46029 | #N/A | #N/A | -0.28774 | #N/A | 0.27436 | #N/A | #N/A | 0.63493 | 1.328663 | 2.16617 | 1.91559 | 0.16265 | 0.11436 | 0.24194 | 0.21343 | 2 | 2.93229 | 0.55262 | 0.37622 | 0.48109 | Not Significant | NS | #N/A |
| 702 | G1SU04 | CYFIP-related Rac1 interactor A OS=Oryctolagus cu | CYRIA | RABBIT | -3.65901 | -2.5151 | -0.20412 | 0.9904 | -0.00892 | 1.32291 | -2.14211 | -0.06883 | 1.0847 | 1.03705 | 1.326093 | 1.23117 | 1.39146 | 0.25322 | 0.19175 | 0.30054 | 0.27744 | 8 | 2.93229 | 0.55262 | 2.90035 | 2.270635 | Not Significant | NS | #N/A |
| 1699 | G1TLH1 | #N/A | GSTM2 | RABBIT | #N/A | 0.50028 | -2.64319 | -0.13467 | -1.76717 | 0.47956 | #N/A | -0.16363 | 0.85089 | 0.07703 | 1.32215 | 1.74516 | 1.91584 | 0.13157 | 0.08788 | 0.21567 | 0.1893 | 6 | 2.93229 | 0.55262 | 1.14795 | 0.952514 | Not Significant | NS | #N/A |
| 88 | G1SF26 | Clathrin heavy chain OS=Oryctolagus cuniculus OX | CLTCL1 | RABBIT | -3.75924 | -0.36795 | -0.11888 | 0.51219 | -0.27323 | 0.78057 | 1.13532 | -0.21679 | 0.74943 | 0.15242 | 1.321615 | 1.66613 | 1.83323 | 0.13425 | 0.09411 | 0.21787 | 0.19607 | 8 | 2.93229 | 0.55262 | 1.57302 | 1.299322 | Not Significant | NS | #N/A |
| 1784 | A0A5F9CSA7 | #N/A | WDR37 | RABBIT | #N/A | -0.63735 | -2.86364 | 1.07666 | -3.20171 | 0.59816 | -1.76395 | 0.32455 | 0 | 0.4034 | 1.318942 | 1.30859 | 1.4845 | 0.232 | 0.1687 | 0.29043 | 0.26263 | 7 | 2.93229 | 0.55262 | 2.25754 | 1.754197 | Not Significant | NS | #N/A |
| 77 | P68135 | Actin, alpha skeletal muscle OS=Oryctolagus cunic | ACTA1 AC | RABBIT | -1.28862 | #N/A | -0.50268 | #N/A | -0.47437 | #N/A | 0.46099 | 0.41819 | #N/A | 0.80217 | 1.315671 | 4.4876 | 2.90279 | 0.01093 | 0.02314 | 0.09732 | 0.11217 | 4 | 2.93229 | 0.55262 | 0.12893 | 0.308146 | Significant | Up in Mtub | ACTA1 ACTA |
| 1960 | G1T9D1 | #N/A | TCP11L1 | RABBIT | #N/A | -1.29551 | 0.22859 | -1.59832 | #N/A | -0.17853 | 0.71093 | -0.03446 | 1.66488 | -0.0299 | 1.314995 | 2.12238 | 2.20823 | 0.07803 | 0.05482 | 0.17131 | 0.1546 | 6 | 2.93229 | 0.55262 | 0.71979 | 0.664909 | Not Significant | NS | #N/A |
| 1205 | A0A5F9CPM7 | RNA polymerase-associated protein LEO1 OS=Oryc | LEO1 | RABBIT | -0.58617 | #N/A | #N/A | #N/A | -0.94864 | #N/A | 0.87449 | 0.53347 | -0.60191 | 1.37844 | 1.313532 | 2.05246 | 2.0473 | 0.10938 | 0.08024 | 0.19697 | 0.1834 | 4 | 2.93229 | 0.55262 | 0.5461 | 0.548857 | Not Significant | NS | #N/A |
| 1165 | G1U460 | methylmalonate-semialdehyde dehydrogenase (Co | ALDH6A1 | RABBIT | -1.65206 | -1.67164 | 0.22028 | -0.10727 | -3.14519 | 1.49968 | -3.55957 | 1.12283 | 0.94337 | 0.20198 | 1.312833 | 1.18618 | 1.34293 | 0.26958 | 0.20651 | 0.30771 | 0.28716 | 8 | 2.93229 | 0.55262 | 3.06236 | 2.38919 | Not Significant | NS | #N/A |
| 1623 | G1STE5 | APC membrane recruitment protein 2 OS=Oryctolag | AMER2 | RABBIT | #N/A | -0.55662 | 0.1292 | #N/A | #N/A | 4.56306 | -0.01566 | 0.58633 | 0 | 0.3563 | 1.311717 | 0.89046 | 1.06718 | 0.41401 | 0.31729 | 0.37693 | 0.35556 | 5 | 2.93229 | 0.55262 | 3.09992 | 2.15827 | Not Significant | NS | #N/A |
| 1404 | G1TCS3 | ribulose-phosphate 3-epimerase OS=Oryctolagus c | #N/A | RABBIT | -0.99205 | -0.65473 | -0.40149 | -0.23606 | #N/A | #N/A | 0.37511 | 0.11406 | 1.46668 | 1.00345 | 1.310908 | 3.77428 | 3.16327 | 0.00924 | 0.0116 | 0.09144 | 0.09305 | 6 | 2.93229 | 0.55262 | 0.24127 | 0.34348 | Significant | Up in Mtub | #N/A |
| 754 | G1SQC0 | CB1 cannabinoid receptor-interacting protein 1 OS= | CNRIP1 | RABBIT | -2.14244 | -1.40897 | -0.15768 | 0.28142 | -0.94074 | 0.76174 | -0.36573 | 0.26396 | 0.91398 | 0.61129 | 1.310729 | 2.67961 | 2.7074 | 0.02794 | 0.02049 | 0.12572 | 0.10939 | 8 | 2.93229 | 0.55262 | 0.59817 | 0.585951 | Significant | Up in Mtub | CNRIP1 |
| 1602 | G1TPD4 | Histone-lysine N-methyltransferase SETD7 OS=Ory | SETD7 | RABBIT | #N/A | -0.39031 | #N/A | -0.05764 | -0.18144 | 1.98411 | #N/A | -0.51709 | 1.68117 | 1.23635 | 1.30593 | 1.95891 | 2.06664 | 0.10744 | 0.0729 | 0.1968 | 0.17581 | 5 | 2.93229 | 0.55262 | 0.76189 | 0.684529 | Not Significant | NS | #N/A |
| 1995 | G1TSL1 | #N/A | IDH3B | RABBIT | #N/A | #N/A | -0.94687 | -0.89879 | #N/A | 0.81346 | #N/A | 0.71658 | 0.23708 | -0.23708 | 1.305341 | 3.59406 | 2.60327 | 0.02288 | 0.03556 | 0.11776 | 0.12808 | 4 | 2.93229 | 0.55262 | 0.17588 | 0.335236 | Significant | Up in Mtub | IDH3B |
| 669 | G1SL03 | Inosine triphosphate pyrophosphatase OS=Oryctol | ITPA | RABBIT | -1.33863 | -0.94607 | -0.49866 | 0.21505 | -0.30084 | 1.01998 | -0.27269 | 0.26865 | 2.16879 | 0.47121 | 1.305019 | 2.6459 | 2.67892 | 0.02944 | 0.02155 | 0.12737 | 0.11031 | 8 | 2.93229 | 0.55262 | 0.60817 | 0.593271 | Significant | Up in Mtub | ITPA |
| 717 | G1SJZ4 | Obg-like ATPase 1 OS=Oryctolagus cuniculus OX=9 | OLA1 | RABBIT | -1.77176 | -1.87782 | 0.17457 | 0.27933 | -0.88635 | 1.55842 | -0.00392 | -0.4183 | 0.8821 | 0.42138 | 1.30434 | 2.27129 | 2.37893 | 0.05278 | 0.0367 | 0.15283 | 0.12876 | 8 | 2.93229 | 0.55262 | 0.82447 | 0.751553 | Significant | Up in Mtub | OLA1 |
| 368 | A0A5F9CA20 | Dynamin 1 OS=Oryctolagus cuniculus OX=9986 GN | DNM1 | RABBIT | -2.74862 | -1.486 | -0.00162 | 0.11463 | -1.02414 | 0.87189 | -0.60636 | 0.35752 | 0.36491 | 0.37759 | 1.302259 | 2.25201 | 2.36193 | 0.0544 | 0.03782 | 0.15398 | 0.12938 | 8 | 2.93229 | 0.55262 | 0.83598 | 0.759976 | Significant | Up in Mtub | DNM1 |
| 1535 | A0A5F9CPV7 | Mitochondrial import receptor subunit TOM7 homol | #N/A | RABBIT | -2.28165 | -1.46462 | -0.25635 | 0.11004 | -1.2639 | 0 | #N/A | 0.00754 | 0.92915 | 0.14634 | 1.302053 | 2.47649 | 2.51403 | 0.04243 | 0.03085 | 0.14529 | 0.12309 | 7 | 2.93229 | 0.55262 | 0.61429 | 0.596079 | Significant | Up in Mtub | #N/A |
| 2161 | G1SUH8 | #N/A | KRT2 | RABBIT | #N/A | #N/A | -0.37497 | -0.67895 | 0.71277 | 3.55948 | #N/A | #N/A | -0.00239 | 0.00239 | 1.300211 | 1.03236 | 1.25631 | 0.36023 | 0.24968 | 0.35186 | 0.31705 | 4 | 2.93229 | 0.55262 | 2.37936 | 1.606666 | Not Significant | NS | #N/A |
| 2736 | A0A5F9CVC9 | #N/A | #N/A | RABBIT | #N/A | #N/A | #N/A | -0.05764 | #N/A | 3.42129 | #N/A | #N/A | #N/A | -0.9376 | 1.299488 | 0.34424 | 0.63094 | 0.78894 | 0.56289 | 0.51029 | 0.46668 | 1 | 2.93229 | 0.55262 | 9.49996 | 2.827971 | Not Significant | NS | #N/A |
| 411 | G1TAP1 | Inorganic pyrophosphatase OS=Oryctolagus cunicu | PPA1 | RABBIT | -1.42533 | -0.61725 | -0.85646 | 0.08873 | -0.54107 | 0.86411 | -0.14637 | 0.45277 | 0.89905 | 1.06588 | 1.297364 | 3.95537 | 3.49217 | 0.0042 | 0.00508 | 0.06755 | 0.06702 | 8 | 2.93229 | 0.55262 | 0.26896 | 0.345044 | Significant | Up in Mtub | PPA1 |
| 2727 | A0A5F9CL29 | #N/A | #N/A | RABBIT | #N/A | #N/A | #N/A | -0.91607 | #N/A | 0.75651 | #N/A | #N/A | 0 | #N/A | 1.29433 | 1.9756 | 1.51773 | 0.2983 | 0.20489 | 0.32334 | 0.28599 | 1 | 2.93229 | 0.55262 | 0.28616 | 0.484854 | Not Significant | NS | #N/A |
| 461 | G1U0Q7 | Septin OS=Oryctolagus cuniculus OX=9986 GN=SE | SEPTIN2 | RABBIT | -0.46085 | -0.89279 | -0.1393 | -0.47443 | -0.98426 | 0.30995 | 1.41599 | 0.38649 | 0.8062 | 0.59868 | 1.293787 | 5.14469 | 3.98194 | 0.00088 | 0.00218 | 0.04345 | 0.05019 | 8 | 2.93229 | 0.55262 | 0.15811 | 0.263922 | Significant | Up in Mtub | SEPTIN2 |
| 596 | G1U2X0 | Carbonyl reductase (NADPH) OS=Oryctolagus cuni | #N/A | RABBIT | -1.92217 | -1.40606 | -0.25808 | 0.39137 | -1.51433 | 0.12172 | 0.13515 | -0.18608 | 0.79774 | 0.89111 | 1.29378 | 2.6895 | 2.7058 | 0.02752 | 0.02054 | 0.12496 | 0.10939 | 8 | 2.93229 | 0.55262 | 0.57852 | 0.571572 | Significant | Up in Mtub | #N/A |
| 1608 | G1SMM5 | DnaJ heat shock protein family (Hsp40) member A2 | DNAJA2 | RABBIT | -3.09761 | -1.88834 | 0.14971 | 0.40053 | -2.29118 | 0.51243 | -1.27227 | 0.43317 | 0.04707 | 0.02094 | 1.293647 | 1.69855 | 1.85993 | 0.12783 | 0.08999 | 0.21348 | 0.1912 | 8 | 2.93229 | 0.55262 | 1.45016 | 1.209421 | Not Significant | NS | #N/A |
| 781 | A0A5F9DI64 | Protein phosphatase 1A OS=Oryctolagus cuniculus | PPM1A | RABBIT | -1.64073 | 0.43027 | -1.75603 | -0.09225 | -0.40426 | 0.8856 | -0.30847 | -0.02976 | 1.6479 | 0.80591 | 1.292835 | 2.32609 | 2.42024 | 0.04846 | 0.03412 | 0.14898 | 0.1262 | 8 | 2.93229 | 0.55262 | 0.77228 | 0.71336 | Significant | Up in Mtub | PPM1A |
| 1275 | G1T983 | Heme-binding protein 1 OS=Oryctolagus cuniculus | HEBP1 | RABBIT | -2.83917 | -0.19532 | -0.67857 | 0.40809 | -1.15956 | 0.58586 | -1.62569 | 0.32878 | 0.9589 | 1.74375 | 1.29123 | 1.6435 | 1.80634 | 0.1389 | 0.09844 | 0.22123 | 0.19787 | 8 | 2.93229 | 0.55262 | 1.54315 | 1.277468 | Not Significant | NS | #N/A |
| 2309 | G1TH64 | #N/A | #N/A | RABBIT | #N/A | #N/A | -0.56524 | -1.69129 | #N/A | #N/A | 0.14823 | #N/A | 0 | 0.33995 | 1.29099 | 2.94436 | 2.26507 | 0.0603 | 0.0646 | 0.15554 | 0.16683 | 3 | 2.93229 | 0.55262 | 0.2307 | 0.389821 | Not Significant | NS | #N/A |
| 2644 | G1SYY4 | #N/A | COMMD3 | RABBIT | #N/A | #N/A | #N/A | -1.01768 | #N/A | -0.08303 | 0.61575 | 0.90946 | -0.15965 | 0.08303 | 1.290792 | 2.52285 | 1.9649 | 0.06516 | 0.09057 | 0.15932 | 0.19171 | 4 | 2.93229 | 0.55262 | 0.21815 | 0.359624 | Not Significant | NS | #N/A |
| 495 | A0A5F9CYT7 | Sodium/potassium-transporting ATPase subunit alp | ATP1A3 | RABBIT | -2.97472 | -2.55411 | 0.56658 | -2.02745 | 0.97512 | 0.01319 | -0.1847 | 0.46834 | -0.01319 | 0.13616 | 1.286874 | 1.55164 | 1.71562 | 0.15935 | 0.1144 | 0.23848 | 0.21343 | 8 | 2.93229 | 0.55262 | 1.71961 | 1.406599 | Not Significant | NS | #N/A |
| 2805 | P33477 | #N/A | ANXA11 AI | RABBIT | #N/A | #N/A | #N/A | #N/A | -0.12633 | 0.91084 | 4.12725 | -0.06437 | #N/A | -0.34829 | 1.282682 | 0.55891 | 0.73995 | 0.61523 | 0.48757 | 0.45457 | 0.43895 | 3 | 2.93229 | 0.55262 | 4.21353 | 2.403969 | Not Significant | NS | #N/A |
| 1793 | G1U4G2 | #N/A | #N/A | RABBIT | #N/A | -0.29282 | 0.17065 | #N/A | -0.85863 | #N/A | #N/A | 1.10565 | 0.80504 | #N/A | 1.282274 | 3.20392 | 2.30813 | 0.04918 | 0.06091 | 0.14906 | 0.16242 | 3 | 2.93229 | 0.55262 | 0.19221 | 0.370358 | Not Significant | NS | #N/A |

| | | | | | | | | | | | | | | | | | | | | | | | | | | | | | |
|---|---|---|---|---|---|---|---|---|---|---|---|---|---|---|---|---|---|---|---|---|---|---|---|---|---|---|---|---|---|
| 1754 | A0A5F9CGB5 | #N/A | PSPC1 | RABBIT | #N/A | -2.44142 | -0.82022 | 1.55627 | -1.66051 | 0.36313 | -0.60726 | 1.08597 | 0 | 1.35174 | 1.280185 | 1.48728 | 1.65883 | 0.18054 | 0.12835 | 0.2553 | 0.22682 | 7 | 2.93229 | 0.55262 | 1.64644 | 1.323514 | Not Significant | NS | #N/A |
| 878 | A0A5F9CQY4 | Cytohesin-2 OS=Oryctolagus cuniculus OX=9986 G | CYTH2 | RABBIT | -0.16702 | #N/A | -1.4441 | -0.05764 | 1.59896 | 1.56527 | #N/A | #N/A | 2.56836 | -0.35274 | 1.277745 | 1.24158 | 1.44054 | 0.26946 | 0.18799 | 0.30771 | 0.2762 | 5 | 2.93229 | 0.55262 | 1.8156 | 1.348722 | Not Significant | NS | #N/A |
| 1207 | G1T6F6 | Galectin OS=Oryctolagus cuniculus OX=9986 GN=L | LGALSL | RABBIT | -2.25948 | -3.3969 | -0.08867 | 0.60627 | -0.03167 | 0.87996 | -0.14858 | 0.11626 | 0.34872 | 0.01829 | 1.277019 | 1.62769 | 1.78867 | 0.14224 | 0.10138 | 0.22477 | 0.20137 | 8 | 2.93229 | 0.55262 | 1.53883 | 1.274307 | Not Significant | NS | #N/A |
| 1412 | Q28749 | Fibronectin (Fragment) OS=Oryctolagus cuniculus ( | FN1 | RABBIT | -0.18667 | -1.2052 | 0.17065 | #N/A | 1.05583 | 3.68683 | 2.74227 | 0.63748 | -0.76569 | -0.12348 | 1.276829 | 1.21734 | 1.38593 | 0.26292 | 0.19609 | 0.30559 | 0.28064 | 7 | 2.93229 | 0.55262 | 2.44472 | 1.886115 | Not Significant | NS | #N/A |
| 1652 | G1U0Q2 | Small ribosomal subunit protein uS19 OS=Oryctola | RPS15 | RABBIT | -2.706 | -1.34452 | -0.09032 | -0.57281 | 1.53044 | 0.26098 | -1.13925 | 1.5742 | 1.1247 | 1.36343 | 1.273454 | 1.48425 | 1.64681 | 0.17604 | 0.12801 | 0.25448 | 0.22682 | 8 | 2.93229 | 0.55262 | 1.84031 | 1.494918 | Not Significant | NS | #N/A |
| 2637 | A0A5F9DJB4 | #N/A | TARBP1 | RABBIT | #N/A | #N/A | #N/A | -0.66503 | #N/A | #N/A | 0.60739 | #N/A | #N/A | #N/A | 1.272427 | #N/A | 1.21034 | #N/A | 0.31461 | #N/A | 0.35371 | 0 | 2.93229 | 0.55262 | #N/A | 0.552617 | Not Significant | NS | #N/A |
| 1242 | G1TYR8 | Cysteine protease OS=Oryctolagus cuniculus OX=9 | ATG4B | RABBIT | #N/A | -1.80926 | #N/A | 0.53997 | -1.27108 | 1.2853 | 0.14584 | -0.21021 | 1.28395 | -0.37855 | 1.272056 | 1.79744 | 1.93273 | 0.12239 | 0.08554 | 0.20979 | 0.18812 | 6 | 2.93229 | 0.55262 | 0.93908 | 0.812214 | Not Significant | NS | #N/A |
| 1302 | G1T3S1 | Proteasome 26S subunit, ATPase 6 OS=Oryctolagus | PSMC6 | RABBIT | #N/A | -2.1023 | -0.79679 | 0.26791 | -1.56284 | -0.4394 | 0 | 0.37719 | 0.0372 | 1.13833 | 1.271172 | 2.34885 | 2.403 | 0.05117 | 0.03728 | 0.15026 | 0.12876 | 7 | 2.93229 | 0.55262 | 0.65086 | 0.621853 | Significant | Up in Mtub | PSMC6 |
| 2473 | P41975 | #N/A | SOD3 | RABBIT | #N/A | #N/A | -1.36031 | #N/A | #N/A | #N/A | 0.30051 | 1.23334 | -1.89239 | 0 | 1.270674 | 0.86656 | 1.06304 | 0.44994 | 0.32911 | 0.39114 | 0.3606 | 3 | 2.93229 | 0.55262 | 1.72015 | 1.143044 | Not Significant | NS | #N/A |
| 1786 | A0A5F9DQ17 | #N/A | CMAS | RABBIT | #N/A | -2.52768 | -0.1263 | 0.72803 | -1.10015 | -0.085 | 0.07463 | 1.31211 | 0 | 1.26823 | 1.270519 | 1.78532 | 1.93675 | 0.11738 | 0.08172 | 0.20536 | 0.18476 | 7 | 2.93229 | 0.55262 | 1.12543 | 0.956322 | Not Significant | NS | #N/A |
| 545 | U3KMP1 | Ras-related protein Rab-10 OS=Oryctolagus cunicu | RAB10 | RABBIT | -1.62982 | -0.16691 | 0.00826 | -0.6027 | -0.23179 | 1.29942 | 0.16239 | 0.35918 | 1.02581 | 0.88118 | 1.270191 | 3.51054 | 3.22535 | 0.00796 | 0.00814 | 0.08529 | 0.0841 | 8 | 2.93229 | 0.55262 | 0.32729 | 0.387726 | Significant | Up in Mtub | RAB10 |
| 1389 | A0A5F9D985 | Rho GTPase activating protein 44 OS=Oryctolagus c | ARHGAP44 | RABBIT | #N/A | -0.97305 | #N/A | -0.28131 | -0.68976 | #N/A | 0 | 0.10347 | 1.44745 | 0.93077 | 1.268463 | 2.87002 | 2.57696 | 0.03499 | 0.033 | 0.13465 | 0.12595 | 5 | 2.93229 | 0.55262 | 0.33486 | 0.41536 | Significant | Up in Mtub | ARHGAP44 |
| 102 | A0A5F9DFJ9 | Glyceraldehyde-3-phosphate dehydrogenase OS=C | #N/A | RABBIT | -2.24041 | -1.18118 | -0.0761 | 0.32459 | -0.71011 | 1.14775 | -0.48749 | 0.18239 | 0.79837 | 0.81526 | 1.267898 | 2.37545 | 2.45023 | 0.04486 | 0.03236 | 0.14686 | 0.12495 | 8 | 2.93229 | 0.55262 | 0.71223 | 0.669415 | Significant | Up in Mtub | #N/A |
| 1054 | A0A5F9DAC4 | Acyl-protein thioesterase 1 OS=Oryctolagus cunicu | #N/A | RABBIT | -0.03967 | -0.0421 | 0.17065 | #N/A | #N/A | #N/A | #N/A | 0.49537 | #N/A | 2.09615 | 1.266138 | 2.09806 | 1.97304 | 0.1268 | 0.09649 | 0.2129 | 0.19688 | 3 | 2.93229 | 0.55262 | 0.43703 | 0.494163 | Not Significant | NS | #N/A |
| 175 | A0A5F9CSP6 | Neurexin 3 OS=Oryctolagus cuniculus OX=9986 GN | NRXN3 | RABBIT | 0.01166 | -0.89973 | -0.94977 | #N/A | 4.12051 | #N/A | #N/A | #N/A | #N/A | 1.83589 | 1.26522 | 0.47006 | 0.6322 | 0.67037 | 0.55083 | 0.47323 | 0.46295 | 3 | 2.93229 | 0.55262 | 5.79588 | 3.204175 | Not Significant | NS | #N/A |
| 1327 | A0A5F9CX56 | Synaptoporin OS=Oryctolagus cuniculus OX=9986 | SYNPR | RABBIT | -3.47811 | #N/A | 0.88563 | -3.21691 | 0.94903 | 0 | 0.16192 | 1.41997 | -0.85248 | -0.49052 | 1.262866 | 1.08117 | 1.24134 | 0.31546 | 0.243 | 0.33163 | 0.31349 | 7 | 2.93229 | 0.55262 | 3.03192 | 2.299959 | Not Significant | NS | #N/A |
| 734 | P18287 | Apolipoprotein E OS=Oryctolagus cuniculus OX=99 | APOE | RABBIT | -2.38124 | -2.66867 | 0.26329 | -0.62809 | 0.38174 | 0.20782 | -0.09264 | 0.46807 | -0.14028 | 0.83071 | 1.261327 | 1.88115 | 2.0242 | 0.09673 | 0.06808 | 0.1866 | 0.17147 | 8 | 2.93229 | 0.55262 | 1.12395 | 0.970709 | Not Significant | NS | #N/A |
| 1538 | A0A5F9CJN2 | Alsin Rho guanine nucleotide exchange factor ALS2 | ALS2 | RABBIT | -4.54831 | #N/A | 1.90407 | #N/A | #N/A | #N/A | #N/A | -0.06437 | #N/A | #N/A | 1.257755 | 0.22508 | 0.42992 | 0.85906 | 0.68976 | 0.52955 | 0.50986 | 1 | 2.93229 | 0.55262 | 20.8165 | 5.705834 | Not Significant | NS | #N/A |
| 1227 | G1TDB3 | Small ribosomal subunit protein eS25 OS=Oryctola | RPS25 | RABBIT | 1.09447 | 0.91774 | 0.55576 | -4.96515 | -4.72319 | -0.75001 | 0.03781 | 0.05863 | -0.03781 | -0.14052 | 1.257696 | 0.89357 | 1.02385 | 0.39763 | 0.32803 | 0.36962 | 0.36006 | 8 | 2.93229 | 0.55262 | 4.95256 | 3.772395 | Not Significant | NS | #N/A |
| 1243 | G1TL06 | Large ribosomal subunit protein uL3 OS=Oryctolagu | RPL3 | RABBIT | -4.47927 | -2.46882 | 0.6531 | -0.47159 | 0.89544 | -0.27143 | -0.10311 | 0.36418 | 0.10311 | 0.32015 | 1.256805 | 1.22618 | 1.38112 | 0.255 | 0.19482 | 0.30166 | 0.27975 | 8 | 2.93229 | 0.55262 | 2.62644 | 2.070196 | Not Significant | NS | #N/A |
| 1210 | A0A5F9CLT6 | Protein phosphatase 1 regulatory subunit 21 OS=Or | PPP1R21 | RABBIT | -0.62174 | #N/A | #N/A | #N/A | #N/A | #N/A | 0.6334 | #N/A | #N/A | #N/A | 1.255138 | #N/A | 1.19389 | #N/A | 0.3201 | #N/A | 0.35646 | 0 | 2.93229 | 0.55262 | #N/A | 0.552617 | Not Significant | NS | #N/A |
| 258 | G1U9R0 | glutathione transferase OS=Oryctolagus cuniculus | #N/A | RABBIT | -1.67136 | -1.30013 | -0.08961 | 0.02878 | -0.6603 | 0.94847 | -0.08642 | 0.1984 | 1.09368 | 0.42405 | 1.254158 | 3.13808 | 2.988 | 0.01385 | 0.01242 | 0.0978 | 0.09506 | 8 | 2.93229 | 0.55262 | 0.39932 | 0.440436 | Significant | Up in Mtub | #N/A |
| 989 | A0A5F9CCM3 | Prosaposin OS=Oryctolagus cuniculus OX=9986 GN | PSAP | RABBIT | 1.48831 | #N/A | -1.96538 | -0.34809 | -3.32129 | 0.26616 | #N/A | #N/A | 0 | 0.38588 | 1.253958 | 1.01783 | 1.20893 | 0.35545 | 0.26149 | 0.35023 | 0.32441 | 5 | 2.93229 | 0.55262 | 2.60193 | 1.844374 | Not Significant | NS | #N/A |
| 789 | A0A5F9DPC3 | Eukaryotic translation initiation factor 3 subunit H C | EIF3H EIF3 | RABBIT | -0.60293 | -0.49433 | 0.00555 | 0.02671 | -0.18281 | -0.08435 | 1.55916 | 0.95168 | 0.53155 | 2.04285 | 1.249736 | 3.15987 | 2.99812 | 0.0134 | 0.0122 | 0.09752 | 0.09479 | 8 | 2.93229 | 0.55262 | 0.39105 | 0.434389 | Significant | Up in Mtub | EIF3H EIF3S3 |
| 2878 | P9WP19 | #N/A | #N/A | MT | #N/A | #N/A | #N/A | #N/A | -0.63107 | #N/A | #N/A | #N/A | 0.61769 | #N/A | 1.248758 | #N/A | 1.18782 | #N/A | 0.32215 | #N/A | 0.35646 | 0 | 2.93229 | 0.55262 | #N/A | 0.552617 | Not Significant | NS | #N/A |
| 988 | G1U7C5 | Ribosome binding protein 1 OS=Oryctolagus cunicu | RRBP1 | RABBIT | 0.07752 | -2.16619 | #N/A | #N/A | -0.07924 | 0.52586 | #N/A | #N/A | #N/A | #N/A | 1.248494 | 0.86318 | 1.1008 | 0.47902 | 0.32178 | 0.40533 | 0.35646 | 2 | 2.93229 | 0.55262 | 1.56902 | 0.96476 | Not Significant | NS | #N/A |
| 618 | G1TPZ1 | Galectin OS=Oryctolagus cuniculus OX=9986 GN=L | LGALS1 | RABBIT | -2.03924 | -1.93128 | 0.0466 | -0.52345 | 0.26807 | -0.06525 | 0.66199 | 0.94074 | 0.06525 | 0.4515 | 1.246706 | 2.39161 | 2.45379 | 0.04375 | 0.03215 | 0.14581 | 0.12472 | 8 | 2.93229 | 0.55262 | 0.67934 | 0.645349 | Significant | Up in Mtub | LGALS1 |
| 2033 | G1U354 | #N/A | #N/A | RABBIT | #N/A | -1.38468 | -0.4723 | 1.2984 | -1.93945 | 0 | 0.49781 | 0.34131 | -0.14434 | 2.41459 | 1.24638 | 1.531 | 1.69532 | 0.16963 | 0.12108 | 0.24852 | 0.21983 | 7 | 2.93229 | 0.55262 | 1.47278 | 1.20112 | Not Significant | NS | #N/A |
| 1591 | G1TDI0 | Proliferation-associated 2G4 OS=Oryctolagus cunic | PA2G4 | RABBIT | #N/A | -2.20917 | -0.20384 | 0.15805 | -2.00008 | 0.40974 | 0 | 0.05825 | -0.00673 | 0.45011 | 1.246034 | 2.28423 | 2.34172 | 0.05628 | 0.04138 | 0.15432 | 0.13463 | 7 | 2.93229 | 0.55262 | 0.66125 | 0.629181 | Significant | Up in Mtub | PA2G4 |
| 746 | G1SXA5 | Heterogeneous nuclear ribonucleoprotein U like 2 C | HNRNPUL | RABBIT | -2.11403 | -0.97987 | -0.28901 | 0.13899 | -1.12121 | 0.07067 | 0.48155 | 0.53294 | -0.07067 | 0.82571 | 1.241067 | 2.96299 | 2.8648 | 0.01806 | 0.01547 | 0.11098 | 0.09934 | 8 | 2.93229 | 0.55262 | 0.4386 | 0.469182 | Significant | Up in Mtub | HNRNPUL2 |
| 380 | A0A5F9DTD8 | Microtubule-associated protein OS=Oryctolagus cu | MAPT | RABBIT | 0.06478 | 1.8381 | -3.06149 | -0.11076 | -3.41996 | -0.46537 | 0.63762 | 0.72013 | -0.45102 | 1.06344 | 1.238826 | 1.17879 | 1.33007 | 0.27235 | 0.21058 | 0.30973 | 0.29031 | 8 | 2.93229 | 0.55262 | 2.76114 | 2.168763 | Not Significant | NS | #N/A |
| 995 | A0A5S8HX12 | Translationally-controlled tumor protein OS=Oryctc | #N/A | RABBIT | -1.35301 | -0.86788 | -0.78013 | -0.05764 | #N/A | 1.19703 | 0.4863 | 0.12356 | -0.21502 | 0.77301 | 1.237641 | 3.39537 | 3.02805 | 0.01151 | 0.01281 | 0.09732 | 0.09594 | 7 | 2.93229 | 0.55262 | 0.29526 | 0.371237 | Significant | Up in Mtub | #N/A |
| 1045 | A0A5F9C9L9 | Endoplasmic reticulum protein 44 OS=Oryctolagus | ERP44 | RABBIT | #N/A | #N/A | #N/A | -0.5199 | -0.0193 | #N/A | 1.12027 | -0.06437 | 1.84211 | #N/A | 1.235607 | 1.6668 | 1.73782 | 0.19415 | 0.13347 | 0.26481 | 0.2333 | 3 | 2.93229 | 0.55262 | 0.65944 | 0.606639 | Not Significant | NS | #N/A |
| 1213 | A0A5F9CG47 | Coatomer subunit zeta OS=Oryctolagus cuniculus ( | COPZ1 | RABBIT | -2.01436 | #N/A | -0.05905 | -0.05764 | -0.24482 | -0.12278 | 1.03516 | 0.43485 | 0.88849 | 0.96256 | 1.233623 | 2.54355 | 2.52234 | 0.03846 | 0.03041 | 0.1391 | 0.12297 | 7 | 2.93229 | 0.55262 | 0.52272 | 0.531549 | Significant | Up in Mtub | COPZ1 |
| 1782 | A0A5F9D087 | #N/A | #N/A | RABBIT | #N/A | -1.04226 | -0.033 | -0.38531 | -0.88621 | 1.43722 | 0.20365 | 0.47313 | #N/A | 0.4695 | 1.232569 | 3.45512 | 2.93643 | 0.01355 | 0.01671 | 0.09752 | 0.10198 | 6 | 2.93229 | 0.55262 | 0.25452 | 0.352381 | Significant | Up in Mtub | #N/A |
| 346 | A0A5S8I1N0 | Serine/threonine-protein phosphatase 2A 55 kDa re | PPP2R2A | RABBIT | -0.40802 | -0.60862 | -0.63012 | 0.40625 | -0.31604 | 1.3941 | #N/A | 0.23829 | #N/A | 1.12496 | 1.230427 | 3.43224 | 2.87564 | 0.01393 | 0.01845 | 0.09783 | 0.10578 | 6 | 2.93229 | 0.55262 | 0.24097 | 0.343276 | Significant | Up in Mtub | PPP2R2A |
| 940 | G1TM55 | Small ribosomal subunit protein eS6 OS=Oryctolag | RPS6 | RABBIT | -2.46268 | -1.30539 | 0.11271 | -0.25575 | 0.04455 | -0.20499 | 0.15536 | 1.01023 | 1.19375 | 0.11513 | 1.227208 | 2.17729 | 2.27195 | 0.06112 | 0.0443 | 0.15706 | 0.14118 | 8 | 2.93229 | 0.55262 | 0.79423 | 0.729421 | Significant | Up in Mtub | RPS6 |
| 2832 | A0A5F9DQI8 | #N/A | ASAP1 | RABBIT | #N/A | #N/A | #N/A | #N/A | -1.05216 | #N/A | 0.41126 | -0.06437 | #N/A | #N/A | 1.225612 | 2.97545 | 1.50717 | 0.20641 | 0.20744 | 0.27238 | 0.28797 | 1 | 2.93229 | 0.55262 | 0.11311 | 0.440849 | Not Significant | NS | #N/A |
| 1908 | A0A5F9DD77 | #N/A | PALLD | RABBIT | #N/A | -0.51211 | #N/A | #N/A | #N/A | #N/A | #N/A | #N/A | 0.70993 | #N/A | 1.222046 | #N/A | 1.16241 | #N/A | 0.33088 | #N/A | 0.36157 | 0 | 2.93229 | 0.55262 | #N/A | 0.552617 | Not Significant | NS | #N/A |
| 2889 | G1TM42 | #N/A | #N/A | RABBIT | #N/A | #N/A | #N/A | #N/A | -1.05205 | -0.29788 | #N/A | #N/A | 0.29788 | 0.4968 | 1.217651 | 2.55007 | 1.67178 | 0.12548 | 0.15623 | 0.21204 | 0.25357 | 2 | 2.93229 | 0.55262 | 0.171 | 0.397875 | Not Significant | NS | #N/A |
| 1527 | G1U0G4 | Protein cereblon OS=Oryctolagus cuniculus OX=99 | CRBN | RABBIT | #N/A | #N/A | #N/A | -1.14988 | #N/A | 0.26385 | -0.22329 | 0.15893 | #N/A | #N/A | 1.216375 | 4.10865 | 1.76753 | 0.05445 | 0.13819 | 0.15398 | 0.23716 | 2 | 2.93229 | 0.55262 | 0.06574 | 0.355191 | Not Significant | NS | #N/A |
| 2316 | G1TQP6 | #N/A | #N/A | RABBIT | #N/A | #N/A | -1.0933 | #N/A | #N/A | 0.56688 | -0.12385 | -0.34544 | 0.12385 | 0.37877 | 1.213336 | 3.00684 | 1.9828 | 0.03967 | 0.08822 | 0.14115 | 0.18954 | 4 | 2.93229 | 0.55262 | 0.13569 | 0.312048 | Not Significant | NS | #N/A |
| 2610 | A0A5F9DIW3 | #N/A | SNX6 | RABBIT | #N/A | #N/A | #N/A | -0.95903 | #N/A | 1.50658 | -0.93964 | -1.29047 | 0.90139 | 1.09014 | 1.212633 | 0.86856 | 1.02296 | 0.43409 | 0.34069 | 0.38352 | 0.36807 | 4 | 2.93229 | 0.55262 | 1.62434 | 1.171012 | Not Significant | NS | #N/A |
| 1501 | A0A5F9D4B2 | BCAS3 microtubule associated cell migration factor | BCAS3 | RABBIT | -0.87226 | #N/A | -0.26544 | -1.32675 | -0.18027 | 1.77475 | 0 | 0.14749 | 0.03429 | 0.79599 | 1.211684 | 2.68564 | 2.60185 | 0.03128 | 0.02654 | 0.12952 | 0.11767 | 7 | 2.93229 | 0.55262 | 0.45235 | 0.48195 | Significant | Up in Mtub | BCAS3 |
| 443 | G1SI79 | RRM domain-containing protein OS=Oryctolagus cu | #N/A | RABBIT | -2.39538 | -0.80355 | 0.15094 | 0.31415 | -0.77747 | 0.64105 | -0.03928 | 0.85757 | 0.01971 | 1.05276 | 1.208627 | 2.28039 | 2.3485 | 0.05204 | 0.03872 | 0.15128 | 0.13043 | 8 | 2.93229 | 0.55262 | 0.70227 | 0.662131 | Significant | Up in Mtub | #N/A |
| 2250 | A0A5F9DL11 | #N/A | PADI2 | RABBIT | #N/A | #N/A | -2.08726 | #N/A | #N/A | -4.66818 | #N/A | 0.47514 | 0 | 0.67426 | 1.207566 | 0.42499 | 0.5741 | 0.69949 | 0.58699 | 0.48373 | 0.47237 | 3 | 2.93229 | 0.55262 | 6.45893 | 3.539483 | Not Significant | NS | #N/A |
| 2248 | A0A5F9CBD5 | #N/A | TOP2B | RABBIT | #N/A | #N/A | 0.17065 | #N/A | -1.838 | 0.6528 | -0.41471 | -0.61887 | 0.75356 | 1.47707 | 1.203647 | 1.42956 | 1.56721 | 0.21223 | 0.15602 | 0.27513 | 0.25356 | 5 | 2.93229 | 0.55262 | 1.01273 | 0.842645 | Not Significant | NS | #N/A |
| 910 | G1SVT8 | CaM kinase-like vesicle-associated protein OS=Ory | CAMKV | RABBIT | -2.88435 | -0.9863 | -0.55223 | 1.0745 | -0.67569 | 0.87834 | -1.24604 | 0.92411 | 0.77382 | 0.66231 | 1.203324 | 1.59446 | 1.74398 | 0.1495 | 0.10917 | 0.23045 | 0.20798 | 8 | 2.93229 | 0.55262 | 1.4239 | 1.1902 | Not Significant | NS | #N/A |
| 871 | A0A5F9DU33 | NAD(P)H dehydrogenase [quinone] 1 OS=Oryctolag | NQO1 | RABBIT | -0.2124 | 0.17169 | 1.28125 | #N/A | -0.01338 | #N/A | #N/A | 1.50931 | #N/A | #N/A | 1.202522 | 1.6094 | 1.52258 | 0.2059 | 0.17925 | 0.27238 | 0.26969 | 3 | 2.93229 | 0.55262 | 0.44663 | 0.499018 | Not Significant | NS | #N/A |
| 2568 | A0A5F9D3V9 | #N/A | TBC1D13 | RABBIT | #N/A | #N/A | #N/A | #N/A | -0.88086 | #N/A | 0 | -1.12944 | 1.51194 | 0.89963 | 1.201395 | 0.93529 | 1.10792 | 0.41863 | 0.31078 | 0.3785 | 0.35218 | 3 | 2.93229 | 0.55262 | 1.31999 | 0.940681 | Not Significant | NS | #N/A |
| 872 | G1SUT7 | Septin OS=Oryctolagus cuniculus OX=9986 PE=3 S | #N/A | RABBIT | -1.91581 | -0.82251 | -0.38027 | -0.0202 | -0.28584 | 1.13054 | -0.03744 | 0.78471 | 0.56923 | 0.13351 | 1.201033 | 3.03439 | 2.87982 | 0.0162 | 0.01506 | 0.10623 | 0.09934 | 8 | 2.93229 | 0.55262 | 0.39166 | 0.434831 | Significant | Up in Mtub | #N/A |
| 2218 | A0A5F9CI00 | #N/A | PGM1 | RABBIT | #N/A | #N/A | -0.93026 | #N/A | -0.01338 | 0.72485 | #N/A | #N/A | #N/A | #N/A | 1.196675 | 1.50707 | 1.3563 | 0.37295 | 0.24765 | 0.35712 | 0.31626 | 1 | 2.93229 | 0.55262 | 0.42033 | 0.518976 | Not Significant | NS | #N/A |
| 2874 | U3KPF5 | #N/A | #N/A | RABBIT | #N/A | #N/A | #N/A | #N/A | -0.36398 | #N/A | #N/A | -0.06437 | 1.72262 | #N/A | 1.193113 | 0.77095 | 1.07703 | 0.58188 | 0.34306 | 0.44383 | 0.36928 | 1 | 2.93229 | 0.55262 | 1.59667 | 0.818124 | Not Significant | NS | #N/A |
| 487 | U3KNF1 | LIM and SH3 domain protein 1 OS=Oryctolagus cun | LASP1 | RABBIT | -0.55378 | -0.7954 | -0.5541 | -0.02119 | -0.19852 | 1.21988 | -0.03645 | 0.48041 | 1.06665 | 1.10057 | 1.190811 | 4.32149 | 3.51374 | 0.00254 | 0.0049 | 0.05406 | 0.06702 | 8 | 2.93229 | 0.55262 | 0.18983 | 0.287136 | Significant | Up in Mtub | LASP1 |
| 96 | Q9TTC6 | Peptidyl-prolyl cis-trans isomerase A OS=Oryctolag | PPIA | RABBIT | -1.16374 | -0.91812 | -0.0805 | -0.29301 | -0.1796 | 1.08301 | 0.16622 | 0.37266 | 0.82164 | 0.85263 | 1.186225 | 4.32895 | 3.50964 | 0.00252 | 0.00493 | 0.05406 | 0.06702 | 8 | 2.93229 | 0.55262 | 0.18772 | 0.285593 | Significant | Up in Mtub | PPIA |
| 2133 | G1SVF2 | #N/A | PSMD1 | RABBIT | #N/A | #N/A | -0.48038 | -0.2296 | #N/A | 0 | -0.15263 | 0.05493 | 2.17367 | 2.03218 | 1.176623 | 1.3364 | 1.4805 | 0.23901 | 0.17733 | 0.29367 | 0.26787 | 5 | 2.93229 | 0.55262 | 1.10739 | 0.902313 | Not Significant | NS | #N/A |
| 1025 | G1SP51 | Small ribosomal subunit protein uS15 OS=Oryctola | RPS13 | RABBIT | -3.20372 | -2.6933 | 0.36896 | -0.06139 | -0.09921 | 0.05703 | -0.29257 | 0.22419 | 0.00375 | 0.20192 | 1.176596 | 1.56065 | 1.70679 | 0.15723 | 0.11607 | 0.23659 | 0.21444 | 8 | 2.93229 | 0.55262 | 1.42096 | 1.188049 | Not Significant | NS | #N/A |
| 544 | G1TLP0 | Histone H2B OS=Oryctolagus cuniculus OX=9986 F | #N/A | RABBIT | -0.14249 | -1.06066 | -0.99407 | 0.66053 | -1.25608 | -0.31071 | 1.99022 | 0.59949 | 0.15415 | 0.62624 | 1.17043 | 2.22215 | 2.28531 | 0.05699 | 0.04327 | 0.15449 | 0.13893 | 8 | 2.93229 | 0.55262 | 0.69356 | 0.655756 | Significant | Up in Mtub | #N/A |
| 579 | G1TMV1 | Destrin OS=Oryctolagus cuniculus OX=9986 PE=3 S | #N/A | RABBIT | -2.41168 | -1.28925 | -0.1422 | 0.13242 | -0.87052 | 0.98624 | 0.10866 | 0.16757 | 0.03782 | -0.03782 | 1.168742 | 2.39241 | 2.41644 | 0.04369 | 0.03435 | 0.14581 | 0.1262 | 8 | 2.93229 | 0.55262 | 0.59663 | 0.584826 | Significant | Up in Mtub | #N/A |
| 570 | G1SXR6 | Kinesin-like protein OS=Oryctolagus cuniculus OX= | KIF5C | RABBIT | -2.85096 | -1.99806 | 0.53463 | 0.17645 | -0.883 | -0.04999 | -0.58556 | 0.53238 | 0.04999 | 0.86962 | 1.167479 | 1.70031 | 1.83612 | 0.12749 | 0.09366 | 0.21329 | 0.19583 | 8 | 2.93229 | 0.55262 | 1.17864 | 1.010726 | Not Significant | NS | #N/A |
| 1816 | G1TES6 | #N/A | HSD17B1( | RABBIT | #N/A | 0.19782 | -1.09044 | #N/A | #N/A | -0.06304 | -0.48338 | 1.98812 | 1.61566 | 0.53328 | 1.16444 | 1.34721 | 1.48616 | 0.23574 | 0.17586 | 0.29167 | 0.26731 | 5 | 2.93229 | 0.55262 | 1.06725 | 0.877008 | Not Significant | NS | #N/A |
| 1171 | G1U0J7 | [tau protein] kinase OS=Oryctolagus cuniculus OX= | GSK3A | RABBIT | -2.91739 | -0.83218 | -0.17342 | 0.47425 | -0.84464 | 0.61955 | -0.58211 | 0.2797 | 0.58956 | 0.61472 | 1.162961 | 1.89294 | 2.0078 | 0.09499 | 0.07002 | 0.18619 | 0.17293 | 8 | 2.93229 | 0.55262 | 0.94362 | 0.838742 | Not Significant | NS | #N/A |
| 1857 | A0A5F9D6B2 | #N/A | #N/A | RABBIT | #N/A | #N/A | -0.65691 | -0.17013 | -2.65967 | 0.29789 | -0.97948 | 0.04813 | 0.20637 | 0.42783 | 1.162387 | 1.78737 | 1.88354 | 0.1241 | 0.09253 | 0.21125 | 0.19442 | 6 | 2.93229 | 0.55262 | 0.793 | 0.714087 | Not Significant | NS | #N/A |
| 706 | G1T6B3 | Serine-threonine kinase receptor-associated protei | STRAP | RABBIT | -1.09101 | -1.24475 | -0.25381 | -0.21357 | 0.1204 | 1.69762 | 0.60827 | -0.04762 | 0.88508 | -0.01674 | 1.16187 | 2.77443 | 2.68233 | 0.02413 | 0.02142 | 0.11931 | 0.11031 | 8 | 2.93229 | 0.55262 | 0.43844 | 0.469063 | Significant | Up in Mtub | STRAP |
| 2579 | A0A5F9D0X2 | #N/A | NUB1 | RABBIT | #N/A | #N/A | #N/A | -0.83113 | #N/A | 1.11967 | -0.48282 | -0.33037 | 1.06996 | 0.266 | 1.159622 | 1.4046 | 1.41272 | 0.23283 | 0.20103 | 0.29083 | 0.28391 | 4 | 2.93229 | 0.55262 | 0.568 | 0.56149 | Not Significant | NS | #N/A |
| 1239 | G1SPZ9 | inorganic diphosphatase OS=Oryctolagus cuniculu | PPA2 | RABBIT | 0.17088 | -0.15612 | -0.45689 | -0.62245 | -0.65685 | 1.24066 | -0.15921 | 0.62624 | 1.30431 | 1.05584 | 1.157854 | 3.70631 | 3.20261 | 0.00599 | 0.00848 | 0.07337 | 0.08441 | 8 | 2.93229 | 0.55262 | 0.24398 | 0.326767 | Significant | Up in Mtub | PPA2 |
| 2129 | G1U9R4 | #N/A | APOB | RABBIT | #N/A | #N/A | -1.42267 | -1.67873 | 0.60142 | 1.96755 | -2.18635 | -0.06437 | #N/A | 1.57104 | 1.155297 | 0.90953 | 1.08385 | 0.40478 | 0.31027 | 0.37324 | 0.35209 | 5 | 2.93229 | 0.55262 | 2.76591 | 1.947735 | Not Significant | NS | #N/A |
| 2812 | A0A5F9D609 | #N/A | FXR2 | RABBIT | #N/A | #N/A | #N/A | #N/A | -0.61627 | #N/A | #N/A | 0.53852 | #N/A | #N/A | 1.154787 | #N/A | 1.09844 | #N/A | 0.35393 | #N/A | 0.37605 | 0 | 2.93229 | 0.55262 | #N/A | 0.552617 | Not Significant | NS | #N/A |
| 1375 | G1SGJ4 | L-serine ammonia-lyase OS=Oryctolagus cuniculus | #N/A | RABBIT | -1.65248 | -0.88412 | -0.19852 | 0.31153 | #N/A | 1.32745 | #N/A | -1.99884 | 2.05327 | 0.80917 | 1.15366 | 1.17296 | 1.34064 | 0.28527 | 0.21313 | 0.31704 | 0.29187 | 6 | 2.93229 | 0.55262 | 1.93473 | 1.481011 | Not Significant | NS | #N/A |
| 868 | G1T5V8 | palmitoyl-protein hydrolase OS=Oryctolagus cunicu | LYPLA2 | RABBIT | -1.30702 | -1.27431 | -0.38832 | -0.08075 | 0.08758 | 1.68338 | -0.36036 | -0.04125 | 1.37392 | 0.14678 | 1.153061 | 2.29575 | 2.33483 | 0.05081 | 0.03967 | 0.15026 | 0.13231 | 8 | 2.93229 | 0.55262 | 0.63066 | 0.609726 | Significant | Up in Mtub | LYPLA2 |
| 1488 | A0A5F9DPW3 | Plakophilin 4 OS=Oryctolagus cuniculus OX=9986 ( | PKP4 | RABBIT | -1.27471 | #N/A | -0.70583 | -0.52516 | -0.49507 | 0 | 1.35925 | 0.41508 | 0.05166 | 0.18203 | 1.151795 | 3.54509 | 2.99586 | 0.00941 | 0.01354 | 0.09187 | 0.09774 | 7 | 2.93229 | 0.55262 | 0.23458 | 0.32847 | Significant | Up in Mtub | PKP4 |
| 1356 | A0A5F9DBL2 | Neuron navigator 1 OS=Oryctolagus cuniculus OX= | NAV1 | RABBIT | -1.635 | -1.01112 | 0.03073 | 0.26021 | -0.23254 | 1.29127 | -0.19183 | 0.07556 | 0.60172 | 1.39178 | 1.151244 | 2.4324 | 2.43672 | 0.04105 | 0.03314 | 0.14411 | 0.12595 | 8 | 2.93229 | 0.55262 | 0.56002 | 0.558036 | Significant | Up in Mtub | NAV1 |
| 374 | G1T845 | Vesicle-fusing ATPase OS=Oryctolagus cuniculus O | NSF | RABBIT | -3.74317 | -1.05248 | 0.249 | -0.12745 | -0.79901 | 0.55016 | -1.08953 | 0.00545 | 0.3727 | 0.44399 | 1.151175 | 1.50803 | 1.65177 | 0.16998 | 0.12698 | 0.24879 | 0.22608 | 8 | 2.93229 | 0.55262 | 1.45681 | 1.214287 | Not Significant | NS | #N/A |
| 1156 | G1U1G0 | Large ribosomal subunit protein eL33 OS=Oryctolag | #N/A | RABBIT | -3.45616 | -1.0777 | 0.22179 | -2.41086 | 0.77843 | #N/A | 0.34628 | -0.06437 | 0.20558 | -0.6407 | 1.150597 | 1.25489 | 1.40999 | 0.24979 | 0.18908 | 0.2994 | 0.27623 | 7 | 2.93229 | 0.55262 | 1.8682 | 1.479803 | Not Significant | NS | #N/A |
| 2312 | U3KN16 | #N/A | IMPDH1 IN | RABBIT | #N/A | #N/A | #N/A | -1.01886 | #N/A | #N/A | 0 | 0.13663 | 0.5054 | -0.11672 | 1.150184 | 3.80991 | 1.84762 | 0.03179 | 0.11473 | 0.13025 | 0.21381 | 3 | 2.93229 | 0.55262 | 0.07291 | 0.310026 | Not Significant | NS | #N/A |
| 1026 | G1U9S6 | GTP-binding nuclear protein Ran OS=Oryctolagus cu | #N/A | RABBIT | -2.07543 | -1.14417 | -0.08064 | 0.38492 | -0.78602 | #N/A | 0.2513 | 0.48524 | 0.4929 | #N/A | 1.15008 | 2.00901 | 2.04305 | 0.09128 | 0.07165 | 0.18159 | 0.1745 | 6 | 2.93229 | 0.55262 | 0.61446 | 0.594157 | Not Significant | NS | #N/A |
| 679 | A0A5F9CKU2 | E74 like ETS transcription factor 1 OS=Oryctolagus ( | ELF1 | RABBIT | -1.41477 | -0.25076 | -2.40199 | 0.62617 | -0.53928 | 0.65225 | -0.29383 | 0.22946 | 0.86698 | 0.3117 | 1.149438 | 2.07503 | 2.15752 | 0.07167 | 0.05409 | 0.16449 | 0.15379 | 8 | 2.93229 | 0.55262 | 0.76712 | 0.709583 | Not Significant | NS | #N/A |
| 2824 | A0A5F9CYZ8 | #N/A | #N/A | RABBIT | #N/A | #N/A | #N/A | #N/A | -0.58085 | 0.56746 | #N/A | #N/A | #N/A | #N/A | 1.148308 | #N/A | 1.09227 | #N/A | 0.35624 | #N/A | 0.37732 | 0 | 2.93229 | 0.55262 | #N/A | 0.552617 | Not Significant | NS | #N/A |
| 517 | A0A5F9DRM4 | peptidylprolyl isomerase OS=Oryctolagus cuniculus | FKBP4 | RABBIT | -2.12289 | -1.42692 | 0.91635 | -0.38362 | -1.26094 | 0.4944 | -0.25412 | 0.18975 | 0.35458 | 0.67623 | 1.147774 | 2.10247 | 2.17932 | 0.06867 | 0.05207 | 0.16349 | 0.15166 | 8 | 2.93229 | 0.55262 | 0.74506 | 0.693444 | Not Significant | NS | #N/A |
| 538 | G1SGX3 | Oxysterol-binding protein OS=Oryctolagus cuniculu | OSBP | RABBIT | -0.92569 | -1.87154 | -0.69232 | 0.75473 | -0.01338 | 1.19688 | -0.64107 | 0.9689 | 0.86732 | #N/A | 1.147647 | 1.84755 | 1.95287 | 0.10715 | 0.07957 | 0.1968 | 0.18311 | 7 | 2.93229 | 0.55262 | 0.85746 | 0.76746 | Not Significant | NS | #N/A |
| 2596 | A0A5F9CBI8 | #N/A | SDHB | RABBIT | #N/A | #N/A | #N/A | -0.51289 | #N/A | #N/A | #N/A | 1.26814 | 0 | #N/A | 1.146965 | 1.04436 | 1.19265 | 0.48619 | 0.29997 | 0.40856 | 0.3468 | 1 | 2.93229 | 0.55262 | 0.80409 | 0.616568 | Not Significant | NS | #N/A |
| 783 | A0A5F9DES8 | Laminin subunit gamma 1 OS=Oryctolagus cunicul | LAMC1 | RABBIT | -3.78979 | -4.42113 | -0.16512 | 0.06141 | 1.80533 | -3.87613 | 1.96504 | 0.69375 | -0.11905 | 0.55234 | 1.145051 | 0.73692 | 0.84735 | 0.48222 | 0.41497 | 0.40669 | 0.40833 | 8 | 2.93229 | 0.55262 | 6.03604 | 4.565261 | Not Significant | NS | #N/A |
| 427 | G1T5Z7 | mitogen-activated protein kinase OS=Oryctolagus c | MAPK3 | RABBIT | #N/A | -0.73295 | -1.07294 | 0.05616 | -0.81928 | 1.41013 | -0.5131 | -0.06437 | 0.47608 | 1.19929 | 1.143858 | 2.45549 | 2.40424 | 0.04375 | 0.0372 | 0.14581 | 0.12876 | 7 | 2.93229 | 0.55262 | 0.48223 | 0.503011 | Significant | Up in Mtub | MAPK3 |
| 1918 | A0A5F9CBU1 | #N/A | MBNL1 | RABBIT | #N/A | #N/A | #N/A | -0.8541 | #N/A | 1.03736 | -0.13284 | 0.53169 | 0.11317 | -0.11317 | 1.141343 | 2.09479 | 1.69804 | 0.10426 | 0.13373 | 0.19372 | 0.23331 | 4 | 2.93229 | 0.55262 | 0.24738 | 0.376494 | Not Significant | NS | #N/A |
| 620 | G1SLC2 | Eukaryotic translation initiation factor 3 subunit F O | EIF3F EIF3 | RABBIT | -0.69554 | -3.20217 | -1.25994 | 0.26415 | -0.02057 | -0.80682 | 0.70027 | 0.33852 | 0.00718 | 0.54983 | 1.140613 | 1.70037 | 1.8298 | 0.12748 | 0.09466 | 0.21329 | 0.19648 | 8 | 2.93229 | 0.55262 | 1.12494 | 0.971428 | Not Significant | NS | #N/A |
| 381 | A0A5F9DRI8 | RAB6B, member RAS oncogene family OS=Oryctola | RAB6B | RABBIT | 1.78889 | 2.14352 | -1.69255 | -2.21484 | -2.13619 | 3.05475 | #N/A | -0.06437 | 2.09643 | -2.22231 | 1.138361 | 0.7498 | 0.87363 | 0.47781 | 0.40295 | 0.40468 | 0.40266 | 7 | 2.93229 | 0.55262 | 5.12214 | 3.773087 | Not Significant | NS | #N/A |
| 247 | A0A5F9C100 | Septin OS=Oryctolagus cuniculus OX=9986 GN=SE | SEPTIN6 | RABBIT | -1.44741 | -1.77739 | -0.16858 | 0.2017 | -0.27272 | 0.8526 | -0.26844 | 0.19796 | 0.59545 | 0.84619 | 1.137629 | 2.57065 | 2.52741 | 0.03309 | 0.02822 | 0.13286 | 0.11898 | 8 | 2.93229 | 0.55262 | 0.48961 | 0.506513 | Significant | Up in Mtub | SEPTIN6 |
| 2202 | G1TGS9 | #N/A | #N/A | RABBIT | #N/A | #N/A | -0.49478 | -1.06642 | #N/A | 0.83625 | #N/A | 0.23347 | #N/A | 0 | 1.137174 | 2.94772 | 2.06625 | 0.06014 | 0.08486 | 0.15554 | 0.18773 | 3 | 2.93229 | 0.55262 | 0.17859 | 0.36347 | Not Significant | NS | #N/A |
| 1397 | A0A5F9CR59 | Kinesin-like protein OS=Oryctolagus cuniculus OX= | KIF3A | RABBIT | #N/A | -0.30799 | #N/A | -0.1691 | #N/A | 1.64444 | #N/A | #N/A | 0 | 1.05063 | 1.136904 | 1.82537 | 1.74639 | 0.16543 | 0.13191 | 0.24483 | 0.23149 | 3 | 2.93229 | 0.55262 | 0.46551 | 0.508564 | Not Significant | NS | #N/A |
| 1580 | G1SEP2 | Gigaxonin OS=Oryctolagus cuniculus OX=9986 GN= | GAN | RABBIT | -0.24046 | -1.43572 | 0.17065 | -0.16676 | 0.95357 | #N/A | -0.13512 | 0.87156 | 2.14969 | 1.08649 | 1.136896 | 1.89211 | 1.98552 | 0.10036 | 0.07537 | 0.18996 | 0.17973 | 7 | 2.93229 | 0.55262 | 0.8023 | 0.728585 | Not Significant | NS | #N/A |
| 946 | G1SYQ5 | Coatomer subunit delta OS=Oryctolagus cuniculus | #N/A | RABBIT | -1.35425 | -0.15638 | -0.92664 | -0.07274 | -0.52998 | 1.14115 | 0.33684 | 0.4631 | 0.68434 | 0.0151 | 1.136106 | 3.72626 | 3.18404 | 0.00582 | 0.00876 | 0.07337 | 0.0849 | 8 | 2.93229 | 0.55262 | 0.2324 | 0.318287 | Significant | Up in Mtub | #N/A |

| | | | | | | | | | | | | | | | | | | | | | | | | | | | | | |
|---|---|---|---|---|---|---|---|---|---|---|---|---|---|---|---|---|---|---|---|---|---|---|---|---|---|---|---|---|---|
| 55 | G1U466 | Tubulin alpha chain OS=Oryctolagus cuniculus OX= | #N/A | RABBIT | -2.20426 | #N/A | #N/A | -0.05764 | -1.06456 | 0.17216 | #N/A | -0.24749 | 0.02557 | 0.14796 | 1.133371 | 2.1339 | 2.07974 | 0.08598 | 0.07144 | 0.17534 | 0.17448 | 5 | 2.93229 | 0.55262 | 0.48359 | 0.509108 | Not Significant | NS | #N/A |
| 600 | G1SSK8 | Citrate synthase OS=Oryctolagus cuniculus OX=99 | CS | RABBIT | -1.3956 | -0.83068 | -0.20428 | 0.22764 | -0.29866 | 1.26129 | -0.75844 | 1.30421 | 0.82376 | 0.52854 | 1.132186 | 2.4145 | 2.41278 | 0.04221 | 0.03457 | 0.14488 | 0.1262 | 8 | 2.93229 | 0.55262 | 0.54969 | 0.550479 | Significant | Up in Mtub | CS |
| 468 | A0A5F9CY33 | Alcohol dehydrogenase class-3 OS=Oryctolagus cu | ADH5 | RABBIT | -1.53821 | -1.12926 | 0.09377 | 0.01924 | -0.74113 | 1.44272 | -0.17009 | 0.18318 | 0.45512 | 0.42787 | 1.126876 | 2.70626 | 2.61169 | 0.02681 | 0.02429 | 0.12384 | 0.11483 | 8 | 2.93229 | 0.55262 | 0.43346 | 0.465423 | Significant | Up in Mtub | ADH5 |
| 2871 | G1T5W1 | #N/A | PLPPR1 | RABBIT | #N/A | #N/A | #N/A | #N/A | -0.57012 | #N/A | #N/A | #N/A | #N/A | 0.55674 | 1.126863 | #N/A | 1.07187 | #N/A | 0.36397 | #N/A | 0.38261 | 0 | 2.93229 | 0.55262 | #N/A | 0.552617 | Not Significant | NS | #N/A |
| 721 | A0A5F9CFR1 | Heterogeneous nuclear ribonucleoprotein D like OS | HNRNPDL | RABBIT | -1.87449 | 0.16313 | -0.45646 | -0.29035 | 0.53702 | -0.57464 | 0.03469 | 0.98097 | 1.79303 | 1.47514 | 1.126068 | 1.86181 | 1.96925 | 0.09965 | 0.07479 | 0.18948 | 0.17859 | 8 | 2.93229 | 0.55262 | 0.91453 | 0.81746 | Not Significant | NS | #N/A |
| 222 | G1SHS7 | WD repeat domain 1 OS=Oryctolagus cuniculus OX | WDR1 | RABBIT | -0.62795 | -0.02735 | -0.89704 | -0.69068 | -0.9312 | 0.60951 | 0.37716 | 0.16081 | 0.68846 | 0.60997 | 1.124025 | 5.93261 | 3.84277 | 0.00035 | 0.00276 | 0.03578 | 0.05341 | 8 | 2.93229 | 0.55262 | 0.08974 | 0.213896 | Significant | Up in Mtub | WDR1 |
| 1962 | A0A5F9C0V7 | #N/A | KIAA0513 | RABBIT | #N/A | #N/A | 0.05662 | #N/A | -0.56525 | 0 | -0.91615 | 1.89571 | 1.5 | 1.86604 | 1.123434 | 1.17035 | 1.32049 | 0.2946 | 0.2235 | 0.32123 | 0.29937 | 5 | 2.93229 | 0.55262 | 1.31633 | 1.034014 | Not Significant | NS | #N/A |
| 997 | B7NZQ2 | Large ribosomal subunit protein uL16 OS=Oryctolag | RPL10 | RABBIT | -3.35919 | -2.42018 | 0.62603 | -0.11217 | 0.3133 | 0.34359 | -0.13026 | -0.00983 | -0.07446 | 0.51928 | 1.120109 | 1.38584 | 1.52804 | 0.2032 | 0.1549 | 0.27183 | 0.25326 | 8 | 2.93229 | 0.55262 | 1.63318 | 1.343349 | Not Significant | NS | #N/A |
| 1055 | A0A5F9DL18 | ADF-H domain-containing protein OS=Oryctolagus | #N/A | RABBIT | -1.43552 | -0.76959 | #N/A | #N/A | #N/A | 0.02102 | -0.08448 | -0.03671 | 0.17813 | 0 | 1.118149 | 5.84757 | 2.74401 | 0.00207 | 0.02549 | 0.05406 | 0.11552 | 5 | 2.93229 | 0.55262 | 0.05223 | 0.237208 | Significant | Up in Mtub | #N/A |
| 1569 | G1SYW3 | Dynein axonemal heavy chain 5 OS=Oryctolagus cu | DNAH5 | RABBIT | #N/A | -0.45994 | #N/A | #N/A | #N/A | #N/A | #N/A | #N/A | #N/A | 0.65776 | 1.117707 | #N/A | 1.06317 | #N/A | 0.36732 | #N/A | 0.38387 | 0 | 2.93229 | 0.55262 | #N/A | 0.552617 | Not Significant | NS | #N/A |
| 2595 | U3KPL6 | #N/A | VPS53 | RABBIT | #N/A | #N/A | #N/A | -0.61104 | #N/A | 1.80216 | #N/A | 1.4642 | -1.23979 | 0 | 1.117682 | 0.71273 | 0.88776 | 0.52745 | 0.40922 | 0.42539 | 0.4061 | 3 | 2.93229 | 0.55262 | 1.96733 | 1.268047 | Not Significant | NS | #N/A |
| 1263 | G1SPA9 | Fibrous sheath interacting protein 2 OS=Oryctolagu | FSIP2 | RABBIT | -0.12882 | #N/A | #N/A | #N/A | -0.82469 | -0.25377 | 0.44508 | 2.23182 | #N/A | 0.14048 | 1.117661 | 1.31336 | 1.45117 | 0.25933 | 0.19043 | 0.30317 | 0.27623 | 4 | 2.93229 | 0.55262 | 0.96559 | 0.790906 | Not Significant | NS | #N/A |
| 2520 | A0A5F9D4R9 | #N/A | PALS2 | RABBIT | #N/A | #N/A | #N/A | -0.24812 | #N/A | 2.60664 | #N/A | #N/A | -0.07368 | 0.07368 | 1.117003 | 0.64201 | 0.86553 | 0.58663 | 0.42683 | 0.44596 | 0.41011 | 2 | 2.93229 | 0.55262 | 2.27029 | 1.249117 | Not Significant | NS | #N/A |
| 2044 | G1TYL5 | #N/A | DNPH1 RC | RABBIT | #N/A | -0.82334 | -0.33534 | -0.28439 | #N/A | 0.22675 | #N/A | 0.29321 | #N/A | 1.38694 | 1.116657 | 2.70039 | 2.21348 | 0.05407 | 0.06285 | 0.15365 | 0.16451 | 4 | 2.93229 | 0.55262 | 0.25649 | 0.381751 | Not Significant | NS | #N/A |
| 2773 | G1T792 | #N/A | #N/A | RABBIT | #N/A | #N/A | #N/A | -0.5871 | #N/A | 0.52946 | #N/A | #N/A | #N/A | #N/A | 1.116566 | #N/A | 1.06208 | #N/A | 0.36774 | #N/A | 0.38387 | 0 | 2.93229 | 0.55262 | #N/A | 0.552617 | Not Significant | NS | #N/A |
| 2409 | G1U612 | #N/A | #N/A | RABBIT | #N/A | #N/A | #N/A | -0.21996 | -0.01338 | 3.38396 | 2.60745 | 1.07165 | -0.976 | -1.0966 | 1.114764 | 0.73138 | 0.87928 | 0.49737 | 0.4051 | 0.41323 | 0.40358 | 5 | 2.93229 | 0.55262 | 3.31878 | 2.296229 | Not Significant | NS | #N/A |
| 820 | A0A5F9DD95 | thioredoxin-dependent peroxiredoxin OS=Oryctolag | PRDX4 | RABBIT | -0.05133 | -0.00972 | #N/A | #N/A | 0.04961 | #N/A | #N/A | #N/A | 1.11093 | #N/A | 1.114744 | 19.0313 | 1.68161 | 0.00275 | 0.15428 | 0.05663 | 0.25312 | 2 | 2.93229 | 0.55262 | 0.00257 | 0.329579 | Not Significant | NS | #N/A |
| 923 | A0A5F9CH69 | Eukaryotic translation initiation factor 4 gamma 1 O | EIF4G1 | RABBIT | -0.9775 | -0.00822 | -1.12097 | 0.76963 | -1.92359 | 1.74447 | 0.49991 | 1.6838 | -1.85404 | 0.20604 | 1.108165 | 1.37734 | 1.5179 | 0.20571 | 0.15741 | 0.27238 | 0.25437 | 8 | 2.93229 | 0.55262 | 1.61833 | 1.33248 | Not Significant | NS | #N/A |
| 372 | G1SZE0 | Vacuolar protein sorting-associated protein 35 OS= | VPS35 | RABBIT | -1.85618 | -0.79828 | -0.28439 | -0.31482 | -0.23086 | 1.03702 | 0.23603 | 0.26768 | 0.29655 | 0.21748 | 1.107861 | 3.21075 | 2.89531 | 0.01241 | 0.01465 | 0.09752 | 0.09934 | 8 | 2.93229 | 0.55262 | 0.29764 | 0.366033 | Significant | Up in Mtub | VPS35 |
| 577 | G1U949 | Microtubule-associated protein RP/EB family memb | MAPRE1 | RABBIT | -0.15544 | 1.42514 | -2.66516 | 0.25805 | -3.96397 | -0.3275 | 0.02566 | 0.34357 | -0.02566 | 0.40577 | 1.104646 | 1.1022 | 1.23944 | 0.30243 | 0.24113 | 0.32474 | 0.31179 | 8 | 2.93229 | 0.55262 | 2.5111 | 1.98579 | Not Significant | NS | #N/A |
| 1584 | G1TEM5 | Protein dpy-30 homolog OS=Oryctolagus cuniculus | #N/A | RABBIT | -1.69233 | 0.19782 | #N/A | 1.03981 | -2.0586 | -0.43397 | -0.18137 | 0.71126 | 0.21872 | 2.06132 | 1.103516 | 1.34074 | 1.48682 | 0.22188 | 0.1681 | 0.28335 | 0.26263 | 7 | 2.93229 | 0.55262 | 1.50542 | 1.224127 | Not Significant | NS | #N/A |
| 1308 | Q0QEW4 | Ribosomal protein L18 (Fragment) OS=Oryctolagus | RPL18 | RABBIT | -3.38829 | -2.3659 | 0.88862 | -0.35114 | 0.28012 | -0.29598 | -0.30446 | 0.35856 | 0.34991 | 0.47119 | 1.103165 | 1.32894 | 1.46952 | 0.22052 | 0.16987 | 0.28284 | 0.26263 | 8 | 2.93229 | 0.55262 | 1.72271 | 1.408864 | Not Significant | NS | #N/A |
| 423 | A0A5F9CCC3 | Rab GDP dissociation inhibitor OS=Oryctolagus cun | #N/A | RABBIT | #N/A | -0.50308 | -0.9365 | -0.48184 | -0.97513 | 0.15416 | 0.00093 | -0.06437 | 1.01917 | 0.77443 | 1.101004 | 4.01134 | 3.09546 | 0.00512 | 0.01143 | 0.07293 | 0.09305 | 7 | 2.93229 | 0.55262 | 0.16741 | 0.281135 | Significant | Up in Mtub | #N/A |
| 1990 | G1TCK9 | #N/A | IARS1 | RABBIT | #N/A | #N/A | -0.90798 | 0.67279 | -2.73597 | 0.03841 | -0.03841 | -0.02092 | -0.31887 | 0.85961 | 1.094346 | 1.42768 | 1.56108 | 0.2033 | 0.15319 | 0.27183 | 0.2528 | 6 | 2.93229 | 0.55262 | 1.10166 | 0.921421 | Not Significant | NS | #N/A |
| 2491 | A0A5F9CJD6 | #N/A | #N/A | RABBIT | #N/A | #N/A | #N/A | -0.57445 | #N/A | #N/A | #N/A | #N/A | 0.51681 | #N/A | 1.091267 | #N/A | 1.03802 | #N/A | 0.37717 | #N/A | 0.38895 | 0 | 2.93229 | 0.55262 | #N/A | 0.552617 | Not Significant | NS | #N/A |
| 1267 | G1T644 | Protein kinase C OS=Oryctolagus cuniculus OX=99 | PRKCE | RABBIT | -0.78416 | -0.59053 | #N/A | #N/A | #N/A | -1.40129 | #N/A | 0.72399 | 0.92027 | 1.36013 | 1.08812 | 1.17666 | 1.33059 | 0.30457 | 0.22542 | 0.32493 | 0.29961 | 4 | 2.93229 | 0.55262 | 1.14022 | 0.891671 | Not Significant | NS | #N/A |
| 1819 | G1TJW8 | #N/A | #N/A | RABBIT | #N/A | #N/A | -1.2539 | -0.11897 | -1.15667 | 0.55891 | #N/A | -0.06437 | 0.11622 | 0.35237 | 1.083963 | 3.15194 | 2.46283 | 0.02532 | 0.03939 | 0.12061 | 0.13165 | 5 | 2.93229 | 0.55262 | 0.20275 | 0.332082 | Significant | Up in Mtub | #N/A |
| 1904 | A0A5F9DSC3 | #N/A | SUCLG2 | RABBIT | #N/A | 0.09735 | #N/A | #N/A | -0.01338 | 1.95345 | -0.48305 | #N/A | #N/A | 1.90706 | 1.083838 | 1.04267 | 1.2319 | 0.37373 | 0.26457 | 0.35717 | 0.32681 | 3 | 2.93229 | 0.55262 | 1.29664 | 0.928873 | Not Significant | NS | #N/A |
| 800 | G1SMY1 | RNA helicase OS=Oryctolagus cuniculus OX=9986 ( | DDX17 | RABBIT | -3.28128 | -1.41378 | 0.33189 | 0.0586 | -0.29799 | 0.07104 | -0.20032 | 0.21923 | -0.07104 | 0.79305 | 1.082906 | 1.58578 | 1.71107 | 0.15145 | 0.11526 | 0.23261 | 0.2141 | 8 | 2.93229 | 0.55262 | 1.16582 | 1.001348 | Not Significant | NS | #N/A |
| 2331 | G1SY84 | #N/A | DYNC1I2 | RABBIT | #N/A | #N/A | -1.59237 | -0.52092 | #N/A | 1.07485 | 0 | 0.11623 | -1.354 | 0.29234 | 1.082528 | 1.51265 | 1.58609 | 0.19078 | 0.1517 | 0.26255 | 0.25099 | 5 | 2.93229 | 0.55262 | 0.73165 | 0.665468 | Not Significant | NS | #N/A |
| 644 | A0A5F9DA95 | Neuronal membrane glycoprotein M6-a OS=Oryctol | GPM6A | RABBIT | -2.44441 | -3.19243 | 0.11879 | -0.21946 | 0.61976 | 0.32815 | 0.32672 | -0.01251 | -0.58913 | 0.21201 | 1.076598 | 1.39178 | 1.52688 | 0.20146 | 0.15519 | 0.2713 | 0.25326 | 8 | 2.93229 | 0.55262 | 1.49592 | 1.242906 | Not Significant | NS | #N/A |
| 1500 | A0A5F9CN24 | Pleckstrin homology like domain family B member 1 | PHLDB1 | RABBIT | -1.45705 | #N/A | #N/A | -0.05764 | #N/A | #N/A | #N/A | #N/A | #N/A | 0.31827 | 1.07561 | 0.88752 | 1.08014 | 0.53789 | 0.34183 | 0.42838 | 0.3686 | 1 | 2.93229 | 0.55262 | 0.97917 | 0.661092 | Not Significant | NS | #N/A |
| 2457 | G1TBC8 | #N/A | CTPS1 | RABBIT | #N/A | #N/A | -1.34351 | 0.20138 | -1.71253 | 1.27589 | 0.04539 | -0.88192 | -0.04539 | 0.21697 | 1.073744 | 1.70836 | 1.78435 | 0.13843 | 0.10828 | 0.22122 | 0.20721 | 6 | 2.93229 | 0.55262 | 0.7407 | 0.678955 | Not Significant | NS | #N/A |
| 1615 | G1SFN0 | BRCA1 associated RING domain 1 OS=Oryctolagus | BARD1 | RABBIT | -2.44023 | #N/A | 0.50496 | #N/A | -1.01717 | #N/A | 0.51093 | -0.32788 | 0.15928 | 0 | 1.069731 | 1.4441 | 1.56865 | 0.20832 | 0.15569 | 0.27318 | 0.25326 | 5 | 2.93229 | 0.55262 | 0.94067 | 0.797221 | Not Significant | NS | #N/A |
| 2753 | P9WP17 | #N/A | #N/A | MT | #N/A | #N/A | #N/A | -0.05764 | -2.13338 | 0.95196 | -1.21818 | 0.22223 | -0.3893 | 0.30044 | 1.06894 | 1.29962 | 1.41656 | 0.25042 | 0.19466 | 0.2994 | 0.27975 | 5 | 2.93229 | 0.55262 | 0.96644 | 0.813465 | Not Significant | NS | #N/A |
| 488 | A0A5F9D155 | Ubiquitin carboxyl-terminal hydrolase OS=Oryctola | USP14 | RABBIT | -1.5139 | -1.36499 | #N/A | 0.4534 | -0.45825 | #N/A | 0.33009 | -0.06437 | 0.77668 | #N/A | 1.068404 | 1.85422 | 1.86426 | 0.12288 | 0.09959 | 0.2103 | 0.19972 | 5 | 2.93229 | 0.55262 | 0.56916 | 0.563043 | Not Significant | NS | #N/A |
| 2137 | U3KM64 | #N/A | CLTA | RABBIT | #N/A | #N/A | -0.77214 | #N/A | -0.68391 | #N/A | 1.13316 | 0.08417 | 0.27927 | -0.14854 | 1.065041 | 2.53585 | 2.02326 | 0.06426 | 0.08313 | 0.15873 | 0.18576 | 4 | 2.93229 | 0.55262 | 0.23519 | 0.36946 | Not Significant | NS | #N/A |
| 2236 | B7NZG7 | #N/A | SNX3 | RABBIT | #N/A | #N/A | -0.6545 | 0.05166 | -2.77043 | 0.38424 | -1.40253 | -0.17367 | 0.43943 | 0.43806 | 1.061529 | 1.36303 | 1.49501 | 0.2218 | 0.16938 | 0.28335 | 0.26263 | 6 | 2.93229 | 0.55262 | 1.13724 | 0.94532 | Not Significant | NS | #N/A |
| 2266 | G1U724 | #N/A | SAE1 | RABBIT | #N/A | #N/A | -1.72457 | 0.68425 | -1.2966 | 1.83269 | 0.02119 | -0.08556 | -1.19307 | 0.82851 | 1.059729 | 1.22747 | 1.37104 | 0.26562 | 0.20382 | 0.30603 | 0.28553 | 6 | 2.93229 | 0.55262 | 1.39756 | 1.120184 | Not Significant | NS | #N/A |
| 2624 | A0A5F9CTL2 | #N/A | SIPA1L3 | RABBIT | #N/A | #N/A | #N/A | -0.88637 | #N/A | 0.51031 | #N/A | #N/A | 0.25544 | -0.25544 | 1.056474 | 2.34632 | 1.4647 | 0.14354 | 0.20367 | 0.22569 | 0.28553 | 2 | 2.93229 | 0.55262 | 0.15206 | 0.390193 | Not Significant | NS | #N/A |
| 2100 | G1T310 | #N/A | PRXL2A | RABBIT | #N/A | #N/A | -0.00891 | -0.30606 | -1.66906 | 0.17957 | 0.29429 | 1.1456 | -0.58842 | 0.94216 | 1.055985 | 1.90583 | 1.91846 | 0.10532 | 0.08752 | 0.19468 | 0.1893 | 6 | 2.93229 | 0.55262 | 0.57564 | 0.568082 | Not Significant | NS | #N/A |
| 47 | A0A1K0GV16 | Globin A1 OS=Oryctolagus cuniculus OX=9986 GN= | GLNA1 | RABBIT | 3.95361 | #N/A | -6.31365 | 4.39161 | -6.92406 | -3.94195 | 5.25306 | -6.74442 | 4.76061 | #N/A | 1.054946 | 0.24207 | 0.29431 | 0.81679 | 0.77524 | 0.51745 | 0.53596 | 6 | 2.93229 | 0.55262 | 37.9848 | 25.6966 | Not Significant | NS | #N/A |
| 969 | G1SRH7 | Serine and arginine rich splicing factor 3 OS=Orycto | SRSF3 | RABBIT | -1.69088 | -1.36333 | 0.11679 | 0.523 | -0.8777 | 0.17961 | -0.59946 | 0.76973 | 0.05386 | 1.57779 | 1.054728 | 1.87943 | 1.95941 | 0.09699 | 0.07605 | 0.18685 | 0.1805 | 8 | 2.93229 | 0.55262 | 0.78735 | 0.724389 | Not Significant | NS | #N/A |
| 2313 | A0A5F9CJ54 | #N/A | RASAL1 | RABBIT | #N/A | #N/A | -0.44137 | #N/A | #N/A | 0.61202 | #N/A | #N/A | #N/A | #N/A | 1.053397 | #N/A | 1.00199 | #N/A | 0.39172 | #N/A | 0.396 | 0 | 2.93229 | 0.55262 | #N/A | 0.552617 | Not Significant | NS | #N/A |
| 1074 | G1TL09 | RING-type E3 ubiquitin transferase OS=Oryctolagus | TRIM32 | RABBIT | #N/A | -0.42221 | #N/A | #N/A | -0.39921 | -1.20889 | 1.66304 | 1.72034 | 0.38583 | #N/A | 1.050794 | 1.0167 | 1.18099 | 0.36679 | 0.27653 | 0.35413 | 0.33229 | 4 | 2.93229 | 0.55262 | 1.42425 | 1.055558 | Not Significant | NS | #N/A |
| 1916 | G1T5E2 | #N/A | #N/A | RABBIT | #N/A | -1.5181 | 0.17065 | -0.08976 | -1.20733 | -0.22627 | 0.09506 | 0.5237 | 0.29919 | 1.25557 | 1.050587 | 2.28332 | 2.22664 | 0.05636 | 0.0503 | 0.15432 | 0.15072 | 7 | 2.93229 | 0.55262 | 0.47045 | 0.49471 | Not Significant | NS | #N/A |
| 2020 | G1T4E6 | #N/A | USP1 | RABBIT | #N/A | -1.02194 | #N/A | #N/A | -0.68413 | 0.08639 | #N/A | 0.50582 | #N/A | 0 | 1.050437 | 4.41846 | 2.07525 | 0.02152 | 0.08381 | 0.11776 | 0.18662 | 3 | 2.93229 | 0.55262 | 0.06782 | 0.307453 | Not Significant | NS | #N/A |
| 2648 | Q6IVG4 | #N/A | ACAP2 CE | RABBIT | #N/A | #N/A | #N/A | -1.16478 | #N/A | 0.10726 | #N/A | -1.04607 | 0 | 0.4812 | 1.050377 | 1.4355 | 1.34245 | 0.24665 | 0.22854 | 0.29787 | 0.30142 | 3 | 2.93229 | 0.55262 | 0.42833 | 0.489762 | Not Significant | NS | #N/A |
| 1039 | G1TM86 | Guanine nucleotide-binding protein-like 1 OS=Oryc | GNL1 | RABBIT | -1.09179 | -2.28905 | 0.48543 | -0.24774 | -0.19671 | 1.4603 | 0.18333 | -0.88735 | 0.75809 | 0.38339 | 1.047521 | 1.71157 | 1.8142 | 0.12534 | 0.09716 | 0.21204 | 0.19688 | 8 | 2.93229 | 0.55262 | 0.93643 | 0.833481 | Not Significant | NS | #N/A |
| 1231 | G1SMT7 | NHL repeat containing 2 OS=Oryctolagus cuniculus | NHLRC2 | RABBIT | -0.34701 | -1.63594 | -0.34318 | -0.00775 | 0.51724 | 1.67045 | -0.22412 | -0.11425 | 1.1087 | 0.97961 | 1.047405 | 2.04926 | 2.0929 | 0.07459 | 0.06049 | 0.1678 | 0.16242 | 8 | 2.93229 | 0.55262 | 0.65309 | 0.626144 | Not Significant | NS | #N/A |
| 807 | G1TC10 | UBC core domain-containing protein OS=Oryctolag | #N/A | RABBIT | -1.60691 | -0.98735 | 0.26942 | -0.15641 | -0.40316 | 1.1599 | -0.39429 | 0.20004 | 0.92201 | 0.45691 | 1.045799 | 2.44703 | 2.38083 | 0.04012 | 0.03658 | 0.14205 | 0.12876 | 8 | 2.93229 | 0.55262 | 0.45662 | 0.482371 | Significant | Up in Mtub | #N/A |
| 2665 | G1SRZ6 | #N/A | BLVRA | RABBIT | #N/A | #N/A | #N/A | -0.62112 | #N/A | 1.58934 | -0.41573 | -0.60637 | 1.12478 | 0.41573 | 1.042664 | 1.00109 | 1.09516 | 0.37343 | 0.31003 | 0.35712 | 0.35209 | 4 | 2.93229 | 0.55262 | 0.90399 | 0.755361 | Not Significant | NS | #N/A |
| 854 | G1T6C0 | small monomeric GTPase OS=Oryctolagus cuniculu | RAB5B | RABBIT | -2.01574 | -1.77649 | -0.22575 | 0.05739 | -0.12841 | -0.72076 | 0.16106 | 0.14441 | 0.75498 | 0.78239 | 1.042215 | 1.9979 | 2.05017 | 0.08078 | 0.06511 | 0.1731 | 0.16763 | 8 | 2.93229 | 0.55262 | 0.68031 | 0.646061 | Not Significant | NS | #N/A |
| 1377 | A0A5F9CYQ1 | Inosine-5'-monophosphate dehydrogenase OS=Ory | IMPDH2 IM | RABBIT | -2.42609 | -0.49219 | 1.12106 | -0.78041 | -0.05889 | 0.04551 | -0.33418 | 1.34145 | 1.05079 | 0.4667 | 1.041357 | 1.59539 | 1.70952 | 0.14929 | 0.11555 | 0.23045 | 0.21416 | 8 | 2.93229 | 0.55262 | 1.06514 | 0.92767 | Not Significant | NS | #N/A |
| 400 | A0A5F9C991 | Eukaryotic translation initiation factor 3 subunit A O | EIF3A EIF3 | RABBIT | -1.12411 | -1.30052 | -0.6581 | -0.12462 | 0.10165 | -0.07684 | 1.13356 | 0.21222 | 0.06698 | 0.75867 | 1.040059 | 2.92808 | 2.67107 | 0.01905 | 0.02186 | 0.11259 | 0.11054 | 8 | 2.93229 | 0.55262 | 0.31542 | 0.379041 | Significant | Up in Mtub | EIF3A EIF3S10 |
| 785 | G1STW0 | Large ribosomal subunit protein eL8 OS=Oryctolagu | RPL7A | RABBIT | -3.75117 | #N/A | 0.77601 | -1.26991 | 0.31599 | -0.03526 | 0.06204 | 0.36472 | -0.11014 | 0 | 1.038539 | 1.15115 | 1.29109 | 0.28747 | 0.22591 | 0.31864 | 0.30003 | 7 | 2.93229 | 0.55262 | 1.80871 | 1.437879 | Not Significant | NS | #N/A |
| 684 | A0A5F9CVA7 | 10-formyltetrahydrofolate dehydrogenase OS=Oryc | ALDH1L2 | RABBIT | -0.04577 | #N/A | 0.39219 | #N/A | -0.92934 | 2.83012 | 0.63927 | #N/A | -0.11447 | 0 | 1.033039 | 1.18411 | 1.33485 | 0.28959 | 0.21897 | 0.32005 | 0.29655 | 5 | 2.93229 | 0.55262 | 1.30476 | 1.02672 | Not Significant | NS | #N/A |
| 858 | G1SFH6 | Ubiquitin like modifier activating enzyme 2 OS=Oryc | UBA2 | RABBIT | -2.36056 | -0.12104 | -0.21132 | 0.51429 | -1.14963 | 0.65902 | -0.01894 | 0.42384 | 0.01894 | 0.74577 | 1.031376 | 1.96599 | 2.02004 | 0.08487 | 0.06857 | 0.17453 | 0.17188 | 8 | 2.93229 | 0.55262 | 0.68804 | 0.651713 | Not Significant | NS | #N/A |
| 2394 | G1T302 | #N/A | THYN1 | RABBIT | #N/A | #N/A | -1.00517 | #N/A | #N/A | 0.4704 | -0.77131 | #N/A | 0 | 0.40009 | 1.029964 | 1.61693 | 1.39307 | 0.20432 | 0.21356 | 0.27183 | 0.29197 | 3 | 2.93229 | 0.55262 | 0.3246 | 0.437309 | Not Significant | NS | #N/A |
| 1736 | U3KMY4 | #N/A | IQCF3 | RABBIT | #N/A | #N/A | #N/A | -0.44056 | #N/A | 1.17788 | #N/A | #N/A | #N/A | 0 | 1.029505 | 1.00924 | 1.09575 | 0.49707 | 0.33572 | 0.41323 | 0.36569 | 1 | 2.93229 | 0.55262 | 0.6937 | 0.588496 | Not Significant | NS | #N/A |
| 2073 | A0A375Z4U2 | #N/A | #N/A | MT | #N/A | -0.53429 | #N/A | #N/A | #N/A | #N/A | #N/A | -0.1398 | 0.07543 | 1.54366 | 1.027385 | 0.97117 | 1.0879 | 0.43391 | 0.32692 | 0.38352 | 0.35977 | 2 | 2.93229 | 0.55262 | 0.83934 | 0.668882 | Not Significant | NS | #N/A |
| 2228 | G1SFX7 | #N/A | ARF6 | RABBIT | #N/A | #N/A | -1.24261 | -1.2307 | #N/A | 0.10143 | -2.0743 | -0.06437 | 0.57287 | 0.40687 | 1.025155 | 1.27923 | 1.38508 | 0.25697 | 0.20373 | 0.30258 | 0.28553 | 5 | 2.93229 | 0.55262 | 0.91746 | 0.78259 | Not Significant | NS | #N/A |
| 12 | P30947 | Heat shock protein HSP 90-beta OS=Oryctolagus cu | HSP90AB1 | RABBIT | -1.90185 | -1.88168 | 0.1628 | -1.77161 | 0.1467 | -0.6199 | 0.29438 | -0.04512 | 0.23989 | 0.00785 | 1.024549 | 1.97751 | 2.02605 | 0.08337 | 0.06787 | 0.17453 | 0.17137 | 8 | 2.93229 | 0.55262 | 0.67107 | 0.6393 | Not Significant | NS | #N/A |
| 1196 | G1SYS4 | Eukaryotic translation initiation factor 4C OS=Oryct | #N/A | RABBIT | -0.04883 | -0.74097 | #N/A | 0.00286 | #N/A | #N/A | 0.75971 | #N/A | #N/A | #N/A | 1.022032 | 2.13107 | 1.40213 | 0.16678 | 0.22057 | 0.24583 | 0.2975 | 2 | 2.93229 | 0.55262 | 0.1725 | 0.398484 | Not Significant | NS | #N/A |
| 661 | G1SDK8 | NEDD8-activating enzyme E1 regulatory subunit OS | NAE1 | RABBIT | -0.71702 | -2.61542 | -0.96783 | -0.22209 | 0.53745 | 0.92761 | 0.16445 | -0.88268 | 0.42902 | 0.48646 | 1.021956 | 1.69297 | 1.79004 | 0.12892 | 0.10115 | 0.21348 | 0.20137 | 8 | 2.93229 | 0.55262 | 0.91097 | 0.814853 | Not Significant | NS | #N/A |
| 1250 | G1SJ43 | Protein tyrosine phosphatase non-receptor type 23 | PTPN23 | RABBIT | #N/A | 1.56059 | #N/A | -2.73086 | #N/A | 2.74125 | 0 | -3.4222 | 2.99839 | -0.13382 | 1.021859 | 0.45211 | 0.55722 | 0.67013 | 0.59275 | 0.47323 | 0.4738 | 5 | 2.93229 | 0.55262 | 7.29776 | 4.804321 | Not Significant | NS | #N/A |
| 663 | G1SX37 | Cofilin-2 OS=Oryctolagus cuniculus OX=9986 GN=( | CFL2 | RABBIT | -0.91902 | -0.04135 | -0.67116 | -0.39483 | -0.15367 | 1.41566 | 0.14029 | 0.49773 | 0.50846 | 0.3523 | 1.018893 | 3.74411 | 3.02458 | 0.00567 | 0.01164 | 0.07337 | 0.09305 | 8 | 2.93229 | 0.55262 | 0.18514 | 0.283706 | Significant | Up in Mtub | CFL2 |
| 1280 | G1TL56 | HIRA interacting protein 3 OS=Oryctolagus cunicul | HIRIP3 | RABBIT | -1.8556 | #N/A | #N/A | -0.05764 | #N/A | #N/A | #N/A | #N/A | 0.06121 | #N/A | 1.017831 | 0.65368 | 0.916 | 0.63142 | 0.41232 | 0.46125 | 0.40746 | 1 | 2.93229 | 0.55262 | 1.61634 | 0.823127 | Not Significant | NS | #N/A |
| 1472 | G1SWN4 | Ubiquitin-conjugating enzyme E2 Z OS=Oryctolagus | UBE2Z | RABBIT | -0.12352 | -1.17469 | #N/A | -0.52119 | #N/A | 0.39533 | -0.83677 | 0.07082 | 1.77703 | 0.63471 | 1.014687 | 1.67018 | 1.72831 | 0.14592 | 0.11825 | 0.22839 | 0.21684 | 6 | 2.93229 | 0.55262 | 0.69205 | 0.64628 | Not Significant | NS | #N/A |
| 2442 | A0A5F9D9H0 | #N/A | #N/A | RABBIT | #N/A | #N/A | -0.11711 | #N/A | #N/A | #N/A | #N/A | 1.79417 | 0 | #N/A | 1.014192 | 0.65272 | 0.91369 | 0.63185 | 0.4134 | 0.46125 | 0.40806 | 1 | 2.93229 | 0.55262 | 1.60951 | 0.821391 | Not Significant | NS | #N/A |
| 550 | G1SLZ8 | Tyrosine-protein phosphatase non-receptor type OS | PTPN11 | RABBIT | -1.84269 | 0.10278 | -1.98286 | 0.51205 | -1.02559 | 0.17073 | -1.00108 | 0.72296 | 0.84378 | 0.09504 | 1.013547 | 1.68933 | 1.78419 | 0.12963 | 0.10214 | 0.21441 | 0.20152 | 8 | 2.93229 | 0.55262 | 0.89991 | 0.806758 | Not Significant | NS | #N/A |
| 2093 | G1TIZ7 | #N/A | ZSWIM1 | RABBIT | #N/A | 0.19415 | 0.17065 | 0.44042 | #N/A | #N/A | 2.75077 | #N/A | #N/A | -0.18705 | 1.013449 | 0.92089 | 1.1057 | 0.42502 | 0.31167 | 0.38058 | 0.35272 | 3 | 2.93229 | 0.55262 | 1.45335 | 1.008122 | Not Significant | NS | #N/A |
| 1325 | G1SLZ9 | Eukaryotic translation initiation factor 4E family mei | EIF4E2 | RABBIT | 0.01166 | 0.27901 | #N/A | -1.52647 | #N/A | 1.60074 | #N/A | #N/A | -0.3979 | #N/A | 1.013353 | 0.97406 | 1.15112 | 0.40187 | 0.29396 | 0.3719 | 0.34341 | 3 | 2.93229 | 0.55262 | 1.29877 | 0.929952 | Not Significant | NS | #N/A |
| 1237 | A0A5F9CD33 | Succinyl-CoA:3-ketoacid-coenzyme A transferase ( | OXCT1 | RABBIT | #N/A | #N/A | #N/A | -0.05949 | -0.79339 | 1.17036 | #N/A | #N/A | #N/A | 0.00185 | 1.012542 | 1.4676 | 1.40205 | 0.27992 | 0.22059 | 0.31436 | 0.2975 | 2 | 2.93229 | 0.55262 | 0.47601 | 0.521552 | Not Significant | NS | #N/A |
| 897 | G1SQ22 | Coatomer subunit gamma OS=Oryctolagus cunicul | COPG1 | RABBIT | -1.52214 | -1.39616 | 0.13573 | -0.02272 | -0.1271 | 0.4363 | 0.93442 | 0.26839 | -0.22212 | 0.71328 | 1.012531 | 2.46756 | 2.37013 | 0.03886 | 0.03727 | 0.1391 | 0.12876 | 8 | 2.93229 | 0.55262 | 0.42094 | 0.456259 | Significant | Up in Mtub | COPG1 |
| 52 | G1U410 | Tubulin beta chain OS=Oryctolagus cuniculus OX=9 | TUBB4A | RABBIT | -2.71525 | -0.84539 | 0.27317 | 0.33959 | -0.90363 | -0.10252 | -0.5577 | 0.55437 | 0.68423 | 0.62491 | 1.010963 | 1.67051 | 1.76711 | 0.13336 | 0.10508 | 0.21764 | 0.20407 | 8 | 2.93229 | 0.55262 | 0.91561 | 0.818248 | Not Significant | NS | #N/A |
| 894 | A0A5F9DSQ7 | Large ribosomal subunit protein eL30 OS=Oryctolag | #N/A | RABBIT | -2.98734 | -2.10238 | 0.89 | -0.7418 | 0.69106 | 0.24293 | 0.54965 | 0.639 | -0.38491 | -0.24293 | 1.010838 | 1.28386 | 1.41141 | 0.23513 | 0.18594 | 0.29167 | 0.27484 | 8 | 2.93229 | 0.55262 | 1.54978 | 1.282316 | Not Significant | NS | #N/A |
| 2756 | G1SXG0 | #N/A | GALNT8 | RABBIT | #N/A | #N/A | #N/A | -0.53321 | #N/A | #N/A | #N/A | #N/A | 0.47557 | #N/A | 1.008787 | #N/A | 0.95956 | #N/A | 0.40954 | #N/A | 0.4061 | 0 | 2.93229 | 0.55262 | #N/A | 0.552617 | Not Significant | NS | #N/A |
| 792 | G1SR28 | Platelet-activating factor acetylhydrolase IB subunit | PAFAH1B3 | RABBIT | -1.06221 | -0.27807 | -0.17345 | 0.28646 | -0.90925 | 0.79826 | -0.36624 | 0.49081 | 0.99969 | 0.95854 | 1.003517 | 2.83732 | 2.58393 | 0.0219 | 0.02552 | 0.11776 | 0.11552 | 8 | 2.93229 | 0.55262 | 0.31273 | 0.377076 | Significant | Up in Mtub | PAFAH1B3 |
| 2070 | A0A5F9CLW2 | #N/A | RO60 | RABBIT | #N/A | -1.95262 | -1.43241 | 0.87369 | #N/A | 0.30767 | -0.42165 | 0.93598 | 0.08522 | -0.08522 | 1.001511 | 1.42544 | 1.53024 | 0.20391 | 0.16057 | 0.27183 | 0.25538 | 6 | 2.93229 | 0.55262 | 0.92558 | 0.803144 | Not Significant | NS | #N/A |
| 812 | G1TPW2 | Methylthioribose-1-phosphate isomerase OS=Oryc | MRI1 | RABBIT | -0.85932 | 0.05793 | -1.52599 | -0.22586 | -0.95997 | 0.45858 | 0.23546 | 0.07553 | 0.24938 | 0.46009 | 0.998451 | 3.44265 | 2.87222 | 0.00879 | 0.01527 | 0.09045 | 0.09934 | 8 | 2.93229 | 0.55262 | 0.21028 | 0.302106 | Significant | Up in Mtub | MRI1 |
| 1503 | A0A5F9C476 | Integrin subunit alpha M OS=Oryctolagus cuniculus | ITGAM | RABBIT | -0.68879 | #N/A | #N/A | #N/A | #N/A | -1.77132 | 1.99632 | #N/A | #N/A | 0.70045 | 0.997269 | 0.4512 | 0.6412 | 0.69605 | 0.54999 | 0.4833 | 0.46295 | 2 | 2.93229 | 0.55262 | 3.664 | 1.814258 | Not Significant | NS | #N/A |
| 442 | G1TF82 | Alanyl-tRNA synthetase domain containing 1 OS=O | AARSD1 | RABBIT | -0.8348 | -0.93121 | -0.35129 | -0.4377 | 0.70986 | 0.63438 | 0.38007 | 1.23004 | 1.34549 | -0.45072 | 0.996882 | 2.28568 | 2.23756 | 0.05161 | 0.04704 | 0.15041 | 0.14585 | 8 | 2.93229 | 0.55262 | 0.47555 | 0.496222 | Significant | Up in Mtub | AARSD1 |
| 2307 | G1U944 | #N/A | NDRG4 | RABBIT | #N/A | #N/A | -0.81718 | -1.08358 | #N/A | #N/A | #N/A | 0.03106 | 0 | 0.09694 | 0.99305 | 9.37601 | 2.05595 | 0.00257 | 0.08607 | 0.05406 | 0.18814 | 3 | 2.93229 | 0.55262 | 0.01346 | 0.279962 | Not Significant | NS | #N/A |
| 2224 | G1U1P4 | #N/A | PRKCG | RABBIT | #N/A | #N/A | -0.40983 | #N/A | #N/A | #N/A | #N/A | #N/A | #N/A | 0.58048 | 0.990318 | #N/A | 0.94199 | #N/A | 0.41714 | #N/A | 0.40859 | 0 | 2.93229 | 0.55262 | #N/A | 0.552617 | Not Significant | NS | #N/A |
| 1640 | A5U6U8 | Fatty-acid-CoA ligase FadD28 OS=Mycobacterium t | #N/A | MT | -0.52039 | #N/A | #N/A | #N/A | #N/A | #N/A | #N/A | 0.46768 | #N/A | #N/A | 0.988072 | #N/A | 0.93986 | #N/A | 0.41807 | #N/A | 0.40859 | 0 | 2.93229 | 0.55262 | #N/A | 0.552617 | Not Significant | NS | #N/A |
| 92 | G1TMP7 | Syntaxin-binding protein 1 OS=Oryctolagus cunicul | STXBP1 | RABBIT | -0.40594 | 0.50079 | 0.6387 | 0.5456 | -6.6467 | 0.27635 | -0.48292 | -0.00083 | -0.16024 | -0.06354 | 0.987275 | 0.69951 | 0.80158 | 0.50406 | 0.43987 | 0.41624 | 0.41667 | 8 | 2.93229 | 0.55262 | 4.97997 | 3.792455 | Not Significant | NS | #N/A |
| 1578 | P62497 | Eukaryotic peptide chain release factor subunit 1 O | ETF1 ERF1 | RABBIT | -2.83911 | -0.28243 | -0.38978 | 0.24521 | -0.18281 | 0.76674 | -0.42459 | 0.19735 | 0.16943 | 0.77007 | 0.985586 | 1.66616 | 1.7551 | 0.13424 | 0.10719 | 0.21787 | 0.20635 | 8 | 2.93229 | 0.55262 | 0.87477 | 0.78836 | Not Significant | NS | #N/A |
| 2558 | A0A5F9CGB2 | #N/A | ARFGEF3 | RABBIT | #N/A | #N/A | #N/A | -0.37077 | #N/A | 1.92054 | #N/A | -0.39097 | #N/A | 0.31313 | 0.984997 | 0.71997 | 0.9003 | 0.54631 | 0.40976 | 0.43084 | 0.4061 | 2 | 2.93229 | 0.55262 | 1.40378 | 0.897754 | Not Significant | NS | #N/A |
| 2086 | G1TBS3 | #N/A | DDX59 | RABBIT | #N/A | -1.73874 | 0.17065 | #N/A | #N/A | #N/A | #N/A | 0.20019 | #N/A | #N/A | 0.984236 | 0.59522 | 0.85879 | 0.6582 | 0.43965 | 0.47085 | 0.41667 | 1 | 2.93229 | 0.55262 | 1.82288 | 0.875652 | Not Significant | NS | #N/A |
| 1491 | G1SCS2 | Cytochrome P450 family 26 subfamily C member 1 | CYP26C1 | RABBIT | #N/A | #N/A | #N/A | -0.52067 | #N/A | 0.46303 | #N/A | #N/A | #N/A | #N/A | 0.983696 | #N/A | 0.93569 | #N/A | 0.4199 | #N/A | 0.40859 | 0 | 2.93229 | 0.55262 | #N/A | 0.552617 | Not Significant | NS | #N/A |

| | | | | | | | | | | | | | | | | | | | | | | | | | | | | | |
|---|---|---|---|---|---|---|---|---|---|---|---|---|---|---|---|---|---|---|---|---|---|---|---|---|---|---|---|---|---|
| 226 | G1SZF7 | Isocitrate dehydrogenase [NADP] OS=Oryctolagus c | IDH2 | RABBIT | -1.25437 | -0.21757 | 0.12769 | 0.01835 | -0.43769 | #N/A | 0 | 1.00748 | 1.04194 | 0.47246 | 0.983189 | 2.78345 | 2.44767 | 0.02716 | 0.03455 | 0.124 | 0.1262 | 7 | 2.93229 | 0.55262 | 0.27726 | 0.358556 | Significant | Up in Mtub | IDH2 |
| 1534 | G1SM52 | Leucine rich repeat containing 59 OS=Oryctolagus c | LRRC59 | RABBIT | #N/A | -0.95418 | #N/A | #N/A | #N/A | #N/A | 0.05688 | #N/A | 0 | #N/A | 0.982622 | 19.9465 | 1.2492 | 0.03189 | 0.28079 | 0.13025 | 0.3349 | 1 | 2.93229 | 0.55262 | 0.00162 | 0.412495 | Not Significant | NS | #N/A |
| 2450 | G1TYC6 | #N/A | #N/A | RABBIT | #N/A | #N/A | 0.25448 | #N/A | -0.47601 | #N/A | -0.08383 | 1.82032 | #N/A | #N/A | 0.979011 | 0.96006 | 1.13032 | 0.43833 | 0.31029 | 0.38563 | 0.35209 | 2 | 2.93229 | 0.55262 | 1.03986 | 0.750191 | Not Significant | NS | #N/A |
| 1089 | G1SUT0 | Transportin 2 OS=Oryctolagus cuniculus OX=9986 ( | TNPO2 | RABBIT | -2.28458 | -1.93737 | 0.91888 | 1.72668 | -2.05971 | 1.2119 | -1.01473 | 0.02591 | -0.09028 | 1.12273 | 0.978324 | 1.03559 | 1.15913 | 0.33068 | 0.2711 | 0.33706 | 0.33012 | 8 | 2.93229 | 0.55262 | 2.23114 | 1.780919 | Not Significant | NS | #N/A |
| 1980 | A0A5F9DHU0 | #N/A | CANX | RABBIT | #N/A | #N/A | 0.15968 | -0.17325 | -0.50235 | -0.30099 | 0.01097 | 2.35824 | 1.09976 | 0.86349 | 0.97827 | 1.53393 | 1.60835 | 0.17594 | 0.14248 | 0.25448 | 0.24186 | 6 | 2.93229 | 0.55262 | 0.76262 | 0.693679 | Not Significant | NS | #N/A |
| 624 | A0A5F9CPJ9 | Kinesin light chain OS=Oryctolagus cuniculus OX=9 | KLC1 | RABBIT | -2.52287 | -0.37033 | -0.14967 | #N/A | 0.00406 | 0.13097 | 0.10658 | #N/A | -0.01744 | 0.65124 | 0.97754 | 1.60006 | 1.67297 | 0.1607 | 0.12892 | 0.23977 | 0.22709 | 6 | 2.93229 | 0.55262 | 0.74649 | 0.682847 | Not Significant | NS | #N/A |
| 697 | G1U1H3 | Ras-related protein Rab-4B OS=Oryctolagus cunicu | #N/A | RABBIT | 0.01166 | #N/A | #N/A | #N/A | #N/A | #N/A | -0.11489 | #N/A | 2.09275 | #N/A | 0.977269 | 0.51116 | 0.78555 | 0.69918 | 0.47676 | 0.48373 | 0.43293 | 1 | 2.93229 | 0.55262 | 2.43683 | 1.031782 | Not Significant | NS | #N/A |
| 692 | G1TWL0 | Heterogeneous nuclear ribonucleoproteins A2/B1 C | #N/A | RABBIT | 2.09738 | -0.8751 | -0.88854 | -0.16424 | -2.23424 | 0.27274 | 2.22063 | 0.04223 | -0.28688 | 0.56464 | 0.975618 | 1.16834 | 1.29274 | 0.2763 | 0.22275 | 0.31174 | 0.29908 | 8 | 2.93229 | 0.55262 | 1.74325 | 1.423892 | Not Significant | NS | #N/A |
| 498 | A0A5F9DNU4 | Erythrocyte membrane protein band 4.1 like 1 OS=( | EPB41L1 | RABBIT | -1.19091 | -0.3849 | -0.30483 | 0.94458 | -1.12735 | 1.28451 | -0.39072 | 0.94979 | 0.57536 | 0.39072 | 0.974615 | 2.03652 | 2.04611 | 0.07608 | 0.06557 | 0.16938 | 0.16855 | 8 | 2.93229 | 0.55262 | 0.57257 | 0.567217 | Not Significant | NS | #N/A |
| 2081 | A0A5F9CLL5 | #N/A | FAHD2A | RABBIT | #N/A | -0.22755 | #N/A | #N/A | #N/A | 0 | #N/A | #N/A | 1.49127 | #N/A | 0.973181 | 0.75354 | 0.95324 | 0.58889 | 0.39531 | 0.44648 | 0.39935 | 1 | 2.93229 | 0.55262 | 1.11194 | 0.694855 | Not Significant | NS | #N/A |
| 976 | G1T7X6 | Laminin subunit beta 1 OS=Oryctolagus cuniculus ( | LAMB1 | RABBIT | -1.70096 | -2.19568 | -1.16036 | 0.23823 | 1.59093 | #N/A | 1.53299 | -0.86964 | 0 | 0.64584 | 0.972864 | 1.07841 | 1.2095 | 0.3166 | 0.25447 | 0.33209 | 0.32053 | 7 | 2.93229 | 0.55262 | 1.80852 | 1.437746 | Not Significant | NS | #N/A |
| 1444 | A0A5F9CL60 | Uncharacterized protein OS=Oryctolagus cuniculus | #N/A | RABBIT | -1.73894 | #N/A | #N/A | -0.15548 | -0.40944 | 0.09785 | -0.75404 | 0.31062 | 0.41717 | 0.9487 | 0.972015 | 1.8864 | 1.85322 | 0.10819 | 0.0971 | 0.1968 | 0.19688 | 6 | 2.93229 | 0.55262 | 0.49783 | 0.515815 | Not Significant | NS | #N/A |
| 1593 | Q28667 | Low-density lipoprotein receptor-related protein/alp | #N/A | RABBIT | -0.7362 | -1.17438 | -0.6774 | 0.0741 | -0.33252 | #N/A | 1.11596 | 0.19103 | 0.30329 | 0 | 0.971852 | 3.02907 | 2.54376 | 0.01914 | 0.02932 | 0.11259 | 0.12083 | 7 | 2.93229 | 0.55262 | 0.22875 | 0.324367 | Significant | Up in Mtub | #N/A |
| 362 | G1SNC4 | Adenylate kinase isoenzyme 1 OS=Oryctolagus cun | AK1 | RABBIT | -1.13392 | -0.82259 | -3.35523 | 0.81828 | -0.01338 | 1.33327 | #N/A | -2.78014 | 0.88137 | 0.84525 | 0.971302 | 0.83985 | 0.96359 | 0.42874 | 0.35812 | 0.38146 | 0.37856 | 7 | 2.93229 | 0.55262 | 2.97228 | 2.257926 | Not Significant | NS | #N/A |
| 1463 | A0A5F9DKW8 | non-specific serine/threonine protein kinase OS=Or | TRIO | RABBIT | #N/A | #N/A | -2.15463 | -0.03996 | -0.72118 | 0.52796 | -1.32832 | 0.43123 | -0.01768 | 0.38281 | 0.971119 | 1.50146 | 1.58004 | 0.18391 | 0.14881 | 0.25808 | 0.24819 | 6 | 2.93229 | 0.55262 | 0.78437 | 0.708289 | Not Significant | NS | #N/A |
| 2221 | G1T361 | #N/A | SUCLG2 | RABBIT | #N/A | #N/A | -0.11856 | -1.23918 | #N/A | #N/A | #N/A | 0.29418 | 0.28921 | #N/A | 0.970565 | 1.73217 | 1.43753 | 0.22538 | 0.21085 | 0.28607 | 0.29037 | 2 | 2.93229 | 0.55262 | 0.31396 | 0.455842 | Not Significant | NS | #N/A |
| 2860 | G1SHV4 | #N/A | TSHZ2 | RABBIT | #N/A | #N/A | #N/A | #N/A | -0.49186 | 0.47848 | #N/A | #N/A | #N/A | #N/A | 0.970345 | #N/A | 0.92299 | #N/A | 0.42551 | #N/A | 0.41009 | 0 | 2.93229 | 0.55262 | #N/A | 0.552617 | Not Significant | NS | #N/A |
| 1019 | Q9TRF8 | Class PI glutathione S-transferase isozyme R12 OS= | #N/A | RABBIT | -1.21349 | -0.83554 | 0.02908 | 0.22867 | -0.54968 | 0.96096 | -0.12232 | 0.05795 | 1.08729 | 0.52477 | 0.969924 | 2.7054 | 2.47686 | 0.02685 | 0.03087 | 0.12384 | 0.12309 | 8 | 2.93229 | 0.55262 | 0.32133 | 0.383367 | Significant | Up in Mtub | #N/A |
| 1641 | G1TEM3 | Fraser extracellular matrix complex subunit 1 OS=O | FRAS1 | RABBIT | #N/A | -0.41073 | #N/A | #N/A | -0.52238 | #N/A | 0 | 0.51384 | #N/A | 0.98658 | 0.966695 | 2.61178 | 1.7741 | 0.07956 | 0.12697 | 0.17277 | 0.22608 | 3 | 2.93229 | 0.55262 | 0.16439 | 0.35629 | Not Significant | NS | #N/A |
| 1475 | G1SQA1 | Carboxymethylenebutenolidase homolog OS=Oryc | CMBL | RABBIT | -3.18111 | 0.07311 | -0.64146 | 0.06707 | 0.42063 | 0.46299 | -0.89068 | -0.62685 | 1.52945 | 1.09232 | 0.965799 | 1.19647 | 1.31905 | 0.26577 | 0.21411 | 0.30603 | 0.29205 | 8 | 2.93229 | 0.55262 | 1.62895 | 1.340256 | Not Significant | NS | #N/A |
| 2107 | G1SEU9 | #N/A | LAMTOR3 | RABBIT | #N/A | -0.52388 | -0.96943 | -0.53066 | -0.54738 | 0.90905 | 0 | 0.05846 | 0.41647 | 0.22784 | 0.965205 | 4.62302 | 2.991 | 0.00242 | 0.01365 | 0.05406 | 0.09774 | 7 | 2.93229 | 0.55262 | 0.09687 | 0.231417 | Significant | Up in Mtub | LAMTOR3 |
| 790 | G1SZ00 | Cysteine and glycine-rich protein 1 OS=Oryctolagus | CSRP1 | RABBIT | -0.92613 | -1.80131 | -0.22843 | -0.61819 | -0.00913 | 0.7742 | 0.23692 | 0.16876 | 0.06613 | -0.00426 | 0.96499 | 2.81575 | 2.53211 | 0.02264 | 0.02798 | 0.11776 | 0.11898 | 8 | 2.93229 | 0.55262 | 0.29363 | 0.363094 | Significant | Up in Mtub | CSRP1 |
| 758 | A0A5F9DLL4 | HECT and RLD domain containing E3 ubiquitin prote | HERC4 | RABBIT | -1.05758 | -0.54742 | -0.67135 | 0.6649 | -0.11554 | 1.69643 | 0.14979 | 0.03779 | 2.34904 | -1.14293 | 0.963422 | 1.39465 | 1.50738 | 0.20063 | 0.16005 | 0.27128 | 0.25503 | 8 | 2.93229 | 0.55262 | 1.19301 | 1.02124 | Not Significant | NS | #N/A |
| 2143 | G1SH86 | #N/A | TBCD | RABBIT | #N/A | #N/A | -1.06483 | -0.25831 | #N/A | 1.28375 | -0.79954 | 0.1363 | #N/A | 0.58164 | 0.962109 | 1.37582 | 1.42245 | 0.24089 | 0.1983 | 0.29524 | 0.28262 | 4 | 2.93229 | 0.55262 | 0.65203 | 0.609978 | Not Significant | NS | #N/A |
| 2827 | G1SJV1 | #N/A | #N/A | RABBIT | #N/A | #N/A | #N/A | #N/A | -0.48635 | 0.47296 | #N/A | #N/A | #N/A | #N/A | 0.959307 | #N/A | 0.9125 | #N/A | 0.4302 | #N/A | 0.41183 | 0 | 2.93229 | 0.55262 | #N/A | 0.552617 | Not Significant | NS | #N/A |
| 1997 | G1U7L1 | #N/A | RPL28 | RABBIT | #N/A | -3.77173 | 0.28538 | -0.1448 | -0.16487 | 0 | 0.37986 | 0.30438 | 0.13565 | -0.77004 | 0.958973 | 1.11046 | 1.23905 | 0.30348 | 0.24381 | 0.32493 | 0.31383 | 7 | 2.93229 | 0.55262 | 1.65727 | 1.331147 | Not Significant | NS | #N/A |
| 876 | G1U3I5 | Heterogeneous nuclear ribonucleoprotein L OS=Ory | HNRNPL | RABBIT | -1.36318 | 0.05841 | -0.17781 | 0.55326 | -1.05465 | 0.13941 | -0.23865 | 1.11447 | 0.2233 | 1.54427 | 0.953352 | 1.96064 | 1.97798 | 0.08557 | 0.07368 | 0.17476 | 0.17651 | 8 | 2.93229 | 0.55262 | 0.59109 | 0.58077 | Not Significant | NS | #N/A |
| 471 | G1SW24 | Alanine--tRNA ligase OS=Oryctolagus cuniculus OX | AARS AARS | RABBIT | -1.62934 | -1.50741 | -0.25526 | -0.05009 | 0.02322 | 0.31072 | -0.20398 | -0.07191 | 0.78083 | 0.53095 | 0.953096 | 2.33586 | 2.23951 | 0.04772 | 0.04688 | 0.14885 | 0.14585 | 8 | 2.93229 | 0.55262 | 0.41622 | 0.452803 | Significant | Up in Mtub | AARS AARS1 |
| 1425 | A0A375YME4 | Putative cyclase (Adenylyl-or guanylyl-)(Adenylate-c | #N/A | MT | -1.02103 | 0.19782 | #N/A | #N/A | #N/A | #N/A | #N/A | 0.54142 | #N/A | #N/A | 0.953029 | 0.90287 | 1.00376 | 0.53247 | 0.3732 | 0.42755 | 0.3868 | 1 | 2.93229 | 0.55262 | 0.7428 | 0.600981 | Not Significant | NS | #N/A |
| 849 | G1U6H4 | Dimethylarginine dimethylaminohydrolase 2 OS=Or | DDAH2 | RABBIT | -2.72644 | 0.26091 | 0.16517 | -1.07174 | 0.43718 | -0.03135 | -0.32354 | 0.49979 | 0.00548 | 1.67444 | 0.951948 | 1.37123 | 1.48313 | 0.20753 | 0.16629 | 0.27264 | 0.26156 | 8 | 2.93229 | 0.55262 | 1.20489 | 1.029933 | Not Significant | NS | #N/A |
| 2772 | G1SDN9 | #N/A | PTGFRN | RABBIT | #N/A | #N/A | #N/A | -0.75266 | -0.04723 | 0.03384 | #N/A | #N/A | 1.06989 | #N/A | 0.95181 | 1.51876 | 1.3628 | 0.26815 | 0.23187 | 0.30684 | 0.30329 | 2 | 2.93229 | 0.55262 | 0.39276 | 0.487795 | Not Significant | NS | #N/A |
| 2368 | A0A0B5H464 | #N/A | #N/A | RABBIT | #N/A | #N/A | -0.61828 | #N/A | -1.1612 | 1.22181 | -1.33511 | 0.11637 | 0 | 0.29543 | 0.949441 | 1.35524 | 1.41164 | 0.23335 | 0.19606 | 0.29088 | 0.28064 | 5 | 2.93229 | 0.55262 | 0.70114 | 0.646238 | Not Significant | NS | #N/A |
| 2390 | G1TZQ6 | #N/A | NDUFA10 | RABBIT | #N/A | #N/A | 0.17065 | #N/A | #N/A | #N/A | #N/A | 2.28813 | #N/A | -0.04996 | 0.948437 | 0.4684 | 0.73596 | 0.72113 | 0.50324 | 0.4902 | 0.445 | 1 | 2.93229 | 0.55262 | 2.73332 | 1.107181 | Not Significant | NS | #N/A |
| 1927 | A0A5F9D6J1 | #N/A | FRZB | RABBIT | #N/A | -1.744 | -1.20213 | 0.28025 | -1.59443 | 0.2983 | -1.65512 | 0.38803 | 0.37805 | 0 | 0.946932 | 1.57514 | 1.65309 | 0.15923 | 0.12953 | 0.23848 | 0.22754 | 7 | 2.93229 | 0.55262 | 0.80313 | 0.729173 | Not Significant | NS | #N/A |
| 1737 | A0A5F9DNC0 | #N/A | NDRG2 | RABBIT | #N/A | 0.27699 | #N/A | -0.35055 | -0.09256 | #N/A | 0.89074 | #N/A | #N/A | #N/A | 0.946106 | 2.59765 | 1.34905 | 0.12172 | 0.23594 | 0.20964 | 0.30623 | 2 | 2.93229 | 0.55262 | 0.09949 | 0.368878 | Not Significant | NS | #N/A |
| 2197 | A0A5F9D129 | #N/A | PLXNA4 | RABBIT | #N/A | #N/A | -1.59669 | -0.12079 | 0.35806 | 2.52411 | -0.73618 | -0.00121 | 1.36941 | -0.69244 | 0.945876 | 0.99679 | 1.12924 | 0.35735 | 0.28821 | 0.35078 | 0.33905 | 6 | 2.93229 | 0.55262 | 1.68835 | 1.315512 | Not Significant | NS | #N/A |
| 482 | A0AAG1WUK3 | Ribosomal protein S3 OS=Oryctolagus cuniculus O) | RPS3 | RABBIT | -2.33685 | -1.43281 | 0.18895 | -0.09599 | -0.05299 | 0.03792 | 0.38716 | 0.28043 | -0.0183 | 0.29975 | 0.943329 | 1.90489 | 1.9306 | 0.09326 | 0.07987 | 0.18402 | 0.18325 | 8 | 2.93229 | 0.55262 | 0.61309 | 0.596872 | Not Significant | NS | #N/A |
| 1410 | A0A5F9CQE6 | Intracellular hyaluronan-binding protein 4 N-termin | #N/A | RABBIT | -0.49796 | #N/A | #N/A | #N/A | #N/A | #N/A | #N/A | 0.44525 | #N/A | #N/A | 0.943211 | #N/A | 0.89718 | #N/A | 0.43712 | #N/A | 0.41582 | 0 | 2.93229 | 0.55262 | #N/A | 0.552617 | Not Significant | NS | #N/A |
| 2155 | A0A5F9DF65 | #N/A | WDR7 | RABBIT | #N/A | #N/A | -0.23882 | #N/A | #N/A | #N/A | #N/A | 1.40805 | 0 | #N/A | 0.942851 | 0.7732 | 0.94462 | 0.58099 | 0.3992 | 0.44338 | 0.40143 | 1 | 2.93229 | 0.55262 | 0.99131 | 0.664178 | Not Significant | NS | #N/A |
| 155 | G1TC44 | Peptidyl-prolyl cis-trans isomerase OS=Oryctolagus | #N/A | RABBIT | -1.40359 | -0.78994 | -0.29689 | 0.02892 | 0.13387 | 0.97139 | -0.08656 | 0.46451 | 1.28774 | -0.25226 | 0.942492 | 2.29648 | 2.20593 | 0.05075 | 0.04972 | 0.15026 | 0.14997 | 8 | 2.93229 | 0.55262 | 0.42109 | 0.456366 | Significant | Up in Mtub | #N/A |
| 2537 | A0A5F9D2K0 | #N/A | #N/A | RABBIT | #N/A | #N/A | #N/A | -0.66913 | #N/A | -0.22512 | #N/A | -0.06437 | 1.08372 | 0.29647 | 0.941801 | 1.44483 | 1.26269 | 0.24425 | 0.25406 | 0.29685 | 0.32053 | 3 | 2.93229 | 0.55262 | 0.33992 | 0.445054 | Not Significant | NS | #N/A |
| 1230 | G1T2U7 | Dynein cytoplasmic 1 intermediate chain 1 OS=Ory | DYNC1I1 | RABBIT | -0.16235 | -1.21297 | -0.91851 | 0.09138 | -0.1624 | 0.19252 | -0.33114 | 0.89982 | 0.78123 | 0.78812 | 0.939079 | 2.73429 | 2.461 | 0.02567 | 0.03175 | 0.12149 | 0.1243 | 8 | 2.93229 | 0.55262 | 0.29489 | 0.364015 | Significant | Up in Mtub | DYNC1I1 |
| 2746 | G1T194 | #N/A | AKAP8L | RABBIT | #N/A | #N/A | #N/A | -1.06282 | #N/A | -0.89119 | #N/A | -0.06437 | 0.02673 | 0.4306 | 0.938265 | 1.51343 | 1.2818 | 0.22738 | 0.24773 | 0.28736 | 0.31626 | 3 | 2.93229 | 0.55262 | 0.30748 | 0.428649 | Not Significant | NS | #N/A |
| 508 | G1TJ67 | RAB3D, member RAS oncogene family OS=Oryctola | RAB3D | RABBIT | #N/A | -1.05937 | 0.15406 | -0.21652 | #N/A | 0.6655 | #N/A | -0.06437 | 0.96023 | 0.69512 | 0.938068 | 2.36133 | 2.00616 | 0.06464 | 0.08006 | 0.15913 | 0.18325 | 5 | 2.93229 | 0.55262 | 0.27054 | 0.374817 | Not Significant | NS | #N/A |
| 2242 | A0A5F9D8G2 | #N/A | NAA10 | RABBIT | #N/A | #N/A | -0.78876 | 0.22502 | -0.77994 | 1.54059 | -1.27363 | -0.34703 | 1.38277 | 1.14579 | 0.93759 | 1.2041 | 1.32064 | 0.27389 | 0.21946 | 0.31045 | 0.29674 | 6 | 2.93229 | 0.55262 | 1.13685 | 0.945059 | Not Significant | NS | #N/A |
| 1217 | G1T8C2 | Double-strand break repair protein OS=Oryctolagus | MRE11 | RABBIT | -1.87441 | -1.08525 | 0.17065 | -0.17505 | #N/A | 0.66758 | -0.86362 | 0.09642 | 0.88528 | 0.18968 | 0.936085 | 1.76313 | 1.79461 | 0.12125 | 0.10316 | 0.20956 | 0.20233 | 7 | 2.93229 | 0.55262 | 0.6264 | 0.604616 | Not Significant | NS | #N/A |
| 2268 | G1TAG4 | #N/A | RHPN2 | RABBIT | #N/A | #N/A | -0.04197 | #N/A | -1.10581 | #N/A | #N/A | 0.14825 | 0.57122 | #N/A | 0.93362 | 1.631 | 1.37446 | 0.24447 | 0.22846 | 0.29685 | 0.30142 | 2 | 2.93229 | 0.55262 | 0.32766 | 0.461401 | Not Significant | NS | #N/A |
| 2367 | G1TEU8 | #N/A | #N/A | RABBIT | #N/A | #N/A | #N/A | -1.13895 | #N/A | -1.82008 | 1.08131 | -2.55382 | 1.10791 | 1.15644 | 0.933297 | 0.46623 | 0.57962 | 0.66531 | 0.58051 | 0.47235 | 0.47216 | 4 | 2.93229 | 0.55262 | 3.33932 | 2.160573 | Not Significant | NS | #N/A |
| 1791 | A0A5F9DCJ0 | #N/A | CRYZ | RABBIT | #N/A | -2.67848 | -0.03123 | 0.36225 | #N/A | 1.05859 | 0 | 0.10898 | -0.56528 | #N/A | 0.933059 | 1.04519 | 1.18355 | 0.34381 | 0.27084 | 0.34542 | 0.33012 | 5 | 2.93229 | 0.55262 | 1.36618 | 1.065437 | Not Significant | NS | #N/A |
| 699 | G1TKL2 | EF-hand domain-containing protein OS=Oryctolagu | #N/A | RABBIT | -0.44642 | -1.15636 | 0.02625 | -0.64647 | -0.055 | 0.04162 | 1.14644 | 0.1577 | 0.57001 | 0.45566 | 0.929886 | 3.21899 | 2.68038 | 0.01226 | 0.0215 | 0.09752 | 0.11031 | 8 | 2.93229 | 0.55262 | 0.20862 | 0.300889 | Significant | Up in Mtub | #N/A |
| 615 | G1T567 | Transforming protein RhoA OS=Oryctolagus cunicul | RHOA | RABBIT | -0.841 | -0.76923 | -0.0688 | -0.22617 | -0.3602 | 1.00081 | 0.3451 | 0.40308 | 0.45915 | 0.16853 | 0.928409 | 4.50102 | 3.08744 | 0.002 | 0.0104 | 0.05406 | 0.09154 | 8 | 2.93229 | 0.55262 | 0.10636 | 0.226059 | Significant | Up in Mtub | RHOA |
| 75 | G1U5S4 | Tubulin beta chain OS=Oryctolagus cuniculus OX=9 | TUBB3 | RABBIT | -1.47787 | -1.17358 | -0.07678 | 0.55902 | -0.92102 | 1.04322 | -0.67557 | 0.18306 | 0.49155 | 0.47252 | 0.921002 | 1.96306 | 1.96195 | 0.08525 | 0.07573 | 0.17453 | 0.18007 | 8 | 2.93229 | 0.55262 | 0.55029 | 0.550917 | Not Significant | NS | #N/A |
| 1314 | A0A5F9D5L9 | Sema domain-containing protein OS=Oryctolagus c | PLXNA3 | RABBIT | -0.58736 | #N/A | #N/A | #N/A | #N/A | #N/A | 0.41356 | -0.47793 | 1.06387 | #N/A | 0.920527 | 1.02992 | 1.05455 | 0.4113 | 0.34052 | 0.37603 | 0.36807 | 2 | 2.93229 | 0.55262 | 0.59913 | 0.571479 | Not Significant | NS | #N/A |
| 2243 | G1SY87 | #N/A | CD200 | RABBIT | #N/A | #N/A | 0.17065 | #N/A | -0.08938 | #N/A | 0.9609 | #N/A | #N/A | #N/A | 0.920259 | 4.0866 | 1.15848 | 0.15278 | 0.31215 | 0.23368 | 0.35276 | 1 | 2.93229 | 0.55262 | 0.03381 | 0.420681 | Not Significant | NS | #N/A |
| 2397 | A0A5F9CWM2 | #N/A | ROGDI | RABBIT | #N/A | #N/A | -0.38132 | #N/A | #N/A | 1.46261 | #N/A | 0.79173 | -0.10425 | 0 | 0.918841 | 1.11797 | 1.11177 | 0.34504 | 0.30925 | 0.34542 | 0.35209 | 3 | 2.93229 | 0.55262 | 0.5404 | 0.546438 | Not Significant | NS | #N/A |
| 126 | P55823 | Elongation factor 2 OS=Oryctolagus cuniculus OX=9 | EEF2 | RABBIT | -1.09687 | -1.17478 | -0.23283 | -0.41705 | -0.13626 | 0.80851 | 0.12287 | 0.11939 | 0.30655 | 0.17806 | 0.918636 | 3.60713 | 2.81162 | 0.00691 | 0.01701 | 0.07833 | 0.10198 | 8 | 2.93229 | 0.55262 | 0.16215 | 0.266879 | Significant | Up in Mtub | EEF2 |
| 747 | A0A5F9DCT1 | Large ribosomal subunit protein uL4 OS=Oryctolagu | RPL4 | RABBIT | -3.12353 | -1.99298 | 0.75371 | -0.22655 | 0.81173 | -0.05219 | 0.05219 | 0.35698 | -0.08853 | 0.53856 | 0.916927 | 1.16201 | 1.27779 | 0.27872 | 0.22779 | 0.3135 | 0.30112 | 8 | 2.93229 | 0.55262 | 1.55664 | 1.28734 | Not Significant | NS | #N/A |
| 1252 | G1TRG8 | Guanine nucleotide-binding protein G(i) subunit alp | GNAI2 | RABBIT | -2.40236 | #N/A | #N/A | -0.53329 | -0.01338 | 0.99362 | 0.80608 | -2.44752 | #N/A | 0.38113 | 0.916337 | 0.81214 | 0.95451 | 0.45362 | 0.368 | 0.39325 | 0.38387 | 5 | 2.93229 | 0.55262 | 2.18237 | 1.579905 | Not Significant | NS | #N/A |
| 352 | G1T277 | Actin-related protein 2/3 complex subunit 3 OS=Ory | #N/A | RABBIT | -0.12725 | -0.67692 | -0.64795 | -0.39579 | -0.50064 | 0.29034 | 0.78499 | 0.07454 | 0.54504 | 0.53594 | 0.91588 | 5.83094 | 3.29326 | 0.00039 | 0.00722 | 0.03578 | 0.07947 | 8 | 2.93229 | 0.55262 | 0.06168 | 0.19336 | Significant | Up in Mtub | #N/A |
| 2452 | G1SJQ4 | #N/A | #N/A | RABBIT | #N/A | #N/A | #N/A | -0.8823 | #N/A | 0 | #N/A | #N/A | #N/A | 0.05877 | 0.911685 | 17.9138 | 1.15898 | 0.0355 | 0.31197 | 0.13507 | 0.35276 | 1 | 2.93229 | 0.55262 | 0.00173 | 0.412523 | Not Significant | NS | #N/A |
| 1437 | G1T047 | Uncharacterized protein OS=Oryctolagus cuniculus | #N/A | RABBIT | #N/A | #N/A | #N/A | -4.37766 | 1.53226 | #N/A | -0.52856 | 0.84849 | -2.89579 | 0.52856 | 0.910872 | 0.41191 | 0.52616 | 0.70152 | 0.61518 | 0.48408 | 0.4825 | 4 | 2.93229 | 0.55262 | 6.52001 | 3.995864 | Not Significant | NS | #N/A |
| 1049 | G1T3T2 | Glutamate decarboxylase 1 OS=Oryctolagus cunicu | GAD1 | RABBIT | -0.88998 | 0.61674 | -0.4749 | -0.2127 | -2.41747 | 0.60057 | -1.09068 | 0.0907 | 1.33041 | 0.24395 | 0.910654 | 1.42948 | 1.52568 | 0.19073 | 0.15548 | 0.26255 | 0.25326 | 8 | 2.93229 | 0.55262 | 1.01458 | 0.890675 | Not Significant | NS | #N/A |
| 2148 | P63169 | #N/A | DYNLL1 DI | RABBIT | #N/A | #N/A | #N/A | #N/A | -0.95673 | 0.25271 | 0.25494 | -0.06437 | -0.63099 | #N/A | 0.9098 | 1.95019 | 1.35399 | 0.14625 | 0.22504 | 0.22839 | 0.29961 | 3 | 2.93229 | 0.55262 | 0.17411 | 0.361204 | Not Significant | NS | #N/A |
| 2676 | G1TJQ2 | #N/A | LYPD6B | RABBIT | #N/A | #N/A | #N/A | -0.4827 | #N/A | #N/A | 0.42506 | #N/A | #N/A | #N/A | 0.907762 | #N/A | 0.86347 | #N/A | 0.45272 | #N/A | 0.4223 | 0 | 2.93229 | 0.55262 | #N/A | 0.552617 | Not Significant | NS | #N/A |
| 1361 | U3KP70 | CUGBP Elav-like family member 2 OS=Oryctolagus | CELF2 | RABBIT | #N/A | -0.91107 | -2.17051 | 1.35065 | -0.81151 | 0 | -1.1534 | 0.7506 | 0.8502 | 0.91052 | 0.907196 | 1.1618 | 1.27896 | 0.2834 | 0.22998 | 0.31656 | 0.30239 | 7 | 2.93229 | 0.55262 | 1.35497 | 1.118091 | Not Significant | NS | #N/A |
| 939 | G1TAT4 | Dual specificity protein phosphatase OS=Oryctolag | #N/A | RABBIT | -1.96937 | -1.63118 | 0.12287 | 0.64023 | -0.10906 | 1.09672 | -1.12023 | -0.01659 | 1.02132 | 0.60185 | 0.905918 | 1.38137 | 1.4813 | 0.20452 | 0.16676 | 0.27183 | 0.26184 | 8 | 2.93229 | 0.55262 | 1.07522 | 0.935045 | Not Significant | NS | #N/A |
| 1247 | A0A5F9C531 | glycogenin glucosyltransferase OS=Oryctolagus cu | #N/A | RABBIT | -1.41241 | #N/A | -0.06523 | -0.79227 | 0.032 | 0 | #N/A | 0.40846 | 0.62153 | #N/A | 0.902805 | 2.10589 | 1.86229 | 0.08908 | 0.09989 | 0.17844 | 0.20009 | 5 | 2.93229 | 0.55262 | 0.31506 | 0.402879 | Not Significant | NS | #N/A |
| 1460 | U3KLV2 | FACT complex subunit SSRP1 OS=Oryctolagus cuni | SSRP1 | RABBIT | #N/A | #N/A | #N/A | -0.30667 | #N/A | -0.41858 | 0.24903 | 1.78272 | -0.33921 | 1.70308 | 0.90208 | 0.76308 | 0.86528 | 0.48794 | 0.41582 | 0.40933 | 0.40833 | 4 | 2.93229 | 0.55262 | 1.16458 | 0.905724 | Not Significant | NS | #N/A |
| 2813 | G1SHA4 | #N/A | TEP1 | RABBIT | #N/A | #N/A | #N/A | #N/A | -0.60609 | #N/A | 1.21555 | 0.52834 | #N/A | -0.85851 | 0.901215 | 0.73873 | 0.88306 | 0.537 | 0.41816 | 0.42838 | 0.40859 | 2 | 2.93229 | 0.55262 | 1.11622 | 0.781153 | Not Significant | NS | #N/A |
| 138 | G1SZW0 | Cytoplasmic FMR1-interacting protein OS=Oryctola | CYFIP1 | RABBIT | -1.49233 | -2.47539 | -1.10838 | 0.55783 | 0.67053 | -0.48778 | 0.20612 | 0.5692 | 0.57225 | -0.20612 | 0.900279 | 1.39989 | 1.49643 | 0.19911 | 0.16284 | 0.26982 | 0.25804 | 8 | 2.93229 | 0.55262 | 1.03397 | 0.904861 | Not Significant | NS | #N/A |
| 2299 | A0A5F9DII4 | #N/A | SRP68 | RABBIT | #N/A | #N/A | -1.02324 | -0.05764 | #N/A | 0.38024 | 0.95821 | -0.57279 | -0.73845 | 1.76648 | 0.899178 | 1.08793 | 1.18727 | 0.32626 | 0.26946 | 0.33572 | 0.32966 | 5 | 2.93229 | 0.55262 | 0.97586 | 0.819403 | Not Significant | NS | #N/A |
| 541 | G1TEG8 | BRO1 domain-containing protein OS=Oryctolagus c | #N/A | RABBIT | -1.07699 | -0.22238 | -1.01284 | 0.0025 | -0.38949 | 0.71846 | 0.63123 | -0.07638 | 0.50763 | 0.01202 | 0.898429 | 3.31938 | 2.67387 | 0.01055 | 0.02175 | 0.09648 | 0.11035 | 8 | 2.93229 | 0.55262 | 0.18314 | 0.282245 | Significant | Up in Mtub | #N/A |
| 1785 | G1TVS8 | #N/A | RPL13A | RABBIT | #N/A | -2.55108 | 0.21587 | -1.48522 | 0.04633 | 0.0155 | -0.11545 | -0.06437 | -0.21406 | 0.14453 | 0.896756 | 1.53992 | 1.60438 | 0.16747 | 0.13992 | 0.24611 | 0.23907 | 7 | 2.93229 | 0.55262 | 0.75359 | 0.69426 | Not Significant | NS | #N/A |
| 2231 | G1SL42 | #N/A | NIPSNAP1 | RABBIT | #N/A | #N/A | -0.58024 | 0.04622 | #N/A | 0.88149 | #N/A | 2.70903 | -0.10385 | -0.96868 | 0.896508 | 0.74728 | 0.89392 | 0.49643 | 0.40133 | 0.41323 | 0.40169 | 4 | 2.93229 | 0.55262 | 1.91903 | 1.341054 | Not Significant | NS | #N/A |
| 1487 | G1TX26 | Calmodulin regulated spectrin associated protein fa | CAMSAP3 | RABBIT | -0.49887 | #N/A | #N/A | -0.16955 | -0.29892 | 0.11191 | -0.72116 | 1.0993 | 1.83049 | 0.54621 | 0.895797 | 1.54102 | 1.5744 | 0.17424 | 0.1501 | 0.25301 | 0.24914 | 6 | 2.93229 | 0.55262 | 0.63358 | 0.607001 | Not Significant | NS | #N/A |
| 1901 | A0A5F9CIN6 | #N/A | STK24 | RABBIT | #N/A | #N/A | #N/A | -0.92477 | #N/A | -0.10536 | -0.52303 | 0.26775 | 0.10536 | 0.10691 | 0.895093 | 2.67052 | 1.52319 | 0.05578 | 0.17194 | 0.15432 | 0.26466 | 4 | 2.93229 | 0.55262 | 0.09362 | 0.28777 | Not Significant | NS | #N/A |
| 1917 | A0A5F9CWG5 | #N/A | CEP250 | RABBIT | #N/A | #N/A | #N/A | -0.80702 | #N/A | #N/A | 0.23712 | -0.06437 | #N/A | #N/A | 0.893393 | 3.42171 | 1.12072 | 0.18101 | 0.32614 | 0.25572 | 0.35925 | 1 | 2.93229 | 0.55262 | 0.04545 | 0.423641 | Not Significant | NS | #N/A |
| 982 | A0A5F9DUB6 | leucine--tRNA ligase OS=Oryctolagus cuniculus OX | LARS1 | RABBIT | -0.96716 | -2.35116 | 0.21542 | 1.42741 | -1.62116 | 0.14783 | 0.25191 | -0.10914 | -0.47573 | 1.3509 | 0.892487 | 1.21013 | 1.31975 | 0.26077 | 0.21389 | 0.30397 | 0.29197 | 8 | 2.93229 | 0.55262 | 1.35981 | 1.143304 | Not Significant | NS | #N/A |
| 2612 | G1SRW3 | #N/A | CFAP36 | RABBIT | #N/A | #N/A | #N/A | -0.66779 | #N/A | #N/A | #N/A | -0.40556 | 0.73013 | 0.3412 | 0.889711 | 1.33499 | 1.13162 | 0.31355 | 0.30979 | 0.33031 | 0.35209 | 2 | 2.93229 | 0.55262 | 0.33312 | 0.463613 | Not Significant | NS | #N/A |
| 2577 | G1SZM8 | #N/A | BORCS5 | RABBIT | #N/A | #N/A | #N/A | -0.47156 | #N/A | #N/A | #N/A | #N/A | 0.41392 | #N/A | 0.885482 | #N/A | 0.84227 | #N/A | 0.46278 | #N/A | 0.42567 | 0 | 2.93229 | 0.55262 | #N/A | 0.552617 | Not Significant | NS | #N/A |
| 1078 | G1U1M3 | Aminoacyl tRNA synthetase complex interacting mu | AIMP1 | RABBIT | 0.01166 | -1.03586 | 1.49968 | -0.59715 | -1.40705 | -0.04237 | 0.25916 | 1.10717 | 0.99039 | #N/A | 0.884328 | 1.40898 | 1.49256 | 0.20168 | 0.16662 | 0.2713 | 0.26184 | 7 | 2.93229 | 0.55262 | 0.8754 | 0.780103 | Not Significant | NS | #N/A |
| 904 | G1TD29 | PHD finger protein 24 OS=Oryctolagus cuniculus O) | PHF24 | RABBIT | -2.02714 | -0.68487 | -0.20519 | 1.17088 | -0.64548 | 1.26496 | -0.67337 | 0.31147 | 0.50316 | 0.62328 | 0.884263 | 1.47047 | 1.5537 | 0.17964 | 0.14871 | 0.25448 | 0.24819 | 8 | 2.93229 | 0.55262 | 0.90404 | 0.809783 | Not Significant | NS | #N/A |
| 329 | G1T4Z2 | ATP-citrate synthase OS=Oryctolagus cuniculus OX | ACLY | RABBIT | -1.1828 | -1.27093 | 0.05134 | 0.02142 | -0.52354 | 0.21616 | -0.07906 | 0.50536 | 0.2349 | 0.62663 | 0.881698 | 2.85457 | 2.45387 | 0.02133 | 0.03215 | 0.11776 | 0.12472 | 8 | 2.93229 | 0.55262 | 0.23851 | 0.322757 | Significant | Up in Mtub | ACLY |
| 1168 | A0A5F9CVG7 | mannose-1-phosphate guanylyltransferase OS=Ory | GMPPB | RABBIT | -1.7192 | #N/A | 0.17065 | 0.15898 | -1.13255 | 1.02737 | -0.11866 | -1.08137 | 1.22153 | 0.20298 | 0.880902 | 1.3997 | 1.48357 | 0.20433 | 0.16895 | 0.27183 | 0.26263 | 7 | 2.93229 | 0.55262 | 0.88019 | 0.783479 | Not Significant | NS | #N/A |
| 1004 | A0A5F9CKH0 | DnaJ homolog subfamily C member 5 OS=Oryctolag | #N/A | RABBIT | -1.54426 | -1.67828 | 0.8225 | -1.25638 | 0.00047 | 0.68021 | 0.27228 | 0.96308 | -0.01385 | -1.15427 | 0.880678 | 1.44119 | 1.52718 | 0.1875 | 0.15512 | 0.26061 | 0.25326 | 8 | 2.93229 | 0.55262 | 0.93354 | 0.831366 | Not Significant | NS | #N/A |
| 992 | G1U7C6 | H1.3 linker histone, cluster member OS=Oryctolagu | H1-3 | RABBIT | 0.08485 | -0.76471 | #N/A | -0.13082 | #N/A | -0.47464 | #N/A | #N/A | 1.36383 | 0.93616 | 0.878679 | 1.43772 | 1.4419 | 0.22389 | 0.19294 | 0.28492 | 0.27822 | 4 | 2.93229 | 0.55262 | 0.56028 | 0.557036 | Not Significant | NS | #N/A |
| 1107 | G1T5N7 | Granulin precursor OS=Oryctolagus cuniculus OX=9 | GRN | RABBIT | -1.05847 | -0.10879 | -0.5518 | 0.70679 | -0.77927 | 0.30661 | 1.84072 | 1.69271 | 0.44123 | -1.68263 | 0.878033 | 1.24645 | 1.35096 | 0.24786 | 0.20401 | 0.29829 | 0.28553 | 8 | 2.93229 | 0.55262 | 1.24055 | 1.056033 | Not Significant | NS | #N/A |
| 2655 | U3KPF0 | #N/A | CPNE3 | RABBIT | #N/A | #N/A | #N/A | -0.50859 | -0.01338 | 0.32887 | 1.98074 | #N/A | -0.4649 | #N/A | 0.875888 | 0.92385 | 1.06046 | 0.4237 | 0.33019 | 0.38008 | 0.36106 | 3 | 2.93229 | 0.55262 | 1.07864 | 0.818632 | Not Significant | NS | #N/A |
| 2160 | G1STU0 | #N/A | WDR77 | RABBIT | #N/A | #N/A | -0.89769 | #N/A | -0.27996 | #N/A | 1.08924 | 0.20221 | -0.42647 | 0.27934 | 0.874898 | 1.73853 | 1.54315 | 0.15711 | 0.16712 | 0.23659 | 0.26191 | 4 | 2.93229 | 0.55262 | 0.33767 | 0.428588 | Not Significant | NS | #N/A |
| 2173 | G1SWI6 | #N/A | RPL9 | RABBIT | #N/A | #N/A | -0.3321 | -0.05764 | -1.44821 | 0.0571 | #N/A | 0.49806 | 0.59436 | -0.10599 | 0.87353 | 2.13977 | 1.84478 | 0.08534 | 0.10261 | 0.17453 | 0.20198 | 5 | 2.93229 | 0.55262 | 0.2857 | 0.384368 | Not Significant | NS | #N/A |
| 739 | G1TH06 | RAN binding protein 1 OS=Oryctolagus cuniculus O | RANBP1 | RABBIT | -0.28676 | 0.14726 | -2.03187 | 0.21177 | -1.76109 | 0.12395 | -0.044 | -0.02037 | 0.36103 | 0.22461 | 0.873184 | 1.79643 | 1.81215 | 0.11015 | 0.09749 | 0.19806 | 0.19688 | 8 | 2.93229 | 0.55262 | 0.59065 | 0.580451 | Not Significant | NS | #N/A |

| | | | | | | | | | | | | | | | | | | | | | | | | | | | | | |
|---|---|---|---|---|---|---|---|---|---|---|---|---|---|---|---|---|---|---|---|---|---|---|---|---|---|---|---|---|---|
| 415 | G1SFM1 | Phytanoyl-CoA 2-hydroxylase interacting protein lik | PHYHIPL | RABBIT | -1.78612 | -1.25052 | -0.07364 | 0.37831 | -0.23485 | 0.83641 | -0.24685 | 0.1571 | 0.33378 | 0.30868 | 0.871189 | 1.99779 | 1.95565 | 0.08079 | 0.07654 | 0.1731 | 0.18101 | 8 | 2.93229 | 0.55262 | 0.47541 | 0.496117 | Not Significant | NS | #N/A |
| 2372 | G1SP25 | #N/A | GNA14 | RABBIT | #N/A | #N/A | -0.61829 | -0.33707 | #N/A | 0.84328 | #N/A | 0.33452 | #N/A | 0 | 0.870278 | 2.6103 | 1.6335 | 0.07966 | 0.15405 | 0.17277 | 0.25312 | 3 | 2.93229 | 0.55262 | 0.13339 | 0.34061 | Not Significant | NS | #N/A |
| 2561 | A0A5F9DN52 | #N/A | RGS6 | RABBIT | #N/A | #N/A | #N/A | -0.46326 | #N/A | 0.40562 | #N/A | #N/A | #N/A | #N/A | 0.868879 | #N/A | 0.82648 | #N/A | 0.4704 | #N/A | 0.42992 | 0 | 2.93229 | 0.55262 | #N/A | 0.552617 | Not Significant | NS | #N/A |
| 1713 | G1TRC0 | #N/A | #N/A | RABBIT | #N/A | #N/A | -0.25199 | 0.15452 | -2.51402 | 0.60482 | -1.67216 | 0.7879 | -0.21216 | 0.48237 | 0.868652 | 1.01778 | 1.13469 | 0.34805 | 0.28604 | 0.34597 | 0.33729 | 6 | 2.93229 | 0.55262 | 1.36579 | 1.098845 | Not Significant | NS | #N/A |
| 2696 | G1T026 | #N/A | #N/A | RABBIT | #N/A | #N/A | #N/A | -0.03891 | #N/A | 4.65554 | -0.42648 | -0.34462 | -0.01873 | 0.28251 | 0.868556 | 0.36756 | 0.4641 | 0.73183 | 0.6568 | 0.49265 | 0.4985 | 4 | 2.93229 | 0.55262 | 4.65333 | 2.918768 | Not Significant | NS | #N/A |
| 387 | G1SSX2 | HECT-type E3 ubiquitin transferase OS=Oryctolagus | HUWE1 | RABBIT | 0.09509 | -2.62375 | -0.47421 | 0.892 | -0.47975 | -0.90973 | 1.25135 | 0.67082 | -0.08343 | 0.80549 | 0.865025 | 1.24216 | 1.34413 | 0.24935 | 0.20614 | 0.2994 | 0.28711 | 8 | 2.93229 | 0.55262 | 1.21239 | 1.035423 | Not Significant | NS | #N/A |
| 636 | A0A5F9C5Q7 | Thioredoxin like 1 OS=Oryctolagus cuniculus OX=99 | TXNL1 | RABBIT | -1.73305 | -1.23155 | 0.18705 | -0.10521 | -0.48156 | 0.02775 | -0.0164 | 0.47751 | 0.03006 | 0.43652 | 0.863953 | 2.3205 | 2.155 | 0.04888 | 0.05432 | 0.14906 | 0.15405 | 8 | 2.93229 | 0.55262 | 0.34654 | 0.401816 | Not Significant | NS | #N/A |
| 1212 | A0A2Z6DUF7 | Dehydrogenase/reductase SDR family member 11 ( | DHRS11 | RABBIT | -0.80609 | -1.34129 | 0.71948 | -1.65052 | 0.68953 | 1.09949 | -1.69517 | 1.08498 | -0.54883 | 1.98601 | 0.863077 | 1.03949 | 1.14948 | 0.32897 | 0.27489 | 0.33706 | 0.33144 | 8 | 2.93229 | 0.55262 | 1.72346 | 1.40941 | Not Significant | NS | #N/A |
| 825 | G1TX94 | Matrin 3 OS=Oryctolagus cuniculus OX=9986 GN=M | MATR3 | RABBIT | -2.72147 | 0.04955 | 0.23523 | 1.94941 | -1.28644 | 0.09839 | -0.06458 | 1.24603 | -0.34443 | 1.60363 | 0.862551 | 0.98755 | 1.09762 | 0.3523 | 0.29596 | 0.34782 | 0.34518 | 8 | 2.93229 | 0.55262 | 1.90718 | 1.543856 | Not Significant | NS | #N/A |
| 665 | A0A5F9DCT4 | Growth arrest specific 7 OS=Oryctolagus cuniculus | GAS7 | RABBIT | 0.01166 | -1.86764 | -1.10883 | 1.12655 | -0.6381 | 0.75513 | -0.91106 | 0.49797 | 1.12657 | #N/A | 0.86242 | 1.23826 | 1.33827 | 0.25553 | 0.21064 | 0.30172 | 0.29031 | 7 | 2.93229 | 0.55262 | 1.07796 | 0.922866 | Not Significant | NS | #N/A |
| 1874 | G1SZ47 | #N/A | RPS23 | RABBIT | #N/A | -2.05495 | -0.07159 | -0.36345 | -0.26394 | 0.01804 | 0.13387 | 0.20769 | 0 | 0.50576 | 0.861559 | 2.06757 | 1.94709 | 0.07749 | 0.08033 | 0.17105 | 0.1834 | 7 | 2.93229 | 0.55262 | 0.38587 | 0.435097 | Not Significant | NS | #N/A |
| 522 | G1SED9 | Eukaryotic translation initiation factor 3 subunit L O | EIF3L EIF3 | RABBIT | -0.91843 | -1.82047 | -0.43046 | -0.01552 | -0.31557 | 0.32943 | 0.04245 | 0.16519 | 0.30538 | -0.04212 | 0.860155 | 2.65661 | 2.33239 | 0.02896 | 0.03984 | 0.12702 | 0.13235 | 8 | 2.93229 | 0.55262 | 0.26208 | 0.34001 | Significant | Up in Mtub | EIF3L EIF3EIP EIF3S6IP |
| 2468 | G1SMP1 | #N/A | #N/A | RABBIT | #N/A | #N/A | #N/A | -0.44732 | #N/A | -0.62861 | #N/A | -0.06437 | 1.39657 | 0.94493 | 0.859456 | 0.83165 | 0.91538 | 0.46662 | 0.39567 | 0.39969 | 0.39935 | 3 | 2.93229 | 0.55262 | 0.8544 | 0.705229 | Not Significant | NS | #N/A |
| 2234 | A0A5F9CUG5 | #N/A | RHOB | RABBIT | #N/A | #N/A | #N/A | -0.20083 | #N/A | 2.06534 | 0 | 1.02849 | -0.46254 | #N/A | 0.858652 | 0.68181 | 0.80297 | 0.54428 | 0.45295 | 0.43035 | 0.4223 | 3 | 2.93229 | 0.55262 | 1.26881 | 0.914803 | Not Significant | NS | #N/A |
| 2091 | G1T8H5 | #N/A | POLR2C | RABBIT | #N/A | #N/A | #N/A | -0.84614 | -0.20584 | #N/A | 0.6383 | 0.35768 | 0 | #N/A | 0.857985 | 2.54333 | 1.60665 | 0.08443 | 0.15982 | 0.17453 | 0.25503 | 3 | 2.93229 | 0.55262 | 0.13656 | 0.342216 | Not Significant | NS | #N/A |
| 2527 | G1T8S4 | #N/A | CLUH | RABBIT | #N/A | #N/A | #N/A | -0.69319 | #N/A | 0.32878 | #N/A | #N/A | #N/A | 0 | 0.857585 | 3.01186 | 1.07304 | 0.20408 | 0.34464 | 0.27183 | 0.37021 | 1 | 2.93229 | 0.55262 | 0.05405 | 0.425829 | Not Significant | NS | #N/A |
| 1438 | G1TRN0 | Argonaute RISC component 1 OS=Oryctolagus cuni | AGO1 | RABBIT | -0.90313 | #N/A | 0.13368 | 0.81212 | 0.02359 | #N/A | 1.95377 | -0.16824 | 1.91993 | -0.21487 | 0.856083 | 1.20824 | 1.30868 | 0.27241 | 0.22331 | 0.30973 | 0.29936 | 6 | 2.93229 | 0.55262 | 1.00404 | 0.855849 | Not Significant | NS | #N/A |
| 819 | A0A5F9C1H6 | Myosin VA OS=Oryctolagus cuniculus OX=9986 GN | MYO5A | RABBIT | -1.15821 | -2.38975 | 1.76591 | -0.16144 | #N/A | 1.05851 | -0.05437 | -0.06437 | 0.07507 | 0.82924 | 0.854685 | 1.04562 | 1.15847 | 0.33049 | 0.27377 | 0.33706 | 0.33128 | 7 | 2.93229 | 0.55262 | 1.48475 | 1.209559 | Not Significant | NS | #N/A |
| 1867 | A0A5F9CWB3 | #N/A | CTBP2 | RABBIT | #N/A | -0.7616 | -1.51534 | 0.04392 | -0.01338 | 0.80962 | -0.81025 | 0.7796 | -0.07964 | 0.76338 | 0.854142 | 1.75187 | 1.74007 | 0.12326 | 0.11268 | 0.2103 | 0.21183 | 7 | 2.93229 | 0.55262 | 0.52825 | 0.535446 | Not Significant | NS | #N/A |
| 1531 | A5U0Y9 | ATP-dependent DNA helicase OS=Mycobacterium t | #N/A | MT | 0.08824 | -1.37124 | -0.37626 | -0.13422 | 0.22667 | #N/A | #N/A | #N/A | #N/A | 0.54049 | 0.853848 | 1.22932 | 1.14216 | 0.28632 | 0.2913 | 0.31788 | 0.34138 | 4 | 2.93229 | 0.55262 | 0.40202 | 0.465721 | Not Significant | NS | #N/A |
| 548 | A0A5F9D0Q9 | RNA transcription, translation and transport factor p | #N/A | RABBIT | -0.61157 | #N/A | #N/A | #N/A | #N/A | #N/A | #N/A | 0.48245 | 0 | #N/A | 0.852792 | 2.04107 | 1.04772 | 0.29002 | 0.35485 | 0.3201 | 0.37672 | 1 | 2.93229 | 0.55262 | 0.11638 | 0.44168 | Not Significant | NS | #N/A |
| 984 | G1TGZ5 | diphosphoinositol-polyphosphate diphosphatase C | NUDT11 | RABBIT | -1.37611 | #N/A | 0.17065 | #N/A | #N/A | 0.99195 | -0.2239 | -0.02815 | #N/A | #N/A | 0.849364 | 1.12593 | 1.18309 | 0.34213 | 0.28201 | 0.3443 | 0.33531 | 3 | 2.93229 | 0.55262 | 0.68288 | 0.618491 | Not Significant | NS | #N/A |
| 722 | A0A5F9CGE5 | Protein transport protein SEC23 OS=Oryctolagus cu | SEC23A | RABBIT | -1.04981 | -0.21841 | -0.32824 | 0.83078 | -1.39036 | -0.75382 | 0.81473 | 0.92309 | 0.68854 | 0.41623 | 0.848965 | 1.73114 | 1.75023 | 0.12167 | 0.10805 | 0.20964 | 0.20706 | 8 | 2.93229 | 0.55262 | 0.60125 | 0.588207 | Not Significant | NS | #N/A |
| 1706 | A0A5F9CMJ3 | #N/A | EIF3C EIF3 | RABBIT | #N/A | -0.97339 | -0.89486 | #N/A | -0.07504 | 0.11271 | #N/A | -0.06437 | 0.22637 | 0.52629 | 0.848015 | 3.01126 | 2.06187 | 0.02971 | 0.07345 | 0.12757 | 0.17651 | 5 | 2.93229 | 0.55262 | 0.13595 | 0.28998 | Not Significant | NS | #N/A |
| 1964 | A0A5F9CV64 | #N/A | SNX4 | RABBIT | #N/A | -1.31125 | -0.58123 | 0.22126 | #N/A | -0.0236 | -0.01497 | 0.31015 | 0.01497 | 1.16744 | 0.84787 | 1.91191 | 1.77234 | 0.10443 | 0.11035 | 0.19378 | 0.20933 | 6 | 2.93229 | 0.55262 | 0.36874 | 0.429106 | Not Significant | NS | #N/A |
| 1098 | G1TWW0 | Leucine carboxyl methyltransferase 1 OS=Oryctolag | LCMT1 | RABBIT | #N/A | -0.78583 | -1.08696 | 1.57023 | -0.02247 | 1.84435 | -0.53832 | -0.06437 | 1.84307 | 0.72477 | 0.843158 | 1.11133 | 1.21814 | 0.30313 | 0.25131 | 0.32493 | 0.31847 | 7 | 2.93229 | 0.55262 | 1.27915 | 1.064656 | Not Significant | NS | #N/A |
| 842 | A0A5F9C8J1 | 3'(2'),5'-bisphosphate nucleotidase 1 OS=Oryctolag | BPNT1 | RABBIT | -0.77656 | 0.54524 | -0.45736 | -1.71089 | 0.32835 | 0.75838 | #N/A | -0.06437 | 1.78554 | -0.7704 | 0.841532 | 1.26198 | 1.35308 | 0.24738 | 0.20602 | 0.29795 | 0.28711 | 7 | 2.93229 | 0.55262 | 0.98816 | 0.859576 | Not Significant | NS | #N/A |
| 2265 | A0A5F9CG26 | #N/A | SCAMP3 | RABBIT | #N/A | #N/A | #N/A | -1.73964 | -0.01338 | 0.12109 | 0.60236 | 0.31604 | -1.12153 | -0.09699 | 0.840707 | 1.25055 | 1.28524 | 0.26643 | 0.23496 | 0.30603 | 0.30526 | 5 | 2.93229 | 0.55262 | 0.64564 | 0.611252 | Not Significant | NS | #N/A |
| 1387 | G1SUM6 | Carboxypeptidase E OS=Oryctolagus cuniculus OX= | #N/A | RABBIT | -1.3848 | 0.84418 | 0.17685 | #N/A | -1.38908 | 0.79577 | 1.24262 | -0.07057 | -0.36177 | #N/A | 0.839724 | 1.24133 | 1.33089 | 0.26081 | 0.21619 | 0.30397 | 0.29349 | 6 | 2.93229 | 0.55262 | 0.91523 | 0.796189 | Not Significant | NS | #N/A |
| 874 | B7NZS8 | Small ribosomal subunit protein uS4 OS=Oryctolag | RPS9 | RABBIT | -2.86299 | -1.70787 | 0.51146 | -0.0631 | 0.00156 | -0.35849 | -0.00033 | 0.27259 | 0.00033 | 0.15777 | 0.838563 | 1.30887 | 1.39826 | 0.22692 | 0.18975 | 0.28703 | 0.27623 | 8 | 2.93229 | 0.55262 | 1.02617 | 0.899153 | Not Significant | NS | #N/A |
| 554 | G1SX42 | RAB GTPase activating protein 1 OS=Oryctolagus cu | RABGAP1 | RABBIT | 0.06742 | -2.69341 | -0.17282 | -0.09692 | 0.0259 | -0.3468 | -0.21319 | 0.39599 | 0.69288 | 0.79179 | 0.838102 | 1.44453 | 1.51602 | 0.18659 | 0.15788 | 0.26042 | 0.25465 | 8 | 2.93229 | 0.55262 | 0.84155 | 0.764053 | Not Significant | NS | #N/A |
| 875 | G1TKY3 | Calpain-2 catalytic subunit OS=Oryctolagus cunicu | CAPN2 | RABBIT | 0.07037 | 2.15892 | -2.17077 | -0.15092 | -1.06657 | -0.05871 | 1.13892 | -0.23386 | 1.45438 | 0.72879 | 0.837694 | 1.06234 | 1.16809 | 0.3191 | 0.26761 | 0.33327 | 0.32868 | 8 | 2.93229 | 0.55262 | 1.55448 | 1.285761 | Not Significant | NS | #N/A |
| 2416 | G1SGS7 | #N/A | UFL1 | RABBIT | #N/A | #N/A | -0.20763 | #N/A | -0.39326 | 0.68956 | 0.37828 | #N/A | #N/A | #N/A | 0.834359 | 4.60429 | 1.42703 | 0.04408 | 0.21369 | 0.14581 | 0.29197 | 2 | 2.93229 | 0.55262 | 0.03284 | 0.341851 | Not Significant | NS | #N/A |
| 1516 | A0A5F9DMU0 | Kinesin-like protein OS=Oryctolagus cuniculus OX= | KIF2A | RABBIT | -0.4796 | 0.20103 | 0.16744 | -0.8537 | 0.40511 | 1.83393 | -0.1616 | 1.27759 | -0.46264 | 1.12008 | 0.833414 | 1.66411 | 1.69125 | 0.13466 | 0.11906 | 0.21791 | 0.21763 | 8 | 2.93229 | 0.55262 | 0.62705 | 0.607083 | Not Significant | NS | #N/A |
| 1099 | A0A5F9CW48 | ENAH actin regulator OS=Oryctolagus cuniculus OX | ENAH | RABBIT | 0.01166 | #N/A | -1.7146 | -0.55373 | -0.95371 | 0.12991 | -0.84489 | -0.00216 | 0.48385 | 0.38352 | 0.832641 | 2.00342 | 1.88482 | 0.08519 | 0.08902 | 0.17453 | 0.1902 | 7 | 2.93229 | 0.55262 | 0.38385 | 0.433673 | Not Significant | NS | #N/A |
| 617 | P10160 | Eukaryotic translation initiation factor 5A-1 OS=Ory | EIF5A | RABBIT | -1.59773 | -0.73823 | -0.17258 | 0.28033 | -0.2924 | 0.51881 | -0.30814 | 0.21465 | 0.37881 | 0.83237 | 0.831422 | 2.24964 | 2.08349 | 0.0546 | 0.06148 | 0.15412 | 0.16292 | 8 | 2.93229 | 0.55262 | 0.34147 | 0.398107 | Not Significant | NS | #N/A |
| 1689 | G1T8W7 | #N/A | ATP6V0A1 | RABBIT | #N/A | -2.08648 | 0.37328 | -1.68726 | 0.52464 | 0.51945 | 0 | 0.60402 | -0.26892 | -0.29942 | 0.829982 | 1.30603 | 1.38707 | 0.23282 | 0.19576 | 0.29083 | 0.28063 | 7 | 2.93229 | 0.55262 | 0.89747 | 0.795659 | Not Significant | NS | #N/A |
| 778 | Q8MK67 | Phosphatidylethanolamine-binding protein 1 OS=O | PEBP1 PBF | RABBIT | -0.16899 | #N/A | #N/A | #N/A | #N/A | #N/A | 0 | #N/A | 1.32159 | #N/A | 0.829783 | 0.725 | 0.85078 | 0.60064 | 0.44359 | 0.45131 | 0.41812 | 1 | 2.93229 | 0.55262 | 0.8733 | 0.634168 | Not Significant | NS | #N/A |
| 1133 | A0A5F9CBG6 | Cold shock domain containing E1 OS=Oryctolagus | CSDE1 | RABBIT | -2.33005 | -1.04225 | -0.19308 | 2.28845 | -1.0049 | 1.00729 | -0.1555 | 0.28936 | 0.1555 | 0.5603 | 0.827758 | 1.04582 | 1.15042 | 0.32621 | 0.27451 | 0.33572 | 0.33144 | 8 | 2.93229 | 0.55262 | 1.56615 | 1.294295 | Not Significant | NS | #N/A |
| 1532 | G1U2E5 | Asparagine synthetase [glutamine-hydrolyzing] OS= | ASNS | RABBIT | -0.73432 | #N/A | #N/A | 2.44129 | -1.16765 | 1.28671 | #N/A | 3.20691 | -0.46759 | 0 | 0.826737 | 0.60755 | 0.72893 | 0.57002 | 0.48701 | 0.43972 | 0.43895 | 5 | 2.93229 | 0.55262 | 3.17438 | 2.205209 | Not Significant | NS | #N/A |
| 737 | G1TLY5 | N-acetylneuraminate synthase OS=Oryctolagus cu | NANS | RABBIT | -0.57626 | -0.2918 | -0.50805 | -0.05764 | -1.51029 | 0.63127 | 0.02975 | 0.15926 | #N/A | 0.12936 | 0.826217 | 2.71347 | 2.21802 | 0.03005 | 0.05104 | 0.12757 | 0.15114 | 7 | 2.93229 | 0.55262 | 0.20603 | 0.30835 | Not Significant | NS | #N/A |
| 1346 | G1T015 | Nucleolus and neural progenitor protein OS=Orycto | NEPRO | RABBIT | -1.15399 | 0.19782 | #N/A | #N/A | #N/A | #N/A | #N/A | 0.34466 | #N/A | #N/A | 0.822744 | 0.70278 | 0.83681 | 0.61002 | 0.45054 | 0.45404 | 0.42119 | 1 | 2.93229 | 0.55262 | 0.9137 | 0.644442 | Not Significant | NS | #N/A |
| 2677 | Q9Q8J0 | #N/A | m110L | RABBIT | #N/A | #N/A | #N/A | -0.18441 | #N/A | 1.27605 | 0 | #N/A | #N/A | #N/A | 0.822439 | 0.74423 | 0.85343 | 0.59269 | 0.44229 | 0.448 | 0.41781 | 1 | 2.93229 | 0.55262 | 0.81415 | 0.619126 | Not Significant | NS | #N/A |
| 2808 | G1SIA4 | #N/A | #N/A | RABBIT | #N/A | #N/A | #N/A | #N/A | -0.44215 | -0.00024 | 1.59297 | -0.41048 | 0.7164 | 0.00024 | 0.82193 | 0.94944 | 0.97344 | 0.39616 | 0.36307 | 0.36919 | 0.3819 | 4 | 2.93229 | 0.55262 | 0.62453 | 0.594112 | Not Significant | NS | #N/A |
| 1430 | G1TVN8 | G protein pathway suppressor 2 OS=Oryctolagus cu | GPS2 | RABBIT | -0.81553 | #N/A | #N/A | #N/A | #N/A | #N/A | -0.00745 | -0.05692 | #N/A | 0.08352 | 0.821916 | 9.99221 | 1.23797 | 0.00987 | 0.27139 | 0.09414 | 0.33018 | 2 | 2.93229 | 0.55262 | 0.00507 | 0.330593 | Not Significant | NS | #N/A |
| 2563 | G1TKL4 | #N/A | TANGO2 | RABBIT | #N/A | #N/A | #N/A | -0.82326 | #N/A | 0.76562 | #N/A | #N/A | 1.07289 | -1.85023 | 0.819345 | 0.44174 | 0.60517 | 0.70185 | 0.57183 | 0.48408 | 0.46955 | 2 | 2.93229 | 0.55262 | 2.58029 | 1.37482 | Not Significant | NS | #N/A |
| 584 | A0A5F9CL93 | RAP1 GTPase activating protein OS=Oryctolagus cu | RAP1GAP | RABBIT | -0.95392 | 0.08875 | -1.04734 | 1.14853 | -0.16202 | 1.63024 | 0.10907 | 0.69821 | 1.58045 | -0.86139 | 0.816515 | 1.32519 | 1.40632 | 0.2217 | 0.18741 | 0.28335 | 0.27558 | 8 | 2.93229 | 0.55262 | 0.94909 | 0.842749 | Not Significant | NS | #N/A |
| 745 | G1SXJ6 | Cullin 4A OS=Oryctolagus cuniculus OX=9986 GN= | CUL4A | RABBIT | 0.06718 | #N/A | #N/A | #N/A | #N/A | 4.05787 | -0.5759 | -0.20745 | -0.05551 | 1.19344 | 0.815311 | 0.39262 | 0.48998 | 0.71463 | 0.63927 | 0.48903 | 0.49195 | 4 | 2.93229 | 0.55262 | 3.59361 | 2.307302 | Not Significant | NS | #N/A |
| 1372 | A0A5F9DDW9 | Eukaryotic translation initiation factor 4E OS=Oryct | #N/A | RABBIT | #N/A | -1.91852 | 0.17065 | 0.34244 | -0.39202 | 0.36477 | #N/A | #N/A | #N/A | #N/A | 0.814131 | 0.70804 | 0.81007 | 0.52997 | 0.44916 | 0.42625 | 0.42107 | 3 | 2.93229 | 0.55262 | 1.05769 | 0.808037 | Not Significant | NS | #N/A |
| 2429 | A0A5F9CSZ8 | #N/A | COMMD8 | RABBIT | #N/A | #N/A | -0.62437 | #N/A | -0.67064 | #N/A | 0.38586 | -0.23181 | 0.16745 | 0.34286 | 0.813591 | 3.84377 | 1.81397 | 0.0184 | 0.11297 | 0.11171 | 0.21215 | 4 | 2.93229 | 0.55262 | 0.05974 | 0.26822 | Not Significant | NS | #N/A |
| 1899 | A0A5F9DUH0 | #N/A | #N/A | RABBIT | #N/A | -1.06726 | 0.19006 | #N/A | #N/A | 1.41557 | #N/A | 0.62831 | -0.52853 | -0.01941 | 0.812585 | 1.1002 | 1.16075 | 0.333 | 0.28415 | 0.3379 | 0.33715 | 4 | 2.93229 | 0.55262 | 0.72733 | 0.653427 | Not Significant | NS | #N/A |
| 2448 | A0A5F9DFA4 | #N/A | #N/A | RABBIT | #N/A | #N/A | -0.05831 | #N/A | #N/A | 1.50265 | #N/A | #N/A | #N/A | 0 | 0.809634 | 0.62216 | 0.79058 | 0.64569 | 0.47414 | 0.46785 | 0.432 | 1 | 2.93229 | 0.55262 | 1.12898 | 0.69919 | Not Significant | NS | #N/A |
| 1070 | G1T9V6 | ubiquitinyl hydrolase 1 OS=Oryctolagus cuniculus ( | USP24 | RABBIT | #N/A | #N/A | 0.31344 | 0.00137 | -2.66331 | -4.03488 | -1.09758 | -0.12338 | 2.6787 | 2.70693 | 0.80879 | 0.44387 | 0.53019 | 0.6727 | 0.6089 | 0.47403 | 0.47955 | 6 | 2.93229 | 0.55262 | 6.22545 | 4.363175 | Not Significant | NS | #N/A |
| 2621 | A0A5F9CRM0 | #N/A | ACP2 | RABBIT | #N/A | #N/A | #N/A | -0.46525 | #N/A | #N/A | #N/A | 0.34324 | #N/A | #N/A | 0.808492 | #N/A | 0.76904 | #N/A | 0.49905 | #N/A | 0.44285 | 0 | 2.93229 | 0.55262 | #N/A | 0.552617 | Not Significant | NS | #N/A |
| 2673 | G1SNQ8 | #N/A | HEXB | RABBIT | #N/A | #N/A | #N/A | -0.40792 | #N/A | 1.6048 | -0.63444 | -0.66505 | 1.34645 | 0.35028 | 0.808327 | 0.69155 | 0.78186 | 0.52727 | 0.46016 | 0.42539 | 0.42509 | 4 | 2.93229 | 0.55262 | 1.13855 | 0.890704 | Not Significant | NS | #N/A |
| 2545 | A0A5F9D703 | #N/A | TPP1 | RABBIT | #N/A | #N/A | #N/A | -0.61287 | #N/A | 1.0434 | -0.58374 | -0.58711 | 0.52274 | 0.5726 | 0.80645 | 0.99541 | 0.99325 | 0.37587 | 0.35398 | 0.35851 | 0.37605 | 4 | 2.93229 | 0.55262 | 0.54697 | 0.549361 | Not Significant | NS | #N/A |
| 1394 | G1SHX1 | E2 ubiquitin-conjugating enzyme OS=Oryctolagus c | #N/A | RABBIT | #N/A | 0.61003 | -3.3742 | 0.32698 | -1.0221 | -0.70502 | 0.25042 | -0.06437 | 0.43946 | -0.22728 | 0.803466 | 0.96844 | 1.0751 | 0.36509 | 0.30775 | 0.354 | 0.35174 | 7 | 2.93229 | 0.55262 | 1.52959 | 1.241163 | Not Significant | NS | #N/A |
| 2557 | A0A375YI16 | #N/A | #N/A | MT | #N/A | #N/A | #N/A | -0.26196 | -0.90626 | 0.20432 | #N/A | 0.22901 | #N/A | #N/A | 0.80077 | 2.48388 | 1.31525 | 0.13098 | 0.24625 | 0.21539 | 0.31578 | 2 | 2.93229 | 0.55262 | 0.10393 | 0.37068 | Not Significant | NS | #N/A |
| 2166 | G1ST96 | #N/A | #N/A | RABBIT | #N/A | #N/A | -0.50218 | -1.47813 | 0.10563 | 0.19042 | -0.11902 | 0.3324 | -0.17257 | 0.62982 | 0.797102 | 2.04105 | 1.78594 | 0.08732 | 0.10801 | 0.17659 | 0.20706 | 6 | 2.93229 | 0.55262 | 0.28597 | 0.373506 | Not Significant | NS | #N/A |
| 2763 | G1TCW1 | #N/A | TFRC | RABBIT | #N/A | #N/A | #N/A | -0.82216 | #N/A | #N/A | 0.01319 | -0.06437 | #N/A | #N/A | 0.79657 | 11.8593 | 1.01224 | 0.05355 | 0.36959 | 0.15354 | 0.38394 | 1 | 2.93229 | 0.55262 | 0.00301 | 0.412849 | Not Significant | NS | #N/A |
| 2642 | G1SL17 | #N/A | MMP28 | RABBIT | #N/A | #N/A | #N/A | #N/A | -0.40372 | #N/A | #N/A | #N/A | #N/A | 0.39034 | 0.794065 | #N/A | 0.75532 | #N/A | 0.50611 | #N/A | 0.44683 | 0 | 2.93229 | 0.55262 | #N/A | 0.552617 | Not Significant | NS | #N/A |
| 1104 | G1U2M2 | Seizure related 6 homolog like 2 OS=Oryctolagus cu | SEZ6L2 | RABBIT | -0.38932 | #N/A | #N/A | #N/A | #N/A | 0.40098 | #N/A | #N/A | #N/A | #N/A | 0.790297 | #N/A | 0.75173 | #N/A | 0.50796 | #N/A | 0.44778 | 0 | 2.93229 | 0.55262 | #N/A | 0.552617 | Not Significant | NS | #N/A |
| 2208 | G1TFB5 | #N/A | #N/A | RABBIT | #N/A | #N/A | -0.5574 | #N/A | #N/A | 0.64267 | 0.75803 | -0.70703 | #N/A | #N/A | 0.788619 | 0.83841 | 0.88347 | 0.49004 | 0.41796 | 0.41038 | 0.40859 | 2 | 2.93229 | 0.55262 | 0.66357 | 0.597607 | Not Significant | NS | #N/A |
| 2659 | A0A5F9DRP3 | #N/A | PPP4R2 | RABBIT | #N/A | #N/A | #N/A | -0.21348 | #N/A | 0.15584 | #N/A | -0.22616 | #N/A | 1.79532 | 0.788483 | 0.63583 | 0.76526 | 0.58994 | 0.47911 | 0.44671 | 0.43349 | 2 | 2.93229 | 0.55262 | 1.15336 | 0.796215 | Not Significant | NS | #N/A |
| 1147 | G1TVJ8 | Paladin OS=Oryctolagus cuniculus OX=9986 GN=P. | PALD1 | RABBIT | #N/A | 0.43407 | #N/A | -0.5297 | -0.24963 | #N/A | 0.66911 | #N/A | #N/A | #N/A | 0.784198 | 1.36985 | 1.03784 | 0.30425 | 0.34752 | 0.32493 | 0.37193 | 2 | 2.93229 | 0.55262 | 0.24579 | 0.428202 | Not Significant | NS | #N/A |
| 2168 | G1TZ76 | #N/A | RPS27 | RABBIT | #N/A | #N/A | 0.07327 | -0.65903 | -1.35692 | 0.31721 | 0.29683 | 0.24952 | -0.28613 | 0.09738 | 0.782524 | 2.32519 | 1.88228 | 0.05903 | 0.09272 | 0.15506 | 0.19448 | 6 | 2.93229 | 0.55262 | 0.21236 | 0.324062 | Not Significant | NS | #N/A |
| 1331 | A0A5F9CBC6 | Calcium/calmodulin dependent protein kinase kina | CAMKK2 | RABBIT | #N/A | #N/A | -0.28758 | 0.19584 | -1.63156 | 1.12813 | -1.04148 | -0.00975 | -0.05461 | 1.0118 | 0.781252 | 1.17618 | 1.24604 | 0.28407 | 0.24444 | 0.31659 | 0.3144 | 6 | 2.93229 | 0.55262 | 0.82724 | 0.737089 | Not Significant | NS | #N/A |
| 343 | G1SRB6 | Peptidylprolyl isomerase D OS=Oryctolagus cunicu | PPID | RABBIT | -0.03395 | -0.46399 | 0.21626 | -0.55415 | -0.4705 | 1.48968 | -0.22143 | 0.0773 | 0.53563 | 0.71357 | 0.780215 | 2.36643 | 2.09393 | 0.0455 | 0.06038 | 0.14686 | 0.16242 | 8 | 2.93229 | 0.55262 | 0.27176 | 0.34709 | Not Significant | NS | #N/A |
| 575 | G1SYC9 | Ras-related protein Rab-8B OS=Oryctolagus cunicu | RAB8B | RABBIT | -0.73646 | -2.22971 | 1.605 | -1.70131 | 0.53586 | 1.02729 | 0.01325 | -0.25366 | 0.5915 | -0.01325 | 0.778351 | 1.04517 | 1.14126 | 0.3265 | 0.27814 | 0.33572 | 0.3326 | 8 | 2.93229 | 0.55262 | 1.3865 | 1.162837 | Not Significant | NS | #N/A |
| 1316 | A0A5F9DLU2 | Tight junction protein 1 OS=Oryctolagus cuniculus ( | TJP1 | RABBIT | -0.36538 | #N/A | #N/A | #N/A | #N/A | #N/A | -0.41369 | 0.97478 | 1.08788 | 0 | 0.777623 | 0.94502 | 0.94034 | 0.41437 | 0.38374 | 0.37703 | 0.39263 | 3 | 2.93229 | 0.55262 | 0.54168 | 0.547086 | Not Significant | NS | #N/A |
| 918 | Q6PNB6 | Guanine nucleotide-binding protein subunit beta-5 | GNB5 | RABBIT | #N/A | -0.86342 | -0.25047 | 0.29835 | -0.5979 | 1.01001 | -0.1932 | -0.06437 | 0.84692 | 0.51933 | 0.777099 | 2.21998 | 1.94412 | 0.06188 | 0.08073 | 0.15739 | 0.18391 | 7 | 2.93229 | 0.55262 | 0.2723 | 0.355054 | Not Significant | NS | #N/A |
| 1771 | A0A5F9D432 | #N/A | RAB37 | RABBIT | #N/A | #N/A | #N/A | -0.76869 | #N/A | 0 | #N/A | #N/A | #N/A | 0.0144 | 0.775893 | 62.2184 | 0.98685 | 0.01023 | 0.38048 | 0.09529 | 0.39063 | 1 | 2.93229 | 0.55262 | 0.0001 | 0.41211 | Not Significant | NS | #N/A |
| 1984 | A0A5F9D7L9 | #N/A | LUC7L3 | RABBIT | #N/A | -0.53685 | -0.35938 | 0.59772 | -0.86482 | 0 | 0.69548 | 1.58836 | 1.33009 | -1.19273 | 0.775075 | 1.2267 | 1.30127 | 0.25959 | 0.22254 | 0.30317 | 0.29903 | 7 | 2.93229 | 0.55262 | 0.88716 | 0.788391 | Not Significant | NS | #N/A |
| 2276 | A0A5F9C9V5 | #N/A | UCHL5 | RABBIT | #N/A | #N/A | -1.6594 | 0.31069 | -0.39044 | 0.71494 | -0.28331 | -0.31712 | 0.60832 | 0.25275 | 0.774836 | 1.5183 | 1.48652 | 0.17974 | 0.17156 | 0.25448 | 0.26461 | 6 | 2.93229 | 0.55262 | 0.48832 | 0.509428 | Not Significant | NS | #N/A |
| 2050 | A0A5S8H3V0 | #N/A | EIF2B5 | RABBIT | #N/A | -2.14713 | 0.04151 | 1.24327 | -1.48248 | -0.08321 | 0.17199 | -0.06437 | 0.43876 | 0.47723 | 0.774291 | 1.13301 | 1.22036 | 0.29452 | 0.25051 | 0.32123 | 0.31769 | 7 | 2.93229 | 0.55262 | 1.03783 | 0.894584 | Not Significant | NS | #N/A |
| 1959 | A0A5F9CIS2 | #N/A | SAFB | RABBIT | #N/A | -0.31973 | #N/A | #N/A | #N/A | #N/A | #N/A | 0.45318 | #N/A | #N/A | 0.772915 | #N/A | 0.7352 | #N/A | 0.5166 | #N/A | 0.45076 | 0 | 2.93229 | 0.55262 | #N/A | 0.552617 | Not Significant | NS | #N/A |
| 2377 | G1STC7 | #N/A | FGF12 FGF | RABBIT | #N/A | #N/A | -0.51537 | 0.48051 | -1.89483 | 0.98098 | -0.47901 | 0.42484 | 0.47901 | -0.76393 | 0.771611 | 1.16342 | 1.23206 | 0.28883 | 0.24937 | 0.31946 | 0.31705 | 6 | 2.93229 | 0.55262 | 0.82475 | 0.735417 | Not Significant | NS | #N/A |
| 2278 | G1T0X2 | #N/A | #N/A | RABBIT | #N/A | #N/A | -3.15753 | -0.11979 | 1.28061 | -0.30757 | 2.23074 | 0.18089 | -1.64635 | 0.06215 | 0.769541 | 0.60738 | 0.70991 | 0.56586 | 0.49587 | 0.43824 | 0.4412 | 6 | 2.93229 | 0.55262 | 3.00986 | 2.203198 | Not Significant | NS | #N/A |
| 2041 | G1U9R5 | #N/A | GYS2 | RABBIT | #N/A | #N/A | #N/A | -0.41239 | #N/A | #N/A | #N/A | #N/A | 0.35475 | #N/A | 0.767144 | #N/A | 0.72971 | #N/A | 0.5195 | #N/A | 0.45188 | 0 | 2.93229 | 0.55262 | #N/A | 0.552617 | Not Significant | NS | #N/A |
| 855 | A0A5F9CYH4 | Contactin-5 OS=Oryctolagus cuniculus OX=9986 G | CNTN5 | RABBIT | 0.52798 | #N/A | #N/A | -2.18903 | #N/A | #N/A | #N/A | -0.06437 | #N/A | #N/A | 0.766159 | 0.32561 | 0.53825 | 0.7996 | 0.61942 | 0.51361 | 0.48406 | 1 | 2.93229 | 0.55262 | 3.69107 | 1.350742 | Not Significant | NS | #N/A |
| 125 | G1TKC3 | Tubulin alpha chain OS=Oryctolagus cuniculus OX= | #N/A | RABBIT | -1.86111 | 4.25089 | 5.10255 | -0.76799 | -2.26 | -0.05732 | 5.03024 | -0.00705 | #N/A | #N/A | 0.762424 | 0.3139 | 0.37841 | 0.76422 | 0.71396 | 0.50299 | 0.5176 | 6 | 2.93229 | 0.55262 | 11.0613 | 7.611519 | Not Significant | NS | #N/A |
| 1507 | A0A5F9CGX9 | PKHD1 ciliary IPT domain containing fibrocystin/pol | PKHD1 | RABBIT | 0.01166 | #N/A | 0.3547 | -2.77678 | #N/A | -0.56013 | #N/A | #N/A | #N/A | 0.47707 | 0.761941 | 0.56978 | 0.71615 | 0.6087 | 0.5011 | 0.45404 | 0.44378 | 3 | 2.93229 | 0.55262 | 2.14589 | 1.358344 | Not Significant | NS | #N/A |
| 2819 | A0A5F9DB75 | #N/A | LAMP5 | RABBIT | #N/A | #N/A | #N/A | #N/A | -0.38657 | 0.37318 | #N/A | #N/A | #N/A | #N/A | 0.759752 | #N/A | 0.72268 | #N/A | 0.52322 | #N/A | 0.45286 | 0 | 2.93229 | 0.55262 | #N/A | 0.552617 | Not Significant | NS | #N/A |
| 2362 | P71838 | #N/A | #N/A | MT | #N/A | #N/A | -0.29444 | #N/A | #N/A | #N/A | 0.46509 | #N/A | #N/A | #N/A | 0.759522 | #N/A | 0.72246 | #N/A | 0.52334 | #N/A | 0.45286 | 0 | 2.93229 | 0.55262 | #N/A | 0.552617 | Not Significant | NS | #N/A |
| 1945 | G1TRL5 | #N/A | EIF5B | RABBIT | #N/A | -0.72648 | #N/A | #N/A | #N/A | 0 | #N/A | #N/A | 0.06429 | #N/A | 0.758629 | 13.625 | 0.9643 | 0.04664 | 0.39037 | 0.14885 | 0.39558 | 1 | 2.93229 | 0.55262 | 0.00207 | 0.412609 | Not Significant | NS | #N/A |
| 2856 | G1SY65 | #N/A | CCDC25 | RABBIT | #N/A | #N/A | #N/A | #N/A | -0.23361 | 1.57348 | #N/A | #N/A | -0.06123 | 0.06123 | 0.758101 | 0.72106 | 0.80528 | 0.54577 | 0.45771 | 0.43066 | 0.42417 | 2 | 2.93229 | 0.55262 | 0.82903 | 0.664701 | Not Significant | NS | #N/A |
| 1407 | A0A5F9CP77 | Coiled-coil domain containing 120 OS=Oryctolagus | CCDC120 | RABBIT | 0.89815 | #N/A | #N/A | -0.66692 | -0.77643 | #N/A | 2.35985 | 0.40645 | -0.46094 | 0 | 0.758075 | 0.87915 | 0.98874 | 0.41956 | 0.35198 | 0.37892 | 0.37531 | 5 | 2.93229 | 0.55262 | 1.27462 | 1.007719 | Not Significant | NS | #N/A |
| 430 | G1SKN4 | Synaptic vesicle glycoprotein 2A OS=Oryctolagus cu | SV2A | RABBIT | -1.51897 | -2.18339 | 0.94245 | -1.50195 | 0.89691 | 0.44441 | 0.13576 | 0.73823 | -0.13576 | -0.76021 | 0.75748 | 1.06698 | 1.1577 | 0.31712 | 0.27165 | 0.33233 | 0.33018 | 8 | 2.93229 | 0.55262 | 1.26 | 1.070261 | Not Significant | NS | #N/A |
| 2263 | A0A5F9CMD8 | #N/A | #N/A | RABBIT | #N/A | #N/A | 0.17065 | -0.6248 | -1.06048 | 0.93653 | #N/A | 0.08205 | -0.10405 | 0.05606 | 0.747528 | 1.82271 | 1.57528 | 0.12796 | 0.15416 | 0.21348 | 0.25312 | 5 | 2.93229 | 0.55262 | 0.28834 | 0.386034 | Not Significant | NS | #N/A |
| 1008 | A0A5F9DNH7 | Pleckstrin and Sec7 domain containing 3 OS=Orycto | PSD3 | RABBIT | -2.00506 | -0.58763 | -0.59753 | 0.77411 | #N/A | 0.67883 | -0.84151 | 0.43194 | 0.44523 | 0 | 0.746925 | 1.27792 | 1.33256 | 0.24202 | 0.21244 | 0.29614 | 0.29139 | 7 | 2.93229 | 0.55262 | 0.75916 | 0.698186 | Not Significant | NS | #N/A |
| 944 | G1SPB2 | mRNA cap guanine-N7 methyltransferase OS=Orycto | RNMT | RABBIT | -1.34715 | -0.44145 | -2.46947 | 2.51045 | -0.35818 | -0.57739 | 0.40629 | 0.49878 | 0.93801 | 0.3448 | 0.743256 | 0.86172 | 0.9566 | 0.41392 | 0.35945 | 0.37693 | 0.37925 | 8 | 2.93229 | 0.55262 | 1.85987 | 1.509237 | Not Significant | NS | #N/A |

| | | | | | | | | | | | | | | | | | | | | | | | | | | | | | |
|---|---|---|---|---|---|---|---|---|---|---|---|---|---|---|---|---|---|---|---|---|---|---|---|---|---|---|---|---|---|
| 281 | G1SK00 | Ubiquitin carboxyl-terminal hydrolase OS=Oryctola | USP5 | RABBIT | -1.12385 | -0.45234 | -0.21021 | 0.15812 | -0.64258 | 0.39035 | 0.17889 | -0.24326 | 0.65726 | 0.4612 | 0.743063 | 2.81926 | 2.23915 | 0.02252 | 0.04691 | 0.11776 | 0.14585 | 8 | 2.93229 | 0.55262 | 0.17367 | 0.275311 | Significant | Up in Mtub | USP5 |
| 1620 | P41035 | Eukaryotic translation initiation factor 2 subunit 2 O | EIF2S2 EIF | RABBIT | #N/A | #N/A | -0.85466 | 0.20058 | #N/A | 2.83774 | -1.74386 | -0.06437 | -0.75959 | 1.79838 | 0.740701 | 0.5179 | 0.61891 | 0.62662 | 0.55333 | 0.45973 | 0.46295 | 5 | 2.93229 | 0.55262 | 2.92206 | 2.046159 | Not Significant | NS | #N/A |
| 444 | G1U7I9 | Spliceosome RNA helicase DDX39B OS=Oryctolagu | DDX39B | RABBIT | -1.242 | -0.71935 | -0.88475 | 0.59068 | -0.25456 | 0.08444 | 0.14164 | 0.3772 | -0.08444 | 0.674 | 0.740566 | 2.16377 | 1.94475 | 0.06242 | 0.07797 | 0.15764 | 0.18205 | 8 | 2.93229 | 0.55262 | 0.29285 | 0.362526 | Not Significant | NS | #N/A |
| 57 | G1SH05 | Tubulin beta chain OS=Oryctolagus cuniculus OX=9 | TUBB | RABBIT | -2.57937 | -1.4182 | -0.44637 | 1.30256 | 0.0757 | 1.62367 | -1.38532 | -0.15344 | 0.22339 | 0.32761 | 0.740318 | 0.90642 | 1.00057 | 0.3912 | 0.33867 | 0.36581 | 0.36727 | 8 | 2.93229 | 0.55262 | 1.66771 | 1.36862 | Not Significant | NS | #N/A |
| 1125 | G1SU30 | Large proline-rich protein BAG6 OS=Oryctolagus cu | BAG6 | RABBIT | #N/A | -0.92296 | #N/A | 0.26202 | -1.20328 | -1.3272 | -0.24033 | 0.54885 | 0.24033 | 1.36526 | 0.738788 | 1.08842 | 1.15913 | 0.31818 | 0.27645 | 0.33256 | 0.33229 | 6 | 2.93229 | 0.55262 | 0.86387 | 0.76169 | Not Significant | NS | #N/A |
| 715 | G1SKL7 | BRCA1 DNA repair associated OS=Oryctolagus cuni | BRCA1 | RABBIT | -0.84981 | -0.19256 | -0.68041 | -0.10532 | -0.61054 | 0.04768 | 0.30844 | 0.13121 | 0.19169 | 0.56924 | 0.737377 | 4.32833 | 2.59846 | 0.00252 | 0.02487 | 0.05406 | 0.11547 | 8 | 2.93229 | 0.55262 | 0.07256 | 0.20132 | Significant | Up in Mtub | BRCA1 |
| 681 | G1SUC8 | Eukaryotic translation initiation factor 3 subunit E O | EIF3E EIF3 | RABBIT | -0.84498 | -1.36504 | -0.47693 | 0.46616 | -0.23623 | 0.16879 | 0.85696 | 0.1312 | -0.16879 | 0.23923 | 0.736884 | 2.11185 | 1.91296 | 0.06768 | 0.08229 | 0.1625 | 0.18516 | 8 | 2.93229 | 0.55262 | 0.30438 | 0.370961 | Not Significant | NS | #N/A |
| 821 | A0A5F9D3D3 | Vesicle-associated membrane protein 2 OS=Oryctol | VAMP2 | RABBIT | -2.2434 | -1.54265 | 1.19236 | -1.32099 | 0.69783 | 0.12935 | -0.10269 | 0.74414 | 0.10269 | -0.40861 | 0.736347 | 1.05668 | 1.14353 | 0.32152 | 0.27724 | 0.33441 | 0.33241 | 8 | 2.93229 | 0.55262 | 1.214 | 1.036601 | Not Significant | NS | #N/A |
| 2406 | G1TUD2 | #N/A | GNG12 | RABBIT | #N/A | #N/A | #N/A | #N/A | -0.35578 | 0.3424 | #N/A | #N/A | -0.35348 | 1.15169 | 0.735984 | 0.84612 | 0.85278 | 0.48658 | 0.43323 | 0.40866 | 0.41335 | 2 | 2.93229 | 0.55262 | 0.56746 | 0.558634 | Not Significant | NS | #N/A |
| 1232 | A0A5F9CPU6 | non-specific serine/threonine protein kinase OS=Or | SLK | RABBIT | -0.95403 | -1.17898 | #N/A | 0.44072 | #N/A | -2.08863 | -0.49836 | 0.63275 | 1.90899 | 0.90413 | 0.735873 | 0.74879 | 0.85228 | 0.48229 | 0.41633 | 0.40669 | 0.40855 | 6 | 2.93229 | 0.55262 | 1.81085 | 1.397798 | Not Significant | NS | #N/A |
| 490 | A0A5F9D0F3 | Ras-related protein Rab-3 OS=Oryctolagus cunicul | RAB3C | RABBIT | -0.84712 | -0.95046 | 0.17192 | -0.08095 | -0.78182 | 0.81992 | 0.07042 | -0.06563 | 0.17528 | 0.18027 | 0.73374 | 2.68279 | 2.17288 | 0.02781 | 0.05266 | 0.12548 | 0.15178 | 8 | 2.93229 | 0.55262 | 0.187 | 0.28507 | Not Significant | NS | #N/A |
| 1522 | P9WM35 | Uncharacterized protein Rv1290c OS=Mycobacteri | #N/A | MT | -0.56656 | -0.13308 | 0.50155 | #N/A | #N/A | #N/A | #N/A | 0.66691 | #N/A | #N/A | 0.732937 | 1.18156 | 0.95092 | 0.35884 | 0.38588 | 0.35078 | 0.39334 | 2 | 2.93229 | 0.55262 | 0.28859 | 0.445556 | Not Significant | NS | #N/A |
| 1093 | B7NZD9 | Cingulin OS=Oryctolagus cuniculus OX=9986 GN=( | CGN | RABBIT | -0.79733 | #N/A | -0.41133 | #N/A | 0.76287 | #N/A | 0.58198 | #N/A | #N/A | #N/A | 0.730578 | 0.7786 | 0.81935 | 0.51771 | 0.45035 | 0.42184 | 0.42119 | 2 | 2.93229 | 0.55262 | 0.66033 | 0.596294 | Not Significant | NS | #N/A |
| 2293 | G1SG72 | #N/A | #N/A | RABBIT | #N/A | #N/A | -0.43512 | 0.14558 | #N/A | 1.50019 | #N/A | -0.55911 | -0.20322 | 1.60174 | 0.729671 | 0.84571 | 0.93822 | 0.44534 | 0.37966 | 0.38942 | 0.39025 | 4 | 2.93229 | 0.55262 | 0.99256 | 0.806466 | Not Significant | NS | #N/A |
| 284 | G1SKV7 | Cytosolic non-specific dipeptidase OS=Oryctolagus | CNDP2 | RABBIT | -0.33259 | 0.20378 | -0.77806 | -0.36304 | -0.57184 | 0.43208 | -0.00596 | 0.17321 | 0.93855 | 0.26758 | 0.729441 | 3.17573 | 2.33134 | 0.01308 | 0.03991 | 0.09752 | 0.13235 | 8 | 2.93229 | 0.55262 | 0.1319 | 0.244743 | Significant | Up in Mtub | CNDP2 |
| 1852 | G1T5B9 | #N/A | NUDT3 | RABBIT | #N/A | -0.30898 | 0.17065 | #N/A | #N/A | 0.65989 | #N/A | #N/A | #N/A | #N/A | 0.729048 | 1.75518 | 0.89604 | 0.32969 | 0.4217 | 0.33706 | 0.4086 | 1 | 2.93229 | 0.55262 | 0.11502 | 0.441334 | Not Significant | NS | #N/A |
| 569 | B7NZF1 | Protein disulfide-isomerase OS=Oryctolagus cunicu | PDIA3 | RABBIT | -0.97147 | 0.61549 | -0.9175 | 0.09292 | -1.73469 | 0.29228 | -0.78234 | 1.20551 | -0.00958 | 0.00958 | 0.726141 | 1.38081 | 1.41955 | 0.20468 | 0.18362 | 0.27183 | 0.27352 | 8 | 2.93229 | 0.55262 | 0.69137 | 0.654156 | Not Significant | NS | #N/A |
| 4 | A0A5F9CTL9 | Spectrin alpha, non-erythrocytic 1 OS=Oryctolagus | SPTAN1 | RABBIT | -0.47025 | -0.52543 | -0.39617 | -0.92271 | 0.26187 | #N/A | 1.10247 | 0.0636 | 0 | 0.09173 | 0.724987 | 2.28656 | 1.90874 | 0.05609 | 0.08558 | 0.15432 | 0.18812 | 7 | 2.93229 | 0.55262 | 0.2234 | 0.320594 | Not Significant | NS | #N/A |
| 676 | P53787 | Elongation factor 1-delta OS=Oryctolagus cuniculu | EEF1D EF1 | RABBIT | -0.71865 | -1.22817 | -0.07389 | -0.01423 | -0.0568 | -0.28642 | 0.80657 | 0.46221 | 0.07249 | 0.4782 | 0.724956 | 2.37163 | 2.02896 | 0.04513 | 0.06753 | 0.14686 | 0.17128 | 8 | 2.93229 | 0.55262 | 0.2336 | 0.319167 | Not Significant | NS | #N/A |
| 2741 | G1TH16 | #N/A | ADO | RABBIT | #N/A | #N/A | #N/A | -0.80709 | -0.778 | 0.65971 | 0.33949 | -0.06437 | 0.28879 | -1.60265 | 0.716741 | 1.0745 | 1.10142 | 0.33169 | 0.30301 | 0.33727 | 0.34914 | 5 | 2.93229 | 0.55262 | 0.63564 | 0.60495 | Not Significant | NS | #N/A |
| 2195 | A0A5F9DFJ2 | #N/A | ARHGEF2 | RABBIT | #N/A | #N/A | -0.86789 | 0.8899 | -3.00356 | 0.84892 | -3.33922 | 0.10611 | -0.17048 | 1.16735 | 0.716388 | 0.5308 | 0.62346 | 0.61462 | 0.54857 | 0.45457 | 0.46295 | 6 | 2.93229 | 0.55262 | 3.41541 | 2.475617 | Not Significant | NS | #N/A |
| 1991 | P30801 | #N/A | S100A6 | RABBIT | #N/A | -0.31136 | #N/A | #N/A | #N/A | -0.10451 | 1.2685 | -0.59552 | 0.10451 | 1.3521 | 0.716373 | 0.75593 | 0.80158 | 0.49176 | 0.44939 | 0.41065 | 0.42107 | 4 | 2.93229 | 0.55262 | 0.74839 | 0.665581 | Not Significant | NS | #N/A |
| 1076 | A0A5F9CF38 | Low molecular weight phosphotyrosine protein pho | #N/A | RABBIT | -0.61731 | -0.57725 | 0.12384 | #N/A | 0.03342 | 1.21109 | -1.34275 | #N/A | 1.18336 | 0.77233 | 0.715334 | 1.11984 | 1.18436 | 0.3056 | 0.26683 | 0.32498 | 0.32866 | 6 | 2.93229 | 0.55262 | 0.81609 | 0.729598 | Not Significant | NS | #N/A |
| 2334 | G1T0H7 | #N/A | TRAPPC3 | RABBIT | #N/A | #N/A | -0.02324 | -0.37049 | -0.69583 | -0.81452 | 0.19389 | 0.96882 | 0.3204 | 1.08716 | 0.714337 | 1.50515 | 1.43427 | 0.18299 | 0.18556 | 0.25752 | 0.27484 | 6 | 2.93229 | 0.55262 | 0.42232 | 0.465097 | Not Significant | NS | #N/A |
| 830 | A0A5F9CR38 | VPS26, retromer complex component B OS=Oryctol | VPS26B | RABBIT | -0.07658 | -0.01382 | -0.23967 | 0.0306 | -0.36622 | 1.22997 | -0.54401 | 1.04062 | 0.81571 | 0.36287 | 0.714167 | 2.19841 | 1.9329 | 0.05914 | 0.07956 | 0.15506 | 0.18311 | 8 | 2.93229 | 0.55262 | 0.26383 | 0.341289 | Not Significant | NS | #N/A |
| 2726 | A5U913 | #N/A | #N/A | MT | #N/A | #N/A | #N/A | -0.38313 | #N/A | 0.32549 | #N/A | #N/A | #N/A | #N/A | 0.708619 | #N/A | 0.67404 | #N/A | 0.5496 | #N/A | 0.46295 | 0 | 2.93229 | 0.55262 | #N/A | 0.552617 | Not Significant | NS | #N/A |
| 1138 | G1U4L4 | Acidic leucine-rich nuclear phosphoprotein 32 fami | #N/A | RABBIT | -0.77782 | 0.44382 | -0.82933 | -0.16168 | 0.27682 | #N/A | -0.83547 | 2.22584 | #N/A | 0.10404 | 0.707777 | 0.94644 | 1.02976 | 0.38046 | 0.3302 | 0.36082 | 0.36106 | 6 | 2.93229 | 0.55262 | 1.04859 | 0.885775 | Not Significant | NS | #N/A |
| 2815 | A0A5F9DV85 | #N/A | CDK5RAP2 | RABBIT | #N/A | #N/A | #N/A | #N/A | -0.35969 | 0.3463 | #N/A | #N/A | #N/A | #N/A | 0.705993 | #N/A | 0.67154 | #N/A | 0.55098 | #N/A | 0.46295 | 0 | 2.93229 | 0.55262 | #N/A | 0.552617 | Not Significant | NS | #N/A |
| 2684 | A0A5F9D214 | #N/A | NCBP1 | RABBIT | #N/A | #N/A | #N/A | -0.28256 | #N/A | #N/A | #N/A | -0.06437 | #N/A | 0.90701 | 0.703885 | 0.83673 | 0.78791 | 0.55644 | 0.47553 | 0.43509 | 0.43269 | 1 | 2.93229 | 0.55262 | 0.47179 | 0.532061 | Not Significant | NS | #N/A |
| 1725 | G1TMW2 | #N/A | TOLLIP | RABBIT | #N/A | 0.03633 | -0.13053 | 0.31999 | -0.30034 | 0.77659 | -0.11602 | -0.06437 | 1.54892 | 1.28008 | 0.703673 | 1.74773 | 1.62436 | 0.124 | 0.13557 | 0.21125 | 0.23455 | 7 | 2.93229 | 0.55262 | 0.36023 | 0.417028 | Not Significant | NS | #N/A |
| 1974 | G1TYF3 | #N/A | NT5C | RABBIT | #N/A | #N/A | #N/A | -0.69891 | #N/A | 0 | #N/A | -1.9533 | 1.15643 | 0.81515 | 0.703486 | 0.45186 | 0.56195 | 0.68204 | 0.59473 | 0.47854 | 0.47447 | 3 | 2.93229 | 0.55262 | 1.93903 | 1.253735 | Not Significant | NS | #N/A |
| 1403 | G1U685 | Secretogranin-1 OS=Oryctolagus cuniculus OX=998 | CHGB | RABBIT | #N/A | 0.19782 | -0.48145 | #N/A | -1.09157 | 0.90792 | #N/A | 0.2842 | 0.54475 | -0.76197 | 0.702125 | 1.33333 | 1.295 | 0.23994 | 0.23174 | 0.29437 | 0.30329 | 5 | 2.93229 | 0.55262 | 0.47538 | 0.50393 | Not Significant | NS | #N/A |
| 2619 | A0A5F9C8E5 | #N/A | NAA50 | RABBIT | #N/A | #N/A | #N/A | -0.32178 | #N/A | 1.07534 | -0.36357 | -0.14432 | 0.07996 | 1.24989 | 0.701242 | 0.87152 | 0.86708 | 0.43266 | 0.4149 | 0.38318 | 0.40833 | 4 | 2.93229 | 0.55262 | 0.53951 | 0.545053 | Not Significant | NS | #N/A |
| 250 | G1T4X0 | Neurochondrin OS=Oryctolagus cuniculus OX=998 | NCDN | RABBIT | -1.0164 | -0.60147 | -0.16518 | 0.52112 | -0.24362 | 1.06032 | -0.13154 | 0.19332 | 0.74669 | 0.13154 | 0.701177 | 2.089 | 1.86248 | 0.07013 | 0.08961 | 0.16349 | 0.19085 | 8 | 2.93229 | 0.55262 | 0.28166 | 0.354334 | Not Significant | NS | #N/A |
| 136 | A0A5F9C9F1 | Creatine kinase B-type OS=Oryctolagus cuniculus ( | #N/A | RABBIT | -0.48133 | 0.63418 | -0.0067 | 0.11971 | -2.2269 | -0.36474 | -0.84991 | 0.75595 | 1.12651 | 0.87168 | 0.700104 | 1.11964 | 1.19123 | 0.29535 | 0.25878 | 0.32166 | 0.32394 | 8 | 2.93229 | 0.55262 | 0.97748 | 0.863524 | Not Significant | NS | #N/A |
| 448 | G1SIV7 | Asparagine--tRNA ligase, cytoplasmic OS=Oryctola | NARS1 | RABBIT | -0.68931 | -1.09538 | -0.83426 | 0.10822 | -0.01256 | 0.48298 | -0.0306 | -0.02338 | 0.52376 | -0.00083 | 0.695042 | 2.59268 | 2.07795 | 0.03198 | 0.06207 | 0.13025 | 0.16373 | 8 | 2.93229 | 0.55262 | 0.17966 | 0.279699 | Not Significant | NS | #N/A |
| 2116 | G1SDV3 | #N/A | BDH2 | RABBIT | #N/A | #N/A | -0.83817 | #N/A | #N/A | 0.04744 | 0 | -0.89424 | 0.26947 | #N/A | 0.693833 | 1.20842 | 0.97332 | 0.31345 | 0.36839 | 0.33031 | 0.38394 | 3 | 2.93229 | 0.55262 | 0.26373 | 0.406526 | Not Significant | NS | #N/A |
| 664 | G1SPD1 | Myotrophin OS=Oryctolagus cuniculus OX=9986 GI | MTPN | RABBIT | -1.23499 | 0.03912 | -0.41723 | 0.17833 | -0.9944 | 0.1587 | -0.38561 | 0.25934 | 0.69073 | 0.31646 | 0.693756 | 2.11914 | 1.86944 | 0.06691 | 0.08856 | 0.16199 | 0.19005 | 8 | 2.93229 | 0.55262 | 0.26794 | 0.344295 | Not Significant | NS | #N/A |
| 2193 | A0A5F9CRR5 | #N/A | PRDX4 | RABBIT | #N/A | #N/A | 0.17065 | -0.18622 | #N/A | #N/A | 0.26411 | 1.90312 | #N/A | -0.11493 | 0.691885 | 0.85371 | 0.92475 | 0.45601 | 0.39116 | 0.39478 | 0.396 | 3 | 2.93229 | 0.55262 | 0.78818 | 0.671743 | Not Significant | NS | #N/A |
| 1734 | A0A5F9C1B6 | #N/A | #N/A | RABBIT | #N/A | 0.41958 | #N/A | -1.37832 | #N/A | -0.69635 | -0.07007 | 0.0057 | #N/A | 1.60781 | 0.691146 | 0.75145 | 0.84854 | 0.49418 | 0.42447 | 0.41196 | 0.40956 | 4 | 2.93229 | 0.55262 | 1.12793 | 0.884579 | Not Significant | NS | #N/A |
| 2092 | G1U6K7 | #N/A | GIMD1 | RABBIT | #N/A | -1.29501 | #N/A | -0.40618 | 0.67448 | #N/A | #N/A | #N/A | #N/A | 0.34854 | 0.690782 | 0.60654 | 0.70357 | 0.60583 | 0.51352 | 0.45335 | 0.44921 | 2 | 2.93229 | 0.55262 | 0.97279 | 0.722993 | Not Significant | NS | #N/A |
| 2630 | A0A5F9D4I0 | #N/A | #N/A | RABBIT | #N/A | #N/A | #N/A | -2.54158 | 1.30663 | #N/A | #N/A | 1.07444 | -0.85714 | 0 | 0.689905 | 0.42975 | 0.55759 | 0.69637 | 0.59752 | 0.4833 | 0.47523 | 3 | 2.93229 | 0.55262 | 3.09258 | 1.837093 | Not Significant | NS | #N/A |
| 518 | D5G340 | Apolipoprotein E (Fragment) OS=Oryctolagus cunic | #N/A | RABBIT | -1.73034 | -1.61537 | 0.12905 | -0.44877 | 0.18977 | 0.25408 | 0.0416 | 0.3037 | 0.04266 | -0.67006 | 0.689531 | 1.53216 | 1.51372 | 0.16402 | 0.15846 | 0.24348 | 0.25503 | 8 | 2.93229 | 0.55262 | 0.50633 | 0.518748 | Not Significant | NS | #N/A |
| 1184 | G1TWD4 | Large ribosomal subunit protein uL15 OS=Oryctolag | #N/A | RABBIT | -0.75737 | -2.39818 | 0.69909 | -0.69979 | 0.00253 | -0.48081 | 0.1331 | 0.25237 | 0.40083 | -0.01591 | 0.68866 | 1.28204 | 1.32425 | 0.23573 | 0.21244 | 0.29167 | 0.29139 | 8 | 2.93229 | 0.55262 | 0.72136 | 0.676096 | Not Significant | NS | #N/A |
| 2883 | G1TZ87 | #N/A | ZNF182 | RABBIT | #N/A | #N/A | #N/A | #N/A | -0.35016 | #N/A | #N/A | #N/A | #N/A | 0.33677 | 0.686927 | #N/A | 0.65341 | #N/A | 0.56109 | #N/A | 0.46564 | 0 | 2.93229 | 0.55262 | #N/A | 0.552617 | Not Significant | NS | #N/A |
| 1617 | G1T088 | Suprabasin OS=Oryctolagus cuniculus OX=9986 GI | SBSN | RABBIT | -0.97875 | -1.45013 | 0.50082 | -0.4696 | 0.43205 | 0 | -0.0896 | 0.33122 | 0.92056 | #N/A | 0.683664 | 1.42637 | 1.40932 | 0.1968 | 0.18927 | 0.26694 | 0.27623 | 7 | 2.93229 | 0.55262 | 0.51052 | 0.522945 | Not Significant | NS | #N/A |
| 716 | G1T398 | UMP-CMP kinase OS=Oryctolagus cuniculus OX=99 | CMPK1 CM | RABBIT | -1.9217 | 0.16206 | -0.81637 | 0.00523 | -0.35725 | 0.20507 | -1.3075 | -0.02861 | 0.65164 | 0.96619 | 0.682963 | 1.26152 | 1.30529 | 0.24266 | 0.2186 | 0.29642 | 0.29628 | 8 | 2.93229 | 0.55262 | 0.73273 | 0.684418 | Not Significant | NS | #N/A |
| 645 | G1TDS5 | Glutamate decarboxylase 2 OS=Oryctolagus cunicu | GAD2 | RABBIT | -0.35283 | 0.23829 | -0.2841 | -0.09811 | -0.53676 | 1.38994 | -0.35289 | 0.31097 | 0.85603 | 0.17418 | 0.682349 | 2.09031 | 1.84172 | 0.06998 | 0.09279 | 0.16349 | 0.19448 | 8 | 2.93229 | 0.55262 | 0.2664 | 0.343169 | Not Significant | NS | #N/A |
| 2798 | G1SEC2 | #N/A | MYO19 | RABBIT | #N/A | #N/A | #N/A | #N/A | -0.34779 | #N/A | #N/A | #N/A | 0.3344 | #N/A | 0.682189 | #N/A | 0.6489 | #N/A | 0.56363 | #N/A | 0.46684 | 0 | 2.93229 | 0.55262 | #N/A | 0.552617 | Not Significant | NS | #N/A |
| 585 | A0A5F9C9H6 | Insulin degrading enzyme OS=Oryctolagus cuniculu | IDE | RABBIT | 0.36273 | 0.30504 | -1.76914 | -2.70229 | 0.56231 | -0.17732 | -0.10722 | -0.71475 | 0.70337 | 0.45676 | 0.680438 | 0.95638 | 1.03801 | 0.36688 | 0.32168 | 0.35413 | 0.35646 | 8 | 2.93229 | 0.55262 | 1.26547 | 1.074265 | Not Significant | NS | #N/A |
| 1923 | A0A5F9C4J1 | #N/A | TP63 | RABBIT | #N/A | #N/A | 0.06688 | -1.20389 | #N/A | #N/A | #N/A | 0.11418 | 0.10377 | #N/A | 0.677483 | 1.06622 | 0.96562 | 0.39799 | 0.37916 | 0.36967 | 0.39016 | 2 | 2.93229 | 0.55262 | 0.40374 | 0.492249 | Not Significant | NS | #N/A |
| 2345 | G1SYK0 | #N/A | #N/A | RABBIT | #N/A | #N/A | #N/A | -0.38583 | #N/A | 1.39829 | -0.38439 | -0.10693 | 0.49566 | 0.04257 | 0.674871 | 0.88367 | 0.85914 | 0.42679 | 0.41898 | 0.38091 | 0.40859 | 4 | 2.93229 | 0.55262 | 0.48605 | 0.514204 | Not Significant | NS | #N/A |
| 654 | A0A5F9DSH4 | Actin binding LIM protein 1 OS=Oryctolagus cunicul | ABLIM1 | RABBIT | -0.36044 | -0.73139 | 0.17065 | -0.93323 | 0.02629 | 0.88514 | -0.12183 | 0.37814 | 0.07894 | #N/A | 0.670724 | 2.1801 | 1.79178 | 0.06563 | 0.10363 | 0.15994 | 0.2026 | 7 | 2.93229 | 0.55262 | 0.21034 | 0.31139 | Not Significant | NS | #N/A |
| 1922 | Q28719 | #N/A | PTGR1 LTB | RABBIT | #N/A | -1.2526 | #N/A | -0.45921 | 0.21007 | 0.8627 | -0.42022 | -0.06437 | 0.30088 | #N/A | 0.670327 | 1.39734 | 1.30407 | 0.22115 | 0.22878 | 0.28316 | 0.30149 | 5 | 2.93229 | 0.55262 | 0.3945 | 0.452952 | Not Significant | NS | #N/A |
| 1426 | G1SEX9 | 5'-nucleotidase domain containing 1 OS=Oryctolag | NT5DC1 | RABBIT | #N/A | -0.69831 | 0.04727 | -1.11982 | #N/A | 0.7399 | 0.12338 | 0.2888 | 0.14159 | -0.90387 | 0.668246 | 1.52815 | 1.40815 | 0.17734 | 0.19293 | 0.25448 | 0.27822 | 6 | 2.93229 | 0.55262 | 0.35854 | 0.422255 | Not Significant | NS | #N/A |
| 2233 | P02251 | #N/A | #N/A | RABBIT | #N/A | #N/A | 0.17065 | #N/A | -2.21705 | -2.17482 | 1.05882 | 0.07513 | 0.25332 | -0.98828 | 0.668033 | 0.59129 | 0.68626 | 0.58004 | 0.5121 | 0.44328 | 0.44921 | 5 | 2.93229 | 0.55262 | 1.82346 | 1.353676 | Not Significant | NS | #N/A |
| 2068 | G1SS48 | #N/A | #N/A | RABBIT | #N/A | -0.05612 | #N/A | #N/A | -0.01338 | #N/A | #N/A | #N/A | #N/A | 0.6327 | 0.667452 | 18.034 | 0.84871 | 0.03526 | 0.44462 | 0.13498 | 0.41885 | 1 | 2.93229 | 0.55262 | 0.00091 | 0.412316 | Not Significant | NS | #N/A |
| 2803 | G1U7G6 | #N/A | SDHA | RABBIT | #N/A | #N/A | #N/A | #N/A | -0.507 | 1.0444 | -0.71619 | 0.46197 | 0.02064 | -0.02064 | 0.665039 | 0.93314 | 0.87811 | 0.40357 | 0.40928 | 0.37287 | 0.4061 | 4 | 2.93229 | 0.55262 | 0.42327 | 0.477982 | Not Significant | NS | #N/A |
| 445 | G1U8T9 | Potassium channel tetramerization domain contain | KCTD12 | RABBIT | -0.69493 | 0.53733 | -0.61393 | -1.19917 | -0.08767 | -0.97495 | 1.19253 | 0.36598 | 0.07429 | 0.60676 | 0.664596 | 1.43209 | 1.42705 | 0.19001 | 0.1815 | 0.26255 | 0.27161 | 8 | 2.93229 | 0.55262 | 0.53841 | 0.542223 | Not Significant | NS | #N/A |
| 1735 | A0A5F9DEH6 | #N/A | #N/A | RABBIT | #N/A | #N/A | -1.01359 | 0.45292 | -0.72278 | -0.09884 | 0.09884 | -0.25213 | 0.79043 | 0.64103 | 0.663685 | 1.55609 | 1.41839 | 0.17069 | 0.19001 | 0.24933 | 0.27623 | 6 | 2.93229 | 0.55262 | 0.34108 | 0.410522 | Not Significant | NS | #N/A |
| 903 | A0A5F9C4T4 | Eukaryotic translation initiation factor 3 subunit K O | EIF3K EIF3 | RABBIT | -1.86255 | 0.47692 | -0.57806 | 0.73944 | -1.1749 | 0.66057 | 1.1151 | 0.07399 | -0.13836 | -0.79286 | 0.663519 | 1.12388 | 1.18344 | 0.29365 | 0.26173 | 0.32101 | 0.32447 | 8 | 2.93229 | 0.55262 | 0.87138 | 0.785879 | Not Significant | NS | #N/A |
| 593 | Q95MF4 | Calbindin OS=Oryctolagus cuniculus OX=9986 PE= | #N/A | RABBIT | 0.01166 | 3.94944 | -1.7949 | -2.68147 | #N/A | #N/A | #N/A | #N/A | #N/A | 0.53466 | 0.663476 | 0.2018 | 0.2753 | 0.85298 | 0.79242 | 0.52783 | 0.53886 | 3 | 2.93229 | 0.55262 | 8.64792 | 4.646467 | Not Significant | NS | #N/A |
| 1367 | G1SI47 | Forkhead box M1 OS=Oryctolagus cuniculus OX=99 | FOXM1 | RABBIT | -0.78745 | -0.94276 | 0.52599 | #N/A | -0.36872 | #N/A | -0.56762 | 0.48028 | 0.46253 | 0.70512 | 0.663314 | 1.52336 | 1.42045 | 0.1785 | 0.18943 | 0.25448 | 0.27623 | 6 | 2.93229 | 0.55262 | 0.3792 | 0.436128 | Not Significant | NS | #N/A |
| 2784 | G1T650 | #N/A | OGDHL | RABBIT | #N/A | #N/A | #N/A | #N/A | -0.36975 | #N/A | #N/A | 0.292 | #N/A | #N/A | 0.661742 | #N/A | 0.62945 | #N/A | 0.57467 | #N/A | 0.46967 | 0 | 2.93229 | 0.55262 | #N/A | 0.552617 | Not Significant | NS | #N/A |
| 776 | G1SYL8 | Elongin B OS=Oryctolagus cuniculus OX=9986 GN= | ELOB | RABBIT | -1.1513 | -0.0734 | -0.26886 | -0.19583 | 0.19804 | 0.47579 | -0.1028 | 0.34108 | 0.97514 | 0.1028 | 0.656671 | 2.24683 | 1.8816 | 0.05484 | 0.08677 | 0.15427 | 0.18876 | 8 | 2.93229 | 0.55262 | 0.21355 | 0.304494 | Not Significant | NS | #N/A |
| 1933 | A0A5F9C3L8 | #N/A | PCNT | RABBIT | #N/A | -0.46189 | #N/A | -0.12449 | #N/A | #N/A | #N/A | 0.55095 | 0.53234 | 0 | 0.654283 | 2.46964 | 1.27552 | 0.09009 | 0.2498 | 0.17996 | 0.31705 | 3 | 2.93229 | 0.55262 | 0.08423 | 0.315748 | Not Significant | NS | #N/A |
| 1063 | G1SM84 | SH3GL interacting endocytic adaptor 1 OS=Oryctol | SGIP1 | RABBIT | -2.07055 | 0.17774 | #N/A | 0.00898 | #N/A | -1.07626 | 0.02008 | 1.23013 | 0.37171 | -0.41925 | 0.653224 | 0.88575 | 0.96095 | 0.40984 | 0.36187 | 0.37548 | 0.38087 | 6 | 2.93229 | 0.55262 | 1.01977 | 0.866415 | Not Significant | NS | #N/A |
| 1173 | G1TDQ3 | Mitotic checkpoint protein BUB3 OS=Oryctolagus c | BUB3 | RABBIT | -3.06116 | -1.27887 | -1.01688 | 0.53056 | 2.04007 | -0.27587 | -0.49445 | 0.36993 | 0.27587 | 0.60297 | 0.652949 | 0.73504 | 0.81827 | 0.4833 | 0.43068 | 0.40684 | 0.41206 | 8 | 2.93229 | 0.55262 | 1.97279 | 1.591864 | Not Significant | NS | #N/A |
| 1831 | A0A5F9CWE6 | #N/A | CAMK1D | RABBIT | #N/A | #N/A | #N/A | -0.44042 | #N/A | 0.15038 | -1.30826 | -0.06437 | #N/A | 2.06802 | 0.65186 | 0.41802 | 0.52004 | 0.70405 | 0.62186 | 0.48491 | 0.48486 | 3 | 2.93229 | 0.55262 | 1.94539 | 1.256952 | Not Significant | NS | #N/A |
| 1149 | G1SG42 | Family with sequence similarity 98 member B OS=C | FAM98B | RABBIT | -3.01362 | -1.22138 | 0.09985 | 0.96309 | 0.12088 | 0.0708 | -0.44616 | -0.72422 | 0.41187 | 0.89028 | 0.650751 | 0.86397 | 0.94476 | 0.41276 | 0.36519 | 0.37673 | 0.38366 | 8 | 2.93229 | 0.55262 | 1.41831 | 1.186111 | Not Significant | NS | #N/A |
| 1724 | G1SRN2 | #N/A | #N/A | RABBIT | #N/A | -1.30212 | -1.91596 | 0.62317 | -0.01338 | 1.77423 | -2.22365 | -1.57969 | 1.35889 | 0.63462 | 0.644952 | 0.61893 | 0.70425 | 0.55556 | 0.49746 | 0.43501 | 0.44238 | 7 | 2.93229 | 0.55262 | 2.41302 | 1.863778 | Not Significant | NS | #N/A |
| 1140 | A0A5F9DA11 | Inter-alpha-trypsin inhibitor heavy chain 2 OS=Oryc | ITIH2 | RABBIT | -1.15705 | -0.20624 | #N/A | #N/A | 0.28038 | #N/A | #N/A | 0.72845 | -0.29377 | 0.40673 | 0.64144 | 1.23621 | 1.14987 | 0.28401 | 0.28832 | 0.31659 | 0.33905 | 4 | 2.93229 | 0.55262 | 0.40385 | 0.466776 | Not Significant | NS | #N/A |
| 1882 | Q865L7 | #N/A | Sult4a1 | RABBIT | #N/A | #N/A | -1.45835 | 0.52647 | -1.15182 | 0.41704 | -2.35371 | -0.48141 | 0.49975 | 1.65054 | 0.64101 | 0.64276 | 0.73297 | 0.54414 | 0.48237 | 0.43035 | 0.43528 | 6 | 2.93229 | 0.55262 | 1.86478 | 1.434022 | Not Significant | NS | #N/A |
| 1753 | U3KMR2 | #N/A | AHSG | RABBIT | #N/A | 0.61797 | #N/A | -1.95611 | #N/A | -1.47409 | 1.44641 | -0.06437 | #N/A | #N/A | 0.638392 | 0.43997 | 0.56157 | 0.68973 | 0.59497 | 0.48139 | 0.47447 | 3 | 2.93229 | 0.55262 | 2.52644 | 1.550795 | Not Significant | NS | #N/A |
| 1349 | A0A5F9C6D3 | Elongation factor like GTPase 1 OS=Oryctolagus cu | EFL1 | RABBIT | #N/A | #N/A | 0.44378 | -0.82169 | -0.44366 | 1.94099 | -0.3124 | -0.01059 | 0.25545 | -0.05377 | 0.63779 | 1.05455 | 1.08987 | 0.33223 | 0.3043 | 0.33759 | 0.3501 | 6 | 2.93229 | 0.55262 | 0.68584 | 0.642102 | Not Significant | NS | #N/A |
| 2489 | G1TYR3 | #N/A | KLC1 | RABBIT | #N/A | #N/A | #N/A | -0.37953 | #N/A | #N/A | #N/A | 0.25753 | #N/A | #N/A | 0.637062 | #N/A | 0.60597 | #N/A | 0.58822 | #N/A | 0.47237 | 0 | 2.93229 | 0.55262 | #N/A | 0.552617 | Not Significant | NS | #N/A |
| 1948 | A0A5F9DH69 | #N/A | HADHA | RABBIT | #N/A | -0.82172 | 0.2791 | #N/A | -0.12654 | -0.10845 | #N/A | #N/A | 1.11551 | 0.23186 | 0.636024 | 1.30825 | 1.17658 | 0.2609 | 0.27818 | 0.30397 | 0.3326 | 4 | 2.93229 | 0.55262 | 0.35453 | 0.43832 | Not Significant | NS | #N/A |
| 1803 | A0A5F9CMA9 | #N/A | DHDH | RABBIT | #N/A | #N/A | 0.06133 | 0.16221 | -1.80313 | #N/A | 0.10932 | #N/A | #N/A | #N/A | 0.635848 | 0.49756 | 0.60619 | 0.66811 | 0.5712 | 0.47285 | 0.46926 | 2 | 2.93229 | 0.55262 | 1.22483 | 0.825193 | Not Significant | NS | #N/A |
| 1225 | G1TDP5 | Adhesion G protein-coupled receptor V1 OS=Oryct | ADGRV1 | RABBIT | 0.01166 | #N/A | #N/A | -0.38653 | #N/A | 0.44816 | #N/A | #N/A | #N/A | #N/A | 0.635596 | 1.84312 | 0.78935 | 0.31647 | 0.47477 | 0.33209 | 0.4323 | 1 | 2.93229 | 0.55262 | 0.07928 | 0.432245 | Not Significant | NS | #N/A |
| 2370 | G1U506 | #N/A | PIP5K1A | RABBIT | #N/A | #N/A | -0.20171 | -0.49034 | #N/A | 1.34793 | #N/A | -1.11349 | 0.54638 | 0.37236 | 0.634326 | 0.81851 | 0.87808 | 0.45903 | 0.4093 | 0.39628 | 0.4061 | 4 | 2.93229 | 0.55262 | 0.80079 | 0.695814 | Not Significant | NS | #N/A |
| 239 | G1SZL3 | 4-aminobutyrate aminotransferase, mitochondrial ( | ABAT | RABBIT | -0.59866 | 0.39527 | -0.00984 | -0.37744 | -0.3073 | 0.93888 | -0.38386 | 0.61616 | 0.91696 | 0.18049 | 0.633321 | 2.08636 | 1.77911 | 0.07042 | 0.103 | 0.16349 | 0.20229 | 8 | 2.93229 | 0.55262 | 0.23036 | 0.316797 | Not Significant | NS | #N/A |
| 436 | A0A5F9CWN2 | Heterogeneous nuclear ribonucleoprotein U OS=Or | HNRNPU | RABBIT | -1.19326 | -0.26303 | 0.07751 | 0.20357 | -0.70176 | 0.18712 | 0.09314 | 0.2114 | -0.17114 | 0.96671 | 0.632839 | 1.97706 | 1.72707 | 0.08343 | 0.11226 | 0.17453 | 0.21133 | 8 | 2.93229 | 0.55262 | 0.25615 | 0.335666 | Not Significant | NS | #N/A |
| 983 | A0A5F9CVW8 | Splicing factor 3a subunit 1 OS=Oryctolagus cunicu | SF3A1 | RABBIT | 0.09627 | -2.67434 | 0.04636 | 1.21748 | -0.09799 | #N/A | 0.92045 | 0.77045 | #N/A | -0.64121 | 0.632338 | 0.67955 | 0.76774 | 0.52213 | 0.46246 | 0.42389 | 0.42567 | 6 | 2.93229 | 0.55262 | 1.62353 | 1.271969 | Not Significant | NS | #N/A |
| 1435 | G1SXK0 | SLAIN motif family member 2 OS=Oryctolagus cunic | SLAIN2 | RABBIT | -0.31029 | #N/A | #N/A | #N/A | #N/A | #N/A | #N/A | #N/A | #N/A | 0.32196 | 0.632251 | #N/A | 0.6014 | #N/A | 0.59088 | #N/A | 0.47335 | 0 | 2.93229 | 0.55262 | #N/A | 0.552617 | Not Significant | NS | #N/A |
| 1671 | G1T1V9 | #N/A | HSPA2 | RABBIT | #N/A | #N/A | -1.38123 | 0.48002 | #N/A | 0.75538 | -0.92911 | -0.17092 | 0 | 1.23643 | 0.628962 | 0.78677 | 0.85132 | 0.46705 | 0.41955 | 0.39969 | 0.40859 | 5 | 2.93229 | 0.55262 | 0.91297 | 0.779763 | Not Significant | NS | #N/A |
| 853 | Q7M2V6 | Gelsolin, cytosolic (Fragment) OS=Oryctolagus cun | #N/A | RABBIT | -0.64508 | -1.08528 | -0.35741 | #N/A | 0.12665 | -0.86831 | 0.9218 | -0.0409 | 0.67343 | 0 | 0.627486 | 1.49484 | 1.41159 | 0.17861 | 0.18862 | 0.25448 | 0.27623 | 7 | 2.93229 | 0.55262 | 0.39157 | 0.439113 | Not Significant | NS | #N/A |
| 2634 | A0A5F9DPT6 | #N/A | SMU1 | RABBIT | #N/A | #N/A | #N/A | -0.52367 | #N/A | #N/A | #N/A | -0.06437 | #N/A | 0.27014 | 0.626554 | 2.16286 | 0.78352 | 0.27571 | 0.47782 | 0.31154 | 0.43323 | 1 | 2.93229 | 0.55262 | 0.05595 | 0.426311 | Not Significant | NS | #N/A |
| 2584 | G1TAA5 | #N/A | DUSP23 | RABBIT | #N/A | #N/A | #N/A | 0.98295 | -0.30556 | 2.62927 | 0 | -0.08722 | 2.47488 | -0.20029 | 0.624635 | 0.54813 | 0.63699 | 0.60718 | 0.54207 | 0.45366 | 0.46147 | 5 | 2.93229 | 0.55262 | 1.85519 | 1.373675 | Not Significant | NS | #N/A |
| 2199 | G1TZW4 | #N/A | #N/A | RABBIT | #N/A | #N/A | -0.65162 | -0.21084 | -0.97091 | 0.5341 | 0.31297 | -1.30109 | 0.34763 | 0.1532 | 0.620489 | 1.31331 | 1.24926 | 0.23707 | 0.24331 | 0.29252 | 0.31366 | 6 | 2.93229 | 0.55262 | 0.41854 | 0.462554 | Not Significant | NS | #N/A |
| 2365 | G1SXM3 | #N/A | PES1 | RABBIT | #N/A | #N/A | -0.22425 | #N/A | #N/A | #N/A | #N/A | #N/A | 0.3949 | #N/A | 0.619142 | #N/A | 0.58893 | #N/A | 0.59819 | #N/A | 0.47527 | 0 | 2.93229 | 0.55262 | #N/A | 0.552617 | Not Significant | NS | #N/A |
| 2658 | G1SV61 | #N/A | FBXW7 | RABBIT | #N/A | #N/A | #N/A | -0.5619 | #N/A | #N/A | 0.11068 | #N/A | 0 | #N/A | 0.617242 | 6.43963 | 0.78361 | 0.09808 | 0.47778 | 0.18746 | 0.43323 | 1 | 2.93229 | 0.55262 | 0.00612 | 0.413641 | Not Significant | NS | #N/A |

| | | | | | | | | | | | | | | | | | | | | | | | | | | | | | |
|---|---|---|---|---|---|---|---|---|---|---|---|---|---|---|---|---|---|---|---|---|---|---|---|---|---|---|---|---|---|
| 1016 | G1TJM8 | Enhancer of rudimentary homolog OS=Oryctolagus | #N/A | RABBIT | -0.70596 | 0.19782 | -0.31407 | -0.10138 | -0.88254 | 0.26199 | #N/A | 0.02939 | 0.23046 | 0.4996 | 0.616585 | 2.5861 | 1.83035 | 0.03615 | 0.09732 | 0.13507 | 0.19688 | 7 | 2.93229 | 0.55262 | 0.12632 | 0.252177 | Not Significant | NS | #N/A |
| 2292 | A0A5F9DRD4 | #N/A | EEF1E1 | RABBIT | #N/A | #N/A | -0.01252 | -0.53141 | #N/A | 0.38169 | -0.4006 | 0.11881 | #N/A | 1.26838 | 0.614036 | 1.1226 | 1.04097 | 0.32443 | 0.33283 | 0.33479 | 0.36278 | 4 | 2.93229 | 0.55262 | 0.39891 | 0.463928 | Not Significant | NS | #N/A |
| 1158 | G1SPN9 | ADP-ribosylation factor-like protein 3 OS=Oryctolag | ARL3 | RABBIT | -0.83045 | -0.89366 | 0.04892 | 0.37448 | -0.60808 | 0.46686 | -0.36991 | 0.05736 | 0.54185 | 0.44471 | 0.609933 | 1.99946 | 1.70892 | 0.08058 | 0.11566 | 0.1731 | 0.21416 | 8 | 2.93229 | 0.55262 | 0.23264 | 0.318464 | Not Significant | NS | #N/A |
| 159 | A0A5F9DAK3 | Hepatocyte growth factor receptor OS=Oryctolagus | MET | RABBIT | -0.97477 | 0.19782 | 0.03841 | #N/A | 0.8025 | #N/A | #N/A | #N/A | 0.62506 | #N/A | 0.609072 | 0.73821 | 0.73553 | 0.51389 | 0.49006 | 0.42096 | 0.43948 | 3 | 2.93229 | 0.55262 | 0.54459 | 0.548558 | Not Significant | NS | #N/A |
| 1364 | A0A5F9DJR7 | Annexin OS=Oryctolagus cuniculus OX=9986 GN=A | ANXA7 | RABBIT | -0.69729 | #N/A | -0.26419 | #N/A | -0.01338 | #N/A | 1.03553 | -0.00517 | 0.39735 | -0.29272 | 0.608702 | 1.60473 | 1.32874 | 0.16946 | 0.22088 | 0.24852 | 0.2975 | 5 | 2.93229 | 0.55262 | 0.24665 | 0.359757 | Not Significant | NS | #N/A |
| 1716 | G1SLX0 | #N/A | GMFB | RABBIT | #N/A | 0.1804 | -0.60789 | -0.29159 | -0.01338 | 0.02664 | -0.01259 | 0.39339 | 0.60405 | 1.10689 | 0.606792 | 2.18347 | 1.69706 | 0.06531 | 0.12075 | 0.15941 | 0.21983 | 7 | 2.93229 | 0.55262 | 0.17162 | 0.284102 | Not Significant | NS | #N/A |
| 1081 | G1SV41 | protein-ribulosamine 3-kinase OS=Oryctolagus cun | FN3K | RABBIT | -0.97104 | -1.59469 | 0.79892 | -0.00657 | #N/A | #N/A | -1.29326 | 1.83658 | 0.15655 | -0.05107 | 0.605546 | 0.72787 | 0.81238 | 0.4941 | 0.43769 | 0.41196 | 0.41582 | 6 | 2.93229 | 0.55262 | 1.38425 | 1.111239 | Not Significant | NS | #N/A |
| 1034 | G1TJG3 | K Homology domain-containing protein OS=Oryctol | #N/A | RABBIT | -1.33097 | 0.68164 | -2.58477 | 1.17649 | -0.49721 | -0.85832 | -0.74798 | 0.66351 | 0.49305 | 0.91698 | 0.604408 | 0.77964 | 0.85555 | 0.45804 | 0.41061 | 0.39583 | 0.40623 | 8 | 2.93229 | 0.55262 | 1.50248 | 1.247703 | Not Significant | NS | #N/A |
| 1514 | G1U6J7 | Ribosomal protein L32 OS=Oryctolagus cuniculus ( | #N/A | RABBIT | #N/A | -2.25073 | #N/A | #N/A | 1.05964 | #N/A | 0 | #N/A | 0.05857 | -0.03656 | 0.602885 | 0.48848 | 0.6035 | 0.65869 | 0.56852 | 0.47097 | 0.46793 | 3 | 2.93229 | 0.55262 | 1.82795 | 1.197563 | Not Significant | NS | #N/A |
| 2717 | A0A5F9DH27 | #N/A | VPS26C | RABBIT | #N/A | #N/A | #N/A | -0.27148 | #N/A | 0.41797 | -0.00724 | -0.1985 | 0.00724 | 1.43113 | 0.601604 | 0.83812 | 0.79145 | 0.44912 | 0.4549 | 0.39066 | 0.42297 | 4 | 2.93229 | 0.55262 | 0.42936 | 0.481497 | Not Significant | NS | #N/A |
| 638 | A0A5F9CCH4 | Beta-arrestin-1 OS=Oryctolagus cuniculus OX=998 | ARRB1 | RABBIT | -0.77194 | -0.31948 | -1.28002 | 0.64596 | #N/A | -0.21727 | 0.29673 | #N/A | 0.38307 | 0.21727 | 0.601323 | 1.39709 | 1.29641 | 0.21187 | 0.22732 | 0.27513 | 0.30097 | 6 | 2.93229 | 0.55262 | 0.37051 | 0.430289 | Not Significant | NS | #N/A |
| 1717 | G1TA11 | #N/A | RARS1 | RABBIT | #N/A | -0.50471 | #N/A | 0.20434 | -0.67736 | 1.53156 | -0.7938 | -0.25356 | 0.1892 | 0.6915 | 0.598886 | 1.05604 | 1.07083 | 0.33161 | 0.31233 | 0.33727 | 0.35276 | 6 | 2.93229 | 0.55262 | 0.60302 | 0.586473 | Not Significant | NS | #N/A |
| 2077 | A5U7Y9 | #N/A | #N/A | MT | #N/A | 0.05401 | #N/A | #N/A | 0.10908 | 1.48249 | -0.12247 | #N/A | #N/A | #N/A | 0.598466 | 0.74533 | 0.77916 | 0.53376 | 0.4716 | 0.42788 | 0.43055 | 2 | 2.93229 | 0.55262 | 0.64473 | 0.589969 | Not Significant | NS | #N/A |
| 2601 | A0A5F9C7R1 | #N/A | #N/A | RABBIT | #N/A | #N/A | #N/A | #N/A | -0.30535 | #N/A | 0.29196 | #N/A | #N/A | #N/A | 0.597307 | #N/A | 0.56816 | #N/A | 0.61052 | #N/A | 0.47994 | 0 | 2.93229 | 0.55262 | #N/A | 0.552617 | Not Significant | NS | #N/A |
| 1721 | G1T6W7 | #N/A | CAT | RABBIT | #N/A | 1.92828 | #N/A | -0.05764 | -1.07326 | #N/A | 3.66416 | #N/A | #N/A | -1.94384 | 0.59437 | 0.24977 | 0.33805 | 0.81889 | 0.74697 | 0.51745 | 0.52878 | 3 | 2.93229 | 0.55262 | 6.79546 | 3.70967 | Not Significant | NS | #N/A |
| 2841 | G1TQ32 | #N/A | SCAF8 | RABBIT | #N/A | #N/A | #N/A | #N/A | -0.01338 | #N/A | 2.44776 | -1.28688 | #N/A | #N/A | 0.593825 | 0.1836 | 0.32797 | 0.8844 | 0.75965 | 0.53681 | 0.53222 | 1 | 2.93229 | 0.55262 | 6.97375 | 2.185542 | Not Significant | NS | #N/A |
| 1051 | G1TAM3 | Tubulin folding cofactor B OS=Oryctolagus cuniculu | TBCB | RABBIT | -0.82652 | 1.59793 | 0.43602 | -0.02081 | -1.45344 | 2.19355 | -0.308 | -0.27256 | 1.11303 | -0.03683 | 0.591202 | 0.82382 | 0.89519 | 0.43391 | 0.38997 | 0.38352 | 0.3954 | 8 | 2.93229 | 0.55262 | 1.28749 | 1.090382 | Not Significant | NS | #N/A |
| 2175 | A0A5F9DQD8 | #N/A | #N/A | RABBIT | #N/A | #N/A | -1.9251 | 0.16107 | #N/A | 0.61064 | -2.49433 | -1.51726 | 1.93581 | 0 | 0.588983 | 0.41495 | 0.4955 | 0.69538 | 0.63368 | 0.4833 | 0.48899 | 5 | 2.93229 | 0.55262 | 2.87815 | 2.01848 | Not Significant | NS | #N/A |
| 1548 | A0A5F9D7R7 | Activating transcription factor 3 OS=Oryctolagus cu | ATF3 | RABBIT | -0.6618 | #N/A | #N/A | -0.05764 | #N/A | #N/A | #N/A | #N/A | 0.22685 | #N/A | 0.586572 | 1.12108 | 0.70731 | 0.4637 | 0.51904 | 0.3979 | 0.45173 | 1 | 2.93229 | 0.55262 | 0.18251 | 0.458496 | Not Significant | NS | #N/A |
| 2126 | A0A5F9DF54 | #N/A | PHGDH | RABBIT | #N/A | #N/A | 0.01743 | #N/A | -0.20321 | 0.83205 | 0.15322 | #N/A | #N/A | #N/A | 0.585527 | 1.64061 | 0.94962 | 0.24257 | 0.38648 | 0.29642 | 0.39349 | 2 | 2.93229 | 0.55262 | 0.12738 | 0.380185 | Not Significant | NS | #N/A |
| 1660 | A0A5F9DAD7 | #N/A | DST | RABBIT | #N/A | -0.05354 | #N/A | -0.18546 | #N/A | #N/A | #N/A | 0.80369 | #N/A | 0.12783 | 0.585262 | 1.6998 | 0.95369 | 0.23127 | 0.38461 | 0.29002 | 0.39322 | 2 | 2.93229 | 0.55262 | 0.11855 | 0.376607 | Not Significant | NS | #N/A |
| 1003 | G1SX80 | Laminin subunit alpha 2 OS=Oryctolagus cuniculus | LAMA2 | RABBIT | -0.5936 | -2.54016 | -3.49304 | -0.05764 | 2.26714 | #N/A | 0.82727 | 0.44069 | -3.43946 | 0.97757 | 0.584974 | 0.39829 | 0.46338 | 0.70228 | 0.65308 | 0.48414 | 0.49788 | 7 | 2.93229 | 0.55262 | 4.79351 | 3.541477 | Not Significant | NS | #N/A |
| 547 | A0A5F9DGI1 | Reticulon OS=Oryctolagus cuniculus OX=9986 GN= | RTN3 | RABBIT | -0.85324 | -0.69139 | 0.53989 | -0.2792 | 0.08324 | 0.33119 | 1.2253 | 0.54264 | -0.27879 | -0.09662 | 0.584887 | 1.59411 | 1.47237 | 0.14958 | 0.16912 | 0.23045 | 0.26263 | 8 | 2.93229 | 0.55262 | 0.33655 | 0.394503 | Not Significant | NS | #N/A |
| 1266 | G1SIW1 | RNA helicase OS=Oryctolagus cuniculus OX=9986 ( | DHX15 | RABBIT | 0.0354 | -2.40789 | 0.04678 | 0.38116 | -0.03712 | -0.9008 | 0.43312 | 0.58341 | -0.11179 | 0.93777 | 0.584677 | 0.97334 | 1.02823 | 0.35889 | 0.32606 | 0.35078 | 0.35925 | 8 | 2.93229 | 0.55262 | 0.90207 | 0.808339 | Not Significant | NS | #N/A |
| 2288 | G1SL52 | #N/A | COG4 | RABBIT | #N/A | #N/A | -0.20631 | #N/A | #N/A | #N/A | 0.37696 | #N/A | #N/A | #N/A | 0.583268 | #N/A | 0.55481 | #N/A | 0.61853 | #N/A | 0.4838 | 0 | 2.93229 | 0.55262 | #N/A | 0.552617 | Not Significant | NS | #N/A |
| 1010 | A0A5F9D5B7 | Endophilin-B1 OS=Oryctolagus cuniculus OX=9986 | SH3GLB1 | RABBIT | -1.29711 | 0.20379 | 0.16468 | 0.13023 | -1.14346 | 0.39449 | -0.13633 | 0.57733 | -0.12521 | 0.26181 | 0.58279 | 1.5791 | 1.46172 | 0.15297 | 0.17196 | 0.23372 | 0.26466 | 8 | 2.93229 | 0.55262 | 0.34052 | 0.39741 | Not Significant | NS | #N/A |
| 410 | G1U4K2 | Synaptotagmin OS=Oryctolagus cuniculus OX=998 | SYT1 | RABBIT | -0.43088 | -1.5894 | 0.52092 | -1.09371 | 0.76264 | 0.40762 | 0.00313 | 0.75434 | -0.00313 | -0.07935 | 0.582609 | 1.21509 | 1.22141 | 0.25897 | 0.24762 | 0.30317 | 0.31626 | 8 | 2.93229 | 0.55262 | 0.57475 | 0.568814 | Not Significant | NS | #N/A |
| 2162 | A0A5F9D6Z2 | #N/A | #N/A | RABBIT | #N/A | #N/A | -0.47651 | -0.05764 | #N/A | 1.54817 | 0.48224 | -0.17931 | -0.4346 | 0.15773 | 0.581922 | 0.99011 | 0.96888 | 0.36758 | 0.36122 | 0.35413 | 0.38042 | 5 | 2.93229 | 0.55262 | 0.49347 | 0.515337 | Not Significant | NS | #N/A |
| 44 | G1SWS9 | Vimentin OS=Oryctolagus cuniculus OX=9986 GN= | VIM | RABBIT | -0.45873 | -0.5982 | -0.13178 | -0.68 | 0.28905 | -3.06188 | 2.00366 | 0.69413 | 0.81887 | 0.85502 | 0.577892 | 0.65512 | 0.72882 | 0.53077 | 0.48143 | 0.42666 | 0.43475 | 8 | 2.93229 | 0.55262 | 1.94533 | 1.571772 | Not Significant | NS | #N/A |
| 581 | G1SF32 | Translocase of outer mitochondrial membrane 70 C | TOMM70 | RABBIT | -0.98717 | 0.34219 | -0.40216 | -0.20201 | 0.39711 | 1.55183 | 0.29933 | -0.77695 | 1.16642 | -0.21629 | 0.575276 | 1.15103 | 1.16922 | 0.28296 | 0.26718 | 0.31631 | 0.32868 | 8 | 2.93229 | 0.55262 | 0.62448 | 0.605203 | Not Significant | NS | #N/A |
| 870 | G1TWQ3 | NAD-dependent protein deacetylase sirtuin-2 OS=C | SIRT2 | RABBIT | -0.10601 | 0.17082 | 0.19765 | 0.20184 | -0.26006 | -0.8044 | -1.63398 | 1.5498 | 1.85138 | 2.11497 | 0.574706 | 0.74491 | 0.81696 | 0.47764 | 0.43139 | 0.40468 | 0.41229 | 8 | 2.93229 | 0.55262 | 1.48807 | 1.237157 | Not Significant | NS | #N/A |
| 456 | A0A5F9C4G4 | Exportin 7 OS=Oryctolagus cuniculus OX=9986 GN | XPO7 | RABBIT | -1.02717 | -3.1246 | #N/A | 0.83973 | 1.01407 | #N/A | #N/A | #N/A | 0 | #N/A | 0.574492 | 0.26556 | 0.3491 | 0.80778 | 0.73907 | 0.5152 | 0.52579 | 3 | 2.93229 | 0.55262 | 3.74383 | 2.166437 | Not Significant | NS | #N/A |
| 2014 | G1SSQ9 | #N/A | DNAL1 | RABBIT | #N/A | #N/A | 0.11535 | -0.37399 | #N/A | 1.67798 | -0.20637 | -0.00907 | 0.31774 | #N/A | 0.574388 | 0.87722 | 0.88346 | 0.4299 | 0.40658 | 0.38166 | 0.40482 | 4 | 2.93229 | 0.55262 | 0.57166 | 0.563604 | Not Significant | NS | #N/A |
| 1862 | G1T923 | #N/A | ATP6AP2 | RABBIT | #N/A | -2.32218 | 0.37525 | #N/A | 0.38538 | -0.06976 | 0 | 0.51423 | -0.22916 | #N/A | 0.574345 | 0.73886 | 0.81221 | 0.49319 | 0.44036 | 0.41161 | 0.41691 | 5 | 2.93229 | 0.55262 | 1.03586 | 0.857219 | Not Significant | NS | #N/A |
| 2877 | P9WIZ9 | #N/A | #N/A | MT | #N/A | #N/A | #N/A | #N/A | -0.29235 | #N/A | 0.27897 | #N/A | #N/A | #N/A | 0.571324 | #N/A | 0.54345 | #N/A | 0.62541 | #N/A | 0.4863 | 0 | 2.93229 | 0.55262 | #N/A | 0.552617 | Not Significant | NS | #N/A |
| 2262 | Q09YN8 | #N/A | TES | RABBIT | #N/A | #N/A | 1.59994 | -2.18645 | -0.82873 | -0.04602 | -0.08458 | -0.01549 | 0.58212 | 0.04602 | 0.568158 | 0.68838 | 0.7631 | 0.51693 | 0.46508 | 0.42179 | 0.42733 | 6 | 2.93229 | 0.55262 | 1.27726 | 1.039378 | Not Significant | NS | #N/A |
| 1768 | G1SRL3 | #N/A | NMT1 | RABBIT | #N/A | #N/A | -0.98995 | 0.29577 | -1.00163 | 0.21129 | -0.54434 | -0.2054 | 0.14103 | 0.40898 | 0.56758 | 1.4677 | 1.2781 | 0.19256 | 0.23343 | 0.26339 | 0.30435 | 6 | 2.93229 | 0.55262 | 0.2804 | 0.369764 | Not Significant | NS | #N/A |
| 1399 | A0A5F9CP03 | Protein kinase C-binding protein NELL2 OS=Oryctol | NELL2 | RABBIT | 0.01166 | -0.29755 | #N/A | #N/A | #N/A | #N/A | 0.42335 | #N/A | #N/A | #N/A | 0.5663 | 2.11472 | 0.7099 | 0.2812 | 0.5176 | 0.31532 | 0.45117 | 1 | 2.93229 | 0.55262 | 0.04781 | 0.424241 | Not Significant | NS | #N/A |
| 832 | G1SJX0 | Wiskott-Aldrich syndrome protein family member O | WASF3 | RABBIT | -1.18383 | -2.42233 | 0.65207 | -0.05764 | 0.79678 | -0.86718 | -0.92839 | #N/A | 1.72699 | 0.55645 | 0.564955 | 0.63799 | 0.71401 | 0.54378 | 0.49166 | 0.43035 | 0.43988 | 7 | 2.93229 | 0.55262 | 1.74255 | 1.391249 | Not Significant | NS | #N/A |
| 2638 | A0A375Z1L1 | #N/A | #N/A | MT | #N/A | #N/A | #N/A | -0.37638 | #N/A | 0 | #N/A | #N/A | #N/A | 0.37651 | 0.564637 | 1.73164 | 0.70297 | 0.3334 | 0.52146 | 0.33807 | 0.45236 | 1 | 2.93229 | 0.55262 | 0.07088 | 0.430109 | Not Significant | NS | #N/A |
| 224 | G1SLQ4 | Actin-related protein 3 OS=Oryctolagus cuniculus ( | #N/A | RABBIT | -0.26346 | -0.22977 | #N/A | #N/A | #N/A | -0.04386 | 0.98285 | 0.24325 | 0.40586 | 0 | 0.564236 | 1.81808 | 1.25013 | 0.12873 | 0.24687 | 0.21348 | 0.3161 | 5 | 2.93229 | 0.55262 | 0.13759 | 0.291013 | Not Significant | NS | #N/A |
| 2486 | A0A5F9DBB3 | #N/A | USP7 | RABBIT | #N/A | #N/A | #N/A | -0.28504 | #N/A | -0.00116 | #N/A | #N/A | 0.00116 | 0.835 | 0.56337 | 1.01204 | 0.75036 | 0.41805 | 0.48727 | 0.37849 | 0.43895 | 2 | 2.93229 | 0.55262 | 0.23241 | 0.422776 | Not Significant | NS | #N/A |
| 2507 | A0A5F9DIC7 | #N/A | ELAVL2 | RABBIT | #N/A | #N/A | #N/A | -0.05764 | #N/A | #N/A | #N/A | 1.16713 | #N/A | -0.16249 | 0.559957 | 0.48629 | 0.5729 | 0.71185 | 0.59787 | 0.48851 | 0.47523 | 1 | 2.93229 | 0.55262 | 0.88394 | 0.636875 | Not Significant | NS | #N/A |
| 2008 | G1TGR8 | #N/A | #N/A | RABBIT | #N/A | -0.3361 | #N/A | -0.09206 | #N/A | #N/A | #N/A | #N/A | 0.03442 | 0.65527 | 0.558928 | 1.67573 | 0.91438 | 0.23578 | 0.40301 | 0.29167 | 0.40266 | 2 | 2.93229 | 0.55262 | 0.11125 | 0.373647 | Not Significant | NS | #N/A |
| 968 | G1T439 | Vesicular glutamate transporter 1 OS=Oryctolagus ( | SLC17A7 | RABBIT | -1.47275 | -1.23909 | 1.09248 | -0.93478 | 1.03806 | 1.02206 | -0.89193 | 1.13074 | 0.14912 | -0.14912 | 0.55539 | 0.81733 | 0.88126 | 0.4374 | 0.39714 | 0.3855 | 0.39983 | 8 | 2.93229 | 0.55262 | 1.15435 | 0.992949 | Not Significant | NS | #N/A |
| 2124 | Q9N0Z6 | #N/A | ATP1A1 | RABBIT | #N/A | #N/A | 0.36263 | -2.62804 | 0.8223 | -0.15281 | 0.15281 | 0.76221 | -0.20101 | -0.19061 | 0.555154 | 0.67116 | 0.7443 | 0.5271 | 0.47582 | 0.42539 | 0.43269 | 6 | 2.93229 | 0.55262 | 1.28284 | 1.043121 | Not Significant | NS | #N/A |
| 833 | G1U1V6 | Y-box-binding protein 1 OS=Oryctolagus cuniculus ( | #N/A | RABBIT | -0.82644 | -1.01994 | -0.49724 | 0.09872 | 1.05266 | #N/A | -0.15636 | 0.90116 | 0.2021 | #N/A | 0.554081 | 1.01047 | 1.01377 | 0.35127 | 0.33736 | 0.34717 | 0.36661 | 6 | 2.93229 | 0.55262 | 0.56377 | 0.560106 | Not Significant | NS | #N/A |
| 2261 | G1T2F2 | #N/A | PPIB | RABBIT | #N/A | #N/A | #N/A | -0.26852 | #N/A | -0.09619 | 0.23275 | 1.58779 | -0.3942 | 0.09619 | 0.553783 | 0.66063 | 0.66864 | 0.54495 | 0.52536 | 0.43035 | 0.45323 | 4 | 2.93229 | 0.55262 | 0.58557 | 0.57163 | Not Significant | NS | #N/A |
| 810 | G1TH09 | 60S ribosomal protein L7a OS=Oryctolagus cunicul | #N/A | RABBIT | -2.50312 | -0.67892 | 0.85143 | -0.83982 | 0.90079 | -0.20696 | 0.20696 | 0.42412 | -0.23962 | 0.30667 | 0.552164 | 0.85678 | 0.91618 | 0.41649 | 0.37934 | 0.37794 | 0.39016 | 8 | 2.93229 | 0.55262 | 1.03834 | 0.908058 | Not Significant | NS | #N/A |
| 478 | G1T239 | ATPase H+ transporting V0 subunit d1 OS=Oryctola | ATP6V0D1 | RABBIT | -1.55446 | -1.50969 | 1.07053 | -1.10559 | 1.20435 | 0.38482 | 0.46086 | 1.19601 | -0.38482 | -0.79367 | 0.551613 | 0.7718 | 0.8382 | 0.46242 | 0.41987 | 0.3975 | 0.40859 | 8 | 2.93229 | 0.55262 | 1.27702 | 1.082717 | Not Significant | NS | #N/A |
| 2682 | G1T3N8 | #N/A | AIMP2 | RABBIT | #N/A | #N/A | #N/A | 0.22189 | #N/A | 4.30147 | -0.38369 | -0.3439 | -0.79531 | 1.07341 | 0.548501 | 0.2388 | 0.30082 | 0.82299 | 0.77237 | 0.5187 | 0.53484 | 4 | 2.93229 | 0.55262 | 4.39632 | 2.770475 | Not Significant | NS | #N/A |
| 1650 | G1SPR0 | SPOC domain containing 1 OS=Oryctolagus cunicu | SPOCD1 | RABBIT | -0.53667 | -1.69435 | #N/A | #N/A | 0.88015 | -0.10333 | #N/A | #N/A | 0.29441 | 0.10333 | 0.548425 | 0.72807 | 0.78894 | 0.5069 | 0.45628 | 0.41787 | 0.42356 | 4 | 2.93229 | 0.55262 | 0.85109 | 0.724838 | Not Significant | NS | #N/A |
| 474 | G1T5W9 | Neuron-specific calcium-binding protein hippocalci | HPCA | RABBIT | -0.02388 | -2.41783 | 0.17604 | 0.36621 | -0.01877 | 0.17259 | -0.19678 | 0.14947 | 0.72332 | -0.02654 | 0.548059 | 1.02164 | 1.05497 | 0.33685 | 0.31419 | 0.34015 | 0.35371 | 8 | 2.93229 | 0.55262 | 0.71945 | 0.6747 | Not Significant | NS | #N/A |
| 2599 | G1SRK6 | #N/A | EPHA4 | RABBIT | #N/A | #N/A | #N/A | -0.44804 | #N/A | 0 | #N/A | #N/A | 0.19979 | #N/A | 0.547938 | 3.16688 | 0.69268 | 0.19472 | 0.52724 | 0.2651 | 0.45323 | 1 | 2.93229 | 0.55262 | 0.01996 | 0.417159 | Not Significant | NS | #N/A |
| 2347 | A0A5F9C456 | #N/A | UBA5 | RABBIT | #N/A | #N/A | #N/A | -0.19733 | #N/A | 1.88223 | -0.29343 | -0.21978 | 0.13969 | 0.24216 | 0.547508 | 0.56391 | 0.60299 | 0.60293 | 0.56571 | 0.4521 | 0.46744 | 4 | 2.93229 | 0.55262 | 0.78556 | 0.687026 | Not Significant | NS | #N/A |
| 1109 | G1SF06 | EH domain containing 3 OS=Oryctolagus cuniculus | EHD3 | RABBIT | 0.11139 | -0.32806 | -0.76123 | -0.53329 | 0.70159 | -0.09973 | -1.3246 | 1.12815 | 0.80908 | 1.4114 | 0.546782 | 0.97513 | 1.01645 | 0.35806 | 0.33138 | 0.35078 | 0.36166 | 8 | 2.93229 | 0.55262 | 0.78604 | 0.723433 | Not Significant | NS | #N/A |
| 1941 | G1TBR6 | #N/A | VIRMA | RABBIT | #N/A | 0.19782 | #N/A | -1.01102 | #N/A | -1.14514 | 1.89769 | -0.01031 | 0.07874 | -0.12525 | 0.545744 | 0.6181 | 0.68519 | 0.56357 | 0.51275 | 0.4379 | 0.44921 | 5 | 2.93229 | 0.55262 | 1.11368 | 0.906275 | Not Significant | NS | #N/A |
| 1249 | G1SJS2 | Translation initiation factor eIF2B subunit alpha OS= | EIF2B1 | RABBIT | -0.28942 | 0.20601 | -0.02466 | -0.06583 | -1.78772 | 0.87301 | 0.59353 | 0.30296 | -1.21161 | 0.19921 | 0.543743 | 1.07083 | 1.09193 | 0.31549 | 0.29834 | 0.33163 | 0.34592 | 8 | 2.93229 | 0.55262 | 0.64459 | 0.619924 | Not Significant | NS | #N/A |
| 117 | G1T7Z6 | Phosphoglycerate kinase OS=Oryctolagus cuniculu | #N/A | RABBIT | -0.70344 | -0.05314 | -0.16056 | 0.21107 | -0.21802 | 0.77521 | -0.08359 | 0.01922 | 0.70796 | 0.37311 | 0.543199 | 2.3693 | 1.73737 | 0.0453 | 0.11037 | 0.14686 | 0.20933 | 8 | 2.93229 | 0.55262 | 0.13141 | 0.244385 | Not Significant | NS | #N/A |
| 2511 | Q5UBU2 | #N/A | #N/A | RABBIT | #N/A | #N/A | #N/A | -0.27888 | #N/A | #N/A | 0 | #N/A | 0.52849 | #N/A | 0.543125 | 1.18668 | 0.66284 | 0.44578 | 0.54426 | 0.38942 | 0.46219 | 1 | 2.93229 | 0.55262 | 0.13965 | 0.447597 | Not Significant | NS | #N/A |
| 1391 | A0A5F9CGB9 | Coiled-coil domain containing 63 OS=Oryctolagus ( | CCDC63 | RABBIT | -0.13281 | 0.58822 | -0.62185 | #N/A | -0.01338 | #N/A | 0.49411 | #N/A | #N/A | #N/A | 0.539063 | 0.96921 | 0.76397 | 0.40393 | 0.47416 | 0.37287 | 0.432 | 3 | 2.93229 | 0.55262 | 0.24748 | 0.398306 | Not Significant | NS | #N/A |
| 2215 | A0A5F9CMJ5 | #N/A | NPTN | RABBIT | #N/A | #N/A | 0.27871 | -1.43533 | 0.41999 | -0.33733 | -0.2035 | 0.8609 | -0.10806 | 1.24673 | 0.537292 | 0.88249 | 0.91376 | 0.41147 | 0.38485 | 0.37603 | 0.39323 | 6 | 2.93229 | 0.55262 | 0.69503 | 0.648276 | Not Significant | NS | #N/A |
| 1911 | G1SQR6 | #N/A | #N/A | RABBIT | #N/A | -0.45895 | #N/A | #N/A | #N/A | 0.02137 | 0.33327 | -0.08573 | -0.14914 | 0.271 | 0.537104 | 2.28594 | 0.96102 | 0.08423 | 0.36885 | 0.17453 | 0.38394 | 4 | 2.93229 | 0.55262 | 0.046 | 0.260297 | Not Significant | NS | #N/A |
| 2353 | A0A5F9CT64 | #N/A | UQCRC1 | RABBIT | #N/A | #N/A | 0.21786 | -1.16928 | #N/A | 0.03432 | #N/A | 0.8555 | -0.61273 | -0.03432 | 0.536402 | 0.86372 | 0.8504 | 0.43646 | 0.4235 | 0.38515 | 0.40953 | 4 | 2.93229 | 0.55262 | 0.51425 | 0.530481 | Not Significant | NS | #N/A |
| 2483 | G1SES9 | #N/A | ATIC | RABBIT | #N/A | #N/A | #N/A | -0.05764 | -0.64976 | 0.88393 | -0.22277 | -0.43934 | 0.584 | 0.10309 | 0.535481 | 1.21574 | 1.03965 | 0.27835 | 0.32915 | 0.31332 | 0.3606 | 5 | 2.93229 | 0.55262 | 0.27715 | 0.378978 | Not Significant | NS | #N/A |
| 1214 | G1TKB3 | Large ribosomal subunit protein eL13 OS=Oryctolag | RPL13 | RABBIT | -2.0469 | 0.04267 | 0.44328 | -0.66261 | 0.29088 | -0.1354 | -0.28539 | 0.07104 | 0.70146 | 0.38772 | 0.534418 | 1.09058 | 1.10239 | 0.30721 | 0.29397 | 0.32551 | 0.34341 | 8 | 2.93229 | 0.55262 | 0.60032 | 0.587528 | Not Significant | NS | #N/A |
| 2115 | A0A5F9D8A6 | #N/A | SNX12 | RABBIT | #N/A | -1.09367 | -0.53905 | -0.55319 | 0.83487 | 0 | 0.92656 | -0.12703 | 0.10155 | 0.06109 | 0.530193 | 1.26399 | 1.19327 | 0.24669 | 0.26049 | 0.29787 | 0.32441 | 7 | 2.93229 | 0.55262 | 0.39099 | 0.438707 | Not Significant | NS | #N/A |
| 755 | G1TLE4 | G protein subunit beta 2 OS=Oryctolagus cuniculus | GNB2 | RABBIT | 1.04974 | #N/A | -1.42348 | -0.97692 | -0.468 | 0.45462 | 0.47052 | 0.47155 | -1.09879 | #N/A | 0.529142 | 0.79543 | 0.84953 | 0.45667 | 0.41777 | 0.39511 | 0.40859 | 6 | 2.93229 | 0.55262 | 0.88505 | 0.775922 | Not Significant | NS | #N/A |
| 208 | Q71V39 | Elongation factor 1-alpha 2 OS=Oryctolagus cunicu | EEF1A2 | RABBIT | -1.68674 | -0.90737 | -0.11019 | 0.31472 | -0.43715 | 0.96129 | -2.32522 | 0.21648 | 0.37388 | 0.58452 | 0.527535 | 0.77677 | 0.83746 | 0.45964 | 0.42027 | 0.39628 | 0.40859 | 8 | 2.93229 | 0.55262 | 1.15307 | 0.992012 | Not Significant | NS | #N/A |
| 590 | G1T196 | Striatin 3 OS=Oryctolagus cuniculus OX=9986 GN= | STRN3 | RABBIT | 0.27188 | 0.13779 | -0.18169 | -0.50506 | -0.14016 | #N/A | 0.7577 | 0.84051 | 0.17377 | 0 | 0.526443 | 2.19891 | 1.56047 | 0.06383 | 0.14992 | 0.15847 | 0.24914 | 7 | 2.93229 | 0.55262 | 0.12737 | 0.252917 | Not Significant | NS | #N/A |
| 843 | G1SP01 | Prolyl endopeptidase OS=Oryctolagus cuniculus O) | PREPL | RABBIT | -1.70034 | 0.13175 | -2.18971 | 0.40929 | 0.33358 | 0.46892 | -0.01217 | -0.05219 | 1.05511 | -1.85286 | 0.52445 | 0.71035 | 0.7748 | 0.49767 | 0.45489 | 0.41323 | 0.42297 | 8 | 2.93229 | 0.55262 | 1.36272 | 1.145431 | Not Significant | NS | #N/A |
| 990 | G1SUK5 | Density-regulated protein OS=Oryctolagus cuniculu | DENR | RABBIT | -0.28052 | -0.97348 | 0.87656 | -0.11267 | 0.06169 | 1.12594 | -0.2333 | -0.09924 | 0.03487 | 1.35176 | 0.521691 | 1.16881 | 1.15201 | 0.27613 | 0.27389 | 0.31174 | 0.33128 | 8 | 2.93229 | 0.55262 | 0.49806 | 0.512693 | Not Significant | NS | #N/A |
| 291 | A0A5F9CLN2 | 4-trimethylaminobutyraldehyde dehydrogenase OS | #N/A | RABBIT | 0.10256 | -0.29293 | 0.01381 | -0.14854 | -0.32918 | 1.12097 | -0.46496 | 0.13867 | 0.73771 | 0.41019 | 0.519371 | 1.84141 | 1.51513 | 0.10282 | 0.1581 | 0.19228 | 0.25477 | 8 | 2.93229 | 0.55262 | 0.19888 | 0.293762 | Not Significant | NS | #N/A |
| 172 | A0A5F9DTG8 | Calcium dependent secretion activator OS=Oryctol | #N/A | RABBIT | -0.52303 | -0.34324 | #N/A | -0.05764 | -0.15355 | 0.19706 | -0.19507 | 0.48101 | 0.39751 | 0.34553 | 0.514572 | 3.15776 | 1.69529 | 0.01598 | 0.12109 | 0.10524 | 0.21983 | 7 | 2.93229 | 0.55262 | 0.05901 | 0.204736 | Not Significant | NS | #N/A |
| 2887 | G1SLH1 | #N/A | NKX3-1 | RABBIT | #N/A | #N/A | #N/A | #N/A | -0.29425 | #N/A | #N/A | 0.2165 | #N/A | #N/A | 0.510758 | #N/A | 0.48583 | #N/A | 0.66107 | #N/A | 0.4996 | 0 | 2.93229 | 0.55262 | #N/A | 0.552617 | Not Significant | NS | #N/A |
| 1848 | A0A5F9DFE5 | #N/A | #N/A | RABBIT | #N/A | -1.64534 | -0.72162 | 0.13306 | -0.01338 | 0.31481 | -0.53808 | 0.27929 | -0.35812 | 0.03926 | 0.509254 | 1.25391 | 1.16934 | 0.25013 | 0.26956 | 0.2994 | 0.32966 | 7 | 2.93229 | 0.55262 | 0.36654 | 0.421478 | Not Significant | NS | #N/A |
| 2156 | A0A5F9CHW6 | #N/A | EIF2S1 | RABBIT | #N/A | #N/A | -0.81719 | #N/A | -0.16098 | 0.433 | -0.80219 | 0.29973 | 0.15831 | 0 | 0.506855 | 1.25795 | 1.02331 | 0.26396 | 0.33635 | 0.30583 | 0.36614 | 5 | 2.93229 | 0.55262 | 0.23192 | 0.350472 | Not Significant | NS | #N/A |
| 2351 | A0A5F9CT24 | #N/A | HCFC1 | RABBIT | #N/A | #N/A | #N/A | -0.5775 | #N/A | #N/A | #N/A | 0.4555 | -1.36906 | 0.69722 | 0.505386 | 0.38744 | 0.47585 | 0.73578 | 0.65451 | 0.49419 | 0.49809 | 2 | 2.93229 | 0.55262 | 1.27616 | 0.846008 | Not Significant | NS | #N/A |
| 2341 | G1TBJ4 | #N/A | NDRG1 | RABBIT | #N/A | #N/A | 0.17065 | -0.30953 | #N/A | 0.2698 | #N/A | -0.15872 | #N/A | 1.1949 | 0.504768 | 0.92478 | 0.82069 | 0.42329 | 0.44354 | 0.37994 | 0.41812 | 3 | 2.93229 | 0.55262 | 0.35751 | 0.453948 | Not Significant | NS | #N/A |
| 1066 | G1SIK0 | Antithrombin-III OS=Oryctolagus cuniculus OX=998 | SERPINC1 | RABBIT | 0.31799 | -0.15763 | #N/A | -0.1273 | 0.22399 | 0 | 3.40205 | 1.22438 | -1.7454 | -0.03753 | 0.504437 | 0.51927 | 0.58699 | 0.61958 | 0.57031 | 0.45663 | 0.46875 | 7 | 2.93229 | 0.55262 | 2.09708 | 1.641114 | Not Significant | NS | #N/A |
| 1881 | A0A5F9C1X6 | #N/A | SLC10A3 | RABBIT | #N/A | -0.46294 | #N/A | -0.03529 | #N/A | -0.99549 | 2.05864 | 0.76781 | -0.55572 | 0 | 0.504165 | 0.55503 | 0.61914 | 0.60279 | 0.55319 | 0.4521 | 0.46295 | 5 | 2.93229 | 0.55262 | 1.17871 | 0.947268 | Not Significant | NS | #N/A |
| 1537 | G1T7K7 | Chromosome 10 open reading frame 71 OS=Orycto | C10orf71 | RABBIT | -1.40217 | -0.94596 | 0.17065 | -0.70417 | 0.08494 | #N/A | 0.03831 | 0.29947 | -0.65036 | 0.08736 | 0.503036 | 1.29757 | 1.18785 | 0.23556 | 0.26252 | 0.29167 | 0.32498 | 7 | 2.93229 | 0.55262 | 0.33398 | 0.398528 | Not Significant | NS | #N/A |
| 2323 | G1SNN6 | #N/A | HAT1 | RABBIT | #N/A | #N/A | -0.14131 | #N/A | #N/A | #N/A | 0.72141 | #N/A | 0 | #N/A | 0.502011 | 0.80353 | 0.5927 | 0.56908 | 0.58578 | 0.43934 | 0.47237 | 1 | 2.93229 | 0.55262 | 0.26021 | 0.478257 | Not Significant | NS | #N/A |
| 537 | G1SLM1 | Calcyclin-binding protein OS=Oryctolagus cuniculu | RABGAP1L | RABBIT | 0.09266 | 0.11683 | -0.22619 | 1.12066 | -0.40367 | -0.8535 | -0.23792 | 1.13508 | 0.95382 | 2.21171 | 0.501782 | 0.83576 | 0.88281 | 0.42754 | 0.39634 | 0.38096 | 0.39935 | 8 | 2.93229 | 0.55262 | 0.90116 | 0.807674 | Not Significant | NS | #N/A |
| 670 | G1SRF7 | Stress-70 protein, mitochondrial OS=Oryctolagus c | HSPA9 | RABBIT | -0.74939 | 0.71673 | 0.5191 | -0.06159 | -0.12026 | -0.08787 | 0.00395 | 1.75527 | 1.27692 | -0.14602 | 0.49953 | 1.05141 | 1.05435 | 0.32379 | 0.31447 | 0.33479 | 0.35371 | 8 | 2.93229 | 0.55262 | 0.56431 | 0.561174 | Not Significant | NS | #N/A |
| 2752 | G1TVC8 | #N/A | CDC27 | RABBIT | #N/A | #N/A | #N/A | -0.33285 | #N/A | #N/A | -0.46434 | 0.21084 | 0.75319 | #N/A | 0.499414 | 0.70906 | 0.62465 | 0.5518 | 0.55995 | 0.43309 | 0.46562 | 2 | 2.93229 | 0.55262 | 0.37207 | 0.479405 | Not Significant | NS | #N/A |
| 2178 | G1U3Q3 | #N/A | ACSBG1 | RABBIT | #N/A | #N/A | -1.28238 | -0.05764 | #N/A | 0.84376 | -2.81148 | 0.74117 | -0.34294 | 0.71101 | 0.498313 | 0.41314 | 0.484 | 0.69662 | 0.64146 | 0.4833 | 0.49339 | 5 | 2.93229 | 0.55262 | 2.07828 | 1.514298 | Not Significant | NS | #N/A |
| 672 | G1TCW5 | Xaa-Pro dipeptidase OS=Oryctolagus cuniculus OX= | PEPD | RABBIT | 0.03232 | 0.28484 | 0.00073 | -0.05764 | -0.46976 | #N/A | -0.29862 | #N/A | 1.21028 | #N/A | 0.497732 | 1.11017 | 0.95842 | 0.31744 | 0.36615 | 0.33233 | 0.38387 | 5 | 2.93229 | 0.55262 | 0.28715 | 0.385286 | Not Significant | NS | #N/A |
| 2840 | P52786 | #N/A | CYP2J1 | RABBIT | #N/A | #N/A | #N/A | #N/A | -0.32131 | #N/A | 1.73645 | #N/A | 0.30793 | -1.52049 | 0.495946 | 0.26309 | 0.3618 | 0.81711 | 0.73248 | 0.51745 | 0.52394 | 2 | 2.93229 | 0.55262 | 2.66523 | 1.409264 | Not Significant | NS | #N/A |
| 501 | G1TD16 | Phosphatidylinositol 5-phosphate 4-kinase type-2 a | PIP4K2A | RABBIT | 1.77484 | -1.15944 | -1.64488 | 1.41317 | -0.62297 | -0.47721 | 1.76139 | -0.78461 | 1.26162 | 0.47721 | 0.495535 | 0.58528 | 0.64851 | 0.57449 | 0.53004 | 0.44118 | 0.45461 | 8 | 2.93229 | 0.55262 | 1.79211 | 1.459651 | Not Significant | NS | #N/A |
| 297 | A0A5F9DBM6 | Erythrocyte membrane protein band 4.1 like 3 OS=( | EPB41L3 | RABBIT | -0.86295 | -0.35767 | 0.16179 | 0.41189 | -0.15682 | 0.92032 | 0.16244 | 0.66555 | -0.08868 | 0.00886 | 0.494454 | 1.68055 | 1.41194 | 0.13136 | 0.18579 | 0.21557 | 0.27484 | 8 | 2.93229 | 0.55262 | 0.21642 | 0.306593 | Not Significant | NS | #N/A |

| | | | | | | | | | | | | | | | | | | | | | | | | | | | | | | |
|---|---|---|---|---|---|---|---|---|---|---|---|---|---|---|---|---|---|---|---|---|---|---|---|---|---|---|---|---|---|---|
| 2830 | P00180 | #N/A | CYP2C1 | RABBIT | #N/A | #N/A | #N/A | #N/A | -0.28599 | #N/A | #N/A | 0.20824 | #N/A | #N/A | 0.494233 | #N/A | 0.47012 | #N/A | 0.67102 | #N/A | 0.50325 | 0 | 2.93229 | 0.55262 | #N/A | 0.552617 | Not Significant | NS | | #N/A |
| 1767 | G1SVB0 | #N/A | RPS7 | RABBIT | #N/A | -0.3646 | -0.03315 | 1.33307 | -0.01338 | 1.91742 | 0.4535 | #N/A | #N/A | -0.19711 | 0.494123 | 0.71932 | 0.76555 | 0.50415 | 0.46611 | 0.41624 | 0.4279 | 5 | 2.93229 | 0.55262 | 0.80893 | 0.714182 | Not Significant | NS | | #N/A |
| 2471 | G1SW10 | #N/A | MTA2 | RABBIT | #N/A | #N/A | #N/A | -0.35354 | #N/A | -0.12529 | 0.12966 | 0.06092 | -0.20881 | 0.83413 | 0.491667 | 1.0884 | 0.7791 | 0.33761 | 0.46168 | 0.34068 | 0.42567 | 4 | 2.93229 | 0.55262 | 0.17005 | 0.331873 | Not Significant | NS | | #N/A |
| 1493 | A0A5F9CNW3 | Zinc finger and BTB domain containing 26 OS=Oryct | ZBTB26 | RABBIT | 0.01166 | -0.39384 | #N/A | #N/A | #N/A | #N/A | #N/A | 0.30021 | #N/A | #N/A | 0.491297 | 1.39901 | 0.60962 | 0.39508 | 0.57558 | 0.36874 | 0.46971 | 1 | 2.93229 | 0.55262 | 0.08222 | 0.432991 | Not Significant | NS | | #N/A |
| 1956 | A0A5F9DBT1 | #N/A | UBE3C | RABBIT | #N/A | #N/A | #N/A | #N/A | -0.01338 | 0.6442 | -0.11846 | -0.16237 | 1.5419 | #N/A | 0.489703 | 0.54675 | 0.56654 | 0.6226 | 0.5918 | 0.45769 | 0.47348 | 3 | 2.93229 | 0.55262 | 0.64178 | 0.597707 | Not Significant | NS | | #N/A |
| 1040 | A0A5F9DNW8 | Small nuclear ribonucleoprotein-associated proteir | #N/A | RABBIT | -1.7416 | -0.52413 | 0.25513 | 0.26931 | -0.16119 | -0.53536 | 0.44616 | 0.28427 | -0.08448 | 0.43183 | 0.488979 | 1.17752 | 1.13539 | 0.27283 | 0.28049 | 0.30997 | 0.3349 | 8 | 2.93229 | 0.55262 | 0.43111 | 0.463698 | Not Significant | NS | | #N/A |
| 536 | Q95JE9 | Tropomyosin beta chain OS=Oryctolagus cuniculus | #N/A | RABBIT | -1.60285 | #N/A | 0.932 | -0.93414 | #N/A | #N/A | 0.79938 | 0.59186 | -1.57805 | 0 | 0.488292 | 0.54334 | 0.61605 | 0.61023 | 0.55512 | 0.45404 | 0.46338 | 5 | 2.93229 | 0.55262 | 1.38451 | 1.07699 | Not Significant | NS | | #N/A |
| 2035 | G1T1U0 | #N/A | AFAP1L2 | RABBIT | #N/A | 0.19782 | -0.0951 | #N/A | #N/A | #N/A | 0.539 | #N/A | #N/A | #N/A | 0.487644 | 1.92228 | 0.6122 | 0.30538 | 0.57404 | 0.32498 | 0.46967 | 1 | 2.93229 | 0.55262 | 0.0429 | 0.422994 | Not Significant | NS | | #N/A |
| 1167 | A0A5F9DFC7 | RHO family interacting cell polarization regulator 1 ( | RIPOR1 | RABBIT | -0.35946 | #N/A | #N/A | -0.02847 | -0.04255 | 1.78818 | #N/A | -1.38155 | 0.61938 | #N/A | 0.485501 | 0.52106 | 0.59907 | 0.62984 | 0.56818 | 0.46099 | 0.46793 | 4 | 2.93229 | 0.55262 | 1.30227 | 0.985172 | Not Significant | NS | | #N/A |
| 2209 | G1TA73 | #N/A | FYN | RABBIT | #N/A | #N/A | -0.3797 | #N/A | #N/A | -0.30271 | #N/A | 0.03318 | 0 | 0.68781 | 0.484267 | 1.03831 | 0.72074 | 0.37546 | 0.49847 | 0.35835 | 0.44285 | 3 | 2.93229 | 0.55262 | 0.17402 | 0.361159 | Not Significant | NS | | #N/A |
| 435 | G1T892 | Microtubule associated protein RP/EB family memb | MAPRE3 | RABBIT | -0.16541 | -0.56988 | -0.24969 | 0.37914 | -0.5232 | 0.64905 | -0.02291 | -0.04146 | 0.46046 | 0.2377 | 0.48238 | 2.22516 | 1.57603 | 0.05672 | 0.14349 | 0.15449 | 0.24263 | 8 | 2.93229 | 0.55262 | 0.11749 | 0.2342 | Not Significant | NS | | #N/A |
| 1953 | A5TZV0 | #N/A | #N/A | MT | #N/A | -0.38176 | -0.10728 | 0.103 | -0.01269 | 0.35864 | -0.0845 | #N/A | 1.25192 | -0.00069 | 0.481027 | 1.49062 | 1.2001 | 0.18665 | 0.26096 | 0.26042 | 0.32441 | 6 | 2.93229 | 0.55262 | 0.20828 | 0.321316 | Not Significant | NS | | #N/A |
| 270 | Q75NJ2 | Aldehyde dehydrogenase 1A1 OS=Oryctolagus cuni | aldDH | RABBIT | -1.98263 | -0.99232 | -2.02638 | 2.92814 | 3.79371 | 4.36469 | 0.04219 | -1.41694 | -0.04219 | 1.17741 | 0.480931 | 0.30284 | 0.34846 | 0.76974 | 0.73411 | 0.50494 | 0.52403 | 8 | 2.93229 | 0.55262 | 6.30509 | 4.762145 | Not Significant | NS | | #N/A |
| 1190 | A0A5F9CR48 | Kinesin family member 1B OS=Oryctolagus cunicul | KIF1B | RABBIT | -1.13881 | 0.08291 | -0.07679 | 0.13163 | 0.09287 | -0.10625 | -0.53417 | 0.11048 | 1.26546 | 0.73157 | 0.475059 | 1.19033 | 1.13287 | 0.26804 | 0.2815 | 0.30684 | 0.33517 | 8 | 2.93229 | 0.55262 | 0.3982 | 0.439619 | Not Significant | NS | | #N/A |
| 465 | G1U0B5 | Protein phosphatase methylesterase 1 OS=Oryctol | PPME1 | RABBIT | -0.05725 | -0.75175 | 0.18494 | -0.64423 | -0.02768 | -0.09885 | 0.05802 | 0.45536 | 0.56778 | 0.09598 | 0.474852 | 2.12144 | 1.53295 | 0.06668 | 0.1537 | 0.16176 | 0.25312 | 8 | 2.93229 | 0.55262 | 0.12525 | 0.239883 | Not Significant | NS | | #N/A |
| 2671 | A0A5F9D634 | #N/A | MRPL16 | RABBIT | #N/A | #N/A | #N/A | -0.26512 | #N/A | #N/A | #N/A | #N/A | 0.20748 | #N/A | 0.472604 | #N/A | 0.44954 | #N/A | 0.68419 | #N/A | 0.50892 | 0 | 2.93229 | 0.55262 | #N/A | 0.552617 | Not Significant | NS | | #N/A |
| 2598 | A0A5F9CIC7 | #N/A | #N/A | RABBIT | #N/A | #N/A | #N/A | -0.2011 | #N/A | 0.14346 | 2.46168 | -1.79817 | #N/A | #N/A | 0.470092 | 0.19089 | 0.27618 | 0.86623 | 0.79361 | 0.53174 | 0.53888 | 2 | 2.93229 | 0.55262 | 4.54841 | 2.172878 | Not Significant | NS | | #N/A |
| 327 | G1SNM1 | Glycine--tRNA ligase OS=Oryctolagus cuniculus OX | GARS1 | RABBIT | -0.63121 | -0.57025 | -0.38057 | -0.16779 | 0.48125 | 0.61474 | -0.00036 | 0.04055 | 0.41409 | 0.00036 | 0.467589 | 1.96834 | 1.47434 | 0.08456 | 0.1686 | 0.17453 | 0.26263 | 8 | 2.93229 | 0.55262 | 0.14108 | 0.251464 | Not Significant | NS | | #N/A |
| 1877 | G1SLT8 | #N/A | HNRNPH3 | RABBIT | #N/A | #N/A | -0.32071 | -0.02102 | #N/A | 0.1887 | #N/A | -0.10099 | -0.4338 | 1.52358 | 0.465236 | 0.71571 | 0.71862 | 0.51373 | 0.49587 | 0.42096 | 0.4412 | 4 | 2.93229 | 0.55262 | 0.56339 | 0.558836 | Not Significant | NS | | #N/A |
| 2600 | A0A5F9CMF9 | #N/A | APBB1 | RABBIT | #N/A | #N/A | #N/A | -0.4731 | #N/A | #N/A | #N/A | -0.01587 | 0 | #N/A | 0.465162 | 33.8397 | 0.59163 | 0.01881 | 0.58643 | 0.11224 | 0.47237 | 1 | 2.93229 | 0.55262 | 0.00013 | 0.412116 | Not Significant | NS | | #N/A |
| 1332 | A0A5F9DE71 | Synaptic Ras GTPase activating protein 1 OS=Oryct | SYNGAP1 | RABBIT | -1.26281 | -2.63087 | 1.90532 | -0.54125 | 0.55745 | 1.17055 | 0.90268 | 0.35946 | -0.42383 | -1.66058 | 0.46409 | 0.50045 | 0.55927 | 0.63023 | 0.58725 | 0.46099 | 0.47237 | 8 | 2.93229 | 0.55262 | 2.14992 | 1.721486 | Not Significant | NS | | #N/A |
| 2627 | A0A5F9C5K9 | #N/A | EEFSEC | RABBIT | #N/A | #N/A | #N/A | -0.45454 | #N/A | 0.21186 | 0.29629 | 0.19128 | -0.44104 | -0.21186 | 0.463842 | 1.32544 | 0.78273 | 0.25566 | 0.45968 | 0.30172 | 0.42488 | 4 | 2.93229 | 0.55262 | 0.10206 | 0.292639 | Not Significant | NS | | #N/A |
| 2774 | G1T3X6 | #N/A | TRIM58 | RABBIT | #N/A | #N/A | #N/A | -0.27907 | -0.38538 | #N/A | 0.04237 | -0.06437 | 0.41202 | #N/A | 0.462236 | 2.42645 | 0.93199 | 0.09363 | 0.3877 | 0.18402 | 0.39426 | 3 | 2.93229 | 0.55262 | 0.04355 | 0.295177 | Not Significant | NS | | #N/A |
| 693 | A0AAG1VU42 | Ribosomal protein S16 OS=Oryctolagus cuniculus ( | #N/A | RABBIT | -1.94448 | -1.30836 | 0.42792 | -0.00928 | 0.28593 | -0.26651 | 0.01589 | 0.15591 | -0.13032 | -0.01589 | 0.461472 | 0.96642 | 0.9704 | 0.36214 | 0.35283 | 0.35245 | 0.3755 | 8 | 2.93229 | 0.55262 | 0.57003 | 0.565361 | Not Significant | NS | | #N/A |
| 2792 | A0A5F9D264 | #N/A | ANKS1B | RABBIT | #N/A | #N/A | #N/A | #N/A | -0.01338 | 1.14164 | -0.48356 | -0.09122 | 1.22479 | #N/A | 0.461296 | 0.47716 | 0.51126 | 0.66585 | 0.62763 | 0.47235 | 0.48758 | 3 | 2.93229 | 0.55262 | 0.7477 | 0.65127 | Not Significant | NS | | #N/A |
| 616 | G1TN08 | Hippocalcin-like protein 1 OS=Oryctolagus cunicul | HPCAL1 | RABBIT | -1.69406 | 1.65944 | 1.06345 | -1.22859 | -0.26147 | -0.61297 | -0.45788 | 1.28757 | 0.24808 | 1.37608 | 0.46042 | 0.59915 | 0.6568 | 0.56565 | 0.5249 | 0.43824 | 0.45323 | 8 | 2.93229 | 0.55262 | 1.47629 | 1.228539 | Not Significant | NS | | #N/A |
| 1342 | A0A5F9CBV2 | Breast carcinoma amplified sequence 1 OS=Orycto | BCAS1 | RABBIT | #N/A | -0.16339 | #N/A | #N/A | #N/A | #N/A | #N/A | 0.29684 | #N/A | #N/A | 0.460229 | #N/A | 0.43777 | #N/A | 0.69179 | #N/A | 0.51015 | 0 | 2.93229 | 0.55262 | #N/A | 0.552617 | Not Significant | NS | | #N/A |
| 268 | G1SHY5 | Hyaluronan and proteoglycan link protein 1 OS=Ory | HAPLN1 | RABBIT | -1.58371 | -2.72034 | 0.18084 | -0.03954 | 1.23077 | 0.4065 | -0.01019 | -0.38214 | -1.13818 | 0.48579 | 0.458752 | 0.605 | 0.66212 | 0.56195 | 0.52161 | 0.43729 | 0.45236 | 8 | 2.93229 | 0.55262 | 1.43744 | 1.200111 | Not Significant | NS | | #N/A |
| 2144 | A0A5F9CFH0 | #N/A | FARSB | RABBIT | #N/A | #N/A | -0.74494 | 0.92288 | -1.26558 | 0.38731 | #N/A | 0.01258 | 0 | -0.0161 | 0.458497 | 0.81278 | 0.81083 | 0.45329 | 0.44111 | 0.39325 | 0.41692 | 5 | 2.93229 | 0.55262 | 0.54553 | 0.548147 | Not Significant | NS | | #N/A |
| 2140 | A0A5F9D7C1 | #N/A | RPL26 | RABBIT | #N/A | #N/A | 0.17065 | #N/A | #N/A | 1.5253 | #N/A | #N/A | #N/A | -0.27379 | 0.455105 | 0.2921 | 0.40945 | 0.81908 | 0.70353 | 0.51745 | 0.51495 | 1 | 2.93229 | 0.55262 | 1.61837 | 0.823643 | Not Significant | NS | | #N/A |
| 1550 | P9WM21 | Uncharacterized protein Rv1337 OS=Mycobacteriu | #N/A | MT | -1.02808 | -1.41036 | #N/A | 0.26192 | 0.37392 | #N/A | 0 | #N/A | 0.52253 | -0.51199 | 0.45416 | 0.7706 | 0.78105 | 0.47576 | 0.45745 | 0.40375 | 0.42417 | 5 | 2.93229 | 0.55262 | 0.59545 | 0.579619 | Not Significant | NS | | #N/A |
| 356 | A0A5F9D1N8 | Vinculin OS=Oryctolagus cuniculus OX=9986 GN=V | VCL | RABBIT | 0.80911 | 1.88234 | -4.87453 | 0.43161 | -1.01135 | #N/A | 2.42543 | -1.80724 | -1.0181 | 0 | 0.452587 | 0.29039 | 0.33871 | 0.77994 | 0.74188 | 0.5073 | 0.52755 | 7 | 2.93229 | 0.55262 | 5.3981 | 3.967581 | Not Significant | NS | | #N/A |
| 2785 | A0A5F9DQ62 | #N/A | ARRB1 | RABBIT | #N/A | #N/A | #N/A | #N/A | -0.26476 | #N/A | #N/A | 0.18701 | #N/A | #N/A | 0.451768 | #N/A | 0.42972 | #N/A | 0.69701 | #N/A | 0.51312 | 0 | 2.93229 | 0.55262 | #N/A | 0.552617 | Not Significant | NS | | #N/A |
| 993 | G1TVE5 | Calcium/calmodulin-dependent protein kinase type | CAMK2D | RABBIT | -0.87285 | #N/A | #N/A | -1.30759 | 2.08789 | #N/A | 0.83743 | #N/A | 0 | #N/A | 0.449567 | 0.31836 | 0.40437 | 0.77109 | 0.70012 | 0.50537 | 0.51428 | 3 | 2.93229 | 0.55262 | 2.39291 | 1.483264 | Not Significant | NS | | #N/A |
| 2852 | G1TLM7 | #N/A | #N/A | RABBIT | #N/A | #N/A | #N/A | #N/A | -0.23697 | #N/A | 0.741 | -0.33225 | #N/A | 0.22359 | 0.447747 | 0.72244 | 0.58105 | 0.54508 | 0.58675 | 0.43035 | 0.47237 | 2 | 2.93229 | 0.55262 | 0.28809 | 0.445353 | Not Significant | NS | | #N/A |
| 1352 | A0A5F9CKR9 | Diphthine--ammonia ligase OS=Oryctolagus cunicu | DPH6 | RABBIT | #N/A | -1.11693 | 0.46479 | #N/A | -0.44422 | #N/A | #N/A | -0.06437 | #N/A | 0.22696 | 0.446753 | 0.74268 | 0.69718 | 0.51154 | 0.51207 | 0.42044 | 0.44921 | 3 | 2.93229 | 0.55262 | 0.43422 | 0.492743 | Not Significant | NS | | #N/A |
| 925 | G1SIZ2 | Small ribosomal subunit protein uS10 OS=Oryctola | RPS20 | RABBIT | -1.61741 | -1.27231 | 0.17765 | -0.01482 | 0.05008 | 0.1881 | 0.03623 | -0.11936 | -0.55374 | -0.007 | 0.444207 | 1.11893 | 1.06301 | 0.29564 | 0.31069 | 0.32166 | 0.35218 | 8 | 2.93229 | 0.55262 | 0.39401 | 0.436552 | Not Significant | NS | | #N/A |
| 534 | G1SQ87 | Profilin OS=Oryctolagus cuniculus OX=9986 GN=PF | PFN2 | RABBIT | -0.73883 | -0.76678 | 0.01929 | -0.04554 | 0.0203 | 0.36178 | -0.25984 | 0.19217 | 0.42649 | -0.0121 | 0.444014 | 1.98899 | 1.43486 | 0.0819 | 0.17931 | 0.17426 | 0.26969 | 8 | 2.93229 | 0.55262 | 0.12459 | 0.239394 | Not Significant | NS | | #N/A |
| 130 | A0A5F9C8F6 | Tenascin C OS=Oryctolagus cuniculus OX=9986 GN | TNC | RABBIT | -0.99685 | -0.61578 | 0.93394 | -1.90379 | 1.11538 | -1.36472 | 1.2376 | 0.87563 | -0.60077 | 0.60077 | 0.443119 | 0.58555 | 0.64068 | 0.57432 | 0.53493 | 0.44118 | 0.45675 | 8 | 2.93229 | 0.55262 | 1.43173 | 1.195929 | Not Significant | NS | | #N/A |
| 1878 | A0A5F9CJQ1 | #N/A | #N/A | RABBIT | #N/A | -2.68536 | 0.30978 | #N/A | 0.24849 | #N/A | 0.02489 | -0.06437 | -0.45038 | -0.57893 | 0.441837 | 0.52298 | 0.58571 | 0.62333 | 0.57436 | 0.45777 | 0.46967 | 5 | 2.93229 | 0.55262 | 1.22358 | 0.975547 | Not Significant | NS | | #N/A |
| 1236 | G1TYD9 | Translation initiation factor eIF2B subunit delta OS= | EIF2B4 | RABBIT | 0.23154 | #N/A | -1.18859 | #N/A | #N/A | 1.37693 | 0 | 0.02567 | -0.20639 | -1.38143 | 0.441481 | 0.53582 | 0.58429 | 0.61505 | 0.57526 | 0.45457 | 0.46967 | 5 | 2.93229 | 0.55262 | 0.96981 | 0.815589 | Not Significant | NS | | #N/A |
| 1520 | A0A5F9CW00 | DLG associated protein 4 OS=Oryctolagus cuniculu | #N/A | RABBIT | -0.28226 | #N/A | 0.17065 | -1.08636 | 0.30577 | 0.58028 | 0.33561 | -0.06579 | 0.52342 | -0.28388 | 0.440977 | 1.31315 | 1.12776 | 0.23054 | 0.28593 | 0.2895 | 0.33729 | 7 | 2.93229 | 0.55262 | 0.25061 | 0.339769 | Not Significant | NS | | #N/A |
| 1401 | G1SPS6 | Deoxyhypusine synthase OS=Oryctolagus cuniculu | DHPS | RABBIT | #N/A | 0.2371 | #N/A | -0.43372 | #N/A | #N/A | #N/A | #N/A | -0.03928 | 0.72158 | 0.439457 | 0.86648 | 0.66796 | 0.47757 | 0.53413 | 0.40468 | 0.45675 | 2 | 2.93229 | 0.55262 | 0.25723 | 0.432839 | Not Significant | NS | | #N/A |
| 2651 | A0A5F9CFK3 | #N/A | SLC24A2 | RABBIT | #N/A | #N/A | #N/A | -0.32685 | #N/A | 0.39993 | #N/A | 0.02096 | -0.08533 | #N/A | 0.438704 | 1.48947 | 0.63773 | 0.27481 | 0.55207 | 0.31101 | 0.46295 | 2 | 2.93229 | 0.55262 | 0.06506 | 0.354918 | Not Significant | NS | | #N/A |
| 2597 | G1TWD8 | #N/A | SELENOO | RABBIT | #N/A | #N/A | #N/A | 0.03514 | -0.61573 | 0.70205 | #N/A | -0.15715 | 0.31287 | -0.27805 | 0.435226 | 1.10958 | 0.84691 | 0.32939 | 0.42531 | 0.33706 | 0.41009 | 4 | 2.93229 | 0.55262 | 0.20514 | 0.35212 | Not Significant | NS | | #N/A |
| 2739 | B7NZS7 | #N/A | TSEN34 | RABBIT | #N/A | #N/A | #N/A | 0.06105 | -0.13207 | #N/A | #N/A | #N/A | 0.96309 | -0.16767 | 0.433219 | 0.75531 | 0.63741 | 0.52889 | 0.55227 | 0.42603 | 0.46295 | 2 | 2.93229 | 0.55262 | 0.32897 | 0.461932 | Not Significant | NS | | #N/A |
| 1178 | G1T7R5 | F-box domain-containing protein OS=Oryctolagus c | #N/A | RABBIT | -1.00016 | -1.16231 | 0.79543 | 0.33833 | -0.13318 | #N/A | -0.47635 | 0.05543 | #N/A | 1.01574 | 0.430648 | 0.72313 | 0.74406 | 0.49681 | 0.47596 | 0.41323 | 0.43269 | 6 | 2.93229 | 0.55262 | 0.66499 | 0.628103 | Not Significant | NS | | #N/A |
| 2279 | G1SDT5 | #N/A | KIAA0319 | RABBIT | #N/A | #N/A | -0.16103 | -0.31961 | #N/A | 0.4506 | #N/A | 0.0444 | -0.10876 | 0.37439 | 0.430477 | 2.09609 | 0.96342 | 0.10411 | 0.36773 | 0.19368 | 0.38387 | 4 | 2.93229 | 0.55262 | 0.05624 | 0.2662 | Not Significant | NS | | #N/A |
| 402 | G1TIC9 | Sulfurtransferase OS=Oryctolagus cuniculus OX=9( | TST | RABBIT | -0.35951 | -0.24648 | 0.52449 | -0.06551 | -0.06197 | 1.54242 | -1.64634 | 0.21302 | 1.82332 | 0.00787 | 0.429859 | 0.67252 | 0.71817 | 0.5202 | 0.48771 | 0.42304 | 0.43895 | 8 | 2.93229 | 0.55262 | 1.02137 | 0.89564 | Not Significant | NS | | #N/A |
| 1625 | G1TTE5 | 40S ribosomal protein S26 OS=Oryctolagus cunicul | #N/A | RABBIT | -2.26218 | -1.83195 | 1.26096 | 0.41238 | 0.49012 | 0.5764 | -0.08038 | 0.01229 | -0.21745 | -0.07665 | 0.428973 | 0.60332 | 0.65475 | 0.56301 | 0.52616 | 0.43789 | 0.45323 | 8 | 2.93229 | 0.55262 | 1.26389 | 1.073111 | Not Significant | NS | | #N/A |
| 735 | A0A5F9C5Y4 | Phosphoacetylglucosamine mutase OS=Oryctolagu | PGM3 | RABBIT | 0.01399 | #N/A | #N/A | #N/A | -0.88841 | #N/A | -0.00233 | -1.18319 | 1.07913 | 0.06963 | 0.428013 | 0.57297 | 0.60694 | 0.59734 | 0.56323 | 0.45022 | 0.46674 | 4 | 2.93229 | 0.55262 | 0.74404 | 0.663068 | Not Significant | NS | | #N/A |
| 252 | P29694 | Elongation factor 1-gamma OS=Oryctolagus cunicu | EEF1G | RABBIT | -0.90849 | -0.65361 | -0.04374 | 0.31147 | -0.14415 | 0.00736 | -0.00736 | 0.48926 | 0.07212 | 0.13565 | 0.42711 | 1.8014 | 1.34777 | 0.10932 | 0.205 | 0.19697 | 0.28599 | 8 | 2.93229 | 0.55262 | 0.14054 | 0.251068 | Not Significant | NS | | #N/A |
| 210 | G1STF7 | Serotransferrin OS=Oryctolagus cuniculus OX=998( | #N/A | RABBIT | -0.43027 | 1.28104 | -2.73504 | 0.40921 | -2.17345 | -3.72694 | 2.43841 | 0.37757 | #N/A | #N/A | 0.42605 | 0.25568 | 0.30417 | 0.80674 | 0.76796 | 0.5152 | 0.53416 | 6 | 2.93229 | 0.55262 | 5.20625 | 3.678557 | Not Significant | NS | | #N/A |
| 1655 | G8ZF20 | #N/A | HBA1 HBA | RABBIT | #N/A | 0.43248 | #N/A | #N/A | -0.6602 | -0.23466 | #N/A | #N/A | #N/A | 0.85801 | 0.425535 | 0.55076 | 0.56334 | 0.63711 | 0.59785 | 0.46416 | 0.47523 | 2 | 2.93229 | 0.55262 | 0.59697 | 0.570603 | Not Significant | NS | | #N/A |
| 780 | G1TAF5 | Mammalian ependymin-related protein 1 OS=Oryct | EPDR1 | RABBIT | -0.00721 | 0.08655 | 0.17065 | 0.25226 | -0.89017 | #N/A | #N/A | 0.02668 | 0.6689 | #N/A | 0.425371 | 1.09964 | 0.87318 | 0.32159 | 0.40822 | 0.33441 | 0.40598 | 5 | 2.93229 | 0.55262 | 0.21377 | 0.339027 | Not Significant | NS | | #N/A |
| 701 | G1T8P4 | Large ribosomal subunit protein P1 OS=Oryctolagus | RPLP1 | RABBIT | -1.20476 | -1.01269 | 0.40149 | -0.36656 | 0.17375 | -0.94146 | 0.92228 | 0.1277 | 0.10367 | -0.10367 | 0.423455 | 0.97558 | 0.95367 | 0.35785 | 0.36086 | 0.35078 | 0.38038 | 8 | 2.93229 | 0.55262 | 0.47101 | 0.492899 | Not Significant | NS | | #N/A |
| 2496 | G1T286 | #N/A | VPS29 | RABBIT | #N/A | #N/A | #N/A | -0.16852 | #N/A | 1.24554 | -0.19733 | 0.00427 | 0.2847 | -0.06863 | 0.422228 | 0.66242 | 0.5884 | 0.54392 | 0.57492 | 0.43035 | 0.46967 | 4 | 2.93229 | 0.55262 | 0.33856 | 0.429106 | Not Significant | NS | | #N/A |
| 375 | A0A5F9CI98 | ELKS/RAB6-interacting/CAST family member 1 OS=( | ERC1 | RABBIT | #N/A | 0.46165 | -2.29258 | 0.52743 | -0.46063 | #N/A | 0.60264 | 1.70187 | -2.11974 | -0.26383 | 0.421267 | 0.40471 | 0.46563 | 0.69973 | 0.65263 | 0.48373 | 0.49788 | 6 | 2.93229 | 0.55262 | 2.16704 | 1.637058 | Not Significant | NS | | #N/A |
| 731 | Q28653 | Serine/threonine-protein phosphatase 2A 56 kDa re | PPP2R5D | RABBIT | -1.03993 | -0.11845 | #N/A | 0.69223 | #N/A | 1.49641 | -0.65882 | -0.37469 | 0.31033 | 0.54931 | 0.41989 | 0.67416 | 0.70243 | 0.52532 | 0.50031 | 0.42484 | 0.44331 | 6 | 2.93229 | 0.55262 | 0.72736 | 0.669993 | Not Significant | NS | | #N/A |
| 2818 | A0A5F9C5Y9 | #N/A | GRIP1 | RABBIT | #N/A | #N/A | #N/A | #N/A | -0.21619 | #N/A | 0.20281 | #N/A | #N/A | #N/A | 0.418994 | #N/A | 0.39855 | #N/A | 0.71747 | #N/A | 0.5186 | 0 | 2.93229 | 0.55262 | #N/A | 0.552617 | Not Significant | NS | | #N/A |
| 2575 | A0A5F9D4W4 | #N/A | CDK5RAP3 | RABBIT | #N/A | #N/A | #N/A | -0.23632 | #N/A | #N/A | #N/A | #N/A | #N/A | 0.17868 | 0.414999 | #N/A | 0.39475 | #N/A | 0.71998 | #N/A | 0.51955 | 0 | 2.93229 | 0.55262 | #N/A | 0.552617 | Not Significant | NS | | #N/A |
| 2097 | G1TQA4 | #N/A | EPM2AIP1 | RABBIT | #N/A | -0.37011 | -0.01837 | 1.27285 | -0.01338 | 1.11882 | -1.21673 | -0.49413 | 1.80941 | 1.94465 | 0.414657 | 0.52782 | 0.58156 | 0.61393 | 0.57382 | 0.45457 | 0.46967 | 7 | 2.93229 | 0.55262 | 1.37147 | 1.129724 | Not Significant | NS | | #N/A |
| 1840 | G1TCN7 | #N/A | TRIM3 | RABBIT | #N/A | #N/A | #N/A | -0.33951 | #N/A | 0.28187 | -0.64114 | -1.51626 | 1.25059 | 0.99672 | 0.413867 | 0.32762 | 0.3776 | 0.75963 | 0.71702 | 0.50158 | 0.5185 | 4 | 2.93229 | 0.55262 | 1.32984 | 1.001081 | Not Significant | NS | | #N/A |
| 1552 | G1U391 | Histone-arginine methyltransferase CARM1 OS=Ory | CARM1 | RABBIT | #N/A | #N/A | #N/A | #N/A | -0.12012 | 1.04499 | -0.22381 | -0.19745 | 0.73763 | 0.10674 | 0.413745 | 0.66112 | 0.58136 | 0.54467 | 0.5794 | 0.43035 | 0.47148 | 4 | 2.93229 | 0.55262 | 0.32638 | 0.422075 | Not Significant | NS | | #N/A |
| 2721 | G1ST93 | #N/A | PRPF39 | RABBIT | #N/A | #N/A | #N/A | -0.26755 | #N/A | #N/A | #N/A | 0.14554 | #N/A | #N/A | 0.413094 | #N/A | 0.39294 | #N/A | 0.72118 | #N/A | 0.51998 | 0 | 2.93229 | 0.55262 | #N/A | 0.552617 | Not Significant | NS | | #N/A |
| 530 | O19048 | Poly(rC)-binding protein 1 OS=Oryctolagus cunicul | PCBP1 | RABBIT | -1.51688 | -0.63446 | -0.86439 | 0.46642 | 0.20512 | 0.00892 | -0.38667 | -0.01599 | 0.1154 | -0.00892 | 0.411386 | 1.10783 | 1.02782 | 0.30013 | 0.32624 | 0.32411 | 0.35925 | 8 | 2.93229 | 0.55262 | 0.34474 | 0.4005 | Not Significant | NS | | #N/A |
| 1429 | A0A5F9CDY7 | Dynamin-like GTPase OPA1, mitochondrial OS=Ory | OPA1 | RABBIT | #N/A | #N/A | #N/A | -0.63872 | #N/A | 1.85712 | -2.90453 | #N/A | 0 | 0.12983 | 0.409322 | 0.18545 | 0.2444 | 0.8647 | 0.81516 | 0.53102 | 0.5433 | 3 | 2.93229 | 0.55262 | 3.89719 | 2.243989 | Not Significant | NS | | #N/A |
| 1432 | G1SDX0 | Amino acid transporter OS=Oryctolagus cuniculus ( | SLC1A2 | RABBIT | #N/A | #N/A | -0.58447 | -0.06499 | -0.02912 | 0.35479 | 0.00735 | 0.66785 | 0.07477 | -0.19596 | 0.407956 | 1.70515 | 1.10948 | 0.13905 | 0.2962 | 0.22123 | 0.34518 | 6 | 2.93229 | 0.55262 | 0.10732 | 0.253505 | Not Significant | NS | | #N/A |
| 2728 | A0A5F9CCS8 | #N/A | #N/A | RABBIT | #N/A | #N/A | #N/A | -0.12648 | #N/A | #N/A | #N/A | #N/A | 0.562 | 0 | 0.407481 | 0.83723 | 0.49474 | 0.55626 | 0.64715 | 0.43509 | 0.49556 | 1 | 2.93229 | 0.55262 | 0.15792 | 0.452244 | Not Significant | NS | | #N/A |
| 1200 | A0A5F9DKF7 | SEC24 homolog D, COPII coat complex component | SEC24D | RABBIT | 1.03588 | -0.94261 | #N/A | 0.50354 | -1.73275 | #N/A | 0 | #N/A | 0.98467 | -0.61583 | 0.406932 | 0.47853 | 0.53665 | 0.65247 | 0.60623 | 0.46988 | 0.47901 | 5 | 2.93229 | 0.55262 | 1.23968 | 0.985698 | Not Significant | NS | | #N/A |
| 1730 | G1SI55 | #N/A | #N/A | RABBIT | #N/A | -0.28108 | #N/A | -0.18604 | #N/A | #N/A | 0.12841 | #N/A | 0.21386 | #N/A | 0.404698 | 6.33287 | 0.70428 | 0.02404 | 0.51311 | 0.11931 | 0.44921 | 2 | 2.93229 | 0.55262 | 0.00408 | 0.330191 | Not Significant | NS | | #N/A |
| 219 | G1SMS3 | Actin-related protein 2 OS=Oryctolagus cuniculus ( | ACTR2 | RABBIT | -0.05818 | -0.29541 | -0.48516 | 0.08159 | -0.05773 | -0.01897 | 0.48288 | 0.16901 | 0.55299 | 0.01897 | 0.403955 | 2.60449 | 1.45676 | 0.0314 | 0.1733 | 0.12952 | 0.26553 | 8 | 2.93229 | 0.55262 | 0.06014 | 0.192233 | Not Significant | NS | | #N/A |
| 1056 | G1TU13 | Small ribosomal subunit protein eS17 OS=Oryctola | RPS17 | RABBIT | -1.08267 | -0.57827 | 0.2824 | -0.08417 | 0.01315 | 0.04668 | -0.41029 | 0.05907 | -0.16029 | 1.03202 | 0.40335 | 1.17079 | 1.0551 | 0.27537 | 0.31414 | 0.31141 | 0.35371 | 8 | 2.93229 | 0.55262 | 0.29672 | 0.365356 | Not Significant | NS | | #N/A |
| 2572 | G1U7X2 | #N/A | SF1 | RABBIT | #N/A | #N/A | #N/A | -0.25984 | #N/A | #N/A | -0.29003 | 0.51786 | #N/A | 0.2022 | 0.403178 | 0.85758 | 0.55503 | 0.48149 | 0.6031 | 0.40669 | 0.47806 | 2 | 2.93229 | 0.55262 | 0.16577 | 0.395754 | Not Significant | NS | | #N/A |
| 2831 | P9WPB3 | #N/A | #N/A | MT | #N/A | #N/A | #N/A | #N/A | -0.20824 | #N/A | 0.19485 | #N/A | #N/A | #N/A | 0.403093 | #N/A | 0.38342 | #N/A | 0.72751 | #N/A | 0.5223 | 0 | 2.93229 | 0.55262 | #N/A | 0.552617 | Not Significant | NS | | #N/A |
| 201 | A0A5F9D216 | Gelsolin OS=Oryctolagus cuniculus OX=9986 GN=( | GSN | RABBIT | -0.35184 | -0.43883 | 0.53416 | -0.70176 | 0.85563 | -0.62754 | 0.97964 | 0.78849 | #N/A | #N/A | 0.400725 | 0.73263 | 0.73442 | 0.4914 | 0.48152 | 0.41059 | 0.43475 | 6 | 2.93229 | 0.55262 | 0.56095 | 0.558217 | Not Significant | NS | | #N/A |
| 1324 | A0A5F9C2U6 | non-specific serine/threonine protein kinase OS=Or | MAST4 | RABBIT | 0.0522 | -0.14991 | #N/A | #N/A | #N/A | #N/A | #N/A | 0.74174 | -0.04054 | #N/A | 0.399453 | 0.98879 | 0.63581 | 0.42699 | 0.55323 | 0.38091 | 0.46295 | 2 | 2.93229 | 0.55262 | 0.1632 | 0.394712 | Not Significant | NS | | #N/A |
| 1474 | G1TDT2 | LSM6 homolog, U6 small nuclear RNA and mRNA de | LSM6 | RABBIT | -2.06229 | -0.21965 | 0.20631 | 1.44634 | -0.12806 | 1.48944 | -1.61353 | -0.10002 | 1.04333 | 0.41701 | 0.398715 | 0.51206 | 0.5622 | 0.62245 | 0.58532 | 0.45769 | 0.47237 | 8 | 2.93229 | 0.55262 | 1.51577 | 1.257427 | Not Significant | NS | | #N/A |
| 1845 | A0A5F9C181 | #N/A | CD44 | RABBIT | #N/A | 0.62469 | #N/A | -0.79898 | 0.79946 | #N/A | 3.50967 | -0.06437 | -0.6304 | -0.38883 | 0.398127 | 0.32407 | 0.38479 | 0.759 | 0.7105 | 0.50158 | 0.51642 | 5 | 2.93229 | 0.55262 | 2.5873 | 1.835149 | Not Significant | NS | | #N/A |
| 1151 | A0A5F9D1X9 | histone deacetylase OS=Oryctolagus cuniculus OX | HDAC2 | RABBIT | -2.58683 | -1.74767 | 1.13289 | -0.49341 | 1.22634 | 0.24866 | -1.40569 | 0.57396 | 0.34404 | -0.24866 | 0.396196 | 0.47257 | 0.5231 | 0.64914 | 0.61134 | 0.46873 | 0.48032 | 8 | 2.93229 | 0.55262 | 1.75723 | 1.434125 | Not Significant | NS | | #N/A |
| 516 | A0A5F9D012 | Oxidation resistance protein 1 OS=Oryctolagus cun | OXR1 | RABBIT | 0.56428 | 0.00093 | -2.39993 | 0.39754 | -1.15967 | -1.83172 | 0.21145 | 1.00089 | 0.16051 | -0.16051 | 0.395491 | 0.54303 | 0.59124 | 0.60191 | 0.56638 | 0.45195 | 0.46777 | 8 | 2.93229 | 0.55262 | 1.32609 | 1.118626 | Not Significant | NS | | #N/A |
| 950 | G1T701 | Dihydrolipoamide acetyltransferase component of | #N/A | RABBIT | 0.02617 | #N/A | -0.86744 | #N/A | 0.14499 | #N/A | #N/A | 1.96755 | -0.0145 | -1.46312 | 0.395405 | 0.37859 | 0.44621 | 0.72423 | 0.66905 | 0.49085 | 0.50246 | 4 | 2.93229 | 0.55262 | 1.63619 | 1.177848 | Not Significant | NS | | #N/A |
| 1269 | G1TLF0 | peptidylprolyl isomerase OS=Oryctolagus cuniculu | #N/A | RABBIT | 0.00331 | -0.59987 | -0.12193 | 0.65554 | #N/A | #N/A | #N/A | #N/A | 0.00836 | 0.75068 | 0.395256 | 0.87876 | 0.73139 | 0.42915 | 0.48852 | 0.38146 | 0.43945 | 4 | 2.93229 | 0.55262 | 0.26975 | 0.389399 | Not Significant | NS | | #N/A |
| 2320 | A5U5D0 | #N/A | #N/A | MT | #N/A | #N/A | 0.44006 | -1.13842 | #N/A | #N/A | 0.33851 | -0.06437 | -0.13615 | #N/A | 0.395179 | 0.63903 | 0.60903 | 0.56825 | 0.56508 | 0.43895 | 0.46714 | 3 | 2.93229 | 0.55262 | 0.45891 | 0.505228 | Not Significant | NS | | #N/A |
| 2611 | G1T0B0 | #N/A | C11orf68 | RABBIT | #N/A | #N/A | #N/A | 0.70346 | -0.27227 | 1.74518 | -0.39855 | -0.06437 | 1.79664 | -0.02558 | 0.395069 | 0.46992 | 0.51498 | 0.6582 | 0.62061 | 0.47085 | 0.48441 | 5 | 2.93229 | 0.55262 | 1.00974 | 0.840755 | Not Significant | NS | | #N/A |
| 751 | A0A5F9DI38 | Heterogeneous nuclear ribonucleoprotein A/B OS=( | HNRNPAB | RABBIT | -0.75208 | 0.76488 | -0.8488 | -0.05478 | -0.82443 | -1.0362 | -0.00286 | -0.0075 | 0.3998 | 0.90538 | 0.394766 | 0.88213 | 0.87011 | 0.40343 | 0.40294 | 0.37287 | 0.40266 | 8 | 2.93229 | 0.55262 | 0.50067 | 0.514603 | Not Significant | NS | | #N/A |
| 2039 | G1TAE2 | #N/A | ELAVL1 | RABBIT | #N/A | -0.68348 | 1.05195 | -1.82975 | #N/A | 1.36438 | 1.00006 | -1.35531 | -3.00594 | 1.53394 | 0.394518 | 0.29403 | 0.34521 | 0.77864 | 0.73793 | 0.50703 | 0.52542 | 6 | 2.93229 | 0.55262 | 3.37571 | 2.448949 | Not Significant | NS | | #N/A |
| 2534 | G1U153 | #N/A | #N/A | RABBIT | #N/A | #N/A | #N/A | -0.05764 | #N/A | 1.3335 | #N/A | -0.35315 | -0.55887 | 0.92119 | 0.393305 | 0.37692 | 0.41642 | 0.73131 | 0.69175 | 0.49255 | 0.51015 | 3 | 2.93229 | 0.55262 | 0.87106 | 0.713658 | Not Significant | NS | | #N/A |
| 708 | G1U6A6 | Nuclear receptor binding protein 1 OS=Oryctolagus | NRBP1 | RABBIT | 0.19896 | 0.08406 | -0.90757 | -1.46323 | 0.16097 | #N/A | #N/A | -0.02298 | 0.0866 | -0.04138 | 0.392774 | 0.86785 | 0.81137 | 0.41883 | 0.43824 | 0.3785 | 0.41582 | 6 | 2.93229 | 0.55262 | 0.38406 | 0.439392 | Not Significant | NS | | #N/A |
| 1647 | G1TIB4 | Small ribosomal subunit protein eS28 OS=Oryctola | RPS28 | RABBIT | #N/A | -0.03175 | #N/A | -0.24217 | 0.17115 | #N/A | #N/A | 0.54328 | -0.93302 | 1.46465 | 0.392563 | 0.55414 | 0.5882 | 0.609 | 0.57505 | 0.45404 | 0.46967 | 4 | 2.93229 | 0.55262 | 0.75279 | 0.66812 | Not Significant | NS | | #N/A |
| 1448 | A0A5F9DG91 | Tyrosine-protein kinase receptor OS=Oryctolagus c | NTRK2 | RABBIT | #N/A | -0.15798 | #N/A | 0.61398 | -0.14472 | 1.78854 | -0.78699 | -0.3621 | 1.7031 | 0.13134 | 0.391018 | 0.53393 | 0.5784 | 0.61259 | 0.57728 | 0.45455 | 0.47042 | 6 | 2.93229 | 0.55262 | 1.00561 | 0.856905 | Not Significant | NS | | #N/A |

| | | | | | | | | | | | | | | | | | | | | | | | | | | | | | | |
|---|---|---|---|---|---|---|---|---|---|---|---|---|---|---|---|---|---|---|---|---|---|---|---|---|---|---|---|---|---|---|
| 1303 | A0A5F9DQV6 | 40S ribosomal protein S24 OS=Oryctolagus cunicul | #N/A | RABBIT | #N/A | -2.11317 | 0.25707 | -0.21112 | -0.01003 | #N/A | -0.00336 | 0.05432 | -0.57423 | 0.00958 | 0.390889 | 0.69791 | 0.71198 | 0.51136 | 0.49465 | 0.42044 | 0.44101 | 6 | 2.93229 | 0.55262 | 0.62739 | 0.602843 | Not Significant | NS | | #N/A |
| 2348 | A0A5F9D1K7 | #N/A | SLC25A16 | RABBIT | #N/A | #N/A | 0.17065 | #N/A | -0.34402 | #N/A | #N/A | #N/A | #N/A | 0.30265 | 0.389331 | 0.87349 | 0.47612 | 0.54292 | 0.65923 | 0.43035 | 0.49924 | 1 | 2.93229 | 0.55262 | 0.13244 | 0.445765 | Not Significant | NS | | #N/A |
| 2326 | A0A5F9CRI0 | #N/A | PCSK6 | RABBIT | #N/A | #N/A | -0.52717 | -0.05764 | #N/A | 0.21885 | 0.42667 | #N/A | -0.36064 | #N/A | 0.387366 | 1.10404 | 0.71946 | 0.35018 | 0.4992 | 0.34667 | 0.44285 | 3 | 2.93229 | 0.55262 | 0.14773 | 0.347861 | Not Significant | NS | | #N/A |
| 714 | G1U978 | Cell division control protein 42 homolog OS=Orycto | #N/A | RABBIT | -0.52544 | -0.80709 | -0.33185 | 0.44825 | 0.55718 | 1.29821 | 0.4441 | -0.80295 | 0.76942 | -0.4441 | 0.384727 | 0.81482 | 0.81574 | 0.43876 | 0.43206 | 0.38578 | 0.41269 | 8 | 2.93229 | 0.55262 | 0.55735 | 0.55608 | Not Significant | NS | | #N/A |
| 2023 | A0A5S8IBE0 | #N/A | JPH1 | RABBIT | #N/A | -1.00659 | #N/A | #N/A | 0.31008 | -0.65584 | #N/A | 0.56564 | 0.555 | -0.32346 | 0.383592 | 0.62259 | 0.61084 | 0.56729 | 0.56079 | 0.43867 | 0.46562 | 4 | 2.93229 | 0.55262 | 0.50614 | 0.525797 | Not Significant | NS | | #N/A |
| 805 | G1TXF6 | Large ribosomal subunit protein eL27 OS=Oryctolag | RPL27 | RABBIT | -1.86474 | -1.81445 | 0.90579 | -0.14885 | 0.73344 | 0.09121 | -0.67983 | 0.39752 | -0.21948 | 0.12652 | 0.380951 | 0.60735 | 0.64653 | 0.56046 | 0.53128 | 0.4366 | 0.45545 | 8 | 2.93229 | 0.55262 | 0.98355 | 0.867964 | Not Significant | NS | | #N/A |
| 1638 | P62943 | Peptidyl-prolyl cis-trans isomerase FKBP1A OS=Ory | FKBP1A FK | RABBIT | -2.31851 | 0.26882 | #N/A | -0.11048 | 0.55673 | #N/A | -0.03036 | 0.09963 | 0.03036 | -0.17944 | 0.380908 | 0.58034 | 0.61785 | 0.58281 | 0.5521 | 0.4443 | 0.46295 | 6 | 2.93229 | 0.55262 | 0.86159 | 0.760158 | Not Significant | NS | | #N/A |
| 806 | G1SQM7 | Activated RNA polymerase II transcriptional coactiv | SUB1 | RABBIT | -0.59741 | 0.0457 | 0.01624 | 0.75495 | -0.45731 | -0.51514 | 0.57799 | 0.58274 | 0.15212 | 0.84599 | 0.376309 | 1.11833 | 0.9981 | 0.29588 | 0.33981 | 0.32166 | 0.3676 | 8 | 2.93229 | 0.55262 | 0.28307 | 0.355367 | Not Significant | NS | | #N/A |
| 2237 | G1SSN5 | #N/A | #N/A | RABBIT | #N/A | #N/A | #N/A | -0.21098 | #N/A | -1.06408 | -0.08781 | 0.02344 | 0.13528 | 1.80267 | 0.372882 | 0.32917 | 0.36904 | 0.75854 | 0.72311 | 0.50158 | 0.52073 | 4 | 2.93229 | 0.55262 | 1.06935 | 0.850777 | Not Significant | NS | | #N/A |
| 2718 | G1TIE0 | #N/A | NUDT16 | RABBIT | #N/A | #N/A | #N/A | 0.07559 | #N/A | -0.21872 | #N/A | #N/A | 1.69623 | -0.13323 | 0.372502 | 0.29821 | 0.35999 | 0.79367 | 0.73376 | 0.51175 | 0.52403 | 2 | 2.93229 | 0.55262 | 1.17022 | 0.803048 | Not Significant | NS | | #N/A |
| 26 | A0A5F9C6Y7 | Heat shock cognate 71 kDa protein OS=Oryctolagus | #N/A | RABBIT | -0.33035 | -0.55589 | -0.18426 | -0.14415 | 0.0306 | 0.20927 | 0.1184 | -0.10835 | 0.24106 | 0.20015 | 0.368918 | 3.15258 | 1.4013 | 0.01355 | 0.18887 | 0.09752 | 0.27623 | 8 | 2.93229 | 0.55262 | 0.03423 | 0.173277 | Not Significant | NS | | #N/A |
| 479 | G1TJN8 | Synaptotagmin binding cytoplasmic RNA interacting | SYNCRIP | RABBIT | -0.90029 | -0.04089 | 0.45876 | 0.36851 | -0.44462 | -0.13029 | 0.13029 | 0.69599 | -0.28446 | 0.87393 | 0.368794 | 1.08135 | 0.97049 | 0.31106 | 0.35278 | 0.32881 | 0.3755 | 8 | 2.93229 | 0.55262 | 0.29079 | 0.361014 | Not Significant | NS | | #N/A |
| 2412 | U3KNX5 | #N/A | #N/A | RABBIT | #N/A | #N/A | #N/A | -0.05764 | #N/A | -0.12981 | #N/A | -0.09208 | 0.07448 | 1.381 | 0.366034 | 0.45436 | 0.4473 | 0.68043 | 0.67053 | 0.47768 | 0.50325 | 3 | 2.93229 | 0.55262 | 0.5192 | 0.535718 | Not Significant | NS | | #N/A |
| 2681 | A0A5F9CH72 | #N/A | CTDNEP1 | RABBIT | #N/A | #N/A | #N/A | -0.96528 | 0.10623 | #N/A | #N/A | -0.06437 | #N/A | #N/A | 0.365159 | 0.39351 | 0.39911 | 0.76133 | 0.71054 | 0.50173 | 0.51642 | 1 | 2.93229 | 0.55262 | 0.57406 | 0.55807 | Not Significant | NS | | #N/A |
| 1999 | G1STX7 | #N/A | KYAT3 | RABBIT | #N/A | -1.80069 | #N/A | #N/A | 1.0727 | 0 | #N/A | #N/A | #N/A | #N/A | 0.363994 | 0.14627 | 0.2458 | 0.90753 | 0.81813 | 0.54318 | 0.54372 | 1 | 2.93229 | 0.55262 | 4.12817 | 1.461899 | Not Significant | NS | | #N/A |
| 2085 | G1SXT2 | #N/A | RNF43 | RABBIT | #N/A | 0.92276 | #N/A | -0.53141 | -0.14171 | #N/A | -0.62158 | -0.06437 | 1.48046 | 0.99291 | 0.363646 | 0.53892 | 0.5706 | 0.61306 | 0.58407 | 0.45455 | 0.47236 | 5 | 2.93229 | 0.55262 | 0.78052 | 0.696274 | Not Significant | NS | | #N/A |
| 2667 | G1T3L9 | #N/A | PTAR1 | RABBIT | #N/A | #N/A | #N/A | 0.036 | -0.10702 | -0.18521 | #N/A | #N/A | 0.84034 | #N/A | 0.363076 | 0.70127 | 0.54909 | 0.55575 | 0.60688 | 0.43501 | 0.47901 | 2 | 2.93229 | 0.55262 | 0.26805 | 0.437228 | Not Significant | NS | | #N/A |
| 1014 | A0A5F9DKA1 | Serine/threonine-protein phosphatase 2A 56 kDa re | PPP2R5C | RABBIT | #N/A | -0.87914 | #N/A | 0.16818 | #N/A | -0.08356 | -0.75623 | -0.06437 | 0.72116 | 0.21874 | 0.362628 | 0.74351 | 0.67 | 0.49061 | 0.52188 | 0.41059 | 0.45237 | 5 | 2.93229 | 0.55262 | 0.33982 | 0.418485 | Not Significant | NS | | #N/A |
| 835 | A0A5F9CNC8 | Peptidyl-prolyl cis-trans isomerase OS=Oryctolagus | #N/A | RABBIT | -1.2217 | -0.71842 | -0.11999 | 0.25439 | -0.09155 | -1.58803 | 0.47965 | 0.44747 | 0.49815 | 0.07817 | 0.362536 | 0.75749 | 0.76107 | 0.47048 | 0.46272 | 0.40136 | 0.42567 | 8 | 2.93229 | 0.55262 | 0.57265 | 0.567276 | Not Significant | NS | | #N/A |
| 1091 | G1TZY1 | Amino acid transporter OS=Oryctolagus cuniculus ( | SLC1A3 | RABBIT | #N/A | #N/A | -0.19593 | -0.49941 | 0.02037 | -1.05404 | 0.67015 | 0.99233 | -0.03375 | 0.10841 | 0.361609 | 0.75208 | 0.72028 | 0.48045 | 0.48977 | 0.40608 | 0.43948 | 6 | 2.93229 | 0.55262 | 0.43346 | 0.472575 | Not Significant | NS | | #N/A |
| 1257 | G1SPB3 | Tetratricopeptide repeat domain 9 OS=Oryctolagus | #N/A | RABBIT | -0.4628 | #N/A | -0.05012 | 1.15135 | -0.76975 | 0.74634 | -0.19683 | 0.93872 | 0.14765 | 0 | 0.360008 | 0.80722 | 0.77896 | 0.4461 | 0.45416 | 0.38942 | 0.42274 | 7 | 2.93229 | 0.55262 | 0.44201 | 0.474663 | Not Significant | NS | | #N/A |
| 383 | G1SUU7 | RNA-splicing ligase RtcB homolog OS=Oryctolagus | RTCB | RABBIT | -0.52198 | -0.54547 | -0.05885 | 0.92429 | -0.22646 | 0.39505 | -0.38403 | 0.4238 | 0.21308 | 0.71699 | 0.358674 | 1.10364 | 0.97053 | 0.30184 | 0.35277 | 0.32467 | 0.3755 | 8 | 2.93229 | 0.55262 | 0.26405 | 0.341448 | Not Significant | NS | | #N/A |
| 2532 | A0A5F9DQR4 | #N/A | VAPA | RABBIT | #N/A | #N/A | #N/A | -0.05764 | #N/A | 0.75785 | -0.15799 | #N/A | #N/A | #N/A | 0.357571 | 0.45083 | 0.40536 | 0.73036 | 0.70629 | 0.49252 | 0.51597 | 1 | 2.93229 | 0.55262 | 0.41938 | 0.518735 | Not Significant | NS | | #N/A |
| 1556 | A0A5F9CYK3 | Heterochromatin protein 1-binding protein 3 OS=Or | HP1BP3 | RABBIT | -2.01219 | #N/A | 1.41143 | -0.43544 | #N/A | 0.05231 | -0.48095 | -0.06437 | #N/A | 0.53638 | 0.356243 | 0.41233 | 0.46391 | 0.69718 | 0.65517 | 0.4833 | 0.49837 | 5 | 2.93229 | 0.55262 | 1.27963 | 1.010881 | Not Significant | NS | | #N/A |
| 419 | A0A5F9DDZ7 | Filamin C OS=Oryctolagus cuniculus OX=9986 GN= | FLNC | RABBIT | -1.28103 | -0.85979 | 1.48511 | -0.93176 | -0.00913 | -3.55234 | 2.47814 | 0.57082 | 0.68568 | -0.00426 | 0.354928 | 0.32072 | 0.3631 | 0.75664 | 0.72345 | 0.50107 | 0.52073 | 8 | 2.93229 | 0.55262 | 3.06178 | 2.388763 | Not Significant | NS | | #N/A |
| 2065 | G1T2W1 | #N/A | SRP72 | RABBIT | #N/A | #N/A | #N/A | -0.23778 | #N/A | 0.78906 | -0.56836 | 0.40994 | -0.23074 | 0.18014 | 0.353781 | 0.60706 | 0.51253 | 0.57658 | 0.6242 | 0.44179 | 0.4858 | 4 | 2.93229 | 0.55262 | 0.28303 | 0.397061 | Not Significant | NS | | #N/A |
| 2669 | A0A5F9DDE8 | #N/A | KCNMA1 | RABBIT | #N/A | #N/A | #N/A | -0.20395 | #N/A | 0.14631 | #N/A | #N/A | #N/A | #N/A | 0.350269 | #N/A | 0.33318 | #N/A | 0.7614 | #N/A | 0.53262 | 0 | 2.93229 | 0.55262 | #N/A | 0.552617 | Not Significant | NS | | #N/A |
| 429 | G1TFZ6 | Septin-type G domain-containing protein OS=Oryct | #N/A | RABBIT | 0.30179 | -0.15269 | -0.2669 | -0.31895 | 0.15537 | -0.0863 | 1.50748 | 0.31482 | 0.0863 | -0.35759 | 0.349218 | 1.01213 | 0.91268 | 0.34111 | 0.3811 | 0.34374 | 0.39096 | 8 | 2.93229 | 0.55262 | 0.29762 | 0.366015 | Not Significant | NS | | #N/A |
| 573 | G1SKZ8 | Large ribosomal subunit protein uL1 OS=Oryctolagu | RPL10A | RABBIT | -1.66106 | -1.72272 | 1.19517 | -0.00466 | 0.75627 | -0.19074 | -0.05298 | 0.42873 | 0.15307 | -0.05315 | 0.344387 | 0.56061 | 0.59462 | 0.59041 | 0.5642 | 0.44684 | 0.46687 | 8 | 2.93229 | 0.55262 | 0.94342 | 0.838595 | Not Significant | NS | | #N/A |
| 2767 | G1T773 | #N/A | #N/A | RABBIT | #N/A | #N/A | #N/A | -0.39204 | #N/A | #N/A | -0.33423 | -0.06437 | 0.03862 | 0.16804 | 0.344051 | 1.44333 | 0.56548 | 0.24464 | 0.59247 | 0.29685 | 0.4738 | 3 | 2.93229 | 0.55262 | 0.04546 | 0.296143 | Not Significant | NS | | #N/A |
| 774 | A0A5F9C396 | 5'-3' exonuclease PLD3 OS=Oryctolagus cuniculus | PLD3 | RABBIT | -0.9067 | -1.48361 | 0.33333 | 0.8941 | -0.17606 | 0.32971 | -1.26495 | 0.68597 | 0.98199 | -0.35749 | 0.342837 | 0.58583 | 0.61585 | 0.57414 | 0.5506 | 0.44118 | 0.46295 | 8 | 2.93229 | 0.55262 | 0.85618 | 0.774759 | Not Significant | NS | | #N/A |
| 2533 | A0A5F9DGB6 | #N/A | ACSF2 | RABBIT | #N/A | #N/A | #N/A | -0.20015 | #N/A | #N/A | 0.14251 | #N/A | #N/A | #N/A | 0.342665 | #N/A | 0.32594 | #N/A | 0.76634 | #N/A | 0.5339 | 0 | 2.93229 | 0.55262 | #N/A | 0.552617 | Not Significant | NS | | #N/A |
| 829 | G1TCM5 | Protocadherin 1 OS=Oryctolagus cuniculus OX=998 | PCDH1 | RABBIT | -0.60947 | 0.18516 | #N/A | -0.04498 | #N/A | #N/A | 0.18601 | #N/A | #N/A | #N/A | 0.342439 | 0.72532 | 0.47107 | 0.54364 | 0.65769 | 0.43035 | 0.49873 | 2 | 2.93229 | 0.55262 | 0.16717 | 0.396323 | Not Significant | NS | | #N/A |
| 2381 | G1U4R8 | #N/A | SYDE2 | RABBIT | #N/A | #N/A | 0.17065 | #N/A | #N/A | #N/A | #N/A | 1.57873 | #N/A | -0.55678 | 0.340324 | 0.18402 | 0.279 | 0.88415 | 0.7943 | 0.53681 | 0.53895 | 1 | 2.93229 | 0.55262 | 2.28021 | 0.991954 | Not Significant | NS | | #N/A |
| 1634 | G1T4F8 | Protein SCAI OS=Oryctolagus cuniculus OX=9986 G | SCAI | RABBIT | 0.36043 | -1.42939 | -0.75266 | 1.14482 | -0.16559 | 0.05365 | -0.05365 | -1.45151 | 1.22191 | 1.08673 | 0.339907 | 0.51909 | 0.55651 | 0.61775 | 0.58907 | 0.45596 | 0.4724 | 8 | 2.93229 | 0.55262 | 1.07193 | 0.932637 | Not Significant | NS | | #N/A |
| 2306 | G1T0V4 | #N/A | SNRPG | RABBIT | #N/A | #N/A | #N/A | -0.24811 | -0.01672 | #N/A | 0.00334 | 0.12172 | 0.73419 | -0.03037 | 0.339636 | 1.22527 | 0.72469 | 0.28769 | 0.49237 | 0.31864 | 0.43991 | 4 | 2.93229 | 0.55262 | 0.10245 | 0.292864 | Not Significant | NS | | #N/A |
| 2125 | G1U8M6 | #N/A | ATP1A2 | RABBIT | #N/A | #N/A | -0.41392 | -1.6566 | 1.00803 | -0.28257 | 0.333 | 0.26727 | -0.67339 | 0.28257 | 0.339539 | 0.54604 | 0.56869 | 0.60473 | 0.58358 | 0.45322 | 0.47236 | 6 | 2.93229 | 0.55262 | 0.72498 | 0.668397 | Not Significant | NS | | #N/A |
| 2158 | G1T846 | #N/A | DARS1 | RABBIT | #N/A | #N/A | -0.62386 | 0.1215 | #N/A | 0.84726 | -0.43055 | 0.02007 | -0.08443 | #N/A | 0.339267 | 0.72825 | 0.61885 | 0.5068 | 0.55579 | 0.41787 | 0.46338 | 4 | 2.93229 | 0.55262 | 0.28937 | 0.400723 | Not Significant | NS | | #N/A |
| 2016 | A0A5F9D116 | #N/A | VAC14 | RABBIT | #N/A | -0.17329 | #N/A | -0.05764 | #N/A | 0.78678 | #N/A | 0.01369 | #N/A | -0.13267 | 0.338068 | 0.91184 | 0.62019 | 0.42908 | 0.55819 | 0.38146 | 0.46503 | 3 | 2.93229 | 0.55262 | 0.16495 | 0.35657 | Not Significant | NS | | #N/A |
| 2588 | G1SP00 | #N/A | VPS33B | RABBIT | #N/A | #N/A | #N/A | -0.10969 | #N/A | #N/A | -0.04754 | 0.68479 | #N/A | 0.04754 | 0.337949 | 0.73497 | 0.46696 | 0.53886 | 0.66043 | 0.42845 | 0.49949 | 2 | 2.93229 | 0.55262 | 0.15857 | 0.392836 | Not Significant | NS | | #N/A |
| 696 | G1TKJ4 | Neurocalcin-delta OS=Oryctolagus cuniculus OX=9 | NCALD | RABBIT | -1.76769 | -1.41646 | 2.60769 | 0.0816 | -1.07578 | -0.13924 | -1.47082 | 0.49891 | 0.83713 | 0.39181 | 0.337685 | 0.37891 | 0.42194 | 0.71461 | 0.68125 | 0.48903 | 0.50745 | 8 | 2.93229 | 0.55262 | 1.98561 | 1.601246 | Not Significant | NS | | #N/A |
| 1511 | G1U3G4 | Metallo-beta-lactamase domain containing 2 OS=O | MBLAC2 | RABBIT | -1.56936 | #N/A | 0.70613 | -0.81393 | 0.38184 | 0 | 0.01233 | 0.82786 | -0.17534 | -0.59819 | 0.337162 | 0.63226 | 0.64431 | 0.5473 | 0.53397 | 0.43118 | 0.45675 | 7 | 2.93229 | 0.55262 | 0.63193 | 0.608516 | Not Significant | NS | | #N/A |
| 320 | G1T7T6 | General vesicular transport factor p115 OS=Oryctol | USO1 | RABBIT | -0.09142 | -0.75949 | -0.03249 | 0.16758 | 0.0897 | -0.21406 | 1.17056 | -0.80791 | 0.55537 | 0.35525 | 0.337065 | 0.89717 | 0.83595 | 0.39582 | 0.42108 | 0.36911 | 0.4086 | 8 | 2.93229 | 0.55262 | 0.35287 | 0.406449 | Not Significant | NS | | #N/A |
| 1282 | G1TE35 | NAD-capped RNA hydrolase NUDT12 OS=Oryctolag | NUDT12 | RABBIT | #N/A | -1.19054 | #N/A | 0.7048 | #N/A | #N/A | 0.94549 | #N/A | -0.76244 | #N/A | 0.334392 | 0.26213 | 0.33635 | 0.81775 | 0.75045 | 0.51745 | 0.52952 | 2 | 2.93229 | 0.55262 | 1.62734 | 0.988407 | Not Significant | NS | | #N/A |
| 606 | G1SH60 | Propionate--CoA ligase OS=Oryctolagus cuniculus ( | ACSS2 | RABBIT | -0.18642 | 0.53865 | -0.72861 | 1.73624 | -0.21897 | 0.63423 | -0.27828 | -0.17569 | 2.52063 | 0.11132 | 0.334267 | 0.49961 | 0.53742 | 0.6308 | 0.60174 | 0.46117 | 0.47764 | 8 | 2.93229 | 0.55262 | 1.11909 | 0.967149 | Not Significant | NS | | #N/A |
| 887 | G1SQ43 | E2 domain-containing protein OS=Oryctolagus cuni | #N/A | RABBIT | 0.45601 | 0.02319 | -1.45077 | 0.77556 | -0.5704 | #N/A | 0.47908 | 0.98653 | 0 | -0.74199 | 0.334186 | 0.60508 | 0.62229 | 0.56422 | 0.54777 | 0.4379 | 0.46295 | 7 | 2.93229 | 0.55262 | 0.67786 | 0.640882 | Not Significant | NS | | #N/A |
| 2716 | A5U2E6 | #N/A | #N/A | MT | #N/A | #N/A | #N/A | -0.19564 | #N/A | #N/A | #N/A | #N/A | 0.138 | #N/A | 0.333649 | #N/A | 0.31737 | #N/A | 0.77222 | #N/A | 0.53484 | 0 | 2.93229 | 0.55262 | #N/A | 0.552617 | Not Significant | NS | | #N/A |
| 1077 | G1SZT8 | Protein SEC13 homolog OS=Oryctolagus cuniculus | SEC13 | RABBIT | -1.7412 | -0.7001 | -0.00675 | 0.90813 | 0.16402 | -0.19992 | 1.14526 | 0.19736 | 0.3078 | -1.15886 | 0.333507 | 0.5717 | 0.60063 | 0.58323 | 0.56033 | 0.44439 | 0.46562 | 8 | 2.93229 | 0.55262 | 0.85077 | 0.770798 | Not Significant | NS | | #N/A |
| 643 | G1SJX1 | Serine/threonine-protein phosphatase 2A 56 kDa re | PPP2R5E | RABBIT | 0.20194 | -1.40689 | -0.65781 | 0.9358 | -0.2214 | 1.01937 | -1.27662 | -0.2245 | 0.8403 | 0.16013 | 0.333409 | 0.58434 | 0.61125 | 0.57509 | 0.55353 | 0.44118 | 0.46295 | 8 | 2.93229 | 0.55262 | 0.81388 | 0.743804 | Not Significant | NS | | #N/A |
| 2620 | A0A5F9CMJ7 | #N/A | MVK | RABBIT | #N/A | #N/A | #N/A | -0.05764 | #N/A | 0.97338 | #N/A | -0.42288 | #N/A | #N/A | 0.332892 | 0.2753 | 0.33458 | 0.82897 | 0.75503 | 0.52053 | 0.53124 | 1 | 2.93229 | 0.55262 | 0.97477 | 0.659971 | Not Significant | NS | | #N/A |
| 1654 | A0A5F9D7Z0 | #N/A | MACF1 | RABBIT | #N/A | -0.66282 | 0.98066 | -0.37179 | #N/A | #N/A | 0.31416 | #N/A | #N/A | #N/A | 0.33214 | 0.32798 | 0.35944 | 0.77408 | 0.73414 | 0.50625 | 0.52403 | 2 | 2.93229 | 0.55262 | 0.76914 | 0.640416 | Not Significant | NS | | #N/A |
| 514 | G1TE96 | Glutathione S-transferase LANCL1 OS=Oryctolagus | LANCL1 | RABBIT | -0.36646 | -1.12667 | 0.12407 | 0.51202 | 0.00329 | 0.65699 | -0.51962 | 0.39236 | 0.28754 | -0.01668 | 0.330872 | 0.96611 | 0.86855 | 0.36228 | 0.40376 | 0.35245 | 0.40294 | 8 | 2.93229 | 0.55262 | 0.29323 | 0.362802 | Not Significant | NS | | #N/A |
| 2749 | A0A5F9DHK3 | #N/A | #N/A | RABBIT | #N/A | #N/A | #N/A | -0.18665 | -0.01338 | 0.38179 | #N/A | #N/A | 0.32399 | -0.01865 | 0.329061 | 1.89363 | 0.66767 | 0.1546 | 0.52945 | 0.23523 | 0.45433 | 3 | 2.93229 | 0.55262 | 0.03624 | 0.291479 | Not Significant | NS | | #N/A |
| 1749 | G1SHZ4 | #N/A | KRT7 | RABBIT | #N/A | -1.19144 | -0.38088 | #N/A | 2.12441 | 2.7978 | -1.82873 | 0.1866 | -0.25097 | 1.65283 | 0.327479 | 0.25421 | 0.29753 | 0.80782 | 0.77287 | 0.5152 | 0.53484 | 6 | 2.93229 | 0.55262 | 3.11158 | 2.271527 | Not Significant | NS | | #N/A |
| 2074 | G1SXA0 | #N/A | GNG3 | RABBIT | #N/A | -0.86646 | #N/A | -0.05764 | 0.64195 | 0.89611 | #N/A | #N/A | -0.43059 | #N/A | 0.326808 | 0.43632 | 0.45702 | 0.6921 | 0.66391 | 0.4819 | 0.50024 | 3 | 2.93229 | 0.55262 | 0.67323 | 0.613612 | Not Significant | NS | | #N/A |
| 2608 | G1T096 | #N/A | SNRPF | RABBIT | #N/A | #N/A | #N/A | -0.53305 | -0.04869 | #N/A | 0.03531 | 0.07179 | -0.19244 | 0.21365 | 0.322948 | 1.65833 | 0.72725 | 0.17259 | 0.4909 | 0.2511 | 0.43974 | 4 | 2.93229 | 0.55262 | 0.05057 | 0.262928 | Not Significant | NS | | #N/A |
| 460 | G1T2N8 | Exportin-T OS=Oryctolagus cuniculus OX=9986 GN | XPOT | RABBIT | -0.3892 | -0.93787 | -0.23514 | 0.43322 | 0.34469 | 0.56829 | -0.15321 | 0.08885 | 0.57039 | -0.24401 | 0.322923 | 1.05575 | 0.90347 | 0.32192 | 0.38575 | 0.33441 | 0.39334 | 8 | 2.93229 | 0.55262 | 0.23389 | 0.319383 | Not Significant | NS | | #N/A |
| 2477 | G1SNA3 | #N/A | STXBP6 | RABBIT | #N/A | #N/A | -0.40455 | -0.01963 | -0.08117 | 0 | #N/A | #N/A | 0.3072 | #N/A | 0.322044 | 1.67743 | 0.64895 | 0.19205 | 0.54066 | 0.26339 | 0.4607 | 3 | 2.93229 | 0.55262 | 0.04423 | 0.295522 | Not Significant | NS | | #N/A |
| 2437 | A0A5F9DH83 | #N/A | GPBP1 | RABBIT | #N/A | #N/A | 0.17065 | -0.61678 | #N/A | 0.09897 | #N/A | #N/A | #N/A | #N/A | 0.322029 | 0.47223 | 0.37527 | 0.71913 | 0.72682 | 0.49014 | 0.52228 | 1 | 2.93229 | 0.55262 | 0.31002 | 0.490923 | Not Significant | NS | | #N/A |
| 2247 | A0A5F9DBS2 | #N/A | CCDC136 | RABBIT | #N/A | #N/A | 0.11586 | -0.262 | -0.17414 | 0.24692 | 0.33243 | 0.27459 | #N/A | 0 | 0.320245 | 2.4813 | 0.88935 | 0.05575 | 0.39998 | 0.15432 | 0.40151 | 5 | 2.93229 | 0.55262 | 0.02856 | 0.222283 | Not Significant | NS | | #N/A |
| 2771 | A0A5K1V4A1 | #N/A | MGMT | RABBIT | #N/A | #N/A | #N/A | -0.24193 | #N/A | #N/A | #N/A | #N/A | 0.15452 | 0 | 0.319183 | 2.38527 | 0.40449 | 0.25273 | 0.70689 | 0.30043 | 0.51618 | 1 | 2.93229 | 0.55262 | 0.01194 | 0.415119 | Not Significant | NS | | #N/A |
| 2426 | G1SKU1 | #N/A | GOLGA1 | RABBIT | #N/A | #N/A | -0.07253 | #N/A | #N/A | #N/A | #N/A | #N/A | #N/A | 0.24318 | 0.315704 | #N/A | 0.3003 | #N/A | 0.78398 | #N/A | 0.53801 | 0 | 2.93229 | 0.55262 | #N/A | 0.552617 | Not Significant | NS | | #N/A |
| 1863 | A0A5F9D097 | #N/A | RGS6 | RABBIT | #N/A | -0.79566 | #N/A | #N/A | 0.19247 | #N/A | #N/A | 0.2329 | -0.20585 | #N/A | 0.315122 | 0.58294 | 0.47131 | 0.61891 | 0.65753 | 0.45659 | 0.49873 | 2 | 2.93229 | 0.55262 | 0.29222 | 0.447029 | Not Significant | NS | | #N/A |
| 2683 | G1TPB0 | #N/A | TKTL2 | RABBIT | #N/A | #N/A | #N/A | -0.18529 | #N/A | #N/A | #N/A | #N/A | #N/A | 0.12765 | 0.312937 | #N/A | 0.29767 | #N/A | 0.7858 | #N/A | 0.53841 | 0 | 2.93229 | 0.55262 | #N/A | 0.552617 | Not Significant | NS | | #N/A |
| 980 | A0A5F9CF46 | Golgin B1 OS=Oryctolagus cuniculus OX=9986 GN= | GOLGB1 | RABBIT | -2.17075 | -1.83965 | 0.57545 | 0.4099 | 1.68419 | #N/A | 1.71601 | -0.06437 | -0.8642 | -0.60996 | 0.312543 | 0.31688 | 0.35874 | 0.76057 | 0.7273 | 0.50173 | 0.5223 | 7 | 2.93229 | 0.55262 | 2.16178 | 1.686707 | Not Significant | NS | | #N/A |
| 1453 | G1TRL8 | Small nuclear ribonucleoprotein Sm D1 OS=Oryctol | SNRPD1 | RABBIT | -1.03061 | -0.97081 | 0.60347 | -0.22204 | -0.24915 | -1.44647 | 0.1644 | 0.46971 | 0.24299 | 0.25477 | 0.310907 | 0.6764 | 0.67225 | 0.51786 | 0.51538 | 0.42184 | 0.4502 | 8 | 2.93229 | 0.55262 | 0.52819 | 0.534743 | Not Significant | NS | | #N/A |
| 83 | G1U0U5 | Heat shock protein family A (Hsp70) member 4 like | HSPA4L | RABBIT | 0.42202 | -0.60015 | -0.00496 | -0.58141 | -0.00377 | 0.42082 | 0.14031 | -0.07398 | 0.39743 | -0.10059 | 0.310451 | 1.38444 | 1.00152 | 0.20361 | 0.33823 | 0.27183 | 0.36703 | 8 | 2.93229 | 0.55262 | 0.12571 | 0.240218 | Not Significant | NS | | #N/A |
| 1575 | G1T2K5 | MYB binding protein 1a OS=Oryctolagus cuniculus ( | MYBBP1A | RABBIT | 0.22218 | #N/A | #N/A | -0.71007 | #N/A | 0.75957 | 0 | -0.35324 | -0.12347 | 0.046 | 0.309715 | 0.77799 | 0.62843 | 0.47176 | 0.54739 | 0.40175 | 0.46295 | 5 | 2.93229 | 0.55262 | 0.2264 | 0.34699 | Not Significant | NS | | #N/A |
| 377 | G1SFC1 | Golgin A4 OS=Oryctolagus cuniculus OX=9986 GN= | GOLGA4 | RABBIT | -0.49598 | -1.90976 | 0.26983 | -0.66598 | 1.15104 | #N/A | 0.88343 | 0.48389 | -1.45529 | 0 | 0.308178 | 0.42139 | 0.45922 | 0.68611 | 0.65596 | 0.48039 | 0.4985 | 7 | 2.93229 | 0.55262 | 1.18856 | 1.000809 | Not Significant | NS | | #N/A |
| 1087 | G1SJF5 | Microtubule associated protein 6 OS=Oryctolagus c | #N/A | RABBIT | 0.42121 | 0.32107 | -0.45871 | -0.18089 | -0.44456 | 0.78636 | -0.50327 | 0.3463 | -0.2227 | 0.78959 | 0.307634 | 0.95478 | 0.83647 | 0.36764 | 0.4208 | 0.35413 | 0.4086 | 8 | 2.93229 | 0.55262 | 0.25954 | 0.338148 | Not Significant | NS | | #N/A |
| 2344 | G1TVQ4 | #N/A | FSD1 | RABBIT | #N/A | #N/A | 0.17065 | #N/A | #N/A | 1.04728 | #N/A | #N/A | #N/A | -0.09194 | 0.307018 | 0.31119 | 0.32998 | 0.80794 | 0.75824 | 0.5152 | 0.53209 | 1 | 2.93229 | 0.55262 | 0.64891 | 0.577105 | Not Significant | NS | | #N/A |
| 631 | A0A5F9DW54 | Phosphodiesterase OS=Oryctolagus cuniculus OX= | PDE1A | RABBIT | 0.10377 | 0.10571 | -0.53483 | 0.32741 | -0.51532 | -0.18719 | 0.56717 | 0.36978 | 0.87241 | -0.6025 | 0.306591 | 0.96052 | 0.83802 | 0.36492 | 0.41996 | 0.354 | 0.40859 | 8 | 2.93229 | 0.55262 | 0.25471 | 0.334614 | Not Significant | NS | | #N/A |
| 2814 | G1STX4 | #N/A | SRP54 | RABBIT | #N/A | #N/A | #N/A | #N/A | -0.60165 | 0.58827 | -1.89473 | -1.85689 | 0.63913 | 1.0358 | 0.303968 | 0.19123 | 0.23054 | 0.85766 | 0.82433 | 0.52914 | 0.5459 | 4 | 2.93229 | 0.55262 | 2.1056 | 1.448705 | Not Significant | NS | | #N/A |
| 1921 | A0A5F9CK37 | #N/A | #N/A | RABBIT | #N/A | 0.49726 | #N/A | -1.10315 | #N/A | #N/A | 0.13991 | #N/A | #N/A | -0.13991 | 0.302944 | 0.37293 | 0.39237 | 0.74502 | 0.71117 | 0.49742 | 0.51654 | 2 | 2.93229 | 0.55262 | 0.6599 | 0.59612 | Not Significant | NS | | #N/A |
| 1287 | G1SNV4 | Splicing factor 3a subunit 3 OS=Oryctolagus cunicu | SF3A3 | RABBIT | -0.07691 | -1.66883 | 0.53676 | 0.52694 | -0.31152 | #N/A | 0 | 0.66387 | -0.53404 | 0.28664 | 0.302827 | 0.59562 | 0.59899 | 0.57018 | 0.56259 | 0.43972 | 0.46666 | 7 | 2.93229 | 0.55262 | 0.57443 | 0.567992 | Not Significant | NS | | #N/A |
| 704 | G1T7S0 | Myosin light chain 12A OS=Oryctolagus cuniculus C | MYL12A | RABBIT | 0.09193 | #N/A | #N/A | -1.17984 | 0.06225 | -1.0419 | 0.96311 | -0.14 | 0.45059 | -0.42889 | 0.302467 | 0.54496 | 0.54887 | 0.60543 | 0.59655 | 0.45328 | 0.47506 | 6 | 2.93229 | 0.55262 | 0.57761 | 0.569403 | Not Significant | NS | | #N/A |
| 1187 | Q9Q8G7 | M134R OS=Myxoma virus (strain Lausanne) OX=31 | m134R | RABBIT | -0.48414 | -0.20154 | #N/A | -0.04196 | -0.02635 | #N/A | 0.27221 | -0.0514 | #N/A | #N/A | 0.298896 | 1.59326 | 0.67579 | 0.18632 | 0.52107 | 0.26042 | 0.45235 | 4 | 2.93229 | 0.55262 | 0.04693 | 0.260828 | Not Significant | NS | | #N/A |
| 2212 | G1SYM3 | #N/A | #N/A | RABBIT | #N/A | #N/A | 0.59206 | -1.06317 | 0.35922 | 0.01748 | 2.32889 | -0.39207 | -0.01748 | -0.63138 | 0.298386 | 0.37191 | 0.4102 | 0.72274 | 0.69133 | 0.49079 | 0.51015 | 6 | 2.93229 | 0.55262 | 1.20694 | 0.992136 | Not Significant | NS | | #N/A |
| 2170 | G1SXE6 | #N/A | DYNC1LI1 | RABBIT | #N/A | #N/A | -0.69939 | -0.05764 | #N/A | 0.51914 | -1.17121 | -0.03502 | -0.07389 | 0.35989 | 0.2983 | 0.57074 | 0.53134 | 0.59287 | 0.60974 | 0.448 | 0.47977 | 5 | 2.93229 | 0.55262 | 0.39024 | 0.450266 | Not Significant | NS | | #N/A |
| 49 | A0A5F9DP24 | Creatine kinase B-type OS=Oryctolagus cuniculus ( | #N/A | RABBIT | -1.48181 | 0.15527 | -0.00476 | -0.01509 | 0.0564 | 0.46064 | -0.45133 | 0.15321 | -0.05904 | 0.09786 | 0.298263 | 0.87295 | 0.78404 | 0.40813 | 0.44967 | 0.3744 | 0.42107 | 8 | 2.93229 | 0.55262 | 0.29185 | 0.361793 | Not Significant | NS | | #N/A |
| 861 | P19949 | Large ribosomal subunit protein uL18 OS=Oryctolag | RPL5 | RABBIT | -1.86157 | -0.29353 | 0.51006 | -0.12459 | 0.35437 | -0.23103 | 0.06695 | 0.24894 | -0.24274 | 0.22797 | 0.29707 | 0.68314 | 0.66817 | 0.51381 | 0.51788 | 0.42096 | 0.45118 | 8 | 2.93229 | 0.55262 | 0.47275 | 0.494172 | Not Significant | NS | | #N/A |
| 2012 | G1T6D1 | #N/A | RPL23 | RABBIT | #N/A | #N/A | -0.08105 | 0.34587 | -0.95266 | 0.2517 | -0.5162 | 0.67569 | -0.61903 | 0.54516 | 0.296745 | 0.65345 | 0.61177 | 0.53769 | 0.55594 | 0.42838 | 0.46338 | 6 | 2.93229 | 0.55262 | 0.38668 | 0.441151 | Not Significant | NS | | #N/A |
| 1928 | A0A8J2HNR2 | #N/A | #N/A | RABBIT | #N/A | -0.08023 | #N/A | #N/A | #N/A | #N/A | #N/A | 0.21368 | #N/A | #N/A | 0.293912 | #N/A | 0.27957 | #N/A | 0.79836 | #N/A | 0.53955 | 0 | 2.93229 | 0.55262 | #N/A | 0.552617 | Not Significant | NS | | #N/A |
| 426 | G1SIT3 | WD repeat domain 47 OS=Oryctolagus cuniculus O | WDR47 | RABBIT | 0.47037 | -0.15643 | -0.63071 | 0.55079 | -0.2796 | 1.28248 | -0.39137 | 0.20185 | 0.78162 | -0.45885 | 0.292264 | 0.72126 | 0.68994 | 0.49129 | 0.50462 | 0.41059 | 0.44574 | 8 | 2.93229 | 0.55262 | 0.41049 | 0.448613 | Not Significant | NS | | #N/A |
| 952 | G1U9D3 | Vacuolar protein sorting-associated protein 28 hom | VPS28 | RABBIT | 0.25447 | -0.84226 | -0.79612 | 0.205 | -0.25619 | -1.39597 | -0.5705 | 0.5123 | 1.05695 | 0.40714 | 0.289004 | 0.5825 | 0.59063 | 0.57628 | 0.56678 | 0.44179 | 0.46785 | 8 | 2.93229 | 0.55262 | 0.61541 | 0.598566 | Not Significant | NS | | #N/A |
| 2592 | G1U950 | #N/A | SLC48A1 | RABBIT | #N/A | #N/A | #N/A | 0.29705 | -0.93709 | #N/A | -0.2232 | 0.15883 | #N/A | #N/A | 0.287833 | 0.44559 | 0.40798 | 0.69948 | 0.70039 | 0.48373 | 0.51428 | 2 | 2.93229 | 0.55262 | 0.41726 | 0.497732 | Not Significant | NS | | #N/A |
| 1738 | G1SEC9 | #N/A | GNB4 | RABBIT | #N/A | -1.32078 | 1.50048 | -0.08058 | 0.00955 | 1.94802 | -0.50018 | 0.82692 | -1.01523 | #N/A | 0.287711 | 0.32573 | 0.36694 | 0.75569 | 0.72221 | 0.50097 | 0.52049 | 6 | 2.93229 | 0.55262 | 1.5604 | 1.229565 | Not Significant | NS | | #N/A |
| 1566 | G1T1E6 | Secernin 3 OS=Oryctolagus cuniculus OX=9986 GN | SCRN3 | RABBIT | -0.23879 | 0.44828 | #N/A | #N/A | -0.37663 | #N/A | 0.78909 | 0.42149 | -0.51717 | #N/A | 0.286851 | 0.61672 | 0.54146 | 0.57079 | 0.60515 | 0.43989 | 0.47901 | 4 | 2.93229 | 0.55262 | 0.32451 | 0.420995 | Not Significant | NS | | #N/A |
| 2096 | G1T461 | #N/A | CZIB | RABBIT | #N/A | 0.19782 | #N/A | #N/A | #N/A | #N/A | #N/A | #N/A | -1.07036 | 2.03582 | 0.284909 | 0.10591 | 0.18171 | 0.93282 | 0.86479 | 0.548 | 0.55783 | 1 | 2.93229 | 0.55262 | 4.82419 | 1.638898 | Not Significant | NS | | #N/A |
| 1751 | G1T2T9 | #N/A | RAB5A | RABBIT | #N/A | #N/A | 0.17065 | -0.37502 | #N/A | 1.27594 | -0.88543 | -0.54894 | 0.5893 | 0.47452 | 0.28326 | 0.4186 | 0.43115 | 0.69288 | 0.67784 | 0.48221 | 0.50607 | 5 | 2.93229 | 0.55262 | 0.65414 | 0.616612 | Not Significant | NS | | #N/A |
| 350 | A0A5F9DHY5 | Protein transport protein Sec31A OS=Oryctolagus c | SEC31A | RABBIT | 0.45974 | -1.13196 | -0.3577 | 0.78717 | -0.21408 | -0.83878 | 1.35402 | 0.13633 | -0.21837 | 0.52051 | 0.282106 | 0.56782 | 0.57595 | 0.58573 | 0.57631 | 0.44576 | 0.47008 | 8 | 2.93229 | 0.55262 | 0.61707 | 0.599785 | Not Significant | NS | | #N/A |

| | | | | | | | | | | | | | | | | | | | | | | | | | | | | | |
|---|---|---|---|---|---|---|---|---|---|---|---|---|---|---|---|---|---|---|---|---|---|---|---|---|---|---|---|---|---|
| 2383 | B8K131 | #N/A | HBZ_1 | RABBIT | #N/A | #N/A | 0.17065 | #N/A | #N/A | #N/A | -0.11603 | #N/A | #N/A | 1.01467 | 0.278669 | 0.28459 | 0.30015 | 0.82349 | 0.77926 | 0.51879 | 0.53632 | 1 | 2.93229 | 0.55262 | 0.63924 | 0.574644 | Not Significant | NS | #N/A |
| 1393 | A0A5F9CPQ1 | RUN and FYVE domain containing 2 OS=Oryctolagu | RUFY2 | RABBIT | -0.62166 | -0.02356 | 0.01559 | 0.35855 | 0.14168 | #N/A | #N/A | #N/A | #N/A | 0.25096 | 0.27684 | 0.69261 | 0.45348 | 0.52666 | 0.66406 | 0.42539 | 0.50024 | 4 | 2.93229 | 0.55262 | 0.13313 | 0.310572 | Not Significant | NS | #N/A |
| 2151 | A0A5F9CSC9 | #N/A | #N/A | RABBIT | #N/A | #N/A | 0.10782 | -1.02087 | -0.6996 | 0.73216 | -3.75441 | -0.00153 | 0.543 | 1.17602 | 0.2766 | 0.22739 | 0.26486 | 0.82767 | 0.79713 | 0.5203 | 0.5392 | 6 | 2.93229 | 0.55262 | 2.77424 | 2.044926 | Not Significant | NS | #N/A |
| 2846 | A0A375YX11 | #N/A | #N/A | MT | #N/A | #N/A | #N/A | #N/A | -0.17631 | #N/A | #N/A | 0.09856 | #N/A | #N/A | 0.274865 | #N/A | 0.26145 | #N/A | 0.81103 | #N/A | 0.54212 | 0 | 2.93229 | 0.55262 | #N/A | 0.552617 | Not Significant | NS | #N/A |
| 1203 | A0A5F9DNC5 | Large ribosomal subunit protein eL24 OS=Oryctolag | #N/A | RABBIT | #N/A | -1.90939 | 0.33065 | -0.01988 | -0.05115 | #N/A | #N/A | #N/A | -0.32296 | 0.04217 | 0.272046 | 0.3543 | 0.3789 | 0.741 | 0.7161 | 0.4961 | 0.51827 | 4 | 2.93229 | 0.55262 | 0.78608 | 0.687329 | Not Significant | NS | #N/A |
| 167 | A0A5F9DGX0 | ATP-dependent 6-phosphofructokinase OS=Oryctol | PFKM | RABBIT | -0.13424 | 0.21923 | 0.12464 | -0.07905 | -0.06323 | 0.44998 | 0.12962 | 0.30032 | 0.79176 | -0.24633 | 0.271601 | 1.47116 | 0.93583 | 0.17946 | 0.36957 | 0.25448 | 0.38394 | 8 | 2.93229 | 0.55262 | 0.08521 | 0.210578 | Not Significant | NS | #N/A |
| 1009 | A0A5F9D944 | Cell adhesion molecule 3 OS=Oryctolagus cuniculu | CADM3 | RABBIT | 0.46645 | 0.71152 | -0.75412 | -0.53944 | -0.29787 | -0.04355 | 0.45386 | -0.06437 | 0.40847 | #N/A | 0.271297 | 0.78149 | 0.68176 | 0.46012 | 0.51097 | 0.39639 | 0.44921 | 7 | 2.93229 | 0.55262 | 0.26781 | 0.351892 | Not Significant | NS | #N/A |
| 685 | G1SXV1 | Septin-4 OS=Oryctolagus cuniculus OX=9986 PE=3 | #N/A | RABBIT | 0.3849 | -0.30471 | -0.24738 | -0.65059 | 0.1285 | -0.02376 | 0.09941 | 0.61731 | 0.02376 | -0.08487 | 0.264226 | 1.20203 | 0.85885 | 0.26372 | 0.40886 | 0.3058 | 0.4061 | 8 | 2.93229 | 0.55262 | 0.1208 | 0.236622 | Not Significant | NS | #N/A |
| 2305 | G1SP95 | #N/A | SLC7A14 | RABBIT | #N/A | #N/A | 0.17065 | #N/A | #N/A | -0.38973 | #N/A | 1.25946 | #N/A | #N/A | 0.264214 | 0.18499 | 0.2478 | 0.88355 | 0.81669 | 0.53673 | 0.54344 | 1 | 2.93229 | 0.55262 | 1.35991 | 0.757915 | Not Significant | NS | #N/A |
| 2708 | A0A5F9DHE2 | #N/A | CCNY | RABBIT | #N/A | #N/A | #N/A | -0.22894 | #N/A | 0.40988 | #N/A | -0.47587 | 0.1713 | #N/A | 0.264038 | 0.49892 | 0.35551 | 0.6673 | 0.73691 | 0.47251 | 0.52494 | 2 | 2.93229 | 0.55262 | 0.21005 | 0.413709 | Not Significant | NS | #N/A |
| 2776 | A0A5F9CMJ0 | #N/A | GFAP | RABBIT | #N/A | #N/A | #N/A | #N/A | -0.03408 | #N/A | 0.75383 | -0.06883 | 0.00447 | #N/A | 0.263905 | 0.50199 | 0.35581 | 0.66549 | 0.73669 | 0.47235 | 0.52494 | 2 | 2.93229 | 0.55262 | 0.20728 | 0.412586 | Not Significant | NS | #N/A |
| 2314 | A0A5F9DNS5 | #N/A | VPS13D | RABBIT | #N/A | #N/A | -1.09049 | 0.61316 | #N/A | 1.10475 | -0.79344 | -0.06437 | -0.46408 | 0.32767 | 0.26077 | 0.36602 | 0.38326 | 0.72933 | 0.7116 | 0.49235 | 0.51654 | 5 | 2.93229 | 0.55262 | 0.72513 | 0.66136 | Not Significant | NS | #N/A |
| 1966 | G1SY53 | #N/A | RPL37A | RABBIT | #N/A | #N/A | 0.68896 | -1.22209 | #N/A | -0.34988 | 0 | 0.33204 | #N/A | #N/A | 0.260617 | 0.34464 | 0.36252 | 0.75311 | 0.72953 | 0.50058 | 0.5223 | 3 | 2.93229 | 0.55262 | 0.68621 | 0.620176 | Not Significant | NS | #N/A |
| 525 | A0A5F9DV00 | Platelet-activating factor acetylhydrolase IB subunit | PAFAH1B2 | RABBIT | -0.448 | 0.12904 | -0.31874 | 0.30706 | -0.13511 | 0.55458 | -0.58504 | 0.1265 | 0.67216 | 0.06878 | 0.260541 | 0.99534 | 0.7876 | 0.34872 | 0.44767 | 0.3464 | 0.42011 | 8 | 2.93229 | 0.55262 | 0.1713 | 0.273575 | Not Significant | NS | #N/A |
| 1827 | A0A5F9DK14 | #N/A | ST6GALNA | RABBIT | #N/A | -0.36002 | 1.4307 | -3.44615 | 0.54446 | -1.38153 | 1.93425 | -2.51567 | #N/A | 1.17178 | 0.259961 | 0.17426 | 0.20645 | 0.86739 | 0.84107 | 0.532 | 0.55124 | 6 | 2.93229 | 0.55262 | 4.45098 | 3.171229 | Not Significant | NS | #N/A |
| 1080 | G1TBI9 | Solute carrier family 17 member 6 OS=Oryctolagus | SLC17A6 | RABBIT | -2.55119 | #N/A | 0.54217 | -0.29687 | 0.22585 | 0.69192 | -0.56523 | 0.68618 | -1.8561 | #N/A | 0.259202 | 0.27975 | 0.31729 | 0.78907 | 0.75832 | 0.51029 | 0.53209 | 6 | 2.93229 | 0.55262 | 1.71702 | 1.334771 | Not Significant | NS | #N/A |
| 127 | G1TVI6 | Amidohydrolase-related domain-containing protein | #N/A | RABBIT | -0.6235 | -0.66688 | 0.31661 | 0.60035 | -0.2307 | 0.41101 | -0.07191 | 0.44906 | 0.07191 | -0.17732 | 0.257376 | 0.91182 | 0.75054 | 0.38852 | 0.46877 | 0.36533 | 0.42912 | 8 | 2.93229 | 0.55262 | 0.19919 | 0.293985 | Not Significant | NS | #N/A |
| 775 | G1SLW8 | Eukaryotic translation initiation factor 3 subunit M C | EIF3M | RABBIT | -0.73488 | -0.45907 | -0.24819 | 0.24065 | 0.21453 | -0.21327 | 0.21327 | 0.49398 | 0.42722 | -0.62286 | 0.25706 | 0.90625 | 0.7478 | 0.39128 | 0.47036 | 0.36581 | 0.42992 | 8 | 2.93229 | 0.55262 | 0.20115 | 0.295418 | Not Significant | NS | #N/A |
| 948 | A0A5F9CRL5 | Glutathione S-transferase omega OS=Oryctolagus c | GSTO1 | RABBIT | -0.25052 | #N/A | 1.38252 | -0.06841 | -0.7317 | 1.10175 | #N/A | -0.0536 | -0.24407 | 0.55566 | 0.256963 | 0.46853 | 0.47492 | 0.65594 | 0.64624 | 0.4707 | 0.49509 | 6 | 2.93229 | 0.55262 | 0.60158 | 0.585506 | Not Significant | NS | #N/A |
| 2691 | P9WQA3 | #N/A | #N/A | MT | #N/A | #N/A | #N/A | -0.08108 | #N/A | 0.41416 | #N/A | -0.06437 | #N/A | #N/A | 0.255977 | 0.61769 | 0.31466 | 0.64774 | 0.76901 | 0.46795 | 0.53467 | 1 | 2.93229 | 0.55262 | 0.11449 | 0.441199 | Not Significant | NS | #N/A |
| 1244 | A0A5F9CBZ8 | Myotrophin OS=Oryctolagus cuniculus OX=9986 PE | #N/A | RABBIT | 0.17444 | -0.22148 | -0.24396 | 0.40199 | -0.24011 | 0.6229 | -0.12372 | -0.1102 | 0.04584 | 0.70833 | 0.254451 | 1.13069 | 0.81975 | 0.29094 | 0.42987 | 0.3201 | 0.41175 | 8 | 2.93229 | 0.55262 | 0.12661 | 0.240873 | Not Significant | NS | #N/A |
| 2515 | A0A5F9C969 | #N/A | #N/A | RABBIT | #N/A | #N/A | #N/A | -0.16424 | #N/A | -0.40372 | -0.06075 | 0.35942 | 0.06075 | 0.49514 | 0.254405 | 0.65422 | 0.41951 | 0.54868 | 0.68754 | 0.43203 | 0.50942 | 4 | 2.93229 | 0.55262 | 0.12602 | 0.306464 | Not Significant | NS | #N/A |
| 1711 | G1SVP7 | #N/A | GSTO1 | RABBIT | #N/A | -0.02824 | #N/A | #N/A | #N/A | #N/A | 0.22606 | #N/A | #N/A | #N/A | 0.254298 | #N/A | 0.24189 | #N/A | 0.82479 | #N/A | 0.54595 | 0 | 2.93229 | 0.55262 | #N/A | 0.552617 | Not Significant | NS | #N/A |
| 2153 | A0A5S8HLQ6 | #N/A | H1-4 | RABBIT | #N/A | #N/A | 0.17556 | #N/A | -0.59058 | #N/A | 0.15977 | -0.06928 | #N/A | #N/A | 0.252757 | 0.63217 | 0.403 | 0.59191 | 0.70382 | 0.44774 | 0.51495 | 2 | 2.93229 | 0.55262 | 0.15986 | 0.393358 | Not Significant | NS | #N/A |
| 1683 | A0A5F9C277 | #N/A | DNM1 | RABBIT | #N/A | -0.03143 | -1.26878 | #N/A | #N/A | 0.53292 | #N/A | 0.32817 | 0.22925 | -2.68059 | 0.252541 | 0.20932 | 0.25064 | 0.84443 | 0.80936 | 0.52496 | 0.54212 | 4 | 2.93229 | 0.55262 | 1.94078 | 1.353602 | Not Significant | NS | #N/A |
| 2027 | G1SFW9 | #N/A | ACIN1 | RABBIT | #N/A | -0.28116 | #N/A | #N/A | #N/A | #N/A | #N/A | -0.05817 | #N/A | 0 | 0.252069 | 5.00349 | 0.32045 | 0.12558 | 0.76493 | 0.21204 | 0.53348 | 1 | 2.93229 | 0.55262 | 0.00169 | 0.412514 | Not Significant | NS | #N/A |
| 1789 | P00169 | #N/A | CYB5A CYI | RABBIT | #N/A | 0.47691 | #N/A | -0.59188 | -0.83583 | -1.17609 | 0.57468 | -0.25616 | 0.1918 | 0.33946 | 0.251668 | 0.49698 | 0.48523 | 0.63688 | 0.63919 | 0.46416 | 0.49195 | 6 | 2.93229 | 0.55262 | 0.48081 | 0.50438 | Not Significant | NS | #N/A |
| 655 | A0A5F9CJM9 | MIF4G domain-containing protein OS=Oryctolagus ( | #N/A | RABBIT | 0.01166 | 0.73897 | -1.11499 | -0.31909 | #N/A | #N/A | #N/A | #N/A | 0.07996 | #N/A | 0.250821 | 0.2917 | 0.29656 | 0.78952 | 0.77691 | 0.51035 | 0.53611 | 3 | 2.93229 | 0.55262 | 0.59149 | 0.572273 | Not Significant | NS | #N/A |
| 243 | P23612 | Tryptophan--tRNA ligase, cytoplasmic OS=Oryctola | WARS1 W/ | RABBIT | 0.03667 | 0.17281 | -1.64431 | -0.25849 | -0.86499 | -2.41423 | 0.37486 | 0.02039 | 0.38279 | 0.3231 | 0.249042 | 0.39093 | 0.41724 | 0.70605 | 0.68458 | 0.4856 | 0.50899 | 8 | 2.93229 | 0.55262 | 1.01457 | 0.890662 | Not Significant | NS | #N/A |
| 1626 | G1SYT1 | Cadherin-5 OS=Oryctolagus cuniculus OX=9986 GI | CDH5 | RABBIT | -0.82178 | -0.32933 | #N/A | #N/A | 0.79407 | #N/A | #N/A | 0.25994 | #N/A | 0 | 0.248982 | 0.39846 | 0.38188 | 0.71696 | 0.71585 | 0.48961 | 0.51827 | 3 | 2.93229 | 0.55262 | 0.46854 | 0.510099 | Not Significant | NS | #N/A |
| 220 | G1T5Y1 | Mitogen-activated protein kinase 1 OS=Oryctolagus | MAPK1 | RABBIT | -0.12825 | -0.0398 | 0.19932 | 0.13079 | -0.33976 | 0.43089 | -0.06534 | 0.15355 | 0.57434 | -0.02867 | 0.248494 | 1.56935 | 0.89183 | 0.15521 | 0.39169 | 0.2359 | 0.396 | 8 | 2.93229 | 0.55262 | 0.06268 | 0.194093 | Not Significant | NS | #N/A |
| 255 | G1SVJ5 | serine--tRNA ligase OS=Oryctolagus cuniculus OX= | SARS1 | RABBIT | -0.06689 | -0.47427 | -0.2282 | 0.18841 | #N/A | 0.36017 | 0.06321 | -0.10074 | 0.19166 | 0 | 0.248097 | 1.63016 | 0.82818 | 0.14709 | 0.42702 | 0.22929 | 0.41011 | 7 | 2.93229 | 0.55262 | 0.05147 | 0.199424 | Not Significant | NS | #N/A |
| 602 | A0A5F9DKX9 | Microtubule-associated protein RP/EB family memb | MAPRE2 | RABBIT | -0.59212 | 0.19782 | -0.03261 | 0.69277 | -0.68291 | 0.66011 | #N/A | -1.25433 | 0.4688 | 0.78121 | 0.24736 | 0.48574 | 0.48871 | 0.64198 | 0.63565 | 0.46655 | 0.49004 | 7 | 2.93229 | 0.55262 | 0.57628 | 0.569294 | Not Significant | NS | #N/A |
| 354 | A0A5F9C8D3 | Cofilin-1 OS=Oryctolagus cuniculus OX=9986 PE=3 | #N/A | RABBIT | -0.26941 | -0.52182 | 0.05861 | 0.20135 | -0.00954 | 0.77107 | -0.00384 | -0.05121 | 0.19775 | -0.21792 | 0.247331 | 1.15482 | 0.81166 | 0.28149 | 0.4343 | 0.3154 | 0.41414 | 8 | 2.93229 | 0.55262 | 0.11467 | 0.23214 | Not Significant | NS | #N/A |
| 382 | U3KNW1 | Acyl-CoA thioesterase 7 OS=Oryctolagus cuniculus | ACOT7 | RABBIT | -0.36875 | -0.39173 | 0.0042 | 0.10881 | 0.26119 | 0.53123 | -0.27629 | -0.2402 | 0.54419 | 0.28354 | 0.24575 | 1.10429 | 0.7951 | 0.30157 | 0.44347 | 0.32467 | 0.41812 | 8 | 2.93229 | 0.55262 | 0.12381 | 0.238827 | Not Significant | NS | #N/A |
| 1148 | G1TUP1 | Programmed cell death 6 OS=Oryctolagus cuniculu | PDCD6 | RABBIT | -0.31276 | 0.15476 | -0.37484 | -0.01458 | -0.1156 | -1.13466 | 0.38827 | 0.0821 | 0.26433 | 0.96279 | 0.24517 | 0.68442 | 0.6264 | 0.51305 | 0.54391 | 0.42096 | 0.46219 | 8 | 2.93229 | 0.55262 | 0.3208 | 0.382976 | Not Significant | NS | #N/A |
| 2260 | G1TRT5 | #N/A | #N/A | RABBIT | #N/A | #N/A | 0.05493 | -0.10388 | #N/A | -0.18554 | #N/A | 0.4355 | 0.04625 | 0.58439 | 0.244628 | 0.90873 | 0.52499 | 0.41489 | 0.61596 | 0.37703 | 0.48277 | 4 | 2.93229 | 0.55262 | 0.09662 | 0.289503 | Not Significant | NS | #N/A |
| 2338 | G1T359 | #N/A | NDUFS1 | RABBIT | #N/A | #N/A | #N/A | -0.20032 | #N/A | -0.55476 | -1.95888 | 2.09306 | 0.14268 | 0.48469 | 0.241681 | 0.14898 | 0.18019 | 0.88878 | 0.86216 | 0.53768 | 0.55718 | 4 | 2.93229 | 0.55262 | 2.19315 | 1.499221 | Not Significant | NS | #N/A |
| 1598 | G1TDD4 | Hexosyltransferase OS=Oryctolagus cuniculus OX= | B3GNT2 | RABBIT | -0.39747 | -0.17027 | 0.30969 | 0.19231 | #N/A | #N/A | 0.99329 | -0.2034 | 0.36978 | -0.26022 | 0.241297 | 0.71968 | 0.59185 | 0.49878 | 0.56862 | 0.41338 | 0.46793 | 6 | 2.93229 | 0.55262 | 0.22483 | 0.332435 | Not Significant | NS | #N/A |
| 1817 | G1TJR3 | #N/A | #N/A | RABBIT | #N/A | -0.78844 | 0.34792 | -0.53388 | 0.53869 | #N/A | -0.17727 | 0.63736 | -0.3021 | 0.36877 | 0.240618 | 0.61019 | 0.54467 | 0.56412 | 0.59931 | 0.4379 | 0.47593 | 6 | 2.93229 | 0.55262 | 0.31099 | 0.390314 | Not Significant | NS | #N/A |
| 710 | G1T1W6 | Adenosylhomocysteinase like 2 OS=Oryctolagus cu | AHCYL2 | RABBIT | -0.02693 | #N/A | -0.81524 | 2.75236 | #N/A | 0.59629 | #N/A | 3.14875 | -0.23772 | 0 | 0.2401 | 0.18604 | 0.22207 | 0.85972 | 0.82987 | 0.52967 | 0.54852 | 5 | 2.93229 | 0.55262 | 2.85518 | 2.004006 | Not Significant | NS | #N/A |
| 2378 | G1TY50 | #N/A | TAMALIN | RABBIT | #N/A | #N/A | 0.0514 | #N/A | #N/A | 1.30695 | -0.48893 | 0.05488 | #N/A | #N/A | 0.239567 | 0.22529 | 0.25301 | 0.84268 | 0.81047 | 0.52455 | 0.54212 | 2 | 2.93229 | 0.55262 | 0.8481 | 0.672432 | Not Significant | NS | #N/A |
| 1365 | A0A5F9C145 | Alpha-mannosidase OS=Oryctolagus cuniculus OX= | MAN2A1 | RABBIT | -0.73745 | -0.02714 | #N/A | 0.72212 | #N/A | #N/A | 0.22496 | #N/A | #N/A | #N/A | 0.239111 | 0.28372 | 0.28062 | 0.8033 | 0.79039 | 0.51441 | 0.53883 | 2 | 2.93229 | 0.55262 | 0.53271 | 0.544544 | Not Significant | NS | #N/A |
| 637 | G1SPK4 | Large ribosomal subunit protein uL10 OS=Oryctolag | RPLP0 | RABBIT | -1.45551 | -0.48548 | 0.34352 | -0.03243 | 0.34911 | -0.62231 | 0.56567 | 0.11254 | -0.11665 | -0.02521 | 0.238966 | 0.61703 | 0.58121 | 0.55436 | 0.57289 | 0.4344 | 0.46967 | 8 | 2.93229 | 0.55262 | 0.37497 | 0.422617 | Not Significant | NS | #N/A |
| 1651 | G1SI90 | ABC-type glutathione-S-conjugate transporter OS=( | ABCC3 | RABBIT | 0.53408 | #N/A | #N/A | #N/A | -1.14049 | #N/A | #N/A | -0.06437 | #N/A | #N/A | 0.23884 | 0.16469 | 0.22243 | 0.89609 | 0.83505 | 0.53964 | 0.54964 | 1 | 2.93229 | 0.55262 | 1.4021 | 0.768644 | Not Significant | NS | #N/A |
| 972 | A0A5F9DH17 | Septin-type G domain-containing protein OS=Oryct | #N/A | RABBIT | -0.12429 | -0.48545 | -0.01168 | -0.00752 | 0.35397 | -0.53236 | 0.61521 | 0.48453 | 0.40172 | -0.05012 | 0.238788 | 0.95396 | 0.73642 | 0.36803 | 0.47698 | 0.35421 | 0.43293 | 8 | 2.93229 | 0.55262 | 0.15664 | 0.26285 | Not Significant | NS | #N/A |
| 2622 | A0A5F9CV57 | #N/A | CSNK1A1 | RABBIT | #N/A | #N/A | #N/A | -0.14797 | #N/A | #N/A | #N/A | #N/A | #N/A | 0.09033 | 0.238295 | #N/A | 0.22667 | #N/A | 0.83556 | #N/A | 0.54964 | 0 | 2.93229 | 0.55262 | #N/A | 0.552617 | Not Significant | NS | #N/A |
| 2196 | A0A5F9DGW6 | #N/A | CUL4B | RABBIT | #N/A | #N/A | -0.48455 | #N/A | 0.01231 | #N/A | 0 | #N/A | #N/A | #N/A | 0.236122 | 0.54874 | 0.28951 | 0.6805 | 0.78682 | 0.47768 | 0.53841 | 1 | 2.93229 | 0.55262 | 0.12344 | 0.443474 | Not Significant | NS | #N/A |
| 2626 | G1T2Y0 | #N/A | GRAP2 | RABBIT | #N/A | #N/A | #N/A | -0.14627 | #N/A | #N/A | #N/A | #N/A | 0.08863 | #N/A | 0.234902 | #N/A | 0.22344 | #N/A | 0.83785 | #N/A | 0.55015 | 0 | 2.93229 | 0.55262 | #N/A | 0.552617 | Not Significant | NS | #N/A |
| 2524 | G1TPS1 | #N/A | #N/A | RABBIT | #N/A | #N/A | #N/A | -0.13083 | #N/A | 1.34451 | -0.39117 | 0.00883 | -0.7207 | 0.27705 | 0.234535 | 0.27065 | 0.2776 | 0.80006 | 0.78942 | 0.51361 | 0.53858 | 4 | 2.93229 | 0.55262 | 0.6258 | 0.594843 | Not Significant | NS | #N/A |
| 454 | G1TEK2 | non-specific serine/threonine protein kinase OS=Or | DCLK1 | RABBIT | -0.11099 | #N/A | #N/A | #N/A | #N/A | #N/A | 0.12265 | #N/A | #N/A | #N/A | 0.233638 | #N/A | 0.22224 | #N/A | 0.8387 | #N/A | 0.55021 | 0 | 2.93229 | 0.55262 | #N/A | 0.552617 | Not Significant | NS | #N/A |
| 2628 | A0A5F9DHD3 | #N/A | PRORP | RABBIT | #N/A | #N/A | #N/A | -0.05764 | #N/A | #N/A | #N/A | #N/A | 0.35131 | -0.0013 | 0.232643 | 0.76183 | 0.29039 | 0.58554 | 0.78619 | 0.44576 | 0.53841 | 1 | 2.93229 | 0.55262 | 0.06217 | 0.427893 | Not Significant | NS | #N/A |
| 2379 | G1U3B2 | #N/A | NUP50 | RABBIT | #N/A | #N/A | 0.16486 | -0.37864 | #N/A | -0.36308 | #N/A | 0.4363 | 0.00579 | 0.40835 | 0.228731 | 0.69523 | 0.46908 | 0.52518 | 0.65341 | 0.42484 | 0.49788 | 4 | 2.93229 | 0.55262 | 0.14432 | 0.317025 | Not Significant | NS | #N/A |
| 2428 | A0A5F9CIX8 | #N/A | PNPLA1 | RABBIT | #N/A | #N/A | -0.02643 | #N/A | #N/A | #N/A | 0.19708 | #N/A | #N/A | #N/A | 0.223515 | #N/A | 0.21261 | #N/A | 0.84555 | #N/A | 0.55249 | 0 | 2.93229 | 0.55262 | #N/A | 0.552617 | Not Significant | NS | #N/A |
| 2407 | A0A5F9DA28 | #N/A | FXR1 | RABBIT | #N/A | #N/A | -1.06715 | 0.55667 | #N/A | 0 | -1.4353 | -1.18464 | 1.66825 | 0.78916 | 0.222735 | 0.20811 | 0.23948 | 0.84336 | 0.81681 | 0.52474 | 0.54344 | 5 | 2.93229 | 0.55262 | 1.63648 | 1.235813 | Not Significant | NS | #N/A |
| 1759 | A0A5F9CTY7 | #N/A | RABEP1 | RABBIT | #N/A | -1.03056 | #N/A | 0.82147 | -0.12324 | #N/A | 0.10986 | #N/A | #N/A | #N/A | 0.220637 | 0.20633 | 0.23235 | 0.85563 | 0.8256 | 0.52893 | 0.54611 | 2 | 2.93229 | 0.55262 | 0.85762 | 0.676294 | Not Significant | NS | #N/A |
| 2149 | G1TG89 | #N/A | RPS15A | RABBIT | #N/A | #N/A | 0.34347 | -0.12273 | -0.37875 | -0.00621 | #N/A | 0.31547 | 0 | 0.36114 | 0.220271 | 1.03827 | 0.57347 | 0.34672 | 0.58222 | 0.34582 | 0.47236 | 5 | 2.93229 | 0.55262 | 0.07716 | 0.252918 | Not Significant | NS | #N/A |
| 920 | G1U6X4 | Methionine aminopeptidase 2 OS=Oryctolagus cun | METAP2 | RABBIT | -0.01614 | 0.02736 | -0.02134 | 0.56771 | -0.49955 | 0.98462 | -0.66665 | 0.34386 | 0.46892 | 0.02781 | 0.220102 | 0.68632 | 0.60004 | 0.51191 | 0.56071 | 0.42051 | 0.46562 | 8 | 2.93229 | 0.55262 | 0.25712 | 0.336379 | Not Significant | NS | #N/A |
| 2854 | G1SUH4 | #N/A | MTSS1 | RABBIT | #N/A | #N/A | #N/A | #N/A | -0.11569 | #N/A | 0.10231 | #N/A | #N/A | #N/A | 0.217995 | #N/A | 0.20736 | #N/A | 0.84929 | #N/A | 0.55382 | 0 | 2.93229 | 0.55262 | #N/A | 0.552617 | Not Significant | NS | #N/A |
| 902 | G1TC03 | Tripartite motif containing 28 OS=Oryctolagus cunic | TRIM28 | RABBIT | -0.82925 | -0.56966 | 0.30186 | 0.49585 | -0.26186 | 0.21849 | -0.13121 | 0.07605 | -0.42864 | 0.47115 | 0.213781 | 0.72547 | 0.60997 | 0.48884 | 0.55435 | 0.40978 | 0.46296 | 8 | 2.93229 | 0.55262 | 0.21709 | 0.307085 | Not Significant | NS | #N/A |
| 662 | A0A5F9CM41 | Acidic leucine-rich nuclear phosphoprotein 32 fami | #N/A | RABBIT | 1.11013 | #N/A | -1.80454 | #N/A | 0.00375 | #N/A | -0.01714 | #N/A | #N/A | #N/A | 0.21308 | 0.12542 | 0.16801 | 0.91166 | 0.87325 | 0.54361 | 0.56055 | 2 | 2.93229 | 0.55262 | 2.16489 | 1.20638 | Not Significant | NS | #N/A |
| 640 | A0A5F9D7T8 | Prostaglandin E synthase 3 OS=Oryctolagus cunicu | #N/A | RABBIT | -0.59991 | -0.81605 | 0.18531 | -0.0806 | 0.07553 | 0.79202 | 0.04613 | -0.07903 | 0.07248 | -1.00514 | 0.212436 | 0.6117 | 0.55305 | 0.55772 | 0.59136 | 0.43586 | 0.47335 | 8 | 2.93229 | 0.55262 | 0.30152 | 0.368872 | Not Significant | NS | #N/A |
| 1846 | G1T7G1 | #N/A | PODXL2 | RABBIT | #N/A | 0.19782 | #N/A | #N/A | #N/A | #N/A | 1.22494 | 1.22692 | -0.63953 | -0.1775 | 0.210888 | 0.19601 | 0.21905 | 0.85713 | 0.83395 | 0.52914 | 0.54958 | 3 | 2.93229 | 0.55262 | 0.92605 | 0.741463 | Not Significant | NS | #N/A |
| 1223 | G1T5H8 | Eukaryotic translation initiation factor 4B OS=Oryct | EIF4B | RABBIT | 0.17213 | #N/A | #N/A | -0.41626 | -0.53898 | -0.99913 | 0.3969 | 0.04379 | -0.10816 | 0.40946 | 0.209609 | 0.55359 | 0.47706 | 0.59986 | 0.64478 | 0.45128 | 0.49451 | 6 | 2.93229 | 0.55262 | 0.2688 | 0.361974 | Not Significant | NS | #N/A |
| 1172 | A0A5F9CXQ6 | Golgin A3 OS=Oryctolagus cuniculus OX=9986 GN= | GOLGA3 | RABBIT | -0.54005 | -2.1689 | 0.26819 | -0.15518 | 1.81564 | -2.95415 | 2.26307 | #N/A | 0.85097 | #N/A | 0.209358 | 0.14696 | 0.17327 | 0.88798 | 0.8663 | 0.53768 | 0.55818 | 6 | 2.93229 | 0.55262 | 3.8051 | 2.737379 | Not Significant | NS | #N/A |
| 1952 | A0A5F9C3X1 | #N/A | PYM1 | RABBIT | #N/A | #N/A | #N/A | -0.05764 | #N/A | #N/A | -0.73368 | -0.18002 | 0.81889 | 0.70162 | 0.209341 | 0.25313 | 0.25251 | 0.81652 | 0.80917 | 0.51745 | 0.54212 | 3 | 2.93229 | 0.55262 | 0.54716 | 0.549858 | Not Significant | NS | #N/A |
| 1413 | G1TGQ1 | Developmentally regulated GTP binding protein 1 O! | DRG1 | RABBIT | #N/A | 0.00983 | -0.13635 | #N/A | 0.55862 | 0.94392 | -0.24647 | 0.71161 | #N/A | 0 | 0.208231 | 0.54963 | 0.45479 | 0.60622 | 0.66145 | 0.45341 | 0.4996 | 5 | 2.93229 | 0.55262 | 0.24605 | 0.359379 | Not Significant | NS | #N/A |
| 504 | G1T545 | Lactoylglutathione lyase OS=Oryctolagus cuniculus | #N/A | RABBIT | 0.03754 | 0.493 | -0.66705 | -0.08351 | -0.64011 | -0.45164 | -0.04132 | 0.01449 | 0.31068 | 0.34328 | 0.207126 | 0.79074 | 0.62594 | 0.4519 | 0.5442 | 0.39238 | 0.46219 | 8 | 2.93229 | 0.55262 | 0.17153 | 0.273748 | Not Significant | NS | #N/A |
| 473 | A0A5F9CEJ7 | oxoglutarate dehydrogenase (succinyl-transferring) | OGDHL | RABBIT | 0.48998 | 0.04026 | 0.17065 | #N/A | #N/A | #N/A | -0.11735 | #N/A | 0.99823 | #N/A | 0.20681 | 0.45947 | 0.35997 | 0.67714 | 0.73134 | 0.47693 | 0.52334 | 3 | 2.93229 | 0.55262 | 0.24311 | 0.396098 | Not Significant | NS | #N/A |
| 1431 | G1SJF1 | WD repeat-containing protein 26 OS=Oryctolagus c | WDR26 | RABBIT | #N/A | -2.81379 | 0.17065 | 0.07394 | 0.57592 | -2.93034 | -0.02742 | -0.76495 | 1.2033 | 1.05828 | 0.206096 | 0.18846 | 0.2153 | 0.85587 | 0.83389 | 0.52893 | 0.54958 | 7 | 2.93229 | 0.55262 | 2.65773 | 2.036241 | Not Significant | NS | #N/A |
| 788 | O46651 | CD166 antigen (Fragment) OS=Oryctolagus cunicul | ALCAM | RABBIT | 0.25988 | -0.0504 | #N/A | 0.51898 | #N/A | 2.75961 | -0.64587 | #N/A | #N/A | -0.76752 | 0.205919 | 0.17636 | 0.21211 | 0.86858 | 0.83812 | 0.53229 | 0.55015 | 4 | 2.93229 | 0.55262 | 2.04502 | 1.413751 | Not Significant | NS | #N/A |
| 1489 | G1SYN3 | Leucine zipper transcription factor-like protein 1 OS | LZTFL1 | RABBIT | #N/A | 0.63305 | #N/A | -0.34618 | #N/A | 1.39084 | -0.08228 | -0.44836 | 0.88464 | 0 | 0.205536 | 0.32855 | 0.32926 | 0.75581 | 0.75049 | 0.50097 | 0.52952 | 5 | 2.93229 | 0.55262 | 0.55907 | 0.556687 | Not Significant | NS | #N/A |
| 1299 | G1SHM1 | Chromobox 3 OS=Oryctolagus cuniculus OX=9986 | #N/A | RABBIT | #N/A | -1.3005 | #N/A | 0.91759 | #N/A | -0.47225 | 0 | -0.38853 | 0.12881 | 0.8019 | 0.205441 | 0.2938 | 0.30584 | 0.78072 | 0.7676 | 0.50758 | 0.53416 | 5 | 2.93229 | 0.55262 | 0.69852 | 0.644584 | Not Significant | NS | #N/A |
| 1030 | G1SHQ9 | Sugar phosphate phosphatase OS=Oryctolagus cur | ARMT1 | RABBIT | 0.01166 | 0.13523 | -0.47268 | 1.00348 | #N/A | 1.23967 | -0.26046 | -1.52897 | 1.84636 | 0.5688 | 0.203657 | 0.282 | 0.30668 | 0.7861 | 0.76542 | 0.50949 | 0.53348 | 7 | 2.93229 | 0.55262 | 1.159 | 0.979981 | Not Significant | NS | #N/A |
| 2678 | A0A5F9DBH6 | #N/A | #N/A | RABBIT | #N/A | #N/A | #N/A | -0.10116 | #N/A | 0.29197 | #N/A | #N/A | 0.00037 | -0.00037 | 0.19848 | 1.01968 | 0.29476 | 0.41515 | 0.78018 | 0.37703 | 0.53656 | 2 | 2.93229 | 0.55262 | 0.02842 | 0.340058 | Not Significant | NS | #N/A |
| 733 | G1SS85 | Glyoxylate and hydroxypyruvate reductase OS=Oryc | GRHPR | RABBIT | -0.07525 | 0.65608 | -0.10591 | 0.43911 | -1.45901 | 1.01716 | -0.43059 | -0.52697 | 0.29969 | 0.08692 | 0.198239 | 0.4293 | 0.42721 | 0.67903 | 0.67752 | 0.47768 | 0.50607 | 8 | 2.93229 | 0.55262 | 0.53309 | 0.538327 | Not Significant | NS | #N/A |
| 2701 | G1SV55 | #N/A | REEP2 | RABBIT | #N/A | #N/A | #N/A | -0.05764 | #N/A | -0.08536 | #N/A | 0.0728 | -0.80705 | 1.37993 | 0.19772 | 0.19413 | 0.21246 | 0.85848 | 0.83886 | 0.52942 | 0.55021 | 3 | 2.93229 | 0.55262 | 0.82988 | 0.69283 | Not Significant | NS | #N/A |
| 599 | G1SLA4 | Protein lin-7 homolog OS=Oryctolagus cuniculus O | LIN7A | RABBIT | -0.27055 | 0.13348 | -0.11993 | 0.00782 | -0.28962 | 0.20525 | -0.10714 | 0.18256 | 0.10098 | 0.06434 | 0.196955 | 2.00616 | 0.76466 | 0.07975 | 0.46066 | 0.17277 | 0.42527 | 8 | 2.93229 | 0.55262 | 0.0241 | 0.165857 | Not Significant | NS | #N/A |
| 744 | A0A5F9D1T0 | AP-2 complex subunit sigma OS=Oryctolagus cunic | #N/A | RABBIT | -0.0084 | -0.00093 | -0.02649 | -0.75206 | 0.57765 | -0.39173 | 0.02006 | 0.44628 | 0.50737 | 0.18982 | 0.196406 | 0.73747 | 0.589 | 0.4819 | 0.56783 | 0.40669 | 0.46793 | 8 | 2.93229 | 0.55262 | 0.17732 | 0.277982 | Not Significant | NS | #N/A |
| 2487 | A0A5F9DTP4 | #N/A | PHYHIP | RABBIT | #N/A | #N/A | #N/A | -0.14702 | #N/A | 0.30752 | #N/A | -0.06437 | -0.27455 | 0.22751 | 0.196051 | 0.65315 | 0.31514 | 0.56025 | 0.76344 | 0.4366 | 0.53345 | 3 | 2.93229 | 0.55262 | 0.07208 | 0.309605 | Not Significant | NS | #N/A |
| 2425 | A0A5F9DU91 | #N/A | ULK2 | RABBIT | #N/A | #N/A | -0.55448 | 0.04856 | #N/A | 0.1975 | #N/A | -0.06437 | -0.30468 | #N/A | 0.19578 | 0.66933 | 0.37616 | 0.55119 | 0.71989 | 0.43284 | 0.51955 | 3 | 2.93229 | 0.55262 | 0.10267 | 0.325074 | Not Significant | NS | #N/A |
| 857 | G1SKT8 | Transgelin-3 OS=Oryctolagus cuniculus OX=9986 G | TAGLN3 | RABBIT | 0.06064 | 1.66834 | -1.96119 | -0.08424 | -1.08798 | 0.59799 | -0.34005 | 0.25453 | -0.96962 | 0.0266 | 0.194777 | 0.29245 | 0.31437 | 0.77738 | 0.75916 | 0.507 | 0.53222 | 8 | 2.93229 | 0.55262 | 1.10893 | 0.959712 | Not Significant | NS | #N/A |
| 1729 | A0A5F9DSZ1 | #N/A | PPFIA2 | RABBIT | #N/A | -0.63913 | #N/A | -0.04755 | 0.06052 | #N/A | -0.01009 | -0.08587 | 0.05009 | #N/A | 0.19343 | 0.87532 | 0.45092 | 0.43081 | 0.66581 | 0.38224 | 0.5009 | 4 | 2.93229 | 0.55262 | 0.07325 | 0.276017 | Not Significant | NS | #N/A |
| 2066 | G1SHJ5 | #N/A | RBM15 | RABBIT | #N/A | #N/A | #N/A | -0.13683 | #N/A | #N/A | #N/A | -0.18381 | 0.07919 | 0.27107 | 0.192311 | 0.7293 | 0.28164 | 0.54166 | 0.78965 | 0.43021 | 0.53858 | 2 | 2.93229 | 0.55262 | 0.05215 | 0.349682 | Not Significant | NS | #N/A |
| 1238 | P19943 | Large ribosomal subunit protein P2 (Fragment) OS= | RPLP2 | RABBIT | -1.70471 | #N/A | 0.27995 | -0.26543 | 0.62478 | -1.31873 | 0.96824 | 0.16721 | -0.21173 | 0 | 0.18735 | 0.30474 | 0.3214 | 0.76943 | 0.75457 | 0.50494 | 0.53124 | 7 | 2.93229 | 0.55262 | 0.83994 | 0.755114 | Not Significant | NS | #N/A |
| 1069 | G1T5V2 | PITH domain containing 1 OS=Oryctolagus cuniculu | PITHD1 | RABBIT | -0.41189 | 0.1864 | 0.76342 | -0.71788 | 0.49787 | #N/A | -0.79025 | -0.05295 | 1.59367 | #N/A | 0.186576 | 0.29469 | 0.30839 | 0.77816 | 0.76485 | 0.50703 | 0.53348 | 6 | 2.93229 | 0.55262 | 0.75159 | 0.686273 | Not Significant | NS | #N/A |
| 293 | B7NZL4 | Septin OS=Oryctolagus cuniculus OX=9986 GN=SE | SEPT8 | RABBIT | -0.0604 | -1.02297 | 0.40434 | -1.04308 | 0.64489 | -0.76364 | 0.21568 | 0.50739 | 0.07206 | -0.18443 | 0.184859 | 0.44788 | 0.43101 | 0.66612 | 0.67483 | 0.47235 | 0.50471 | 8 | 2.93229 | 0.55262 | 0.42588 | 0.459876 | Not Significant | NS | #N/A |
| 1860 | G1T2M0 | #N/A | KRAS | RABBIT | #N/A | -1.11535 | 0.17065 | 0.22475 | -0.25033 | -1.1468 | -0.15027 | 0.32572 | 0.17918 | 0.50269 | 0.184674 | 0.43048 | 0.40976 | 0.67979 | 0.69068 | 0.47768 | 0.51015 | 7 | 2.93229 | 0.55262 | 0.40897 | 0.451381 | Not Significant | NS | #N/A |
| 462 | A0A5F9DB45 | RAB6A, member RAS oncogene family OS=Oryctola | RAB6A | RABBIT | -4.32126 | -1.46211 | 1.12368 | 1.0081 | 1.17986 | -1.05042 | -0.95302 | 1.16792 | -2.40311 | 1.68872 | 0.184362 | 0.1398 | 0.15975 | 0.89227 | 0.87599 | 0.53823 | 0.56084 | 8 | 2.93229 | 0.55262 | 4.34775 | 3.329813 | Not Significant | NS | #N/A |
| 1260 | G1SPJ2 | ADP ribosylation factor like GTPase 6 interacting pro | ARL6IP1 | RABBIT | -0.69359 | -0.56782 | 0.56638 | -0.09223 | 0.02121 | 0.97538 | 1.86275 | 0.42868 | -1.26325 | -1.84837 | 0.184247 | 0.25275 | 0.27522 | 0.80684 | 0.78828 | 0.5152 | 0.53858 | 8 | 2.93229 | 0.55262 | 1.32854 | 1.120417 | Not Significant | NS | #N/A |
| 182 | G1SHX8 | Versican core protein OS=Oryctolagus cuniculus O) | VCAN | RABBIT | -1.17676 | #N/A | #N/A | -0.21968 | 1.00269 | 0.09628 | 0.60702 | -0.49997 | #N/A | 0 | 0.182077 | 0.30756 | 0.31222 | 0.77082 | 0.76292 | 0.50537 | 0.53345 | 5 | 2.93229 | 0.55262 | 0.60082 | 0.583002 | Not Significant | NS | #N/A |

| | | | | | | | | | | | | | | | | | | | | | | | | | | | | | |
|---|---|---|---|---|---|---|---|---|---|---|---|---|---|---|---|---|---|---|---|---|---|---|---|---|---|---|---|---|---|
| 1895 | A0A5F9CE02 | #N/A | ATP2B1 | RABBIT | #N/A | #N/A | 0.49783 | #N/A | -0.85883 | 0 | #N/A | #N/A | #N/A | #N/A | 0.180504 | 0.15363 | 0.18335 | 0.90295 | 0.86359 | 0.54177 | 0.55769 | 1 | 2.93229 | 0.55262 | 0.92026 | 0.646111 | Not Significant | NS | #N/A |
| 1499 | A0A5F9D315 | Elastin microfibril interfacer 1 OS=Oryctolagus cuni | EMILIN1 | RABBIT | -0.4537 | #N/A | 0.2988 | -0.92517 | 0.67449 | -1.49871 | 1.92993 | -0.06437 | 0.18886 | -0.16671 | 0.179196 | 0.25682 | 0.27766 | 0.8047 | 0.78697 | 0.51471 | 0.53841 | 7 | 2.93229 | 0.55262 | 1.08186 | 0.92561 | Not Significant | NS | #N/A |
| 1452 | G1TIU7 | Phosphofurin acidic cluster sorting protein 2 OS=Or | PACS2 | RABBIT | #N/A | -0.27336 | #N/A | -0.05764 | 0.17289 | 0.3699 | #N/A | -0.1172 | #N/A | #N/A | 0.179053 | 0.72722 | 0.35232 | 0.5197 | 0.73678 | 0.42287 | 0.52494 | 3 | 2.93229 | 0.55262 | 0.07275 | 0.309943 | Not Significant | NS | #N/A |
| 2240 | A0A5F9DGI5 | #N/A | SULT1C2 | RABBIT | #N/A | #N/A | 0.26157 | -1.14852 | #N/A | 0 | 0.17234 | 0.80252 | -1.92724 | -0.36974 | 0.179051 | 0.21047 | 0.23131 | 0.84161 | 0.82293 | 0.52455 | 0.54561 | 5 | 2.93229 | 0.55262 | 1.03386 | 0.855958 | Not Significant | NS | #N/A |
| 890 | A0A5F9CE76 | Tumor susceptibility 101 OS=Oryctolagus cuniculus | TSG101 | RABBIT | 0.2642 | -0.52469 | #N/A | 0.25397 | -0.21883 | -1.52529 | 0.91224 | 1.2687 | -0.04289 | 0 | 0.17889 | 0.31121 | 0.32324 | 0.7647 | 0.75322 | 0.50299 | 0.53058 | 7 | 2.93229 | 0.55262 | 0.73426 | 0.680634 | Not Significant | NS | #N/A |
| 412 | G1SY93 | Rac family small GTPase 1 OS=Oryctolagus cunicul | RAC1 | RABBIT | -0.40514 | -0.71849 | -0.03659 | 0.04979 | 0.02168 | -0.45614 | -0.03507 | 0.10552 | 0.12062 | 0.05806 | 0.176349 | 0.95825 | 0.6082 | 0.366 | 0.55548 | 0.35413 | 0.46338 | 8 | 2.93229 | 0.55262 | 0.08467 | 0.210184 | Not Significant | NS | #N/A |
| 2359 | G1TND8 | #N/A | PAQR8 | RABBIT | #N/A | #N/A | -0.04907 | #N/A | #N/A | #N/A | 0.0452 | -0.10956 | 0.43666 | #N/A | 0.173166 | 0.53269 | 0.24971 | 0.64751 | 0.81288 | 0.46795 | 0.54293 | 2 | 2.93229 | 0.55262 | 0.07926 | 0.360674 | Not Significant | NS | #N/A |
| 1298 | A0A5F9CQ32 | TATA element modulatory factor 1 OS=Oryctolagus | TMF1 | RABBIT | #N/A | 0.43467 | -0.61007 | #N/A | -0.1437 | #N/A | #N/A | 0.06596 | #N/A | #N/A | 0.172324 | 0.28514 | 0.22508 | 0.80235 | 0.83094 | 0.51411 | 0.54901 | 2 | 2.93229 | 0.55262 | 0.27392 | 0.439607 | Not Significant | NS | #N/A |
| 2111 | A0A5F9DAL1 | #N/A | #N/A | RABBIT | #N/A | -0.56951 | #N/A | -0.30091 | 0.22989 | #N/A | 0.32856 | -1.05946 | 0.59954 | #N/A | 0.169724 | 0.30042 | 0.29107 | 0.77883 | 0.77951 | 0.50703 | 0.53632 | 4 | 2.93229 | 0.55262 | 0.47877 | 0.510009 | Not Significant | NS | #N/A |
| 2438 | G1TB40 | #N/A | RPA2 | RABBIT | #N/A | #N/A | 0.17065 | #N/A | #N/A | #N/A | #N/A | 0.78905 | #N/A | -0.10902 | 0.169361 | 0.21776 | 0.19276 | 0.8635 | 0.85669 | 0.53073 | 0.55532 | 1 | 2.93229 | 0.55262 | 0.40327 | 0.514636 | Not Significant | NS | #N/A |
| 2308 | G1T4L9 | #N/A | MYEF2 | RABBIT | #N/A | #N/A | 0.13525 | -0.45997 | 0.33605 | -0.06594 | #N/A | 0.54749 | #N/A | 0.0354 | 0.168543 | 0.55217 | 0.36815 | 0.61023 | 0.72375 | 0.45404 | 0.52073 | 4 | 2.93229 | 0.55262 | 0.13975 | 0.314391 | Not Significant | NS | #N/A |
| 625 | G1SVI9 | Vesicle-associated membrane protein-associated p | VAPA | RABBIT | -0.95636 | -0.06494 | -0.40804 | -0.07045 | 0.09512 | -0.94006 | 0.01281 | 0.13562 | 0.16971 | 0.05899 | 0.168344 | 0.59972 | 0.49226 | 0.56529 | 0.63227 | 0.43824 | 0.4889 | 8 | 2.93229 | 0.55262 | 0.19699 | 0.292376 | Not Significant | NS | #N/A |
| 2640 | A0A5F9CDC8 | #N/A | SNX9 | RABBIT | #N/A | #N/A | #N/A | 0.11906 | -0.20847 | -0.1767 | 0.42367 | #N/A | #N/A | #N/A | 0.168188 | 0.49185 | 0.2743 | 0.67151 | 0.79497 | 0.47349 | 0.53895 | 2 | 2.93229 | 0.55262 | 0.11693 | 0.37595 | Not Significant | NS | #N/A |
| 1940 | G1SFD8 | #N/A | DDX23 | RABBIT | #N/A | -0.38661 | #N/A | 0.05045 | #N/A | 0.00669 | #N/A | #N/A | -0.00669 | #N/A | 0.168079 | 0.76877 | 0.28495 | 0.5224 | 0.78726 | 0.42389 | 0.53841 | 2 | 2.93229 | 0.55262 | 0.0478 | 0.347918 | Not Significant | NS | #N/A |
| 1682 | A0A5F9CC02 | #N/A | DCLK1 | RABBIT | #N/A | 1.0008 | -0.90478 | 0.07284 | -0.8188 | 0.06187 | #N/A | -0.51109 | 0.52921 | -0.06187 | 0.167016 | 0.33747 | 0.33059 | 0.74726 | 0.74858 | 0.49801 | 0.52913 | 6 | 2.93229 | 0.55262 | 0.48987 | 0.510468 | Not Significant | NS | #N/A |
| 2535 | G1SLF8 | #N/A | ECPAS | RABBIT | #N/A | #N/A | #N/A | 0.03229 | #N/A | 2.62046 | -1.64559 | -0.15429 | -0.10904 | 0.28195 | 0.16641 | 0.09861 | 0.11998 | 0.92619 | 0.9079 | 0.54606 | 0.5704 | 4 | 2.93229 | 0.55262 | 2.37314 | 1.603079 | Not Significant | NS | #N/A |
| 1893 | A0A5F9C3E4 | #N/A | GSPT2 | RABBIT | #N/A | 0.01651 | #N/A | #N/A | #N/A | 0.18131 | #N/A | #N/A | #N/A | #N/A | 0.164798 | #N/A | 0.15676 | #N/A | 0.88561 | #N/A | 0.56421 | 0 | 2.93229 | 0.55262 | #N/A | 0.552617 | Not Significant | NS | #N/A |
| 2304 | G1T8P3 | #N/A | SNRNP70 | RABBIT | #N/A | #N/A | -0.25662 | 0.23473 | #N/A | -0.01911 | 0 | 0.42774 | -0.57421 | 0.9311 | 0.164045 | 0.37274 | 0.31862 | 0.72462 | 0.75824 | 0.49085 | 0.53209 | 5 | 2.93229 | 0.55262 | 0.2767 | 0.378696 | Not Significant | NS | #N/A |
| 2217 | A0A5F9DTA5 | #N/A | #N/A | RABBIT | #N/A | #N/A | #N/A | -0.17532 | #N/A | 0.0385 | -0.68368 | -0.10287 | 0.5225 | 0.16256 | 0.162721 | 0.33679 | 0.25253 | 0.7532 | 0.80795 | 0.50058 | 0.54212 | 4 | 2.93229 | 0.55262 | 0.19453 | 0.345997 | Not Significant | NS | #N/A |
| 2112 | G1SWN3 | #N/A | TSKS | RABBIT | #N/A | #N/A | #N/A | -1.36919 | 1.17249 | -0.11943 | #N/A | 0.31101 | 0 | #N/A | 0.162204 | 0.16868 | 0.19453 | 0.87678 | 0.85225 | 0.53468 | 0.55412 | 3 | 2.93229 | 0.55262 | 1.10961 | 0.834291 | Not Significant | NS | #N/A |
| 2108 | G1SP11 | #N/A | PRKRA | RABBIT | #N/A | #N/A | -1.56074 | 1.17419 | #N/A | #N/A | #N/A | -0.48493 | #N/A | 0.42056 | 0.161091 | 0.11183 | 0.14893 | 0.92117 | 0.8875 | 0.54531 | 0.56462 | 2 | 2.93229 | 0.55262 | 2.07494 | 1.169904 | Not Significant | NS | #N/A |
| 457 | G1SCE4 | Clathrin light chain OS=Oryctolagus cuniculus OX= | CLTA | RABBIT | -0.50036 | -0.76351 | 0.81718 | -0.83994 | 0.63515 | -0.02031 | 0.35241 | 0.47317 | 0.02031 | -0.67619 | 0.160177 | 0.39274 | 0.37648 | 0.70477 | 0.71376 | 0.48517 | 0.5176 | 8 | 2.93229 | 0.55262 | 0.41585 | 0.452535 | Not Significant | NS | #N/A |
| 907 | G1SCV9 | L-aminoadipate-semialdehyde dehydrogenase-pho | AASDHPPT | RABBIT | -0.84744 | -0.90934 | 0.08775 | 0.31725 | 0.21124 | 0.24089 | -1.40293 | 0.01853 | 1.00735 | -0.20601 | 0.159673 | 0.33669 | 0.33746 | 0.74502 | 0.74216 | 0.49742 | 0.52755 | 8 | 2.93229 | 0.55262 | 0.56228 | 0.559686 | Not Significant | NS | #N/A |
| 2289 | G1SGH8 | #N/A | C12orf4 | RABBIT | #N/A | #N/A | 3.3509 | -1.22678 | -0.13025 | #N/A | 1.64742 | #N/A | 0 | #N/A | 0.159089 | 0.08443 | 0.11185 | 0.93803 | 0.91463 | 0.54911 | 0.57198 | 3 | 2.93229 | 0.55262 | 4.26076 | 2.427854 | Not Significant | NS | #N/A |
| 597 | G1TMD8 | Serine/threonine-protein phosphatase OS=Oryctola | PPP5C | RABBIT | 0.06686 | -1.3 | -0.37857 | 0.81514 | -0.06858 | -0.17915 | -0.93133 | 0.15374 | 0.48967 | 0.39626 | 0.158869 | 0.37056 | 0.36088 | 0.72058 | 0.72506 | 0.49014 | 0.52124 | 8 | 2.93229 | 0.55262 | 0.45952 | 0.484489 | Not Significant | NS | #N/A |
| 2063 | G1T1K0 | #N/A | MPP2 | RABBIT | #N/A | #N/A | -0.37707 | 0.17515 | -0.36407 | 1.6423 | -2.49781 | -0.29715 | 0.33573 | 0.6651 | 0.158299 | 0.16984 | 0.19192 | 0.87072 | 0.8521 | 0.53296 | 0.55412 | 6 | 2.93229 | 0.55262 | 1.62893 | 1.275601 | Not Significant | NS | #N/A |
| 197 | G1SQ02 | Peroxiredoxin-1 OS=Oryctolagus cuniculus OX=998 | #N/A | RABBIT | -0.17527 | 0.13475 | 0.19855 | -0.29255 | 0.13275 | -0.21523 | 0.43152 | 0.23109 | 0.36137 | -0.0279 | 0.156523 | 1.0032 | 0.56369 | 0.34514 | 0.58434 | 0.34542 | 0.47236 | 8 | 2.93229 | 0.55262 | 0.06086 | 0.192759 | Not Significant | NS | #N/A |
| 1884 | G1SN67 | #N/A | #N/A | RABBIT | #N/A | 0.19782 | #N/A | #N/A | #N/A | #N/A | 0.73273 | #N/A | -0.02631 | #N/A | 0.155388 | 0.23639 | 0.18212 | 0.85222 | 0.86449 | 0.52779 | 0.55783 | 1 | 2.93229 | 0.55262 | 0.28807 | 0.485342 | Not Significant | NS | #N/A |
| 1481 | G1TCS5 | Thioredoxin domain containing 11 OS=Oryctolagus | TXNDC11 | RABBIT | -0.48052 | #N/A | #N/A | #N/A | #N/A | #N/A | -2.04387 | #N/A | 0.57392 | 0.49218 | 0.1546 | 0.08996 | 0.12088 | 0.93652 | 0.90855 | 0.5491 | 0.57061 | 2 | 2.93229 | 0.55262 | 2.21517 | 1.226769 | Not Significant | NS | #N/A |
| 729 | G1SSP0 | Striatin OS=Oryctolagus cuniculus OX=9986 GN=ST | STRN | RABBIT | -0.25074 | -0.84108 | -0.16735 | 0.16615 | 0.74253 | 0.39064 | 0.30251 | -0.27081 | 0.20645 | -0.22503 | 0.15085 | 0.51279 | 0.43079 | 0.62196 | 0.67499 | 0.45769 | 0.50471 | 8 | 2.93229 | 0.55262 | 0.21635 | 0.306544 | Not Significant | NS | #N/A |
| 1373 | U3KNF3 | Pyridoxal dependent decarboxylase domain contair | PDXDC1 | RABBIT | #N/A | #N/A | #N/A | -0.08841 | #N/A | #N/A | 0.03077 | #N/A | 0.21935 | -0.06621 | 0.149714 | 0.8929 | 0.22332 | 0.46613 | 0.83224 | 0.39952 | 0.54924 | 2 | 2.93229 | 0.55262 | 0.02109 | 0.337085 | Not Significant | NS | #N/A |
| 2523 | A0A5F9DF93 | #N/A | #N/A | RABBIT | #N/A | #N/A | #N/A | -0.47002 | 0.61688 | -1.4061 | 2.07481 | #N/A | #N/A | 0 | 0.149473 | 0.10937 | 0.13806 | 0.91981 | 0.89476 | 0.54531 | 0.56755 | 3 | 2.93229 | 0.55262 | 2.24119 | 1.40654 | Not Significant | NS | #N/A |
| 1353 | G1STF9 | Eukaryotic translation initiation factor 3 subunit I OS | EIF3I EIF3S | RABBIT | #N/A | #N/A | #N/A | -0.25617 | #N/A | 0.85754 | 0.19853 | -1.27226 | -0.69012 | 0.36667 | 0.148243 | 0.15769 | 0.16674 | 0.88234 | 0.87233 | 0.53662 | 0.56055 | 4 | 2.93229 | 0.55262 | 0.73648 | 0.658706 | Not Significant | NS | #N/A |
| 1808 | G1T8E2 | #N/A | GSK3B | RABBIT | #N/A | -1.35499 | 0.17065 | 2.22399 | #N/A | #N/A | -0.76004 | 1.74922 | #N/A | #N/A | 0.14804 | 0.09066 | 0.11792 | 0.93348 | 0.91002 | 0.54802 | 0.57132 | 3 | 2.93229 | 0.55262 | 3.19971 | 1.891269 | Not Significant | NS | #N/A |
| 1809 | A0A5F9CS42 | #N/A | OSBPL1A | RABBIT | #N/A | -0.72849 | 1.03973 | 1.68003 | -2.87919 | 2.11946 | -3.14491 | -0.93321 | 1.58853 | 0 | 0.147955 | 0.10605 | 0.12308 | 0.91852 | 0.9045 | 0.54531 | 0.56976 | 7 | 2.93229 | 0.55262 | 4.32519 | 3.211423 | Not Significant | NS | #N/A |
| 1328 | Q6DQX5 | Coxsackie virus and adenovirus receptor (Fragment | #N/A | RABBIT | 0.49324 | -2.40702 | #N/A | #N/A | 0.35835 | #N/A | #N/A | #N/A | -0.37173 | #N/A | 0.146747 | 0.07764 | 0.10684 | 0.94518 | 0.91913 | 0.55171 | 0.57326 | 2 | 2.93229 | 0.55262 | 2.67949 | 1.415046 | Not Significant | NS | #N/A |
| 2504 | A0A5F9D7L1 | #N/A | CLASP1 | RABBIT | #N/A | #N/A | #N/A | 0.3553 | -0.47175 | 1.8168 | #N/A | -0.30461 | 0.24025 | -1.40181 | 0.145883 | 0.14077 | 0.16362 | 0.89485 | 0.8747 | 0.53912 | 0.56083 | 4 | 2.93229 | 0.55262 | 1.4319 | 1.059975 | Not Significant | NS | #N/A |
| 25 | A0A5F9DLC0 | Creatine kinase B-type OS=Oryctolagus cuniculus ( | #N/A | RABBIT | -0.5364 | 0.54148 | -0.16632 | -0.09634 | -0.21356 | 0.1099 | -0.17605 | -0.02567 | 0.14496 | 0.20491 | 0.145835 | 0.77266 | 0.49916 | 0.46194 | 0.62756 | 0.39732 | 0.48758 | 8 | 2.93229 | 0.55262 | 0.08906 | 0.213398 | Not Significant | NS | #N/A |
| 6 | A0A5F9DCD4 | Clathrin heavy chain OS=Oryctolagus cuniculus OX | CLTC | RABBIT | -0.54247 | -0.8099 | 0.71589 | -0.3903 | 0.81775 | -0.18611 | 0.45324 | 0.46925 | 0.18611 | -0.4081 | 0.144683 | 0.38104 | 0.35641 | 0.71309 | 0.72832 | 0.48883 | 0.5223 | 8 | 2.93229 | 0.55262 | 0.36044 | 0.411989 | Not Significant | NS | #N/A |
| 2152 | A0A5F9CH62 | #N/A | #N/A | RABBIT | #N/A | #N/A | -0.68823 | 0.00511 | #N/A | 0.31331 | -0.63674 | -0.85612 | 0.18921 | 0 | 0.143495 | 0.33424 | 0.28186 | 0.75176 | 0.78526 | 0.50033 | 0.53833 | 5 | 2.93229 | 0.55262 | 0.26331 | 0.370257 | Not Significant | NS | #N/A |
| 2490 | G1TLT8 | #N/A | RPSA | RABBIT | #N/A | #N/A | #N/A | 0.18037 | -0.32356 | -0.22574 | 0.50975 | -0.06437 | -0.28187 | 0.42125 | 0.1434 | 0.46716 | 0.31875 | 0.66003 | 0.75815 | 0.47104 | 0.53209 | 5 | 2.93229 | 0.55262 | 0.13461 | 0.28913 | Not Significant | NS | #N/A |
| 169 | G1SVH0 | Adenosylhomocysteinase OS=Oryctolagus cunicul | AHCY | RABBIT | -0.06752 | -0.2535 | -0.07929 | -0.07683 | 0.00581 | 0.11673 | 0.23481 | -0.53725 | 0.20111 | 0.22069 | 0.141481 | 0.92094 | 0.51132 | 0.38401 | 0.61929 | 0.36226 | 0.48406 | 8 | 2.93229 | 0.55262 | 0.059 | 0.191401 | Not Significant | NS | #N/A |
| 296 | A0A5F9C3B5 | Heterogeneous nuclear ribonucleoprotein K OS=Or | #N/A | RABBIT | -0.68848 | -0.08468 | 0.0527 | 0.49834 | 0.18458 | -0.07603 | 0.07603 | 0.34886 | -0.16974 | 0.48986 | 0.141304 | 0.60847 | 0.44965 | 0.55975 | 0.66175 | 0.4366 | 0.4996 | 8 | 2.93229 | 0.55262 | 0.13483 | 0.246887 | Not Significant | NS | #N/A |
| 2062 | G1U5A4 | #N/A | TRDN | RABBIT | #N/A | -0.15848 | #N/A | -0.41196 | 0.7539 | #N/A | #N/A | 0.39465 | 0 | #N/A | 0.136171 | 0.28362 | 0.2321 | 0.79515 | 0.82426 | 0.51196 | 0.5459 | 3 | 2.93229 | 0.55262 | 0.27662 | 0.413042 | Not Significant | NS | #N/A |
| 1114 | G1TN89 | Heparan sulfate proteoglycan 2 OS=Oryctolagus cu | HSPG2 | RABBIT | -0.07016 | #N/A | -0.04652 | #N/A | 2.28706 | 2.08394 | 3.67181 | -0.74855 | -0.79675 | 0.08182 | 0.134993 | 0.10385 | 0.12163 | 0.92067 | 0.90588 | 0.54531 | 0.56976 | 6 | 2.93229 | 0.55262 | 3.16828 | 2.309608 | Not Significant | NS | #N/A |
| 2866 | G1TE38 | #N/A | #N/A | RABBIT | #N/A | #N/A | #N/A | #N/A | -0.01338 | 0.91474 | #N/A | #N/A | -0.67591 | #N/A | 0.1328 | 0.0964 | 0.12658 | 0.93882 | 0.90548 | 0.54931 | 0.56976 | 1 | 2.93229 | 0.55262 | 1.26507 | 0.733798 | Not Significant | NS | #N/A |
| 2737 | G1SVU9 | #N/A | PTEN | RABBIT | #N/A | #N/A | #N/A | -0.03908 | #N/A | #N/A | #N/A | 0.37831 | -0.08112 | -0.01856 | 0.131955 | 0.45863 | 0.19215 | 0.69151 | 0.85529 | 0.48172 | 0.55504 | 2 | 2.93229 | 0.55262 | 0.06208 | 0.35371 | Not Significant | NS | #N/A |
| 553 | G1STI3 | KH RNA binding domain containing, signal transduc | KHDRBS1 | RABBIT | -0.80998 | -0.57196 | 0.47938 | 0.30816 | -0.03462 | -0.67978 | 0.02124 | 0.39111 | -0.19335 | 0.48989 | 0.131626 | 0.40402 | 0.35567 | 0.69678 | 0.72886 | 0.4833 | 0.5223 | 8 | 2.93229 | 0.55262 | 0.26534 | 0.342398 | Not Significant | NS | #N/A |
| 2531 | A0A5F9CMI3 | #N/A | #N/A | RABBIT | #N/A | #N/A | #N/A | 0.28999 | -0.01338 | 2.28865 | -0.188 | -1.79172 | 2.63531 | -1.59871 | 0.130801 | 0.08317 | 0.10026 | 0.93695 | 0.92263 | 0.5491 | 0.57345 | 5 | 2.93229 | 0.55262 | 3.53364 | 2.431659 | Not Significant | NS | #N/A |
| 1129 | A0A5F9C5I6 | V-type proton ATPase subunit C OS=Oryctolagus cu | ATP6V1C2 | RABBIT | -0.73633 | -0.28089 | 0.38426 | -0.12956 | 0.45504 | -0.43184 | 0.69687 | 0.14909 | -0.15668 | 0.07192 | 0.127365 | 0.43997 | 0.36673 | 0.6716 | 0.72082 | 0.47349 | 0.51993 | 8 | 2.93229 | 0.55262 | 0.20951 | 0.301536 | Not Significant | NS | #N/A |
| 2653 | A0A1E3TKK3 | #N/A | #N/A | MT | #N/A | #N/A | #N/A | -0.09162 | #N/A | #N/A | #N/A | #N/A | #N/A | 0.03398 | 0.125603 | #N/A | 0.11947 | #N/A | 0.91261 | #N/A | 0.57143 | 0 | 2.93229 | 0.55262 | #N/A | 0.552617 | Not Significant | NS | #N/A |
| 1930 | G1SQQ0 | #N/A | NINL | RABBIT | #N/A | -0.12598 | #N/A | -0.8352 | 1.17244 | #N/A | 0.64962 | 0.25102 | -0.31539 | #N/A | 0.124664 | 0.19147 | 0.197 | 0.85748 | 0.84948 | 0.52914 | 0.55382 | 4 | 2.93229 | 0.55262 | 0.63587 | 0.600657 | Not Significant | NS | #N/A |
| 2760 | G1SYR5 | #N/A | EIF2B2 | RABBIT | #N/A | #N/A | #N/A | -0.05764 | #N/A | #N/A | #N/A | #N/A | -0.30954 | 0.44314 | 0.12444 | 0.19091 | 0.14603 | 0.87991 | 0.89108 | 0.53586 | 0.56587 | 1 | 2.93229 | 0.55262 | 0.28326 | 0.484118 | Not Significant | NS | #N/A |
| 1996 | A0A5F9DIF6 | #N/A | #N/A | RABBIT | #N/A | #N/A | #N/A | 0.10374 | #N/A | -0.53114 | -0.16138 | 1.55887 | -0.50537 | 0.77657 | 0.123768 | 0.12368 | 0.13359 | 0.90753 | 0.89752 | 0.54318 | 0.56784 | 4 | 2.93229 | 0.55262 | 0.83447 | 0.71525 | Not Significant | NS | #N/A |
| 2239 | G1T5U2 | #N/A | #N/A | RABBIT | #N/A | #N/A | 0.1496 | #N/A | 0.00766 | 0.56412 | #N/A | -0.41675 | 0.68789 | -0.0281 | 0.123155 | 0.31541 | 0.24002 | 0.76822 | 0.81726 | 0.50485 | 0.54353 | 4 | 2.93229 | 0.55262 | 0.20328 | 0.351043 | Not Significant | NS | #N/A |
| 480 | G1SRT1 | Adaptor protein, phosphotyrosine interacting with P | APPL1 | RABBIT | 0.06905 | -0.85944 | -0.30456 | 0.46175 | 0.25533 | -0.05739 | -0.60608 | 0.07076 | 0.96253 | -0.13665 | 0.122207 | 0.35308 | 0.31879 | 0.73315 | 0.75589 | 0.49265 | 0.53149 | 8 | 2.93229 | 0.55262 | 0.29949 | 0.367384 | Not Significant | NS | #N/A |
| 221 | G1SYD6 | Lamin A/C OS=Oryctolagus cuniculus OX=9986 GN | LMNA | RABBIT | -0.80202 | -0.38933 | 0.67078 | -0.41646 | 0.50008 | #N/A | 0.28207 | 0.41467 | -0.56207 | 0 | 0.121058 | 0.32242 | 0.29124 | 0.75655 | 0.77686 | 0.50107 | 0.53611 | 7 | 2.93229 | 0.55262 | 0.31328 | 0.383942 | Not Significant | NS | #N/A |
| 2290 | G1TSG1 | #N/A | RPL22 | RABBIT | #N/A | #N/A | 0.17065 | -0.03607 | -0.36241 | #N/A | -0.46307 | 0.4021 | -0.14844 | 0.38831 | 0.120668 | 0.42672 | 0.29303 | 0.68733 | 0.77702 | 0.48039 | 0.53611 | 5 | 2.93229 | 0.55262 | 0.13708 | 0.290689 | Not Significant | NS | #N/A |
| 2285 | G1SWB7 | #N/A | CARMIL2 | RABBIT | #N/A | #N/A | -1.32189 | -0.05764 | 0.67582 | #N/A | 0.82842 | #N/A | #N/A | -1.06047 | 0.118544 | 0.11498 | 0.13552 | 0.91572 | 0.89668 | 0.54451 | 0.56784 | 3 | 2.93229 | 0.55262 | 1.27545 | 0.918156 | Not Significant | NS | #N/A |
| 822 | G1TE78 | Astrocytic phosphoprotein PEA-15 OS=Oryctolagus | PEA15 | RABBIT | -0.87575 | 1.60235 | -0.33419 | 0.17383 | -0.64712 | -0.42451 | 0.10325 | 0.19284 | -0.10325 | 0.74153 | 0.118149 | 0.24555 | 0.24705 | 0.81221 | 0.80945 | 0.51658 | 0.54212 | 8 | 2.93229 | 0.55262 | 0.5788 | 0.571777 | Not Significant | NS | #N/A |
| 209 | G1U7L4 | 78 kDa glucose-regulated protein OS=Oryctolagus ( | HSPA5 | RABBIT | -0.76055 | 0.34539 | 0.14988 | -0.03687 | -0.16264 | -1.31232 | 0.75921 | 0.68499 | 0.19304 | -0.20265 | 0.117411 | 0.27876 | 0.27 | 0.7875 | 0.79219 | 0.50995 | 0.53886 | 8 | 2.93229 | 0.55262 | 0.44349 | 0.472761 | Not Significant | NS | #N/A |
| 796 | G1T7R1 | Fatty acid-binding protein, heart OS=Oryctolagus cu | FABP3 | RABBIT | 0.18004 | 3.57714 | -1.4994 | #N/A | -2.72367 | #N/A | #N/A | #N/A | #N/A | 0 | 0.116471 | 0.03809 | 0.05173 | 0.97201 | 0.96044 | 0.55955 | 0.58421 | 3 | 2.93229 | 0.55262 | 7.48021 | 4.055949 | Not Significant | NS | #N/A |
| 2095 | A0A5F9DN71 | #N/A | USF3 | RABBIT | #N/A | 0.19782 | #N/A | #N/A | #N/A | #N/A | #N/A | #N/A | 2.27796 | -1.65385 | 0.114234 | 0.03355 | 0.06049 | 0.97865 | 0.95472 | 0.56139 | 0.58315 | 1 | 2.93229 | 0.55262 | 7.72957 | 2.377751 | Not Significant | NS | #N/A |
| 1348 | A0A5F9DKR4 | Dynein axonemal heavy chain 17 OS=Oryctolagus c | DNAH17 | RABBIT | -0.05077 | #N/A | #N/A | #N/A | #N/A | #N/A | #N/A | #N/A | #N/A | 0.06243 | 0.113198 | #N/A | 0.10767 | #N/A | 0.9212 | #N/A | 0.57341 | 0 | 2.93229 | 0.55262 | #N/A | 0.552617 | Not Significant | NS | #N/A |
| 2569 | G1SM27 | #N/A | #N/A | RABBIT | #N/A | #N/A | #N/A | -0.07713 | 0.00611 | -0.31507 | 0.42107 | 1.12632 | -0.92251 | #N/A | 0.112965 | 0.16926 | 0.1718 | 0.87381 | 0.8685 | 0.53437 | 0.55917 | 4 | 2.93229 | 0.55262 | 0.59391 | 0.576446 | Not Significant | NS | #N/A |
| 914 | A0A5F9CCH8 | SEC23 interacting protein OS=Oryctolagus cunicul | SEC23IP | RABBIT | 1.01063 | #N/A | -0.50553 | 0.90468 | -1.99878 | #N/A | -0.11313 | 0.00963 | 0 | #N/A | 0.11275 | 0.13462 | 0.15049 | 0.89817 | 0.88414 | 0.54013 | 0.56374 | 5 | 2.93229 | 0.55262 | 1.20262 | 0.962335 | Not Significant | NS | #N/A |
| 1892 | A0A5F9D4D9 | #N/A | TRPC4 | RABBIT | #N/A | 0.0437 | #N/A | #N/A | #N/A | #N/A | 0.15412 | #N/A | #N/A | #N/A | 0.110424 | #N/A | 0.10504 | #N/A | 0.92312 | #N/A | 0.57345 | 0 | 2.93229 | 0.55262 | #N/A | 0.552617 | Not Significant | NS | #N/A |
| 173 | G1SP34 | Arp2/3 complex 34 kDa subunit OS=Oryctolagus cu | ARPC2 | RABBIT | 0.18024 | -0.20064 | -0.23969 | -0.0455 | -0.02552 | -0.27534 | 0.39018 | -0.10875 | 0.17743 | 0.03451 | 0.109827 | 0.80311 | 0.40656 | 0.44511 | 0.69218 | 0.38942 | 0.51022 | 8 | 2.93229 | 0.55262 | 0.04675 | 0.182437 | Not Significant | NS | #N/A |
| 724 | G1TA04 | Pre-mRNA-processing factor 19 OS=Oryctolagus cu | PRPF19 | RABBIT | -0.19357 | -0.7112 | 0.36951 | 0.0409 | -0.11192 | -1.78335 | 0.50365 | 0.83598 | -0.46951 | 0.85583 | 0.109773 | 0.20552 | 0.21202 | 0.8423 | 0.83599 | 0.52455 | 0.54964 | 8 | 2.93229 | 0.55262 | 0.7132 | 0.670127 | Not Significant | NS | #N/A |
| 2475 | A0A5F9D1C3 | #N/A | #N/A | RABBIT | #N/A | #N/A | #N/A | -0.05764 | #N/A | -0.01369 | #N/A | -0.41036 | 0.39759 | 0.22948 | 0.108395 | 0.27643 | 0.16742 | 0.80017 | 0.8726 | 0.51361 | 0.56055 | 3 | 2.93229 | 0.55262 | 0.12301 | 0.33536 | Not Significant | NS | #N/A |
| 2512 | G1TDN8 | #N/A | KRT73 | RABBIT | #N/A | #N/A | #N/A | -0.08274 | #N/A | #N/A | #N/A | #N/A | #N/A | 0.0251 | 0.107833 | #N/A | 0.10257 | #N/A | 0.92491 | #N/A | 0.57362 | 0 | 2.93229 | 0.55262 | #N/A | 0.552617 | Not Significant | NS | #N/A |
| 535 | A0A5F9C3W2 | protein-synthesizing GTPase OS=Oryctolagus cunic | EIF2S3 | RABBIT | 0.00661 | -0.92705 | -0.0254 | 0.65982 | 0.06605 | 0.2651 | 0.85231 | -0.16408 | 0.00505 | -0.6409 | 0.10749 | 0.30376 | 0.27669 | 0.76906 | 0.78718 | 0.50494 | 0.53841 | 8 | 2.93229 | 0.55262 | 0.31305 | 0.377308 | Not Significant | NS | #N/A |
| 877 | G1TAX9 | Multifunctional fusion protein OS=Oryctolagus cuni | SNAP25 | RABBIT | -1.8672 | 0.12124 | 0.48829 | -0.37952 | 0.7132 | -0.55362 | 0.07658 | 0.43899 | -0.61405 | 0.26459 | 0.107294 | 0.21157 | 0.21567 | 0.83773 | 0.83321 | 0.5237 | 0.54958 | 8 | 2.93229 | 0.55262 | 0.64295 | 0.618721 | Not Significant | NS | #N/A |
| 2255 | A0A5F9CT54 | #N/A | #N/A | RABBIT | #N/A | #N/A | 0.24778 | #N/A | -0.46196 | 0 | #N/A | #N/A | #N/A | #N/A | 0.107086 | 0.17422 | 0.12671 | 0.89019 | 0.90538 | 0.53768 | 0.56976 | 1 | 2.93229 | 0.55262 | 0.25187 | 0.476135 | Not Significant | NS | #N/A |
| 2432 | G1SER1 | #N/A | DTHD1 | RABBIT | #N/A | #N/A | 0.17424 | -0.51483 | #N/A | #N/A | #N/A | -0.06437 | #N/A | #N/A | 0.105927 | 0.1775 | 0.12583 | 0.88816 | 0.90604 | 0.53768 | 0.56976 | 1 | 2.93229 | 0.55262 | 0.23741 | 0.472458 | Not Significant | NS | #N/A |
| 2670 | A0A5F9CNI0 | #N/A | CSMD3 | RABBIT | #N/A | #N/A | #N/A | -0.07986 | #N/A | #N/A | #N/A | #N/A | 0.02222 | #N/A | 0.102071 | #N/A | 0.09709 | #N/A | 0.92891 | #N/A | 0.57439 | 0 | 2.93229 | 0.55262 | #N/A | 0.552617 | Not Significant | NS | #N/A |
| 2479 | A0A0A0MQP7 | #N/A | ACTA2 | RABBIT | #N/A | #N/A | #N/A | -0.07956 | #N/A | 0.02192 | #N/A | #N/A | #N/A | #N/A | 0.101481 | #N/A | 0.09653 | #N/A | 0.92932 | #N/A | 0.57439 | 0 | 2.93229 | 0.55262 | #N/A | 0.552617 | Not Significant | NS | #N/A |
| 133 | A0A5F9DJ93 | ATP-dependent 6-phosphofructokinase OS=Oryctol | PFKP | RABBIT | 0.01166 | 0.25073 | 0.10687 | -0.27017 | 0.02068 | -0.16701 | 0.02182 | #N/A | 0.77249 | -0.13063 | 0.100211 | 0.46403 | 0.30738 | 0.65671 | 0.7649 | 0.4707 | 0.53348 | 7 | 2.93229 | 0.55262 | 0.10364 | 0.236189 | Not Significant | NS | #N/A |
| 713 | A0A5F9C3Y7 | SLIT-ROBO Rho GTPase activating protein 2 OS=Ory | #N/A | RABBIT | -0.20819 | 0.43705 | -0.56568 | -0.42722 | 0.20647 | 0.40493 | -0.5786 | 0.77218 | -0.94649 | 0.28837 | 0.099594 | 0.26662 | 0.24791 | 0.7965 | 0.8088 | 0.51222 | 0.54212 | 8 | 2.93229 | 0.55262 | 0.34883 | 0.403488 | Not Significant | NS | #N/A |
| 1106 | G1SVT3 | Splicing factor 3B subunit 5 OS=Oryctolagus cunicu | SF3B5 | RABBIT | -2.28684 | -0.75724 | 0.17065 | 0.84251 | 0.34726 | #N/A | 0.13657 | 0.43333 | -1.08935 | -0.43406 | 0.098356 | 0.14212 | 0.15343 | 0.89099 | 0.88113 | 0.53768 | 0.56282 | 7 | 2.93229 | 0.55262 | 1.06427 | 0.913214 | Not Significant | NS | #N/A |
| 2176 | P41316 | #N/A | CRYAB | RABBIT | #N/A | #N/A | -0.57071 | 0.00326 | -0.06324 | -0.40198 | -1.32216 | -0.0145 | 0.85396 | 0.32264 | 0.097822 | 0.19343 | 0.18877 | 0.853 | 0.85449 | 0.52783 | 0.55474 | 6 | 2.93229 | 0.55262 | 0.47953 | 0.503526 | Not Significant | NS | #N/A |
| 886 | G1T1C5 | Immunoglobulin binding protein 1 OS=Oryctolagus | #N/A | RABBIT | 0.81103 | -1.36349 | #N/A | -0.05764 | 0.13307 | -1.33857 | 1.23468 | -0.31939 | 0.42807 | -0.12265 | 0.095687 | 0.15287 | 0.16184 | 0.88282 | 0.87467 | 0.53662 | 0.56083 | 7 | 2.93229 | 0.55262 | 0.87068 | 0.776779 | Not Significant | NS | #N/A |
| 2873 | G1T0H9 | #N/A | EXOC3 | RABBIT | #N/A | #N/A | #N/A | #N/A | -0.05329 | 0.0399 | #N/A | #N/A | #N/A | #N/A | 0.093189 | #N/A | 0.08864 | #N/A | 0.93507 | #N/A | 0.57601 | 0 | 2.93229 | 0.55262 | #N/A | 0.552617 | Not Significant | NS | #N/A |
| 766 | A0A5F9DAE4 | DDHD domain containing 1 OS=Oryctolagus cunicu | #N/A | RABBIT | 1.65949 | -1.02148 | -0.99734 | -0.77818 | 1.25465 | #N/A | 0.22913 | 0.34261 | -0.22913 | #N/A | 0.090776 | 0.11385 | 0.12543 | 0.91307 | 0.90296 | 0.54361 | 0.5696 | 6 | 2.93229 | 0.55262 | 1.19204 | 0.982131 | Not Significant | NS | #N/A |
| 310 | G1SS73 | ATP-dependent RNA helicase DDX1 OS=Oryctolagu | DDX1 | RABBIT | -0.80964 | -0.55187 | 0.21514 | 0.85277 | -0.15567 | -0.04449 | -0.42621 | 0.15037 | 0.21185 | 0.10632 | 0.08942 | 0.28421 | 0.24637 | 0.78347 | 0.80996 | 0.50847 | 0.54212 | 8 | 2.93229 | 0.55262 | 0.24748 | 0.329328 | Not Significant | NS | #N/A |
| 1804 | G1TLK9 | #N/A | ACADVL | RABBIT | #N/A | 0.1952 | #N/A | #N/A | #N/A | #N/A | #N/A | #N/A | 0 | 0.56726 | 0.088427 | 0.18 | 0.10727 | 0.88662 | 0.91982 | 0.53768 | 0.57327 | 1 | 2.93229 | 0.55262 | 0.16089 | 0.452999 | Not Significant | NS | #N/A |
| 386 | A0A5F9CGP0 | Histone H4 OS=Oryctolagus cuniculus OX=9986 PE | #N/A | RABBIT | -0.70537 | 0.2284 | 0.41327 | -0.12273 | 0.04357 | -1.07722 | 1.86641 | 0.31259 | -0.79417 | -0.03058 | 0.083977 | 0.15217 | 0.15809 | 0.88282 | 0.87727 | 0.53662 | 0.5612 | 8 | 2.93229 | 0.55262 | 0.76141 | 0.705405 | Not Significant | NS | #N/A |
| 2087 | A0A5F9CP79 | #N/A | #N/A | RABBIT | #N/A | #N/A | 0.17065 | 0.17062 | #N/A | #N/A | -0.39709 | 1.24412 | #N/A | -0.09006 | 0.081686 | 0.1256 | 0.12293 | 0.90799 | 0.90622 | 0.54323 | 0.56976 | 3 | 2.93229 | 0.55262 | 0.50754 | 0.529824 | Not Significant | NS | #N/A |
| 245 | G1T8Z0 | Peroxiredoxin-6 OS=Oryctolagus cuniculus OX=998 | PRDX6 | RABBIT | -0.29879 | 1.12776 | -0.34397 | 0.07805 | -0.69786 | -0.1265 | -0.53973 | 0.06214 | 0.47714 | 0.38687 | 0.078947 | 0.21713 | 0.19986 | 0.83354 | 0.84526 | 0.52241 | 0.55249 | 8 | 2.93229 | 0.55262 | 0.33049 | 0.390066 | Not Significant | NS | #N/A |
| 2321 | A0A5F9DR76 | #N/A | CPEB2 | RABBIT | #N/A | #N/A | 0.14245 | 0.1157 | -0.12468 | 0.0282 | #N/A | -0.42954 | 0.76871 | #N/A | 0.077969 | 0.21701 | 0.16246 | 0.83882 | 0.87557 | 0.52415 | 0.56083 | 4 | 2.93229 | 0.55262 | 0.19363 | 0.345476 | Not Significant | NS | #N/A |
| 1344 | A0A5F9CT85 | MARVEL domain-containing protein OS=Oryctolagu | #N/A | RABBIT | -1.16767 | -1.37751 | 1.04605 | #N/A | 1.079 | #N/A | 0.45814 | 0.60944 | -0.45814 | -0.72864 | 0.075228 | 0.09998 | 0.10961 | 0.92362 | 0.91514 | 0.54544 | 0.57202 | 6 | 2.93229 | 0.55262 | 1.13232 | 0.942015 | Not Significant | NS | #N/A |

| | | | | | | | | | | | | | | | | | | | | | | | | | | | | |
|---|---|---|---|---|---|---|---|---|---|---|---|---|---|---|---|---|---|---|---|---|---|---|---|---|---|---|---|---|
| 1752 | A0AAA9WW19 | #N/A | #N/A | RABBIT | #N/A | #N/A | 0.1486 | -0.3675 | 0.03501 | -0.18061 | -0.02191 | 0.12788 | 0.10672 | 0.02191 | 0.072097 | 0.53045 | 0.21821 | 0.61485 | 0.83217 | 0.45457 | 0.54924 | 6 | 2.93229 | 0.55262 | 0.03464 | 0.20468 | Not Significant | NS | #N/A |
| 511 | A0A5F9DT47 | Cullin associated and neddylation dissociated 2 (pu | CAND2 | RABBIT | 0.03951 | 0.05179 | #N/A | #N/A | 0.12652 | #N/A | #N/A | 1.06237 | -0.61243 | -0.02784 | 0.068092 | 0.13853 | 0.12532 | 0.89651 | 0.90383 | 0.53968 | 0.56972 | 4 | 2.93229 | 0.55262 | 0.36238 | 0.442848 | Not Significant | NS | #N/A |
| 608 | G1T5Y2 | CYFIP related Rac1 interactor B OS=Oryctolagus cu | #N/A | RABBIT | -0.05162 | 1.16612 | -3.75738 | 0.4106 | 0.0499 | 0.07614 | -0.67094 | -1.71399 | -0.10285 | 0.56843 | 0.067836 | 0.07201 | 0.08058 | 0.94436 | 0.93723 | 0.55171 | 0.57605 | 8 | 2.93229 | 0.55262 | 2.21836 | 1.771567 | Not Significant | NS | #N/A |
| 1954 | G1SVG6 | #N/A | NF1 | RABBIT | #N/A | #N/A | -2.30958 | 3.65535 | -0.60661 | 1.75707 | #N/A | -1.40783 | #N/A | 0.59323 | 0.067765 | 0.03388 | 0.04317 | 0.9746 | 0.96679 | 0.56016 | 0.58521 | 4 | 2.93229 | 0.55262 | 6.00168 | 3.696781 | Not Significant | NS | #N/A |
| 1571 | G1SX04 | Mucolipin 2 OS=Oryctolagus cuniculus OX=9986 GI | MCOLN2 | RABBIT | 0.13156 | 0.12683 | #N/A | -0.43983 | #N/A | #N/A | #N/A | 0.00662 | #N/A | #N/A | 0.067099 | 0.17687 | 0.09524 | 0.8759 | 0.92788 | 0.53468 | 0.57433 | 2 | 2.93229 | 0.55262 | 0.10794 | 0.372303 | Not Significant | NS | #N/A |
| 1286 | G1TI79 | Calcium/calmodulin dependent protein kinase I OS | CAMK1 | RABBIT | #N/A | -0.74706 | 0.16857 | 0.72629 | -0.01338 | 0.19682 | 0.95187 | 0.85588 | -0.40623 | -1.09814 | 0.066435 | 0.12928 | 0.13041 | 0.90077 | 0.89885 | 0.5409 | 0.56826 | 7 | 2.93229 | 0.55262 | 0.58685 | 0.576741 | Not Significant | NS | #N/A |
| 2071 | G1SUN1 | #N/A | ARL6IP5 | RABBIT | #N/A | #N/A | -1.91578 | 0.74842 | #N/A | -0.18932 | 0.46607 | 0.33066 | -3.20033 | 0 | 0.065094 | 0.04861 | 0.05769 | 0.96311 | 0.95543 | 0.55731 | 0.58322 | 5 | 2.93229 | 0.55262 | 2.56177 | 1.819054 | Not Significant | NS | #N/A |
| 2225 | G1T6X0 | #N/A | WDR91 | RABBIT | #N/A | #N/A | -0.30606 | -0.12377 | #N/A | 0.3566 | #N/A | -1.26897 | 0.06613 | 0.24168 | 0.06377 | 0.11211 | 0.10599 | 0.91613 | 0.91859 | 0.54454 | 0.57313 | 4 | 2.93229 | 0.55262 | 0.43137 | 0.482654 | Not Significant | NS | #N/A |
| 506 | G1U5Q7 | Actin-related protein 2/3 complex subunit 4 OS=Ory | ARPC4 | RABBIT | 0.04983 | -0.10499 | -0.02693 | -0.0958 | -0.06505 | -0.48916 | 0.26588 | 0.03556 | 0.22102 | 0.04205 | 0.063659 | 0.46394 | 0.2355 | 0.65505 | 0.81817 | 0.4707 | 0.54372 | 8 | 2.93229 | 0.55262 | 0.04707 | 0.182669 | Not Significant | NS | #N/A |
| 795 | G1T704 | Sorbitol dehydrogenase OS=Oryctolagus cuniculus | SORD | RABBIT | 1.12059 | 1.09021 | -1.14007 | -0.1618 | -0.61109 | 0.31976 | 0.6838 | -0.38308 | 0.10416 | -0.11505 | 0.062347 | 0.12737 | 0.12871 | 0.90179 | 0.89992 | 0.54129 | 0.56842 | 8 | 2.93229 | 0.55262 | 0.59903 | 0.586582 | Not Significant | NS | #N/A |
| 2163 | G1TFM5 | #N/A | RPS5 | RABBIT | #N/A | #N/A | 0.13856 | -0.17294 | 0.01871 | -0.27449 | 0.43481 | 0.24609 | -0.28231 | 0.15701 | 0.061446 | 0.30292 | 0.17422 | 0.77218 | 0.86558 | 0.50582 | 0.55792 | 6 | 2.93229 | 0.55262 | 0.07715 | 0.233234 | Not Significant | NS | #N/A |
| 959 | G1TD94 | LDL receptor related protein 1B OS=Oryctolagus cu | LRP1B | RABBIT | 3.31823 | -3.64684 | #N/A | -0.00699 | #N/A | #N/A | -0.05065 | #N/A | #N/A | #N/A | 0.061215 | 0.01522 | 0.02314 | 0.98924 | 0.98245 | 0.56305 | 0.58933 | 2 | 2.93229 | 0.55262 | 12.1363 | 5.24969 | Not Significant | NS | #N/A |
| 1457 | A5U514 | Cobalt ABC transporter ATP-binding protein OS=My | #N/A | MT | 0.01166 | #N/A | #N/A | #N/A | #N/A | #N/A | -0.2273 | 0.37287 | #N/A | #N/A | 0.061123 | 0.1176 | 0.07375 | 0.92548 | 0.94481 | 0.54588 | 0.57916 | 1 | 2.93229 | 0.55262 | 0.18011 | 0.457886 | Not Significant | NS | #N/A |
| 1700 | A0A5F9DPP1 | #N/A | #N/A | RABBIT | #N/A | -0.81451 | 0.71975 | 0.22317 | -0.46799 | 0.15866 | #N/A | -0.17165 | -0.19203 | 0.10729 | 0.060462 | 0.16977 | 0.14416 | 0.87077 | 0.88858 | 0.53296 | 0.56489 | 6 | 2.93229 | 0.55262 | 0.25368 | 0.351818 | Not Significant | NS | #N/A |
| 2525 | G1T9I3 | #N/A | PLAA | RABBIT | #N/A | #N/A | #N/A | 0.18307 | #N/A | #N/A | #N/A | -0.30508 | 3.31125 | -2.27672 | 0.06008 | 0.01836 | 0.02748 | 0.98702 | 0.97916 | 0.56303 | 0.58931 | 2 | 2.93229 | 0.55262 | 8.03176 | 3.585346 | Not Significant | NS | #N/A |
| 741 | G1T9V1 | Pyruvate dehydrogenase E1 component subunit bet | PDHB | RABBIT | -1.13973 | -1.47649 | 0.7182 | -0.10459 | 0.25503 | -0.31035 | -3.45996 | 1.46081 | 0.04695 | 0.81137 | 0.059284 | 0.06266 | 0.07015 | 0.95157 | 0.94534 | 0.55456 | 0.57928 | 8 | 2.93229 | 0.55262 | 2.23761 | 1.785659 | Not Significant | NS | #N/A |
| 2413 | A0A5F9CZN0 | #N/A | ABCF1 | RABBIT | #N/A | #N/A | 0.00784 | 0.10517 | #N/A | #N/A | #N/A | #N/A | -0.2394 | 0.46498 | 0.056284 | 0.15831 | 0.09133 | 0.88876 | 0.93082 | 0.53768 | 0.5747 | 2 | 2.93229 | 0.55262 | 0.12641 | 0.379794 | Not Significant | NS | #N/A |
| 132 | G1TUX2 | Aconitate hydratase, mitochondrial OS=Oryctolagu | ACO2 | RABBIT | -0.31748 | 0.33395 | -0.29859 | 0.34759 | -0.26947 | -0.01483 | -1.04088 | 0.44328 | 0.66367 | 0.01483 | 0.054013 | 0.16261 | 0.14434 | 0.87486 | 0.88786 | 0.53468 | 0.56464 | 8 | 2.93229 | 0.55262 | 0.27582 | 0.350061 | Not Significant | NS | #N/A |
| 2269 | U3KPG5 | #N/A | SRFBP1 | RABBIT | #N/A | #N/A | 0.42141 | #N/A | -0.52704 | 0 | #N/A | #N/A | #N/A | #N/A | 0.052813 | 0.0643 | 0.05943 | 0.95912 | 0.95551 | 0.55696 | 0.58322 | 1 | 2.93229 | 0.55262 | 0.44978 | 0.526464 | Not Significant | NS | #N/A |
| 933 | G1SV81 | Dynactin subunit 5 OS=Oryctolagus cuniculus OX=9 | DCTN5 | RABBIT | 0.26136 | -1.8847 | -0.15154 | 0.22081 | 0.85577 | -0.78496 | 0.7237 | 0.31234 | -0.2497 | -0.43752 | 0.052435 | 0.09754 | 0.10077 | 0.9247 | 0.92156 | 0.54564 | 0.57341 | 8 | 2.93229 | 0.55262 | 0.72252 | 0.676947 | Not Significant | NS | #N/A |
| 1739 | G1U758 | #N/A | #N/A | RABBIT | #N/A | 0.426 | -1.36237 | -0.31114 | 0.81619 | 0.68341 | #N/A | #N/A | -0.85002 | 0 | 0.052291 | 0.07718 | 0.08181 | 0.94147 | 0.93682 | 0.55054 | 0.57605 | 5 | 2.93229 | 0.55262 | 0.78686 | 0.700272 | Not Significant | NS | #N/A |
| 496 | A0A5F9CRG2 | Coiled-coil domain containing 91 OS=Oryctolagus c | CCDC91 | RABBIT | -0.3446 | -0.79543 | 0.66886 | -0.52788 | 1.22855 | #N/A | 0.66539 | 0.2919 | -0.66368 | #N/A | 0.051968 | 0.08808 | 0.09039 | 0.93268 | 0.92997 | 0.548 | 0.57459 | 6 | 2.93229 | 0.55262 | 0.65266 | 0.61982 | Not Significant | NS | #N/A |
| 2400 | G1SWW1 | #N/A | RUNDC1 | RABBIT | #N/A | #N/A | 0.00673 | -0.05764 | #N/A | 0.37495 | #N/A | -0.48398 | 0.18505 | #N/A | 0.050796 | 0.15066 | 0.09513 | 0.8898 | 0.92734 | 0.53768 | 0.5742 | 3 | 2.93229 | 0.55262 | 0.1364 | 0.342134 | Not Significant | NS | #N/A |
| 1630 | G1SMN9 | Armadillo repeat containing X-linked 3 OS=Oryctola | ARMCX3 | RABBIT | -1.07995 | -1.45332 | 0.14513 | 0.46636 | 0.70321 | 0.04448 | 0.02552 | -0.52918 | -0.67201 | 0.15905 | 0.049287 | 0.10648 | 0.10603 | 0.91782 | 0.91748 | 0.54531 | 0.57285 | 8 | 2.93229 | 0.55262 | 0.53563 | 0.540186 | Not Significant | NS | #N/A |
| 196 | A0A5F9DTD6 | Chromosome 1 open reading frame 43 OS=Oryctola | C1orf43 | RABBIT | 0.49115 | 0.19782 | -0.08296 | -0.07215 | 0.45255 | #N/A | 1.38641 | -0.07009 | -0.36058 | 0.02932 | 0.048982 | 0.13274 | 0.11902 | 0.89813 | 0.90763 | 0.54013 | 0.5704 | 7 | 2.93229 | 0.55262 | 0.30257 | 0.376391 | Not Significant | NS | #N/A |
| 417 | G1TS93 | U2A'/phosphoprotein 32 family A C-terminal domai | #N/A | RABBIT | -0.17118 | 0.49542 | -0.19942 | 0.45153 | 0.0326 | 0.2831 | -0.04598 | -0.23398 | 0.91625 | -0.06554 | 0.048981 | 0.19359 | 0.15035 | 0.85132 | 0.88323 | 0.52779 | 0.56358 | 8 | 2.93229 | 0.55262 | 0.16004 | 0.265335 | Not Significant | NS | #N/A |
| 2131 | A0AAG1W3S3 | #N/A | #N/A | RABBIT | #N/A | #N/A | 0.04986 | -0.95715 | 0.28134 | -0.75994 | 0.15215 | 0.05642 | -0.37717 | 0.12867 | 0.048676 | 0.13322 | 0.11274 | 0.89837 | 0.91273 | 0.54013 | 0.57143 | 6 | 2.93229 | 0.55262 | 0.25031 | 0.349553 | Not Significant | NS | #N/A |
| 2257 | A0A5F9DBJ4 | #N/A | CAB39L | RABBIT | #N/A | #N/A | 0.17065 | -0.19386 | 0.03798 | #N/A | 0.92683 | 0.88526 | -0.96283 | -0.63539 | 0.04854 | 0.08164 | 0.083 | 0.9381 | 0.93591 | 0.54911 | 0.57601 | 5 | 2.93229 | 0.55262 | 0.60602 | 0.586278 | Not Significant | NS | #N/A |
| 1740 | G1TJW1 | #N/A | RPS8 | RABBIT | #N/A | -1.96832 | 0.54045 | -0.48832 | 1.42094 | -0.61062 | -0.00075 | 0.05933 | 0 | 0.16774 | 0.046955 | 0.07135 | 0.0763 | 0.94512 | 0.94069 | 0.55171 | 0.57754 | 7 | 2.93229 | 0.55262 | 0.96251 | 0.841496 | Not Significant | NS | #N/A |
| 2743 | G1TN22 | #N/A | PPIL2 | RABBIT | #N/A | #N/A | #N/A | -0.08422 | #N/A | #N/A | #N/A | -0.03779 | #N/A | #N/A | 0.046426 | #N/A | 0.04416 | #N/A | 0.96761 | #N/A | 0.58521 | 0 | 2.93229 | 0.55262 | #N/A | 0.552617 | Not Significant | NS | #N/A |
| 1319 | G1TM83 | Small nuclear ribonucleoprotein polypeptide A OS= | SNRPA | RABBIT | -1.09292 | -1.02267 | 0.17065 | 0.48965 | 0.09785 | #N/A | 0.02808 | -0.11216 | -1.08805 | 0.26989 | 0.045927 | 0.10099 | 0.0981 | 0.92239 | 0.9238 | 0.54531 | 0.57362 | 7 | 2.93229 | 0.55262 | 0.45954 | 0.48702 | Not Significant | NS | #N/A |
| 2417 | U3KNP0 | #N/A | HFM1 | RABBIT | #N/A | #N/A | -0.01896 | 0.03991 | -0.00474 | 0.93483 | -0.41756 | -0.32157 | #N/A | 0 | 0.043522 | 0.11913 | 0.09653 | 0.90981 | 0.9255 | 0.54361 | 0.57362 | 5 | 2.93229 | 0.55262 | 0.2288 | 0.348501 | Not Significant | NS | #N/A |
| 2433 | A0A5F9CX93 | #N/A | #N/A | RABBIT | #N/A | #N/A | 0.06416 | #N/A | #N/A | 0.1065 | #N/A | #N/A | #N/A | #N/A | 0.04234 | #N/A | 0.04027 | #N/A | 0.97046 | #N/A | 0.5865 | 0 | 2.93229 | 0.55262 | #N/A | 0.552617 | Not Significant | NS | #N/A |
| 2754 | G1SHI5 | #N/A | SMYD5 | RABBIT | #N/A | #N/A | #N/A | -0.01655 | #N/A | -0.04109 | -0.29265 | -0.3521 | 0.39528 | 0.41518 | 0.041478 | 0.10336 | 0.06788 | 0.92265 | 0.9478 | 0.54531 | 0.58017 | 4 | 2.93229 | 0.55262 | 0.13421 | 0.31119 | Not Significant | NS | #N/A |
| 1146 | G1T873 | HECT and RLD domain containing E3 ubiquitin prote | HERC1 | RABBIT | #N/A | #N/A | -0.17448 | 0.85726 | -0.64589 | -0.25339 | 0.25339 | 0.24471 | 0.77186 | -0.74981 | 0.041056 | 0.08685 | 0.08263 | 0.93362 | 0.93597 | 0.54802 | 0.57601 | 6 | 2.93229 | 0.55262 | 0.41901 | 0.462874 | Not Significant | NS | #N/A |
| 2674 | P71719 | #N/A | #N/A | MT | #N/A | #N/A | #N/A | -0.05618 | #N/A | 0.20464 | #N/A | -0.06582 | -0.1854 | #N/A | 0.040659 | 0.17622 | 0.05997 | 0.87635 | 0.95453 | 0.53468 | 0.58315 | 2 | 2.93229 | 0.55262 | 0.03993 | 0.344726 | Not Significant | NS | #N/A |
| 2843 | G1TIF2 | #N/A | WDFY3 | RABBIT | #N/A | #N/A | #N/A | #N/A | 0.54922 | -0.04985 | #N/A | 2.9098 | -0.50849 | 0 | 0.038649 | 0.02209 | 0.02812 | 0.98376 | 0.97849 | 0.56255 | 0.58927 | 3 | 2.93229 | 0.55262 | 2.44855 | 1.511404 | Not Significant | NS | #N/A |
| 1692 | P15122 | #N/A | AKR1B1 | RABBIT | #N/A | 2.39478 | -1.0997 | 0.03741 | -1.05406 | -0.09505 | 0.30934 | #N/A | #N/A | #N/A | 0.037541 | 0.03043 | 0.03657 | 0.97718 | 0.97186 | 0.56077 | 0.58693 | 4 | 2.93229 | 0.55262 | 2.02979 | 1.404958 | Not Significant | NS | #N/A |
| 1821 | G1SRX0 | #N/A | WDR70 | RABBIT | #N/A | -0.22798 | #N/A | -0.44109 | #N/A | 2.14125 | 0.45744 | #N/A | 0.38345 | -4.17915 | 0.03528 | 0.01734 | 0.02204 | 0.98699 | 0.98304 | 0.56303 | 0.58933 | 4 | 2.93229 | 0.55262 | 5.51775 | 3.417549 | Not Significant | NS | #N/A |
| 1746 | G1TLW0 | #N/A | AMDHD2 | RABBIT | #N/A | 1.21134 | #N/A | -0.77868 | -0.36558 | #N/A | 0.0241 | #N/A | -0.0241 | 0.17122 | 0.034712 | 0.05698 | 0.05707 | 0.95729 | 0.9561 | 0.55612 | 0.58337 | 4 | 2.93229 | 0.55262 | 0.55664 | 0.554936 | Not Significant | NS | #N/A |
| 1037 | G1T0Z6 | RBR-type E3 ubiquitin transferase OS=Oryctolagus ( | ARIH1 | RABBIT | -1.28343 | 0.84151 | -1.71712 | 0.77039 | 0.20972 | -0.5544 | 0.19414 | 0.36515 | -0.19414 | -0.81659 | 0.034618 | 0.06006 | 0.06295 | 0.95358 | 0.95094 | 0.55492 | 0.58146 | 8 | 2.93229 | 0.55262 | 0.83049 | 0.755956 | Not Significant | NS | #N/A |
| 1283 | G1SP27 | Coiled-coil domain containing 6 OS=Oryctolagus cu | CCDC6 | RABBIT | -1.32877 | #N/A | #N/A | 0.40862 | -0.00078 | #N/A | -0.9581 | -0.07697 | #N/A | 0.2155 | 0.033784 | 0.05346 | 0.05436 | 0.95993 | 0.95818 | 0.55716 | 0.58399 | 4 | 2.93229 | 0.55262 | 0.59908 | 0.579425 | Not Significant | NS | #N/A |
| 1449 | G1TXG5 | Large ribosomal subunit protein eL34 OS=Oryctolag | RPL34 | RABBIT | #N/A | #N/A | #N/A | -0.0455 | #N/A | #N/A | #N/A | #N/A | -0.01214 | #N/A | 0.033355 | #N/A | 0.03173 | #N/A | 0.97672 | #N/A | 0.58862 | 0 | 2.93229 | 0.55262 | #N/A | 0.552617 | Not Significant | NS | #N/A |
| 70 | G1TR82 | Tubulin alpha chain OS=Oryctolagus cuniculus OX= | TUBA4A | RABBIT | -1.98427 | 2.60786 | 3.5444 | -0.06928 | -1.76427 | -0.12238 | 2.67081 | -0.05272 | -0.11348 | 0.10506 | 0.030572 | 0.02445 | 0.02787 | 0.98109 | 0.97827 | 0.56183 | 0.58927 | 8 | 2.93229 | 0.55262 | 3.90827 | 3.008205 | Not Significant | NS | #N/A |
| 2576 | A0A5F9CCN8 | #N/A | CNOT4 | RABBIT | #N/A | #N/A | #N/A | -0.04345 | #N/A | -0.01419 | #N/A | #N/A | #N/A | #N/A | 0.02926 | #N/A | 0.02783 | #N/A | 0.97958 | #N/A | 0.58931 | 0 | 2.93229 | 0.55262 | #N/A | 0.552617 | Not Significant | NS | #N/A |
| 232 | A0A5F9C1E0 | X-prolyl aminopeptidase 1 OS=Oryctolagus cunicul | XPNPEP1 | RABBIT | 0.00967 | -0.11952 | -0.00255 | -0.14299 | -0.01139 | 0.24006 | 0.09589 | -0.99378 | 0.39884 | 0.13527 | 0.028611 | 0.11447 | 0.08829 | 0.91169 | 0.93124 | 0.54361 | 0.57471 | 8 | 2.93229 | 0.55262 | 0.15618 | 0.262515 | Not Significant | NS | #N/A |
| 2516 | G1TBL0 | #N/A | BZW2 | RABBIT | #N/A | #N/A | #N/A | -0.00788 | #N/A | 0.20833 | -0.13622 | -0.24687 | -0.04976 | 0.32355 | 0.027685 | 0.10583 | 0.04894 | 0.92081 | 0.96235 | 0.54531 | 0.58421 | 4 | 2.93229 | 0.55262 | 0.05703 | 0.266656 | Not Significant | NS | #N/A |
| 2602 | A0A5F9CC91 | #N/A | AACS | RABBIT | #N/A | #N/A | #N/A | -0.05764 | #N/A | 0.96299 | #N/A | -1.1181 | -0.72923 | 0.74616 | 0.023093 | 0.01981 | 0.02277 | 0.98544 | 0.98258 | 0.56303 | 0.58933 | 3 | 2.93229 | 0.55262 | 1.08708 | 0.822901 | Not Significant | NS | #N/A |
| 2607 | A0A5F9DSQ8 | #N/A | ARL6 | RABBIT | #N/A | #N/A | #N/A | -0.11021 | #N/A | 0.05257 | #N/A | -0.42004 | #N/A | 0.09195 | 0.01837 | 0.05584 | 0.02646 | 0.96055 | 0.97993 | 0.55716 | 0.58931 | 2 | 2.93229 | 0.55262 | 0.08117 | 0.361451 | Not Significant | NS | #N/A |
| 1670 | A0A5F9CIR3 | #N/A | #N/A | RABBIT | #N/A | -0.06191 | #N/A | 0.44475 | -0.60849 | 0.12806 | #N/A | -0.72721 | 0.36993 | 0 | 0.01791 | 0.0474 | 0.03916 | 0.96403 | 0.96973 | 0.55731 | 0.58626 | 5 | 2.93229 | 0.55262 | 0.24477 | 0.358573 | Not Significant | NS | #N/A |
| 1695 | A0A5F9DBS7 | #N/A | DST | RABBIT | #N/A | 0.09002 | #N/A | #N/A | #N/A | #N/A | #N/A | #N/A | 0.1078 | #N/A | 0.017778 | #N/A | 0.01691 | #N/A | 0.98759 | #N/A | 0.59037 | 0 | 2.93229 | 0.55262 | #N/A | 0.552617 | Not Significant | NS | #N/A |
| 2142 | A0A5F9DG98 | #N/A | TRIM42 | RABBIT | #N/A | #N/A | 1.26602 | #N/A | -0.95168 | #N/A | 0 | 1.52311 | -0.99841 | #N/A | 0.01773 | 0.01411 | 0.0175 | 0.98963 | 0.98661 | 0.56305 | 0.59037 | 3 | 2.93229 | 0.55262 | 1.89467 | 1.231305 | Not Significant | NS | #N/A |
| 2223 | A0A5F9DDX9 | #N/A | #N/A | RABBIT | #N/A | #N/A | -0.22973 | #N/A | #N/A | -1.73868 | 0.32569 | 0.13321 | 0.41139 | -0.19757 | 0.016536 | 0.01707 | 0.01824 | 0.9872 | 0.98596 | 0.56303 | 0.59037 | 4 | 2.93229 | 0.55262 | 0.78196 | 0.68495 | Not Significant | NS | #N/A |
| 1317 | G1TEF7 | Piwi like RNA-mediated gene silencing 3 OS=Orycto | PIWIL3 | RABBIT | #N/A | #N/A | #N/A | 0.02596 | -0.09698 | #N/A | -0.60886 | #N/A | 0.56908 | #N/A | 0.015625 | 0.02639 | 0.02277 | 0.98134 | 0.98272 | 0.56183 | 0.58933 | 2 | 2.93229 | 0.55262 | 0.35067 | 0.470727 | Not Significant | NS | #N/A |
| 2339 | G1SKT7 | #N/A | #N/A | RABBIT | #N/A | #N/A | 0.17065 | #N/A | #N/A | -0.18424 | #N/A | 0.63932 | -0.37816 | 0.66615 | 0.015117 | 0.0248 | 0.02078 | 0.98177 | 0.9841 | 0.56185 | 0.58975 | 3 | 2.93229 | 0.55262 | 0.29713 | 0.423417 | Not Significant | NS | #N/A |
| 689 | G1SMR7 | Large ribosomal subunit protein uL11 OS=Oryctolag | RPL12 | RABBIT | -1.40358 | -0.33398 | 0.83127 | -0.08282 | 0.60249 | -0.4936 | -0.39332 | 0.49679 | 0.02518 | 0.05374 | 0.015084 | 0.03487 | 0.03405 | 0.97304 | 0.97345 | 0.5597 | 0.58747 | 8 | 2.93229 | 0.55262 | 0.46773 | 0.490499 | Not Significant | NS | #N/A |
| 613 | G1SIX4 | Tubulin polymerization promoting protein OS=Oryct | TPPP | RABBIT | -0.1468 | -0.26267 | -0.05889 | 0.2913 | 0.14508 | 0.43464 | -0.8605 | -0.31235 | 0.58606 | 0.19168 | 0.014303 | 0.05046 | 0.04162 | 0.96099 | 0.96755 | 0.55716 | 0.58521 | 8 | 2.93229 | 0.55262 | 0.20082 | 0.295179 | Not Significant | NS | #N/A |
| 586 | A0A5F9DUC2 | Neurobeachin OS=Oryctolagus cuniculus OX=9986 | NBEA | RABBIT | -0.38474 | -0.85495 | -0.0338 | 1.63842 | 0.2408 | -0.81512 | -0.86269 | 1.2693 | 0.8759 | 0.20445 | 0.013219 | 0.0219 | 0.02315 | 0.98306 | 0.98194 | 0.56237 | 0.58933 | 8 | 2.93229 | 0.55262 | 0.91096 | 0.814841 | Not Significant | NS | #N/A |
| 2702 | Q7M3D2 | #N/A | #N/A | RABBIT | #N/A | #N/A | #N/A | 0.00869 | #N/A | -0.06633 | 0.24748 | #N/A | #N/A | -0.12652 | 0.009525 | 0.04108 | 0.01405 | 0.97097 | 0.98934 | 0.55939 | 0.59097 | 2 | 2.93229 | 0.55262 | 0.04033 | 0.344888 | Not Significant | NS | #N/A |
| 260 | G1SSC9 | Importin 7 OS=Oryctolagus cuniculus OX=9986 GN | IPO7 | RABBIT | 0.20861 | 0.02438 | -0.15004 | -0.06226 | 0.33737 | 0.00462 | 0.53645 | -0.0927 | 0.08934 | -0.1374 | 0.008449 | 0.05634 | 0.0307 | 0.95646 | 0.97606 | 0.55607 | 0.58845 | 8 | 2.93229 | 0.55262 | 0.05623 | 0.189376 | Not Significant | NS | #N/A |
| 2729 | A0A5F9CYZ1 | #N/A | #N/A | RABBIT | #N/A | #N/A | #N/A | #N/A | -0.01338 | -0.72205 | #N/A | 0.12237 | 0.63604 | -0.06717 | 0.005681 | 0.00905 | 0.00773 | 0.99334 | 0.99409 | 0.56449 | 0.59222 | 3 | 2.93229 | 0.55262 | 0.31505 | 0.432477 | Not Significant | NS | #N/A |
| 1639 | G1TIR7 | U6 snRNA-associated Sm-like protein LSm8 OS=Ory | LSM8 | RABBIT | #N/A | 0.24449 | -0.24249 | 0.46562 | -0.48224 | #N/A | 0 | #N/A | #N/A | #N/A | 0.003655 | 0.00751 | 0.00538 | 0.99448 | 0.99588 | 0.56449 | 0.59254 | 3 | 2.93229 | 0.55262 | 0.1893 | 0.368882 | Not Significant | NS | #N/A |
| 2629 | G1SX50 | #N/A | RBM19 | RABBIT | #N/A | #N/A | #N/A | -0.02982 | #N/A | #N/A | #N/A | #N/A | #N/A | -0.02781 | 0.002009 | #N/A | 0.00191 | #N/A | 0.9986 | #N/A | 0.59285 | 0 | 2.93229 | 0.55262 | #N/A | 0.552617 | Not Significant | NS | #N/A |
| 2636 | A5U215 | #N/A | #N/A | MT | #N/A | #N/A | #N/A | 0.03279 | -0.10381 | -0.25347 | #N/A | #N/A | #N/A | 0.18354 | 0.000544 | 0.00238 | 0.00092 | 0.99832 | 0.9993 | 0.56535 | 0.59306 | 2 | 2.93229 | 0.55262 | 0.05241 | 0.349787 | Not Significant | NS | #N/A |
| 1261 | A0A5F9CZ99 | Cysteine-rich protein 2 OS=Oryctolagus cuniculus ( | #N/A | RABBIT | 0.35454 | -0.00283 | #N/A | #N/A | -0.01486 | 0.86944 | 0.03736 | -0.06437 | #N/A | -0.39945 | -0.00154 | -0.0046 | -0.00353 | 0.99651 | 0.99727 | 0.56498 | 0.59271 | 5 | 2.93229 | 0.55262 | 0.19202 | 0.325317 | Not Significant | NS | #N/A |
| 2123 | A0A5F9C4D8 | #N/A | #N/A | RABBIT | #N/A | #N/A | -0.05346 | 0.05726 | #N/A | #N/A | 0 | #N/A | #N/A | #N/A | -0.0019 | -0.01981 | -0.00241 | 0.98739 | 0.99819 | 0.56303 | 0.59281 | 1 | 2.93229 | 0.55262 | 0.00613 | 0.413642 | Not Significant | NS | #N/A |
| 1466 | G1SZI0 | SPG7 matrix AAA peptidase subunit, paraplegin OS= | SPG7 | RABBIT | 0.53765 | -0.22473 | 0.02149 | -0.05764 | #N/A | -0.38345 | 0.63648 | 0.1908 | 0.12213 | -0.23213 | -0.00242 | -0.00976 | -0.00709 | 0.99249 | 0.99449 | 0.56423 | 0.59225 | 7 | 2.93229 | 0.55262 | 0.13704 | 0.259728 | Not Significant | NS | #N/A |
| 1601 | G1SLS0 | WBP2 N-terminal like OS=Oryctolagus cuniculus OX | #N/A | RABBIT | 0.12289 | #N/A | #N/A | -0.16886 | #N/A | -0.72897 | #N/A | #N/A | 0.67784 | #N/A | -0.00258 | -0.00359 | -0.00351 | 0.99746 | 0.99733 | 0.5653 | 0.59271 | 2 | 2.93229 | 0.55262 | 0.51606 | 0.537795 | Not Significant | NS | #N/A |
| 2418 | A0A5F9CTZ7 | #N/A | ARFGEF1 | RABBIT | #N/A | #N/A | #N/A | -0.16561 | #N/A | -1.11699 | #N/A | #N/A | 0.10797 | 0.50439 | -0.0026 | -0.00266 | -0.00286 | 0.99812 | 0.99783 | 0.56535 | 0.5928 | 2 | 2.93229 | 0.55262 | 0.71443 | 0.618231 | Not Significant | NS | #N/A |
| 267 | G1SDU6 | threonine--tRNA ligase OS=Oryctolagus cuniculus ( | TARS1 | RABBIT | 0.20467 | -0.25859 | -0.24607 | -0.09681 | 0.34293 | -0.14685 | -0.40308 | 0.23596 | 0.20728 | 0.03917 | -0.00273 | -0.01604 | -0.00962 | 0.9876 | 0.9925 | 0.56303 | 0.59209 | 8 | 2.93229 | 0.55262 | 0.07237 | 0.201187 | Not Significant | NS | #N/A |
| 2024 | A0A5F9D8H3 | #N/A | SEC22B | RABBIT | #N/A | -0.57738 | #N/A | 0.16417 | -0.01338 | #N/A | -0.90105 | 0.43482 | -0.7236 | 0.60789 | -0.00329 | -0.00663 | -0.00628 | 0.99497 | 0.99515 | 0.56452 | 0.59244 | 5 | 2.93229 | 0.55262 | 0.42185 | 0.470191 | Not Significant | NS | #N/A |
| 1411 | G1SVJ4 | Bromodomain and PHD finger containing 3 OS=Oryc | BRPF3 | RABBIT | 0 | #N/A | #N/A | 0.00744 | #N/A | #N/A | #N/A | #N/A | 0 | #N/A | -0.00372 | -0.57735 | -0.00473 | 0.66667 | 0.99646 | 0.47235 | 0.5926 | 1 | 2.93229 | 0.55262 | 2.8E-05 | 0.412091 | Not Significant | NS | #N/A |
| 1761 | A0A5F9C4A3 | #N/A | RTN4 | RABBIT | #N/A | -0.2429 | 0.29801 | -0.33842 | -0.01338 | -0.56355 | 0.61546 | 0.43191 | -0.99074 | 0.10859 | -0.00549 | -0.015 | -0.0134 | 0.98845 | 0.98957 | 0.56303 | 0.59097 | 7 | 2.93229 | 0.55262 | 0.29787 | 0.373075 | Not Significant | NS | #N/A |
| 2055 | Q864X1 | #N/A | hspb3 | RABBIT | #N/A | 0.06959 | #N/A | #N/A | #N/A | #N/A | #N/A | 0.06387 | #N/A | #N/A | -0.00572 | #N/A | -0.00544 | #N/A | 0.99601 | #N/A | 0.59254 | 0 | 2.93229 | 0.55262 | #N/A | 0.552617 | Not Significant | NS | #N/A |
| 251 | A0A5F9CFU9 | Brain abundant membrane attached signal protein 1 | BASP1 | RABBIT | -1.00245 | 0.43295 | -0.31335 | -0.08677 | 0.64519 | -0.74809 | -0.41208 | 0.43685 | 0.02913 | 0.33667 | -0.00662 | -0.018 | -0.01664 | 0.98608 | 0.98703 | 0.56303 | 0.59037 | 8 | 2.93229 | 0.55262 | 0.33778 | 0.395401 | Not Significant | NS | #N/A |
| 844 | A0A5F9CGM6 | calcium/calmodulin-dependent protein kinase OS= | CAMK2A | RABBIT | -2.01588 | -1.32587 | 0.00857 | 0.10444 | 0.49926 | 0.79633 | -2.46663 | 0.66667 | 0.80355 | -2.56302 | -0.00672 | -0.0072 | -0.00805 | 0.99443 | 0.99372 | 0.56449 | 0.59221 | 8 | 2.93229 | 0.55262 | 2.18033 | 1.743737 | Not Significant | NS | #N/A |
| 2427 | A0A5F9CF89 | #N/A | #N/A | RABBIT | #N/A | #N/A | -0.12209 | #N/A | #N/A | 0.39747 | 0.19763 | 0.17045 | -1.21925 | -0.19763 | -0.00818 | -0.01156 | -0.01084 | 0.99133 | 0.99166 | 0.56379 | 0.592 | 4 | 2.93229 | 0.55262 | 0.41672 | 0.474203 | Not Significant | NS | #N/A |
| 2356 | A0A5F9DBK4 | #N/A | GPRC5B | RABBIT | #N/A | #N/A | 0.17065 | -0.29359 | 0.50768 | #N/A | #N/A | 0.31921 | -0.08116 | #N/A | -0.00923 | -0.02754 | -0.0173 | 0.97976 | 0.98676 | 0.5618 | 0.59037 | 3 | 2.93229 | 0.55262 | 0.13462 | 0.341233 | Not Significant | NS | #N/A |
| 2858 | G1U897 | #N/A | GULP1 | RABBIT | #N/A | #N/A | #N/A | #N/A | -0.00197 | #N/A | #N/A | #N/A | #N/A | -0.01141 | -0.00944 | #N/A | -0.00898 | #N/A | 0.99341 | #N/A | 0.59221 | 0 | 2.93229 | 0.55262 | #N/A | 0.552617 | Not Significant | NS | #N/A |
| 1856 | A0A5F9C3H3 | #N/A | SLC26A6 | RABBIT | #N/A | 0.19782 | -0.17803 | 0.47892 | -0.28492 | 0.70845 | #N/A | #N/A | -1.33565 | 0.75817 | -0.00979 | -0.01596 | -0.0164 | 0.98788 | 0.98732 | 0.56303 | 0.59037 | 5 | 2.93229 | 0.55262 | 0.64498 | 0.610839 | Not Significant | NS | #N/A |
| 1824 | A0A5F9DH30 | #N/A | STAG2 | RABBIT | #N/A | 0.0717 | #N/A | #N/A | #N/A | #N/A | #N/A | 0.06175 | #N/A | #N/A | -0.00995 | #N/A | -0.00946 | #N/A | 0.99306 | #N/A | 0.59221 | 0 | 2.93229 | 0.55262 | #N/A | 0.552617 | Not Significant | NS | #N/A |
| 1678 | A0A5F9DSK6 | #N/A | VCAN | RABBIT | #N/A | -0.90043 | 0.92368 | #N/A | #N/A | #N/A | #N/A | #N/A | 0 | #N/A | -0.01162 | -0.00736 | -0.01038 | 0.99532 | 0.99222 | 0.56452 | 0.59209 | 1 | 2.93229 | 0.55262 | 1.66369 | 0.835169 | Not Significant | NS | #N/A |
| 2493 | G1SLQ3 | #N/A | NQO1 | RABBIT | #N/A | #N/A | #N/A | -0.05764 | #N/A | 0.4563 | -0.56708 | #N/A | 0.42112 | -0.61125 | -0.01759 | -0.02649 | -0.02341 | 0.98053 | 0.98209 | 0.56183 | 0.58933 | 3 | 2.93229 | 0.55262 | 0.35271 | 0.451521 | Not Significant | NS | #N/A |
| 207 | A0A5F9CJZ7 | F-actin-capping protein subunit beta OS=Oryctolag | CAPZB | RABBIT | -0.53131 | 0.19782 | -0.25863 | 0.50127 | 0.04018 | #N/A | 0.53137 | 0.51989 | -0.10409 | -1.05834 | -0.01766 | -0.04572 | -0.04179 | 0.96481 | 0.96749 | 0.55738 | 0.58521 | 7 | 2.93229 | 0.55262 | 0.33159 | 0.396846 | Not Significant | NS | #N/A |
| 2315 | G1SI62 | #N/A | PWP1 | RABBIT | #N/A | #N/A | 0.03473 | 0.00167 | #N/A | -0.00194 | #N/A | #N/A | 0.00194 | #N/A | -0.0182 | -1.09372 | -0.03175 | 0.38823 | 0.97592 | 0.3653 | 0.58845 | 2 | 2.93229 | 0.55262 | 0.00028 | 0.328648 | Not Significant | NS | #N/A |
| 916 | G1SMM7 | Small nuclear ribonucleoprotein Sm D3 OS=Oryctol | SNRPD3 | RABBIT | -1.24234 | -0.45726 | 0.5274 | 0.06848 | 0.15008 | -1.08278 | 0.09987 | 0.27575 | -0.24107 | -0.09987 | -0.0189 | -0.04893 | -0.04604 | 0.96218 | 0.96411 | 0.55731 | 0.58481 | 8 | 2.93229 | 0.55262 | 0.37285 | 0.421066 | Not Significant | NS | #N/A |
| 321 | A0A5F9CMB3 | S-formylglutathione hydrolase OS=Oryctolagus cun | #N/A | RABBIT | -0.3598 | #N/A | 0.39839 | #N/A | #N/A | #N/A | 0 | #N/A | #N/A | #N/A | -0.0193 | -0.02939 | -0.02262 | 0.9813 | 0.98306 | 0.56183 | 0.58933 | 1 | 2.93229 | 0.55262 | 0.28743 | 0.485178 | Not Significant | NS | #N/A |
| 466 | A0A5F9DBT3 | Phosphodiesterase OS=Oryctolagus cuniculus OX= | PDE1B | RABBIT | -0.21885 | 0.42833 | -0.31926 | #N/A | 0.21733 | 0.33983 | -0.51566 | #N/A | 0.43802 | -0.23541 | -0.02019 | -0.06974 | -0.05267 | 0.94667 | 0.95916 | 0.55229 | 0.58399 | 6 | 2.93229 | 0.55262 | 0.16769 | 0.29405 | Not Significant | NS | #N/A |
| 2632 | G1SJX4 | #N/A | RASGRF2 | RABBIT | #N/A | #N/A | #N/A | -0.05764 | #N/A | -0.3136 | #N/A | #N/A | 0.15408 | #N/A | -0.02212 | -0.05462 | -0.02724 | 0.96526 | 0.9796 | 0.55742 | 0.58931 | 1 | 2.93229 | 0.55262 | 0.10936 | 0.439895 | Not Significant | NS | #N/A |
| 507 | G1U0A2 | Visinin-like protein 1 OS=Oryctolagus cuniculus OX | VSNL1 | RABBIT | -0.9347 | -0.5659 | 0.14021 | 0.13922 | 0.01706 | 0.09665 | -2.02636 | 0.4773 | -0.06736 | 0.19768 | -0.0236 | -0.04729 | -0.04802 | 0.96344 | 0.96257 | 0.55731 | 0.58421 | 8 | 2.93229 | 0.55262 | 0.62257 | 0.603808 | Not Significant | NS | #N/A |
| 2692 | G1TBG2 | #N/A | EXOC2 | RABBIT | #N/A | #N/A | #N/A | #N/A | 0.52223 | 0 | -0.11769 | -0.59031 | #N/A | 2.70212 | -0.0237 | -0.01422 | -0.01793 | 0.98955 | 0.98628 | 0.56305 | 0.59037 | 3 | 2.93229 | 0.55262 | 2.22322 | 1.397453 | Not Significant | NS | #N/A |

| | | | | | | | | | | | | | | | | | | | | | | | | | | | | | |
|---|---|---|---|---|---|---|---|---|---|---|---|---|---|---|---|---|---|---|---|---|---|---|---|---|---|---|---|---|---|
| 2867 | A0A5F9C1R4 | #N/A | #N/A | RABBIT | #N/A | #N/A | #N/A | #N/A | 0.00566 | #N/A | #N/A | #N/A | #N/A | -0.01904 | -0.0247 | #N/A | -0.02349 | #N/A | 0.98276 | #N/A | 0.58933 | 0 | 2.93229 | 0.55262 | #N/A | 0.552617 | Not Significant | NS | #N/A |
| 2102 | A0A5F9DUX5 | #N/A | SH3BGRL3 | RABBIT | #N/A | #N/A | 0.17065 | #N/A | #N/A | #N/A | -0.63802 | #N/A | #N/A | 0.92858 | -0.02537 | -0.0187 | -0.02434 | 0.9881 | 0.98176 | 0.56303 | 0.58933 | 1 | 2.93229 | 0.55262 | 1.22713 | 0.724148 | Not Significant | NS | #N/A |
| 1427 | G1SPJ1 | Nitric oxide synthase trafficking OS=Oryctolagus cu | NOSTRIN | RABBIT | 0.38052 | -0.32659 | #N/A | #N/A | #N/A | #N/A | 0 | #N/A | #N/A | #N/A | -0.02697 | -0.04404 | -0.03192 | 0.97198 | 0.97609 | 0.55955 | 0.58845 | 1 | 2.93229 | 0.55262 | 0.25 | 0.475661 | Not Significant | NS | #N/A |
| 1470 | A0A5F9DG94 | L1 transposable element RRM domain-containing p | #N/A | RABBIT | -1.06883 | #N/A | 0.418 | -0.55973 | -0.20018 | 0.21267 | 0.25766 | #N/A | 0.18679 | -2.18148 | -0.02841 | -0.04197 | -0.045 | 0.96788 | 0.96509 | 0.55819 | 0.58511 | 6 | 2.93229 | 0.55262 | 0.91624 | 0.796867 | Not Significant | NS | #N/A |
| 1554 | A0A5F9D4C1 | Neurolysin, mitochondrial OS=Oryctolagus cunicul | #N/A | RABBIT | #N/A | 1.97745 | -1.91685 | #N/A | #N/A | #N/A | #N/A | #N/A | 0 | #N/A | -0.0303 | -0.00898 | -0.01617 | 0.99428 | 0.98788 | 0.56449 | 0.59037 | 1 | 2.93229 | 0.55262 | 7.58282 | 2.340433 | Not Significant | NS | #N/A |
| 416 | Q6Y8G4 | Drebrin 1 isoform a OS=Oryctolagus cuniculus OX= | #N/A | RABBIT | -0.11622 | -0.68442 | 0.10018 | 0.87888 | 0.53186 | 0.05465 | -0.05465 | 0.32048 | 0.53232 | -0.2996 | -0.03142 | -0.10282 | -0.08795 | 0.92063 | 0.93151 | 0.54531 | 0.57471 | 8 | 2.93229 | 0.55262 | 0.23337 | 0.319002 | Not Significant | NS | #N/A |
| 2509 | G1U001 | #N/A | RPL38 | RABBIT | #N/A | #N/A | #N/A | 0.21202 | #N/A | -0.31144 | -0.26966 | #N/A | #N/A | 1.12108 | -0.03202 | -0.03402 | -0.03586 | 0.97595 | 0.9728 | 0.5605 | 0.58729 | 2 | 2.93229 | 0.55262 | 0.66466 | 0.598051 | Not Significant | NS | #N/A |
| 1318 | A0A5F9D6S0 | Structural maintenance of chromosomes 2 OS=Ory | SMC2 | RABBIT | 0.14727 | -0.42202 | 0.17246 | #N/A | #N/A | #N/A | #N/A | -0.06618 | #N/A | #N/A | -0.03208 | -0.08263 | -0.0454 | 0.94167 | 0.96557 | 0.55054 | 0.58519 | 2 | 2.93229 | 0.55262 | 0.11302 | 0.374364 | Not Significant | NS | #N/A |
| 1790 | G1U4Y8 | #N/A | CRPPA | RABBIT | #N/A | -0.13876 | #N/A | 0.14066 | #N/A | -0.0363 | #N/A | -0.02806 | #N/A | #N/A | -0.03313 | -0.23706 | -0.05712 | 0.83468 | 0.95669 | 0.52268 | 0.58352 | 2 | 2.93229 | 0.55262 | 0.01954 | 0.336457 | Not Significant | NS | #N/A |
| 1590 | A0A5F9C1A1 | Complexin-1 OS=Oryctolagus cuniculus OX=9986 F | #N/A | RABBIT | -1.1763 | 1.2547 | -0.04383 | -0.05764 | -0.84698 | #N/A | -2.34265 | 0.4116 | 0.43115 | 0.66641 | -0.03436 | -0.04365 | -0.0481 | 0.96641 | 0.96259 | 0.55764 | 0.58421 | 7 | 2.93229 | 0.55262 | 1.37756 | 1.134011 | Not Significant | NS | #N/A |
| 1849 | G1SRZ8 | #N/A | CNOT1 | RABBIT | #N/A | #N/A | #N/A | 0.07605 | #N/A | 0.53231 | -0.8332 | -0.27194 | 0.90591 | -0.13369 | -0.03618 | -0.04815 | -0.04646 | 0.96391 | 0.96425 | 0.55731 | 0.58481 | 4 | 2.93229 | 0.55262 | 0.47053 | 0.50525 | Not Significant | NS | #N/A |
| 1714 | G1U7K9 | #N/A | #N/A | RABBIT | #N/A | 0.20244 | 0.39882 | -0.33997 | #N/A | -0.00462 | -2.01834 | 0.96151 | -0.11181 | 1.41542 | -0.03867 | -0.04802 | -0.053 | 0.96326 | 0.9589 | 0.55731 | 0.58399 | 6 | 2.93229 | 0.55262 | 1.21588 | 0.998143 | Not Significant | NS | #N/A |
| 998 | A0A5F9CU78 | Nidogen 2 OS=Oryctolagus cuniculus OX=9986 GN | NID2 | RABBIT | -0.52819 | #N/A | 0.17065 | -0.49943 | 0.65484 | -0.09587 | 0.18742 | 0.34376 | 0.31438 | -1.19899 | -0.03933 | -0.09556 | -0.08957 | 0.92654 | 0.93041 | 0.54606 | 0.57465 | 7 | 2.93229 | 0.55262 | 0.37638 | 0.42841 | Not Significant | NS | #N/A |
| 2246 | G1SXN3 | #N/A | LAMA4 | RABBIT | #N/A | #N/A | -0.39133 | #N/A | 0.5486 | #N/A | 0.67212 | #N/A | -0.59454 | #N/A | -0.03984 | -0.05052 | -0.05228 | 0.9643 | 0.96035 | 0.55731 | 0.58421 | 2 | 2.93229 | 0.55262 | 0.62197 | 0.58074 | Not Significant | NS | #N/A |
| 287 | G1TY06 | Glutathione S-transferase OS=Oryctolagus cunicul | #N/A | RABBIT | 0.14498 | 1.82444 | -1.40816 | 0.64457 | -2.54395 | 0.69287 | #N/A | -1.48437 | #N/A | -0.13332 | -0.04065 | -0.03608 | -0.04172 | 0.97239 | 0.96764 | 0.55955 | 0.58521 | 6 | 2.93229 | 0.55262 | 2.37961 | 1.779845 | Not Significant | NS | #N/A |
| 2310 | G1T155 | #N/A | SPON1 | RABBIT | #N/A | #N/A | -0.30778 | 0.2658 | #N/A | #N/A | #N/A | -0.06437 | #N/A | #N/A | -0.04338 | -0.08732 | -0.05257 | 0.94455 | 0.96064 | 0.55171 | 0.58421 | 1 | 2.93229 | 0.55262 | 0.16449 | 0.453915 | Not Significant | NS | #N/A |
| 582 | G1T4P8 | Glutaredoxin 3 OS=Oryctolagus cuniculus OX=9986 | GLRX3 | RABBIT | -0.29941 | 0.52021 | -1.19416 | 0.14907 | 0.15596 | -0.95294 | -0.3066 | -0.23371 | 0.38205 | 0.21439 | -0.04569 | -0.12145 | -0.11322 | 0.90633 | 0.91191 | 0.54298 | 0.57143 | 8 | 2.93229 | 0.55262 | 0.35391 | 0.407208 | Not Significant | NS | #N/A |
| 943 | G1TCX1 | protein-ribulosamine 3-kinase OS=Oryctolagus cun | FN3KRP | RABBIT | -1.24389 | 0.19782 | 0.16215 | #N/A | 0.89857 | -2.15057 | 0.19134 | 1.26037 | 1.23134 | -0.74458 | -0.04609 | -0.05549 | -0.0616 | 0.9573 | 0.9521 | 0.55612 | 0.58197 | 7 | 2.93229 | 0.55262 | 1.53311 | 1.243638 | Not Significant | NS | #N/A |
| 2336 | A0A5F9CQ60 | #N/A | TMEM30A | RABBIT | #N/A | #N/A | -0.18413 | #N/A | -0.01338 | 0.06409 | 0.51769 | 0.43243 | -0.94241 | -0.7988 | -0.04664 | -0.09057 | -0.08375 | 0.93135 | 0.93533 | 0.548 | 0.57601 | 5 | 2.93229 | 0.55262 | 0.37884 | 0.44308 | Not Significant | NS | #N/A |
| 1888 | G1STR7 | #N/A | SPTA1 | RABBIT | #N/A | #N/A | -1.09428 | #N/A | 1.12413 | #N/A | 0.89257 | -0.95694 | #N/A | #N/A | -0.0471 | -0.03262 | -0.04347 | 0.97694 | 0.96703 | 0.56077 | 0.58521 | 2 | 2.93229 | 0.55262 | 2.0855 | 1.174187 | Not Significant | NS | #N/A |
| 639 | G1U754 | Histidine-rich glycoprotein OS=Oryctolagus cunicul | HRG | RABBIT | -0.15694 | -1.31987 | -4.34391 | 0.13461 | 0.15522 | -5.23547 | 1.92429 | 0.10578 | 0.29152 | -2.86571 | -0.04974 | -0.03234 | -0.03718 | 0.97499 | 0.97101 | 0.56017 | 0.58662 | 8 | 2.93229 | 0.55262 | 5.91263 | 4.474956 | Not Significant | NS | #N/A |
| 129 | G1TCZ8 | Protein phosphatase 2 scaffold subunit Aalpha OS= | PPP2R1A | RABBIT | 0.0293 | -0.0028 | -0.2771 | 0.02946 | 0.06772 | 0.06902 | -0.01764 | -0.21798 | 0.16631 | -0.40588 | -0.05055 | -0.41953 | -0.19119 | 0.68587 | 0.85188 | 0.48039 | 0.55412 | 8 | 2.93229 | 0.55262 | 0.0363 | 0.174787 | Not Significant | NS | #N/A |
| 911 | A0A5F9CXT4 | Epoxide hydrolase 1 OS=Oryctolagus cuniculus OX= | EPHX1 | RABBIT | -1.45165 | #N/A | 1.45164 | -0.18186 | 1.39996 | -0.33383 | 1.68125 | 0.24128 | -0.31888 | 0 | -0.05056 | -0.06795 | -0.07425 | 0.94773 | 0.94228 | 0.55254 | 0.57823 | 7 | 2.93229 | 0.55262 | 1.23029 | 1.030221 | Not Significant | NS | #N/A |
| 1226 | G1TF09 | Mannose receptor C type 2 OS=Oryctolagus cunicu | MRC2 | RABBIT | 0.32976 | #N/A | -0.08781 | #N/A | 0.41078 | -1.04973 | 2.66214 | 0.1941 | -0.58281 | -0.39264 | -0.05137 | -0.05832 | -0.06535 | 0.95538 | 0.94933 | 0.55567 | 0.5809 | 6 | 2.93229 | 0.55262 | 1.45425 | 1.158264 | Not Significant | NS | #N/A |
| 1549 | G1SKG9 | E3 ubiquitin-protein ligase OS=Oryctolagus cunicul | HECTD1 | RABBIT | -1.4626 | 0.06092 | -0.55449 | 0.42246 | 0.18206 | -1.37015 | 0.18486 | 0.07254 | 0.41733 | -0.9259 | -0.05393 | -0.11103 | -0.11198 | 0.91433 | 0.91287 | 0.54413 | 0.57143 | 8 | 2.93229 | 0.55262 | 0.58988 | 0.579887 | Not Significant | NS | #N/A |
| 915 | A0A5F9C6Q3 | Transthyretin OS=Oryctolagus cuniculus OX=9986 ( | TTR | RABBIT | -0.76386 | 2.69762 | -3.17091 | 0.27645 | -0.0943 | -2.75009 | 2.16304 | 0.36828 | -1.19033 | 0.08092 | -0.05464 | -0.04373 | -0.04984 | 0.96619 | 0.96115 | 0.55764 | 0.58421 | 8 | 2.93229 | 0.55262 | 3.90325 | 3.004535 | Not Significant | NS | #N/A |
| 2690 | O06137 | #N/A | #N/A | MT | #N/A | #N/A | #N/A | -0.00137 | #N/A | #N/A | #N/A | #N/A | -0.05627 | #N/A | -0.0549 | #N/A | -0.05222 | #N/A | 0.96171 | #N/A | 0.58421 | 0 | 2.93229 | 0.55262 | #N/A | 0.552617 | Not Significant | NS | #N/A |
| 2585 | G1U044 | #N/A | USP54 | RABBIT | #N/A | #N/A | #N/A | -0.03339 | #N/A | #N/A | #N/A | -0.08862 | #N/A | #N/A | -0.05523 | #N/A | -0.05254 | #N/A | 0.96147 | #N/A | 0.58421 | 0 | 2.93229 | 0.55262 | #N/A | 0.552617 | Not Significant | NS | #N/A |
| 750 | G1T7M3 | TIP41-like protein OS=Oryctolagus cuniculus OX=9 | TIPRL | RABBIT | -1.23208 | 0.20437 | 0.70722 | 0.81971 | -0.10091 | 0.33176 | -0.00655 | -0.9619 | 1.13075 | -0.37347 | -0.05554 | -0.10934 | -0.1115 | 0.91563 | 0.91324 | 0.54451 | 0.57145 | 8 | 2.93229 | 0.55262 | 0.64511 | 0.620299 | Not Significant | NS | #N/A |
| 500 | G1SVG8 | Oligodendrocyte myelin glycoprotein OS=Oryctolag | OMG | RABBIT | -0.35921 | -1.47031 | 0.4289 | 0.54285 | 0.80348 | -0.91275 | -0.2256 | 0.16123 | -0.27883 | 0.91408 | -0.05751 | -0.11264 | -0.11501 | 0.91309 | 0.91052 | 0.54361 | 0.57142 | 8 | 2.93229 | 0.55262 | 0.6518 | 0.625195 | Not Significant | NS | #N/A |
| 1696 | A0A5F9D3T3 | #N/A | DLG1 | RABBIT | #N/A | 0.31268 | #N/A | -0.17249 | #N/A | -0.46985 | #N/A | #N/A | 0.49407 | #N/A | -0.05798 | -0.10745 | -0.08676 | 0.92424 | 0.93428 | 0.54558 | 0.57579 | 2 | 2.93229 | 0.55262 | 0.29113 | 0.446588 | Not Significant | NS | #N/A |
| 2838 | G1TDL1 | #N/A | MAGI3 | RABBIT | #N/A | #N/A | #N/A | #N/A | 0.02301 | -0.03639 | #N/A | #N/A | #N/A | #N/A | -0.0594 | #N/A | -0.05651 | #N/A | 0.95857 | #N/A | 0.58399 | 0 | 2.93229 | 0.55262 | #N/A | 0.552617 | Not Significant | NS | #N/A |
| 1810 | G1TUB8 | #N/A | RPL11 | RABBIT | #N/A | #N/A | 0.50223 | 0.03618 | -0.43672 | -0.28894 | -0.35455 | 0.34692 | 0.24508 | -0.09382 | -0.06295 | -0.23107 | -0.16441 | 0.82494 | 0.87307 | 0.51907 | 0.56055 | 6 | 2.93229 | 0.55262 | 0.13917 | 0.274895 | Not Significant | NS | #N/A |
| 227 | G1TXS2 | Myosin XVIIIA OS=Oryctolagus cuniculus OX=9986 | MYO18A | RABBIT | 1.15394 | -0.45968 | 0.17065 | -0.52894 | 1.41193 | -0.07438 | 1.26099 | #N/A | 0.76411 | -0.80597 | -0.06339 | -0.10439 | -0.10981 | 0.91978 | 0.91475 | 0.54531 | 0.57198 | 7 | 2.93229 | 0.55262 | 0.81939 | 0.740633 | Not Significant | NS | #N/A |
| 1095 | A0A5F9C8W6 | Fibulin 2 OS=Oryctolagus cuniculus OX=9986 GN=I | FBLN2 | RABBIT | 0.63086 | -0.23616 | -0.24359 | 0.15418 | 0.09147 | #N/A | 1.02417 | -0.16922 | #N/A | -0.81388 | -0.06566 | -0.14672 | -0.13653 | 0.88816 | 0.89443 | 0.53768 | 0.56755 | 6 | 2.93229 | 0.55262 | 0.37552 | 0.433661 | Not Significant | NS | #N/A |
| 2146 | G1TT27 | #N/A | RPL8 | RABBIT | #N/A | #N/A | -0.94602 | -0.12257 | 1.22619 | 0.18693 | -0.37156 | 0.43878 | -0.3937 | 0.06493 | -0.06746 | -0.1322 | -0.12943 | 0.89914 | 0.89989 | 0.54019 | 0.56842 | 6 | 2.93229 | 0.55262 | 0.48814 | 0.509308 | Not Significant | NS | #N/A |
| 451 | A0A5F9CXR1 | Myosin heavy chain 10 OS=Oryctolagus cuniculus ( | MYH10 | RABBIT | -0.51787 | -0.21236 | 0.65525 | -1.04083 | 0.64245 | -1.31757 | 0.04332 | 0.33853 | -0.04332 | 0.1683 | -0.06748 | -0.15193 | -0.14953 | 0.883 | 0.88386 | 0.53662 | 0.56374 | 8 | 2.93229 | 0.55262 | 0.49314 | 0.509093 | Not Significant | NS | #N/A |
| 1519 | G1T1R8 | Ectonucleoside triphosphate diphosphohydrolase 7 | ENTPD7 | RABBIT | 0.36875 | -0.50412 | -0.31458 | #N/A | 0.48992 | #N/A | 0 | -0.21408 | 0.03435 | #N/A | -0.0699 | -0.23399 | -0.1669 | 0.82427 | 0.87163 | 0.51905 | 0.56055 | 5 | 2.93229 | 0.55262 | 0.15299 | 0.300717 | Not Significant | NS | #N/A |
| 1709 | A0A5F9DBB2 | #N/A | HOOK3 | RABBIT | #N/A | 0.19782 | #N/A | #N/A | #N/A | #N/A | 0.30276 | #N/A | -0.04826 | #N/A | -0.07057 | -0.23214 | -0.0881 | 0.85479 | 0.9341 | 0.52871 | 0.57579 | 1 | 2.93229 | 0.55262 | 0.06161 | 0.42775 | Not Significant | NS | #N/A |
| 1390 | A0A5F9CCS4 | Rho guanine nucleotide exchange factor 12 OS=Ory | ARHGEF12 | RABBIT | -0.64117 | #N/A | 0.17774 | #N/A | 0.1343 | -0.58184 | 0.76954 | 0.45222 | -0.00709 | -1.53896 | -0.07152 | -0.12364 | -0.12613 | 0.90564 | 0.90242 | 0.54293 | 0.56947 | 6 | 2.93229 | 0.55262 | 0.62735 | 0.602816 | Not Significant | NS | #N/A |
| 2730 | A0A5F9DSZ7 | #N/A | #N/A | RABBIT | #N/A | #N/A | #N/A | 0.00694 | #N/A | -0.06458 | #N/A | #N/A | #N/A | #N/A | -0.07153 | #N/A | -0.06804 | #N/A | 0.95013 | #N/A | 0.58118 | 0 | 2.93229 | 0.55262 | #N/A | 0.552617 | Not Significant | NS | #N/A |
| 2779 | G1SQ70 | #N/A | A2M | RABBIT | #N/A | #N/A | #N/A | #N/A | 0.34121 | #N/A | 1.97786 | -0.41896 | #N/A | -0.75257 | -0.07243 | -0.04211 | -0.0566 | 0.97023 | 0.95708 | 0.55919 | 0.58356 | 2 | 2.93229 | 0.55262 | 2.21854 | 1.228136 | Not Significant | NS | #N/A |
| 2169 | G1U598 | #N/A | #N/A | RABBIT | #N/A | #N/A | 0.17715 | #N/A | #N/A | #N/A | #N/A | 0.35897 | -0.0065 | -0.04201 | -0.07366 | -0.2874 | -0.10806 | 0.80085 | 0.9182 | 0.51382 | 0.5731 | 2 | 2.93229 | 0.55262 | 0.04927 | 0.348514 | Not Significant | NS | #N/A |
| 483 | G1TBS1 | protein deglycase OS=Oryctolagus cuniculus OX=9 | PARK7 | RABBIT | -0.02282 | 1.59182 | -0.27328 | 0.45457 | -1.22266 | -0.40087 | -0.54028 | -0.02989 | 0.68993 | 0.43814 | -0.07412 | -0.14303 | -0.14655 | 0.88981 | 0.88616 | 0.53768 | 0.56421 | 8 | 2.93229 | 0.55262 | 0.67144 | 0.639566 | Not Significant | NS | #N/A |
| 847 | G1TTL2 | Rho GTPase activating protein 5 OS=Oryctolagus cu | ARHGAP5 | RABBIT | -0.21722 | -0.87071 | 0.17065 | 0.08427 | 0.43295 | #N/A | 0.12149 | -0.68377 | -0.191 | 0.13019 | -0.07576 | -0.24939 | -0.20361 | 0.81022 | 0.84277 | 0.51584 | 0.55164 | 7 | 2.93229 | 0.55262 | 0.20509 | 0.30769 | Not Significant | NS | #N/A |
| 1015 | G1SR99 | Secretory carrier-associated membrane protein OS | SCAMP5 | RABBIT | -1.02006 | #N/A | 0.46975 | -1.67465 | 0.71554 | 0.4297 | -1.17677 | 0.44112 | 0 | -1.96324 | -0.07648 | -0.10288 | -0.11242 | 0.92094 | 0.91273 | 0.54531 | 0.57143 | 7 | 2.93229 | 0.55262 | 1.22808 | 1.028662 | Not Significant | NS | #N/A |
| 278 | A0A5F9DM35 | Serine/threonine-protein phosphatase OS=Oryctola | #N/A | RABBIT | 0.25071 | -0.17108 | -0.27366 | 0.42817 | 0.03862 | 0.32352 | -0.24913 | -0.0628 | -0.12175 | -0.00156 | -0.07689 | -0.4769 | -0.27477 | 0.64619 | 0.78861 | 0.46795 | 0.53858 | 8 | 2.93229 | 0.55262 | 0.06499 | 0.195786 | Not Significant | NS | #N/A |
| 1517 | G1T4H3 | Protein disulfide-isomerase A6 OS=Oryctolagus cur | PDIA6 | RABBIT | 0.19281 | 0.5585 | -0.29575 | -0.02508 | -0.37652 | -1.23871 | -0.03256 | 0.01526 | 1.51631 | -0.59474 | -0.07768 | -0.15949 | -0.16097 | 0.87723 | 0.87505 | 0.53468 | 0.56083 | 8 | 2.93229 | 0.55262 | 0.59308 | 0.582225 | Not Significant | NS | #N/A |
| 1510 | A0A5F9CAE9 | protein-tyrosine-phosphatase OS=Oryctolagus cun | MTMR4 | RABBIT | 0.39748 | -1.60967 | 0.41689 | #N/A | #N/A | #N/A | #N/A | #N/A | 0 | -0.68601 | -0.0779 | -0.0861 | -0.09725 | 0.93681 | 0.92573 | 0.5491 | 0.57362 | 3 | 2.93229 | 0.55262 | 0.98243 | 0.769978 | Not Significant | NS | #N/A |
| 742 | P47789 | Myelin proteolipid protein OS=Oryctolagus cunicul | PLP1 PLP | RABBIT | -1.06338 | #N/A | 0.58204 | -0.65786 | 1.45314 | -0.15158 | 0.15158 | #N/A | #N/A | #N/A | -0.07848 | -0.09023 | -0.10033 | 0.93244 | 0.92292 | 0.548 | 0.57345 | 4 | 2.93229 | 0.55262 | 1.00881 | 0.815845 | Not Significant | NS | #N/A |
| 1614 | G1SRD2 | KAT8 regulatory NSL complex subunit 1 like OS=Ory | KANSL1L | RABBIT | 0.8904 | 0.45808 | -0.36081 | -2.07174 | #N/A | 0.53146 | #N/A | -2.08577 | -0.26026 | 0.40693 | -0.0809 | -0.09091 | -0.10252 | 0.93052 | 0.92061 | 0.54797 | 0.57341 | 6 | 2.93229 | 0.55262 | 1.58366 | 1.245192 | Not Significant | NS | #N/A |
| 2689 | A5U050 | #N/A | #N/A | MT | #N/A | #N/A | #N/A | -0.0201 | #N/A | #N/A | #N/A | -0.10191 | #N/A | #N/A | -0.08181 | #N/A | -0.07782 | #N/A | 0.94298 | #N/A | 0.57845 | 0 | 2.93229 | 0.55262 | #N/A | 0.552617 | Not Significant | NS | #N/A |
| 1987 | G1U4T3 | #N/A | AGAP2 | RABBIT | #N/A | 0.31581 | #N/A | #N/A | #N/A | -0.11798 | #N/A | -0.36047 | 1.17384 | #N/A | -0.08401 | -0.08821 | -0.09358 | 0.93775 | 0.92912 | 0.54911 | 0.57439 | 2 | 2.93229 | 0.55262 | 0.68028 | 0.604385 | Not Significant | NS | #N/A |
| 2571 | A0A6M5U9B2 | #N/A | COX2 | RABBIT | #N/A | #N/A | #N/A | -0.05764 | #N/A | #N/A | #N/A | 0.18836 | #N/A | -0.47325 | -0.08481 | -0.14801 | -0.10125 | 0.90645 | 0.92431 | 0.54298 | 0.57362 | 1 | 2.93229 | 0.55262 | 0.21886 | 0.467741 | Not Significant | NS | #N/A |
| 2652 | G1TH36 | #N/A | SH3KBP1 | RABBIT | #N/A | #N/A | #N/A | 0.01364 | #N/A | #N/A | #N/A | #N/A | #N/A | -0.07128 | -0.08493 | #N/A | -0.08078 | #N/A | 0.94081 | #N/A | 0.57754 | 0 | 2.93229 | 0.55262 | #N/A | 0.552617 | Not Significant | NS | #N/A |
| 2079 | G1T6W3 | #N/A | #N/A | RABBIT | #N/A | -1.32455 | 0.15187 | 1.463 | 0.00539 | 0.15805 | #N/A | 1.80376 | -1.46337 | -0.54413 | -0.08535 | -0.09537 | -0.10764 | 0.92713 | 0.91666 | 0.54619 | 0.57255 | 6 | 2.93229 | 0.55262 | 1.60193 | 1.257464 | Not Significant | NS | #N/A |
| 265 | G1SI98 | O-GlcNAcase OS=Oryctolagus cuniculus OX=9986 | OGA | RABBIT | 0.14472 | -0.42126 | #N/A | #N/A | -0.01338 | -0.86291 | 0.1 | #N/A | 0.32116 | -0.28662 | -0.08545 | -0.25299 | -0.19548 | 0.81035 | 0.84993 | 0.51584 | 0.55382 | 5 | 2.93229 | 0.55262 | 0.19557 | 0.327557 | Not Significant | NS | #N/A |
| 1265 | A0A5F9CDH0 | ApaG domain-containing protein OS=Oryctolagus c | #N/A | RABBIT | -0.80165 | -0.58084 | 2.35202 | 0.15833 | -0.2701 | #N/A | #N/A | 0.17115 | 0 | #N/A | -0.08597 | -0.09028 | -0.1017 | 0.93157 | 0.92152 | 0.548 | 0.57341 | 5 | 2.93229 | 0.55262 | 1.29551 | 1.020886 | Not Significant | NS | #N/A |
| 2382 | A0A5F9D8X1 | #N/A | #N/A | RABBIT | #N/A | #N/A | 0.17065 | #N/A | #N/A | 0.3964 | #N/A | #N/A | #N/A | -0.22766 | -0.08628 | -0.15965 | -0.10369 | 0.89921 | 0.92249 | 0.54019 | 0.57345 | 1 | 2.93229 | 0.55262 | 0.19473 | 0.461603 | Not Significant | NS | #N/A |
| 2075 | A0A5F9D4Y8 | #N/A | AGFG2 | RABBIT | #N/A | #N/A | 0.17694 | 0.25788 | -0.25784 | 0.31273 | -0.67529 | -0.07065 | 0.3625 | -0.0734 | -0.08782 | -0.32037 | -0.22887 | 0.75955 | 0.82412 | 0.50158 | 0.5459 | 6 | 2.93229 | 0.55262 | 0.14088 | 0.276047 | Not Significant | NS | #N/A |
| 2 | A0A5F9CJ94 | Spectrin alpha, non-erythrocytic 1 OS=Oryctolagus | SPTAN1 | RABBIT | 0.18341 | -0.8345 | 0.17065 | -0.69595 | 0.58965 | #N/A | 0.607 | -0.03911 | -0.57012 | -0.8343 | -0.09179 | -0.21876 | -0.20654 | 0.83308 | 0.84054 | 0.52235 | 0.55111 | 7 | 2.93229 | 0.55262 | 0.39121 | 0.438861 | Not Significant | NS | #N/A |
| 1513 | G1SMQ8 | Vacuolar protein sorting-associated protein 18 hom | VPS18 | RABBIT | #N/A | #N/A | -0.05262 | #N/A | #N/A | 0.73966 | -1.62101 | 1.13637 | 0.22328 | -1.21172 | -0.09406 | -0.07084 | -0.08257 | 0.94692 | 0.93653 | 0.55229 | 0.57605 | 4 | 2.93229 | 0.55262 | 1.46916 | 1.081472 | Not Significant | NS | #N/A |
| 1707 | A0A5F9C8Z5 | #N/A | #N/A | RABBIT | #N/A | #N/A | 0.18403 | 0.26945 | -0.28964 | 0.28905 | -0.01338 | -0.3978 | #N/A | #N/A | -0.09532 | -0.36096 | -0.21526 | 0.73639 | 0.83576 | 0.49437 | 0.54964 | 4 | 2.93229 | 0.55262 | 0.1046 | 0.294108 | Not Significant | NS | #N/A |
| 9 | G1SDU5 | Microtubule actin crosslinking factor 1 OS=Oryctola | MACF1 | RABBIT | 0.12944 | -0.78764 | 0.35732 | -0.51455 | 0.82148 | #N/A | 0.17283 | -0.18214 | -0.27545 | #N/A | -0.09613 | -0.23924 | -0.21221 | 0.81888 | 0.83671 | 0.51745 | 0.54964 | 6 | 2.93229 | 0.55262 | 0.30271 | 0.384752 | Not Significant | NS | #N/A |
| 1246 | G1SJ71 | Zyg-11 family member B, cell cycle regulator OS=Or | ZYG11B | RABBIT | 1.01027 | #N/A | -1.22229 | 1.35731 | -0.87449 | 0.1502 | 0.1089 | #N/A | -0.26395 | -0.1089 | -0.09614 | -0.14582 | -0.15541 | 0.88884 | 0.87995 | 0.53768 | 0.56253 | 6 | 2.93229 | 0.55262 | 0.86934 | 0.765369 | Not Significant | NS | #N/A |
| 2297 | A0A5F9DI74 | #N/A | #N/A | RABBIT | #N/A | #N/A | 0.18718 | -0.37316 | #N/A | -0.01653 | 0.05062 | #N/A | -0.93317 | 0.1389 | -0.09705 | -0.23554 | -0.18565 | 0.82535 | 0.85803 | 0.51907 | 0.55561 | 4 | 2.93229 | 0.55262 | 0.22636 | 0.364365 | Not Significant | NS | #N/A |
| 54 | B8K174 | Hemoglobin, beta (Predicted) OS=Oryctolagus cuni | HBB | RABBIT | -3.78178 | -0.19806 | 0.56653 | -3.02804 | 0.79998 | 1.03215 | -2.15693 | 0.58306 | -6.10802 | 0.51541 | -0.09859 | -0.05988 | -0.06898 | 0.95372 | 0.94625 | 0.55492 | 0.57963 | 8 | 2.93229 | 0.55262 | 6.77641 | 5.107048 | Not Significant | NS | #N/A |
| 151 | G1SMY6 | Exportin 1 OS=Oryctolagus cuniculus OX=9986 GN= | XPO1 | RABBIT | 0.0155 | 0.02527 | -0.20404 | 0.6181 | 0.11448 | -0.73502 | -0.05478 | 0.17022 | 0.69744 | -0.00384 | -0.09906 | -0.37041 | -0.29649 | 0.72069 | 0.77241 | 0.49014 | 0.53484 | 8 | 2.93229 | 0.55262 | 0.1788 | 0.279068 | Not Significant | NS | #N/A |
| 1300 | A0A5F9C508 | FRA10A associated CGG repeat 1 OS=Oryctolagus ( | FRA10AC1 | RABBIT | 0.1092 | #N/A | 0.07311 | #N/A | #N/A | -0.14233 | #N/A | #N/A | 0.1247 | #N/A | -0.09997 | -0.74204 | -0.1725 | 0.53537 | 0.8699 | 0.42838 | 0.55987 | 2 | 2.93229 | 0.55262 | 0.01815 | 0.335896 | Not Significant | NS | #N/A |
| 2227 | A0A5F9DGH8 | #N/A | #N/A | RABBIT | #N/A | #N/A | -0.17544 | #N/A | 0.3327 | 0.38567 | #N/A | #N/A | #N/A | -0.43226 | -0.10193 | -0.2117 | -0.15681 | 0.85195 | 0.88161 | 0.52779 | 0.56282 | 2 | 2.93229 | 0.55262 | 0.23181 | 0.422531 | Not Significant | NS | #N/A |
| 2105 | G1T2N4 | #N/A | U2SURP | RABBIT | #N/A | 0.76932 | 0.07735 | #N/A | -0.01661 | #N/A | 0 | 0.05859 | -0.01809 | 0.6576 | -0.10216 | -0.36195 | -0.24823 | 0.73218 | 0.81027 | 0.49265 | 0.54212 | 5 | 2.93229 | 0.55262 | 0.13656 | 0.290362 | Not Significant | NS | #N/A |
| 1902 | A0A5F9D014 | #N/A | #N/A | RABBIT | #N/A | #N/A | 0.13662 | #N/A | #N/A | #N/A | 0.03403 | #N/A | #N/A | #N/A | -0.10259 | #N/A | -0.09759 | #N/A | 0.92855 | #N/A | 0.57439 | 0 | 2.93229 | 0.55262 | #N/A | 0.552617 | Not Significant | NS | #N/A |
| 2003 | O19132 | #N/A | NOS1 | RABBIT | #N/A | -0.29302 | #N/A | #N/A | #N/A | 0.49084 | -2.32085 | #N/A | #N/A | 0.63943 | -0.10384 | -0.05392 | -0.07452 | 0.9619 | 0.94353 | 0.55731 | 0.57858 | 2 | 2.93229 | 0.55262 | 2.78182 | 1.456539 | Not Significant | NS | #N/A |
| 513 | G1TAI4 | Phosphatidylinositol-5-phosphate 4-kinase type 2 b | PIP4K2B | RABBIT | -0.11955 | 0.32904 | -0.23336 | 0.78969 | -0.47233 | -1.0599 | -0.49936 | 0.08198 | 1.00624 | 0.23791 | -0.10532 | -0.2538 | -0.24467 | 0.80605 | 0.81124 | 0.5152 | 0.54212 | 8 | 2.93229 | 0.55262 | 0.43053 | 0.463274 | Not Significant | NS | #N/A |
| 1525 | G1SQN6 | BCL2 interacting protein 2 OS=Oryctolagus cunicul | BNIP2 | RABBIT | -0.04684 | -0.105 | #N/A | 0.06117 | #N/A | 0.0585 | #N/A | 0.33662 | -0.80572 | #N/A | -0.10664 | -0.307 | -0.22459 | 0.77416 | 0.82877 | 0.50625 | 0.548 | 4 | 2.93229 | 0.55262 | 0.18099 | 0.338182 | Not Significant | NS | #N/A |
| 2462 | Q28735 | #N/A | TMED10 TI | RABBIT | #N/A | #N/A | 0.10679 | #N/A | #N/A | #N/A | #N/A | -0.0005 | #N/A | #N/A | -0.10729 | #N/A | -0.10206 | #N/A | 0.92529 | #N/A | 0.57362 | 0 | 2.93229 | 0.55262 | #N/A | 0.552617 | Not Significant | NS | #N/A |
| 2549 | A0A5F9CKE8 | #N/A | DDX19A | RABBIT | #N/A | #N/A | #N/A | 0.02484 | #N/A | -0.08248 | #N/A | #N/A | #N/A | #N/A | -0.10732 | #N/A | -0.10208 | #N/A | 0.92527 | #N/A | 0.57362 | 0 | 2.93229 | 0.55262 | #N/A | 0.552617 | Not Significant | NS | #N/A |
| 846 | G1SFE9 | Activator of HSP90 ATPase activity 1 OS=Oryctolagu | #N/A | RABBIT | -1.54965 | 2.6868 | -0.91315 | 2.40781 | -1.22147 | 0.7909 | -0.31181 | 0.24744 | -0.43072 | 0.56991 | -0.10892 | -0.11328 | -0.12698 | 0.9126 | 0.90126 | 0.54361 | 0.56895 | 8 | 2.93229 | 0.55262 | 2.31128 | 1.839569 | Not Significant | NS | #N/A |
| 452 | A0A5F9C2T0 | CAP-Gly domain containing linker protein 1 OS=Ory | CLIP1 | RABBIT | -0.23784 | -2.48961 | 0.50027 | -0.35458 | 0.88909 | -3.01013 | 0.87632 | 0.645 | -0.9996 | 0.2495 | -0.10925 | -0.11797 | -0.13182 | 0.909 | 0.89752 | 0.54361 | 0.56784 | 8 | 2.93229 | 0.55262 | 2.14398 | 1.717143 | Not Significant | NS | #N/A |
| 2389 | A0A5F9DDI7 | #N/A | ARFIP2 | RABBIT | #N/A | #N/A | #N/A | -0.14096 | #N/A | 0.22067 | #N/A | 0.23069 | -1.45491 | 0 | -0.10993 | -0.12143 | -0.12644 | 0.91103 | 0.90356 | 0.54361 | 0.56972 | 3 | 2.93229 | 0.55262 | 0.65563 | 0.604713 | Not Significant | NS | #N/A |
| 484 | G1SYD3 | Tyrosine--tRNA ligase OS=Oryctolagus cuniculus O) | YARS1 | RABBIT | -0.30373 | -0.60823 | 2.88387 | 0.52032 | 0.302 | -0.61524 | -0.96385 | -0.59536 | 2.06591 | 2.34963 | -0.11063 | -0.11653 | -0.13049 | 0.9101 | 0.89855 | 0.54361 | 0.56826 | 8 | 2.93229 | 0.55262 | 2.25319 | 1.797054 | Not Significant | NS | #N/A |
| 2456 | G1SWW4 | #N/A | SYNPO | RABBIT | #N/A | #N/A | 0.17065 | #N/A | #N/A | #N/A | #N/A | -0.14525 | #N/A | 0.26261 | -0.11197 | -0.317 | -0.1389 | 0.80457 | 0.89635 | 0.51471 | 0.56784 | 1 | 2.93229 | 0.55262 | 0.08318 | 0.433236 | Not Significant | NS | #N/A |
| 2859 | A0A5F9CU31 | #N/A | #N/A | RABBIT | #N/A | #N/A | #N/A | #N/A | 0.01735 | #N/A | #N/A | -0.0951 | #N/A | #N/A | -0.11244 | #N/A | -0.10696 | #N/A | 0.92172 | #N/A | 0.57341 | 0 | 2.93229 | 0.55262 | #N/A | 0.552617 | Not Significant | NS | #N/A |
| 2654 | G1TK30 | #N/A | CSAD | RABBIT | #N/A | #N/A | #N/A | -0.05764 | #N/A | #N/A | -1.78364 | #N/A | 1.4421 | #N/A | -0.11314 | -0.0405 | -0.07013 | 0.97423 | 0.94751 | 0.56016 | 0.58017 | 1 | 2.93229 | 0.55262 | 5.2027 | 1.735157 | Not Significant | NS | #N/A |
| 2465 | A0A5F9CAK3 | #N/A | CRIP3 | RABBIT | #N/A | #N/A | 0.0203 | 0.20601 | #N/A | #N/A | #N/A | #N/A | 0 | #N/A | -0.11315 | -0.70355 | -0.14316 | 0.60969 | 0.8932 | 0.45404 | 0.56699 | 1 | 2.93229 | 0.55262 | 0.01724 | 0.416469 | Not Significant | NS | #N/A |
| 2751 | A0A5F9CXS0 | #N/A | RGMB | RABBIT | #N/A | #N/A | #N/A | -0.26171 | 0.19068 | #N/A | -0.51404 | #N/A | #N/A | 0.21383 | -0.1146 | -0.26744 | -0.18052 | 0.81419 | 0.86393 | 0.51693 | 0.5577 | 2 | 2.93229 | 0.55262 | 0.18361 | 0.402989 | Not Significant | NS | #N/A |
| 1229 | U3KMK1 | Vesicular inhibitory amino acid transporter OS=Ory | SLC32A1 | RABBIT | #N/A | -1.45227 | 0.52048 | #N/A | -0.01338 | 0.36099 | #N/A | -1.22033 | #N/A | #N/A | -0.11462 | -0.11912 | -0.13741 | 0.91271 | 0.89525 | 0.54361 | 0.56766 | 3 | 2.93229 | 0.55262 | 1.11089 | 0.83494 | Not Significant | NS | #N/A |
| 2422 | G1SG02 | #N/A | ZFHX4 | RABBIT | #N/A | #N/A | 0.14308 | #N/A | #N/A | #N/A | #N/A | #N/A | 0.02757 | #N/A | -0.11551 | #N/A | -0.10987 | #N/A | 0.9196 | #N/A | 0.57327 | 0 | 2.93229 | 0.55262 | #N/A | 0.552617 | Not Significant | NS | #N/A |
| 1235 | B7NZQ5 | ATPase, H+ transporting, lysosomal accessory prot | ATP6AP1 | RABBIT | -1.39631 | #N/A | 0.41249 | #N/A | 0.5966 | -0.15308 | 0.15308 | 0.96107 | -0.25322 | -1.93294 | -0.11595 | -0.14809 | -0.16259 | 0.88712 | 0.87446 | 0.53768 | 0.56083 | 6 | 2.93229 | 0.55262 | 1.14942 | 0.953501 | Not Significant | NS | #N/A |
| 1142 | G1TYY2 | Phosphorylase b kinase regulatory subunit OS=Ory | PHKA2 | RABBIT | -1.40042 | -1.26053 | 3.8851 | #N/A | 0.55821 | #N/A | #N/A | -0.63596 | 1.28731 | #N/A | -0.11991 | -0.06191 | -0.07838 | 0.95361 | 0.93974 | 0.55492 | 0.57729 | 4 | 2.93229 | 0.55262 | 5.00276 | 3.120393 | Not Significant | NS | #N/A |
| 1170 | G1SM50 | Splicing factor 3b subunit 4 OS=Oryctolagus cunicu | SF3B4 | RABBIT | #N/A | -0.9125 | #N/A | 0.99252 | 0.1774 | #N/A | -0.16373 | -0.06437 | -0.72629 | 0.81469 | -0.12073 | -0.20257 | -0.20613 | 0.84745 | 0.84188 | 0.52595 | 0.55137 | 5 | 2.93229 | 0.55262 | 0.60889 | 0.588087 | Not Significant | NS | #N/A |

| | | | | | | | | | | | | | | | | | | | | | | | | | | | | | |
|---|---|---|---|---|---|---|---|---|---|---|---|---|---|---|---|---|---|---|---|---|---|---|---|---|---|---|---|---|---|
| 2424 | B7NZI9 | #N/A | ERGIC3 | RABBIT | #N/A | #N/A | 0.14585 | #N/A | #N/A | 0.0248 | #N/A | #N/A | #N/A | #N/A | -0.12104 | #N/A | -0.11514 | #N/A | 0.91577 | #N/A | 0.5722 | 0 | 2.93229 | 0.55262 | #N/A | 0.552617 | Not Significant | NS | #N/A |
| 458 | G1SDC1 | Actin related protein 3B OS=Oryctolagus cuniculus | #N/A | RABBIT | -0.68166 | -0.0963 | -1.40377 | 0.54264 | 0.16042 | -2.69774 | 0.32551 | 0.06026 | 0.34911 | -0.12462 | -0.12176 | -0.18171 | -0.19551 | 0.86033 | 0.84858 | 0.52967 | 0.55382 | 8 | 2.93229 | 0.55262 | 1.12258 | 0.969705 | Not Significant | NS | #N/A |
| 667 | G1SH42 | Striatin-interacting protein 1 OS=Oryctolagus cunic | STRIP1 | RABBIT | 0.0075 | -1.42639 | -0.05205 | 1.31875 | 0.89829 | #N/A | 1.03407 | 0.37225 | 0 | -1.29796 | -0.12213 | -0.17753 | -0.19145 | 0.86412 | 0.85203 | 0.53089 | 0.55412 | 7 | 2.93229 | 0.55262 | 1.05167 | 0.904334 | Not Significant | NS | #N/A |
| 2711 | A0A5F9DNE4 | #N/A | PRKAG2 | RABBIT | #N/A | #N/A | #N/A | -0.1753 | #N/A | 0.11766 | #N/A | 0.10038 | -1.11189 | #N/A | -0.12265 | -0.15068 | -0.1459 | 0.89405 | 0.88977 | 0.53886 | 0.56544 | 2 | 2.93229 | 0.55262 | 0.49695 | 0.530044 | Not Significant | NS | #N/A |
| 1998 | G1SUV7 | #N/A | #N/A | RABBIT | #N/A | -0.19895 | #N/A | 0.21298 | 0.24642 | #N/A | 0.04012 | -0.10449 | #N/A | -0.04634 | -0.12372 | -0.82898 | -0.30123 | 0.45372 | 0.77207 | 0.39325 | 0.53484 | 4 | 2.93229 | 0.55262 | 0.03341 | 0.253029 | Not Significant | NS | #N/A |
| 2037 | G1TGY9 | #N/A | VPS11 | RABBIT | #N/A | 0.16185 | #N/A | #N/A | #N/A | 0.03597 | #N/A | #N/A | #N/A | #N/A | -0.12588 | #N/A | -0.11974 | #N/A | 0.91242 | #N/A | 0.57143 | 0 | 2.93229 | 0.55262 | #N/A | 0.552617 | Not Significant | NS | #N/A |
| 2706 | A0A5F9D3X8 | #N/A | DCLRE1A | RABBIT | #N/A | #N/A | #N/A | -0.05764 | #N/A | #N/A | -0.77737 | #N/A | #N/A | 0.41001 | -0.12604 | -0.12257 | -0.13383 | 0.92235 | 0.90011 | 0.54531 | 0.56842 | 1 | 2.93229 | 0.55262 | 0.70494 | 0.591352 | Not Significant | NS | #N/A |
| 604 | G1SMI5 | Abraxas 2, BRISC complex subunit OS=Oryctolagus | ABRAXAS2 | RABBIT | 0.1433 | -0.14044 | 0.22971 | 0.5369 | -0.07849 | -0.41444 | 0.4542 | -0.12343 | 0.67577 | -0.54333 | -0.12844 | -0.47924 | -0.38405 | 0.6446 | 0.70831 | 0.46729 | 0.51642 | 8 | 2.93229 | 0.55262 | 0.17958 | 0.279638 | Not Significant | NS | #N/A |
| 150 | G1T432 | Adenylyl cyclase-associated protein OS=Oryctolagu | #N/A | RABBIT | 0.19108 | 0.53387 | -0.34626 | 0.24406 | -0.2491 | -0.53122 | 0.70269 | -0.12636 | -0.37603 | 0.06199 | -0.12851 | -0.47601 | -0.38293 | 0.6468 | 0.70911 | 0.46795 | 0.51642 | 8 | 2.93229 | 0.55262 | 0.18223 | 0.281576 | Not Significant | NS | #N/A |
| 1216 | G1SYQ9 | RalA binding protein 1 OS=Oryctolagus cuniculus O | RALBP1 | RABBIT | -0.14524 | -0.78289 | 0.32755 | 0.47155 | 0.64218 | -0.18345 | -0.2676 | 0.99098 | 0.5365 | -1.20852 | -0.12905 | -0.28311 | -0.28072 | 0.78428 | 0.78417 | 0.50854 | 0.53801 | 8 | 2.93229 | 0.55262 | 0.51945 | 0.528343 | Not Significant | NS | #N/A |
| 1396 | A0A5F9DAD4 | WD repeat domain phosphoinositide-interacting pr | WDR45 | RABBIT | #N/A | -1.15292 | -0.34524 | 0.65233 | 0.88654 | -0.7557 | -1.42765 | 0.45152 | 1.24407 | #N/A | -0.13212 | -0.17347 | -0.19063 | 0.86799 | 0.85308 | 0.53214 | 0.55425 | 6 | 2.93229 | 0.55262 | 1.16014 | 0.960704 | Not Significant | NS | #N/A |
| 612 | G1T501 | Cell cycle and apoptosis regulator 2 OS=Oryctolagu | CCAR2 | RABBIT | -0.65054 | -0.95922 | 0.70286 | 0.24451 | 0.81046 | -1.17379 | -0.30215 | -0.70411 | 0.70564 | 0.96155 | -0.13219 | -0.2437 | -0.25226 | 0.8136 | 0.80552 | 0.51678 | 0.54212 | 8 | 2.93229 | 0.55262 | 0.73554 | 0.686474 | Not Significant | NS | #N/A |
| 1673 | U3KNN0 | #N/A | COPS2 | RABBIT | #N/A | #N/A | #N/A | -0.45911 | 1.17184 | #N/A | #N/A | -0.10304 | 0.77393 | 0 | -0.13273 | -0.18826 | -0.19178 | 0.86269 | 0.85431 | 0.53045 | 0.55474 | 3 | 2.93229 | 0.55262 | 0.59652 | 0.574817 | Not Significant | NS | #N/A |
| 2430 | G1U8Y7 | #N/A | SRCAP | RABBIT | #N/A | #N/A | #N/A | #N/A | -0.01338 | #N/A | #N/A | #N/A | -0.55318 | 0.25954 | -0.13344 | -0.18958 | -0.15469 | 0.88072 | 0.88468 | 0.53613 | 0.56387 | 1 | 2.93229 | 0.55262 | 0.33026 | 0.496071 | Not Significant | NS | #N/A |
| 2650 | A0A5F9D4E7 | #N/A | #N/A | RABBIT | #N/A | #N/A | #N/A | -0.68744 | 0.90336 | #N/A | -0.18315 | 0.08089 | -0.0772 | 0.0772 | -0.13353 | -0.26894 | -0.23696 | 0.80128 | 0.81954 | 0.51387 | 0.54399 | 4 | 2.93229 | 0.55262 | 0.32868 | 0.423402 | Not Significant | NS | #N/A |
| 2844 | G1SCG4 | #N/A | NUGGC | RABBIT | #N/A | #N/A | #N/A | #N/A | 0.06155 | #N/A | #N/A | #N/A | #N/A | -0.07493 | -0.13648 | #N/A | -0.12982 | #N/A | 0.9051 | #N/A | 0.56976 | 0 | 2.93229 | 0.55262 | #N/A | 0.552617 | Not Significant | NS | #N/A |
| 2565 | A0A5F9D3F9 | #N/A | COMMD2 | RABBIT | #N/A | #N/A | #N/A | -0.05764 | #N/A | #N/A | -0.78053 | -1.00885 | 0.45394 | 0.55887 | -0.1365 | -0.14973 | -0.1564 | 0.89048 | 0.8809 | 0.53768 | 0.56282 | 3 | 2.93229 | 0.55262 | 0.66488 | 0.609388 | Not Significant | NS | #N/A |
| 926 | G1SIT6 | Actin-related protein 2/3 complex subunit 5 OS=Ory | ARPC5L | RABBIT | -0.18021 | -0.53094 | 0.23473 | -0.12171 | 0.32702 | -0.58618 | 0.07196 | 0.09083 | 0.46878 | -1.00239 | -0.13718 | -0.44802 | -0.38358 | 0.66602 | 0.70864 | 0.47235 | 0.51642 | 8 | 2.93229 | 0.55262 | 0.23437 | 0.319729 | Not Significant | NS | #N/A |
| 1646 | P9WIL9 | Protein PafC OS=Mycobacterium tuberculosis (strai | #N/A | MT | -0.2138 | 0.42328 | #N/A | 0.17698 | #N/A | -0.47837 | 1.01362 | -0.36877 | 0.28085 | -0.50362 | -0.14008 | -0.33815 | -0.30422 | 0.74677 | 0.76793 | 0.49801 | 0.53416 | 6 | 2.93229 | 0.55262 | 0.32176 | 0.397543 | Not Significant | NS | #N/A |
| 1320 | A0A5F9DC21 | PHD finger protein 5A OS=Oryctolagus cuniculus O | PHF5A | RABBIT | -0.25356 | #N/A | 0.43893 | -0.95865 | 0.50751 | #N/A | 0.26522 | 0.28828 | -0.69941 | -0.68272 | -0.14071 | -0.31778 | -0.29837 | 0.76142 | 0.77225 | 0.50173 | 0.53484 | 6 | 2.93229 | 0.55262 | 0.39216 | 0.444833 | Not Significant | NS | #N/A |
| 1085 | G1TSJ3 | Cleavage and polyadenylation specificity factor sub | #N/A | RABBIT | 0.01166 | #N/A | -0.09942 | #N/A | 0.4978 | #N/A | -0.15021 | 0.14162 | #N/A | #N/A | -0.14097 | -0.54109 | -0.27544 | 0.62604 | 0.79232 | 0.45953 | 0.53886 | 3 | 2.93229 | 0.55262 | 0.08145 | 0.314346 | Not Significant | NS | #N/A |
| 357 | A0A5F9C0T9 | Phosphoribosyl pyrophosphate synthase-associate | PRPSAP2 | RABBIT | 0.31101 | -0.37846 | 0.43416 | -1.28055 | 1.19111 | #N/A | -0.19754 | 0.25589 | -0.40455 | 0 | -0.14201 | -0.29079 | -0.28897 | 0.77964 | 0.77854 | 0.5073 | 0.53632 | 7 | 2.93229 | 0.55262 | 0.52998 | 0.536661 | Not Significant | NS | #N/A |
| 738 | U3KPB1 | Protein phosphatase 1H OS=Oryctolagus cuniculus | PPM1H | RABBIT | 0.16827 | 0.69067 | -1.84407 | 0.32438 | -0.17 | -2.90217 | -0.36648 | 1.57839 | 0.33395 | -0.18879 | -0.14287 | -0.16711 | -0.18535 | 0.87143 | 0.85635 | 0.53314 | 0.55531 | 8 | 2.93229 | 0.55262 | 1.82749 | 1.485543 | Not Significant | NS | #N/A |
| 373 | G1SN05 | Eukaryotic initiation factor 4A-II OS=Oryctolagus cu | #N/A | RABBIT | 0.76607 | -0.04879 | -0.38245 | 0.41991 | -0.24511 | -1.45105 | -0.0956 | 0.03123 | 0.58917 | 0.72047 | -0.14308 | -0.32429 | -0.31855 | 0.75404 | 0.75607 | 0.50058 | 0.53149 | 8 | 2.93229 | 0.55262 | 0.48667 | 0.50436 | Not Significant | NS | #N/A |
| 360 | G1T868 | Clathrin light chain OS=Oryctolagus cuniculus OX=! | CLTB | RABBIT | 0.58232 | 0.28369 | -1.87485 | -0.14351 | 2.06885 | -0.14375 | 0.69149 | 1.06893 | -0.1605 | -1.26709 | -0.14549 | -0.19327 | -0.21133 | 0.85156 | 0.83652 | 0.52779 | 0.54964 | 8 | 2.93229 | 0.55262 | 1.41662 | 1.184874 | Not Significant | NS | #N/A |
| 2177 | G1TN62 | #N/A | RPS19 | RABBIT | #N/A | #N/A | 2.01101 | 0.06191 | -0.3233 | -0.2916 | -0.11955 | 0.58934 | -0.57676 | 2.57065 | -0.14879 | -0.16127 | -0.18198 | 0.87717 | 0.85966 | 0.53468 | 0.55599 | 6 | 2.93229 | 0.55262 | 1.59602 | 1.253491 | Not Significant | NS | #N/A |
| 1890 | G1SFG7 | #N/A | NES | RABBIT | #N/A | 0.52146 | -1.26628 | -0.26266 | 0.85185 | 0.38497 | 0 | -1.57754 | -0.38918 | 0.64275 | -0.14889 | -0.2462 | -0.25873 | 0.81259 | 0.80113 | 0.51659 | 0.5404 | 7 | 2.93229 | 0.55262 | 0.81272 | 0.735932 | Not Significant | NS | #N/A |
| 1469 | A0A5F9DC47 | Small nuclear ribonucleoprotein E OS=Oryctolagus | #N/A | RABBIT | -1.06936 | -0.39084 | 0.22124 | 0.36422 | 0.39983 | -1.241 | -0.00711 | 0.43849 | -0.41726 | 0.00711 | -0.14897 | -0.37233 | -0.35466 | 0.71932 | 0.72959 | 0.49014 | 0.5223 | 8 | 2.93229 | 0.55262 | 0.40021 | 0.441085 | Not Significant | NS | #N/A |
| 619 | G1T107 | E2 ubiquitin-conjugating enzyme OS=Oryctolagus c | #N/A | RABBIT | 0.01652 | 0.98534 | 0.48683 | -0.0625 | -0.3163 | -0.11716 | 0.54422 | 0.23492 | -0.27136 | -0.02686 | -0.14923 | -0.5488 | -0.44315 | 0.59812 | 0.6663 | 0.45058 | 0.50105 | 8 | 2.93229 | 0.55262 | 0.18484 | 0.283488 | Not Significant | NS | #N/A |
| 2434 | G1SWN1 | #N/A | SNF8 | RABBIT | #N/A | #N/A | #N/A | #N/A | 0.09743 | 0 | #N/A | #N/A | -0.10544 | #N/A | -0.15015 | -1.64438 | -0.19066 | 0.34783 | 0.85823 | 0.34597 | 0.55561 | 1 | 2.93229 | 0.55262 | 0.00556 | 0.413497 | Not Significant | NS | #N/A |
| 1894 | A0A5F9D9N7 | #N/A | TMEM106/ | RABBIT | #N/A | 0.17405 | #N/A | #N/A | #N/A | 0.02377 | #N/A | #N/A | #N/A | #N/A | -0.15028 | #N/A | -0.14294 | #N/A | 0.89559 | #N/A | 0.56766 | 0 | 2.93229 | 0.55262 | #N/A | 0.552617 | Not Significant | NS | #N/A |
| 1943 | A0A5F9DNR3 | #N/A | #N/A | RABBIT | #N/A | #N/A | #N/A | 0.26693 | -0.01338 | 0.5755 | -0.48804 | -0.40299 | 0.62441 | -0.4288 | -0.15076 | -0.34781 | -0.29485 | 0.74215 | 0.77568 | 0.49664 | 0.53605 | 5 | 2.93229 | 0.55262 | 0.26839 | 0.37346 | Not Significant | NS | #N/A |
| 2182 | G1TG85 | #N/A | ANK1 | RABBIT | #N/A | #N/A | 0.79007 | #N/A | -0.31798 | -0.05563 | #N/A | 0.29357 | #N/A | 0 | -0.15673 | -0.35949 | -0.27545 | 0.74305 | 0.79231 | 0.49702 | 0.53886 | 3 | 2.93229 | 0.55262 | 0.2281 | 0.388504 | Not Significant | NS | #N/A |
| 2052 | G1SV10 | #N/A | WASL | RABBIT | #N/A | 0.2195 | 0.38044 | -0.05764 | #N/A | -0.05483 | -0.1169 | 0.28691 | -0.01924 | #N/A | -0.15678 | -1.03874 | -0.42905 | 0.34653 | 0.6793 | 0.34582 | 0.50683 | 5 | 2.93229 | 0.55262 | 0.03905 | 0.2289 | Not Significant | NS | #N/A |
| 1631 | A0A5F9C0R6 | Nucleoporin 155 OS=Oryctolagus cuniculus OX=99 | NUP155 | RABBIT | 0.05204 | #N/A | #N/A | #N/A | #N/A | #N/A | #N/A | -0.10475 | #N/A | #N/A | -0.15679 | #N/A | -0.14914 | #N/A | 0.89111 | #N/A | 0.56587 | 0 | 2.93229 | 0.55262 | #N/A | 0.552617 | Not Significant | NS | #N/A |
| 1708 | G1SY56 | #N/A | PHYHIP | RABBIT | #N/A | -0.01479 | 2.56879 | #N/A | -1.44246 | #N/A | 0.21261 | #N/A | #N/A | #N/A | -0.1579 | -0.06726 | -0.09658 | 0.9525 | 0.92686 | 0.55487 | 0.57411 | 2 | 2.93229 | 0.55262 | 4.13389 | 2.004791 | Not Significant | NS | #N/A |
| 1398 | G1SPF1 | Ribosomal RNA processing 12 homolog OS=Orycto | RRP12 | RABBIT | #N/A | #N/A | #N/A | -0.20882 | 0.13779 | #N/A | #N/A | 0.16627 | -0.55419 | #N/A | -0.15845 | -0.39637 | -0.25264 | 0.73012 | 0.81073 | 0.49252 | 0.54212 | 2 | 2.93229 | 0.55262 | 0.1598 | 0.393333 | Not Significant | NS | #N/A |
| 168 | G1SPH6 | Phosphoglucomutase 2 like 1 OS=Oryctolagus cuni | PGM2L1 | RABBIT | 0.59472 | 0.06229 | -0.41869 | 0.88352 | 0.12214 | 0.57236 | -0.3363 | -0.4966 | 0.85399 | -0.14362 | -0.15883 | -0.45702 | -0.41332 | 0.65981 | 0.68737 | 0.47104 | 0.50942 | 8 | 2.93229 | 0.55262 | 0.30196 | 0.36919 | Not Significant | NS | #N/A |
| 1605 | G1T4U3 | Dual specificity phosphatase 19 OS=Oryctolagus cu | DUSP19 | RABBIT | 0.98008 | #N/A | #N/A | -0.66209 | #N/A | #N/A | #N/A | #N/A | 0.15812 | -0.15812 | -0.159 | -0.19015 | -0.20323 | 0.86674 | 0.84707 | 0.53183 | 0.55327 | 2 | 2.93229 | 0.55262 | 0.69918 | 0.612046 | Not Significant | NS | #N/A |
| 956 | G1U118 | G3BP stress granule assembly factor 1 OS=Oryctola | #N/A | RABBIT | 0.24382 | #N/A | #N/A | 1.06319 | -0.4232 | -1.09013 | 1.99039 | -0.19286 | -0.16836 | 0.1285 | -0.1611 | -0.21611 | -0.23498 | 0.83606 | 0.81952 | 0.52288 | 0.54399 | 6 | 2.93229 | 0.55262 | 1.04192 | 0.88129 | Not Significant | NS | #N/A |
| 2101 | A5U878 | #N/A | #N/A | MT | #N/A | -0.00191 | #N/A | #N/A | #N/A | #N/A | 0.26618 | 0.13536 | #N/A | -0.89122 | -0.16131 | -0.2204 | -0.19928 | 0.84601 | 0.85 | 0.52558 | 0.55382 | 2 | 2.93229 | 0.55262 | 0.40176 | 0.491447 | Not Significant | NS | #N/A |
| 214 | G1T7Z0 | 6-phosphogluconate dehydrogenase, decarboxylat | PGD | RABBIT | 0.20211 | 0.84907 | -0.73675 | 0.26536 | -0.29677 | -0.87506 | 0.46914 | -0.35588 | -0.19044 | 0.41931 | -0.16319 | -0.44293 | -0.40974 | 0.66955 | 0.68991 | 0.47323 | 0.50986 | 8 | 2.93229 | 0.55262 | 0.33935 | 0.396554 | Not Significant | NS | #N/A |
| 2405 | P9WNS9 | #N/A | #N/A | MT | #N/A | #N/A | 0.16711 | #N/A | #N/A | 0.00354 | #N/A | #N/A | #N/A | #N/A | -0.16357 | #N/A | -0.15559 | #N/A | 0.88645 | #N/A | 0.56421 | 0 | 2.93229 | 0.55262 | #N/A | 0.552617 | Not Significant | NS | #N/A |
| 2481 | G1TAF8 | #N/A | STIP1 | RABBIT | #N/A | #N/A | #N/A | 0.07766 | #N/A | #N/A | -0.04939 | 0.0045 | -0.30448 | 0 | -0.165 | -1.00534 | -0.27691 | 0.3888 | 0.79125 | 0.36536 | 0.53886 | 3 | 2.93229 | 0.55262 | 0.02155 | 0.284052 | Not Significant | NS | #N/A |
| 1405 | A0A5F9DVP4 | Solute carrier family 35 member F5 OS=Oryctolagus | SLC35F5 | RABBIT | -0.32746 | #N/A | #N/A | 0.65839 | #N/A | #N/A | #N/A | #N/A | 0 | #N/A | -0.16546 | -0.19381 | -0.18459 | 0.87813 | 0.86268 | 0.535 | 0.55731 | 1 | 2.93229 | 0.55262 | 0.48594 | 0.535661 | Not Significant | NS | #N/A |
| 2761 | G1T5L2 | #N/A | RLBP1 | RABBIT | #N/A | #N/A | #N/A | 0.1424 | #N/A | -0.0734 | #N/A | #N/A | -0.0149 | 0.0149 | -0.16687 | -3.21683 | -0.25181 | 0.08456 | 0.81134 | 0.17453 | 0.54212 | 2 | 2.93229 | 0.55262 | 0.00202 | 0.329354 | Not Significant | NS | #N/A |
| 1420 | A0A5F9CIG2 | TERF1 interacting nuclear factor 2 OS=Oryctolagus | TINF2 | RABBIT | -1.0614 | 0.93795 | 0.17065 | 0.29995 | #N/A | #N/A | -0.08054 | #N/A | #N/A | #N/A | -0.16732 | -0.17908 | -0.1891 | 0.86929 | 0.85632 | 0.5325 | 0.55531 | 3 | 2.93229 | 0.55262 | 0.69843 | 0.626356 | Not Significant | NS | #N/A |
| 171 | G1SQ23 | Actin related protein 1B OS=Oryctolagus cuniculus | ACTR1B | RABBIT | 0.7454 | -0.0685 | -0.59341 | 0.01957 | 0.21579 | -0.67484 | 0.38429 | -0.07283 | -0.1647 | 0.00847 | -0.16769 | -0.60816 | -0.49466 | 0.55995 | 0.63063 | 0.4366 | 0.4889 | 8 | 2.93229 | 0.55262 | 0.19008 | 0.287322 | Not Significant | NS | #N/A |
| 2284 | O46373 | #N/A | SLC25A4 / | RABBIT | #N/A | #N/A | 2.00585 | -0.85652 | #N/A | -0.22398 | #N/A | 0.15961 | 3.42296 | -1.73402 | -0.16852 | -0.09114 | -0.11498 | 0.93176 | 0.91172 | 0.548 | 0.57143 | 4 | 2.93229 | 0.55262 | 4.55897 | 2.864323 | Not Significant | NS | #N/A |
| 2719 | A0A5F9CJ27 | #N/A | NPAS1 | RABBIT | #N/A | #N/A | #N/A | 0.04526 | #N/A | -0.31806 | #N/A | -0.06437 | -0.39535 | 0.28152 | -0.16932 | -0.49635 | -0.26762 | 0.65374 | 0.79805 | 0.47019 | 0.53955 | 3 | 2.93229 | 0.55262 | 0.0931 | 0.320235 | Not Significant | NS | #N/A |
| 18 | A0A5F9DKI8 | Actin, cytoplasmic 2 OS=Oryctolagus cuniculus OX | #N/A | RABBIT | -1.56196 | 1.35527 | 2.09209 | 1.85723 | -0.6645 | -0.1801 | -0.06564 | -0.40379 | 2.81081 | 0.06564 | -0.17024 | -0.18065 | -0.20216 | 0.86114 | 0.84351 | 0.52994 | 0.55173 | 8 | 2.93229 | 0.55262 | 2.22026 | 1.772963 | Not Significant | NS | #N/A |
| 1726 | A0A5F9C8A9 | #N/A | CADPS2 | RABBIT | #N/A | 0.19782 | 0.84646 | -0.06165 | #N/A | 0.22932 | #N/A | -0.45014 | -0.51544 | 1.365 | -0.17036 | -0.30226 | -0.30144 | 0.77462 | 0.77083 | 0.50633 | 0.53484 | 5 | 2.93229 | 0.55262 | 0.54457 | 0.547547 | Not Significant | NS | #N/A |
| 2884 | A0A5F9CG99 | #N/A | #N/A | RABBIT | #N/A | #N/A | #N/A | #N/A | 0.04637 | #N/A | #N/A | -0.12411 | #N/A | #N/A | -0.17048 | #N/A | -0.16216 | #N/A | 0.88171 | #N/A | 0.56282 | 0 | 2.93229 | 0.55262 | #N/A | 0.552617 | Not Significant | NS | #N/A |
| 344 | G1SGN0 | Rab3 GTPase-activating protein catalytic subunit OS | RAB3GAP1 | RABBIT | -0.033 | -0.57188 | -0.12873 | 0.65734 | 0.08702 | -0.67922 | 0.04466 | 0.92248 | 0.1235 | -1.25354 | -0.17057 | -0.40524 | -0.39241 | 0.69593 | 0.7023 | 0.4833 | 0.51495 | 8 | 2.93229 | 0.55262 | 0.44293 | 0.472352 | Not Significant | NS | #N/A |
| 1277 | G1T4G4 | Neuronal pentraxin 1 OS=Oryctolagus cuniculus OX | NPTX1 | RABBIT | -0.76857 | 0.18926 | 0.42498 | 1.29476 | 0.35805 | #N/A | -0.06021 | 0.73396 | 0 | -0.16055 | -0.17139 | -0.41409 | -0.38897 | 0.6912 | 0.7055 | 0.48172 | 0.51574 | 7 | 2.93229 | 0.55262 | 0.3807 | 0.431456 | Not Significant | NS | #N/A |
| 449 | G1SJJ8 | phosphatidylinositol-3,4-bisphosphate 4-phosphat | INPP4A | RABBIT | 0.0338 | -0.40179 | -0.14277 | 0.52786 | 0.6408 | -0.49632 | -0.02213 | 0.16797 | 0.84579 | -0.69761 | -0.17204 | -0.51242 | -0.45691 | 0.6222 | 0.65668 | 0.45769 | 0.4985 | 8 | 2.93229 | 0.55262 | 0.28182 | 0.35445 | Not Significant | NS | #N/A |
| 1950 | B7NZL8 | #N/A | ZNF259 | RABBIT | #N/A | -0.05109 | #N/A | 0.06404 | 0.13905 | #N/A | #N/A | #N/A | #N/A | -0.12168 | -0.17235 | -1.5585 | -0.25894 | 0.25944 | 0.80614 | 0.30317 | 0.54212 | 2 | 2.93229 | 0.55262 | 0.00917 | 0.332255 | Not Significant | NS | #N/A |
| 1636 | G1TD82 | Leucine rich repeat containing 10 OS=Oryctolagus ( | #N/A | RABBIT | -1.81406 | #N/A | 2.15908 | #N/A | #N/A | 0 | #N/A | #N/A | #N/A | #N/A | -0.17251 | -0.05014 | -0.09056 | 0.96811 | 0.93227 | 0.55819 | 0.57497 | 1 | 2.93229 | 0.55262 | 7.89292 | 2.419293 | Not Significant | NS | #N/A |
| 2521 | G1U3A7 | #N/A | STRN4 | RABBIT | #N/A | #N/A | #N/A | 0.76197 | #N/A | 3.70805 | #N/A | -1.12237 | -0.8196 | #N/A | -0.17327 | -0.05546 | -0.08264 | 0.96081 | 0.93738 | 0.55716 | 0.57605 | 2 | 2.93229 | 0.55262 | 7.32071 | 3.297022 | Not Significant | NS | #N/A |
| 1567 | A5U216 | DNA-3-methyladenine glycosylase II OS=Mycobacte | #N/A | MT | -0.04314 | -0.24261 | #N/A | 0.15743 | 0.58323 | #N/A | -0.26254 | -0.00957 | 0.12152 | -0.08906 | -0.17364 | -0.89538 | -0.50989 | 0.40507 | 0.62249 | 0.37324 | 0.48491 | 6 | 2.93229 | 0.55262 | 0.07521 | 0.231935 | Not Significant | NS | #N/A |
| 2078 | G1SUF1 | #N/A | PBX2 | RABBIT | #N/A | 0.19782 | #N/A | #N/A | #N/A | #N/A | 0.46667 | #N/A | #N/A | -0.42158 | -0.17528 | -0.22785 | -0.19993 | 0.85738 | 0.85145 | 0.52914 | 0.55412 | 1 | 2.93229 | 0.55262 | 0.39449 | 0.512404 | Not Significant | NS | #N/A |
| 2139 | G1SML9 | #N/A | DNAJA1 | RABBIT | #N/A | #N/A | 0.11936 | 0.41891 | #N/A | -0.09083 | -0.79531 | 0.69097 | 0 | 0.66436 | -0.1753 | -0.37506 | -0.33087 | 0.723 | 0.74931 | 0.49079 | 0.52913 | 5 | 2.93229 | 0.55262 | 0.31208 | 0.401 | Not Significant | NS | #N/A |
| 2747 | G1SI81 | #N/A | HS3ST5 | RABBIT | #N/A | #N/A | #N/A | 0.05947 | #N/A | -0.1171 | #N/A | #N/A | #N/A | #N/A | -0.17657 | #N/A | -0.16795 | #N/A | 0.87753 | #N/A | 0.5612 | 0 | 2.93229 | 0.55262 | #N/A | 0.552617 | Not Significant | NS | #N/A |
| 1038 | G1TBQ6 | VAMP associated protein B and C OS=Oryctolagus ( | VAPB | RABBIT | -0.1931 | 0.19782 | #N/A | 0.75197 | 0.4032 | -0.24305 | -1.24082 | -0.78138 | 1.15986 | 1.66721 | -0.17761 | -0.26984 | -0.2886 | 0.79506 | 0.77882 | 0.51196 | 0.53632 | 7 | 2.93229 | 0.55262 | 0.96273 | 0.841654 | Not Significant | NS | #N/A |
| 2845 | G1SG48 | #N/A | WIPI2 | RABBIT | #N/A | #N/A | #N/A | #N/A | -0.01338 | #N/A | -2.17782 | 0.8599 | -0.51231 | 1.06107 | -0.17891 | -0.1069 | -0.13493 | 0.92162 | 0.89713 | 0.54531 | 0.56784 | 3 | 2.93229 | 0.55262 | 2.24092 | 1.406402 | Not Significant | NS | #N/A |
| 515 | A0A5F9CFG8 | protein-tyrosine-phosphatase OS=Oryctolagus cun | PTPRD | RABBIT | 0.71016 | 0.3221 | -0.04244 | 0.08507 | 0.4058 | -0.12428 | -0.25812 | -0.44567 | 1.74431 | -0.33594 | -0.18008 | -0.41799 | -0.40768 | 0.68695 | 0.69138 | 0.48039 | 0.51015 | 8 | 2.93229 | 0.55262 | 0.46402 | 0.48778 | Not Significant | NS | #N/A |
| 528 | G1TRH5 | Coronin OS=Oryctolagus cuniculus OX=9986 GN=( | CORO1C | RABBIT | -0.11605 | 3.25896 | -0.49042 | -0.3497 | -0.29128 | -0.02553 | 0.02553 | 0.7967 | 0.26399 | 0.04903 | -0.18036 | -0.24618 | -0.26825 | 0.81174 | 0.7935 | 0.5165 | 0.53888 | 8 | 2.93229 | 0.55262 | 1.34182 | 1.130137 | Not Significant | NS | #N/A |
| 2013 | A0A5F9DJ45 | #N/A | BCOR | RABBIT | #N/A | 1.30415 | -0.60043 | #N/A | 0.15237 | 0 | #N/A | -0.1707 | -0.15194 | 0.73671 | -0.18185 | -0.34421 | -0.33476 | 0.74469 | 0.74649 | 0.49742 | 0.52878 | 5 | 2.93229 | 0.55262 | 0.47847 | 0.50588 | Not Significant | NS | #N/A |
| 1366 | G1SM04 | Serine/threonine-protein kinase 3 OS=Oryctolagus ( | STK3 | RABBIT | 0.01166 | #N/A | -0.45791 | #N/A | #N/A | -1.60859 | 0.36723 | #N/A | 0.02439 | #N/A | -0.18253 | -0.22642 | -0.24473 | 0.83543 | 0.81491 | 0.5227 | 0.5433 | 3 | 2.93229 | 0.55262 | 0.77986 | 0.667537 | Not Significant | NS | #N/A |
| 1092 | G1T058 | Axonemal dynein light intermediate polypeptide 1 O | DNALI1 | RABBIT | 0.06511 | #N/A | #N/A | #N/A | #N/A | #N/A | #N/A | -0.11781 | #N/A | #N/A | -0.18292 | #N/A | -0.174 | #N/A | 0.87318 | #N/A | 0.56055 | 0 | 2.93229 | 0.55262 | #N/A | 0.552617 | Not Significant | NS | #N/A |
| 2700 | G1THJ8 | #N/A | #N/A | RABBIT | #N/A | #N/A | #N/A | 0.13346 | #N/A | 0.52921 | -0.08837 | -0.69263 | -0.0588 | 0.0588 | -0.18382 | -0.3845 | -0.28625 | 0.72017 | 0.78305 | 0.49014 | 0.53768 | 4 | 2.93229 | 0.55262 | 0.19046 | 0.343648 | Not Significant | NS | #N/A |
| 1031 | U3KM98 | Seizure related 6 homolog like OS=Oryctolagus cun | #N/A | RABBIT | -0.37563 | -0.18544 | 0.17065 | 1.09153 | 0.3137 | #N/A | 0.57391 | 0.35639 | -0.44473 | -0.41608 | -0.18559 | -0.50337 | -0.45116 | 0.63015 | 0.66156 | 0.46099 | 0.4996 | 7 | 2.93229 | 0.55262 | 0.30208 | 0.376046 | Not Significant | NS | #N/A |
| 1912 | G1SWM8 | #N/A | SCYL2 | RABBIT | #N/A | 0.16369 | 0.17065 | 0.58247 | 0.65376 | #N/A | -0.01073 | 0.7446 | -0.11579 | #N/A | -0.18661 | -0.67948 | -0.45705 | 0.52701 | 0.65989 | 0.42539 | 0.49949 | 5 | 2.93229 | 0.55262 | 0.12931 | 0.285789 | Not Significant | NS | #N/A |
| 1400 | A0A5F9C7X7 | Myotubularin-related protein 12 OS=Oryctolagus cu | MTMR12 | RABBIT | -1.04955 | -0.75911 | #N/A | 1.02423 | 2.35932 | -0.22925 | 0 | 1.26832 | 0.39095 | -0.39517 | -0.18675 | -0.23987 | -0.26394 | 0.81731 | 0.79722 | 0.51745 | 0.5392 | 7 | 2.93229 | 0.55262 | 1.34703 | 1.112495 | Not Significant | NS | #N/A |
| 2544 | G1TLC6 | #N/A | MARK2 | RABBIT | #N/A | #N/A | #N/A | 0.08641 | #N/A | #N/A | -0.46179 | -0.20842 | #N/A | 0.3672 | -0.18742 | -0.38208 | -0.25608 | 0.73918 | 0.80822 | 0.49534 | 0.54212 | 2 | 2.93229 | 0.55262 | 0.18046 | 0.40171 | Not Significant | NS | #N/A |
| 719 | G1SKT0 | Branched-chain-amino-acid aminotransferase OS= | BCAT1 | RABBIT | -0.21147 | 0.19782 | -0.04893 | 0.32584 | 0.53967 | 0.69464 | -0.50317 | #N/A | 0.12807 | -0.43181 | -0.18865 | -0.65507 | -0.51951 | 0.53335 | 0.61479 | 0.42779 | 0.48241 | 7 | 2.93229 | 0.55262 | 0.18431 | 0.293041 | Not Significant | NS | #N/A |
| 1050 | A0A5F9C1I5 | pyridoxal 5'-phosphate synthase OS=Oryctolagus c | PNPO | RABBIT | -0.23804 | 0.19782 | #N/A | #N/A | #N/A | #N/A | #N/A | -2.14289 | 0.88243 | 0.63392 | -0.18874 | -0.14951 | -0.18565 | 0.89064 | 0.8589 | 0.53768 | 0.55571 | 3 | 2.93229 | 0.55262 | 1.91222 | 1.240176 | Not Significant | NS | #N/A |
| 99 | G1U8F0 | AP-2 complex subunit alpha OS=Oryctolagus cunic | AP2A1 | RABBIT | 0.0046 | -0.54293 | 0.34057 | -0.05058 | 0.48099 | 0.1215 | -0.03183 | 0.05767 | -0.23679 | -0.6217 | -0.18876 | -0.84654 | -0.61026 | 0.42185 | 0.55416 | 0.3798 | 0.46296 | 8 | 2.93229 | 0.55262 | 0.1243 | 0.239184 | Not Significant | NS | #N/A |
| 139 | G1T9F3 | Karyopherin subunit beta 1 OS=Oryctolagus cunicu | KPNB1 | RABBIT | 0.01256 | 0.20653 | -0.32204 | -0.01317 | 0.15003 | -0.72834 | 0.17501 | -0.16606 | -0.00089 | -0.19037 | -0.18891 | -1.06605 | -0.65863 | 0.31751 | 0.52376 | 0.33233 | 0.45286 | 8 | 2.93229 | 0.55262 | 0.07851 | 0.205673 | Not Significant | NS | #N/A |
| 753 | G1U826 | V-type proton ATPase subunit G OS=Oryctolagus cu | #N/A | RABBIT | -0.03053 | -0.12413 | -0.118 | -0.54704 | 1.20339 | -1.4508 | 0.12235 | 0.62467 | 0.0895 | 0.0422 | -0.19115 | -0.417 | -0.41412 | 0.68764 | 0.68679 | 0.48039 | 0.50931 | 8 | 2.93229 | 0.55262 | 0.52532 | 0.532641 | Not Significant | NS | #N/A |
| 2185 | G1SM05 | #N/A | SAR1A | RABBIT | #N/A | #N/A | -0.43951 | 1.93781 | -1.03646 | 0.78947 | -0.76158 | -1.55807 | 0.73134 | 0.61016 | -0.19168 | -0.20879 | -0.23542 | 0.84152 | 0.81919 | 0.52455 | 0.54399 | 6 | 2.93229 | 0.55262 | 1.58038 | 1.242988 | Not Significant | NS | #N/A |
| 2470 | A0A5F9D8T2 | #N/A | #N/A | RABBIT | #N/A | #N/A | 0.18143 | #N/A | #N/A | #N/A | -0.01078 | #N/A | #N/A | #N/A | -0.19222 | #N/A | -0.18284 | #N/A | 0.86683 | #N/A | 0.55831 | 0 | 2.93229 | 0.55262 | #N/A | 0.552617 | Not Significant | NS | #N/A |
| 120 | G1TIZ1 | Ubiquitin carboxyl-terminal hydrolase OS=Oryctola | UCHL1 | RABBIT | -0.12822 | 0.38151 | -0.37479 | 0.25016 | 0.18275 | 0.04279 | -0.34162 | -0.45718 | 0.14176 | -0.04279 | -0.19369 | -1.08093 | -0.67318 | 0.31123 | 0.51481 | 0.32881 | 0.45011 | 8 | 2.93229 | 0.55262 | 0.08027 | 0.206965 | Not Significant | NS | #N/A |
| 1854 | G1T2N1 | #N/A | SPARC | RABBIT | #N/A | 0.16388 | #N/A | #N/A | #N/A | #N/A | #N/A | -0.03043 | #N/A | #N/A | -0.19431 | #N/A | -0.18483 | #N/A | 0.86541 | #N/A | 0.55792 | 0 | 2.93229 | 0.55262 | #N/A | 0.552617 | Not Significant | NS | #N/A |
| 395 | A0A5F9DPK6 | COP9 signalosome subunit 7B OS=Oryctolagus cur | COPS7B | RABBIT | 0.38199 | 0.17858 | -0.90045 | -0.05764 | 0.85211 | #N/A | -0.67714 | -0.04921 | 0.28411 | -0.00915 | -0.20376 | -0.54587 | -0.49187 | 0.60211 | 0.6335 | 0.45195 | 0.48899 | 7 | 2.93229 | 0.55262 | 0.30964 | 0.381375 | Not Significant | NS | #N/A |
| 2617 | A0A5F9DPW7 | #N/A | RFC2 | RABBIT | #N/A | #N/A | #N/A | 0.07346 | #N/A | -0.1311 | #N/A | #N/A | #N/A | #N/A | -0.20456 | #N/A | -0.19458 | #N/A | 0.85842 | #N/A | 0.55561 | 0 | 2.93229 | 0.55262 | #N/A | 0.552617 | Not Significant | NS | #N/A |
| 2641 | A0A5F9C168 | #N/A | NEXN | RABBIT | #N/A | #N/A | #N/A | 0.0743 | #N/A | #N/A | #N/A | #N/A | #N/A | -0.13194 | -0.20624 | #N/A | -0.19618 | #N/A | 0.85728 | #N/A | 0.55549 | 0 | 2.93229 | 0.55262 | #N/A | 0.552617 | Not Significant | NS | #N/A |
| 912 | A0A5F9C3U6 | Argininosuccinate lyase OS=Oryctolagus cuniculus | ASL | RABBIT | 0.00689 | 2.59476 | #N/A | -0.23521 | 0.59483 | #N/A | -0.35966 | -0.06437 | #N/A | 2.02614 | -0.20628 | -0.20921 | -0.24111 | 0.84254 | 0.81559 | 0.52455 | 0.5433 | 5 | 2.93229 | 0.55262 | 1.66657 | 1.254779 | Not Significant | NS | #N/A |
| 2799 | G1SW57 | #N/A | NT5E | RABBIT | #N/A | #N/A | #N/A | #N/A | -0.01338 | #N/A | #N/A | 0.06447 | -0.507 | #N/A | -0.20788 | -0.42003 | -0.25201 | 0.74684 | 0.81365 | 0.49801 | 0.54324 | 1 | 2.93229 | 0.55262 | 0.16329 | 0.45361 | Not Significant | NS | #N/A |
| 2768 | A0A5F9D9Z3 | #N/A | #N/A | RABBIT | #N/A | #N/A | #N/A | 0.04474 | #N/A | -0.2915 | #N/A | 0.05195 | -0.41392 | 0 | -0.2081 | -0.82626 | -0.3405 | 0.46924 | 0.74521 | 0.40077 | 0.52878 | 3 | 2.93229 | 0.55262 | 0.05075 | 0.298818 | Not Significant | NS | #N/A |
| 304 | G1SFI2 | Protein S100 OS=Oryctolagus cuniculus OX=9986 ( | S100B | RABBIT | 0.12078 | 1.84888 | -0.5884 | 0.13146 | -0.56843 | -1.73567 | -0.10912 | 0.29597 | -0.23361 | 1.68608 | -0.20812 | -0.29509 | -0.31989 | 0.77544 | 0.75508 | 0.50663 | 0.53124 | 8 | 2.93229 | 0.55262 | 1.2436 | 1.058266 | Not Significant | NS | #N/A |

| | | | | | | | | | | | | | | | | | | | | | | | | | | | | | | |
|---|---|---|---|---|---|---|---|---|---|---|---|---|---|---|---|---|---|---|---|---|---|---|---|---|---|---|---|---|---|---|
| 913 | A0A5F9DSC5 | Serum amyloid A like 1 OS=Oryctolagus cuniculus ( | SAAL1 | RABBIT | #N/A | 0.1127 | #N/A | 1.31549 | -0.81669 | #N/A | 0 | -0.3624 | 0.27934 | 0.06435 | -0.20851 | -0.38616 | -0.37883 | 0.71526 | 0.71475 | 0.48917 | 0.51773 | 5 | 2.93229 | 0.55262 | 0.4998 | 0.519322 | Not Significant | NS | | #N/A |
| 1896 | G1TMM3 | #N/A | #N/A | RABBIT | #N/A | -1.70814 | 3.11129 | -0.28063 | #N/A | 0.76821 | -0.21265 | 0.07497 | 0.32025 | -0.13934 | -0.21189 | -0.19797 | -0.22765 | 0.84961 | 0.82504 | 0.52706 | 0.54595 | 6 | 2.93229 | 0.55262 | 2.14798 | 1.624256 | Not Significant | NS | | #N/A |
| 2325 | Q7ARS9 | #N/A | #N/A | MT | #N/A | #N/A | -0.14452 | #N/A | 0.8092 | #N/A | -0.37236 | -0.52978 | 1.06818 | 0.31517 | -0.21203 | -0.34145 | -0.33618 | 0.74995 | 0.74668 | 0.49957 | 0.52878 | 4 | 2.93229 | 0.55262 | 0.51414 | 0.530416 | Not Significant | NS | | #N/A |
| 648 | A0A5F9D958 | Poly(rC) binding protein 2 OS=Oryctolagus cuniculu | #N/A | RABBIT | 0.11203 | #N/A | #N/A | #N/A | #N/A | #N/A | -0.10036 | #N/A | #N/A | #N/A | -0.21239 | #N/A | -0.20203 | #N/A | 0.8531 | #N/A | 0.55425 | 0 | 2.93229 | 0.55262 | #N/A | 0.552617 | Not Significant | NS | | #N/A |
| 2723 | G1T9F6 | #N/A | NDUFA6 | RABBIT | #N/A | #N/A | #N/A | -0.05764 | #N/A | #N/A | #N/A | 0.04136 | #N/A | -0.58154 | -0.21245 | -0.39383 | -0.25537 | 0.76115 | 0.81124 | 0.50173 | 0.54212 | 1 | 2.93229 | 0.55262 | 0.194 | 0.461418 | Not Significant | NS | | #N/A |
| 1668 | A0A5F9C772 | #N/A | CHL1 | RABBIT | #N/A | 0.67265 | -1.63211 | 0.46026 | -1.06498 | #N/A | 0.39789 | 0.19331 | -2.74981 | -0.25768 | -0.21303 | -0.2309 | -0.26173 | 0.82507 | 0.79947 | 0.51907 | 0.53965 | 6 | 2.93229 | 0.55262 | 1.70242 | 1.324964 | Not Significant | NS | | #N/A |
| 561 | G1SK22 | Ubiquitin-ribosomal protein eS31 fusion protein OS | RPS27A | RABBIT | -0.6935 | 1.26056 | 0.30222 | -0.76438 | 0.04089 | #N/A | 0.3137 | -0.62774 | -0.42259 | 0 | -0.21332 | -0.46466 | -0.45279 | 0.65628 | 0.66043 | 0.4707 | 0.49949 | 7 | 2.93229 | 0.55262 | 0.46834 | 0.493221 | Not Significant | NS | | #N/A |
| 106 | A0A5F9DMB6 | Nck-associated protein 1 OS=Oryctolagus cuniculu | NCKAP1 | RABBIT | -0.13616 | -0.9027 | 0.17065 | -0.05215 | 0.78659 | -1.31233 | -0.42916 | 0.50172 | 0.27507 | #N/A | -0.21442 | -0.45324 | -0.44598 | 0.66409 | 0.66517 | 0.47235 | 0.50064 | 7 | 2.93229 | 0.55262 | 0.49736 | 0.513672 | Not Significant | NS | | #N/A |
| 290 | G1T4G9 | Tropomodulin 2 OS=Oryctolagus cuniculus OX=998 | TMOD2 | RABBIT | 0.4229 | -1.458 | 0.29519 | -0.97622 | 0.25266 | -2.16028 | 0.46788 | -0.0971 | -0.78466 | 0.03273 | -0.21559 | -0.35931 | -0.37949 | 0.72866 | 0.71159 | 0.49212 | 0.51654 | 8 | 2.93229 | 0.55262 | 0.90005 | 0.806857 | Not Significant | NS | | #N/A |
| 657 | G1SRQ2 | Ubiquitin carboxyl-terminal hydrolase OS=Oryctola | UCHL3 | RABBIT | 0.10809 | 1.79573 | -1.64772 | 0.81287 | -0.10981 | -1.52288 | -0.1315 | -0.19138 | 0.63543 | 1.09075 | -0.21574 | -0.29913 | -0.32526 | 0.77247 | 0.75112 | 0.50582 | 0.52975 | 8 | 2.93229 | 0.55262 | 1.30048 | 1.099886 | Not Significant | NS | | #N/A |
| 2680 | P9WLC9 | #N/A | #N/A | MT | #N/A | #N/A | #N/A | 0.02343 | #N/A | -0.01037 | #N/A | #N/A | -0.58086 | 0.01037 | -0.21705 | -0.56025 | -0.3073 | 0.63169 | 0.77117 | 0.46125 | 0.53484 | 2 | 2.93229 | 0.55262 | 0.11257 | 0.374184 | Not Significant | NS | | #N/A |
| 2461 | G1STE8 | #N/A | MAP2K6 | RABBIT | #N/A | #N/A | 0.17065 | #N/A | #N/A | #N/A | #N/A | #N/A | 0.23968 | -0.33383 | -0.21772 | -0.43837 | -0.26386 | 0.73699 | 0.80513 | 0.49455 | 0.54212 | 1 | 2.93229 | 0.55262 | 0.16446 | 0.453905 | Not Significant | NS | | #N/A |
| 1832 | A0A5F9CNT7 | #N/A | SEC24B | RABBIT | #N/A | 0.08081 | #N/A | -0.04191 | -0.02912 | #N/A | #N/A | 0.61592 | 0.57901 | -1.83997 | -0.21828 | -0.26833 | -0.29768 | 0.80172 | 0.77467 | 0.51393 | 0.53578 | 4 | 2.93229 | 0.55262 | 0.99263 | 0.806507 | Not Significant | NS | | #N/A |
| 2542 | G1TYF2 | #N/A | GRK3 | RABBIT | #N/A | #N/A | #N/A | 0.08042 | #N/A | #N/A | #N/A | #N/A | #N/A | -0.13806 | -0.21847 | #N/A | -0.20781 | #N/A | 0.84897 | #N/A | 0.55382 | 0 | 2.93229 | 0.55262 | #N/A | 0.552617 | Not Significant | NS | | #N/A |
| 1712 | P06813 | #N/A | CAPNS1 C | RABBIT | #N/A | 1.43255 | #N/A | -1.2199 | #N/A | #N/A | 0.11642 | -0.18078 | -0.71619 | 0.32869 | -0.21929 | -0.24908 | -0.27793 | 0.81557 | 0.78917 | 0.51745 | 0.53858 | 4 | 2.93229 | 0.55262 | 1.03348 | 0.830078 | Not Significant | NS | | #N/A |
| 2834 | A0A5F9DNF4 | #N/A | #N/A | RABBIT | #N/A | #N/A | #N/A | #N/A | -0.01338 | 0.0515 | #N/A | #N/A | -0.52139 | #N/A | -0.22156 | -0.44658 | -0.26854 | 0.73262 | 0.80178 | 0.49265 | 0.5404 | 1 | 2.93229 | 0.55262 | 0.1641 | 0.453814 | Not Significant | NS | | #N/A |
| 1973 | A0A5F9CW79 | #N/A | KMO | RABBIT | #N/A | 0.35386 | -0.3537 | 0.47859 | -0.01338 | #N/A | -0.36324 | #N/A | 0.17761 | -0.13387 | -0.22284 | -0.86203 | -0.55487 | 0.42807 | 0.59428 | 0.38119 | 0.47435 | 5 | 2.93229 | 0.55262 | 0.11456 | 0.276492 | Not Significant | NS | | #N/A |
| 1561 | G1SYZ7 | DISC1 scaffold protein OS=Oryctolagus cuniculus ( | DISC1 | RABBIT | 0.08506 | -0.38234 | 0.29142 | -0.10929 | 0.56264 | -0.33521 | 0.25424 | -0.2842 | 0.05165 | -0.35759 | -0.22372 | -1.1015 | -0.74791 | 0.30271 | 0.4703 | 0.32474 | 0.42992 | 8 | 2.93229 | 0.55262 | 0.10313 | 0.223692 | Not Significant | NS | | #N/A |
| 1476 | A0A5F9CM99 | Dystrobrevin OS=Oryctolagus cuniculus OX=9986 ( | DTNA | RABBIT | #N/A | #N/A | #N/A | -0.05764 | #N/A | -0.63915 | #N/A | #N/A | 0.07435 | #N/A | -0.22476 | -0.36374 | -0.26576 | 0.77791 | 0.80377 | 0.50703 | 0.54149 | 1 | 2.93229 | 0.55262 | 0.25454 | 0.476815 | Not Significant | NS | | #N/A |
| 2662 | B7NZH6 | #N/A | OBSL1 | RABBIT | #N/A | #N/A | #N/A | -0.58902 | 0.97553 | #N/A | #N/A | -0.33122 | #N/A | 0.26686 | -0.22544 | -0.26918 | -0.28795 | 0.81302 | 0.78509 | 0.51664 | 0.53833 | 2 | 2.93229 | 0.55262 | 0.70138 | 0.612938 | Not Significant | NS | | #N/A |
| 1072 | A0A5F9CRW2 | High mobility group nucleosome binding domain 5 ( | HMGN5 | RABBIT | -2.79975 | 1.13108 | -0.28038 | -0.74053 | 1.33472 | #N/A | 0 | -0.05133 | -2.00272 | 0.06587 | -0.22607 | -0.23671 | -0.26712 | 0.81966 | 0.79484 | 0.51749 | 0.53895 | 7 | 2.93229 | 0.55262 | 2.027 | 1.591719 | Not Significant | NS | | #N/A |
| 1757 | G1SZ91 | #N/A | FABP5 | RABBIT | #N/A | 0.212 | #N/A | #N/A | #N/A | #N/A | -0.01418 | #N/A | #N/A | #N/A | -0.22617 | #N/A | -0.21514 | #N/A | 0.84375 | #N/A | 0.55173 | 0 | 2.93229 | 0.55262 | #N/A | 0.552617 | Not Significant | NS | | #N/A |
| 87 | A0A5F9D8G4 | Cytoplasmic FMR1-interacting protein OS=Oryctola | CYFIP2 | RABBIT | 0.17834 | -0.30723 | #N/A | 0.30928 | #N/A | #N/A | #N/A | #N/A | #N/A | -0.16668 | -0.22681 | -0.60472 | -0.32234 | 0.60683 | 0.76041 | 0.45363 | 0.53222 | 2 | 2.93229 | 0.55262 | 0.1055 | 0.371316 | Not Significant | NS | | #N/A |
| 651 | A0A5F9C4E2 | Aldo-keto reductase family 1 member B1 OS=Oryct | AKR1B1 | RABBIT | 0.01166 | #N/A | #N/A | #N/A | #N/A | #N/A | #N/A | -0.62647 | #N/A | 0.19577 | -0.22701 | -0.31881 | -0.26265 | 0.80353 | 0.80601 | 0.51441 | 0.54212 | 1 | 2.93229 | 0.55262 | 0.33804 | 0.498048 | Not Significant | NS | | #N/A |
| 1483 | A0A5F9CB41 | Maturin OS=Oryctolagus cuniculus OX=9986 GN=P | PLEKHA8 | RABBIT | 0.35603 | -0.27102 | 0.17065 | 0.02208 | 0.39576 | -0.06906 | #N/A | -0.724 | 0.62338 | -0.20001 | -0.22712 | -0.8113 | -0.63322 | 0.4439 | 0.54089 | 0.38936 | 0.4607 | 7 | 2.93229 | 0.55262 | 0.17416 | 0.285891 | Not Significant | NS | | #N/A |
| 1036 | G1SSN9 | RAN binding protein 9 OS=Oryctolagus cuniculus O | RANBP9 | RABBIT | 1.54759 | #N/A | 0.20067 | -0.08766 | -0.12195 | #N/A | -0.44029 | 0.75429 | #N/A | #N/A | -0.22766 | -0.32735 | -0.33773 | 0.75982 | 0.74556 | 0.50158 | 0.52878 | 4 | 2.93229 | 0.55262 | 0.64488 | 0.605852 | Not Significant | NS | | #N/A |
| 2513 | G1U0H7 | #N/A | SH3GL3 | RABBIT | #N/A | #N/A | #N/A | 0.19967 | -0.07628 | 0.18003 | 0.06289 | -0.79561 | -0.11425 | #N/A | -0.22843 | -0.67584 | -0.46509 | 0.5362 | 0.65612 | 0.42838 | 0.4985 | 4 | 2.93229 | 0.55262 | 0.15232 | 0.321642 | Not Significant | NS | | #N/A |
| 2187 | G1TTQ5 | #N/A | RPL36 | RABBIT | #N/A | #N/A | 0.17065 | -0.54525 | 0.50394 | #N/A | -0.39113 | #N/A | #N/A | 0.01702 | -0.23017 | -0.53834 | -0.40684 | 0.62772 | 0.69839 | 0.4603 | 0.5134 | 3 | 2.93229 | 0.55262 | 0.21936 | 0.384089 | Not Significant | NS | | #N/A |
| 917 | G1SUK4 | Mannose-6-phosphate isomerase OS=Oryctolagus | MPI | RABBIT | 0.01166 | #N/A | #N/A | #N/A | 0.44913 | -0.00089 | #N/A | #N/A | #N/A | #N/A | -0.23129 | -0.61049 | -0.28587 | 0.65107 | 0.78941 | 0.46919 | 0.53858 | 1 | 2.93229 | 0.55262 | 0.09569 | 0.436418 | Not Significant | NS | | #N/A |
| 2538 | G1TBW7 | #N/A | GNAQ | RABBIT | #N/A | #N/A | #N/A | -0.05764 | #N/A | -2.30557 | -0.14289 | #N/A | 0.2932 | 0.99879 | -0.23148 | -0.14537 | -0.18166 | 0.89364 | 0.8619 | 0.53883 | 0.55718 | 3 | 2.93229 | 0.55262 | 2.02842 | 1.298943 | Not Significant | NS | | #N/A |
| 1703 | G1TKV4 | #N/A | #N/A | RABBIT | #N/A | 0.2655 | #N/A | -0.12532 | 0.37629 | -0.87015 | 1.8983 | 0.19217 | -0.86252 | -0.65589 | -0.23177 | -0.32582 | -0.35074 | 0.75563 | 0.73391 | 0.50097 | 0.52403 | 6 | 2.93229 | 0.55262 | 0.94881 | 0.818746 | Not Significant | NS | | #N/A |
| 205 | G1U6N8 | Nuclear mitotic apparatus protein 1 OS=Oryctolagu | NUMA1 | RABBIT | -1.00902 | -1.27873 | 0.78575 | -0.98873 | 0.87964 | -4.19585 | 0.97936 | 0.31909 | -0.38345 | 0.50797 | -0.23236 | -0.22136 | -0.24975 | 0.83036 | 0.80741 | 0.52093 | 0.54212 | 8 | 2.93229 | 0.55262 | 2.75454 | 2.163932 | Not Significant | NS | | #N/A |
| 595 | G1T874 | Zinc finger ZZ-type and EF-hand domain containing | ZZEF1 | RABBIT | -0.34898 | #N/A | -0.75905 | 0.303 | 2.12508 | #N/A | 1.72082 | -1.59907 | 1.68398 | -1.42305 | -0.23435 | -0.20813 | -0.24142 | 0.84201 | 0.81468 | 0.52455 | 0.5433 | 6 | 2.93229 | 0.55262 | 2.53544 | 1.884519 | Not Significant | NS | | #N/A |
| 543 | G1SF95 | Heterogeneous nuclear ribonucleoprotein D OS=Or | HNRNPD | RABBIT | -0.22881 | 0.12212 | 0.06599 | 0.7774 | -0.00883 | 0 | 0.32645 | 0.70444 | -1.3876 | #N/A | -0.23475 | -0.52872 | -0.50942 | 0.61334 | 0.62158 | 0.45455 | 0.48486 | 7 | 2.93229 | 0.55262 | 0.43808 | 0.471891 | Not Significant | NS | | #N/A |
| 840 | G1TI40 | Small nuclear ribonucleoprotein Sm D2 OS=Oryctol | SNRPD2 | RABBIT | -1.42827 | -2.46551 | 1.35188 | 0.50971 | 1.81331 | -1.85146 | -0.46758 | 1.25145 | -0.79334 | 0.46758 | -0.23489 | -0.24001 | -0.2694 | 0.81636 | 0.79264 | 0.51745 | 0.53886 | 8 | 2.93229 | 0.55262 | 2.39462 | 1.900555 | Not Significant | NS | | #N/A |
| 492 | G1SEM0 | Fermitin family member 2 OS=Oryctolagus cuniculu | FERMT2 | RABBIT | -0.3673 | 2.4738 | 0.26001 | -0.147 | -0.25093 | -0.42956 | -0.11712 | 0.65572 | 0.23906 | 0.44115 | -0.23587 | -0.41738 | -0.43575 | 0.68738 | 0.6715 | 0.48039 | 0.50325 | 8 | 2.93229 | 0.55262 | 0.79839 | 0.732466 | Not Significant | NS | | #N/A |
| 2256 | G1U426 | #N/A | UNC80 | RABBIT | #N/A | #N/A | 0.20401 | #N/A | #N/A | -0.03336 | #N/A | #N/A | #N/A | #N/A | -0.23737 | #N/A | -0.22579 | #N/A | 0.83618 | #N/A | 0.54964 | 0 | 2.93229 | 0.55262 | #N/A | 0.552617 | Not Significant | NS | | #N/A |
| 1123 | G1TAL4 | TGF-beta receptor type-2 OS=Oryctolagus cuniculu | TGFBR2 | RABBIT | -0.23682 | -0.36593 | 0.37344 | 0.07456 | 0.80767 | #N/A | 0.54779 | -0.19656 | #N/A | -0.67289 | -0.2378 | -0.61924 | -0.5374 | 0.55852 | 0.60412 | 0.43625 | 0.47865 | 6 | 2.93229 | 0.55262 | 0.27651 | 0.367151 | Not Significant | NS | | #N/A |
| 2742 | G1STE1 | #N/A | NSUN4 | RABBIT | #N/A | #N/A | #N/A | -0.13782 | 0.61621 | #N/A | #N/A | #N/A | #N/A | 0 | -0.2392 | -0.3663 | -0.28062 | 0.77647 | 0.79314 | 0.50687 | 0.53888 | 1 | 2.93229 | 0.55262 | 0.28428 | 0.484377 | Not Significant | NS | | #N/A |
| 234 | G1T9S4 | Acetyltransferase component of pyruvate dehydrog | DLAT | RABBIT | 0.05686 | -0.91254 | 1.10632 | -0.53518 | 1.35341 | -1.447 | -0.64556 | 1.5476 | -0.0452 | 0.45996 | -0.23982 | -0.3562 | -0.38353 | 0.7309 | 0.70868 | 0.49252 | 0.51642 | 8 | 2.93229 | 0.55262 | 1.13317 | 0.977455 | Not Significant | NS | | #N/A |
| 1408 | A0A5F9DTM1 | Vitellogenin domain-containing protein OS=Oryctol | #N/A | RABBIT | 0.1261 | #N/A | #N/A | #N/A | #N/A | #N/A | #N/A | #N/A | #N/A | -0.11444 | -0.24053 | #N/A | -0.2288 | #N/A | 0.83405 | #N/A | 0.54958 | 0 | 2.93229 | 0.55262 | #N/A | 0.552617 | Not Significant | NS | | #N/A |
| 1222 | G1SHG0 | Large ribosomal subunit protein eL31 OS=Oryctolag | RPL31 | RABBIT | -2.96781 | -1.76341 | 1.28215 | 1.05424 | 1.2615 | -0.05672 | -2.47016 | 0.25068 | -0.1187 | 0.05672 | -0.24097 | -0.23461 | -0.2643 | 0.8204 | 0.79647 | 0.51751 | 0.5392 | 8 | 2.93229 | 0.55262 | 2.63737 | 2.078193 | Not Significant | NS | | #N/A |
| 2848 | A0A5F9CQN7 | #N/A | TRIM24 | RABBIT | #N/A | #N/A | #N/A | #N/A | -0.01338 | #N/A | 0.25147 | #N/A | -0.76276 | #N/A | -0.24226 | -0.27581 | -0.26846 | 0.82867 | 0.80184 | 0.52053 | 0.5404 | 1 | 2.93229 | 0.55262 | 0.51434 | 0.542882 | Not Significant | NS | | #N/A |
| 1521 | G1SZA0 | Kelch like family member 5 OS=Oryctolagus cunicu | KLHL5 | RABBIT | 0.46408 | #N/A | #N/A | 0.26764 | -0.35847 | -0.17173 | #N/A | -0.06437 | #N/A | #N/A | -0.24247 | -0.75142 | -0.45799 | 0.50698 | 0.66326 | 0.41787 | 0.50024 | 3 | 2.93229 | 0.55262 | 0.12495 | 0.33634 | Not Significant | NS | | #N/A |
| 2322 | A0A5F9D8Z2 | #N/A | ANLN | RABBIT | #N/A | #N/A | 0.29893 | #N/A | -0.68818 | 0.94917 | -0.12828 | 0.62498 | -3.19461 | #N/A | -0.24256 | -0.16711 | -0.20566 | 0.87539 | 0.84296 | 0.53468 | 0.55164 | 4 | 2.93229 | 0.55262 | 2.80904 | 1.854595 | Not Significant | NS | | #N/A |
| 2474 | G1T7W7 | #N/A | STT3A | RABBIT | #N/A | #N/A | 0.20696 | #N/A | #N/A | -0.03631 | #N/A | #N/A | #N/A | #N/A | -0.24327 | #N/A | -0.2314 | #N/A | 0.83221 | #N/A | 0.54924 | 0 | 2.93229 | 0.55262 | #N/A | 0.552617 | Not Significant | NS | | #N/A |
| 192 | G1T8R1 | Platelet-activating factor acetylhydrolase IB subuni | PAFAH1B1 | RABBIT | 0.38627 | 0.30242 | -0.37981 | 0.13054 | -0.10822 | -0.62302 | -0.27686 | 0.01304 | -0.04423 | 0.04423 | -0.24361 | -1.29907 | -0.83546 | 0.23011 | 0.42134 | 0.2895 | 0.4086 | 8 | 2.93229 | 0.55262 | 0.08792 | 0.212559 | Not Significant | NS | | #N/A |
| 337 | A0A5F9DL56 | Dihydrolipoyl dehydrogenase OS=Oryctolagus cuni | #N/A | RABBIT | 0.60172 | 0.00168 | 0.3668 | -0.97527 | 0.29711 | -1.52064 | -0.77395 | 1.06867 | -0.33091 | 0.62988 | -0.24379 | -0.4486 | -0.46455 | 0.66562 | 0.65137 | 0.47235 | 0.49768 | 8 | 2.93229 | 0.55262 | 0.73835 | 0.68853 | Not Significant | NS | | #N/A |
| 2745 | G1SWL5 | #N/A | PWWP3B | RABBIT | #N/A | #N/A | #N/A | -1.12506 | 1.61356 | #N/A | 0 | #N/A | #N/A | #N/A | -0.24425 | -0.10299 | -0.17065 | 0.93467 | 0.87292 | 0.54842 | 0.56055 | 1 | 2.93229 | 0.55262 | 3.75002 | 1.365732 | Not Significant | NS | | #N/A |
| 1697 | G1TXT1 | #N/A | H3C7 | RABBIT | #N/A | 0.19782 | -1.39805 | 1.73827 | -1.18251 | 0.14311 | 0.11797 | -1.47984 | #N/A | #N/A | -0.24514 | -0.25312 | -0.29085 | 0.81025 | 0.77862 | 0.51584 | 0.53632 | 5 | 2.93229 | 0.55262 | 1.60779 | 1.217733 | Not Significant | NS | | #N/A |
| 2128 | G1SHI9 | #N/A | OGDH | RABBIT | #N/A | #N/A | 0.28082 | #N/A | #N/A | #N/A | -0.62679 | 1.14319 | -0.37875 | 0 | -0.24641 | -0.28153 | -0.28863 | 0.7966 | 0.78268 | 0.51222 | 0.53764 | 3 | 2.93229 | 0.55262 | 0.61282 | 0.583063 | Not Significant | NS | | #N/A |
| 2609 | G1THY5 | #N/A | PPIL1 | RABBIT | #N/A | #N/A | #N/A | 0.74699 | #N/A | 2.42194 | -0.40256 | #N/A | -0.01785 | 0 | -0.24661 | -0.1704 | -0.20838 | 0.87554 | 0.8419 | 0.53468 | 0.55137 | 3 | 2.93229 | 0.55262 | 1.67554 | 1.120489 | Not Significant | NS | | #N/A |
| 1783 | G1SL25 | #N/A | GDAP1L1 | RABBIT | #N/A | 0.29806 | #N/A | #N/A | #N/A | 1.19078 | -0.93781 | #N/A | #N/A | -0.10024 | -0.24715 | -0.1996 | -0.24008 | 0.86025 | 0.81992 | 0.52967 | 0.54404 | 2 | 2.93229 | 0.55262 | 1.14986 | 0.794795 | Not Significant | NS | | #N/A |
| 2862 | G1TBK0 | #N/A | ANKMY2 | RABBIT | #N/A | #N/A | #N/A | #N/A | -0.01338 | -0.83394 | #N/A | #N/A | 0.30079 | #N/A | -0.25319 | -0.25765 | -0.27244 | 0.83947 | 0.79899 | 0.52431 | 0.53955 | 1 | 2.93229 | 0.55262 | 0.6438 | 0.575805 | Not Significant | NS | | #N/A |
| 889 | A0A5F9CSG4 | Histone-binding protein RBBP7 OS=Oryctolagus cu | #N/A | RABBIT | 1.55529 | -0.04352 | 0.853 | -0.04932 | -0.48303 | -0.00832 | 0.99907 | -0.27827 | -0.22491 | 0.06761 | -0.25545 | -0.58745 | -0.57457 | 0.5731 | 0.57721 | 0.44118 | 0.47042 | 8 | 2.93229 | 0.55262 | 0.47272 | 0.494153 | Not Significant | NS | | #N/A |
| 2084 | G1TA83 | #N/A | ANXA4 | RABBIT | #N/A | -0.54624 | 0.22879 | #N/A | #N/A | 0.07381 | #N/A | -1.32107 | 0 | #N/A | -0.25703 | -0.39395 | -0.38621 | 0.71995 | 0.71281 | 0.49014 | 0.5172 | 3 | 2.93229 | 0.55262 | 0.51082 | 0.531479 | Not Significant | NS | | #N/A |
| 1508 | A0A5F9D8W6 | Uncharacterized protein OS=Oryctolagus cuniculus | #N/A | RABBIT | 0.20495 | #N/A | #N/A | #N/A | #N/A | #N/A | -0.03985 | -0.06437 | #N/A | #N/A | -0.25706 | -12.1084 | -0.32693 | 0.05246 | 0.76038 | 0.15219 | 0.53222 | 1 | 2.93229 | 0.55262 | 0.0003 | 0.41216 | Not Significant | NS | | #N/A |
| 1741 | P13280 | #N/A | GYG1 GYG | RABBIT | #N/A | 0.24765 | #N/A | #N/A | #N/A | #N/A | 0 | #N/A | #N/A | -0.0204 | -0.25785 | -14.5942 | -0.32795 | 0.04355 | 0.75967 | 0.14581 | 0.53222 | 1 | 2.93229 | 0.55262 | 0.00021 | 0.412137 | Not Significant | NS | | #N/A |
| 2272 | G1TP04 | #N/A | MAL2 | RABBIT | #N/A | #N/A | 0.54849 | -0.57607 | 0.89983 | -0.00323 | 0.00323 | 0.64411 | -0.46444 | -0.02082 | -0.25898 | -0.64481 | -0.57183 | 0.5429 | 0.58154 | 0.43035 | 0.47236 | 6 | 2.93229 | 0.55262 | 0.30246 | 0.384582 | Not Significant | NS | | #N/A |
| 334 | G1SUC6 | ELKS/RAB6-interacting/CAST family member 2 OS=( | ERC2 | RABBIT | #N/A | -1.24564 | 2.22899 | -2.61101 | 2.66365 | #N/A | -0.93086 | #N/A | 0.93086 | #N/A | -0.259 | -0.12782 | -0.16237 | 0.90446 | 0.87565 | 0.54245 | 0.56083 | 4 | 2.93229 | 0.55262 | 5.47427 | 3.392463 | Not Significant | NS | | #N/A |
| 2697 | A0A5F9DV01 | #N/A | JAKMIP3 | RABBIT | #N/A | #N/A | #N/A | 0.06852 | #N/A | #N/A | #N/A | -0.19052 | #N/A | #N/A | -0.25904 | #N/A | -0.2464 | #N/A | 0.82161 | #N/A | 0.54495 | 0 | 2.93229 | 0.55262 | #N/A | 0.552617 | Not Significant | NS | | #N/A |
| 361 | G1SZA3 | RAB3 GTPase activating non-catalytic protein subur | RAB3GAP2 | RABBIT | 0.22399 | -0.30713 | 0.36488 | -0.2572 | 0.36622 | -0.68364 | -0.07674 | 0.01238 | 0.95213 | -1.11115 | -0.25956 | -0.68193 | -0.63839 | 0.51454 | 0.53636 | 0.42125 | 0.45752 | 8 | 2.93229 | 0.55262 | 0.36218 | 0.413258 | Not Significant | NS | | #N/A |
| 2645 | G1TZ95 | #N/A | #N/A | RABBIT | #N/A | #N/A | #N/A | -0.23936 | 0.63463 | #N/A | #N/A | -0.06437 | #N/A | #N/A | -0.262 | -0.34615 | -0.29978 | 0.78785 | 0.77952 | 0.50995 | 0.53632 | 1 | 2.93229 | 0.55262 | 0.38192 | 0.509208 | Not Significant | NS | | #N/A |
| 131 | G1SM15 | AP-2 complex subunit mu OS=Oryctolagus cuniculu | AP2M1 | RABBIT | 0.22092 | -0.25497 | 0.39132 | -0.10974 | 0.62902 | -0.21547 | -0.31009 | 0.1916 | 0.0521 | -0.15503 | -0.26269 | -1.41543 | -0.9037 | 0.19467 | 0.38563 | 0.2651 | 0.39334 | 8 | 2.93229 | 0.55262 | 0.08611 | 0.211236 | Not Significant | NS | | #N/A |
| 1108 | A0A5F9C2W8 | phosphatidylinositol-3-phosphatase OS=Oryctolag | MTMR6 | RABBIT | -0.11463 | 0.85094 | -1.2954 | 0.56569 | -0.59126 | -1.69566 | -0.85765 | 0.17526 | 0.35364 | 0.12629 | -0.26269 | -0.4765 | -0.49494 | 0.64646 | 0.63044 | 0.46795 | 0.4889 | 8 | 2.93229 | 0.55262 | 0.75981 | 0.70424 | Not Significant | NS | | #N/A |
| 1622 | G1T9J3 | NBAS subunit of NRZ tethering complex OS=Oryctol | NBAS | RABBIT | 0.03326 | 0.84785 | #N/A | -0.15543 | #N/A | #N/A | #N/A | #N/A | -0.02159 | #N/A | -0.26349 | -0.42796 | -0.34252 | 0.71036 | 0.74607 | 0.4881 | 0.52878 | 2 | 2.93229 | 0.55262 | 0.28429 | 0.443812 | Not Significant | NS | | #N/A |
| 1750 | A0A5F9C1Z9 | #N/A | PLCG1 | RABBIT | #N/A | 0.6584 | #N/A | #N/A | #N/A | #N/A | #N/A | 2.6762 | -1.03182 | -0.46058 | -0.2638 | -0.11443 | -0.16382 | 0.91935 | 0.87637 | 0.54531 | 0.56087 | 2 | 2.93229 | 0.55262 | 3.98586 | 1.944766 | Not Significant | NS | | #N/A |
| 649 | G1SLM2 | Target of myb1 like 2 membrane trafficking protein ( | TOM1L2 | RABBIT | -0.2057 | 0.32853 | 0.08803 | 0.02498 | 0.29858 | -0.27466 | -0.13827 | 0.12076 | 0.33511 | -0.82837 | -0.26397 | -1.19562 | -0.85662 | 0.26608 | 0.41004 | 0.30603 | 0.40614 | 8 | 2.93229 | 0.55262 | 0.12186 | 0.237401 | Not Significant | NS | | #N/A |
| 1978 | G1TE28 | #N/A | PEX2 | RABBIT | #N/A | 0.14015 | 0.25135 | #N/A | 0.04428 | #N/A | -0.12143 | #N/A | #N/A | #N/A | -0.26669 | -2.22875 | -0.4003 | 0.15564 | 0.70569 | 0.23612 | 0.51574 | 2 | 2.93229 | 0.55262 | 0.01074 | 0.33289 | Not Significant | NS | | #N/A |
| 2036 | G1TYD8 | #N/A | #N/A | RABBIT | #N/A | 0.23227 | #N/A | #N/A | #N/A | #N/A | #N/A | #N/A | #N/A | -0.03445 | -0.26672 | #N/A | -0.2537 | #N/A | 0.81647 | #N/A | 0.54344 | 0 | 2.93229 | 0.55262 | #N/A | 0.552617 | Not Significant | NS | | #N/A |
| 371 | G1SCY4 | F-actin-capping protein subunit alpha OS=Oryctola | #N/A | RABBIT | 0.35523 | -0.11024 | -0.50206 | 0.14 | 0.10435 | -1.67105 | 0.47647 | -0.08082 | -0.09173 | 0.01645 | -0.26759 | -0.6807 | -0.64452 | 0.51527 | 0.53253 | 0.42132 | 0.4563 | 8 | 2.93229 | 0.55262 | 0.38633 | 0.430932 | Not Significant | NS | | #N/A |
| 1779 | G1T0D8 | #N/A | DTD1 | RABBIT | #N/A | 0.20074 | #N/A | #N/A | #N/A | #N/A | #N/A | -0.06728 | #N/A | #N/A | -0.26802 | #N/A | -0.25494 | #N/A | 0.8156 | #N/A | 0.5433 | 0 | 2.93229 | 0.55262 | #N/A | 0.552617 | Not Significant | NS | | #N/A |
| 162 | G1TS42 | Glycogen debranching enzyme OS=Oryctolagus cu | AGL | RABBIT | 0.01166 | -0.31941 | -0.35596 | 0.36186 | 0.1116 | #N/A | 0.02319 | -0.79015 | 0.09201 | -0.55337 | -0.26903 | -1.10184 | -0.79184 | 0.30697 | 0.44695 | 0.32551 | 0.4199 | 7 | 2.93229 | 0.55262 | 0.13248 | 0.256518 | Not Significant | NS | | #N/A |
| 405 | G1TYZ6 | A-kinase anchoring protein 5 OS=Oryctolagus cunic | AKAP5 | RABBIT | 0.97087 | -2.27289 | 0.08479 | 1.04679 | 0.09433 | -1.32971 | -0.70964 | 0.0215 | 0.86717 | -0.275 | -0.26992 | -0.3834 | -0.41551 | 0.71141 | 0.68581 | 0.48851 | 0.50924 | 8 | 2.93229 | 0.55262 | 1.23908 | 1.054952 | Not Significant | NS | | #N/A |
| 358 | G1TGK3 | Phosphoribosyl pyrophosphate synthetase associa | PRPSAP1 | RABBIT | 0.08511 | -1.75781 | 0.30141 | 0.0306 | 0.8786 | #N/A | -0.04326 | -0.06437 | -1.29555 | -0.04961 | -0.27078 | -0.47395 | -0.49155 | 0.64996 | 0.63372 | 0.46888 | 0.48899 | 7 | 2.93229 | 0.55262 | 0.72535 | 0.674352 | Not Significant | NS | | #N/A |
| 1794 | A0A5F9CZG9 | #N/A | #N/A | RABBIT | #N/A | 1.39102 | -0.03095 | 0.04205 | #N/A | #N/A | #N/A | -0.16405 | 0.95042 | -0.20594 | -0.2739 | -0.45834 | -0.45531 | 0.6705 | 0.66281 | 0.47323 | 0.50018 | 4 | 2.93229 | 0.55262 | 0.53567 | 0.542838 | Not Significant | NS | | #N/A |
| 1572 | G1T8U2 | GDP-mannose 4,6-dehydratase OS=Oryctolagus cu | GMDS | RABBIT | -0.31774 | -0.57192 | 0.96114 | -0.2427 | 0.92645 | 0.18507 | 0.53909 | 0.45613 | -0.28819 | -1.5131 | -0.27525 | -0.55141 | -0.55995 | 0.59641 | 0.5868 | 0.44987 | 0.47237 | 8 | 2.93229 | 0.55262 | 0.62292 | 0.60406 | Not Significant | NS | | #N/A |
| 784 | G1TM49 | Cell division cycle and apoptosis regulator 1 OS=Or | CCAR1 | RABBIT | -0.2835 | #N/A | 1.22367 | -1.25093 | 0.62672 | #N/A | -1.4106 | 0.80607 | -0.47596 | 0.29517 | -0.27532 | -0.37969 | -0.41324 | 0.71725 | 0.68918 | 0.48961 | 0.50986 | 6 | 2.93229 | 0.55262 | 1.05157 | 0.887773 | Not Significant | NS | | #N/A |
| 2810 | G1SR64 | #N/A | FER1L5 | RABBIT | #N/A | #N/A | #N/A | #N/A | 0.09959 | #N/A | #N/A | -0.17734 | #N/A | #N/A | -0.27693 | #N/A | -0.26342 | #N/A | 0.80965 | #N/A | 0.54212 | 0 | 2.93229 | 0.55262 | #N/A | 0.552617 | Not Significant | NS | | #N/A |
| 428 | A0A5F9CZC1 | Abl interactor 1 OS=Oryctolagus cuniculus OX=998 | ABI1 | RABBIT | -0.8311 | -1.39249 | 0.59165 | 0.15324 | 0.70816 | -1.01843 | -0.08742 | 0.65282 | -1.80294 | 0.08742 | -0.2796 | -0.46682 | -0.49288 | 0.65308 | 0.63185 | 0.46994 | 0.4889 | 8 | 2.93229 | 0.55262 | 0.89684 | 0.804515 | Not Significant | NS | | #N/A |
| 2633 | A0A5F9D8F8 | #N/A | ARPP21 | RABBIT | #N/A | #N/A | #N/A | -0.05764 | 0.04059 | #N/A | -0.29044 | #N/A | #N/A | #N/A | -0.28192 | -3.31415 | -0.35804 | 0.18656 | 0.7387 | 0.26042 | 0.52575 | 1 | 2.93229 | 0.55262 | 0.00482 | 0.41331 | Not Significant | NS | | #N/A |
| 316 | G1SR75 | Serine/threonine-protein phosphatase OS=Oryctola | PPP3CC | RABBIT | 1.0521 | -0.29085 | #N/A | 0.19256 | -0.24053 | -0.51383 | #N/A | -0.06437 | 0.26424 | #N/A | -0.28297 | -0.68461 | -0.59411 | 0.52403 | 0.56899 | 0.4245 | 0.46793 | 5 | 2.93229 | 0.55262 | 0.29288 | 0.388894 | Not Significant | NS | | #N/A |
| 1152 | A0A5F9CKX8 | NAC-A/B domain-containing protein OS=Oryctolagu | #N/A | RABBIT | -0.10504 | #N/A | 0.5734 | 0.68264 | -0.01338 | #N/A | -0.40693 | 0.78991 | #N/A | -0.38208 | -0.2841 | -0.6985 | -0.60103 | 0.51601 | 0.5646 | 0.42132 | 0.46697 | 5 | 2.93229 | 0.55262 | 0.2836 | 0.383044 | Not Significant | NS | | #N/A |
| 459 | G1SDF2 | Protein-L-isoaspartate O-methyltransferase OS=Or | PCMT1 | RABBIT | 0.45686 | -0.10212 | -0.10249 | -0.05764 | 0.06218 | -0.59688 | -0.89924 | 0.05605 | 0.50785 | #N/A | -0.28442 | -0.93728 | -0.76476 | 0.37981 | 0.4622 | 0.36082 | 0.42567 | 7 | 2.93229 | 0.55262 | 0.20462 | 0.307359 | Not Significant | NS | | #N/A |
| 2460 | A0A5F9C0Q2 | #N/A | #N/A | RABBIT | #N/A | #N/A | 0.09246 | 1.04156 | #N/A | #N/A | #N/A | 1.02366 | 0 | -0.17858 | -0.28531 | -0.47624 | -0.44605 | 0.66644 | 0.67138 | 0.47235 | 0.50325 | 3 | 2.93229 | 0.55262 | 0.43071 | 0.490965 | Not Significant | NS | | #N/A |
| 2498 | Q95223 | #N/A | ARRB1 | RABBIT | #N/A | #N/A | #N/A | -0.11689 | #N/A | 0.05925 | #N/A | #N/A | 0.23986 | -1.51145 | -0.28722 | -0.25824 | -0.2963 | 0.82037 | 0.77907 | 0.51751 | 0.53632 | 2 | 2.93229 | 0.55262 | 0.9278 | 0.70475 | Not Significant | NS | | #N/A |
| 2631 | A0A5F9CVG0 | #N/A | KIF13B | RABBIT | #N/A | #N/A | #N/A | -0.05764 | #N/A | 1.03691 | #N/A | #N/A | #N/A | -1.73432 | -0.29107 | -0.12128 | -0.20168 | 0.92317 | 0.85017 | 0.54539 | 0.55382 | 1 | 2.93229 | 0.55262 | 3.83984 | 1.388575 | Not Significant | NS | | #N/A |
| 2007 | G1SWP6 | #N/A | CDHR4 | RABBIT | #N/A | 0.19782 | #N/A | #N/A | #N/A | 0.00694 | -0.19925 | #N/A | #N/A | #N/A | -0.29397 | -1.64637 | -0.37149 | 0.34749 | 0.72942 | 0.34597 | 0.5223 | 1 | 2.93229 | 0.55262 | 0.02126 | 0.417489 | Not Significant | NS | | #N/A |
| 1155 | G1SHH0 | Ubiquitin conjugation factor E4 OS=Oryctolagus cu | UBE4A | RABBIT | #N/A | #N/A | #N/A | -0.0819 | #N/A | 0 | #N/A | -1.57805 | 0.00963 | 0.06069 | -0.29503 | -0.32937 | -0.34131 | 0.76353 | 0.74463 | 0.5029 | 0.52866 | 3 | 2.93229 | 0.55262 | 0.64191 | 0.597771 | Not Significant | NS | | #N/A |
| 146 | A0A5F9C7Q4 | Stress-induced-phosphoprotein 1 OS=Oryctolagus | STIP1 | RABBIT | 0.4365 | -0.35032 | 0.17065 | #N/A | 0.8888 | -0.0099 | #N/A | #N/A | #N/A | #N/A | -0.29631 | -0.51187 | -0.41455 | 0.64404 | 0.69305 | 0.46712 | 0.51042 | 3 | 2.93229 | 0.55262 | 0.26808 | 0.408722 | Not Significant | NS | | #N/A |
| 1337 | G1U1H4 | Ras-related GTP-binding protein OS=Oryctolagus cu | RRAGB | RABBIT | 0.12225 | #N/A | #N/A | #N/A | #N/A | #N/A | #N/A | -0.17495 | #N/A | #N/A | -0.2972 | #N/A | -0.2827 | #N/A | 0.79618 | #N/A | 0.5392 | 0 | 2.93229 | 0.55262 | #N/A | 0.552617 | Not Significant | NS | | #N/A |

| | | | | | | | | | | | | | | | | | | | | | | | | | | | | | |
|---|---|---|---|---|---|---|---|---|---|---|---|---|---|---|---|---|---|---|---|---|---|---|---|---|---|---|---|---|---|
| 2770 | G1U7F1 | #N/A | #N/A | RABBIT | #N/A | #N/A | #N/A | 0.08762 | #N/A | #N/A | #N/A | -0.20962 | #N/A | #N/A | -0.29724 | #N/A | -0.28274 | #N/A | 0.79616 | #N/A | 0.5392 | 0 | 2.93229 | 0.55262 | #N/A | 0.552617 | Not Significant | NS | #N/A |
| 2781 | P58772 | #N/A | TPM1 TPM | RABBIT | #N/A | #N/A | #N/A | #N/A | 0.18409 | #N/A | 1.07825 | -0.26184 | -1.15961 | #N/A | -0.29849 | -0.22953 | -0.28157 | 0.83979 | 0.7897 | 0.52431 | 0.53858 | 2 | 2.93229 | 0.55262 | 1.26831 | 0.842822 | Not Significant | NS | #N/A |
| 1058 | Q28733 | Titin (Fragment) OS=Oryctolagus cuniculus OX=998 | #N/A | RABBIT | 1.39403 | 0.50692 | -1.35911 | #N/A | #N/A | #N/A | 0 | #N/A | -0.23625 | #N/A | -0.29873 | -0.2842 | -0.33688 | 0.79474 | 0.74781 | 0.51196 | 0.52913 | 3 | 2.93229 | 0.55262 | 1.32584 | 0.943642 | Not Significant | NS | #N/A |
| 1838 | A0A5F9CJH2 | #N/A | #N/A | RABBIT | #N/A | 0.24861 | #N/A | #N/A | #N/A | #N/A | #N/A | #N/A | #N/A | -0.05079 | -0.2994 | #N/A | -0.28479 | #N/A | 0.79473 | #N/A | 0.53895 | 0 | 2.93229 | 0.55262 | #N/A | 0.552617 | Not Significant | NS | #N/A |
| 301 | G1TCR0 | Ketimine reductase mu-crystallin OS=Oryctolagus c | CRYM | RABBIT | 0.36583 | 0.44706 | -0.90344 | 1.47722 | -0.46789 | 0.62619 | -0.3863 | -1.25802 | 0.68863 | -0.24924 | -0.2995 | -0.54794 | -0.56812 | 0.59869 | 0.58144 | 0.45077 | 0.47236 | 8 | 2.93229 | 0.55262 | 0.74693 | 0.694813 | Not Significant | NS | #N/A |
| 2048 | A0A5F9D345 | #N/A | #N/A | RABBIT | #N/A | -0.47446 | 0.09683 | 0.01618 | #N/A | -1.91388 | 0.70266 | 1.24004 | -3.6078 | 1.4781 | -0.29969 | -0.22363 | -0.26253 | 0.83046 | 0.79886 | 0.52093 | 0.53955 | 6 | 2.93229 | 0.55262 | 3.36731 | 2.443303 | Not Significant | NS | #N/A |
| 2245 | G1SKY7 | #N/A | TARDBP | RABBIT | #N/A | #N/A | -0.74465 | 1.09648 | #N/A | 0 | -1.67026 | 0.55144 | -0.97614 | 1.46391 | -0.30212 | -0.28903 | -0.33127 | 0.78416 | 0.74902 | 0.50854 | 0.52913 | 5 | 2.93229 | 0.55262 | 1.56099 | 1.188229 | Not Significant | NS | #N/A |
| 1137 | A0A5F9CLA4 | urocanate hydratase OS=Oryctolagus cuniculus OX | UROC1 | RABBIT | 0.76887 | -0.25588 | 0.08783 | #N/A | 0.74239 | #N/A | 0.52447 | #N/A | 0 | -0.42755 | -0.3035 | -0.80472 | -0.66415 | 0.45752 | 0.52543 | 0.39561 | 0.45323 | 5 | 2.93229 | 0.55262 | 0.24383 | 0.35798 | Not Significant | NS | #N/A |
| 2870 | P9WKA5 | #N/A | #N/A | MT | #N/A | #N/A | #N/A | #N/A | 0.14547 | #N/A | -0.15885 | #N/A | #N/A | #N/A | -0.30432 | #N/A | -0.28947 | #N/A | 0.79148 | #N/A | 0.53886 | 0 | 2.93229 | 0.55262 | #N/A | 0.552617 | Not Significant | NS | #N/A |
| 2252 | A0A5F9D729 | #N/A | SRC | RABBIT | #N/A | #N/A | #N/A | 0.04603 | #N/A | 2.9398 | -2.72381 | -2.44584 | -0.10367 | 1.04169 | -0.3044 | -0.11638 | -0.14804 | 0.91296 | 0.88653 | 0.54361 | 0.56421 | 4 | 2.93229 | 0.55262 | 5.70145 | 3.523549 | Not Significant | NS | #N/A |
| 2539 | G1U0I7 | #N/A | ATXN2L | RABBIT | #N/A | #N/A | #N/A | 0.23957 | #N/A | 0 | #N/A | -0.09156 | -0.21609 | 0.04232 | -0.3059 | -2.3915 | -0.51728 | 0.09661 | 0.62368 | 0.1866 | 0.48561 | 3 | 2.93229 | 0.55262 | 0.01309 | 0.279774 | Not Significant | NS | #N/A |
| 295 | A0A5F9DC49 | Dihydrolipoyllysine-residue succinyltransferase cor | DLST | RABBIT | -0.13297 | -0.73652 | 0.75469 | -0.38943 | 1.03749 | -2.97856 | -0.34115 | 1.38575 | 0.14463 | 0.78517 | -0.30749 | -0.37226 | -0.41143 | 0.71936 | 0.68871 | 0.49014 | 0.50985 | 8 | 2.93229 | 0.55262 | 1.70564 | 1.396373 | Not Significant | NS | #N/A |
| 1455 | G1SS64 | Elongator complex protein 5 OS=Oryctolagus cunic | ELP5 | RABBIT | #N/A | 0.19782 | #N/A | #N/A | #N/A | #N/A | 0.14134 | -0.1294 | 0.09615 | -0.55189 | -0.30877 | -0.87145 | -0.48523 | 0.44764 | 0.64491 | 0.39001 | 0.49451 | 3 | 2.93229 | 0.55262 | 0.10043 | 0.323945 | Not Significant | NS | #N/A |
| 1613 | G1TEJ9 | dolichyl-phosphate-mannose--protein mannosyltra | TMTC2 | RABBIT | #N/A | #N/A | 0.17065 | 0.26941 | #N/A | #N/A | -0.06053 | -0.03758 | -0.17051 | #N/A | -0.30957 | -4.79999 | -0.64588 | 0.01721 | 0.54252 | 0.10978 | 0.46162 | 3 | 2.93229 | 0.55262 | 0.00499 | 0.275679 | Not Significant | NS | #N/A |
| 254 | A0A5F9DBD2 | Aldehyde dehydrogenase, mitochondrial OS=Oryct | ALDH2 | RABBIT | -0.21826 | 2.35811 | 0.99625 | -0.10626 | -0.69285 | -0.40716 | -0.07824 | 0.78917 | 0.04862 | 0.43046 | -0.31083 | -0.53074 | -0.55802 | 0.61001 | 0.58808 | 0.45404 | 0.47237 | 8 | 2.93229 | 0.55262 | 0.85746 | 0.775692 | Not Significant | NS | #N/A |
| 614 | G1TBW9 | Dynein light chain OS=Oryctolagus cuniculus OX=9 | DYNLL2 | RABBIT | -0.10829 | 0.15186 | 0.195 | 0.20996 | -0.01488 | 0.15427 | 0.0015 | -0.03564 | -0.81559 | -0.42763 | -0.31135 | -1.66381 | -1.06846 | 0.13472 | 0.30834 | 0.21791 | 0.35174 | 8 | 2.93229 | 0.55262 | 0.08755 | 0.212288 | Not Significant | NS | #N/A |
| 85 | G1SR29 | H(+)-transporting two-sector ATPase OS=Oryctolag | ATP6V1A | RABBIT | 0.15181 | -0.04711 | 0.33047 | -0.51868 | 0.4567 | -1.09437 | 0.16587 | 0.3628 | -0.14015 | -0.49135 | -0.31408 | -1.0149 | -0.87321 | 0.33986 | 0.40132 | 0.34272 | 0.40169 | 8 | 2.93229 | 0.55262 | 0.23943 | 0.323432 | Not Significant | NS | #N/A |
| 736 | A0A5F9CD94 | Astrotactin 1 OS=Oryctolagus cuniculus OX=9986 ( | ASTN1 | RABBIT | 1.05487 | 0.02168 | -0.01459 | 1.57492 | -2.97616 | #N/A | 2.00116 | #N/A | 0.17614 | -3.32384 | -0.31432 | -0.20262 | -0.24014 | 0.84613 | 0.81564 | 0.52558 | 0.5433 | 6 | 2.93229 | 0.55262 | 4.51229 | 3.212412 | Not Significant | NS | #N/A |
| 2661 | G1TYF8 | #N/A | #N/A | RABBIT | #N/A | #N/A | #N/A | 0.42259 | 0.12288 | 0.40649 | -0.17842 | -0.20063 | #N/A | -0.19998 | -0.31587 | -1.30019 | -0.69033 | 0.26339 | 0.51243 | 0.30573 | 0.44921 | 4 | 2.93229 | 0.55262 | 0.0787 | 0.27916 | Not Significant | NS | #N/A |
| 2189 | G1T0M7 | #N/A | TMEM163 | RABBIT | #N/A | #N/A | 1.02624 | #N/A | -1.51012 | 0.91283 | 0 | 1.2391 | -2.49245 | -2.45081 | -0.31632 | -0.20975 | -0.25192 | 0.84214 | 0.80751 | 0.52455 | 0.54212 | 5 | 2.93229 | 0.55262 | 3.24913 | 2.252325 | Not Significant | NS | #N/A |
| 1154 | A0A5F9DDF0 | U2A'/phosphoprotein 32 family A C-terminal domai | #N/A | RABBIT | 0.09428 | -0.69106 | 0.07837 | 1.27223 | 0.91337 | #N/A | 0 | 1.47731 | -1.29643 | -0.11276 | -0.31641 | -0.4986 | -0.52939 | 0.63334 | 0.60816 | 0.46211 | 0.47919 | 7 | 2.93229 | 0.55262 | 0.89489 | 0.793841 | Not Significant | NS | #N/A |
| 2439 | A0A5F9CVG2 | #N/A | #N/A | RABBIT | #N/A | #N/A | 0.70088 | -0.79282 | 0.58187 | 0.102 | #N/A | -0.543 | 0 | -0.17454 | -0.3172 | -0.72976 | -0.64984 | 0.49827 | 0.53415 | 0.41326 | 0.45675 | 5 | 2.93229 | 0.55262 | 0.32387 | 0.408431 | Not Significant | NS | #N/A |
| 2710 | A5U293 | #N/A | #N/A | MT | #N/A | #N/A | #N/A | 0.13004 | #N/A | #N/A | #N/A | #N/A | #N/A | -0.18767 | -0.31771 | #N/A | -0.30221 | #N/A | 0.78266 | #N/A | 0.53764 | 0 | 2.93229 | 0.55262 | #N/A | 0.552617 | Not Significant | NS | #N/A |
| 1374 | Q95216 | Helicase-like transcription factor OS=Oryctolagus c | HLTF RUS | RABBIT | 0.01166 | #N/A | #N/A | #N/A | #N/A | #N/A | 0.03181 | #N/A | #N/A | -0.64525 | -0.31838 | -0.54299 | -0.37904 | 0.68332 | 0.72424 | 0.4792 | 0.52086 | 1 | 2.93229 | 0.55262 | 0.2292 | 0.470371 | Not Significant | NS | #N/A |
| 2733 | G1SU44 | #N/A | ZSCAN25 | RABBIT | #N/A | #N/A | #N/A | 0.13084 | #N/A | #N/A | #N/A | #N/A | -0.18848 | #N/A | -0.31933 | #N/A | -0.30374 | #N/A | 0.7816 | #N/A | 0.53732 | 0 | 2.93229 | 0.55262 | #N/A | 0.552617 | Not Significant | NS | #N/A |
| 1907 | A0A5F9CMX3 | #N/A | DIP2B | RABBIT | #N/A | 0.43723 | 0.14219 | 0.36243 | -0.11913 | 0.48362 | 0 | 0.71967 | -0.83051 | -0.95639 | -0.3224 | -0.80813 | -0.74838 | 0.44561 | 0.47158 | 0.38942 | 0.43055 | 7 | 2.93229 | 0.55262 | 0.35369 | 0.412418 | Not Significant | NS | #N/A |
| 2002 | A0A5F9DW02 | #N/A | GSTA5 | RABBIT | #N/A | -0.6548 | #N/A | #N/A | 0.09543 | 0.40881 | -2.82124 | -0.17318 | 0.17561 | #N/A | -0.32281 | -0.28143 | -0.33394 | 0.79234 | 0.7483 | 0.5115 | 0.52913 | 4 | 2.93229 | 0.55262 | 1.75419 | 1.245936 | Not Significant | NS | #N/A |
| 552 | G1SPH4 | MOB-like protein phocein OS=Oryctolagus cuniculu | #N/A | RABBIT | 0.12133 | #N/A | -0.04944 | #N/A | 0.09158 | 0.207 | #N/A | -0.16933 | #N/A | -0.84977 | -0.32519 | -1.03662 | -0.70524 | 0.35846 | 0.50366 | 0.35078 | 0.44513 | 4 | 2.93229 | 0.55262 | 0.14761 | 0.318924 | Not Significant | NS | #N/A |
| 1618 | G1SGZ7 | Pinin OS=Oryctolagus cuniculus OX=9986 GN=PNN | PNN | RABBIT | -0.29861 | 1.0019 | #N/A | 0.02558 | #N/A | #N/A | -0.08322 | #N/A | #N/A | #N/A | -0.32617 | -0.41727 | -0.39386 | 0.71701 | 0.71013 | 0.48961 | 0.51642 | 2 | 2.93229 | 0.55262 | 0.45827 | 0.514361 | Not Significant | NS | #N/A |
| 771 | U3KM36 | Ubiquitin carboxyl-terminal hydrolase OS=Oryctola | USP15 | RABBIT | 0.33761 | 0.04061 | -1.52589 | 0.93251 | -0.11547 | -2.97755 | 0.56744 | -0.86849 | 1.20981 | 0.10209 | -0.32721 | -0.39183 | -0.43355 | 0.70541 | 0.67304 | 0.48539 | 0.50369 | 8 | 2.93229 | 0.55262 | 1.74344 | 1.424038 | Not Significant | NS | #N/A |
| 2090 | G1SYS2 | #N/A | MSTO1 | RABBIT | #N/A | 0.19782 | #N/A | #N/A | #N/A | 0.51821 | #N/A | #N/A | -0.77743 | #N/A | -0.32743 | -0.29181 | -0.33802 | 0.81925 | 0.75262 | 0.51745 | 0.53054 | 1 | 2.93229 | 0.55262 | 0.83935 | 0.625534 | Not Significant | NS | #N/A |
| 2186 | G1TXG3 | #N/A | AGAP3 | RABBIT | #N/A | #N/A | -0.12285 | 0.60791 | #N/A | 0.69563 | #N/A | -0.31309 | 0.24872 | -0.97531 | -0.32854 | -0.5605 | -0.53754 | 0.60505 | 0.60771 | 0.45322 | 0.47919 | 4 | 2.93229 | 0.55262 | 0.45811 | 0.498084 | Not Significant | NS | #N/A |
| 2301 | G1SSG3 | #N/A | CPNE6 | RABBIT | #N/A | #N/A | 0.41385 | #N/A | #N/A | 0.87644 | #N/A | -0.30756 | #N/A | -0.31437 | -0.32901 | -0.41562 | -0.39547 | 0.71804 | 0.70902 | 0.48992 | 0.51642 | 2 | 2.93229 | 0.55262 | 0.46999 | 0.519114 | Not Significant | NS | #N/A |
| 2311 | A0A5F9CT76 | #N/A | SLC26A3 | RABBIT | #N/A | #N/A | 1.02792 | -0.49765 | #N/A | #N/A | #N/A | -0.06437 | #N/A | #N/A | -0.3295 | -0.2494 | -0.31974 | 0.8444 | 0.76543 | 0.52496 | 0.53348 | 1 | 2.93229 | 0.55262 | 1.16369 | 0.708016 | Not Significant | NS | #N/A |
| 2032 | G1TQX1 | #N/A | SRP14 | RABBIT | #N/A | #N/A | #N/A | 0.08396 | #N/A | 0.52559 | -0.27682 | #N/A | -1.23529 | 0 | -0.33059 | -0.40046 | -0.39912 | 0.71563 | 0.70378 | 0.48919 | 0.51495 | 3 | 2.93229 | 0.55262 | 0.54517 | 0.548851 | Not Significant | NS | #N/A |
| 2259 | G1SFS8 | #N/A | SND1 | RABBIT | #N/A | #N/A | #N/A | -0.04156 | #N/A | -2.27176 | 0.40055 | 0.18014 | -0.15678 | -0.01608 | -0.33123 | -0.27943 | -0.31707 | 0.79377 | 0.76052 | 0.51175 | 0.53222 | 4 | 2.93229 | 0.55262 | 1.17094 | 0.909397 | Not Significant | NS | #N/A |
| 2369 | A0A375YEK3 | #N/A | #N/A | MT | #N/A | #N/A | 0.25155 | #N/A | #N/A | #N/A | -0.0809 | #N/A | #N/A | #N/A | -0.33245 | #N/A | -0.31623 | #N/A | 0.773 | #N/A | 0.53484 | 0 | 2.93229 | 0.55262 | #N/A | 0.552617 | Not Significant | NS | #N/A |
| 2034 | A0A8J2E3R8 | #N/A | #N/A | RABBIT | #N/A | 0.17248 | #N/A | -0.12654 | 0.50514 | -0.54685 | #N/A | #N/A | 0.02534 | 0.07325 | -0.33312 | -1.23325 | -0.74875 | 0.285 | 0.47864 | 0.31704 | 0.43349 | 4 | 2.93229 | 0.55262 | 0.10944 | 0.296901 | Not Significant | NS | #N/A |
| 2401 | G1TRZ2 | #N/A | LAMP1 | RABBIT | #N/A | #N/A | 0.18708 | #N/A | #N/A | 0.30275 | 0.6733 | -0.0808 | -1.49323 | -0.13961 | -0.3346 | -0.37234 | -0.38727 | 0.72853 | 0.71017 | 0.49212 | 0.51642 | 4 | 2.93229 | 0.55262 | 0.67299 | 0.622071 | Not Significant | NS | #N/A |
| 1595 | G1SVQ9 | Cilia and flagella associated protein 58 OS=Oryctol | CFAP58 | RABBIT | 0.17392 | #N/A | #N/A | #N/A | #N/A | #N/A | #N/A | #N/A | -0.16226 | #N/A | -0.33618 | #N/A | -0.31977 | #N/A | 0.77057 | #N/A | 0.53484 | 0 | 2.93229 | 0.55262 | #N/A | 0.552617 | Not Significant | NS | #N/A |
| 328 | A0A5F9DDV7 | Filamin B OS=Oryctolagus cuniculus OX=9986 GN= | FLNB | RABBIT | -0.34056 | -1.97551 | -0.34477 | 0.29459 | 0.40829 | -3.52573 | #N/A | 0.63438 | #N/A | 0.7066 | -0.33665 | -0.28806 | -0.33428 | 0.78299 | 0.74588 | 0.50847 | 0.52878 | 6 | 2.93229 | 0.55262 | 2.56095 | 1.901656 | Not Significant | NS | #N/A |
| 2788 | A0A5F9CZR1 | #N/A | RAB2B | RABBIT | #N/A | #N/A | #N/A | #N/A | 0.16207 | #N/A | -0.17545 | #N/A | #N/A | #N/A | -0.33752 | #N/A | -0.32105 | #N/A | 0.76969 | #N/A | 0.53484 | 0 | 2.93229 | 0.55262 | #N/A | 0.552617 | Not Significant | NS | #N/A |
| 1778 | G1SK75 | #N/A | CPPED1 | RABBIT | #N/A | -0.00717 | #N/A | 0.36526 | 0.1916 | #N/A | 0.34031 | -0.47459 | -0.33273 | #N/A | -0.3389 | -1.23954 | -0.75977 | 0.2829 | 0.47243 | 0.31631 | 0.43108 | 4 | 2.93229 | 0.55262 | 0.11213 | 0.298451 | Not Significant | NS | #N/A |
| 2005 | A0A5F9CPM2 | #N/A | COL4A5 | RABBIT | #N/A | #N/A | #N/A | #N/A | 0.11216 | -0.02522 | -0.41076 | -0.83604 | 0.10876 | 0.02522 | -0.33977 | -0.7867 | -0.54537 | 0.47545 | 0.6026 | 0.40372 | 0.47806 | 4 | 2.93229 | 0.55262 | 0.15544 | 0.323442 | Not Significant | NS | #N/A |
| 259 | G1T2L1 | Proteasome subunit alpha type OS=Oryctolagus cu | #N/A | RABBIT | 0.29541 | 0.19782 | -0.02064 | 0.10396 | 0.66128 | #N/A | 0.67084 | -0.31343 | -0.67299 | -0.05565 | -0.34037 | -1.20638 | -0.94577 | 0.26686 | 0.36671 | 0.30628 | 0.38387 | 7 | 2.93229 | 0.55262 | 0.1769 | 0.287822 | Not Significant | NS | #N/A |
| 2040 | O53507 | #N/A | #N/A | MT | #N/A | #N/A | 0.25615 | #N/A | #N/A | #N/A | #N/A | #N/A | -0.0855 | #N/A | -0.34166 | #N/A | -0.32498 | #N/A | 0.76699 | #N/A | 0.53414 | 0 | 2.93229 | 0.55262 | #N/A | 0.552617 | Not Significant | NS | #N/A |
| 2335 | A0A5F9DMB1 | #N/A | DSTYK | RABBIT | #N/A | #N/A | -0.00293 | 0.14513 | #N/A | #N/A | -0.65349 | 0.10921 | #N/A | #N/A | -0.34324 | -0.88356 | -0.54981 | 0.47014 | 0.60642 | 0.4013 | 0.47901 | 2 | 2.93229 | 0.55262 | 0.15091 | 0.389727 | Not Significant | NS | #N/A |
| 2110 | G1SEF2 | #N/A | CC2D1B | RABBIT | #N/A | 0.19782 | #N/A | #N/A | 0.22791 | #N/A | -0.13515 | #N/A | #N/A | #N/A | -0.34801 | -13.3535 | -0.44259 | 0.04759 | 0.68132 | 0.14885 | 0.50745 | 1 | 2.93229 | 0.55262 | 0.00045 | 0.412199 | Not Significant | NS | #N/A |
| 1473 | A0A5F9DPQ6 | NAC-A/B domain-containing protein OS=Oryctolagu | #N/A | RABBIT | -0.08119 | 0.16051 | 0.60285 | 0.31625 | 0.32941 | #N/A | -0.17559 | 0.14728 | -0.30433 | 0 | -0.34873 | -2.26372 | -1.16151 | 0.05801 | 0.27259 | 0.15468 | 0.33035 | 7 | 2.93229 | 0.55262 | 0.05274 | 0.200315 | Not Significant | NS | #N/A |
| 1157 | G1TS96 | DBIRD complex subunit ZNF326 OS=Oryctolagus cu | ZNF326 | RABBIT | 0.34042 | #N/A | #N/A | #N/A | #N/A | 0.9709 | 0 | #N/A | -0.82168 | -0.18624 | -0.34967 | -0.42145 | -0.42109 | 0.7018 | 0.68852 | 0.48408 | 0.50985 | 3 | 2.93229 | 0.55262 | 0.55072 | 0.551657 | Not Significant | NS | #N/A |
| 187 | G1T4X8 | Proteasome subunit beta OS=Oryctolagus cuniculu | PSMB2 | RABBIT | 0.61394 | 0.40115 | -0.15243 | -0.83219 | 0.58146 | #N/A | 0.61413 | -0.38797 | -1.13826 | 0 | -0.35041 | -0.7809 | -0.75514 | 0.46045 | 0.46769 | 0.39639 | 0.42854 | 7 | 2.93229 | 0.55262 | 0.44745 | 0.478499 | Not Significant | NS | #N/A |
| 229 | G1SZ14 | Proteasome subunit alpha type-3 OS=Oryctolagus c | PSMA3 | RABBIT | 0.49226 | 0.16139 | 0.2062 | -0.05764 | 0.70267 | #N/A | 0.70748 | -0.09966 | -0.65765 | -0.14865 | -0.35059 | -1.20802 | -0.96214 | 0.26626 | 0.35882 | 0.30603 | 0.37905 | 7 | 2.93229 | 0.55262 | 0.18717 | 0.295063 | Not Significant | NS | #N/A |
| 2366 | A0A5F9DHM0 | #N/A | THSD7A | RABBIT | #N/A | #N/A | 0.0283 | #N/A | 0.53893 | #N/A | 0.19886 | #N/A | 0 | -0.40061 | -0.35086 | -1.18268 | -0.67258 | 0.32213 | 0.52653 | 0.33441 | 0.45323 | 3 | 2.93229 | 0.55262 | 0.10561 | 0.326564 | Not Significant | NS | #N/A |
| 199 | G1TBC4 | V-type proton ATPase subunit H OS=Oryctolagus cu | ATP6V1H | RABBIT | 0.61288 | -0.93464 | 0.09338 | 0.01963 | 0.56493 | -0.91361 | 0.22483 | 0.17416 | -0.45322 | -0.44431 | -0.35366 | -1.00604 | -0.914 | 0.34385 | 0.38043 | 0.34542 | 0.39063 | 8 | 2.93229 | 0.55262 | 0.30895 | 0.374307 | Not Significant | NS | #N/A |
| 1144 | A0A1E3T7J7 | Iron-regulated universal stress protein family protei | #N/A | MT | 0.57371 | #N/A | -0.15483 | 0.22979 | #N/A | #N/A | 0.0029 | -0.41464 | #N/A | -0.0029 | -0.35444 | -1.40785 | -0.80803 | 0.23194 | 0.44591 | 0.29043 | 0.41978 | 4 | 2.93229 | 0.55262 | 0.09507 | 0.28861 | Not Significant | NS | #N/A |
| 2755 | G1SU72 | #N/A | SEMA3G | RABBIT | #N/A | #N/A | #N/A | 0.27192 | #N/A | -0.18485 | -0.08177 | 0.0174 | #N/A | #N/A | -0.35499 | -3.03987 | -0.53301 | 0.09332 | 0.61716 | 0.18402 | 0.48339 | 2 | 2.93229 | 0.55262 | 0.01023 | 0.332683 | Not Significant | NS | #N/A |
| 2694 | G1SPV8 | #N/A | IPO13 | RABBIT | #N/A | #N/A | #N/A | 0.27713 | #N/A | #N/A | 0 | #N/A | -0.15772 | #N/A | -0.35599 | -2.60632 | -0.45107 | 0.23323 | 0.67569 | 0.29088 | 0.50502 | 1 | 2.93229 | 0.55262 | 0.01244 | 0.415247 | Not Significant | NS | #N/A |
| 2136 | G1STH1 | #N/A | RPS3A | RABBIT | #N/A | #N/A | 0.45561 | -0.13675 | #N/A | #N/A | -0.47269 | #N/A | #N/A | 0.07911 | -0.35622 | -0.88006 | -0.56681 | 0.47165 | 0.59566 | 0.40175 | 0.47479 | 2 | 2.93229 | 0.55262 | 0.16384 | 0.394972 | Not Significant | NS | #N/A |
| 1800 | A0A5F9DEH9 | #N/A | #N/A | RABBIT | #N/A | 0.19782 | #N/A | #N/A | #N/A | #N/A | 0.10143 | #N/A | -0.41968 | #N/A | -0.35695 | -0.79095 | -0.43611 | 0.57398 | 0.68563 | 0.44118 | 0.50924 | 1 | 2.93229 | 0.55262 | 0.13578 | 0.446612 | Not Significant | NS | #N/A |
| 519 | G1T6S6 | V-type proton ATPase subunit F OS=Oryctolagus cu | #N/A | RABBIT | -0.50989 | 0.74321 | -0.25939 | -0.15406 | 0.83687 | -1.15758 | 0.52641 | 0.03206 | 0.18474 | -0.72664 | -0.35955 | -0.86728 | -0.8358 | 0.41104 | 0.42116 | 0.37603 | 0.4086 | 8 | 2.93229 | 0.55262 | 0.42967 | 0.462646 | Not Significant | NS | #N/A |
| 1504 | A0A5F9DG36 | Apolipoprotein B OS=Oryctolagus cuniculus OX=99 | APOB | RABBIT | 0.59346 | 0.12577 | #N/A | #N/A | #N/A | #N/A | #N/A | #N/A | 0 | #N/A | -0.35962 | -0.88788 | -0.44271 | 0.53776 | 0.68124 | 0.42838 | 0.50745 | 1 | 2.93229 | 0.55262 | 0.10936 | 0.439896 | Not Significant | NS | #N/A |
| 1100 | A0A5F9CGI0 | SOSS complex subunit B2 OS=Oryctolagus cunicul | #N/A | RABBIT | 0.41111 | 0.11828 | #N/A | 0.21193 | #N/A | #N/A | -0.21409 | -0.20621 | 0.07954 | #N/A | -0.3607 | -2.7841 | -0.88658 | 0.04961 | 0.40501 | 0.14957 | 0.40358 | 4 | 2.93229 | 0.55262 | 0.02518 | 0.248279 | Not Significant | NS | #N/A |
| 1685 | G1T6X1 | #N/A | KRT15 | RABBIT | #N/A | 1.03518 | #N/A | 0.05341 | -0.3386 | #N/A | #N/A | #N/A | #N/A | -0.11105 | -0.36105 | -0.44184 | -0.42884 | 0.70179 | 0.68612 | 0.48408 | 0.50926 | 2 | 2.93229 | 0.55262 | 0.5008 | 0.531606 | Not Significant | NS | #N/A |
| 91 | G1SEJ4 | Vacuolar proton pump subunit B OS=Oryctolagus c | ATP6V1B2 | RABBIT | 0.47268 | 0.13403 | 0.23444 | -0.22447 | 0.4682 | -0.8502 | 0.38676 | 0.35771 | -0.2186 | -0.39858 | -0.36156 | -1.3507 | -1.08169 | 0.21375 | 0.30267 | 0.27684 | 0.34911 | 8 | 2.93229 | 0.55262 | 0.17914 | 0.279311 | Not Significant | NS | #N/A |
| 1259 | P9WI99 | Putative aminoglycoside phosphotransferase OS=M | #N/A | MT | 0.58856 | -0.49739 | 0.35745 | #N/A | #N/A | #N/A | 0 | -0.39794 | -0.45832 | 0.00754 | -0.36172 | -1.15351 | -0.84988 | 0.30085 | 0.4203 | 0.32455 | 0.40859 | 5 | 2.93229 | 0.55262 | 0.16858 | 0.310542 | Not Significant | NS | #N/A |
| 932 | A0A5F9D4W9 | non-specific serine/threonine protein kinase OS=Or | AKT3 | RABBIT | 0.24735 | 1.05734 | -0.5647 | 0.70148 | -0.24907 | 0.64264 | -0.32329 | -0.33881 | 0.59938 | -1.19868 | -0.36223 | -0.79813 | -0.79041 | 0.44784 | 0.44609 | 0.39001 | 0.41978 | 8 | 2.93229 | 0.55262 | 0.51495 | 0.525053 | Not Significant | NS | #N/A |
| 2274 | G1U204 | #N/A | TMEM143 | RABBIT | #N/A | #N/A | 0.17065 | -0.21247 | 0.8515 | #N/A | #N/A | #N/A | 0.14015 | -0.32573 | -0.36268 | -0.82883 | -0.63672 | 0.46798 | 0.54808 | 0.40016 | 0.46295 | 3 | 2.93229 | 0.55262 | 0.22977 | 0.389352 | Not Significant | NS | #N/A |
| 1723 | A0A5F9CRX8 | #N/A | SYNE1 | RABBIT | #N/A | -1.32848 | #N/A | #N/A | 1.05843 | -4.01459 | 0.98617 | 0.84285 | -0.30704 | 0 | -0.36349 | -0.22 | -0.26625 | 0.83457 | 0.79683 | 0.52268 | 0.5392 | 5 | 2.93229 | 0.55262 | 3.90003 | 2.662612 | Not Significant | NS | #N/A |
| 922 | A0A5F9DF75 | Neuroligin 1 OS=Oryctolagus cuniculus OX=9986 G | NLGN1 | RABBIT | -0.05597 | 1.81773 | #N/A | 0.06655 | -0.97945 | #N/A | 0.06763 | -0.55568 | 0.33289 | -0.45242 | -0.36411 | -0.58634 | -0.61568 | 0.57902 | 0.55346 | 0.44327 | 0.46295 | 6 | 2.93229 | 0.55262 | 0.77124 | 0.699473 | Not Significant | NS | #N/A |
| 1732 | G1SR27 | #N/A | GRB2 | RABBIT | #N/A | 0.59761 | -0.7823 | 0.48513 | -0.12033 | #N/A | -0.74157 | 0.06139 | 0.10695 | -0.70809 | -0.36535 | -0.92627 | -0.82691 | 0.39004 | 0.42982 | 0.36581 | 0.41175 | 6 | 2.93229 | 0.55262 | 0.31116 | 0.390423 | Not Significant | NS | #N/A |
| 213 | P00489 | Glycogen phosphorylase, muscle form OS=Oryctol | PYGM | RABBIT | 0.04597 | 0.53254 | -0.30368 | 0.39468 | 0.13454 | -0.55933 | -0.61601 | -0.18712 | 0.37139 | -0.0343 | -0.36588 | -1.57548 | -1.16428 | 0.1538 | 0.26909 | 0.23444 | 0.32966 | 8 | 2.93229 | 0.55262 | 0.13483 | 0.246893 | Not Significant | NS | #N/A |
| 2222 | Q8HZX6 | #N/A | #N/A | RABBIT | #N/A | #N/A | 0.16035 | #N/A | 0.10795 | #N/A | #N/A | -0.05406 | -0.41044 | #N/A | -0.3664 | -2.03437 | -0.62681 | 0.17891 | 0.55865 | 0.25448 | 0.46519 | 2 | 2.93229 | 0.55262 | 0.03244 | 0.341689 | Not Significant | NS | #N/A |
| 33 | G1SIB9 | Alpha-1,4 glucan phosphorylase OS=Oryctolagus c | PYGB | RABBIT | 0.16038 | 0.30088 | 0.00437 | 0.09161 | 0.20195 | -0.48273 | 0.03305 | -0.16626 | -0.03305 | -0.42415 | -0.36647 | -3.19815 | -1.39615 | 0.01265 | 0.19037 | 0.09752 | 0.27623 | 8 | 2.93229 | 0.55262 | 0.03283 | 0.172245 | Not Significant | NS | #N/A |
| 1947 | A0A5F9DM60 | #N/A | SGSM1 | RABBIT | #N/A | 0.26771 | #N/A | 0.11726 | -0.08327 | 0.26361 | #N/A | -0.59274 | -0.46865 | #N/A | -0.36649 | -1.28203 | -0.81345 | 0.26909 | 0.443 | 0.30764 | 0.41812 | 4 | 2.93229 | 0.55262 | 0.12258 | 0.304482 | Not Significant | NS | #N/A |
| 403 | A0A5F9CNZ9 | cAMP-dependent protein kinase OS=Oryctolagus cu | PRKACB | RABBIT | 0.19438 | -0.21102 | 0.17262 | 0.33848 | 0.08154 | -0.00197 | -0.63335 | -0.54026 | 0.08421 | -0.17404 | -0.36828 | -2.16802 | -1.29878 | 0.06201 | 0.22075 | 0.15739 | 0.2975 | 8 | 2.93229 | 0.55262 | 0.07214 | 0.201014 | Not Significant | NS | #N/A |
| 2809 | G1SJB0 | #N/A | PPP1R12B | RABBIT | #N/A | #N/A | #N/A | #N/A | -0.01338 | #N/A | 0.03582 | #N/A | -0.80097 | #N/A | -0.36919 | -0.50946 | -0.42583 | 0.70003 | 0.6925 | 0.48373 | 0.51024 | 1 | 2.93229 | 0.55262 | 0.3501 | 0.501117 | Not Significant | NS | #N/A |
| 1979 | A0A5F9CQV7 | #N/A | #N/A | RABBIT | #N/A | #N/A | #N/A | 0.15585 | #N/A | #N/A | #N/A | #N/A | -0.21349 | #N/A | -0.36934 | #N/A | -0.35131 | #N/A | 0.74908 | #N/A | 0.52913 | 0 | 2.93229 | 0.55262 | #N/A | 0.552617 | Not Significant | NS | #N/A |
| 953 | G1SE74 | Transcription elongation factor spt6 OS=Oryctolagu | SUPT6H | RABBIT | 0.06684 | #N/A | #N/A | 0.3784 | #N/A | #N/A | #N/A | -0.11954 | -0.17876 | #N/A | -0.37177 | -2.34455 | -0.63878 | 0.14372 | 0.55145 | 0.22571 | 0.46295 | 2 | 2.93229 | 0.55262 | 0.02514 | 0.338731 | Not Significant | NS | #N/A |
| 408 | A0A5F9CYA0 | Microtubule-associated protein OS=Oryctolagus cu | MAPT | RABBIT | -0.67254 | 0.51633 | 0.44963 | -0.7868 | 0.24344 | -1.16842 | -0.15991 | 0.09554 | -1.18245 | 0.30581 | -0.3719 | -0.87797 | -0.85187 | 0.40556 | 0.41256 | 0.37324 | 0.40746 | 8 | 2.93229 | 0.55262 | 0.44857 | 0.476481 | Not Significant | NS | #N/A |
| 924 | A0A5F9CNQ8 | Keratin, type II cytoskeletal 5 OS=Oryctolagus cunic | KRT5 | RABBIT | -2.3646 | 3.04687 | -0.48798 | 0.51136 | 1.32837 | 1.76241 | -1.25866 | -0.85382 | -0.569 | 1.09087 | -0.37244 | -0.34552 | -0.39052 | 0.73861 | 0.70365 | 0.49518 | 0.51495 | 8 | 2.93229 | 0.55262 | 2.90483 | 2.273915 | Not Significant | NS | #N/A |
| 1201 | G1TFS5 | Single-stranded DNA-binding protein OS=Oryctolag | #N/A | RABBIT | -0.4746 | 0.19782 | 0.55668 | -0.41773 | 0.34912 | #N/A | -0.87726 | #N/A | #N/A | 0.2168 | -0.37249 | -0.82384 | -0.71441 | 0.44753 | 0.49546 | 0.39001 | 0.4412 | 5 | 2.93229 | 0.55262 | 0.29204 | 0.388367 | Not Significant | NS | #N/A |
| 2735 | G1T7P8 | #N/A | #N/A | RABBIT | #N/A | #N/A | #N/A | 0.1266 | #N/A | #N/A | #N/A | -0.49229 | #N/A | 0 | -0.37275 | -0.8743 | -0.45732 | 0.54263 | 0.67157 | 0.43035 | 0.50325 | 1 | 2.93229 | 0.55262 | 0.12118 | 0.4429 | Not Significant | NS | #N/A |
| 2011 | G1SQB1 | #N/A | MMUT | RABBIT | #N/A | 0.19782 | #N/A | #N/A | #N/A | #N/A | #N/A | -0.75907 | #N/A | 0.40651 | -0.3741 | -0.37061 | -0.39942 | 0.77406 | 0.71033 | 0.50625 | 0.51642 | 1 | 2.93229 | 0.55262 | 0.6793 | 0.584832 | Not Significant | NS | #N/A |
| 103 | Q8HZN0 | Serine/threonine-protein phosphatase OS=Oryctola | #N/A | RABBIT | -0.13414 | #N/A | 0.17164 | #N/A | 0.36121 | #N/A | #N/A | #N/A | 0 | -0.48336 | -0.37458 | -1.44547 | -0.73238 | 0.24409 | 0.49184 | 0.29685 | 0.43988 | 3 | 2.93229 | 0.55262 | 0.08059 | 0.313908 | Not Significant | NS | #N/A |
| 2141 | G1U765 | #N/A | SYT2 | RABBIT | #N/A | #N/A | 0.81931 | #N/A | -0.01338 | #N/A | -1.12664 | 1.44714 | #N/A | -0.23886 | -0.37575 | -0.36742 | -0.432 | 0.7377 | 0.681 | 0.4948 | 0.50745 | 3 | 2.93229 | 0.55262 | 1.25502 | 0.907829 | Not Significant | NS | #N/A |
| 72 | A0A5F9DGZ0 | Actin gamma 1 OS=Oryctolagus cuniculus OX=998 | #N/A | RABBIT | 1.06823 | 1.71842 | 2.11267 | -1.61528 | -1.13029 | 2.86952 | 2.87758 | -2.79618 | -1.6213 | -1.05656 | -0.37614 | -0.26738 | -0.30635 | 0.79594 | 0.76509 | 0.51222 | 0.53348 | 8 | 2.93229 | 0.55262 | 4.94746 | 3.768665 | Not Significant | NS | #N/A |
| 2606 | G1SN76 | #N/A | STK10 | RABBIT | #N/A | #N/A | #N/A | -0.1606 | #N/A | #N/A | #N/A | 0.17969 | -1.89481 | 0.10296 | -0.37679 | -0.27743 | -0.34599 | 0.8075 | 0.74362 | 0.5152 | 0.52816 | 2 | 2.93229 | 0.55262 | 1.38342 | 0.8895 | Not Significant | NS | #N/A |
| 567 | G1U522 | cAMP-dependent protein kinase type II-alpha regula | PRKAR2A | RABBIT | -0.07489 | 1.20005 | -0.12646 | 0.31302 | -0.85008 | -1.96768 | 0.47118 | 0.02219 | -0.17824 | 0.22832 | -0.37718 | -0.68793 | -0.71373 | 0.51095 | 0.49035 | 0.42044 | 0.43948 | 8 | 2.93229 | 0.55262 | 0.75152 | 0.698171 | Not Significant | NS | #N/A |
| 2114 | G1TAF9 | #N/A | BPTF | RABBIT | #N/A | 0.19782 | #N/A | #N/A | 0.29174 | #N/A | #N/A | -0.1368 | #N/A | #N/A | -0.38158 | -4.69133 | -0.48468 | 0.1337 | 0.65366 | 0.21787 | 0.49788 | 1 | 2.93229 | 0.55262 | 0.00441 | 0.413205 | Not Significant | NS | #N/A |
| 2740 | G1SU37 | #N/A | USP10 | RABBIT | #N/A | #N/A | #N/A | 0.37209 | #N/A | #N/A | 0 | -0.09089 | -0.81537 | 0.86331 | -0.38283 | -0.49806 | -0.47845 | 0.65267 | 0.64945 | 0.46988 | 0.49666 | 3 | 2.93229 | 0.55262 | 0.47265 | 0.512176 | Not Significant | NS | #N/A |
| 2145 | A0A5F9CE65 | #N/A | RELCH | RABBIT | #N/A | #N/A | -1.30048 | 1.09816 | 0.61538 | -0.95039 | #N/A | -0.69313 | #N/A | 0.90773 | -0.38295 | -0.40954 | -0.47126 | 0.70312 | 0.65193 | 0.4845 | 0.49788 | 4 | 2.93229 | 0.55262 | 1.31155 | 0.990527 | Not Significant | NS | #N/A |

| | | | | | | | | | | | | | | | | | | | | | | | | | | | | | | |
|---|---|---|---|---|---|---|---|---|---|---|---|---|---|---|---|---|---|---|---|---|---|---|---|---|---|---|---|---|---|---|
| 1276 | P35566 | MARCKS-related protein OS=Oryctolagus cuniculus | MARCKSL1 | RABBIT | -0.09329 | 0.64863 | 0.28514 | -0.72338 | 0.24871 | -0.90319 | -0.31945 | 0.19311 | -0.63336 | 0.10495 | -0.38475 | -1.23189 | -1.06436 | 0.25297 | 0.31011 | 0.30048 | 0.35209 | 8 | 2.93229 | 0.55262 | 0.24386 | 0.326678 | Not Significant | NS | | #N/A |
| 485 | P34826 | Elongation factor 1-beta OS=Oryctolagus cuniculus | EEF1B EF1 | RABBIT | 0.01039 | -0.21133 | 0.37075 | 0.0778 | 0.20184 | -1.09147 | 0.00128 | -0.19112 | 0.12794 | -0.3224 | -0.38505 | -1.64095 | -1.22018 | 0.13944 | 0.24806 | 0.22137 | 0.31646 | 8 | 2.93229 | 0.55262 | 0.13765 | 0.248952 | Not Significant | NS | | #N/A |
| 1688 | G1U9I8 | #N/A | KRT1 | RABBIT | #N/A | 0.8028 | -0.5136 | -0.26368 | 0.89783 | 0.96541 | -1.65375 | -1.12285 | 0 | 1.03571 | -0.38594 | -0.55764 | -0.602 | 0.59446 | 0.56066 | 0.44875 | 0.46562 | 7 | 2.93229 | 0.55262 | 1.06441 | 0.913314 | Not Significant | NS | | #N/A |
| 909 | A0A5F9DHL1 | AVL9 cell migration associated OS=Oryctolagus cui | AVL9 | RABBIT | 1.80174 | 1.88457 | 0.2378 | -1.46595 | -0.33669 | -0.06714 | -0.20267 | #N/A | #N/A | 0.38371 | -0.38633 | -0.44695 | -0.49932 | 0.67059 | 0.62962 | 0.47323 | 0.4889 | 6 | 2.93229 | 0.55262 | 1.40086 | 1.122402 | Not Significant | NS | | #N/A |
| 2559 | A0A5F9C7N7 | #N/A | XRCC6 | RABBIT | #N/A | #N/A | #N/A | -0.05764 | #N/A | -1.76753 | 0.09909 | #N/A | -0.25255 | 0.1398 | -0.38766 | -0.38572 | -0.41992 | 0.72543 | 0.68933 | 0.49085 | 0.50986 | 3 | 2.93229 | 0.55262 | 0.80805 | 0.681792 | Not Significant | NS | | #N/A |
| 81 | A0A5F9DCX4 | Tripeptidyl-peptidase 2 OS=Oryctolagus cuniculus ( | TPP2 | RABBIT | -0.00942 | -1.58039 | 0.55204 | -0.37276 | 1.35091 | -2.47751 | 0.84695 | -0.00538 | -0.38447 | 0.02108 | -0.38794 | -0.52382 | -0.57158 | 0.6146 | 0.57917 | 0.45457 | 0.47148 | 8 | 2.93229 | 0.55262 | 1.37123 | 1.151659 | Not Significant | NS | | #N/A |
| 58 | G1U9S2 | Albumin OS=Oryctolagus cuniculus OX=9986 GN=A | ALB | RABBIT | 0.25833 | 1.5601 | -1.16318 | 0.52326 | -0.26005 | -1.8451 | 2.49504 | 0.61123 | -1.94664 | -0.33752 | -0.38829 | -0.41247 | -0.46154 | 0.69083 | 0.65346 | 0.4817 | 0.49788 | 8 | 2.93229 | 0.55262 | 2.21545 | 1.769443 | Not Significant | NS | | #N/A |
| 2868 | A0A5F9C6G3 | #N/A | #N/A | RABBIT | #N/A | #N/A | #N/A | #N/A | 0.18789 | #N/A | -0.20128 | #N/A | #N/A | #N/A | -0.38917 | #N/A | -0.37018 | #N/A | 0.73637 | #N/A | 0.52494 | 0 | 2.93229 | 0.55262 | #N/A | 0.552617 | Not Significant | NS | | #N/A |
| 2540 | A0A5F9C2Q9 | #N/A | PRKCB | RABBIT | #N/A | #N/A | #N/A | -0.13735 | #N/A | 0.72089 | -0.76668 | 0.01535 | 0.3254 | -2.92974 | -0.38961 | -0.24531 | -0.2957 | 0.81829 | 0.77612 | 0.51745 | 0.53611 | 4 | 2.93229 | 0.55262 | 2.10214 | 1.446707 | Not Significant | NS | | #N/A |
| 283 | G1T918 | Proteasome subunit beta OS=Oryctolagus cuniculu | PSMB4 | RABBIT | 0.72835 | 0.19782 | -0.13068 | -0.08317 | 0.88699 | #N/A | 0.69036 | -0.32677 | -0.67256 | 0.02539 | -0.39076 | -1.1225 | -0.98051 | 0.29867 | 0.35011 | 0.32349 | 0.37401 | 7 | 2.93229 | 0.55262 | 0.26929 | 0.352939 | Not Significant | NS | | #N/A |
| 1288 | G1TK43 | Purine rich element binding protein G OS=Oryctolag | PURG | RABBIT | -0.31708 | 0.26961 | 0.77691 | -0.01038 | -1.0355 | -1.75325 | 0.11562 | 0.04472 | -0.04726 | -0.63364 | -0.39148 | -0.84545 | -0.84196 | 0.42242 | 0.41785 | 0.37981 | 0.40859 | 8 | 2.93229 | 0.55262 | 0.53601 | 0.540467 | Not Significant | NS | | #N/A |
| 1512 | G1SGH3 | Fructose-2,6-bisphosphatase TIGAR OS=Oryctolag | TIGAR | RABBIT | 1.1973 | 0.19843 | -0.7246 | -0.33225 | -0.014 | 0.99665 | -1.99664 | -1.55255 | 0.7508 | 0.16733 | -0.39186 | -0.56752 | -0.61335 | 0.58593 | 0.55219 | 0.44576 | 0.46295 | 8 | 2.93229 | 0.55262 | 1.1919 | 1.02043 | Not Significant | NS | | #N/A |
| 1490 | A0A5F9CHM4 | Cytochrome P450 family 20 subfamily A member 1 ( | CYP20A1 | RABBIT | #N/A | 0.38032 | #N/A | 0.30501 | #N/A | -0.43364 | #N/A | -0.42142 | 0.83846 | -0.1825 | -0.39244 | -0.86613 | -0.72404 | 0.43528 | 0.49274 | 0.38434 | 0.44001 | 4 | 2.93229 | 0.55262 | 0.27372 | 0.391692 | Not Significant | NS | | #N/A |
| 927 | G1SV75 | Acyl carrier protein OS=Oryctolagus cuniculus OX=9 | NDUFAB1 | RABBIT | 0.01166 | 1.59351 | -0.2661 | 0.8311 | -0.64686 | #N/A | -1.59409 | 0.73877 | 0.87653 | -0.38155 | -0.39475 | -0.57927 | -0.62361 | 0.58056 | 0.54694 | 0.44328 | 0.46295 | 7 | 2.93229 | 0.55262 | 1.03194 | 0.890432 | Not Significant | NS | | #N/A |
| 2363 | G1SGM5 | #N/A | BLTP3A | RABBIT | #N/A | #N/A | 0.17065 | 0.15975 | -0.22851 | #N/A | 1.23819 | #N/A | #N/A | -1.95977 | -0.39475 | -0.32792 | -0.4028 | 0.76452 | 0.70121 | 0.50299 | 0.51466 | 3 | 2.93229 | 0.55262 | 1.73895 | 1.152556 | Not Significant | NS | | #N/A |
| 467 | A0A5F9C7A3 | Huntingtin interacting protein 1 OS=Oryctolagus cu | HIP1 | RABBIT | 0.35848 | -3.08656 | 0.64383 | 0.70001 | -0.53719 | -0.94651 | 0.5557 | 0.61031 | -0.34681 | -3.77066 | -0.39531 | -0.36874 | -0.41661 | 0.72189 | 0.68502 | 0.49049 | 0.5091 | 8 | 2.93229 | 0.55262 | 2.87332 | 2.250852 | Not Significant | NS | | #N/A |
| 1898 | G1TP27 | #N/A | GEN1 | RABBIT | #N/A | 0.69661 | 0.0942 | #N/A | #N/A | 0 | #N/A | #N/A | #N/A | #N/A | -0.39541 | -0.75792 | -0.47694 | 0.58712 | 0.6587 | 0.44596 | 0.49906 | 1 | 2.93229 | 0.55262 | 0.18145 | 0.458227 | Not Significant | NS | | #N/A |
| 962 | G1U2J7 | DDHD domain containing 2 OS=Oryctolagus cunicu | DDHD2 | RABBIT | 0.47187 | 0.19066 | 0.17065 | -0.18302 | 0.8341 | #N/A | -0.28872 | 0.74128 | 0.38143 | -1.23216 | -0.39639 | -0.92874 | -0.8824 | 0.38393 | 0.39841 | 0.36226 | 0.40088 | 7 | 2.93229 | 0.55262 | 0.40481 | 0.448446 | Not Significant | NS | | #N/A |
| 2360 | A0A5F9DK72 | #N/A | SKIC2 | RABBIT | #N/A | #N/A | #N/A | 0.11892 | #N/A | #N/A | #N/A | -0.24093 | -1.24599 | 0.65297 | -0.39691 | -0.36181 | -0.41246 | 0.75214 | 0.69732 | 0.50035 | 0.51312 | 2 | 2.93229 | 0.55262 | 0.90254 | 0.694507 | Not Significant | NS | | #N/A |
| 2586 | A0A5F9DBG9 | #N/A | ERI3 | RABBIT | #N/A | #N/A | #N/A | 0.29396 | #N/A | #N/A | #N/A | #N/A | -0.20769 | 0 | -0.39781 | -2.21172 | -0.50265 | 0.27033 | 0.64206 | 0.30832 | 0.49343 | 1 | 2.93229 | 0.55262 | 0.02157 | 0.417568 | Not Significant | NS | | #N/A |
| 2205 | A0A5F9CY37 | #N/A | AP3M2 | RABBIT | #N/A | #N/A | 1.54222 | -0.54531 | #N/A | 0.74495 | -1.16799 | 1.04575 | -0.12095 | 0 | -0.3981 | -0.46858 | -0.51481 | 0.65909 | 0.62072 | 0.47102 | 0.48441 | 5 | 2.93229 | 0.55262 | 1.03118 | 0.854272 | Not Significant | NS | | #N/A |
| 118 | G1TAH7 | Transketolase OS=Oryctolagus cuniculus OX=9986 | TKT | RABBIT | 0.77471 | 1.132 | -0.84918 | 0.49832 | -0.06506 | -0.66235 | 0.03558 | -0.39779 | -0.03558 | 0.55926 | -0.39833 | -0.98468 | -0.94139 | 0.35362 | 0.36684 | 0.34889 | 0.38387 | 8 | 2.93229 | 0.55262 | 0.40911 | 0.447599 | Not Significant | NS | | #N/A |
| 109 | A0A5F9C3D7 | T-complex protein 1 subunit delta OS=Oryctolagus ( | CCT4 | RABBIT | 0.76579 | 0.03164 | #N/A | #N/A | #N/A | #N/A | 0 | #N/A | #N/A | #N/A | -0.39872 | -0.62712 | -0.46959 | 0.64341 | 0.6635 | 0.46712 | 0.50024 | 1 | 2.93229 | 0.55262 | 0.26948 | 0.480615 | Not Significant | NS | | #N/A |
| 61 | G1TUC8 | Actinin alpha 4 OS=Oryctolagus cuniculus OX=998 | ACTN4 | RABBIT | 0.53264 | 0.19782 | #N/A | #N/A | 0.67264 | #N/A | 0.77498 | -0.18867 | -0.30931 | -0.00262 | -0.39911 | -1.28094 | -0.93977 | 0.25641 | 0.37506 | 0.30236 | 0.38769 | 5 | 2.93229 | 0.55262 | 0.16642 | 0.309185 | Not Significant | NS | | #N/A |
| 158 | A0A5F9CLG6 | Neurocan OS=Oryctolagus cuniculus OX=9986 GN= | NCAN | RABBIT | -0.41283 | -0.64306 | 0.47747 | 1.02223 | 1.38368 | -0.19147 | 0.30932 | -0.06437 | #N/A | -0.19096 | -0.39987 | -0.87191 | -0.84935 | 0.41216 | 0.41569 | 0.37643 | 0.40833 | 7 | 2.93229 | 0.55262 | 0.46739 | 0.492551 | Not Significant | NS | | #N/A |
| 510 | G1SUD0 | Wiskott-Aldrich syndrome protein family member O | WASF1 | RABBIT | -0.26929 | -0.44589 | 0.4516 | 0.84524 | 0.56861 | -2.46563 | -0.3369 | 1.25967 | 1.03371 | -0.34057 | -0.4 | -0.56377 | -0.61165 | 0.58836 | 0.55327 | 0.44644 | 0.46295 | 8 | 2.93229 | 0.55262 | 1.25852 | 1.06918 | Not Significant | NS | | #N/A |
| 1312 | A0A5F9DKI0 | Sm protein F OS=Oryctolagus cuniculus OX=9986 ( | SNRPF | RABBIT | 0.01166 | #N/A | 0.51941 | #N/A | #N/A | -0.13487 | #N/A | #N/A | #N/A | #N/A | -0.4004 | -0.91058 | -0.49016 | 0.52977 | 0.65011 | 0.42625 | 0.49694 | 1 | 2.93229 | 0.55262 | 0.1289 | 0.444865 | Not Significant | NS | | #N/A |
| 1047 | G1TCY8 | Transducin beta like 1 X-linked receptor 1 OS=Oryct | TBL1XR1 | RABBIT | 0.17431 | #N/A | #N/A | #N/A | #N/A | #N/A | #N/A | -0.22702 | #N/A | #N/A | -0.40133 | #N/A | -0.38174 | #N/A | 0.72863 | #N/A | 0.5223 | 0 | 2.93229 | 0.55262 | #N/A | 0.552617 | Not Significant | NS | | #N/A |
| 2880 | G1SYG8 | #N/A | ZNF638 | RABBIT | #N/A | #N/A | #N/A | #N/A | 0.16213 | #N/A | #N/A | -0.23988 | #N/A | #N/A | -0.40201 | #N/A | -0.3824 | #N/A | 0.7282 | #N/A | 0.5223 | 0 | 2.93229 | 0.55262 | #N/A | 0.552617 | Not Significant | NS | | #N/A |
| 238 | G1T519 | Proteasome subunit alpha type OS=Oryctolagus cu | PSMA4 | RABBIT | 0.56503 | 0.35964 | -0.08262 | -0.05764 | 0.7152 | #N/A | 0.788 | -0.18445 | -0.89104 | -0.12455 | -0.40293 | -1.14023 | -1.00287 | 0.2917 | 0.33973 | 0.32069 | 0.3676 | 7 | 2.93229 | 0.55262 | 0.2775 | 0.35872 | Not Significant | NS | | #N/A |
| 583 | A0A5F9C8G2 | 1,4-alpha-glucan branching enzyme OS=Oryctolagu | GBE1 | RABBIT | 0.9071 | #N/A | -0.70552 | 0.43824 | 0.49593 | -0.61878 | -0.50647 | -0.41045 | 0.93798 | 0 | -0.40348 | -0.91085 | -0.8769 | 0.39266 | 0.40125 | 0.36686 | 0.40169 | 7 | 2.93229 | 0.55262 | 0.43605 | 0.470462 | Not Significant | NS | | #N/A |
| 2445 | P36425 | #N/A | SPA17 SP1 | RABBIT | #N/A | #N/A | 0.17065 | #N/A | 0.37107 | #N/A | -0.13489 | #N/A | #N/A | #N/A | -0.40575 | -2.33769 | -0.51292 | 0.25733 | 0.63547 | 0.3027 | 0.49004 | 1 | 2.93229 | 0.55262 | 0.02008 | 0.417191 | Not Significant | NS | | #N/A |
| 3 | A0A5F9CJ90 | Spectrin alpha, non-erythrocytic 1 OS=Oryctolagus | SPTAN1 | RABBIT | #N/A | #N/A | -0.06696 | 0.17997 | 0.75436 | #N/A | 0.96144 | -0.47906 | -0.84052 | #N/A | -0.4085 | -0.67884 | -0.67636 | 0.53449 | 0.52073 | 0.42823 | 0.45229 | 4 | 2.93229 | 0.55262 | 0.54318 | 0.547174 | Not Significant | NS | | #N/A |
| 955 | Q28618 | Y-box-binding protein 1 OS=Oryctolagus cuniculus ( | YBX1 YB1 | RABBIT | -1.13153 | 0.36257 | -0.30045 | 0.36432 | 1.10246 | #N/A | 0.56111 | -0.06437 | -1.07722 | -0.73876 | -0.40928 | -0.76879 | -0.78309 | 0.46716 | 0.45184 | 0.39969 | 0.42172 | 7 | 2.93229 | 0.55262 | 0.62982 | 0.607026 | Not Significant | NS | | #N/A |
| 2300 | G1SFF1 | #N/A | PDE4A | RABBIT | #N/A | #N/A | 0.25803 | #N/A | #N/A | #N/A | #N/A | -0.15174 | #N/A | #N/A | -0.40977 | #N/A | -0.38977 | #N/A | 0.72328 | #N/A | 0.52073 | 0 | 2.93229 | 0.55262 | #N/A | 0.552617 | Not Significant | NS | | #N/A |
| 233 | G1T373 | Ribose-phosphate pyrophosphokinase 1 OS=Oryct | #N/A | RABBIT | 0.13958 | -0.54753 | 0.33522 | -0.5396 | 0.87746 | -1.95192 | 0.02086 | 0.205 | -0.04442 | -0.02086 | -0.4113 | -0.84918 | -0.85583 | 0.42046 | 0.41046 | 0.37927 | 0.40623 | 8 | 2.93229 | 0.55262 | 0.58647 | 0.577392 | Not Significant | NS | | #N/A |
| 1657 | G1TJC3 | #N/A | #N/A | RABBIT | #N/A | 2.07629 | -2.16387 | -0.29316 | #N/A | -1.78124 | -3.40423 | 1.70298 | 0.23552 | 0.54308 | -0.41386 | -0.27446 | -0.32475 | 0.79294 | 0.75285 | 0.51166 | 0.53054 | 6 | 2.93229 | 0.55262 | 4.26349 | 3.045284 | Not Significant | NS | | #N/A |
| 1304 | G1TBL3 | Coiled-coil domain containing 112 OS=Oryctolagus | CCDC112 | RABBIT | 0.01166 | #N/A | #N/A | #N/A | 0.60011 | #N/A | #N/A | #N/A | -0.10827 | #N/A | -0.41415 | -0.81269 | -0.5007 | 0.56555 | 0.6433 | 0.43824 | 0.49399 | 1 | 2.93229 | 0.55262 | 0.17313 | 0.456113 | Not Significant | NS | | #N/A |
| 2778 | A0A5F9CPF7 | #N/A | ACTN1 | RABBIT | #N/A | #N/A | #N/A | #N/A | 0.48864 | #N/A | 0.83009 | -0.06437 | -0.14665 | -0.32393 | -0.41485 | -0.71951 | -0.58116 | 0.52382 | 0.58252 | 0.4245 | 0.47236 | 3 | 2.93229 | 0.55262 | 0.26595 | 0.407649 | Not Significant | NS | | #N/A |
| 2861 | G1SXX2 | #N/A | SRSF6 | RABBIT | #N/A | #N/A | #N/A | #N/A | 0.20166 | #N/A | -0.21504 | #N/A | #N/A | #N/A | -0.41671 | #N/A | -0.39637 | #N/A | 0.71891 | #N/A | 0.51921 | 0 | 2.93229 | 0.55262 | #N/A | 0.552617 | Not Significant | NS | | #N/A |
| 1350 | G1TMG9 | RING-type E3 ubiquitin transferase OS=Oryctolagus | SYVN1 | RABBIT | 0.01166 | 0.16839 | #N/A | 0.40452 | #N/A | #N/A | #N/A | #N/A | 0.45386 | -0.90291 | -0.41938 | -0.79626 | -0.69141 | 0.48407 | 0.51544 | 0.40702 | 0.4502 | 3 | 2.93229 | 0.55262 | 0.33288 | 0.441493 | Not Significant | NS | | #N/A |
| 531 | G1TZQ5 | SEC24 homolog C, COPII coat complex component | #N/A | RABBIT | 0.05011 | 0.43125 | #N/A | -0.05764 | 0.29183 | #N/A | -0.32262 | -0.08318 | -0.31675 | #N/A | -0.41974 | -2.84724 | -1.15153 | 0.03594 | 0.28304 | 0.13507 | 0.33607 | 5 | 2.93229 | 0.55262 | 0.03726 | 0.227767 | Not Significant | NS | | #N/A |
| 1755 | G1SUS7 | #N/A | slc4a10 | RABBIT | #N/A | -0.61707 | 0.12147 | 0.40662 | 0.33708 | #N/A | #N/A | -0.01519 | #N/A | -0.7181 | -0.42867 | -1.03997 | -0.8199 | 0.35708 | 0.43955 | 0.35078 | 0.41667 | 4 | 2.93229 | 0.55262 | 0.22653 | 0.364464 | Not Significant | NS | | #N/A |
| 1705 | G1TD41 | #N/A | HNRNPH1 | RABBIT | #N/A | 0.76201 | 1.38049 | -1.38409 | -0.01338 | -2.7737 | -2.26308 | 2.01846 | -0.7868 | 2.58054 | -0.43117 | -0.32063 | -0.37137 | 0.75785 | 0.71816 | 0.50158 | 0.51888 | 7 | 2.93229 | 0.55262 | 4.01871 | 2.995423 | Not Significant | NS | | #N/A |
| 1358 | A0A5F9CTS6 | Nidogen 1 OS=Oryctolagus cuniculus OX=9986 PE= | #N/A | RABBIT | #N/A | #N/A | #N/A | -0.37588 | 1.23973 | #N/A | 0 | #N/A | #N/A | #N/A | -0.43192 | -0.3087 | -0.40887 | 0.80938 | 0.70392 | 0.51584 | 0.51495 | 1 | 2.93229 | 0.55262 | 1.30509 | 0.743975 | Not Significant | NS | | #N/A |
| 1451 | G1T1E0 | Influenza virus NS1A binding protein OS=Oryctolagu | IVNS1ABP | RABBIT | -0.10773 | #N/A | 0.17065 | #N/A | 1.53754 | -0.25155 | 0.41342 | 0.37924 | -0.14421 | #N/A | -0.43426 | -0.91985 | -0.85222 | 0.39985 | 0.41908 | 0.37074 | 0.40859 | 5 | 2.93229 | 0.55262 | 0.38207 | 0.445117 | Not Significant | NS | | #N/A |
| 1423 | A0A5F9DQ39 | Nipsnap homolog 2 OS=Oryctolagus cuniculus OX= | NIPSNAP2 | RABBIT | 0.01166 | #N/A | #N/A | #N/A | #N/A | #N/A | #N/A | 0.60219 | -1.44855 | #N/A | -0.43484 | -0.24484 | -0.36488 | 0.84713 | 0.73398 | 0.52595 | 0.52403 | 1 | 2.93229 | 0.55262 | 2.10275 | 0.946824 | Not Significant | NS | | #N/A |
| 2211 | G1TN13 | #N/A | ITSN1 | RABBIT | #N/A | #N/A | -0.2635 | 0.62481 | #N/A | #N/A | -0.31463 | 1.26174 | 0.31463 | -2.28307 | -0.43598 | -0.37691 | -0.44793 | 0.72538 | 0.66787 | 0.49085 | 0.50179 | 4 | 2.93229 | 0.55262 | 1.78403 | 1.263157 | Not Significant | NS | | #N/A |
| 353 | T2FI70 | Fatty acid synthase (Fragment) OS=Oryctolagus cui | FASN | RABBIT | 0.36323 | -0.31768 | 0.00255 | 0.11046 | 0.68932 | -0.87334 | 0.25927 | -0.43439 | 0.18868 | -0.4739 | -0.43631 | -1.59345 | -1.29137 | 0.14973 | 0.22321 | 0.23045 | 0.29936 | 8 | 2.93229 | 0.55262 | 0.18744 | 0.285389 | Not Significant | NS | | #N/A |
| 211 | G1SKS0 | Actin-related protein 2/3 complex subunit OS=Oryc | ARPC1A | RABBIT | 0.29877 | 0.02815 | -0.10706 | 0.22205 | 0.19525 | -0.98904 | 0.04962 | 0.11383 | -0.04962 | -0.67145 | -0.43676 | -1.8828 | -1.39045 | 0.09649 | 0.19205 | 0.1866 | 0.27763 | 8 | 2.93229 | 0.55262 | 0.13453 | 0.246671 | Not Significant | NS | | #N/A |
| 2403 | G1SSC1 | #N/A | USP12 | RABBIT | #N/A | #N/A | #N/A | -0.05764 | #N/A | #N/A | #N/A | -1.08969 | #N/A | 0.09901 | -0.4377 | -0.42519 | -0.46458 | 0.74406 | 0.66678 | 0.49742 | 0.5012 | 1 | 2.93229 | 0.55262 | 0.7065 | 0.591749 | Not Significant | NS | | #N/A |
| 1792 | G1SX57 | #N/A | SGTB | RABBIT | #N/A | 1.29055 | #N/A | 1.13876 | -1.25453 | -0.70077 | -0.84934 | 0.36769 | 1.37925 | -0.43206 | -0.43864 | -0.53716 | -0.59435 | 0.61048 | 0.56703 | 0.45404 | 0.46785 | 6 | 2.93229 | 0.55262 | 1.25028 | 1.021255 | Not Significant | NS | | #N/A |
| 2623 | A0A5F9C3D2 | #N/A | TBC1D2B | RABBIT | #N/A | #N/A | #N/A | 0.19144 | #N/A | #N/A | -0.24908 | #N/A | #N/A | #N/A | -0.44051 | #N/A | -0.41902 | #N/A | 0.704 | #N/A | 0.51495 | 0 | 2.93229 | 0.55262 | #N/A | 0.552617 | Not Significant | NS | | #N/A |
| 803 | A0A5F9CZL2 | Prothymosin alpha OS=Oryctolagus cuniculus OX=9 | #N/A | RABBIT | 0.15848 | 2.69035 | -1.21277 | -0.05764 | -1.02747 | #N/A | 0.71084 | -1.33039 | -1.29709 | 0.58855 | -0.44221 | -0.47302 | -0.5326 | 0.65059 | 0.60602 | 0.46908 | 0.47901 | 7 | 2.93229 | 0.55262 | 1.94219 | 1.531947 | Not Significant | NS | | #N/A |
| 1560 | U3KMT3 | Solute carrier family 33 member 1 OS=Oryctolagus | SLC33A1 | RABBIT | 0.19315 | #N/A | #N/A | -0.65536 | 0.992 | #N/A | 0.21371 | -0.24586 | #N/A | -0.76674 | -0.44289 | -0.80008 | -0.7679 | 0.46849 | 0.46789 | 0.40036 | 0.42854 | 4 | 2.93229 | 0.55262 | 0.45965 | 0.498972 | Not Significant | NS | | #N/A |
| 391 | G1SLX2 | guanylate cyclase OS=Oryctolagus cuniculus OX=9 | GUCY1A2 | RABBIT | -0.35232 | 0.0072 | -0.07229 | 1.48125 | 0.62608 | -0.73808 | 0.28665 | 0.02921 | 0 | #N/A | -0.44353 | -1.05922 | -0.99932 | 0.32468 | 0.34137 | 0.33479 | 0.36834 | 7 | 2.93229 | 0.55262 | 0.38965 | 0.437761 | Not Significant | NS | | #N/A |
| 906 | A0A5F9DA47 | Clathrin/coatomer adaptor adaptin-like N-terminal ( | #N/A | RABBIT | 0.64957 | 0.32268 | 0.43876 | -0.77614 | 0.73928 | -0.20936 | -0.03742 | -0.02695 | -0.10547 | -0.46779 | -0.44423 | -1.55977 | -1.28967 | 0.15744 | 0.22378 | 0.23659 | 0.29952 | 8 | 2.93229 | 0.55262 | 0.20278 | 0.296617 | Not Significant | NS | | #N/A |
| 216 | G1SGA5 | Vesicle amine transport 1 like OS=Oryctolagus cuni | VAT1L | RABBIT | 0.30141 | #N/A | #N/A | #N/A | #N/A | #N/A | 0 | -0.18803 | 0.04436 | -0.43702 | -0.44658 | -1.82312 | -0.73241 | 0.1658 | 0.49183 | 0.24513 | 0.43988 | 3 | 2.93229 | 0.55262 | 0.048 | 0.29743 | Not Significant | NS | | #N/A |
| 1048 | A0A5F9DQ50 | Calpain small subunit 1 OS=Oryctolagus cuniculus | CAPNS1 | RABBIT | 1.71252 | #N/A | -0.63034 | #N/A | 0.05623 | -0.06962 | #N/A | #N/A | #N/A | #N/A | -0.44909 | -0.32291 | -0.4062 | 0.7774 | 0.70162 | 0.507 | 0.51474 | 2 | 2.93229 | 0.55262 | 1.45061 | 0.916744 | Not Significant | NS | | #N/A |
| 2099 | A0A375YZN5 | #N/A | #N/A | MT | #N/A | 0.32352 | #N/A | #N/A | #N/A | #N/A | #N/A | #N/A | -0.1257 | #N/A | -0.44921 | #N/A | -0.42729 | #N/A | 0.69859 | #N/A | 0.5134 | 0 | 2.93229 | 0.55262 | #N/A | 0.552617 | Not Significant | NS | | #N/A |
| 1333 | P9WIS7 | Dihydrolipoyllysine-residue acetyltransferase comp | #N/A | MT | 0.96966 | #N/A | 0.47428 | #N/A | -1.00453 | #N/A | -0.30363 | #N/A | #N/A | #N/A | -0.4501 | -0.37951 | -0.44821 | 0.74082 | 0.673 | 0.4961 | 0.50369 | 2 | 2.93229 | 0.55262 | 1.05496 | 0.756311 | Not Significant | NS | | #N/A |
| 979 | U3KNT5 | D-ribitol-5-phosphate cytidylyltransferase OS=Oryc | CRPPA | RABBIT | 0.01166 | #N/A | #N/A | #N/A | 1.9919 | #N/A | -0.35462 | #N/A | 2.40276 | -0.39665 | -0.45129 | -0.32117 | -0.40756 | 0.76916 | 0.6979 | 0.50494 | 0.51333 | 3 | 2.93229 | 0.55262 | 2.36929 | 1.471324 | Not Significant | NS | | #N/A |
| 1421 | A0A5F9D0L3 | Conserved oligomeric Golgi complex subunit 7 OS= | COG7 | RABBIT | 0.23647 | #N/A | #N/A | #N/A | #N/A | #N/A | -0.43116 | #N/A | #N/A | 0 | -0.45205 | -1.21066 | -0.55916 | 0.43952 | 0.60636 | 0.38598 | 0.47901 | 1 | 2.93229 | 0.55262 | 0.09295 | 0.435721 | Not Significant | NS | | #N/A |
| 1555 | A0A5F9CLT4 | Hydroxysteroid 17-beta dehydrogenase 14 OS=Ory | HSD17B14 | RABBIT | 0.29519 | #N/A | 1.20392 | -0.50375 | #N/A | #N/A | 0 | 0.58185 | -0.49119 | -0.5744 | -0.45272 | -0.87182 | -0.84193 | 0.42319 | 0.42448 | 0.37994 | 0.40956 | 5 | 2.93229 | 0.55262 | 0.46226 | 0.495663 | Not Significant | NS | | #N/A |
| 1209 | A0A5F9DBH7 | Galectin 3 binding protein OS=Oryctolagus cuniculu | LGALS3BP | RABBIT | 2.15653 | #N/A | -1.31106 | #N/A | #N/A | #N/A | #N/A | -0.69086 | 0.62649 | #N/A | -0.45492 | -0.24528 | -0.34653 | 0.82911 | 0.74324 | 0.52053 | 0.5281 | 2 | 2.93229 | 0.55262 | 3.4399 | 1.723385 | Not Significant | NS | | #N/A |
| 2302 | G1TD00 | #N/A | C6orf163 | RABBIT | #N/A | #N/A | 0.70689 | -0.05764 | #N/A | -0.13107 | #N/A | #N/A | #N/A | #N/A | -0.4557 | -0.68826 | -0.5335 | 0.61624 | 0.62242 | 0.45508 | 0.48491 | 1 | 2.93229 | 0.55262 | 0.29225 | 0.486404 | Not Significant | NS | | #N/A |
| 2436 | G1TC60 | #N/A | MTM1 | RABBIT | #N/A | #N/A | 0.17065 | #N/A | 0.06573 | #N/A | #N/A | #N/A | -0.33897 | #N/A | -0.45716 | -5.03102 | -0.58049 | 0.12491 | 0.59322 | 0.21204 | 0.47395 | 1 | 2.93229 | 0.55262 | 0.0055 | 0.413483 | Not Significant | NS | | #N/A |
| 1733 | G1SL07 | #N/A | LIN7C | RABBIT | #N/A | 0.19405 | #N/A | 0.72087 | #N/A | #N/A | 0 | #N/A | #N/A | #N/A | -0.45746 | -1.00268 | -0.55844 | 0.49915 | 0.60681 | 0.41338 | 0.47901 | 1 | 2.93229 | 0.55262 | 0.13877 | 0.447373 | Not Significant | NS | | #N/A |
| 1906 | A0A5F9C9L1 | #N/A | HSDL2 | RABBIT | #N/A | #N/A | 0.30657 | #N/A | #N/A | 1.15606 | #N/A | -0.20028 | -1.41179 | #N/A | -0.45858 | -0.30915 | -0.3976 | 0.78644 | 0.70755 | 0.50949 | 0.51642 | 2 | 2.93229 | 0.55262 | 1.65022 | 0.997686 | Not Significant | NS | | #N/A |
| 1841 | G1SQZ5 | #N/A | CEP152 | RABBIT | #N/A | #N/A | -0.44198 | 0.3118 | 1.32959 | #N/A | 0.07696 | -0.14133 | -0.11207 | #N/A | -0.45861 | -0.88563 | -0.82295 | 0.42585 | 0.43793 | 0.38072 | 0.41582 | 4 | 2.93229 | 0.55262 | 0.40223 | 0.465843 | Not Significant | NS | | #N/A |
| 592 | G1SFB8 | Cortactin-binding protein 2 OS=Oryctolagus cunicu | CTTNBP2 | RABBIT | 0.30132 | -4.11264 | 1.74838 | 0.25471 | 1.14325 | -1.24865 | -0.64131 | -0.35402 | -1.12157 | 0.39986 | -0.46014 | -0.42816 | -0.48383 | 0.67983 | 0.63805 | 0.47768 | 0.49167 | 8 | 2.93229 | 0.55262 | 2.88742 | 2.261176 | Not Significant | NS | | #N/A |
| 823 | A0A5F9D3V7 | Sperm flagellar 2 OS=Oryctolagus cuniculus OX=99 | SPEF2 | RABBIT | 3.45228 | #N/A | 0.17065 | #N/A | -1.41462 | #N/A | -0.85715 | #N/A | 1.40688 | #N/A | -0.46124 | -0.22683 | -0.3029 | 0.83514 | 0.7723 | 0.5227 | 0.53484 | 3 | 2.93229 | 0.55262 | 4.96196 | 2.782455 | Not Significant | NS | | #N/A |
| 804 | A0A5F9DNW6 | Crystallin zeta like 1 OS=Oryctolagus cuniculus OX= | CRYZL1 | RABBIT | 0.89382 | 0.19782 | 0.02163 | #N/A | 0.62281 | #N/A | #N/A | #N/A | -0.02809 | #N/A | -0.46211 | -1.04101 | -0.69579 | 0.37439 | 0.51288 | 0.35756 | 0.44921 | 3 | 2.93229 | 0.55262 | 0.15764 | 0.352874 | Not Significant | NS | | #N/A |
| 2758 | G1TKQ8 | #N/A | UBE3A | RABBIT | #N/A | #N/A | #N/A | 0.27572 | #N/A | #N/A | #N/A | -0.29137 | -0.49763 | 0.22701 | -0.46306 | -1.0741 | -0.64625 | 0.39517 | 0.54698 | 0.36874 | 0.46295 | 2 | 2.93229 | 0.55262 | 0.13939 | 0.385059 | Not Significant | NS | | #N/A |
| 965 | G1SFH8 | Phosducin-like protein 3 OS=Oryctolagus cuniculus | PDCL3 | RABBIT | 0.63219 | 0.7042 | -0.56316 | 0.35389 | -0.01338 | #N/A | -0.07204 | -0.41302 | #N/A | #N/A | -0.46527 | -1.16048 | -0.9413 | 0.29824 | 0.37433 | 0.32334 | 0.38756 | 5 | 2.93229 | 0.55262 | 0.22964 | 0.34903 | Not Significant | NS | | #N/A |
| 86 | G1U8W5 | Brevican OS=Oryctolagus cuniculus OX=9986 GN=I | BCAN | RABBIT | -0.10113 | -0.61147 | 0.54738 | 0.45037 | 1.01137 | -1.39711 | 0.86349 | 0.04843 | -0.40833 | -0.13835 | -0.46568 | -1.01068 | -1.00514 | 0.34176 | 0.33656 | 0.34416 | 0.36614 | 8 | 2.93229 | 0.55262 | 0.53076 | 0.53662 | Not Significant | NS | | #N/A |
| 1284 | A0A375YIV4 | Putative 3-oxoacyl-[acyl-carrier protein] reductase F | #N/A | MT | 0.32027 | #N/A | -0.37224 | #N/A | 0.33395 | #N/A | #N/A | -0.37298 | #N/A | #N/A | -0.46697 | -1.00145 | -0.64374 | 0.42209 | 0.54848 | 0.3798 | 0.46295 | 2 | 2.93229 | 0.55262 | 0.16308 | 0.394661 | Not Significant | NS | | #N/A |
| 56 | G1SL02 | AP complex subunit beta OS=Oryctolagus cuniculus | AP2B1 | RABBIT | 0.43159 | 0.1024 | 0.20842 | 0.16181 | 0.55516 | -0.48138 | -0.20122 | 0.07849 | -0.03777 | -0.2379 | -0.46783 | -3.64084 | -1.75116 | 0.00658 | 0.10789 | 0.07586 | 0.20706 | 8 | 2.93229 | 0.55262 | 0.04128 | 0.178431 | Not Significant | NS | | #N/A |
| 2031 | A0A375YBG0 | #N/A | #N/A | MT | #N/A | 0.33325 | #N/A | #N/A | #N/A | #N/A | #N/A | #N/A | -0.13543 | #N/A | -0.46868 | #N/A | -0.44581 | #N/A | 0.68659 | #N/A | 0.50931 | 0 | 2.93229 | 0.55262 | #N/A | 0.552617 | Not Significant | NS | | #N/A |
| 1676 | A0A5F9D7X3 | #N/A | DCTN2 | RABBIT | #N/A | -0.5173 | 0.21158 | -0.09857 | #N/A | -2.45059 | 0.55619 | 0.084 | #N/A | #N/A | -0.4687 | -0.48968 | -0.56653 | 0.65 | 0.5889 | 0.46888 | 0.4724 | 4 | 2.93229 | 0.55262 | 1.37421 | 1.026687 | Not Significant | NS | | #N/A |
| 188 | G1SH47 | Amphiphysin OS=Oryctolagus cuniculus OX=9986 ( | AMPH | RABBIT | 0.79321 | 0.55061 | -0.73907 | 0.27263 | #N/A | #N/A | -0.2339 | 0.16954 | -0.29496 | -0.63905 | -0.46894 | -1.24954 | -1.08934 | 0.258 | 0.30452 | 0.30277 | 0.3501 | 6 | 2.93229 | 0.55262 | 0.28168 | 0.370626 | Not Significant | NS | | #N/A |
| 1645 | U3KN46 | Myb-like, SWIRM and MPN domain-containing prote | MYSM1 | RABBIT | 0.10185 | -0.60564 | 0.63954 | #N/A | 0.39758 | #N/A | -0.52298 | -0.15455 | #N/A | #N/A | -0.4721 | -1.12391 | -0.8968 | 0.32394 | 0.3999 | 0.33479 | 0.40151 | 4 | 2.93229 | 0.55262 | 0.23525 | 0.369495 | Not Significant | NS | | #N/A |
| 621 | A0A5F9D9W6 | Endophilin-B2 OS=Oryctolagus cuniculus OX=9986 | SH3GLB2 | RABBIT | 0.27925 | -0.3379 | 0.64075 | 0.39493 | -0.24369 | -0.3531 | -1.52912 | 0.16595 | 0.58275 | -0.50632 | -0.47463 | -1.17578 | -1.1233 | 0.27349 | 0.28537 | 0.31045 | 0.33729 | 8 | 2.93229 | 0.55262 | 0.40738 | 0.446339 | Not Significant | NS | | #N/A |
| 2548 | G1SQE8 | #N/A | H2BC26 | RABBIT | #N/A | #N/A | #N/A | 0.17655 | #N/A | #N/A | #N/A | -0.29855 | #N/A | #N/A | -0.4751 | #N/A | -0.45191 | #N/A | 0.68266 | #N/A | 0.50801 | 0 | 2.93229 | 0.55262 | #N/A | 0.552617 | Not Significant | NS | | #N/A |
| 1797 | G1SW94 | #N/A | CYTH1 | RABBIT | #N/A | 0.19782 | #N/A | #N/A | #N/A | #N/A | -0.56758 | 0.01214 | #N/A | #N/A | -0.47554 | -0.94719 | -0.57573 | 0.51726 | 0.59614 | 0.42183 | 0.47495 | 1 | 2.93229 | 0.55262 | 0.16804 | 0.454816 | Not Significant | NS | | #N/A |
| 2184 | G1TQK1 | #N/A | #N/A | RABBIT | #N/A | #N/A | 0.25184 | #N/A | #N/A | -0.38305 | #N/A | -0.06437 | #N/A | #N/A | -0.47555 | -1.7231 | -0.5956 | 0.33476 | 0.58402 | 0.33875 | 0.47236 | 1 | 2.93229 | 0.55262 | 0.05078 | 0.424997 | Not Significant | NS | | #N/A |
| 2842 | A0A5F9CZX3 | #N/A | #N/A | RABBIT | #N/A | #N/A | #N/A | #N/A | 0.42305 | #N/A | #N/A | -0.06437 | #N/A | -0.04131 | -0.47589 | -23.8351 | -0.60525 | 0.02669 | 0.5782 | 0.12384 | 0.47095 | 1 | 2.93229 | 0.55262 | 0.00027 | 0.412151 | Not Significant | NS | | #N/A |
| 2232 | A0A5F9CTC4 | #N/A | MAT2A | RABBIT | #N/A | #N/A | 0.32343 | #N/A | #N/A | -0.15278 | #N/A | #N/A | #N/A | #N/A | -0.47621 | #N/A | -0.45297 | #N/A | 0.68198 | #N/A | 0.50772 | 0 | 2.93229 | 0.55262 | #N/A | 0.552617 | Not Significant | NS | | #N/A |
| 568 | G1TDT6 | Isochorismatase domain-containing protein 1 OS=( | ISOC1 | RABBIT | 1.16604 | 1.81372 | -0.12525 | -0.05764 | -5.80305 | #N/A | -0.45514 | -0.02256 | 0.47837 | -4.31435 | -0.47719 | -0.26371 | -0.30924 | 0.7996 | 0.76353 | 0.51361 | 0.53345 | 7 | 2.93229 | 0.55262 | 7.27634 | 5.291311 | Not Significant | NS | | #N/A |
| 94 | G1SWY6 | Early endosome antigen 1 OS=Oryctolagus cunicul | EEA1 | RABBIT | -0.03837 | -1.83479 | 1.03098 | -0.51251 | 1.39585 | -3.22888 | 0.76071 | 0.19014 | -0.12728 | 0.05003 | -0.47929 | -0.52622 | -0.58714 | 0.61301 | 0.56903 | 0.45455 | 0.46793 | 8 | 2.93229 | 0.55262 | 2.07392 | 1.665872 | Not Significant | NS | | #N/A |
| 1131 | A0A5F9DIP4 | Histone deacetylase 6 OS=Oryctolagus cuniculus ( | HDAC6 | RABBIT | #N/A | #N/A | #N/A | -0.05764 | #N/A | 0.03369 | #N/A | -1.31399 | 0.3436 | -1.2206 | -0.48169 | -0.50632 | -0.53883 | 0.6475 | 0.60961 | 0.46795 | 0.47977 | 3 | 2.93229 | 0.55262 | 0.72404 | 0.639309 | Not Significant | NS | | #N/A |

| | | | | | | | | | | | | | | | | | | | | | | | | | | | | | |
|---|---|---|---|---|---|---|---|---|---|---|---|---|---|---|---|---|---|---|---|---|---|---|---|---|---|---|---|---|---|
| 1909 | G1TIJ4 | #N/A | GRIK5 | RABBIT | #N/A | 0.43559 | #N/A | 0.26873 | #N/A | 0 | -0.14187 | 0.05505 | -0.39653 | -0.16554 | -0.48194 | -3.47342 | -1.2236 | 0.01778 | 0.2562 | 0.11065 | 0.32205 | 5 | 2.93229 | 0.55262 | 0.0275 | 0.221619 | Not Significant | NS | #N/A |
| 2786 | G1U3I6 | #N/A | #N/A | RABBIT | #N/A | #N/A | #N/A | #N/A | 0.33666 | -0.61483 | -0.01583 | 0.02466 | -0.1525 | 0.01583 | -0.4852 | -1.63913 | -0.84327 | 0.17653 | 0.42721 | 0.25448 | 0.41011 | 4 | 2.93229 | 0.55262 | 0.07302 | 0.275883 | Not Significant | NS | #N/A |
| 40 | G1SY02 | Tubulin beta chain OS=Oryctolagus cuniculus OX=9 | #N/A | RABBIT | -1.90464 | 4.61125 | -0.2857 | 0.05821 | -0.8945 | 0.30296 | -1.51483 | -0.18021 | 0.13086 | 0.41284 | -0.4866 | -0.4132 | -0.4695 | 0.69032 | 0.64794 | 0.48157 | 0.49594 | 8 | 2.93229 | 0.55262 | 3.46711 | 2.685375 | Not Significant | NS | #N/A |
| 610 | Q6X276 | Huntingtin interacting protein 1-related OS=Oryctol | #N/A | RABBIT | 0.56319 | -1.05792 | 0.96199 | 0.23203 | -0.31517 | -2.89472 | -0.33994 | -0.35404 | 1.05223 | 0.46738 | -0.49064 | -0.64494 | -0.70612 | 0.53702 | 0.49489 | 0.42838 | 0.44101 | 8 | 2.93229 | 0.55262 | 1.4469 | 1.207033 | Not Significant | NS | #N/A |
| 1837 | A0A5F9DUH7 | #N/A | TBL1XR1 | RABBIT | #N/A | 0.08366 | -0.63927 | 0.9242 | 1.59653 | #N/A | 0.03804 | #N/A | #N/A | -0.03804 | -0.49128 | -0.67133 | -0.70593 | 0.53879 | 0.50326 | 0.42845 | 0.445 | 4 | 2.93229 | 0.55262 | 0.71404 | 0.645762 | Not Significant | NS | #N/A |
| 2294 | G1TX70 | #N/A | #N/A | RABBIT | #N/A | #N/A | #N/A | #N/A | 0.2391 | -0.25248 | #N/A | #N/A | #N/A | #N/A | -0.49158 | #N/A | -0.4676 | #N/A | 0.67263 | #N/A | 0.50369 | 0 | 2.93229 | 0.55262 | #N/A | 0.552617 | Not Significant | NS | #N/A |
| 808 | G1TUP2 | IRG-type G domain-containing protein OS=Oryctola | #N/A | RABBIT | 0.76701 | 0.91325 | -0.90054 | 0.4832 | -1.01708 | -1.83471 | -0.66307 | -0.25576 | 0.1914 | 0.3488 | -0.49184 | -0.86017 | -0.90016 | 0.41473 | 0.38744 | 0.37703 | 0.39423 | 8 | 2.93229 | 0.55262 | 0.81736 | 0.746351 | Not Significant | NS | #N/A |
| 303 | G1SG83 | Adenylyl cyclase-associated protein OS=Oryctolagu | CAP2 | RABBIT | 0.41349 | 0.28755 | -0.32452 | 0.84365 | -0.36171 | -1.26466 | 0.15059 | -0.05664 | -0.44694 | -0.00773 | -0.49677 | -1.44895 | -1.3032 | 0.18539 | 0.21929 | 0.25965 | 0.29674 | 8 | 2.93229 | 0.55262 | 0.29386 | 0.363263 | Not Significant | NS | #N/A |
| 1388 | A0A5F9C2Z6 | Diacylglycerol kinase OS=Oryctolagus cuniculus O) | DGKI | RABBIT | 0.01166 | #N/A | -0.25536 | 0.50369 | #N/A | #N/A | #N/A | -0.90625 | #N/A | 0.086 | -0.49679 | -1.0613 | -0.85395 | 0.36642 | 0.42627 | 0.35413 | 0.41011 | 3 | 2.93229 | 0.55262 | 0.26293 | 0.406122 | Not Significant | NS | #N/A |
| 2391 | G1U1K4 | #N/A | #N/A | RABBIT | #N/A | #N/A | 0.12764 | #N/A | 0.02963 | #N/A | #N/A | -0.01688 | -0.82449 | #N/A | -0.49932 | -1.22754 | -0.79385 | 0.34449 | 0.46375 | 0.34542 | 0.42633 | 2 | 2.93229 | 0.55262 | 0.16546 | 0.395628 | Not Significant | NS | #N/A |
| 1027 | G1TBZ1 | GABA type A receptor associated protein like 2 OS= | GABARAPL | RABBIT | 0.1412 | 1.36607 | -0.15733 | 0.61238 | -0.44051 | -1.07758 | #N/A | 0.46737 | 0 | -0.17099 | -0.49966 | -1.0893 | -1.06118 | 0.31209 | 0.31373 | 0.32949 | 0.35365 | 7 | 2.93229 | 0.55262 | 0.46757 | 0.492677 | Not Significant | NS | #N/A |
| 2790 | G1T7V6 | #N/A | FRMD6 | RABBIT | #N/A | #N/A | #N/A | #N/A | 0.33015 | -0.38606 | #N/A | 0.57178 | -0.87616 | 0 | -0.50276 | -0.73451 | -0.66108 | 0.51584 | 0.53338 | 0.42132 | 0.45675 | 3 | 2.93229 | 0.55262 | 0.37482 | 0.462704 | Not Significant | NS | #N/A |
| 2441 | G1SI58 | #N/A | EPN3 | RABBIT | #N/A | #N/A | 0.4547 | -0.52071 | #N/A | -1.94313 | -1.54561 | -0.06437 | 0.45196 | 0.41779 | -0.50366 | -0.56968 | -0.6317 | 0.59353 | 0.54535 | 0.44827 | 0.46288 | 5 | 2.93229 | 0.55262 | 1.11665 | 0.908148 | Not Significant | NS | #N/A |
| 2216 | G1SMV4 | #N/A | ATP2B2 | RABBIT | #N/A | #N/A | 0.17065 | #N/A | #N/A | -0.72131 | #N/A | 0.05087 | #N/A | #N/A | -0.50587 | -0.75646 | -0.59133 | 0.58771 | 0.58662 | 0.44618 | 0.47237 | 1 | 2.93229 | 0.55262 | 0.29814 | 0.487901 | Not Significant | NS | #N/A |
| 963 | Q9TRH8 | Glutathione S-transferase class MU PI7.8 isozyme ( | #N/A | RABBIT | 0.84958 | 2.16673 | -0.7632 | -0.69732 | #N/A | #N/A | 0.25701 | -0.60782 | #N/A | 0 | -0.50589 | -0.59127 | -0.66392 | 0.58005 | 0.52556 | 0.44328 | 0.45323 | 5 | 2.93229 | 0.55262 | 1.25491 | 0.995299 | Not Significant | NS | #N/A |
| 1468 | G1SQ76 | Ubiquitin carboxyl-terminal hydrolase OS=Oryctola | USP13 | RABBIT | #N/A | #N/A | #N/A | 0.06661 | -0.01338 | #N/A | -1.9251 | #N/A | 0.68045 | -0.19396 | -0.50615 | -0.51187 | -0.59564 | 0.64404 | 0.57342 | 0.46712 | 0.46967 | 3 | 2.93229 | 0.55262 | 1.17333 | 0.866515 | Not Significant | NS | #N/A |
| 378 | G1SWI7 | Proteasome 20S subunit alpha 8 OS=Oryctolagus c | PSMA8 | RABBIT | 0.4955 | 0.19782 | 0.06249 | -0.06162 | 0.74098 | #N/A | 0.49552 | -0.39439 | -0.98433 | 0.00244 | -0.50722 | -1.57883 | -1.32675 | 0.15838 | 0.21428 | 0.23777 | 0.29205 | 7 | 2.93229 | 0.55262 | 0.22936 | 0.324794 | Not Significant | NS | #N/A |
| 128 | G1SFE4 | phosphoinositide 5-phosphatase OS=Oryctolagus c | SYNJ1 | RABBIT | 0.92592 | 0.53635 | -0.28344 | 0.28837 | 0.0731 | -0.23228 | -0.91537 | -0.15085 | 0.76779 | -0.47227 | -0.50866 | -1.4791 | -1.33197 | 0.17738 | 0.20997 | 0.25448 | 0.28986 | 8 | 2.93229 | 0.55262 | 0.29566 | 0.364583 | Not Significant | NS | #N/A |
| 1256 | G1SUH9 | Cadherin 10 OS=Oryctolagus cuniculus OX=9986 G | CDH10 | RABBIT | 0.86127 | #N/A | #N/A | -0.45039 | 0.62072 | #N/A | -0.17884 | 0.11447 | -0.43067 | #N/A | -0.50888 | -1.17569 | -0.99053 | 0.30492 | 0.35522 | 0.32493 | 0.37688 | 4 | 2.93229 | 0.55262 | 0.28102 | 0.395902 | Not Significant | NS | #N/A |
| 1972 | A0A5F9DGG0 | #N/A | #N/A | RABBIT | #N/A | #N/A | #N/A | -0.05764 | #N/A | -3.25338 | #N/A | -0.14291 | 0.94533 | 0.18348 | -0.50923 | -0.24645 | -0.32203 | 0.82124 | 0.75848 | 0.51781 | 0.53209 | 3 | 2.93229 | 0.55262 | 3.41563 | 2.000461 | Not Significant | NS | #N/A |
| 1993 | A0A5F9DTP3 | #N/A | ZNF711 | RABBIT | #N/A | -0.09687 | #N/A | 1.11542 | #N/A | #N/A | 0 | #N/A | #N/A | #N/A | -0.50927 | -0.48509 | -0.53729 | 0.71247 | 0.62002 | 0.48864 | 0.48431 | 1 | 2.93229 | 0.55262 | 0.73481 | 0.598951 | Not Significant | NS | #N/A |
| 144 | G1SJN4 | COP9 signalosome complex subunit 4 OS=Oryctola | COPS4 | RABBIT | 0.71605 | 0.1644 | 0.08733 | 0.18963 | 0.51902 | -2.79569 | 0.03342 | -0.19945 | -0.37534 | 2.46025 | -0.51065 | -0.6063 | -0.67145 | 0.56112 | 0.51587 | 0.43688 | 0.45035 | 8 | 2.93229 | 0.55262 | 1.77342 | 1.445971 | Not Significant | NS | #N/A |
| 440 | A0A5F9CDH9 | CAP-Gly domain containing linker protein 2 OS=Ory | CLIP2 | RABBIT | 0.78336 | -0.79824 | -0.37704 | 0.65157 | 0.58904 | -1.86249 | -0.45956 | -0.20227 | 0.68169 | 0.13791 | -0.51068 | -0.96134 | -0.99055 | 0.36453 | 0.34332 | 0.35393 | 0.36928 | 8 | 2.93229 | 0.55262 | 0.70549 | 0.664487 | Not Significant | NS | #N/A |
| 1097 | A0A5F9DLJ4 | Myocyte enhancer factor 2D OS=Oryctolagus cunic | MEF2D | RABBIT | -0.19901 | #N/A | 0.76961 | 0.14965 | 0.39274 | -0.2218 | -0.77547 | 0.06853 | -0.23424 | 0 | -0.51084 | -2.0798 | -1.5003 | 0.07611 | 0.16464 | 0.16938 | 0.25992 | 7 | 2.93229 | 0.55262 | 0.13407 | 0.257634 | Not Significant | NS | #N/A |
| 1174 | G1SWG9 | protein-serine/threonine phosphatase OS=Oryctola | #N/A | RABBIT | 0.86256 | 0.53046 | -0.62155 | 0.15838 | -0.2294 | #N/A | -0.37361 | #N/A | #N/A | #N/A | -0.5137 | -0.79513 | -0.71146 | 0.47105 | 0.50003 | 0.40162 | 0.44329 | 4 | 2.93229 | 0.55262 | 0.34783 | 0.434451 | Not Significant | NS | #N/A |
| 919 | A0A5F9D4N4 | Alpha-synuclein OS=Oryctolagus cuniculus OX=99 | SNCA | RABBIT | -0.32464 | 1.68991 | #N/A | 0.14791 | -0.11884 | #N/A | -0.29932 | 0.04109 | -0.73348 | 0.33109 | -0.51374 | -1.00417 | -0.99512 | 0.35406 | 0.34587 | 0.34909 | 0.37092 | 6 | 2.93229 | 0.55262 | 0.52349 | 0.533051 | Not Significant | NS | #N/A |
| 147 | G1SPM5 | Alpha-centractin OS=Oryctolagus cuniculus OX=99 | ACTR1A | RABBIT | 0.92157 | -0.03635 | -0.00428 | 0.06285 | 0.67848 | -0.6664 | 0.21465 | -0.02933 | -0.43326 | -0.03503 | -0.51433 | -2.02653 | -1.57658 | 0.07727 | 0.14337 | 0.17093 | 0.24263 | 8 | 2.93229 | 0.55262 | 0.16103 | 0.266065 | Not Significant | NS | #N/A |
| 2593 | U3KPG4 | #N/A | #N/A | RABBIT | #N/A | #N/A | #N/A | 0.25857 | #N/A | -0.90816 | 0.05794 | 0.14143 | -0.05794 | -0.514 | -0.51472 | -1.05889 | -0.79723 | 0.34936 | 0.45175 | 0.34656 | 0.42172 | 4 | 2.93229 | 0.55262 | 0.1969 | 0.347367 | Not Significant | NS | #N/A |
| 176 | G1SDA8 | Proteasome subunit alpha type OS=Oryctolagus cu | PSMA1 | RABBIT | 0.59063 | 0.33137 | 0.17065 | -0.40258 | 0.89998 | #N/A | 0.77276 | -0.36394 | -1.06331 | -0.13511 | -0.51541 | -1.24248 | -1.16809 | 0.25407 | 0.27004 | 0.30105 | 0.32977 | 7 | 2.93229 | 0.55262 | 0.3824 | 0.432651 | Not Significant | NS | #N/A |
| 2453 | U3KP25 | #N/A | TMEM35B | RABBIT | #N/A | #N/A | 0.68671 | -0.31712 | 0.57636 | -0.06437 | #N/A | 0 | #N/A | -0.53703 | -0.51578 | -1.43233 | -1.07395 | 0.22532 | 0.3188 | 0.28607 | 0.35646 | 4 | 2.93229 | 0.55262 | 0.19451 | 0.345985 | Not Significant | NS | #N/A |
| 1219 | G1STP9 | Thy-1 membrane glycoprotein OS=Oryctolagus cun | THY1 | RABBIT | -1.22272 | 0.46314 | 0.81305 | -0.09437 | 1.13205 | 0.03674 | -0.31311 | 0.24566 | -0.42817 | -1.03448 | -0.5169 | -1.10328 | -1.10224 | 0.30198 | 0.29403 | 0.32467 | 0.34341 | 8 | 2.93229 | 0.55262 | 0.54876 | 0.549797 | Not Significant | NS | #N/A |
| 622 | A0A5F9CLC6 | non-specific serine/threonine protein kinase OS=Or | SRPK2 | RABBIT | -0.90893 | -1.03428 | 0.50353 | 0.3494 | 1.21766 | -1.95173 | -0.79597 | 0.28236 | -0.00424 | 0.00424 | -0.51854 | -0.8729 | -0.92022 | 0.40816 | 0.37732 | 0.3744 | 0.38895 | 8 | 2.93229 | 0.55262 | 0.88224 | 0.79383 | Not Significant | NS | #N/A |
| 2464 | G1SU11 | #N/A | CETN2 | RABBIT | #N/A | #N/A | 1.12488 | -1.03452 | #N/A | -1.49074 | 0.11176 | -1.33836 | 0.34723 | 0 | -0.5192 | -0.59966 | -0.66178 | 0.57487 | 0.52687 | 0.44118 | 0.45323 | 5 | 2.93229 | 0.55262 | 1.07092 | 0.879322 | Not Significant | NS | #N/A |
| 2342 | U3KM12 | #N/A | GTF3C4 | RABBIT | #N/A | #N/A | 0.20149 | #N/A | -0.01338 | 1.25076 | #N/A | -1.34311 | #N/A | -1.18793 | -0.52082 | -0.47898 | -0.57322 | 0.6647 | 0.58755 | 0.47235 | 0.47237 | 3 | 2.93229 | 0.55262 | 1.41875 | 0.990628 | Not Significant | NS | #N/A |
| 2249 | G1TPL7 | #N/A | DPP3 | RABBIT | #N/A | #N/A | #N/A | -0.00964 | -0.48236 | -3.2488 | 0.27672 | -1.23039 | 0 | 0.36627 | -0.52124 | -0.45322 | -0.52741 | 0.66938 | 0.61234 | 0.47323 | 0.48071 | 5 | 2.93229 | 0.55262 | 1.88954 | 1.395328 | Not Significant | NS | #N/A |
| 1728 | A0A5F9DIG0 | #N/A | GRIPAP1 | RABBIT | #N/A | -0.23342 | 0.01244 | #N/A | 1.21435 | #N/A | 0.42113 | #N/A | -1.1508 | 0.15821 | -0.52161 | -0.78962 | -0.81699 | 0.47392 | 0.4411 | 0.40265 | 0.41692 | 4 | 2.93229 | 0.55262 | 0.65455 | 0.611434 | Not Significant | NS | #N/A |
| 1961 | G1U4K1 | #N/A | STAT5A | RABBIT | #N/A | 0.36061 | #N/A | #N/A | #N/A | -0.16279 | #N/A | #N/A | #N/A | #N/A | -0.52341 | #N/A | -0.49786 | #N/A | 0.65352 | #N/A | 0.49788 | 0 | 2.93229 | 0.55262 | #N/A | 0.552617 | Not Significant | NS | #N/A |
| 491 | A0A5F9DVA7 | Cytosolic purine 5'-nucleotidase OS=Oryctolagus c | #N/A | RABBIT | -0.06794 | 1.69921 | -0.14615 | 0.26971 | -0.01338 | #N/A | 0.19187 | 0.79396 | -0.69578 | -0.9951 | -0.52455 | -0.98663 | -1.00462 | 0.35669 | 0.33893 | 0.35078 | 0.36732 | 7 | 2.93229 | 0.55262 | 0.62813 | 0.605839 | Not Significant | NS | #N/A |
| 850 | B7NZB4 | Cadherin-2 (Fragment) OS=Oryctolagus cuniculus ( | CDH2 | RABBIT | 1.18334 | 0.59757 | -0.15494 | -0.05764 | 0.25101 | #N/A | 1.15951 | -0.53727 | -0.45956 | -0.80853 | -0.52533 | -1.09513 | -1.08232 | 0.3097 | 0.30469 | 0.32767 | 0.3501 | 7 | 2.93229 | 0.55262 | 0.51135 | 0.523534 | Not Significant | NS | #N/A |
| 1363 | G1T0W8 | Fibrinogen beta chain OS=Oryctolagus cuniculus O | FGB | RABBIT | #N/A | -2.71477 | #N/A | 0.40092 | 1.05973 | -1.49775 | 2.01901 | 0.35877 | -5.18391 | -0.42313 | -0.52736 | -0.29016 | -0.34656 | 0.78146 | 0.73695 | 0.50784 | 0.52494 | 6 | 2.93229 | 0.55262 | 6.19348 | 4.341702 | Not Significant | NS | #N/A |
| 2664 | A0A5F9C0N8 | #N/A | GNE | RABBIT | #N/A | #N/A | #N/A | 0.25377 | #N/A | 0.05215 | -0.72319 | -0.05269 | -0.63311 | -0.01168 | -0.52748 | -1.29282 | -0.85959 | 0.26569 | 0.41875 | 0.30603 | 0.40859 | 4 | 2.93229 | 0.55262 | 0.13872 | 0.313797 | Not Significant | NS | #N/A |
| 1568 | A0A5F9DC97 | Large ribosomal subunit protein uL30-like ferredoxi | #N/A | RABBIT | 0.30083 | #N/A | 0.16708 | 0.42388 | 0.47409 | #N/A | -0.10146 | #N/A | -0.45803 | 0 | -0.52796 | -3.72507 | -1.45413 | 0.01364 | 0.18431 | 0.09752 | 0.27407 | 5 | 2.93229 | 0.55262 | 0.03444 | 0.22599 | Not Significant | NS | #N/A |
| 1866 | G1THF6 | #N/A | LRRC49 | RABBIT | #N/A | 0.19782 | #N/A | #N/A | #N/A | #N/A | #N/A | #N/A | -1.08588 | 0.41897 | -0.53127 | -0.40766 | -0.51846 | 0.75357 | 0.63194 | 0.50058 | 0.4889 | 1 | 2.93229 | 0.55262 | 1.13228 | 0.700028 | Not Significant | NS | #N/A |
| 143 | Q09YN4 | F-actin-capping protein subunit alpha-2 OS=Orycto | CAPZA2 | RABBIT | 0.03231 | 0.22662 | -0.31938 | 0.12363 | 0.02506 | -1.81723 | -0.02065 | -0.21446 | 0.10306 | -0.63084 | -0.53367 | -1.48165 | -1.35892 | 0.17671 | 0.20155 | 0.25448 | 0.28418 | 8 | 2.93229 | 0.55262 | 0.32434 | 0.385566 | Not Significant | NS | #N/A |
| 1526 | A0A5F9C4F1 | ATP-dependent RNA helicase DHX30 OS=Oryctolag | DHX30 | RABBIT | 0.11588 | 0.96387 | -1.97136 | 0.33571 | 1.08585 | -0.64543 | #N/A | -0.16858 | #N/A | -0.47244 | -0.5348 | -0.72152 | -0.7836 | 0.49773 | 0.45355 | 0.41323 | 0.42263 | 6 | 2.93229 | 0.55262 | 1.03015 | 0.873385 | Not Significant | NS | #N/A |
| 2738 | G1T4J6 | #N/A | L3MBTL4 | RABBIT | #N/A | #N/A | #N/A | 0.48145 | #N/A | #N/A | #N/A | #N/A | -0.10698 | 0 | -0.53494 | -5.77395 | -0.67921 | 0.10917 | 0.53488 | 0.19697 | 0.45675 | 1 | 2.93229 | 0.55262 | 0.00572 | 0.413539 | Not Significant | NS | #N/A |
| 1533 | A0A5F9DTM7 | NADH:ubiquinone oxidoreductase subunit A12 OS= | NDUFA12 | RABBIT | 6.85764 | #N/A | -2.5557 | -2.68245 | 6.28368 | 4.0946 | 6.26529 | -0.88502 | -2.27161 | 0 | -0.53514 | -0.18085 | -0.21415 | 0.86161 | 0.83476 | 0.53001 | 0.54964 | 7 | 2.93229 | 0.55262 | 19.4585 | 13.87699 | Not Significant | NS | #N/A |
| 1295 | G1SET2 | RNA binding motif protein 45 OS=Oryctolagus cunic | RBM45 | RABBIT | -0.18043 | 0.31997 | 0.41396 | 0.263 | 0.0034 | #N/A | -0.97026 | -0.08115 | -0.06629 | #N/A | -0.53655 | -2.0416 | -1.41813 | 0.08725 | 0.19008 | 0.17659 | 0.27623 | 6 | 2.93229 | 0.55262 | 0.1295 | 0.268401 | Not Significant | NS | #N/A |
| 1710 | G1T7W2 | #N/A | #N/A | RABBIT | #N/A | 0.28383 | 0.12589 | 0.86302 | -0.01338 | -0.55427 | -0.46576 | #N/A | #N/A | 0.35428 | -0.53676 | -1.61499 | -1.23533 | 0.16723 | 0.25204 | 0.24611 | 0.3189 | 5 | 2.93229 | 0.55262 | 0.18936 | 0.323646 | Not Significant | NS | #N/A |
| 1635 | G1TXX1 | Gem nuclear organelle associated protein 4 OS=Or | GEMIN4 | RABBIT | 0.44518 | #N/A | 0.17065 | #N/A | #N/A | #N/A | #N/A | #N/A | #N/A | -0.22967 | -0.53759 | -2.26113 | -0.67596 | 0.26509 | 0.53673 | 0.30603 | 0.45761 | 1 | 2.93229 | 0.55262 | 0.03768 | 0.421667 | Not Significant | NS | #N/A |
| 1339 | G1T2U1 | Amidophosphoribosyltransferase OS=Oryctolagus ( | PPAT | RABBIT | #N/A | -0.72355 | -0.08935 | 0.17986 | 1.04435 | -1.92575 | -0.55161 | -0.06437 | 0.03868 | 0.32471 | -0.53849 | -0.9707 | -0.99943 | 0.36403 | 0.34131 | 0.35391 | 0.36834 | 7 | 2.93229 | 0.55262 | 0.68386 | 0.645116 | Not Significant | NS | #N/A |
| 947 | A0A5F9D592 | Interacts with SUPT6H, CTD assembly factor 1 OS=( | IWS1 | RABBIT | 1.08292 | -0.66231 | -0.67669 | #N/A | 0.84964 | #N/A | 0.9103 | 0.59477 | -2.40758 | -0.65913 | -0.5388 | -0.60535 | -0.6827 | 0.56712 | 0.51211 | 0.43867 | 0.44921 | 6 | 2.93229 | 0.55262 | 1.58446 | 1.245724 | Not Significant | NS | #N/A |
| 27 | G1TEG5 | Tenascin R OS=Oryctolagus cuniculus OX=9986 GN | TNR | RABBIT | 0.02071 | -0.78931 | 0.74587 | 0.04372 | 1.1087 | -1.82999 | 0.47568 | -0.07342 | -0.03987 | -0.09828 | -0.53911 | -1.05154 | -1.07471 | 0.32373 | 0.30565 | 0.33479 | 0.35054 | 8 | 2.93229 | 0.55262 | 0.65713 | 0.629096 | Not Significant | NS | #N/A |
| 2371 | A0A5F9D7E9 | #N/A | ADAMTSL1 | RABBIT | #N/A | #N/A | #N/A | 0.00603 | #N/A | #N/A | #N/A | -1.06731 | #N/A | 0 | -0.53968 | -0.58387 | -0.59046 | 0.66356 | 0.58714 | 0.47235 | 0.47237 | 1 | 2.93229 | 0.55262 | 0.56958 | 0.55693 | Not Significant | NS | #N/A |
| 2235 | G1U484 | #N/A | LLGL1 | RABBIT | #N/A | #N/A | #N/A | -0.05764 | #N/A | 0.51948 | -1.64645 | #N/A | -1.48778 | 0.22457 | -0.53991 | -0.42816 | -0.50443 | 0.69742 | 0.63214 | 0.4833 | 0.4889 | 3 | 2.93229 | 0.55262 | 1.27211 | 0.916469 | Not Significant | NS | #N/A |
| 1931 | A0A5F9D5F4 | #N/A | LIMCH1 | RABBIT | #N/A | #N/A | #N/A | 0.24115 | #N/A | -0.29878 | #N/A | #N/A | #N/A | #N/A | -0.53993 | #N/A | -0.51358 | #N/A | 0.64373 | #N/A | 0.49405 | 0 | 2.93229 | 0.55262 | #N/A | 0.552617 | Not Significant | NS | #N/A |
| 2826 | G1T4K4 | #N/A | IFT122 | RABBIT | #N/A | #N/A | #N/A | #N/A | 0.2314 | #N/A | #N/A | -0.30915 | #N/A | #N/A | -0.54055 | #N/A | -0.51417 | #N/A | 0.64337 | #N/A | 0.49399 | 0 | 2.93229 | 0.55262 | #N/A | 0.552617 | Not Significant | NS | #N/A |
| 817 | A0A5F9DNV6 | Coiled-coil domain containing 88A OS=Oryctolagus | CCDC88A | RABBIT | -0.92655 | #N/A | 0.97956 | -0.99546 | 2.40922 | #N/A | -2.63325 | 1.13196 | 1.6127 | -0.80891 | -0.54107 | -0.42527 | -0.49851 | 0.68547 | 0.63017 | 0.48039 | 0.4889 | 6 | 2.93229 | 0.55262 | 3.23739 | 2.356033 | Not Significant | NS | #N/A |
| 509 | G1SF44 | Heat shock factor-binding protein 1 OS=Oryctolagu | HSBP1 | RABBIT | -1.75568 | 1.31751 | -1.66819 | -0.08286 | 0.26684 | -4.97223 | -0.02076 | 0.09746 | 0.24684 | 0.02076 | -0.54111 | -0.46187 | -0.52466 | 0.65647 | 0.61029 | 0.4707 | 0.47994 | 8 | 2.93229 | 0.55262 | 3.43137 | 2.65922 | Not Significant | NS | #N/A |
| 2713 | A0A5F9CSY4 | #N/A | MTMR1 | RABBIT | #N/A | #N/A | #N/A | 0.22989 | #N/A | 0 | #N/A | #N/A | -0.62468 | #N/A | -0.54223 | -1.00229 | -0.65156 | 0.49927 | 0.5508 | 0.41338 | 0.46295 | 1 | 2.93229 | 0.55262 | 0.19511 | 0.461701 | Not Significant | NS | #N/A |
| 2647 | A0A5F9DUF0 | #N/A | FASTK | RABBIT | #N/A | #N/A | #N/A | -0.15594 | 1.17764 | #N/A | #N/A | -0.05367 | #N/A | -0.01069 | -0.54303 | -0.81397 | -0.76114 | 0.50116 | 0.48136 | 0.41436 | 0.43475 | 2 | 2.93229 | 0.55262 | 0.44507 | 0.509008 | Not Significant | NS | #N/A |
| 1113 | A0A5F9CT01 | Staufen double-stranded RNA binding protein 1 OS= | STAU1 | RABBIT | 0.16957 | -0.01616 | 0.17065 | 1.12647 | 0.18588 | -0.1504 | #N/A | -0.28261 | #N/A | #N/A | -0.54379 | -1.59064 | -1.16824 | 0.17256 | 0.27662 | 0.2511 | 0.33229 | 5 | 2.93229 | 0.55262 | 0.16696 | 0.309524 | Not Significant | NS | #N/A |
| 2399 | G1TZN7 | #N/A | COX5A | RABBIT | #N/A | #N/A | 0.35885 | 0.20341 | #N/A | -0.12376 | #N/A | 0.05939 | -0.33448 | -0.65375 | -0.54428 | -2.32134 | -1.19619 | 0.08102 | 0.27093 | 0.1731 | 0.33012 | 4 | 2.93229 | 0.55262 | 0.0733 | 0.276046 | Not Significant | NS | #N/A |
| 864 | G1SE51 | Sperm associated antigen 9 OS=Oryctolagus cunicu | SPAG9 | RABBIT | 0.18377 | 1.45875 | -1.95094 | 0.95044 | 0.77474 | -1.39838 | -0.17211 | -0.301 | -0.93021 | 1.49701 | -0.54429 | -0.70528 | -0.77352 | 0.50065 | 0.45561 | 0.41429 | 0.42329 | 8 | 2.93229 | 0.55262 | 1.48894 | 1.2378 | Not Significant | NS | #N/A |
| 1492 | A0A5F9C857 | Myelin regulatory factor like OS=Oryctolagus cunicu | MYRFL | RABBIT | 0.06374 | #N/A | 0.11857 | #N/A | 0.56283 | #N/A | -0.2976 | #N/A | #N/A | #N/A | -0.54598 | -1.72757 | -0.78925 | 0.22621 | 0.46619 | 0.28687 | 0.4279 | 2 | 2.93229 | 0.55262 | 0.07491 | 0.358911 | Not Significant | NS | #N/A |
| 2241 | A0A5F9C7M1 | #N/A | ZFC3H1 | RABBIT | #N/A | #N/A | 0.43415 | #N/A | #N/A | -0.22502 | #N/A | #N/A | #N/A | 0 | -0.54667 | -2.80519 | -0.68995 | 0.218 | 0.52878 | 0.28083 | 0.45399 | 1 | 2.93229 | 0.55262 | 0.02532 | 0.418522 | Not Significant | NS | #N/A |
| 1540 | G1T2P9 | rRNA methyltransferase 1, mitochondrial OS=Oryct | MRM1 | RABBIT | 0.01166 | #N/A | #N/A | #N/A | 0.02214 | #N/A | #N/A | #N/A | #N/A | -0.53041 | -0.54731 | -60.2984 | -0.69613 | 0.01056 | 0.5253 | 0.09648 | 0.45323 | 1 | 2.93229 | 0.55262 | 5.5E-05 | 0.412098 | Not Significant | NS | #N/A |
| 1653 | Q9N120 | Low density lipoprotein-related protein 1 (Fragment | LRP1 | RABBIT | #N/A | #N/A | -0.09228 | 3.8639 | -0.70282 | #N/A | 1.01523 | -0.06437 | #N/A | #N/A | -0.5475 | -0.2895 | -0.38367 | 0.79105 | 0.7146 | 0.5109 | 0.51773 | 3 | 2.93229 | 0.55262 | 4.29193 | 2.443613 | Not Significant | NS | #N/A |
| 560 | P08628 | Thioredoxin OS=Oryctolagus cuniculus OX=9986 G | TXN | RABBIT | 0.02373 | 0.41696 | -0.0283 | 0.15003 | 0.329 | -1.40708 | -0.01639 | -0.07644 | 0.40223 | -0.75017 | -0.54785 | -1.66143 | -1.47021 | 0.1352 | 0.16969 | 0.2182 | 0.26263 | 8 | 2.93229 | 0.55262 | 0.27183 | 0.347147 | Not Significant | NS | #N/A |
| 2725 | A0A5F9DDP9 | #N/A | SFR1 | RABBIT | #N/A | #N/A | #N/A | 0.21328 | #N/A | #N/A | #N/A | -0.33529 | #N/A | #N/A | -0.54857 | #N/A | -0.5218 | #N/A | 0.63865 | #N/A | 0.49191 | 0 | 2.93229 | 0.55262 | #N/A | 0.552617 | Not Significant | NS | #N/A |
| 1359 | A0A5F9D3T8 | C-type lectin domain containing 16A OS=Oryctolag | CLEC16A | RABBIT | 0.51234 | 0.21429 | 0.03905 | -0.0741 | 0.88166 | #N/A | 0.25361 | -0.44407 | #N/A | -0.51153 | -0.54865 | -1.88143 | -1.39865 | 0.10894 | 0.19567 | 0.19697 | 0.28063 | 6 | 2.93229 | 0.55262 | 0.15944 | 0.288514 | Not Significant | NS | #N/A |
| 194 | A0A5F9D3G3 | Cullin-5 OS=Oryctolagus cuniculus OX=9986 GN=C | CUL5 | RABBIT | 0.8476 | 0.15025 | -0.12278 | 0.45747 | 0.70691 | #N/A | -0.64164 | 0.2047 | 0 | -0.12867 | -0.54929 | -2.14153 | -1.5871 | 0.06948 | 0.14378 | 0.16349 | 0.24288 | 7 | 2.93229 | 0.55262 | 0.1462 | 0.266184 | Not Significant | NS | #N/A |
| 2722 | G1SQN8 | #N/A | LSM14B | RABBIT | #N/A | #N/A | #N/A | #N/A | 0.36662 | #N/A | 0.32758 | -0.55152 | -0.32758 | #N/A | -0.55046 | -1.04351 | -0.74165 | 0.40626 | 0.49208 | 0.37337 | 0.43988 | 2 | 2.93229 | 0.55262 | 0.2087 | 0.41316 | Not Significant | NS | #N/A |
| 2340 | A0A5F9CBD0 | #N/A | #N/A | RABBIT | #N/A | #N/A | 0.26123 | #N/A | #N/A | -0.8063 | #N/A | -0.0461 | #N/A | -0.01826 | -0.55145 | -1.06804 | -0.7462 | 0.39734 | 0.48956 | 0.36958 | 0.43948 | 2 | 2.93229 | 0.55262 | 0.19994 | 0.409611 | Not Significant | NS | #N/A |
| 2566 | G1SZH8 | #N/A | VPS52 | RABBIT | #N/A | #N/A | #N/A | 0.31358 | #N/A | #N/A | #N/A | 0.45463 | -0.37122 | -0.80189 | -0.55307 | -0.75012 | -0.68157 | 0.53142 | 0.52619 | 0.42694 | 0.45323 | 2 | 2.93229 | 0.55262 | 0.40773 | 0.493865 | Not Significant | NS | #N/A |
| 119 | G1SID3 | Vacuolar protein sorting 13 homolog C OS=Oryctola | VPS13C | RABBIT | 0.61025 | -0.80199 | 0.17065 | -0.38371 | 1.39643 | #N/A | -0.45258 | 0.01928 | 0.11631 | -1.10781 | -0.55453 | -1.10979 | -1.11044 | 0.30375 | 0.29297 | 0.32493 | 0.34299 | 7 | 2.93229 | 0.55262 | 0.55482 | 0.554171 | Not Significant | NS | #N/A |
| 863 | G1TR30 | Microtubule associated protein 1 light chain 3 beta | #N/A | RABBIT | 0.51887 | 0.97271 | 0.04986 | 1.05154 | -0.07112 | -0.00038 | -0.37092 | 0.13106 | -0.01467 | 0.00038 | -0.55528 | -2.26811 | -1.72892 | 0.05305 | 0.11192 | 0.15329 | 0.21129 | 8 | 2.93229 | 0.55262 | 0.14984 | 0.257875 | Not Significant | NS | #N/A |
| 2410 | G1SV62 | #N/A | CDC16 | RABBIT | #N/A | #N/A | 0.51277 | #N/A | #N/A | #N/A | #N/A | -0.06437 | -0.02065 | #N/A | -0.55528 | -14.6677 | -0.70607 | 0.04334 | 0.51973 | 0.14581 | 0.45188 | 1 | 2.93229 | 0.55262 | 0.00096 | 0.412327 | Not Significant | NS | #N/A |
| 2766 | G1TFZ7 | #N/A | PPM1G | RABBIT | #N/A | #N/A | #N/A | 0.25038 | #N/A | #N/A | #N/A | #N/A | #N/A | -0.30802 | -0.5584 | #N/A | -0.53115 | #N/A | 0.63291 | #N/A | 0.48899 | 0 | 2.93229 | 0.55262 | #N/A | 0.552617 | Not Significant | NS | #N/A |
| 2821 | A0A5F9DJT5 | #N/A | #N/A | RABBIT | #N/A | #N/A | #N/A | #N/A | 0.24071 | #N/A | #N/A | -0.31846 | #N/A | #N/A | -0.55917 | #N/A | -0.53189 | #N/A | 0.63246 | #N/A | 0.4889 | 0 | 2.93229 | 0.55262 | #N/A | 0.552617 | Not Significant | NS | #N/A |
| 1843 | A0A5F9DGY7 | #N/A | ELAVL4 | RABBIT | #N/A | 0.3822 | -0.01373 | 2.79841 | #N/A | -0.54051 | -1.16904 | 2.17168 | -1.19232 | 3.2114 | -0.55938 | -0.40452 | -0.47596 | 0.69986 | 0.64553 | 0.48373 | 0.49476 | 6 | 2.93229 | 0.55262 | 3.58546 | 2.589842 | Not Significant | NS | #N/A |
| 1611 | P9WF09 | Toxin RelK OS=Mycobacterium tuberculosis (strain | #N/A | MT | 0.61882 | 1.10885 | #N/A | #N/A | -0.36633 | -0.32208 | #N/A | #N/A | -0.11544 | 0.11544 | -0.56114 | -1.24201 | -1.07276 | 0.28208 | 0.3193 | 0.31581 | 0.35646 | 4 | 2.93229 | 0.55262 | 0.30619 | 0.410423 | Not Significant | NS | #N/A |
| 1679 | G1SK48 | #N/A | BLMH | RABBIT | #N/A | 1.02777 | #N/A | -0.05764 | #N/A | #N/A | -0.07651 | #N/A | #N/A | #N/A | -0.56158 | -0.59742 | -0.6117 | 0.65717 | 0.57433 | 0.4708 | 0.46967 | 1 | 2.93229 | 0.55262 | 0.58906 | 0.561885 | Not Significant | NS | #N/A |
| 678 | G1T0R2 | Potassium channel tetramerization domain contain | KCTD16 | RABBIT | 0.06222 | -1.10931 | -0.31959 | 0.22215 | 0.97214 | #N/A | -1.51665 | -0.06437 | 0.39244 | -1.19873 | -0.56235 | -1.01281 | -1.04301 | 0.34487 | 0.32166 | 0.34542 | 0.35646 | 7 | 2.93229 | 0.55262 | 0.68508 | 0.645974 | Not Significant | NS | #N/A |
| 1336 | A0A5F9DKL5 | Telomerase-binding protein EST1A OS=Oryctolagus | SMG6 | RABBIT | 1.1199 | #N/A | 0.56648 | -1.18201 | #N/A | -0.39583 | #N/A | #N/A | #N/A | #N/A | -0.56396 | -0.40648 | -0.51088 | 0.72376 | 0.63147 | 0.49085 | 0.4889 | 2 | 2.93229 | 0.55262 | 1.44372 | 0.91395 | Not Significant | NS | #N/A |
| 1495 | G1SFB5 | Leucine rich repeat and Ig domain containing 4 OS= | LINGO4 | RABBIT | -0.94946 | #N/A | -0.18898 | #N/A | 3.37411 | 0.7435 | 0.24324 | -0.45367 | 0.61074 | -0.24324 | -0.56511 | -0.55311 | -0.63263 | 0.60017 | 0.54283 | 0.45128 | 0.46166 | 6 | 2.93229 | 0.55262 | 1.95724 | 1.496133 | Not Significant | NS | #N/A |
| 257 | G1TGK9 | RNA helicase OS=Oryctolagus cuniculus OX=9986 ( | DHX9 | RABBIT | -0.51227 | -0.0059 | 0.93159 | 1.096 | 0.76981 | -0.87644 | 0.0977 | 0.47021 | -0.0977 | -0.15029 | -0.56715 | -1.50132 | -1.40167 | 0.17167 | 0.18876 | 0.25051 | 0.27623 | 8 | 2.93229 | 0.55262 | 0.35677 | 0.409298 | Not Significant | NS | #N/A |
| 300 | G1SHV9 | Proteasome subunit beta OS=Oryctolagus cuniculu | PSMB3 | RABBIT | 0.66613 | 0.42304 | 0.00878 | -0.15043 | 0.59288 | #N/A | 0.29608 | -0.37169 | -0.96438 | 0 | -0.56808 | -1.88835 | -1.53367 | 0.10092 | 0.15632 | 0.19016 | 0.25357 | 7 | 2.93229 | 0.55262 | 0.20111 | 0.304887 | Not Significant | NS | #N/A |
| 2583 | A0A5F9CLS4 | #N/A | ARHGEF11 | RABBIT | #N/A | #N/A | #N/A | 0.08031 | #N/A | #N/A | #N/A | #N/A | 0 | -0.97679 | -0.5687 | -0.67229 | -0.63579 | 0.62319 | 0.56003 | 0.45777 | 0.46562 | 1 | 2.93229 | 0.55262 | 0.47705 | 0.533401 | Not Significant | NS | #N/A |
| 1018 | G1U8P2 | Ribosomal protein L19 OS=Oryctolagus cuniculus ( | #N/A | RABBIT | 2.80059 | #N/A | -0.22608 | #N/A | -0.47628 | #N/A | #N/A | -0.08486 | 0.02049 | 0.45627 | -0.56877 | -0.53355 | -0.63137 | 0.62192 | 0.54804 | 0.45769 | 0.46295 | 4 | 2.93229 | 0.55262 | 1.70459 | 1.217317 | Not Significant | NS | #N/A |
| 302 | G1SG65 | Guanylate cyclase soluble subunit beta-1 OS=Oryc | GUCY1B1 | RABBIT | 0.40197 | 0.31336 | -0.44555 | 0.97485 | 0.39153 | -0.23217 | -0.69133 | -0.5879 | 0.41269 | -0.11554 | -0.57008 | -1.9041 | -1.61317 | 0.09338 | 0.13517 | 0.18402 | 0.23421 | 8 | 2.93229 | 0.55262 | 0.2241 | 0.312213 | Not Significant | NS | #N/A |

| | | | | | | | | | | | | | | | | | | | | | | | | | | | | | |
|---|---|---|---|---|---|---|---|---|---|---|---|---|---|---|---|---|---|---|---|---|---|---|---|---|---|---|---|---|---|
| 759 | G1U2J2 | Heterogeneous nuclear ribonucleoprotein H2 OS=C | #N/A | RABBIT | -1.92443 | #N/A | #N/A | 1.40815 | #N/A | 0.34487 | 0 | -3.02167 | 0.48115 | -1.94667 | -0.57033 | -0.38852 | -0.46547 | 0.71362 | 0.65411 | 0.48897 | 0.498 | 5 | 2.93229 | 0.55262 | 3.07835 | 2.144676 | Not Significant | NS | #N/A |
| 1545 | O77822 | 3',5'-cyclic-AMP phosphodiesterase (Fragment) OS | PDE4A | RABBIT | 0.17679 | -0.03551 | #N/A | -0.01528 | 0.05161 | -1.01014 | #N/A | #N/A | -0.04236 | #N/A | -0.57065 | -1.87155 | -1.19259 | 0.13459 | 0.27225 | 0.21791 | 0.33034 | 4 | 2.93229 | 0.55262 | 0.12396 | 0.305276 | Not Significant | NS | #N/A |
| 2022 | A0A5F9DRW5 | #N/A | LYST | RABBIT | #N/A | 0.38501 | #N/A | #N/A | #N/A | #N/A | -0.18719 | #N/A | #N/A | #N/A | -0.5722 | #N/A | -0.54428 | #N/A | 0.6249 | #N/A | 0.48613 | 0 | 2.93229 | 0.55262 | #N/A | 0.552617 | Not Significant | NS | #N/A |
| 1241 | G1T9W4 | E3 ubiquitin-protein ligase PPP1R11 OS=Oryctolagu | PPP1R11 | RABBIT | 1.54625 | #N/A | -0.42354 | -0.05764 | #N/A | #N/A | -0.80785 | #N/A | 0.37247 | #N/A | -0.57271 | -0.63897 | -0.71933 | 0.56828 | 0.49928 | 0.43895 | 0.44285 | 3 | 2.93229 | 0.55262 | 0.96402 | 0.760667 | Not Significant | NS | #N/A |
| 244 | G1TEH2 | V-type proton ATPase subunit C OS=Oryctolagus cu | ATP6V1C1 | RABBIT | -0.02189 | -0.39015 | 0.28608 | -0.02409 | 0.33159 | -1.66373 | 0.14221 | 0.35555 | -0.39983 | -1.11622 | -0.57271 | -1.42726 | -1.36084 | 0.19135 | 0.20096 | 0.26271 | 0.28391 | 8 | 2.93229 | 0.55262 | 0.40254 | 0.442793 | Not Significant | NS | #N/A |
| 1664 | G1SXB8 | #N/A | AP1B1 | RABBIT | #N/A | 0.34678 | -0.04952 | 0.5179 | 0.3671 | -1.37499 | 0 | 0.07597 | -0.05802 | -0.02986 | -0.57295 | -1.73714 | -1.47893 | 0.12593 | 0.17016 | 0.21238 | 0.26283 | 7 | 2.93229 | 0.55262 | 0.24174 | 0.333519 | Not Significant | NS | #N/A |
| 1296 | P71898 | Conserved protein OS=Mycobacterium tuberculosi: | #N/A | MT | 0.23411 | #N/A | #N/A | -0.21819 | #N/A | #N/A | 0.16055 | -1.2956 | #N/A | #N/A | -0.57549 | -0.75485 | -0.76614 | 0.52912 | 0.47863 | 0.42603 | 0.43349 | 2 | 2.93229 | 0.55262 | 0.58124 | 0.564223 | Not Significant | NS | #N/A |
| 1122 | G1TF35 | Protein phosphatase, Mg2+/Mn2+ dependent 1E OS | PPM1E | RABBIT | 0.01166 | 0.36573 | 0.20958 | 0.1554 | #N/A | 0.4377 | -0.7014 | -1.25886 | -0.38092 | -0.04753 | -0.5758 | -1.73234 | -1.48041 | 0.12682 | 0.16977 | 0.2129 | 0.26263 | 7 | 2.93229 | 0.55262 | 0.24551 | 0.336174 | Not Significant | NS | #N/A |
| 1465 | G1T911 | Profilin OS=Oryctolagus cuniculus OX=9986 PE=3 S | #N/A | RABBIT | 1.00674 | #N/A | #N/A | -0.02389 | #N/A | -0.03375 | -0.13881 | #N/A | #N/A | #N/A | -0.5777 | -1.11529 | -0.87357 | 0.38076 | 0.42284 | 0.36082 | 0.40913 | 2 | 2.93229 | 0.55262 | 0.26831 | 0.437332 | Not Significant | NS | #N/A |
| 2526 | U3KNB8 | #N/A | FARP1 | RABBIT | #N/A | #N/A | #N/A | 0.26321 | #N/A | #N/A | -0.32085 | #N/A | #N/A | #N/A | -0.58406 | #N/A | -0.55556 | #N/A | 0.61808 | #N/A | 0.48367 | 0 | 2.93229 | 0.55262 | #N/A | 0.552617 | Not Significant | NS | #N/A |
| 2047 | G1SP32 | #N/A | HIBCH | RABBIT | #N/A | 0.33995 | #N/A | #N/A | #N/A | -0.42626 | #N/A | -0.06437 | #N/A | #N/A | -0.58526 | -1.86742 | -0.72981 | 0.31299 | 0.5066 | 0.33019 | 0.44704 | 1 | 2.93229 | 0.55262 | 0.06548 | 0.428736 | Not Significant | NS | #N/A |
| 1582 | G1T791 | ADAM metallopeptidase with thrombospondin type | ADAMTS18 | RABBIT | 0.2996 | #N/A | #N/A | #N/A | #N/A | #N/A | #N/A | #N/A | -0.28794 | #N/A | -0.58754 | #N/A | -0.55887 | #N/A | 0.61608 | #N/A | 0.48277 | 0 | 2.93229 | 0.55262 | #N/A | 0.552617 | Not Significant | NS | #N/A |
| 338 | A0A5F9D1J7 | Methanethiol oxidase OS=Oryctolagus cuniculus O: | SELENBP1 | RABBIT | 0.06126 | 0.848 | 0.12105 | 0.66056 | 0.48589 | -0.76708 | -0.88209 | -0.74455 | 2.72453 | -1.09242 | -0.58767 | -0.79665 | -0.86884 | 0.44865 | 0.40361 | 0.39048 | 0.40294 | 8 | 2.93229 | 0.55262 | 1.36045 | 1.143769 | Not Significant | NS | #N/A |
| 1607 | U3KNX7 | Uncharacterized protein OS=Oryctolagus cuniculus | #N/A | RABBIT | #N/A | #N/A | -0.28571 | #N/A | 1.33423 | #N/A | #N/A | -0.06437 | #N/A | #N/A | -0.58862 | -0.41958 | -0.55654 | 0.74709 | 0.60799 | 0.49801 | 0.47919 | 1 | 2.93229 | 0.55262 | 1.31209 | 0.745756 | Not Significant | NS | #N/A |
| 2384 | A0A5F9DIT6 | #N/A | CRYBG2 | RABBIT | #N/A | #N/A | 0.06228 | 0.83042 | 0.09499 | -0.80894 | -0.67524 | 0.1354 | -0.35682 | 0.40302 | -0.58975 | -1.63909 | -1.37593 | 0.15231 | 0.20236 | 0.23344 | 0.28485 | 6 | 2.93229 | 0.55262 | 0.24273 | 0.344459 | Not Significant | NS | #N/A |
| 384 | G1SNM5 | RAB11 binding and LisH domain, coiled-coil and HE | RELCH | RABBIT | 0.14716 | 0.19782 | #N/A | #N/A | #N/A | #N/A | -0.41876 | #N/A | #N/A | #N/A | -0.59125 | -13.476 | -0.75173 | 0.04715 | 0.4947 | 0.14885 | 0.44101 | 1 | 2.93229 | 0.55262 | 0.00128 | 0.41241 | Not Significant | NS | #N/A |
| 1939 | G1SN83 | #N/A | LAMB2 | RABBIT | #N/A | 0.54395 | -0.9975 | #N/A | 1.49607 | #N/A | 0.22319 | -0.73168 | #N/A | -0.22319 | -0.5914 | -0.76103 | -0.8328 | 0.48903 | 0.43271 | 0.40978 | 0.41308 | 4 | 2.93229 | 0.55262 | 0.90585 | 0.756434 | Not Significant | NS | #N/A |
| 677 | G1TJP8 | Nuclear casein kinase and cyclin dependent kinase | NUCKS1 | RABBIT | 1.145 | 2.27024 | -2.44567 | 0.71294 | -0.23851 | -2.13774 | -0.7024 | 0.16076 | -0.28713 | 1.44841 | -0.59242 | -0.60147 | -0.67547 | 0.56418 | 0.51341 | 0.4379 | 0.44921 | 8 | 2.93229 | 0.55262 | 2.42538 | 1.92306 | Not Significant | NS | #N/A |
| 1633 | A0A5F9CF61 | Coiled-coil domain-containing protein OS=Oryctola | #N/A | RABBIT | 0.94513 | -0.86118 | -0.43766 | 0.41854 | 3.48794 | -1.65712 | 2.78668 | -0.66107 | 0 | #N/A | -0.59343 | -0.49303 | -0.56724 | 0.63708 | 0.58314 | 0.46416 | 0.47236 | 7 | 2.93229 | 0.55262 | 3.21953 | 2.43218 | Not Significant | NS | #N/A |
| 2454 | G1T9C7 | #N/A | CCDC198 | RABBIT | #N/A | #N/A | 0.38306 | #N/A | #N/A | #N/A | -0.21241 | #N/A | #N/A | #N/A | -0.59547 | #N/A | -0.56642 | #N/A | 0.61156 | #N/A | 0.48032 | 0 | 2.93229 | 0.55262 | #N/A | 0.552617 | Not Significant | NS | #N/A |
| 1088 | G1SF40 | N-terminal EF-hand calcium binding protein 1 OS=C | NECAB1 | RABBIT | -0.00815 | 0.95702 | -1.20242 | 1.08868 | 0.00643 | -1.10939 | 0.53407 | -1.22668 | 0.27445 | -0.61557 | -0.59694 | -1.09334 | -1.13333 | 0.30607 | 0.28132 | 0.32524 | 0.33517 | 8 | 2.93229 | 0.55262 | 0.74522 | 0.693561 | Not Significant | NS | #N/A |
| 826 | A0A5F9D3N8 | Uncharacterized protein OS=Oryctolagus cuniculus | #N/A | RABBIT | -0.58989 | 1.0519 | -0.56833 | 1.99226 | -1.01682 | -3.84598 | -0.85789 | 0.9535 | 1.02569 | 0.60155 | -0.59845 | -0.55117 | -0.62325 | 0.59657 | 0.5459 | 0.44987 | 0.46295 | 8 | 2.93229 | 0.55262 | 2.94731 | 2.304999 | Not Significant | NS | #N/A |
| 7 | G1U974 | Dihydropyrimidinase-related protein 2 OS=Oryctola | DPYSL2 | RABBIT | 0.37239 | 0.56041 | 0.06122 | 0.16112 | 0.14391 | -1.07044 | -0.27024 | 0.01723 | -0.0816 | -0.29611 | -0.60004 | -2.82805 | -1.97578 | 0.02222 | 0.07396 | 0.11776 | 0.17686 | 8 | 2.93229 | 0.55262 | 0.11255 | 0.230583 | Not Significant | NS | #N/A |
| 2765 | G1T163 | #N/A | CASTOR2 | RABBIT | #N/A | #N/A | #N/A | 0.27285 | #N/A | -0.33049 | #N/A | #N/A | #N/A | #N/A | -0.60334 | #N/A | -0.5739 | #N/A | 0.60709 | #N/A | 0.47901 | 0 | 2.93229 | 0.55262 | #N/A | 0.552617 | Not Significant | NS | #N/A |
| 2703 | P9WGL1 | #N/A | #N/A | MT | #N/A | #N/A | #N/A | 0.42282 | #N/A | -0.54992 | #N/A | #N/A | 0.19913 | -0.19913 | -0.60613 | -1.40063 | -0.84545 | 0.29631 | 0.43694 | 0.32189 | 0.41582 | 2 | 2.93229 | 0.55262 | 0.14046 | 0.385489 | Not Significant | NS | #N/A |
| 240 | G1SCY1 | Synaptosome associated protein 91 OS=Oryctolagu | SNAP91 | RABBIT | -0.08234 | 0.77887 | 0.58073 | -0.00503 | 0.03513 | -1.09602 | -0.62524 | -0.11287 | 0.63144 | -0.5297 | -0.60795 | -1.79505 | -1.6064 | 0.11038 | 0.13666 | 0.19806 | 0.23549 | 8 | 2.93229 | 0.55262 | 0.28676 | 0.35807 | Not Significant | NS | #N/A |
| 1355 | G1SPK0 | Peflin OS=Oryctolagus cuniculus OX=9986 GN=PEF | PEF1 | RABBIT | #N/A | #N/A | #N/A | -0.05764 | 2.31312 | #N/A | #N/A | 2.60068 | -0.65012 | -0.39131 | -0.60799 | -0.37748 | -0.49001 | 0.73094 | 0.64172 | 0.49252 | 0.49339 | 3 | 2.93229 | 0.55262 | 3.11306 | 1.84745 | Not Significant | NS | #N/A |
| 2181 | G1SIT5 | #N/A | RPL35 | RABBIT | #N/A | #N/A | 0.38246 | -0.11009 | 0.32815 | -0.33201 | -1.2228 | 0.15615 | -0.70043 | 0.05245 | -0.6095 | -1.70877 | -1.42785 | 0.13835 | 0.18735 | 0.22122 | 0.27558 | 6 | 2.93229 | 0.55262 | 0.23855 | 0.341654 | Not Significant | NS | #N/A |
| 1379 | G1TI53 | Unconventional myosin-VI OS=Oryctolagus cunicul | MYO6 | RABBIT | -2.1999 | #N/A | 1.38343 | #N/A | 1.03501 | -2.46291 | 0.37797 | 0.10076 | -0.16513 | -0.54054 | -0.61082 | -0.57042 | -0.656 | 0.5891 | 0.52836 | 0.44648 | 0.45385 | 6 | 2.93229 | 0.55262 | 2.15001 | 1.625621 | Not Significant | NS | #N/A |
| 1985 | A0A5F9DE08 | #N/A | MCM8 | RABBIT | #N/A | #N/A | 0.39074 | #N/A | #N/A | #N/A | -0.22009 | #N/A | #N/A | #N/A | -0.61083 | #N/A | -0.58103 | #N/A | 0.60286 | #N/A | 0.47806 | 0 | 2.93229 | 0.55262 | #N/A | 0.552617 | Not Significant | NS | #N/A |
| 463 | G1SFR8 | Small ribosomal subunit protein eS12 OS=Oryctola | RPS12 | RABBIT | -0.37252 | 0.11949 | 0.91264 | 0.02069 | 0.11907 | -0.72153 | 0.34529 | 0.0238 | -1.42299 | -0.49341 | -0.61364 | -1.65427 | -1.5342 | 0.13667 | 0.1534 | 0.21984 | 0.25289 | 8 | 2.93229 | 0.55262 | 0.344 | 0.399955 | Not Significant | NS | #N/A |
| 2794 | G1SU70 | #N/A | MSH5 | RABBIT | #N/A | #N/A | #N/A | #N/A | 0.27118 | #N/A | #N/A | -0.34893 | #N/A | #N/A | -0.62011 | #N/A | -0.58985 | #N/A | 0.59765 | #N/A | 0.47523 | 0 | 2.93229 | 0.55262 | #N/A | 0.552617 | Not Significant | NS | #N/A |
| 1858 | A0A5F9CWH7 | #N/A | ZBTB18 | RABBIT | #N/A | 0.50639 | #N/A | #N/A | #N/A | #N/A | #N/A | -0.37294 | -0.34666 | 0.37497 | -0.62127 | -1.26769 | -0.84903 | 0.33252 | 0.43512 | 0.33764 | 0.4147 | 2 | 2.93229 | 0.55262 | 0.18013 | 0.401577 | Not Significant | NS | #N/A |
| 1677 | G1SV22 | #N/A | PEBP1 | RABBIT | #N/A | 2.77297 | -1.42327 | 0.07733 | #N/A | -0.13497 | #N/A | -1.01383 | #N/A | 0.71167 | -0.62139 | -0.46903 | -0.57482 | 0.66347 | 0.58358 | 0.47235 | 0.47236 | 4 | 2.93229 | 0.55262 | 2.63277 | 1.752885 | Not Significant | NS | #N/A |
| 2171 | A0A5F9CRR4 | #N/A | MPI | RABBIT | #N/A | #N/A | 0.15964 | 1.16197 | #N/A | #N/A | 0.01101 | -0.16193 | 1.28756 | -0.97954 | -0.62153 | -0.81005 | -0.86607 | 0.46335 | 0.41541 | 0.39783 | 0.40833 | 4 | 2.93229 | 0.55262 | 0.78495 | 0.686678 | Not Significant | NS | #N/A |
| 242 | G1T3Z6 | Protein kinase cAMP-dependent type II regulatory su | PRKAR2B | RABBIT | 0.78297 | -0.20006 | 0.1878 | 0.78646 | 0.73395 | -0.29562 | -0.01715 | -0.28596 | 0.26824 | -0.48825 | -0.62197 | -2.60352 | -1.95657 | 0.03145 | 0.07642 | 0.12952 | 0.18098 | 8 | 2.93229 | 0.55262 | 0.14268 | 0.252633 | Not Significant | NS | #N/A |
| 206 | G1TTB9 | Protein phosphatase 3 regulatory subunit B, alpha ( | PPP3R1 | RABBIT | 0.53124 | 0.31495 | -0.56554 | 0.26969 | -0.13051 | -2.47233 | 0.18179 | -0.41051 | 0.20452 | -0.19617 | -0.6225 | -1.16606 | -1.20282 | 0.27717 | 0.25445 | 0.31248 | 0.32053 | 8 | 2.93229 | 0.55262 | 0.71249 | 0.669611 | Not Significant | NS | #N/A |
| 1834 | G1TE69 | #N/A | SRSF1 | RABBIT | #N/A | #N/A | 1.30873 | -1.17926 | 1.04178 | -0.96621 | -0.18988 | 0.12551 | -0.92714 | 0.78052 | -0.62586 | -0.8636 | -0.93345 | 0.42098 | 0.37513 | 0.3795 | 0.38769 | 6 | 2.93229 | 0.55262 | 0.98474 | 0.842886 | Not Significant | NS | #N/A |
| 2855 | A0A5F9DFP5 | #N/A | ZNF567 | RABBIT | #N/A | #N/A | #N/A | #N/A | 0.30699 | -0.32037 | #N/A | #N/A | #N/A | #N/A | -0.62736 | #N/A | -0.59675 | #N/A | 0.5936 | #N/A | 0.47404 | 0 | 2.93229 | 0.55262 | #N/A | 0.552617 | Not Significant | NS | #N/A |
| 2088 | G1T303 | #N/A | TOP3B | RABBIT | #N/A | 0.39115 | -0.02268 | 1.05059 | #N/A | 1.78152 | #N/A | #N/A | -1.4609 | -0.78746 | -0.62863 | -0.60671 | -0.71394 | 0.5768 | 0.49859 | 0.44179 | 0.44285 | 4 | 2.93229 | 0.55262 | 1.61037 | 1.162951 | Not Significant | NS | #N/A |
| 2506 | B7NZM4 | #N/A | DRG1 | RABBIT | #N/A | #N/A | #N/A | 0.287 | #N/A | #N/A | #N/A | #N/A | -0.34464 | #N/A | -0.63165 | #N/A | -0.60082 | #N/A | 0.59122 | #N/A | 0.47335 | 0 | 2.93229 | 0.55262 | #N/A | 0.552617 | Not Significant | NS | #N/A |
| 2574 | G1U2V1 | #N/A | UNC119 | RABBIT | #N/A | #N/A | #N/A | 0.28808 | #N/A | #N/A | -0.34572 | #N/A | #N/A | #N/A | -0.6338 | #N/A | -0.60287 | #N/A | 0.59002 | #N/A | 0.47294 | 0 | 2.93229 | 0.55262 | #N/A | 0.552617 | Not Significant | NS | #N/A |
| 2604 | A0A5F9DJ36 | #N/A | ASTN2 | RABBIT | #N/A | #N/A | #N/A | 0.28976 | #N/A | #N/A | -0.3474 | #N/A | #N/A | #N/A | -0.63716 | #N/A | -0.60606 | #N/A | 0.58816 | #N/A | 0.47237 | 0 | 2.93229 | 0.55262 | #N/A | 0.552617 | Not Significant | NS | #N/A |
| 1345 | A0A5F9C5P4 | Golgin subfamily A conserved domain-containing pı | #N/A | RABBIT | -0.912 | 1.59722 | #N/A | 0.52974 | -0.01338 | #N/A | 0.42798 | -0.62858 | -0.82486 | #N/A | -0.64222 | -0.91627 | -0.98071 | 0.40156 | 0.3557 | 0.3719 | 0.37715 | 5 | 2.93229 | 0.55262 | 0.84218 | 0.735136 | Not Significant | NS | #N/A |
| 2517 | G1TIM6 | #N/A | NOVA2 | RABBIT | #N/A | #N/A | #N/A | 0.29282 | #N/A | #N/A | #N/A | #N/A | #N/A | -0.35045 | -0.64327 | #N/A | -0.61188 | #N/A | 0.58479 | #N/A | 0.47237 | 0 | 2.93229 | 0.55262 | #N/A | 0.552617 | Not Significant | NS | #N/A |
| 2528 | G1SKL8 | #N/A | CRYZL1 | RABBIT | #N/A | #N/A | #N/A | 0.34344 | #N/A | -1.3703 | 0 | 1.25908 | #N/A | -1.10534 | -0.64758 | -0.48297 | -0.57907 | 0.66217 | 0.58384 | 0.4723 | 0.47236 | 3 | 2.93229 | 0.55262 | 1.43826 | 1.000494 | Not Significant | NS | #N/A |
| 981 | Q7YQK3 | Amino acid transporter heavy chain SLC3A2 OS=Or | SLC3A2 | RABBIT | -0.78989 | 2.07276 | #N/A | -0.06847 | 1.07482 | -0.97455 | 0.12564 | -0.06437 | 0.60111 | -0.06597 | -0.64793 | -1.03666 | -1.09719 | 0.33437 | 0.29845 | 0.33859 | 0.34592 | 7 | 2.93229 | 0.55262 | 0.86811 | 0.774971 | Not Significant | NS | #N/A |
| 68 | B7NZQ6 | Rab GDP dissociation inhibitor OS=Oryctolagus cun | GDI1 | RABBIT | 0.4833 | 1.3286 | 0.02691 | 0.87495 | 0.13035 | -0.25184 | -0.26723 | 0.44917 | -0.14705 | -0.18189 | -0.64859 | -2.34985 | -1.91227 | 0.04669 | 0.08239 | 0.14885 | 0.18516 | 8 | 2.93229 | 0.55262 | 0.19046 | 0.287599 | Not Significant | NS | #N/A |
| 1573 | A0A5F9DCI7 | Gamma-glutamyltransferase 5 OS=Oryctolagus cur | GGT5 | RABBIT | 0.37218 | #N/A | #N/A | -0.05764 | 0.56129 | #N/A | #N/A | -0.40655 | -0.31503 | #N/A | -0.65273 | -2.73277 | -1.28887 | 0.07177 | 0.24542 | 0.16449 | 0.31496 | 3 | 2.93229 | 0.55262 | 0.06846 | 0.307776 | Not Significant | NS | #N/A |
| 264 | G1SGM2 | Protein arginine N-methyltransferase 5 OS=Oryctola | PRMT5 | RABBIT | 0.84045 | -0.61099 | -0.11124 | -0.04924 | 0.45265 | -2.61392 | -0.0084 | 0.08279 | 0.06774 | -0.27333 | -0.65335 | -1.13249 | -1.18723 | 0.29023 | 0.26029 | 0.3201 | 0.32441 | 8 | 2.93229 | 0.55262 | 0.83207 | 0.757117 | Not Significant | NS | #N/A |
| 2204 | A0A5F9CYW1 | #N/A | AP1S2 | RABBIT | #N/A | #N/A | 0.27068 | 0.39689 | #N/A | #N/A | 0.02614 | -0.09051 | -0.98626 | -0.22773 | -0.65337 | -1.89672 | -1.3233 | 0.13073 | 0.22771 | 0.21527 | 0.30112 | 4 | 2.93229 | 0.55262 | 0.15822 | 0.325044 | Not Significant | NS | #N/A |
| 957 | A0A5F9C4F5 | BRICK1 subunit of SCAR/WAVE actin nucleating cor | BRK1 | RABBIT | 0.14153 | -0.04927 | 0.50576 | 0.39426 | 0.35031 | -0.58708 | -1.1674 | 0.42616 | -0.47143 | -0.12987 | -0.65444 | -2.32883 | -1.91264 | 0.04825 | 0.08234 | 0.14885 | 0.18516 | 8 | 2.93229 | 0.55262 | 0.19743 | 0.292698 | Not Significant | NS | #N/A |
| 271 | G1SS33 | V-type proton ATPase subunit E 1 OS=Oryctolagus c | ATP6V1E1 | RABBIT | 0.58753 | 0.18326 | 0.18521 | -0.79509 | 0.65419 | -1.70293 | 0.53957 | 0.04296 | -0.75055 | -0.58982 | -0.65517 | -1.42405 | -1.41567 | 0.19225 | 0.18473 | 0.26339 | 0.27413 | 8 | 2.93229 | 0.55262 | 0.52918 | 0.535466 | Not Significant | NS | #N/A |
| 313 | G1SXL3 | STI1 domain-containing protein OS=Oryctolagus cu | #N/A | RABBIT | 0.76727 | 0.4287 | -0.0321 | 0.23741 | 0.20874 | -1.12647 | 0.19079 | -0.25516 | -0.20717 | -0.27287 | -0.65618 | -2.58926 | -2.01272 | 0.03215 | 0.06944 | 0.13058 | 0.17249 | 8 | 2.93229 | 0.55262 | 0.16056 | 0.265717 | Not Significant | NS | #N/A |
| 1667 | A0A5F9CFN6 | #N/A | #N/A | RABBIT | #N/A | 0.50725 | #N/A | #N/A | -0.01338 | #N/A | -0.40996 | #N/A | #N/A | #N/A | -0.65689 | -1.45691 | -0.80263 | 0.38294 | 0.46789 | 0.36172 | 0.42854 | 1 | 2.93229 | 0.55262 | 0.13553 | 0.446549 | Not Significant | NS | #N/A |
| 1506 | P48738 | Phosphatidylinositol transfer protein alpha isoform | PITPNA PIT | RABBIT | -1.44967 | 2.70243 | #N/A | #N/A | #N/A | #N/A | #N/A | 0.58736 | -0.65173 | #N/A | -0.65856 | -0.30397 | -0.44083 | 0.78986 | 0.67799 | 0.51035 | 0.50607 | 2 | 2.93229 | 0.55262 | 4.6938 | 2.23183 | Not Significant | NS | #N/A |
| 1195 | G1SDD5 | Fibroblast growth factor OS=Oryctolagus cuniculus | FGF2 FGF2 | RABBIT | 0.56259 | #N/A | 0.04276 | -0.00546 | 0.54516 | #N/A | 0 | -0.60864 | #N/A | -0.5091 | -0.65884 | -2.72525 | -1.66805 | 0.04151 | 0.13418 | 0.14427 | 0.23357 | 5 | 2.93229 | 0.55262 | 0.10019 | 0.267437 | Not Significant | NS | #N/A |
| 1542 | U3KPF9 | Microtubule associated scaffold protein 2 OS=Oryc | #N/A | RABBIT | 0.78223 | 0.1799 | #N/A | -0.15947 | 1.28132 | #N/A | 0.42089 | -0.04645 | -0.55903 | -0.36754 | -0.65902 | -1.71286 | -1.51134 | 0.13757 | 0.16524 | 0.22077 | 0.26016 | 6 | 2.93229 | 0.55262 | 0.29607 | 0.380287 | Not Significant | NS | #N/A |
| 289 | G1T2Y5 | Nucleoprotein TPR OS=Oryctolagus cuniculus OX=9 | TPR | RABBIT | -0.69753 | -1.99812 | 0.07313 | 0.03988 | 0.11337 | -6.55793 | 0.25651 | 0.86475 | 0.16687 | -0.49528 | -0.65916 | -0.46193 | -0.52955 | 0.65644 | 0.60701 | 0.4707 | 0.47901 | 8 | 2.93229 | 0.55262 | 5.09067 | 3.873461 | Not Significant | NS | #N/A |
| 2329 | A0A5F9D6I7 | #N/A | MPDU1 | RABBIT | #N/A | #N/A | 1.18351 | #N/A | -0.01338 | #N/A | #N/A | -0.07498 | #N/A | #N/A | -0.66004 | -0.63677 | -0.69911 | 0.63902 | 0.52362 | 0.46486 | 0.45286 | 1 | 2.93229 | 0.55262 | 0.71628 | 0.594238 | Not Significant | NS | #N/A |
| 2194 | A0A5F9CK08 | #N/A | KLC4 | RABBIT | #N/A | #N/A | -0.65728 | #N/A | 1.27719 | -1.53022 | 0.82793 | #N/A | #N/A | #N/A | -0.6611 | -0.43349 | -0.58626 | 0.70693 | 0.5835 | 0.48598 | 0.47236 | 2 | 2.93229 | 0.55262 | 2.32576 | 1.271611 | Not Significant | NS | #N/A |
| 38 | A0A5F9C2P9 | Ankyrin 2 OS=Oryctolagus cuniculus OX=9986 GN= | ANK2 | RABBIT | 0.7505 | -1.95265 | 0.46492 | 0.00318 | 0.79446 | -1.10611 | 0.31694 | -1.72873 | -0.06082 | -0.66964 | -0.66175 | -1.05447 | -1.12259 | 0.32247 | 0.28566 | 0.33441 | 0.33729 | 8 | 2.93229 | 0.55262 | 0.98461 | 0.868739 | Not Significant | NS | #N/A |
| 2829 | G1STZ5 | #N/A | LRRK2 | RABBIT | #N/A | #N/A | #N/A | #N/A | 0.2606 | #N/A | 0 | #N/A | #N/A | -0.80467 | -0.66293 | -0.9513 | -0.7698 | 0.51588 | 0.48505 | 0.42132 | 0.43748 | 1 | 2.93229 | 0.55262 | 0.32375 | 0.494414 | Not Significant | NS | #N/A |
| 21 | A0A5F9C6L0 | Spectrin beta chain OS=Oryctolagus cuniculus OX= | SPTBN2 | RABBIT | -0.1703 | -0.1958 | 1.0877 | -0.39099 | 0.89582 | -3.243 | 0.19371 | 0.95619 | -0.06056 | 0.06056 | -0.6639 | -0.83961 | -0.92349 | 0.4255 | 0.37569 | 0.38072 | 0.38803 | 8 | 2.93229 | 0.55262 | 1.56312 | 1.292081 | Not Significant | NS | #N/A |
| 1775 | G1SL80 | #N/A | UROD | RABBIT | #N/A | 0.43089 | #N/A | #N/A | #N/A | -0.23307 | #N/A | #N/A | #N/A | #N/A | -0.66396 | #N/A | -0.63156 | #N/A | 0.57346 | #N/A | 0.46967 | 0 | 2.93229 | 0.55262 | #N/A | 0.552617 | Not Significant | NS | #N/A |
| 1130 | A0A5F9CAN2 | High mobility group protein HMG-I/HMG-Y OS=Oryc | #N/A | RABBIT | 0.21231 | 0.32306 | 0.28898 | 0.24801 | -0.62982 | #N/A | -0.89539 | -0.06437 | -1.11472 | -0.22931 | -0.66446 | -2.19446 | -1.78846 | 0.06425 | 0.10419 | 0.15873 | 0.20323 | 7 | 2.93229 | 0.55262 | 0.20374 | 0.306735 | Not Significant | NS | #N/A |
| 1807 | G1U0Z2 | #N/A | ADAM11 | RABBIT | #N/A | -0.57658 | #N/A | -0.70964 | 3.38411 | #N/A | 0 | 0.06378 | #N/A | #N/A | -0.66741 | -0.38491 | -0.5048 | 0.72597 | 0.6319 | 0.49098 | 0.4889 | 3 | 2.93229 | 0.55262 | 3.60775 | 2.097622 | Not Significant | NS | #N/A |
| 1428 | G1SZP8 | phosphatidylinositol-4,5-bisphosphate 3-kinase OS | PIK3CA | RABBIT | 0.01166 | #N/A | #N/A | 0.40006 | #N/A | -0.02922 | #N/A | 0.16759 | #N/A | -1.52882 | -0.66935 | -0.94733 | -0.96603 | 0.41336 | 0.37174 | 0.37693 | 0.38582 | 3 | 2.93229 | 0.55262 | 0.59907 | 0.576108 | Not Significant | NS | #N/A |
| 2267 | A0A5F9DJL1 | #N/A | AFDN | RABBIT | #N/A | #N/A | #N/A | -0.05764 | -0.22185 | -3.83255 | #N/A | 0.84972 | 0.55172 | #N/A | -0.67062 | -0.34304 | -0.45627 | 0.7542 | 0.66442 | 0.50058 | 0.5003 | 3 | 2.93229 | 0.55262 | 4.58607 | 2.592365 | Not Significant | NS | #N/A |
| 885 | G1THY3 | Ubiquitin-conjugating enzyme E2 variant 1 OS=Oryc | #N/A | RABBIT | 0.41059 | #N/A | -0.31871 | 1.30181 | 0.98524 | 0 | -0.24379 | -0.19082 | -0.0416 | 0.0898 | -0.67201 | -2.09766 | -1.76025 | 0.07413 | 0.10907 | 0.16701 | 0.20798 | 7 | 2.93229 | 0.55262 | 0.22807 | 0.323887 | Not Significant | NS | #N/A |
| 1180 | A0A5F9DAQ1 | GTPase activating protein and VPS9 domains 1 OS= | GAPVD1 | RABBIT | #N/A | 0.19782 | 0.71304 | 2.38208 | -0.74936 | 1.05295 | -0.58897 | -0.66009 | 0.39673 | -0.38329 | -0.67243 | -0.97792 | -1.05449 | 0.36069 | 0.31664 | 0.35207 | 0.35506 | 7 | 2.93229 | 0.55262 | 1.0507 | 0.903653 | Not Significant | NS | #N/A |
| 2567 | A0A5F9D0G6 | #N/A | CDK6 | RABBIT | #N/A | #N/A | #N/A | 0.30808 | #N/A | #N/A | #N/A | #N/A | -0.36572 | #N/A | -0.6738 | #N/A | -0.64092 | #N/A | 0.56814 | #N/A | 0.46793 | 0 | 2.93229 | 0.55262 | #N/A | 0.552617 | Not Significant | NS | #N/A |
| 2121 | G1T1Z6 | #N/A | CYFIP2 | RABBIT | #N/A | #N/A | 0.21958 | #N/A | 0.25602 | -1.51769 | -0.52584 | 0.34333 | -0.04893 | #N/A | -0.67508 | -1.12049 | -1.08833 | 0.32523 | 0.31283 | 0.33513 | 0.35298 | 4 | 2.93229 | 0.55262 | 0.48399 | 0.513019 | Not Significant | NS | #N/A |
| 1648 | O53166 | Aconitate hydratase A OS=Mycobacterium tubercul | #N/A | MT | 0.42341 | 1.63895 | 0.11241 | #N/A | -0.01338 | #N/A | -0.2547 | -0.42409 | 0.27146 | #N/A | -0.67613 | -1.40909 | -1.31518 | 0.21786 | 0.22519 | 0.28083 | 0.29961 | 5 | 2.93229 | 0.55262 | 0.39469 | 0.453072 | Not Significant | NS | #N/A |
| 1028 | G1SEH8 | Cerebellin 1 precursor OS=Oryctolagus cuniculus C | CBLN1 | RABBIT | 0.42234 | 0.75646 | 0.086 | #N/A | 0.07127 | #N/A | #N/A | -0.15025 | #N/A | -0.53701 | -0.67765 | -2.50082 | -1.45243 | 0.06671 | 0.19009 | 0.16176 | 0.27623 | 4 | 2.93229 | 0.55262 | 0.0979 | 0.290241 | Not Significant | NS | #N/A |
| 2715 | G1U027 | #N/A | VAV1 | RABBIT | #N/A | #N/A | #N/A | 0.12318 | #N/A | -1.1156 | #N/A | #N/A | 0 | #N/A | -0.68098 | -0.70485 | -0.73625 | 0.60913 | 0.50309 | 0.45404 | 0.445 | 1 | 2.93229 | 0.55262 | 0.62228 | 0.570333 | Not Significant | NS | #N/A |
| 1795 | G1SH81 | #N/A | #N/A | RABBIT | #N/A | 0.43947 | #N/A | #N/A | #N/A | #N/A | #N/A | #N/A | -0.24165 | #N/A | -0.68112 | #N/A | -0.64788 | #N/A | 0.5642 | #N/A | 0.46687 | 0 | 2.93229 | 0.55262 | #N/A | 0.552617 | Not Significant | NS | #N/A |
| 838 | A0A5F9CH92 | Myelin basic protein OS=Oryctolagus cuniculus OX: | MBP | RABBIT | 0.01166 | #N/A | #N/A | #N/A | #N/A | #N/A | 2.08623 | #N/A | #N/A | -3.42693 | -0.68201 | -0.14284 | -0.26927 | 0.90967 | 0.80126 | 0.54361 | 0.5404 | 1 | 2.93229 | 0.55262 | 15.1975 | 4.27688 | Not Significant | NS | #N/A |
| 576 | G1SHD6 | S-adenosylmethionine synthase OS=Oryctolagus cı | MAT2A | RABBIT | 0.73383 | 0.93846 | #N/A | 0.27791 | -0.06847 | #N/A | 0.05508 | -0.1663 | -0.53027 | -0.20608 | -0.68233 | -2.6582 | -1.8573 | 0.03761 | 0.09648 | 0.13819 | 0.19688 | 6 | 2.93229 | 0.55262 | 0.13178 | 0.269931 | Not Significant | NS | #N/A |
| 1211 | G1SRE8 | X-ray repair cross complementing 5 OS=Oryctolagu | XRCC5 | RABBIT | -0.56673 | -1.15918 | 0.3039 | 0.54964 | 1.16338 | -4.10209 | 0.93195 | -0.19761 | -0.4658 | 0.71177 | -0.68256 | -0.68418 | -0.76911 | 0.51319 | 0.45812 | 0.42096 | 0.42417 | 8 | 2.93229 | 0.55262 | 2.48813 | 1.968984 | Not Significant | NS | #N/A |
| 524 | A0A5F9CBZ3 | S-phase kinase-associated protein 1 OS=Oryctolag | #N/A | RABBIT | 0.11753 | 0.70563 | 0.19305 | -0.31135 | -0.21735 | -3.4304 | -0.0224 | 0.3913 | 0.19647 | -0.08375 | -0.68726 | -0.93207 | -1.01647 | 0.37857 | 0.33137 | 0.35991 | 0.36166 | 8 | 2.93229 | 0.55262 | 1.35918 | 1.142843 | Not Significant | NS | #N/A |
| 1577 | G1U4R3 | Zinc finger protein 106 OS=Oryctolagus cuniculus C | ZNF106 | RABBIT | #N/A | #N/A | 0.48392 | -0.05764 | 0.58554 | #N/A | -0.39735 | #N/A | -0.3068 | #N/A | -0.68935 | -2.65222 | -1.34729 | 0.07685 | 0.22707 | 0.17055 | 0.30086 | 3 | 2.93229 | 0.55262 | 0.08107 | 0.31415 | Not Significant | NS | #N/A |
| 2122 | G1U9U0 | #N/A | CCT4 | RABBIT | #N/A | #N/A | 0.29739 | -0.05764 | 0.2909 | -1.43758 | #N/A | -0.07272 | -0.58636 | 0.04209 | -0.69052 | -1.68196 | -1.45444 | 0.1534 | 0.18422 | 0.23414 | 0.27407 | 5 | 2.93229 | 0.55262 | 0.28894 | 0.386413 | Not Significant | NS | #N/A |
| 831 | A0A5F9DR44 | Epiplakin 1 OS=Oryctolagus cuniculus OX=9986 PE | #N/A | RABBIT | 1.38549 | 0.80232 | -0.41 | -0.54845 | 0.34365 | #N/A | 0 | -0.75192 | #N/A | #N/A | -0.69056 | -1.07672 | -1.08878 | 0.33079 | 0.30822 | 0.33706 | 0.35174 | 5 | 2.93229 | 0.55262 | 0.58762 | 0.574682 | Not Significant | NS | #N/A |
| 2172 | A0A5F9D3U3 | #N/A | SEMA4D | RABBIT | #N/A | #N/A | 0.07336 | #N/A | 0.08391 | -3.69997 | #N/A | 1.48133 | 0.15084 | -0.38067 | -0.69075 | -0.41816 | -0.52219 | 0.6973 | 0.6178 | 0.4833 | 0.48367 | 4 | 2.93229 | 0.55262 | 3.63827 | 2.333072 | Not Significant | NS | #N/A |
| 1637 | G1SM25 | Src like adaptor 2 OS=Oryctolagus cuniculus OX=9! | SLA2 | RABBIT | 0.64732 | #N/A | #N/A | #N/A | 0.0276 | #N/A | #N/A | -0.66726 | #N/A | -0.04099 | -0.69158 | -1.56989 | -1.08374 | 0.25702 | 0.32859 | 0.30258 | 0.36045 | 2 | 2.93229 | 0.55262 | 0.19407 | 0.407228 | Not Significant | NS | #N/A |
| 1920 | G1SZ37 | #N/A | HIBADH | RABBIT | #N/A | 0.47281 | #N/A | -0.05764 | #N/A | #N/A | #N/A | #N/A | #N/A | -0.48452 | -0.69211 | -1.5066 | -0.84441 | 0.37305 | 0.44675 | 0.35712 | 0.4199 | 1 | 2.93229 | 0.55262 | 0.14069 | 0.447861 | Not Significant | NS | #N/A |
| 142 | A0A5F9DCB4 | SH3 domain containing GRB2 like 2, endophilin A1 ( | SH3GL2 | RABBIT | 0.85502 | 0.97836 | -0.39869 | 0.19538 | 0.10809 | -0.92623 | -0.52199 | 0.13748 | -0.29449 | -0.12147 | -0.69297 | -2.22084 | -1.91799 | 0.05711 | 0.08159 | 0.15449 | 0.18476 | 8 | 2.93229 | 0.55262 | 0.24341 | 0.326346 | Not Significant | NS | #N/A |
| 851 | A0A5F9CBG4 | DDB1 and CUL4 associated factor 11 OS=Oryctolag | DCAF11 | RABBIT | 1.47462 | 0.19782 | -0.0642 | #N/A | #N/A | #N/A | 0.02078 | #N/A | #N/A | -0.33699 | -0.69419 | -1.10546 | -1.06228 | 0.34965 | 0.32943 | 0.34662 | 0.36068 | 3 | 2.93229 | 0.55262 | 0.4732 | 0.512457 | Not Significant | NS | #N/A |
| 881 | A0A5F9D452 | IMD domain-containing protein OS=Oryctolagus cu | #N/A | RABBIT | 1.18161 | #N/A | 0.46251 | -0.05764 | -0.04135 | -0.97729 | -0.83726 | 0.94749 | 0.78133 | -1.45649 | -0.69473 | -1.13498 | -1.19589 | 0.29375 | 0.25951 | 0.32101 | 0.32441 | 7 | 2.93229 | 0.55262 | 0.83261 | 0.749948 | Not Significant | NS | #N/A |
| 1938 | A0A5F9DKX1 | #N/A | ANKRD13A | RABBIT | #N/A | 0.19782 | #N/A | #N/A | 0.26009 | #N/A | #N/A | #N/A | #N/A | -0.46846 | -0.69741 | -12.9327 | -0.88653 | 0.04913 | 0.42622 | 0.14906 | 0.41011 | 1 | 2.93229 | 0.55262 | 0.00194 | 0.412577 | Not Significant | NS | #N/A |

| | | | | | | | | | | | | | | | | | | | | | | | | | | | | | |
|---|---|---|---|---|---|---|---|---|---|---|---|---|---|---|---|---|---|---|---|---|---|---|---|---|---|---|---|---|---|
| 1497 | A0A5F9DIF7 | Uncharacterized protein OS=Oryctolagus cuniculus | #N/A | RABBIT | 0.01166 | #N/A | #N/A | #N/A | 0.42489 | #N/A | #N/A | #N/A | -0.48083 | #N/A | -0.6991 | -1.95354 | -0.86666 | 0.30119 | 0.4358 | 0.32455 | 0.41511 | 1 | 2.93229 | 0.55262 | 0.08538 | 0.433795 | Not Significant | NS | #N/A |
| 217 | B7NZE9 | Creatine kinase U-type, mitochondrial OS=Oryctola | CKMT1A | RABBIT | 0.60629 | 0.91578 | -0.31739 | 0.36631 | -0.0001 | -0.86822 | 0.03231 | -0.07764 | -0.3947 | -0.62746 | -0.70132 | -2.55356 | -2.07271 | 0.03399 | 0.06264 | 0.13313 | 0.16446 | 8 | 2.93229 | 0.55262 | 0.18857 | 0.286219 | Not Significant | NS | #N/A |
| 1772 | P35748 | #N/A | MYH11 | RABBIT | #N/A | 0.43424 | #N/A | #N/A | 0.3101 | #N/A | -0.35955 | -0.30078 | #N/A | #N/A | -0.70234 | -10.2276 | -1.22178 | 0.00942 | 0.27695 | 0.09187 | 0.33229 | 2 | 2.93229 | 0.55262 | 0.00472 | 0.330448 | Not Significant | NS | #N/A |
| 2213 | G1SWD0 | #N/A | PCSK1N | RABBIT | #N/A | #N/A | 0.3054 | #N/A | #N/A | -0.13474 | -1.63476 | 0.57161 | #N/A | #N/A | -0.70469 | -0.54164 | -0.66457 | 0.64234 | 0.53613 | 0.46658 | 0.45752 | 2 | 2.93229 | 0.55262 | 1.26951 | 0.843311 | Not Significant | NS | #N/A |
| 141 | A0A5F9CBZ6 | Secernin 1 OS=Oryctolagus cuniculus OX=9986 GN | SCRN1 | RABBIT | 0.84776 | 1.39366 | -0.40685 | 0.45707 | 0.09529 | -0.5727 | -0.25321 | -0.11948 | 0.05511 | -0.2478 | -0.705 | -2.16703 | -1.90652 | 0.0621 | 0.08319 | 0.15739 | 0.18576 | 8 | 2.93229 | 0.55262 | 0.2646 | 0.341853 | Not Significant | NS | #N/A |
| 59 | A0A5F9DN27 | Heat shock protein 105 kDa OS=Oryctolagus cunicu | HSPH1 | RABBIT | 0.87786 | 0.16027 | -0.0094 | 0.39487 | 0.48581 | -1.01749 | -0.21353 | 0.03841 | 0.03755 | -0.46135 | -0.70517 | -2.83977 | -2.1823 | 0.02182 | 0.0518 | 0.11776 | 0.15166 | 8 | 2.93229 | 0.55262 | 0.15415 | 0.261031 | Not Significant | NS | #N/A |
| 2764 | G1SFX1 | #N/A | C14orf119 | RABBIT | #N/A | #N/A | #N/A | 0.32379 | #N/A | #N/A | -0.38143 | #N/A | #N/A | #N/A | -0.70521 | #N/A | -0.6708 | #N/A | 0.55139 | #N/A | 0.46295 | 0 | 2.93229 | 0.55262 | #N/A | 0.552617 | Not Significant | NS | #N/A |
| 2060 | A0A5F9DAF5 | #N/A | CIROP | RABBIT | #N/A | 0.45304 | #N/A | #N/A | #N/A | -0.25522 | #N/A | #N/A | #N/A | #N/A | -0.70827 | #N/A | -0.67371 | #N/A | 0.54978 | #N/A | 0.46295 | 0 | 2.93229 | 0.55262 | #N/A | 0.552617 | Not Significant | NS | #N/A |
| 274 | A0A5F9DNX0 | Tripartite motif-containing protein 2 OS=Oryctolagu | TRIM2 | RABBIT | 0.55061 | 0.70578 | -0.20542 | 0.84851 | 0.1121 | -1.21163 | -0.11541 | 0.03393 | -0.14373 | -0.0983 | -0.70934 | -2.35909 | -2.00272 | 0.04602 | 0.07063 | 0.1479 | 0.17326 | 8 | 2.93229 | 0.55262 | 0.22603 | 0.313628 | Not Significant | NS | #N/A |
| 2113 | G1U4G0 | #N/A | #N/A | RABBIT | #N/A | 0.47873 | #N/A | #N/A | 0.04715 | #N/A | -0.83807 | #N/A | #N/A | -0.06053 | -0.71224 | -1.60184 | -1.1141 | 0.25035 | 0.31656 | 0.2994 | 0.35506 | 2 | 2.93229 | 0.55262 | 0.19771 | 0.408704 | Not Significant | NS | #N/A |
| 725 | G1SRM8 | SH3 domain binding glutamate rich protein like 2 OS | SH3BGRL2 | RABBIT | 2.84951 | 1.87643 | 0.17065 | -1.15332 | -0.01776 | -1.95333 | 2.21248 | -0.84359 | #N/A | 0.71012 | -0.71368 | -0.62711 | -0.71866 | 0.55048 | 0.48891 | 0.43252 | 0.43948 | 7 | 2.93229 | 0.55262 | 2.87813 | 2.191577 | Not Significant | NS | #N/A |
| 2393 | G1TDJ3 | #N/A | ESYT1 | RABBIT | #N/A | #N/A | 0.44268 | #N/A | #N/A | -0.27203 | #N/A | #N/A | #N/A | #N/A | -0.71471 | #N/A | -0.67984 | #N/A | 0.5464 | #N/A | 0.46295 | 0 | 2.93229 | 0.55262 | #N/A | 0.552617 | Not Significant | NS | #N/A |
| 1321 | G1SSE1 | Diaphanous related formin 2 OS=Oryctolagus cunic | DIAPH2 | RABBIT | #N/A | #N/A | -0.18736 | 0.74499 | 0.15299 | 0.21068 | -0.18248 | -0.06437 | -1.88564 | #N/A | -0.71733 | -1.18153 | -1.20991 | 0.29052 | 0.26113 | 0.3201 | 0.32441 | 5 | 2.93229 | 0.55262 | 0.63186 | 0.602569 | Not Significant | NS | #N/A |
| 425 | A0A5F9C2C6 | CSN8/PSMD8/EIF3K domain-containing protein OS= | #N/A | RABBIT | 1.14838 | -0.04381 | -0.23402 | 0.41738 | 0.40692 | -2.43866 | 0.71844 | -0.03565 | -0.02872 | -0.11603 | -0.71909 | -1.22452 | -1.28811 | 0.25559 | 0.2243 | 0.30172 | 0.29961 | 8 | 2.93229 | 0.55262 | 0.86213 | 0.779114 | Not Significant | NS | #N/A |
| 1175 | A0A5F9D6G0 | Kinesin family member 21B OS=Oryctolagus cunicu | KIF21B | RABBIT | 0.01166 | #N/A | -0.08789 | 0.72336 | 0.47726 | #N/A | #N/A | -0.43837 | #N/A | #N/A | -0.71946 | -1.67494 | -1.09116 | 0.19254 | 0.31753 | 0.26339 | 0.35559 | 3 | 2.93229 | 0.55262 | 0.14761 | 0.3478 | Not Significant | NS | #N/A |
| 1868 | P9WQ63 | #N/A | #N/A | MT | #N/A | 0.59642 | -0.22795 | 0.45525 | #N/A | #N/A | -0.44654 | #N/A | #N/A | #N/A | -0.72111 | -1.41646 | -0.97847 | 0.29233 | 0.37336 | 0.3209 | 0.3868 | 2 | 2.93229 | 0.55262 | 0.19438 | 0.407355 | Not Significant | NS | #N/A |
| 1371 | G1T528 | Transcription activation suppressor OS=Oryctolagu | TASOR | RABBIT | 0.12958 | #N/A | 0.17065 | #N/A | 0.15781 | -0.02778 | -1.11273 | #N/A | #N/A | #N/A | -0.72294 | -1.78662 | -1.29754 | 0.17197 | 0.24261 | 0.2507 | 0.31323 | 3 | 2.93229 | 0.55262 | 0.19648 | 0.372516 | Not Significant | NS | #N/A |
| 1294 | G1SSB5 | DNA-directed RNA polymerases I, II, and III subunit | POLR2H | RABBIT | 0.82863 | 0.20985 | -0.64751 | -0.06967 | 1.25724 | -2.6272 | -0.01616 | 0.27742 | 0.45434 | -0.13431 | -0.72489 | -1.10521 | -1.18518 | 0.3012 | 0.26107 | 0.32455 | 0.32441 | 8 | 2.93229 | 0.55262 | 1.07547 | 0.93523 | Not Significant | NS | #N/A |
| 2709 | G1SD38 | #N/A | STAM2 | RABBIT | #N/A | #N/A | #N/A | 0.18105 | 0.49429 | -0.47518 | #N/A | -0.30306 | #N/A | #N/A | -0.72679 | -4.06688 | -1.24372 | 0.05548 | 0.26944 | 0.15432 | 0.32966 | 2 | 2.93229 | 0.55262 | 0.03194 | 0.341486 | Not Significant | NS | #N/A |
| 407 | G1T2F8 | V-type proton ATPase subunit D OS=Oryctolagus cu | ATP6V1D | RABBIT | 0.7297 | 0.04836 | 0.16057 | -0.29013 | 0.59272 | -2.45518 | 0.01008 | 0.33063 | 0.08748 | -0.37103 | -0.72785 | -1.3492 | -1.39493 | 0.21421 | 0.19073 | 0.27719 | 0.27624 | 8 | 2.93229 | 0.55262 | 0.72756 | 0.680637 | Not Significant | NS | #N/A |
| 627 | A0A5F9CXD1 | Eukaryotic translation initiation factor 4 gamma 2 O | EIF4G2 | RABBIT | 0.5949 | 1.23312 | #N/A | 3.08455 | -2.68407 | 0.41529 | -0.01506 | -0.57786 | -0.6823 | 0 | -0.72911 | -0.67451 | -0.76987 | 0.52163 | 0.45929 | 0.42374 | 0.42475 | 7 | 2.93229 | 0.55262 | 2.59655 | 1.993123 | Not Significant | NS | #N/A |
| 1742 | A0A5F9C333 | #N/A | DDX5 | RABBIT | #N/A | 0.19782 | 1.19086 | -0.11385 | 0.41551 | -1.19789 | -1.7397 | 2.02364 | -2.43301 | 1.80426 | -0.73112 | -0.67622 | -0.77184 | 0.52061 | 0.45818 | 0.42314 | 0.42417 | 7 | 2.93229 | 0.55262 | 2.59773 | 1.993956 | Not Significant | NS | #N/A |
| 2219 | A0A5F9D0E9 | #N/A | SPEN | RABBIT | #N/A | #N/A | 0.17065 | #N/A | 0.77238 | #N/A | -0.31223 | -0.64837 | #N/A | 0.18147 | -0.73122 | -1.90665 | -1.33057 | 0.15263 | 0.23219 | 0.23368 | 0.30343 | 3 | 2.93229 | 0.55262 | 0.1765 | 0.36241 | Not Significant | NS | #N/A |
| 2127 | A0A5F9CXN2 | #N/A | #N/A | RABBIT | #N/A | #N/A | -0.05608 | 1.05956 | 0.21334 | #N/A | #N/A | #N/A | -0.32605 | #N/A | -0.73166 | -1.08846 | -0.92826 | 0.39008 | 0.39643 | 0.36581 | 0.39935 | 2 | 2.93229 | 0.55262 | 0.33888 | 0.46595 | Not Significant | NS | #N/A |
| 1675 | A0A5F9CT56 | #N/A | VAT1L | RABBIT | #N/A | 0.49898 | 0.17065 | -0.31384 | 0.12833 | -0.61184 | #N/A | #N/A | #N/A | #N/A | -0.73287 | -1.96315 | -1.14188 | 0.14441 | 0.29749 | 0.22656 | 0.34543 | 3 | 2.93229 | 0.55262 | 0.11149 | 0.329536 | Not Significant | NS | #N/A |
| 166 | G1SCT1 | Prolyl endopeptidase OS=Oryctolagus cuniculus OX | PREP | RABBIT | 0.76772 | 1.5841 | -0.37802 | 1.14743 | -0.12901 | -0.26814 | -0.40951 | -0.61934 | 0.50713 | 0.11563 | -0.73329 | -1.73151 | -1.67992 | 0.1216 | 0.12129 | 0.20964 | 0.21983 | 8 | 2.93229 | 0.55262 | 0.44838 | 0.476339 | Not Significant | NS | #N/A |
| 66 | G1T926 | Dynactin subunit 1 OS=Oryctolagus cuniculus OX=9 | DCTN1 | RABBIT | 0.67943 | -0.34976 | 0.20083 | -0.08782 | 0.79757 | -2.06713 | 0.12266 | 0.04301 | -0.25724 | -0.27167 | -0.73412 | -1.60027 | -1.58957 | 0.14821 | 0.14041 | 0.22981 | 0.23954 | 8 | 2.93229 | 0.55262 | 0.52613 | 0.533235 | Not Significant | NS | #N/A |
| 1547 | A0A5F9D391 | Large ribosomal subunit protein eL42 OS=Oryctolag | #N/A | RABBIT | #N/A | #N/A | -0.16106 | 0.02407 | 0.57943 | 0 | 0.43617 | #N/A | -0.98846 | -1.79439 | -0.73415 | -1.18186 | -1.21954 | 0.2904 | 0.25766 | 0.3201 | 0.32317 | 5 | 2.93229 | 0.55262 | 0.66148 | 0.621239 | Not Significant | NS | #N/A |
| 2615 | G1SIJ3 | #N/A | AKTIP | RABBIT | #N/A | #N/A | #N/A | 0.33961 | #N/A | #N/A | #N/A | #N/A | #N/A | -0.39725 | -0.73686 | #N/A | -0.70091 | #N/A | 0.5349 | #N/A | 0.45675 | 0 | 2.93229 | 0.55262 | #N/A | 0.552617 | Not Significant | NS | #N/A |
| 611 | P14755 | Lambda-crystallin OS=Oryctolagus cuniculus OX=9 | CRYL1 GU | RABBIT | 0.35617 | 0.79458 | 0.17065 | 0.61811 | -0.1022 | #N/A | -0.8639 | -0.23108 | 0.05117 | -0.43479 | -0.73711 | -2.98348 | -2.16017 | 0.02041 | 0.05627 | 0.11586 | 0.15773 | 7 | 2.93229 | 0.55262 | 0.13565 | 0.258747 | Not Significant | NS | #N/A |
| 1198 | A0A5F9DF95 | Syntaphilin OS=Oryctolagus cuniculus OX=9986 GN | SNPH | RABBIT | 1.12158 | #N/A | 0.26475 | #N/A | 0.26753 | -0.0941 | #N/A | -0.26504 | -0.19955 | #N/A | -0.73752 | -2.54787 | -1.63216 | 0.06345 | 0.14708 | 0.15826 | 0.24654 | 4 | 2.93229 | 0.55262 | 0.12568 | 0.306272 | Not Significant | NS | #N/A |
| 815 | G1T0C5 | Dynactin subunit 6 OS=Oryctolagus cuniculus OX=9 | DCTN6 | RABBIT | 0.99134 | -0.77586 | -0.2654 | -0.0114 | 1.10198 | -2.36805 | 0.49151 | 0.39192 | -0.04624 | -1.15963 | -0.74623 | -1.14098 | -1.22298 | 0.28688 | 0.24705 | 0.31827 | 0.3161 | 8 | 2.93229 | 0.55262 | 1.06938 | 0.93077 | Not Significant | NS | #N/A |
| 2229 | A0A5F9CTJ4 | #N/A | VAMP1 | RABBIT | #N/A | #N/A | 0.27867 | #N/A | -0.0061 | #N/A | #N/A | -0.06437 | -0.70872 | -1.05969 | -0.74721 | -1.91128 | -1.35316 | 0.15193 | 0.22529 | 0.23311 | 0.29961 | 3 | 2.93229 | 0.55262 | 0.18341 | 0.365906 | Not Significant | NS | #N/A |
| 1886 | A5U5S0 | #N/A | #N/A | MT | #N/A | 0.19782 | 0.80208 | -0.56114 | 0.98201 | #N/A | -0.53194 | 0.31405 | #N/A | -0.97077 | -0.75141 | -1.44743 | -1.39765 | 0.20743 | 0.20007 | 0.27264 | 0.28371 | 5 | 2.93229 | 0.55262 | 0.46201 | 0.495503 | Not Significant | NS | #N/A |
| 594 | G1T452 | Rab proteins geranylgeranyltransferase component | CHM | RABBIT | 1.26127 | -0.26953 | -1.21811 | 0.89146 | 1.47228 | -1.18147 | 0.28063 | -1.45462 | 1.01437 | -0.28063 | -0.75182 | -1.0985 | -1.18568 | 0.30394 | 0.26088 | 0.32493 | 0.32441 | 8 | 2.93229 | 0.55262 | 1.17102 | 1.00515 | Not Significant | NS | #N/A |
| 1301 | G1T5Q8 | Protein phosphatase 1 regulatory subunit 8 OS=Ory | PPP1R8 | RABBIT | #N/A | 2.41655 | #N/A | -0.05764 | -0.186 | -0.35727 | #N/A | 0.30199 | #N/A | #N/A | -0.75194 | -0.67099 | -0.80956 | 0.55027 | 0.44944 | 0.43252 | 0.42107 | 3 | 2.93229 | 0.55262 | 1.50703 | 1.035273 | Not Significant | NS | #N/A |
| 2666 | G1T6L3 | #N/A | C3orf38 | RABBIT | #N/A | #N/A | #N/A | 0.3472 | #N/A | -0.40484 | #N/A | #N/A | #N/A | #N/A | -0.75204 | #N/A | -0.71534 | #N/A | 0.52714 | #N/A | 0.45323 | 0 | 2.93229 | 0.55262 | #N/A | 0.552617 | Not Significant | NS | #N/A |
| 393 | P09451 | Ferritin light chain OS=Oryctolagus cuniculus OX=9 | FTL | RABBIT | 0.73544 | 0.98036 | -0.9 | #N/A | 0.17916 | #N/A | #N/A | #N/A | -0.81479 | -0.19255 | -0.75241 | -1.1482 | -1.15675 | 0.31488 | 0.28568 | 0.33147 | 0.33729 | 4 | 2.93229 | 0.55262 | 0.57254 | 0.564115 | Not Significant | NS | #N/A |
| 46 | A0A5F9CNS6 | Ankyrin 2 OS=Oryctolagus cuniculus OX=9986 GN= | ANK2 | RABBIT | 0.91399 | 5.50599 | #N/A | 0.24706 | -0.50654 | #N/A | -0.3047 | 6.65414 | -0.65831 | -2.54435 | -0.75343 | -0.31022 | -0.37425 | 0.76689 | 0.71695 | 0.5042 | 0.5185 | 6 | 2.93229 | 0.55262 | 11.7972 | 8.105815 | Not Significant | NS | #N/A |
| 1861 | G1SUJ6 | #N/A | CHN1 | RABBIT | #N/A | 3.54257 | -0.48698 | #N/A | -0.33364 | 0.30487 | #N/A | #N/A | 0 | #N/A | -0.75488 | -0.44254 | -0.57912 | 0.68806 | 0.58381 | 0.48046 | 0.47236 | 3 | 2.93229 | 0.55262 | 3.49168 | 2.038924 | Not Significant | NS | #N/A |
| 1596 | G1TNZ9 | Complexin-2 OS=Oryctolagus cuniculus OX=9986 ( | CPLX2 | RABBIT | 0.01166 | 0.68761 | 0.10269 | #N/A | 0.14986 | #N/A | #N/A | #N/A | -0.51805 | #N/A | -0.756 | -2.21551 | -1.19486 | 0.11352 | 0.27772 | 0.20022 | 0.3326 | 3 | 2.93229 | 0.55262 | 0.09315 | 0.320262 | Not Significant | NS | #N/A |
| 2188 | G1SEJ8 | #N/A | GNAI1 | RABBIT | #N/A | #N/A | 3.66156 | -0.05764 | #N/A | #N/A | -0.38094 | -0.1329 | 3.64393 | #N/A | -0.7586 | -0.34816 | -0.4679 | 0.75072 | 0.65655 | 0.49986 | 0.4985 | 3 | 2.93229 | 0.55262 | 5.69713 | 3.154233 | Not Significant | NS | #N/A |
| 406 | G1SN21 | Purine nucleoside phosphorylase OS=Oryctolagus ( | PNP | RABBIT | 1.04822 | 2.28232 | #N/A | -0.27332 | -1.51898 | #N/A | 1.08083 | -2.20642 | #N/A | 0 | -0.75976 | -0.60068 | -0.71549 | 0.57424 | 0.49482 | 0.44118 | 0.44101 | 5 | 2.93229 | 0.55262 | 2.74248 | 1.932962 | Not Significant | NS | #N/A |
| 2643 | A0A5F9CUD5 | #N/A | ENOPH1 M | RABBIT | #N/A | #N/A | #N/A | 0.35111 | #N/A | #N/A | #N/A | #N/A | #N/A | -0.40875 | -0.75986 | #N/A | -0.72278 | #N/A | 0.52317 | #N/A | 0.45286 | 0 | 2.93229 | 0.55262 | #N/A | 0.552617 | Not Significant | NS | #N/A |
| 2503 | G1TUX8 | #N/A | PAK3 | RABBIT | #N/A | #N/A | #N/A | -0.03564 | #N/A | 0 | -1.59797 | #N/A | #N/A | #N/A | -0.76335 | -0.5516 | -0.72612 | 0.6791 | 0.50862 | 0.47768 | 0.44813 | 1 | 2.93229 | 0.55262 | 1.27676 | 0.73677 | Not Significant | NS | #N/A |
| 1061 | G1SGS0 | Peptidase M60 domain-containing protein OS=Oryc | #N/A | RABBIT | 3.98411 | #N/A | #N/A | 3.50845 | #N/A | -3.56609 | #N/A | #N/A | -5.22865 | #N/A | -8.14365 | -9.4186 | -10.2464 | 0.01109 | 0.00016 | 0.09732 | 0.0188 | 2 | 2.93229 | 0.55262 | 0.74759 | 0.631678 | Significant | Down mtub | #N/A |
| 418 | A0A5F9DQI5 | Abl interactor 2 OS=Oryctolagus cuniculus OX=998 | #N/A | RABBIT | 1.84604 | 0.29191 | -0.6714 | 0.15395 | -0.10747 | -2.31428 | -1.09837 | 0.10978 | 1.32051 | -0.33137 | -0.76535 | -1.03775 | -1.13175 | 0.32973 | 0.28195 | 0.33706 | 0.33531 | 8 | 2.93229 | 0.55262 | 1.35981 | 1.143301 | Not Significant | NS | #N/A |
| 2408 | G1SZG3 | #N/A | SLC30A7 | RABBIT | #N/A | #N/A | 0.46826 | #N/A | #N/A | -0.29761 | #N/A | #N/A | #N/A | #N/A | -0.76587 | #N/A | -0.72849 | #N/A | 0.52014 | #N/A | 0.452 | 0 | 2.93229 | 0.55262 | #N/A | 0.552617 | Not Significant | NS | #N/A |
| 1192 | G1TIK7 | Sorting nexin OS=Oryctolagus cuniculus OX=9986 ( | SNX18 | RABBIT | 1.06618 | 0.33931 | #N/A | -0.19913 | #N/A | #N/A | -0.3638 | #N/A | #N/A | #N/A | -0.76592 | -1.0446 | -0.94562 | 0.40586 | 0.38833 | 0.37324 | 0.39443 | 2 | 2.93229 | 0.55262 | 0.40321 | 0.492033 | Not Significant | NS | #N/A |
| 888 | G1TIL8 | Potassium channel tetramerization domain contain | KCTD8 | RABBIT | -0.87655 | 0.21649 | 0.02246 | 0.14471 | 0.36615 | 0 | #N/A | -1.42178 | 0.09348 | -1.84555 | -0.76811 | -1.53691 | -1.53791 | 0.1682 | 0.1553 | 0.24693 | 0.25326 | 7 | 2.93229 | 0.55262 | 0.55506 | 0.55434 | Not Significant | NS | #N/A |
| 2364 | G1T3X4 | #N/A | EPC2 | RABBIT | #N/A | #N/A | 0.47078 | #N/A | #N/A | #N/A | -0.30013 | #N/A | #N/A | #N/A | -0.77091 | #N/A | -0.73329 | #N/A | 0.51761 | #N/A | 0.45117 | 0 | 2.93229 | 0.55262 | #N/A | 0.552617 | Not Significant | NS | #N/A |
| 2357 | G1SHP4 | #N/A | #N/A | RABBIT | #N/A | #N/A | 0.65607 | #N/A | #N/A | #N/A | -0.28088 | -0.11934 | 0.05498 | #N/A | -0.77116 | -3.97591 | -1.14538 | 0.05783 | 0.30457 | 0.15468 | 0.3501 | 2 | 2.93229 | 0.55262 | 0.02821 | 0.339976 | Not Significant | NS | #N/A |
| 2825 | Q9X5M0 | #N/A | #N/A | MT | #N/A | #N/A | #N/A | #N/A | 0.38294 | #N/A | #N/A | #N/A | #N/A | -0.39632 | -0.77927 | #N/A | -0.74124 | #N/A | 0.51343 | #N/A | 0.44921 | 0 | 2.93229 | 0.55262 | #N/A | 0.552617 | Not Significant | NS | #N/A |
| 1322 | A0A5F9CME7 | Crystallin beta-gamma domain containing 1 OS=Or | CRYBG1 | RABBIT | 0.46921 | #N/A | 0.54072 | #N/A | -0.38346 | #N/A | 0.74768 | #N/A | -0.86307 | -1.6034 | -0.78175 | -1.03563 | -1.12297 | 0.35887 | 0.29883 | 0.35078 | 0.34595 | 4 | 2.93229 | 0.55262 | 0.8547 | 0.726925 | Not Significant | NS | #N/A |
| 174 | G1SU71 | Proteasome subunit beta OS=Oryctolagus cuniculu | PSMB1 | RABBIT | 0.51199 | 0.0809 | 0.49185 | -0.05764 | 0.7409 | #N/A | 0.81308 | -0.62457 | -1.5412 | -0.37085 | -0.78448 | -1.71388 | -1.66846 | 0.13027 | 0.12639 | 0.21499 | 0.2257 | 7 | 2.93229 | 0.55262 | 0.46558 | 0.491275 | Not Significant | NS | #N/A |
| 2421 | G1SHW7 | #N/A | HES7 | RABBIT | #N/A | #N/A | 0.47764 | #N/A | #N/A | -0.30699 | #N/A | #N/A | #N/A | #N/A | -0.78462 | #N/A | -0.74633 | #N/A | 0.51077 | #N/A | 0.44921 | 0 | 2.93229 | 0.55262 | #N/A | 0.552617 | Not Significant | NS | #N/A |
| 486 | G1SCT8 | RAP1 GTPase activating protein 2 OS=Oryctolagus c | RAP1GAP2 | RABBIT | 1.24608 | 0.48724 | -0.11877 | 0.83568 | -0.9403 | #N/A | -0.81916 | -1.04925 | 0.41357 | #N/A | -0.78693 | -1.29281 | -1.33853 | 0.24362 | 0.21379 | 0.29685 | 0.29197 | 6 | 2.93229 | 0.55262 | 0.69471 | 0.648062 | Not Significant | NS | #N/A |
| 1662 | G1U7V8 | #N/A | #N/A | RABBIT | #N/A | #N/A | 0.49474 | #N/A | #N/A | -1.4553 | 0.24956 | 0.34803 | -0.24956 | -0.35701 | -0.78759 | -1.00132 | -0.98643 | 0.37333 | 0.35709 | 0.35712 | 0.3777 | 4 | 2.93229 | 0.55262 | 0.51555 | 0.531231 | Not Significant | NS | #N/A |
| 1971 | G1SGG7 | #N/A | KCTD3 | RABBIT | #N/A | 0.94664 | #N/A | -0.08265 | 0.827 | #N/A | -0.05726 | -0.03936 | -0.58223 | #N/A | -0.78995 | -2.13169 | -1.62929 | 0.10002 | 0.14769 | 0.1899 | 0.24729 | 4 | 2.93229 | 0.55262 | 0.20599 | 0.352607 | Not Significant | NS | #N/A |
| 2594 | G1SUT8 | #N/A | SEC24A | RABBIT | #N/A | #N/A | #N/A | 0.61465 | #N/A | #N/A | #N/A | -0.27559 | -0.4761 | 0.21123 | -0.79481 | -1.94738 | -1.11779 | 0.19086 | 0.31512 | 0.26255 | 0.35406 | 2 | 2.93229 | 0.55262 | 0.12493 | 0.379195 | Not Significant | NS | #N/A |
| 2214 | A0A5F9CRK2 | #N/A | RMND5A | RABBIT | #N/A | #N/A | -0.21392 | 0.25118 | -0.01338 | #N/A | -2.77456 | 0.02668 | 0.1271 | -0.52925 | -0.79547 | -0.98209 | -1.08988 | 0.37116 | 0.30776 | 0.3561 | 0.35174 | 5 | 2.93229 | 0.55262 | 1.12467 | 0.913201 | Not Significant | NS | #N/A |
| 2724 | G1U2Y0 | #N/A | NFKB1 | RABBIT | #N/A | #N/A | #N/A | 0.55405 | -0.01338 | #N/A | #N/A | #N/A | -7.17909 | #N/A | -7.44942 | -15.1592 | -9.03683 | 0.04193 | 0.0009 | 0.14462 | 0.036 | 1 | 2.93229 | 0.55262 | 0.16099 | 0.453024 | Significant | Down mtub | NFKB1 |
| 2130 | A0A5F9CAM0 | #N/A | XPO7 | RABBIT | #N/A | #N/A | 1.05869 | #N/A | #N/A | 1.23134 | -0.10982 | -0.06437 | #N/A | -0.01202 | -0.79741 | -1.10076 | -1.02364 | 0.3514 | 0.34592 | 0.34717 | 0.37092 | 3 | 2.93229 | 0.55262 | 0.41982 | 0.485462 | Not Significant | NS | #N/A |
| 1760 | A0A5F9C4V3 | #N/A | GIT1 | RABBIT | #N/A | -0.31261 | #N/A | #N/A | 0.45312 | 0.2729 | 0.24594 | -0.06437 | -1.05931 | -3.03233 | -0.79769 | -0.74891 | -0.86111 | 0.48763 | 0.41445 | 0.4093 | 0.40833 | 5 | 2.93229 | 0.55262 | 1.62073 | 1.225887 | Not Significant | NS | #N/A |
| 1208 | G1SK10 | Glycerophosphodiester phosphodiesterase 1 OS=C | GDE1 | RABBIT | 0.94381 | #N/A | 0.17065 | -0.23323 | 1.41521 | -1.70481 | 1.02283 | 0.01952 | -0.06345 | -0.41955 | -0.8032 | -1.3472 | -1.41123 | 0.21989 | 0.18872 | 0.28229 | 0.27623 | 7 | 2.93229 | 0.55262 | 0.78989 | 0.719843 | Not Significant | NS | #N/A |
| 51 | B6V9S8 | T-complex protein 1 subunit eta OS=Oryctolagus cu | #N/A | RABBIT | 1.04449 | 0.1148 | 0.16302 | -0.05001 | 0.77864 | -1.60942 | -0.02736 | 0.03082 | -0.51529 | 0.15287 | -0.80386 | -2.07414 | -1.95421 | 0.07177 | 0.07673 | 0.16449 | 0.1812 | 8 | 2.93229 | 0.55262 | 0.37552 | 0.423019 | Not Significant | NS | #N/A |
| 787 | A0A5F9CC63 | Phosphatidylinositol binding clathrin assembly prot | PICALM | RABBIT | 0.33631 | 0.19782 | 0.29205 | -0.36025 | 0.35759 | #N/A | -0.69838 | 1.07006 | -0.95046 | -1.98158 | -0.80479 | -1.39417 | -1.44952 | 0.20592 | 0.17802 | 0.27238 | 0.26845 | 7 | 2.93229 | 0.55262 | 0.7405 | 0.68503 | Not Significant | NS | #N/A |
| 712 | G1SP52 | Neuroligin 2 OS=Oryctolagus cuniculus OX=9986 G | NLGN2 | RABBIT | 1.01776 | #N/A | -0.96343 | 1.97716 | -0.01338 | -1.36085 | 0.45679 | 0.61963 | -1.03631 | -0.18187 | -0.80505 | -1.12575 | -1.22225 | 0.29738 | 0.24983 | 0.32281 | 0.31705 | 7 | 2.93229 | 0.55262 | 1.13645 | 0.964084 | Not Significant | NS | #N/A |
| 551 | G1U6W5 | SRC kinase signaling inhibitor 1 OS=Oryctolagus cu | SRCIN1 | RABBIT | 1.10849 | -0.5507 | -0.35997 | -0.06454 | 1.07593 | -2.48697 | -0.75751 | -0.00773 | 0.41923 | 0.0069 | -0.80706 | -1.2867 | -1.3697 | 0.23418 | 0.19826 | 0.29142 | 0.28262 | 8 | 2.93229 | 0.55262 | 0.98354 | 0.867953 | Not Significant | NS | #N/A |
| 179 | G1T4Q9 | Proteasome subunit beta OS=Oryctolagus cuniculu | PSMB5 | RABBIT | 0.53698 | 0.18398 | 0.17065 | 0.09171 | 1.02636 | #N/A | 0.24165 | -0.49038 | -1.12497 | -0.25495 | -0.8091 | -2.54544 | -2.12756 | 0.03836 | 0.05945 | 0.1391 | 0.16179 | 7 | 2.93229 | 0.55262 | 0.22453 | 0.321387 | Not Significant | NS | #N/A |
| 2494 | A0A5F9CJ53 | #N/A | RUFY3 | RABBIT | #N/A | #N/A | #N/A | 0.65517 | #N/A | #N/A | -0.63423 | 0.40913 | 0 | -0.3915 | -0.80932 | -1.58234 | -1.17585 | 0.21172 | 0.28468 | 0.27513 | 0.33729 | 3 | 2.93229 | 0.55262 | 0.20928 | 0.378991 | Not Significant | NS | #N/A |
| 67 | G1SCN8 | T-complex protein 1 subunit gamma OS=Oryctolagu | CCT3 | RABBIT | 0.88925 | 0.02284 | 0.28806 | -0.19527 | 0.61091 | -2.00568 | 0.00958 | -0.02387 | -0.40599 | -0.00958 | -0.81027 | -1.8674 | -1.82527 | 0.0988 | 0.09538 | 0.18811 | 0.19678 | 8 | 2.93229 | 0.55262 | 0.47068 | 0.492654 | Not Significant | NS | #N/A |
| 2333 | A0A5F9D0S9 | #N/A | GCDH | RABBIT | #N/A | #N/A | 0.7646 | #N/A | #N/A | -0.32581 | #N/A | 1.03779 | -0.8961 | 0 | -0.81063 | -0.893 | -0.93098 | 0.43765 | 0.38818 | 0.3855 | 0.39443 | 3 | 2.93229 | 0.55262 | 0.65923 | 0.60653 | Not Significant | NS | #N/A |
| 276 | G1T5J8 | Dynactin subunit 4 OS=Oryctolagus cuniculus OX=9 | DCTN4 | RABBIT | 1.2162 | 0.06562 | 0.63566 | -0.35781 | 0.64447 | -2.5917 | 1.35481 | 0.06783 | -0.19095 | -0.50093 | -0.81302 | -1.17282 | -1.26826 | 0.27461 | 0.23105 | 0.31101 | 0.30329 | 8 | 2.93229 | 0.55262 | 1.20138 | 1.027368 | Not Significant | NS | #N/A |
| 60 | G1SHZ8 | T-complex protein 1 subunit theta OS=Oryctolagus ( | CCT8 | RABBIT | 0.64719 | -0.31717 | 0.26714 | -0.05853 | 0.83563 | -2.29278 | 0.08586 | -0.06347 | -0.34199 | -0.08752 | -0.81483 | -1.65472 | -1.67469 | 0.13658 | 0.12233 | 0.21984 | 0.22013 | 8 | 2.93229 | 0.55262 | 0.60622 | 0.591842 | Not Significant | NS | #N/A |
| 1586 | A0A5F9DAX6 | Uncharacterized protein OS=Oryctolagus cuniculus | #N/A | RABBIT | 0.5195 | #N/A | #N/A | 0.60263 | 0.67167 | #N/A | -0.6575 | -0.10825 | 0 | -0.11488 | -0.81809 | -4.56772 | -2.19126 | 0.00601 | 0.06009 | 0.07337 | 0.16242 | 5 | 2.93229 | 0.55262 | 0.05499 | 0.238945 | Not Significant | NS | #N/A |
| 2469 | G1SVI4 | #N/A | #N/A | RABBIT | #N/A | #N/A | 0.43797 | #N/A | -0.01338 | #N/A | -1.38786 | -0.04388 | #N/A | -0.39054 | -0.81972 | -1.49969 | -1.33201 | 0.23066 | 0.23174 | 0.2895 | 0.30329 | 3 | 2.93229 | 0.55262 | 0.35852 | 0.454461 | Not Significant | NS | #N/A |
| 2271 | G1SW47 | #N/A | #N/A | RABBIT | #N/A | #N/A | 1.75061 | #N/A | -0.19845 | #N/A | #N/A | -0.27741 | 0.18507 | #N/A | -0.82225 | -0.82095 | -0.95889 | 0.49796 | 0.38223 | 0.41323 | 0.39172 | 2 | 2.93229 | 0.55262 | 1.00319 | 0.735319 | Not Significant | NS | #N/A |
| 76 | B2ZDY6 | T-complex protein 1 subunit alpha OS=Oryctolagus | #N/A | RABBIT | 0.50929 | -0.18132 | 0.2702 | 0.05655 | 0.8403 | -2.02644 | -0.09955 | 0.04176 | -0.29757 | -0.26325 | -0.82802 | -1.97814 | -1.91219 | 0.08329 | 0.0824 | 0.17453 | 0.18516 | 8 | 2.93229 | 0.55262 | 0.43803 | 0.468766 | Not Significant | NS | #N/A |
| 580 | G1TEQ9 | Glucosamine-6-phosphate isomerase OS=Oryctola | GNPDA2 | RABBIT | 0.30874 | #N/A | #N/A | -0.35471 | 0.88407 | #N/A | -0.55035 | #N/A | #N/A | #N/A | -0.82971 | -1.15912 | -1.03246 | 0.3661 | 0.3498 | 0.35413 | 0.37391 | 2 | 2.93229 | 0.55262 | 0.38429 | 0.484362 | Not Significant | NS | #N/A |
| 367 | A0A5F9CHM5 | Disks large homolog 1 OS=Oryctolagus cuniculus C | DLG1 | RABBIT | 0.82366 | 1.3557 | -0.73178 | 1.01611 | 0.06649 | -0.80406 | -0.07987 | 0.92604 | -1.14216 | -0.52349 | -0.83074 | -1.60319 | -1.64261 | 0.14756 | 0.12889 | 0.2295 | 0.22709 | 8 | 2.93229 | 0.55262 | 0.67128 | 0.639449 | Not Significant | NS | #N/A |
| 720 | G1T5I9 | Cleavage and polyadenylation specificity factor sub | CPSF6 | RABBIT | 0.04638 | -0.39648 | -0.8495 | 1.16487 | 1.99233 | #N/A | -1.13997 | 0.19259 | -0.81514 | 0 | -0.83215 | -1.27126 | -1.35817 | 0.24425 | 0.20446 | 0.29685 | 0.28585 | 7 | 2.93229 | 0.55262 | 0.95219 | 0.834224 | Not Significant | NS | #N/A |
| 1924 | G1TS24 | #N/A | FAT3 | RABBIT | #N/A | 1.36199 | 0.17065 | #N/A | #N/A | -0.06742 | #N/A | #N/A | #N/A | #N/A | -0.83374 | -0.8081 | -0.88434 | 0.56732 | 0.42727 | 0.43867 | 0.41011 | 1 | 2.93229 | 0.55262 | 0.70964 | 0.59255 | Not Significant | NS | #N/A |
| 154 | G1TY77 | non-specific serine/threonine protein kinase OS=Or | ROCK2 | RABBIT | 0.40418 | -1.84092 | 3.12508 | -1.13028 | 0.82921 | -3.12554 | -0.30013 | 0.63259 | 0.28153 | -0.28153 | -0.83607 | -0.76713 | -0.86766 | 0.46504 | 0.40422 | 0.39881 | 0.40317 | 8 | 2.93229 | 0.55262 | 2.96952 | 2.321251 | Not Significant | NS | #N/A |
| 2510 | G1SSX5 | #N/A | MARS1 | RABBIT | #N/A | #N/A | #N/A | 0.00719 | #N/A | 0.19334 | #N/A | -2.62813 | #N/A | -0.06483 | -0.84039 | -0.4666 | -0.63465 | 0.68668 | 0.55392 | 0.48039 | 0.46296 | 2 | 2.93229 | 0.55262 | 2.43297 | 1.315086 | Not Significant | NS | #N/A |
| 2570 | G1SKE2 | #N/A | TIAL1 | RABBIT | #N/A | #N/A | #N/A | 0.43133 | #N/A | -0.81816 | #N/A | #N/A | #N/A | 0 | -0.84041 | -1.1861 | -0.97315 | 0.44594 | 0.38647 | 0.38942 | 0.39349 | 1 | 2.93229 | 0.55262 | 0.33469 | 0.497197 | Not Significant | NS | #N/A |
| 1585 | G1TDA6 | TAL bHLH transcription factor 1, erythroid differenti | TAL1 | RABBIT | -0.26097 | -1.17634 | 0.44328 | #N/A | 0.90896 | -2.1411 | 0.41579 | #N/A | #N/A | #N/A | -0.84139 | -0.81092 | -0.94277 | 0.4629 | 0.37749 | 0.39768 | 0.38895 | 4 | 2.93229 | 0.55262 | 1.43541 | 1.061995 | Not Significant | NS | #N/A |
| 834 | G1SH63 | Glutathione synthetase OS=Oryctolagus cuniculus | GSS | RABBIT | 1.12799 | 2.33993 | -0.77903 | 1.45998 | -0.38558 | #N/A | 0.03869 | #N/A | -0.03869 | -0.26952 | -0.8425 | -1.07941 | -1.18442 | 0.32188 | 0.26681 | 0.33441 | 0.32866 | 6 | 2.93229 | 0.55262 | 1.14228 | 0.948704 | Not Significant | NS | #N/A |
| 628 | A0A5F9DVB3 | Epidermal growth factor receptor pathway substrat | EPS15L1 | RABBIT | -0.23019 | -0.5883 | -0.00444 | 0.84402 | 0.40752 | -4.48314 | 0.25811 | 0.41087 | 0.14527 | -0.14527 | -0.84856 | -0.87738 | -0.98392 | 0.40586 | 0.34643 | 0.37324 | 0.37123 | 8 | 2.93229 | 0.55262 | 2.33843 | 1.859436 | Not Significant | NS | #N/A |
| 64 | B7NZE7 | Microtubule-associated protein 1A (Predicted) OS= | MAP1A | RABBIT | 0.43028 | #N/A | #N/A | #N/A | #N/A | #N/A | -0.41862 | #N/A | #N/A | #N/A | -0.8489 | #N/A | -0.80748 | #N/A | 0.47972 | #N/A | 0.43381 | 0 | 2.93229 | 0.55262 | #N/A | 0.552617 | Not Significant | NS | #N/A |
| 2459 | A5U4B4 | #N/A | #N/A | MT | #N/A | #N/A | #N/A | #N/A | 0.60394 | #N/A | #N/A | -0.49155 | #N/A | 0 | -0.84972 | -1.99605 | -1.04261 | 0.29567 | 0.35694 | 0.32166 | 0.3777 | 1 | 2.93229 | 0.55262 | 0.12081 | 0.442807 | Not Significant | NS | #N/A |

| | | | | | | | | | | | | | | | | | | | | | | | | | | | | | |
|---|---|---|---|---|---|---|---|---|---|---|---|---|---|---|---|---|---|---|---|---|---|---|---|---|---|---|---|---|---|
| 2849 | A0A5F9DSA9 | #N/A | ARHGEF4( | RABBIT | #N/A | #N/A | #N/A | #N/A | 0.38637 | #N/A | #N/A | -0.46412 | #N/A | #N/A | -0.85049 | #N/A | -0.80899 | #N/A | 0.47897 | #N/A | 0.43349 | 0 | 2.93229 | 0.55262 | #N/A | 0.552617 | Not Significant | NS | #N/A |
| 315 | G1TNI4 | Glutathione S-transferase OS=Oryctolagus cunicul | GSTM2 | RABBIT | 0.8882 | 2.33465 | -0.52769 | 2.16147 | -0.62989 | 0.30974 | -0.18266 | #N/A | 0 | -0.15403 | -0.85209 | -1.17601 | -1.27962 | 0.27803 | 0.22976 | 0.3132 | 0.30233 | 7 | 2.93229 | 0.55262 | 1.16663 | 0.985358 | Not Significant | NS | #N/A |
| 801 | G1SFA4 | BMP/retinoic acid-inducible neural-specific protein | BRINP1 | RABBIT | 0.20881 | #N/A | 0.17065 | 0.3642 | -0.31419 | #N/A | -0.74476 | #N/A | #N/A | #N/A | -0.85213 | -2.59912 | -1.35446 | 0.08044 | 0.2249 | 0.1731 | 0.29961 | 3 | 2.93229 | 0.55262 | 0.08599 | 0.31664 | Not Significant | NS | #N/A |
| 1351 | G1T443 | Chromosome 11 open reading frame 54 OS=Orycto | C11orf54 | RABBIT | #N/A | 1.46184 | -0.642 | 0.94457 | 0.59002 | -0.26196 | -0.53144 | -0.06437 | 0.35664 | -0.82367 | -0.85357 | -1.878 | -1.82393 | 0.10247 | 0.09835 | 0.19228 | 0.19787 | 7 | 2.93229 | 0.55262 | 0.45906 | 0.486682 | Not Significant | NS | #N/A |
| 865 | Q8WN94 | Acyl-CoA-binding protein OS=Oryctolagus cuniculu | DBI | RABBIT | -0.01738 | 3.0346 | 0.47791 | -0.17985 | 0.01566 | -0.83218 | 0.63698 | -0.76837 | -1.08555 | 1.10413 | -0.85519 | -1.14491 | -1.25065 | 0.28534 | 0.23717 | 0.31704 | 0.30736 | 8 | 2.93229 | 0.55262 | 1.39483 | 1.16893 | Not Significant | NS | #N/A |
| 1806 | A0A5F9C2L8 | #N/A | LUC7L2 | RABBIT | #N/A | -0.34962 | 0.55069 | -0.05764 | 1.67861 | -0.99566 | 0.20952 | #N/A | #N/A | -0.4167 | -0.85645 | -1.41431 | -1.44702 | 0.21641 | 0.18623 | 0.27929 | 0.27501 | 5 | 2.93229 | 0.55262 | 0.62864 | 0.600536 | Not Significant | NS | #N/A |
| 1661 | B6V9S9 | #N/A | CCT2 | RABBIT | #N/A | -0.14805 | 0.4665 | 0.23427 | 0.94581 | -1.90013 | -0.0338 | 0.06313 | -0.54244 | 0 | -0.85728 | -1.84109 | -1.80241 | 0.10817 | 0.10186 | 0.1968 | 0.20137 | 7 | 2.93229 | 0.55262 | 0.48182 | 0.502718 | Not Significant | NS | #N/A |
| 2059 | G1TAM2 | #N/A | DPY19L1 | RABBIT | #N/A | 0.19782 | #N/A | 0.99797 | #N/A | #N/A | -0.26011 | #N/A | #N/A | #N/A | -0.85801 | -1.2382 | -0.99725 | 0.4325 | 0.37598 | 0.38318 | 0.3881 | 1 | 2.93229 | 0.55262 | 0.32012 | 0.493491 | Not Significant | NS | #N/A |
| 292 | G1T1D9 | COP9 signalosome subunit 5 OS=Oryctolagus cuni | COPS5 | RABBIT | 0.86874 | 0.11026 | -0.21785 | 0.23652 | 0.71071 | -2.16983 | 0.2941 | 0.0232 | -0.28612 | -0.45745 | -0.8609 | -1.8089 | -1.81477 | 0.10807 | 0.09706 | 0.1968 | 0.19688 | 8 | 2.93229 | 0.55262 | 0.56626 | 0.562597 | Not Significant | NS | #N/A |
| 1415 | A0A5F9CSX9 | Sortilin-related receptor OS=Oryctolagus cuniculus | SORL1 | RABBIT | 3.06587 | #N/A | #N/A | #N/A | #N/A | #N/A | #N/A | #N/A | -3.05421 | #N/A | -6.12008 | #N/A | -5.82143 | #N/A | 0.01075 | #N/A | 0.09154 | 0 | 2.93229 | 0.55262 | #N/A | 0.552617 | Significant | Down mtub | SORL1 |
| 571 | P21195 | Protein disulfide-isomerase OS=Oryctolagus cunicu | P4HB PDIA | RABBIT | 2.46872 | 2.06906 | -1.35052 | -0.02368 | -1.33055 | -2.69022 | 1.00531 | 0.31553 | -1.0816 | -0.03396 | -0.86359 | -0.83134 | -0.93728 | 0.42989 | 0.36886 | 0.38166 | 0.38394 | 8 | 2.93229 | 0.55262 | 2.69774 | 2.122371 | Not Significant | NS | #N/A |
| 1502 | A0A5F9DWB8 | K Homology domain-containing protein OS=Oryctol | #N/A | RABBIT | #N/A | 0.04977 | #N/A | 0.09042 | 0.67365 | #N/A | 1.37963 | -1.8905 | #N/A | -1.27259 | -0.86576 | -0.84622 | -0.99323 | 0.44508 | 0.35399 | 0.38942 | 0.37605 | 4 | 2.93229 | 0.55262 | 1.57008 | 1.139702 | Not Significant | NS | #N/A |
| 2590 | G1SR38 | #N/A | #N/A | RABBIT | #N/A | #N/A | #N/A | -0.25694 | 1.40164 | #N/A | #N/A | 0.13494 | #N/A | -0.72406 | -0.86691 | -0.92825 | -1.04958 | 0.45127 | 0.34259 | 0.39206 | 0.3692 | 2 | 2.93229 | 0.55262 | 0.87219 | 0.682201 | Not Significant | NS | #N/A |
| 285 | G1SWW8 | Serine/threonine-protein phosphatase OS=Oryctola | PPP1CC | RABBIT | 3.31627 | -0.0662 | -0.90919 | -0.07601 | 3.25097 | 0.94576 | -0.35274 | 0.72491 | -0.15559 | 0.01837 | -0.86703 | -0.92403 | -1.03367 | 0.3825 | 0.32362 | 0.36153 | 0.35728 | 8 | 2.93229 | 0.55262 | 2.20105 | 1.758905 | Not Significant | NS | #N/A |
| 1656 | A0A5F9DRD6 | #N/A | ACTN1 | RABBIT | #N/A | 0.19782 | #N/A | 0.43469 | #N/A | #N/A | -5.39115 | #N/A | #N/A | #N/A | -5.70741 | -27.8225 | -7.19736 | 0.02287 | 0.00211 | 0.11776 | 0.05019 | 1 | 2.93229 | 0.55262 | 0.02805 | 0.419218 | Significant | Down mtub | ACTN1 |
| 2385 | G1T861 | #N/A | BLTP1 | RABBIT | #N/A | #N/A | 0.59418 | #N/A | #N/A | #N/A | #N/A | -0.3349 | -0.76848 | 0.27054 | -0.87179 | -1.4467 | -1.13953 | 0.28491 | 0.30678 | 0.31704 | 0.3516 | 2 | 2.93229 | 0.55262 | 0.27235 | 0.438971 | Not Significant | NS | #N/A |
| 1103 | A0A5F9CQP0 | superoxide dismutase OS=Oryctolagus cuniculus C | #N/A | RABBIT | 0.22148 | 0.77271 | #N/A | #N/A | #N/A | #N/A | -0.20982 | -0.54047 | #N/A | #N/A | -0.87224 | -2.7139 | -1.43314 | 0.11318 | 0.21203 | 0.20012 | 0.29139 | 2 | 2.93229 | 0.55262 | 0.1033 | 0.370421 | Not Significant | NS | #N/A |
| 1182 | G1SLI7 | FYVE and coiled-coil domain containing 1 OS=Oryct | FYCO1 | RABBIT | #N/A | #N/A | #N/A | -0.05764 | #N/A | #N/A | #N/A | -2.06498 | 0.19987 | #N/A | -0.87492 | -0.44606 | -0.69245 | 0.73289 | 0.52737 | 0.49265 | 0.45323 | 1 | 2.93229 | 0.55262 | 2.56478 | 1.064319 | Not Significant | NS | #N/A |
| 123 | P00939 | Triosephosphate isomerase OS=Oryctolagus cunic | TPI1 | RABBIT | 1.07553 | 2.17988 | 0.13143 | 0.73391 | -0.23087 | 0.10065 | 0.03922 | -0.39522 | -0.10005 | -0.13473 | -0.876 | -2.0535 | -1.99681 | 0.0741 | 0.07135 | 0.16701 | 0.17448 | 8 | 2.93229 | 0.55262 | 0.45495 | 0.481145 | Not Significant | NS | #N/A |
| 2836 | G1SFG8 | #N/A | DOCK1 | RABBIT | #N/A | #N/A | #N/A | #N/A | 0.56949 | 1.36124 | 0.07616 | -1.34684 | -1.55707 | -0.07616 | -0.87803 | -0.67578 | -0.7839 | 0.53623 | 0.45904 | 0.42838 | 0.42474 | 4 | 2.93229 | 0.55262 | 1.40676 | 1.045465 | Not Significant | NS | #N/A |
| 1186 | A0A5F9DMV9 | RNA helicase OS=Oryctolagus cuniculus OX=9986 | DDX4 | RABBIT | 1.32344 | #N/A | #N/A | 0.16783 | #N/A | #N/A | 0 | #N/A | -0.07893 | -0.31947 | -0.87843 | -1.96002 | -1.531 | 0.14485 | 0.17721 | 0.22701 | 0.26787 | 3 | 2.93229 | 0.55262 | 0.24103 | 0.395046 | Not Significant | NS | #N/A |
| 424 | G1TUE0 | Fumarate hydratase, mitochondrial OS=Oryctolagu | #N/A | RABBIT | 0.90624 | 1.60946 | -0.4987 | 0.60785 | -0.14642 | -1.5069 | -0.07362 | 0.00925 | -0.51815 | 0.17283 | -0.879 | -1.82049 | -1.83328 | 0.10617 | 0.09411 | 0.19552 | 0.19607 | 8 | 2.93229 | 0.55262 | 0.58283 | 0.574729 | Not Significant | NS | #N/A |
| 1479 | G1T0N3 | C2 domain-containing protein OS=Oryctolagus cun | CC2D2B | RABBIT | -1.00187 | 1.78703 | -0.04341 | 0.15642 | #N/A | 0.79645 | -2.1063 | #N/A | #N/A | #N/A | -0.87947 | -0.70777 | -0.85171 | 0.51815 | 0.42282 | 0.42184 | 0.40913 | 4 | 2.93229 | 0.55262 | 2.05871 | 1.421649 | Not Significant | NS | #N/A |
| 2693 | A0A5F9CM50 | #N/A | MAN2A2 | RABBIT | #N/A | #N/A | #N/A | 5.565 | #N/A | #N/A | #N/A | #N/A | 0 | -0.14674 | -5.63837 | -44.3695 | -7.14788 | 0.01435 | 0.00216 | 0.09894 | 0.05019 | 1 | 2.93229 | 0.55262 | 0.01077 | 0.414821 | Significant | Down mtub | MAN2A2 |
| 110 | P62160 | Calmodulin OS=Oryctolagus cuniculus OX=9986 G | CALM CAM | RABBIT | 0.60155 | 1.15647 | 0.01063 | 0.12325 | -0.1053 | -2.1948 | 0.07521 | -0.10026 | -0.43547 | 0.0359 | -0.8812 | -1.81033 | -1.82684 | 0.10784 | 0.09513 | 0.1968 | 0.19659 | 8 | 2.93229 | 0.55262 | 0.59235 | 0.581693 | Not Significant | NS | #N/A |
| 2025 | A0A5F9CXV4 | #N/A | RTCA | RABBIT | #N/A | #N/A | 0.99402 | -0.05764 | #N/A | #N/A | -0.41319 | #N/A | #N/A | #N/A | -0.88138 | -0.96774 | -0.96798 | 0.51044 | 0.38875 | 0.42044 | 0.39446 | 1 | 2.93229 | 0.55262 | 0.55299 | 0.552712 | Not Significant | NS | #N/A |
| 202 | G1T8P1 | 10-formyltetrahydrofolate dehydrogenase OS=Oryc | ALDH1L1 | RABBIT | -0.1882 | -0.27608 | 1.13878 | 1.42873 | 1.00533 | -1.53979 | 0.19986 | 0.86582 | -0.30884 | -0.51562 | -0.88143 | -1.64928 | -1.70168 | 0.1377 | 0.11705 | 0.22077 | 0.21509 | 8 | 2.93229 | 0.55262 | 0.71404 | 0.670745 | Not Significant | NS | #N/A |
| 958 | G1SFJ8 | Fatty acid-binding protein, brain OS=Oryctolagus cu | FABP7 | RABBIT | -0.2591 | 4.15174 | 0.59984 | -0.03848 | 0.06019 | -0.69534 | -0.49244 | -0.08352 | -0.14771 | 1.49869 | -0.8869 | -0.97371 | -1.08644 | 0.35872 | 0.30066 | 0.35078 | 0.34737 | 8 | 2.93229 | 0.55262 | 2.07413 | 1.666024 | Not Significant | NS | #N/A |
| 1982 | G1TUF8 | #N/A | SARM1 | RABBIT | #N/A | 0.45441 | #N/A | #N/A | #N/A | 0 | #N/A | -0.86747 | #N/A | #N/A | -0.88814 | -1.18222 | -1.01766 | 0.44697 | 0.3673 | 0.38995 | 0.38387 | 1 | 2.93229 | 0.55262 | 0.37625 | 0.507766 | Not Significant | NS | #N/A |
| 2872 | G1T1N8 | #N/A | SMPD3 | RABBIT | #N/A | #N/A | #N/A | #N/A | 2.78181 | -2.79519 | #N/A | #N/A | #N/A | #N/A | -5.577 | #N/A | -5.30485 | #N/A | 0.01385 | #N/A | 0.09774 | 0 | 2.93229 | 0.55262 | #N/A | 0.552617 | Significant | Down mtub | SMPD3 |
| 674 | G1U1Y9 | V-type proton ATPase subunit G OS=Oryctolagus cu | ATP6V1G2 | RABBIT | 0.13712 | 0.6579 | 0.72132 | -0.86595 | 0.16079 | -2.43301 | 1.13744 | -0.18982 | -1.11364 | -1.04057 | -0.89016 | -1.36231 | -1.46001 | 0.21021 | 0.17242 | 0.27469 | 0.26481 | 8 | 2.93229 | 0.55262 | 1.06738 | 0.929309 | Not Significant | NS | #N/A |
| 936 | G1SW67 | Core histone macro-H2A OS=Oryctolagus cuniculu | MACROH2 | RABBIT | 0.32037 | 0.19782 | 0.76809 | 0.99302 | -0.30664 | #N/A | -0.9139 | -0.08235 | #N/A | #N/A | -0.89266 | -2.03138 | -1.73498 | 0.09794 | 0.12128 | 0.18746 | 0.21983 | 5 | 2.93229 | 0.55262 | 0.27586 | 0.378169 | Not Significant | NS | #N/A |
| 2769 | L0TC47 | #N/A | #N/A | MT | #N/A | #N/A | #N/A | 0.84475 | #N/A | 0 | #N/A | #N/A | #N/A | -0.0968 | -0.89315 | -10.6543 | -1.13439 | 0.05958 | 0.32101 | 0.15524 | 0.35646 | 1 | 2.93229 | 0.55262 | 0.00468 | 0.413275 | Not Significant | NS | #N/A |
| 687 | A0A5F9CR18 | BRISC and BRCA1-A complex member 2 OS=Orycto | BABAM2 | RABBIT | 1.44625 | -0.37712 | 0.01194 | 0.79498 | 1.29791 | #N/A | -0.62377 | -0.06437 | 0.33335 | -0.68285 | -0.8942 | -1.96016 | -1.90608 | 0.0908 | 0.08596 | 0.18112 | 0.18814 | 7 | 2.93229 | 0.55262 | 0.46246 | 0.489077 | Not Significant | NS | #N/A |
| 431 | G1T4F3 | Semaphorin 4D OS=Oryctolagus cuniculus OX=998 | SEMA4D | RABBIT | 0.89462 | 0.46394 | #N/A | -0.32376 | #N/A | #N/A | -0.55055 | #N/A | #N/A | #N/A | -0.89548 | -1.25519 | -1.1155 | 0.3362 | 0.31602 | 0.33996 | 0.35483 | 2 | 2.93229 | 0.55262 | 0.38173 | 0.483325 | Not Significant | NS | #N/A |
| 1163 | G1SDB9 | SEC14 like lipid binding 2 OS=Oryctolagus cuniculu | SEC14L2 | RABBIT | 1.99534 | #N/A | 0 | #N/A | #N/A | -0.2847 | 0.58451 | #N/A | #N/A | 0 | -0.89773 | -1.10331 | -1.19704 | 0.35045 | 0.27693 | 0.3467 | 0.33229 | 3 | 2.93229 | 0.55262 | 0.79448 | 0.674927 | Not Significant | NS | #N/A |
| 414 | G1T235 | proteasome endopeptidase complex OS=Oryctolag | PSMB6 | RABBIT | 0.88433 | 0.07943 | 0.2164 | -0.05764 | 0.85874 | #N/A | 0.43617 | -0.24812 | -1.34074 | -0.85389 | -0.8979 | -2.21214 | -2.06248 | 0.0626 | 0.0663 | 0.15764 | 0.16941 | 7 | 2.93229 | 0.55262 | 0.36611 | 0.421174 | Not Significant | NS | #N/A |
| 671 | A0A5F9CTE8 | Microtubule associated protein 1B OS=Oryctolagus | #N/A | RABBIT | 0.31202 | 0.15466 | 0.12918 | 0.28561 | 0.30992 | -1.94974 | 0.33407 | -0.0229 | -0.65175 | -1.01443 | -0.89923 | -2.24286 | -2.13788 | 0.05518 | 0.05596 | 0.15427 | 0.15729 | 8 | 2.93229 | 0.55262 | 0.40186 | 0.442297 | Not Significant | NS | #N/A |
| 2291 | G1SGR6 | #N/A | RPL29 | RABBIT | #N/A | #N/A | 0.36302 | 0.17218 | 0.73968 | #N/A | -0.3654 | -0.25673 | #N/A | -0.80081 | -0.89927 | -3.81955 | -2.07502 | 0.01879 | 0.07704 | 0.11224 | 0.18168 | 4 | 2.93229 | 0.55262 | 0.08315 | 0.281728 | Not Significant | NS | #N/A |
| 2028 | A0A5F9DSH1 | #N/A | GMPR2 GN | RABBIT | #N/A | 3.6405 | -1.84062 | #N/A | #N/A | #N/A | -0.43647 | #N/A | 0.43647 | #N/A | -0.89994 | -0.32429 | -0.48442 | 0.77649 | 0.64883 | 0.50687 | 0.49641 | 2 | 2.93229 | 0.55262 | 7.70116 | 3.45129 | Not Significant | NS | #N/A |
| 1433 | G1T0I5 | Presequence protease, mitochondrial OS=Oryctola | PITRM1 | RABBIT | #N/A | 0.19782 | 0.83743 | #N/A | #N/A | #N/A | #N/A | #N/A | #N/A | -5.02683 | -5.54445 | -10.0096 | -6.64518 | 0.06339 | 0.00283 | 0.15826 | 0.05387 | 1 | 2.93229 | 0.55262 | 0.20455 | 0.464101 | Significant | Down mtub | PITRM1 |
| 2191 | G1SCK3 | #N/A | WDR81 | RABBIT | #N/A | #N/A | 0.17065 | #N/A | 0.5615 | -0.89075 | -1.56017 | 0.18574 | 1.19391 | -1.60857 | -0.90204 | -0.99125 | -1.10617 | 0.36708 | 0.30107 | 0.35413 | 0.34761 | 5 | 2.93229 | 0.55262 | 1.18301 | 0.949976 | Not Significant | NS | #N/A |
| 563 | G1U971 | Eukaryotic translation initiation factor 3 subunit C C | EIF3C EIF3 | RABBIT | 0.01166 | #N/A | #N/A | 0.32687 | #N/A | #N/A | -0.73287 | #N/A | #N/A | #N/A | -0.90214 | -3.30477 | -1.13026 | 0.18706 | 0.32256 | 0.26045 | 0.35646 | 1 | 2.93229 | 0.55262 | 0.04968 | 0.424717 | Not Significant | NS | #N/A |
| 2157 | G1SWV9 | #N/A | NAPB | RABBIT | #N/A | #N/A | 2.04374 | -0.37411 | #N/A | #N/A | 0.10326 | 0.42986 | -0.7004 | -0.10326 | -0.90245 | -1.09822 | -1.20066 | 0.33378 | 0.2693 | 0.33821 | 0.32966 | 4 | 2.93229 | 0.55262 | 0.90034 | 0.753259 | Not Significant | NS | #N/A |
| 1120 | A0A5F9DP20 | LUC7 like OS=Oryctolagus cuniculus OX=9986 GN= | LUC7L | RABBIT | 1.7871 | -0.1101 | #N/A | #N/A | #N/A | #N/A | #N/A | -0.06437 | #N/A | #N/A | -0.90287 | -0.54951 | -0.79046 | 0.68012 | 0.4742 | 0.47768 | 0.432 | 1 | 2.93229 | 0.55262 | 1.7997 | 0.869755 | Not Significant | NS | #N/A |
| 1075 | G1SH26 | Nucleoredoxin OS=Oryctolagus cuniculus OX=998 | #N/A | RABBIT | 0.90034 | 3.64128 | 0.33867 | -0.92911 | -0.40793 | -0.04196 | -0.87931 | -0.0224 | -0.12589 | 0.09113 | -0.90434 | -1.10851 | -1.2235 | 0.29985 | 0.24686 | 0.32411 | 0.3161 | 8 | 2.93229 | 0.55262 | 1.66389 | 1.365822 | Not Significant | NS | #N/A |
| 2731 | G1STB0 | #N/A | MIS18BP1 | RABBIT | #N/A | #N/A | #N/A | 0.42356 | #N/A | -0.48119 | #N/A | #N/A | #N/A | #N/A | -0.90475 | #N/A | -0.8606 | #N/A | 0.45407 | #N/A | 0.42274 | 0 | 2.93229 | 0.55262 | #N/A | 0.552617 | Not Significant | NS | #N/A |
| 331 | P67873 | Casein kinase II subunit beta OS=Oryctolagus cunic | CSNK2B C | RABBIT | 0.12845 | -0.36616 | 0.71769 | -0.17442 | 1.19999 | -1.82163 | 0.1181 | 0.14206 | -0.53048 | -0.92804 | -0.90511 | -1.93991 | -1.93591 | 0.08836 | 0.07915 | 0.17722 | 0.18311 | 8 | 2.93229 | 0.55262 | 0.54422 | 0.546473 | Not Significant | NS | #N/A |
| 2443 | G1SRC9 | #N/A | BIRC6 | RABBIT | #N/A | #N/A | 0.63423 | #N/A | #N/A | #N/A | 0.38815 | -0.81633 | -0.38815 | #N/A | -0.90634 | -1.28555 | -1.13328 | 0.32736 | 0.30916 | 0.33637 | 0.35209 | 2 | 2.93229 | 0.55262 | 0.37279 | 0.479698 | Not Significant | NS | #N/A |
| 900 | G1T345 | E3 ubiquitin-protein ligase OS=Oryctolagus cunicul | UBR1 | RABBIT | 0.60917 | -0.13128 | 0.1457 | 0.27951 | 1.79717 | #N/A | 0 | -0.51841 | 0.96936 | -1.92828 | -0.90939 | -1.39187 | -1.48648 | 0.20658 | 0.16819 | 0.27238 | 0.26263 | 7 | 2.93229 | 0.55262 | 0.94861 | 0.831699 | Not Significant | NS | #N/A |
| 1185 | A0A5F9DVJ1 | Cell adhesion molecule 2 OS=Oryctolagus cuniculu | CADM2 | RABBIT | 0.09326 | 1.54169 | -0.99027 | 0.10707 | 0.17286 | -0.37154 | -0.0816 | -1.72762 | #N/A | #N/A | -0.91184 | -1.39879 | -1.47494 | 0.21139 | 0.17458 | 0.27499 | 0.26652 | 6 | 2.93229 | 0.55262 | 0.79677 | 0.716622 | Not Significant | NS | #N/A |
| 1530 | P9WJU7 | Phthiocerol dimycocerosate exporter MmpL7 OS=M | #N/A | MT | 0.36463 | #N/A | #N/A | #N/A | -0.01338 | #N/A | #N/A | -0.7363 | #N/A | #N/A | -0.91193 | -2.78561 | -1.13515 | 0.21942 | 0.32073 | 0.28192 | 0.35646 | 1 | 2.93229 | 0.55262 | 0.07145 | 0.430253 | Not Significant | NS | #N/A |
| 867 | A0A5F9D1R0 | Protein kinase C and casein kinase substrate in neu | PACSIN2 | RABBIT | 2.52279 | 1.51912 | #N/A | -0.02327 | -1.43395 | 0.91548 | #N/A | -0.09873 | -0.97556 | -0.90823 | -0.91293 | -0.9367 | -1.06941 | 0.38507 | 0.31293 | 0.36278 | 0.35298 | 6 | 2.93229 | 0.55262 | 1.89977 | 1.457529 | Not Significant | NS | #N/A |
| 2748 | P96901 | #N/A | #N/A | MT | #N/A | #N/A | #N/A | 0.428 | #N/A | #N/A | -0.48564 | #N/A | #N/A | #N/A | -0.91363 | #N/A | -0.86905 | #N/A | 0.4501 | #N/A | 0.42119 | 0 | 2.93229 | 0.55262 | #N/A | 0.552617 | Not Significant | NS | #N/A |
| 122 | G1TVT1 | Adducin 1 OS=Oryctolagus cuniculus OX=9986 GN | ADD1 | RABBIT | 0.58802 | 0.20879 | -0.02657 | 0.10829 | 0.71536 | -2.44043 | 0.24246 | -0.07534 | -0.27469 | -0.43541 | -0.91546 | -1.84753 | -1.87274 | 0.10186 | 0.08807 | 0.19155 | 0.18947 | 8 | 2.93229 | 0.55262 | 0.61381 | 0.597397 | Not Significant | NS | #N/A |
| 330 | A0A5F9CNZ2 | Bleomycin hydrolase OS=Oryctolagus cuniculus O) | BLMH | RABBIT | 1.07031 | #N/A | 0.15714 | #N/A | 0.42518 | #N/A | #N/A | -0.72987 | #N/A | 0 | -0.91581 | -2.06657 | -1.60166 | 0.13067 | 0.16091 | 0.21527 | 0.25569 | 3 | 2.93229 | 0.55262 | 0.23567 | 0.392332 | Not Significant | NS | #N/A |
| 1402 | A0A5F9CZ86 | Tyrosine-protein kinase OS=Oryctolagus cuniculus | ABL1 | RABBIT | -0.53694 | #N/A | 0.66686 | #N/A | 0.55992 | -0.20833 | 0.20833 | 0.17964 | -3.09551 | -0.52455 | -0.91803 | -1.05633 | -1.18115 | 0.33148 | 0.26804 | 0.33727 | 0.32896 | 6 | 2.93229 | 0.55262 | 1.41617 | 1.132684 | Not Significant | NS | #N/A |
| 326 | A0A5F9DPZ4 | Nucleosome assembly protein 1 like 1 OS=Oryctola | NAP1L1 | RABBIT | 0.50487 | 0.52923 | -0.26728 | -0.03962 | 0.30104 | -2.0856 | 0.39943 | -0.08239 | -1.34987 | -0.4462 | -0.91857 | -1.94017 | -1.94379 | 0.08832 | 0.0781 | 0.17722 | 0.18205 | 8 | 2.93229 | 0.55262 | 0.56039 | 0.558305 | Not Significant | NS | #N/A |
| 2480 | G1TAJ3 | #N/A | #N/A | RABBIT | #N/A | #N/A | #N/A | 0.4308 | #N/A | #N/A | #N/A | #N/A | -0.48844 | #N/A | -0.91923 | #N/A | -0.87438 | #N/A | 0.44762 | #N/A | 0.42011 | 0 | 2.93229 | 0.55262 | #N/A | 0.552617 | Not Significant | NS | #N/A |
| 895 | B7NZE6 | Tumor protein p53 binding protein 1 (Predicted), 5 p | TP53BP1 | RABBIT | 0.476 | -0.38426 | 0.01941 | #N/A | 0.98211 | -1.44487 | #N/A | #N/A | #N/A | 0.15125 | -0.92013 | -1.39686 | -1.41028 | 0.23497 | 0.20171 | 0.29167 | 0.28418 | 4 | 2.93229 | 0.55262 | 0.57853 | 0.567571 | Not Significant | NS | #N/A |
| 115 | A0A5F9CTH5 | Microtubule-associated protein OS=Oryctolagus cu | MAP2 | RABBIT | -0.17115 | 0.89032 | -2.21353 | 2.08208 | 0.49684 | -3.35884 | 0.18281 | 0.62998 | -0.61203 | -0.36214 | -0.92096 | -0.92541 | -1.04009 | 0.38182 | 0.32075 | 0.36126 | 0.35646 | 8 | 2.93229 | 0.55262 | 2.47597 | 1.960081 | Not Significant | NS | #N/A |
| 2042 | Q8WMG3 | #N/A | ATP1B2 | RABBIT | #N/A | #N/A | 1.15783 | 1.13983 | -0.30837 | 0 | -1.41752 | 0.92441 | #N/A | -0.54274 | -0.92206 | -1.30091 | -1.3967 | 0.25001 | 0.20034 | 0.2994 | 0.28371 | 5 | 2.93229 | 0.55262 | 0.8612 | 0.747125 | Not Significant | NS | #N/A |
| 605 | Q29502 | Serine/threonine-protein kinase PAK 2 OS=Oryctola | PAK2 | RABBIT | -0.27417 | 1.48122 | #N/A | 0.18241 | #N/A | -1.7885 | 0.10054 | -0.16491 | 0.26031 | -0.70265 | -0.92219 | -1.47412 | -1.53776 | 0.19089 | 0.15874 | 0.26255 | 0.25503 | 6 | 2.93229 | 0.55262 | 0.73381 | 0.674325 | Not Significant | NS | #N/A |
| 768 | A0A5F9CGL3 | SH3 domain containing GRB2 like 3, endophilin A3 ( | SH3GL3 | RABBIT | 1.24322 | 0.80816 | -1.10587 | #N/A | #N/A | #N/A | #N/A | #N/A | #N/A | -0.61034 | -0.92551 | -0.64135 | -0.81726 | 0.58698 | 0.45144 | 0.44596 | 0.42172 | 2 | 2.93229 | 0.55262 | 1.56184 | 0.961846 | Not Significant | NS | #N/A |
| 65 | A0A5F9D287 | phosphopyruvate hydratase OS=Oryctolagus cunic | ENO1 | RABBIT | 0.76981 | 1.83861 | -0.55593 | 0.3646 | 0.16721 | -0.75291 | 0.30342 | -0.76449 | -0.67251 | -0.18059 | -0.93028 | -2.08511 | -2.05488 | 0.07055 | 0.06459 | 0.16349 | 0.16683 | 8 | 2.93229 | 0.55262 | 0.49763 | 0.51238 | Not Significant | NS | #N/A |
| 1632 | G1SFF5 | Serine palmitoyltransferase long chain base subuni | SPTLC2 | RABBIT | -0.57327 | 0.64924 | #N/A | 1.18048 | 0.14713 | #N/A | -0.08652 | 0.02216 | -1.40846 | -0.84754 | -0.93099 | -1.85064 | -1.82337 | 0.11369 | 0.10181 | 0.20028 | 0.20137 | 6 | 2.93229 | 0.55262 | 0.50614 | 0.521399 | Not Significant | NS | #N/A |
| 2492 | G1SJC7 | #N/A | G3BP2 | RABBIT | #N/A | #N/A | #N/A | 0.85019 | #N/A | -0.49306 | 0.39127 | 0.13966 | -0.20403 | -0.23838 | -0.9311 | -2.45 | -1.54362 | 0.07045 | 0.16701 | 0.16349 | 0.26191 | 4 | 2.93229 | 0.55262 | 0.12036 | 0.303199 | Not Significant | NS | #N/A |
| 84 | G1T3Y8 | 60 kDa heat shock protein, mitochondrial OS=Oryct | HSPD1 | RABBIT | 0.7716 | 1.28319 | -0.14934 | 0.104 | -0.17502 | -2.51611 | -0.69953 | 0.6847 | -0.63442 | 0.3437 | -0.93122 | -1.48639 | -1.58195 | 0.17548 | 0.14214 | 0.2543 | 0.24166 | 8 | 2.93229 | 0.55262 | 0.98124 | 0.866273 | Not Significant | NS | #N/A |
| 559 | G1ST90 | Cystathionine gamma-lyase OS=Oryctolagus cunic | CTH | RABBIT | 0.06261 | 0.66561 | 0.11971 | -0.52313 | 1.38322 | #N/A | -1.96273 | -0.36003 | 0.54946 | #N/A | -0.9327 | -1.35882 | -1.45092 | 0.22306 | 0.181 | 0.28411 | 0.27126 | 6 | 2.93229 | 0.55262 | 0.8834 | 0.774814 | Not Significant | NS | #N/A |
| 1456 | A0A5F9C227 | Coiled-coil domain containing 102B OS=Oryctolagu | #N/A | RABBIT | #N/A | #N/A | #N/A | #N/A | 4.79949 | #N/A | 0 | #N/A | -1.22351 | #N/A | -5.41124 | -5.10694 | -5.69245 | 0.1231 | 0.00495 | 0.2103 | 0.06702 | 1 | 2.93229 | 0.55262 | 0.74848 | 0.602427 | Significant | Down mtub | #N/A |
| 1913 | G1SGW1 | #N/A | TAP1 | RABBIT | #N/A | 0.53386 | #N/A | #N/A | #N/A | #N/A | #N/A | -0.4004 | #N/A | #N/A | -0.93426 | #N/A | -0.88867 | #N/A | 0.44101 | #N/A | 0.41692 | 0 | 2.93229 | 0.55262 | #N/A | 0.552617 | Not Significant | NS | #N/A |
| 1357 | A0A5F9CZP1 | Centrosomal protein 290 OS=Oryctolagus cuniculu | CEP290 | RABBIT | 0.01166 | #N/A | -0.84887 | #N/A | 2.50591 | #N/A | #N/A | #N/A | 0.75955 | -1.51627 | -0.9346 | -0.60252 | -0.77764 | 0.58933 | 0.46665 | 0.44648 | 0.42808 | 3 | 2.93229 | 0.55262 | 2.88728 | 1.73327 | Not Significant | NS | #N/A |
| 193 | G1T7Y7 | F-actin-capping protein subunit beta OS=Oryctolag | CAPZB | RABBIT | 0.13339 | 0.08288 | 0.60231 | 0.37408 | 0.97507 | -2.08866 | -0.12652 | -0.29467 | 0.05647 | -0.05647 | -0.93551 | -2.16043 | -2.1104 | 0.06275 | 0.05869 | 0.15764 | 0.16071 | 8 | 2.93229 | 0.55262 | 0.46877 | 0.49126 | Not Significant | NS | #N/A |
| 1883 | G1TF14 | #N/A | TMSB10 | RABBIT | #N/A | 0.68691 | #N/A | #N/A | #N/A | #N/A | -0.10762 | #N/A | -0.74807 | 0.10762 | -0.93627 | -1.82165 | -1.26805 | 0.2101 | 0.26134 | 0.27469 | 0.32441 | 2 | 2.93229 | 0.55262 | 0.19812 | 0.408872 | Not Significant | NS | #N/A |
| 1073 | G1TI55 | Mitochondrial import inner membrane translocase | TIMM9 | RABBIT | 1.35036 | 1.77868 | -0.69252 | #N/A | -0.01338 | -0.60832 | #N/A | #N/A | 0.30616 | -0.6917 | -0.93707 | -1.27748 | -1.38415 | 0.25753 | 0.20401 | 0.3027 | 0.28553 | 5 | 2.93229 | 0.55262 | 0.9224 | 0.785707 | Not Significant | NS | #N/A |
| 856 | A0A5F9DTT8 | NAD-dependent protein deacetylase sirtuin-2 OS=C | SIRT2 | RABBIT | -1.88638 | -0.31499 | 0.51302 | 0.18042 | 0.22813 | 0.36624 | -0.23806 | 0.77717 | -3.39959 | -3.48122 | -0.93913 | -0.91633 | -1.03212 | 0.38628 | 0.32431 | 0.36369 | 0.35781 | 8 | 2.93229 | 0.55262 | 2.62594 | 2.06983 | Not Significant | NS | #N/A |
| 1443 | G1T8W6 | RAB guanine nucleotide exchange factor 1 OS=Oryc | RABGEF1 | RABBIT | -0.40843 | #N/A | #N/A | 0.36246 | 0.71951 | #N/A | #N/A | #N/A | -0.71575 | #N/A | -0.94026 | -1.41251 | -1.19633 | 0.29332 | 0.28589 | 0.32101 | 0.33729 | 2 | 2.93229 | 0.55262 | 0.33233 | 0.463294 | Not Significant | NS | #N/A |
| 1891 | G1TJL9 | #N/A | #N/A | RABBIT | #N/A | 0.56995 | #N/A | #N/A | #N/A | -0.37213 | #N/A | #N/A | #N/A | #N/A | -0.94207 | #N/A | -0.8961 | #N/A | 0.43761 | #N/A | 0.41582 | 0 | 2.93229 | 0.55262 | #N/A | 0.552617 | Not Significant | NS | #N/A |
| 2203 | G1TF29 | #N/A | ATP8A1 | RABBIT | #N/A | #N/A | 0.72001 | #N/A | -0.01338 | -0.53939 | -1.9275 | 0.70018 | #N/A | #N/A | -0.94222 | -0.92628 | -1.08733 | 0.42262 | 0.31908 | 0.37981 | 0.35646 | 3 | 2.93229 | 0.55262 | 1.24166 | 0.901069 | Not Significant | NS | #N/A |
| 659 | G1TVG7 | Splicing factor 3B subunit 1 domain-containing prot | #N/A | RABBIT | -0.02366 | -1.27729 | 0.20597 | 0.55189 | 0.77056 | -4.42456 | 0.59182 | 0.46787 | -0.30994 | -0.8186 | -0.94418 | -0.95777 | -1.07568 | 0.36622 | 0.30524 | 0.35413 | 0.35029 | 8 | 2.93229 | 0.55262 | 2.42954 | 1.926107 | Not Significant | NS | #N/A |
| 1599 | A0A5F9CP59 | TBC1 domain family member 22B OS=Oryctolagus | TBC1D22E | RABBIT | 0.51123 | #N/A | #N/A | #N/A | #N/A | 0.66824 | #N/A | #N/A | -0.49957 | -1.47129 | -0.94544 | -0.76431 | -0.91895 | 0.52454 | 0.40083 | 0.42469 | 0.40169 | 2 | 2.93229 | 0.55262 | 1.14759 | 0.793874 | Not Significant | NS | #N/A |
| 1748 | G1TKX3 | #N/A | FGG | RABBIT | #N/A | -0.5838 | #N/A | 0.60266 | 0.4211 | -1.88242 | 2.15377 | 0.35845 | -4.20583 | -0.42282 | -0.94642 | -0.65073 | -0.76825 | 0.53933 | 0.46217 | 0.42859 | 0.42567 | 6 | 2.93229 | 0.55262 | 3.9662 | 2.84559 | Not Significant | NS | #N/A |
| 1188 | G1SGF5 | Plexin B1 OS=Oryctolagus cuniculus OX=9986 GN= | PLXNB1 | RABBIT | 0.22474 | #N/A | #N/A | 0.68495 | -0.22646 | #N/A | -0.72394 | #N/A | #N/A | #N/A | -0.95168 | -1.80856 | -1.28287 | 0.21225 | 0.25651 | 0.27513 | 0.3222 | 2 | 2.93229 | 0.55262 | 0.20767 | 0.412746 | Not Significant | NS | #N/A |
| 98 | P13491 | L-lactate dehydrogenase A chain OS=Oryctolagus c | LDHA | RABBIT | 0.93989 | 1.36338 | -0.22661 | #N/A | 0.26162 | -1.11576 | 0.32294 | -0.33937 | #N/A | -0.3377 | -0.95204 | -2.07203 | -1.97437 | 0.08365 | 0.08002 | 0.17453 | 0.18325 | 6 | 2.93229 | 0.55262 | 0.42223 | 0.465034 | Not Significant | NS | #N/A |
| 1665 | A0A5F9C125 | #N/A | ILF3 | RABBIT | #N/A | 0.54786 | #N/A | 0.53698 | #N/A | #N/A | -0.34709 | 0.28273 | -1.18849 | -0.38834 | -0.95272 | -2.10703 | -1.75922 | 0.10283 | 0.12236 | 0.19228 | 0.22013 | 4 | 2.93229 | 0.55262 | 0.2726 | 0.391045 | Not Significant | NS | #N/A |
| 236 | U3KME3 | Adducin 3 OS=Oryctolagus cuniculus OX=9986 GN | ADD3 | RABBIT | 0.86752 | -0.08307 | 0.35292 | -0.23848 | 1.00965 | -2.63079 | 1.02338 | 0.11647 | -1.03466 | -0.33231 | -0.95329 | -1.44032 | -1.54675 | 0.18774 | 0.15037 | 0.26069 | 0.24933 | 8 | 2.93229 | 0.55262 | 1.09514 | 0.949625 | Not Significant | NS | #N/A |
| 1446 | G1U0B4 | Cytochrome c domain-containing protein OS=Oryct | #N/A | RABBIT | -0.20967 | 1.84254 | #N/A | #N/A | #N/A | #N/A | #N/A | -0.49769 | #N/A | 0.22133 | -0.95462 | -0.878 | -1.06207 | 0.47255 | 0.33741 | 0.40195 | 0.36661 | 2 | 2.93229 | 0.55262 | 1.18214 | 0.807882 | Not Significant | NS | #N/A |
| 2591 | A0A5F9CVY7 | #N/A | ADAM10 | RABBIT | #N/A | #N/A | #N/A | 0.72556 | #N/A | -0.46264 | 0 | #N/A | #N/A | #N/A | -0.95688 | -2.38827 | -1.17878 | 0.25244 | 0.30486 | 0.30043 | 0.3501 | 1 | 2.93229 | 0.55262 | 0.10702 | 0.439299 | Not Significant | NS | #N/A |
| 743 | G1SU97 | Dihydrolipoamide acetyltransferase component of | PDHX | RABBIT | -0.07105 | 0.28054 | 2.02735 | -1.04441 | 1.31876 | -1.62634 | -2.13876 | 1.58038 | -0.66584 | 0.57715 | -0.95692 | -1.09686 | -1.21897 | 0.30462 | 0.2485 | 0.32493 | 0.31654 | 8 | 2.93229 | 0.55262 | 1.90278 | 1.540638 | Not Significant | NS | #N/A |
| 1949 | A4L9L8 | #N/A | Bfsp2 | RABBIT | #N/A | 0.57748 | #N/A | #N/A | #N/A | #N/A | -0.37966 | #N/A | #N/A | #N/A | -0.95714 | #N/A | -0.91044 | #N/A | 0.43112 | #N/A | 0.41226 | 0 | 2.93229 | 0.55262 | #N/A | 0.552617 | Not Significant | NS | #N/A |
| 100 | G1T6I6 | Interleukin enhancer binding factor 3 OS=Oryctolag | ILF3 | RABBIT | -0.09577 | #N/A | 0.27808 | #N/A | 0.39322 | -5.20682 | #N/A | #N/A | #N/A | #N/A | -5.39866 | -18.2885 | -7.84658 | 0.00298 | 0.00057 | 0.06045 | 0.02813 | 2 | 2.93229 | 0.55262 | 0.06535 | 0.355036 | Significant | Down mtub | ILF3 |

| | | | | | | | | | | | | | | | | | | | | | | | | | | | | | |
|---|---|---|---|---|---|---|---|---|---|---|---|---|---|---|---|---|---|---|---|---|---|---|---|---|---|---|---|---|---|
| 385 | G1TCQ4 | Glucosamine-6-phosphate isomerase OS=Oryctola | GNPDA1 | RABBIT | 1.60431 | 1.88165 | -1.50817 | -0.05764 | 0.48784 | #N/A | 0.8892 | -1.39451 | -1.35485 | -0.05944 | -0.9615 | -1.13701 | -1.26534 | 0.29295 | 0.23463 | 0.32101 | 0.30522 | 7 | 2.93229 | 0.55262 | 1.58912 | 1.283117 | Not Significant | NS | #N/A |
| 1583 | G1TYE2 | Latexin OS=Oryctolagus cuniculus OX=9986 GN=L) | LXN | RABBIT | -0.18903 | 3.28395 | #N/A | -0.09565 | #N/A | #N/A | #N/A | #N/A | #N/A | 0.03801 | -0.96175 | -0.42093 | -0.60169 | 0.71473 | 0.57396 | 0.48903 | 0.46967 | 2 | 2.93229 | 0.55262 | 3.91532 | 1.916165 | Not Significant | NS | #N/A |
| 656 | A0A5F9CJE1 | Calsyntenin 1 OS=Oryctolagus cuniculus OX=9986 | CLSTN1 | RABBIT | 0.32792 | 1.30907 | -2.53052 | 1.99701 | 0.37386 | -2.26493 | -0.45119 | -0.62838 | 0.1897 | -0.1897 | -0.96437 | -1.09619 | -1.2192 | 0.3049 | 0.24842 | 0.32493 | 0.31654 | 8 | 2.93229 | 0.55262 | 1.9349 | 1.564142 | Not Significant | NS | #N/A |
| 1478 | A0A5F9CZ92 | Solute carrier family 37 member 4 OS=Oryctolagus | SLC37A4 | RABBIT | 0.48844 | #N/A | #N/A | #N/A | #N/A | -0.47678 | #N/A | #N/A | #N/A | #N/A | -0.96522 | #N/A | -0.91812 | #N/A | 0.42768 | #N/A | 0.41011 | 0 | 2.93229 | 0.55262 | #N/A | 0.552617 | Not Significant | NS | #N/A |
| 1989 | G1U9H0 | #N/A | LRRC69 | RABBIT | #N/A | 0.19782 | #N/A | #N/A | 0.67281 | -1.24551 | 0.34865 | #N/A | -0.69426 | #N/A | -0.96569 | -1.53556 | -1.47673 | 0.22222 | 0.19076 | 0.28347 | 0.27624 | 3 | 2.93229 | 0.55262 | 0.47459 | 0.513158 | Not Significant | NS | #N/A |
| 2083 | A0A5F9DVG9 | #N/A | NUDT19 | RABBIT | #N/A | 1.22313 | #N/A | -0.03902 | -0.032 | #N/A | -0.58319 | #N/A | #N/A | #N/A | -0.96722 | -1.15268 | -1.13708 | 0.36821 | 0.30771 | 0.35421 | 0.35174 | 2 | 2.93229 | 0.55262 | 0.52808 | 0.542665 | Not Significant | NS | #N/A |
| 1963 | A0A5F9C7N1 | #N/A | #N/A | RABBIT | #N/A | 0.55059 | #N/A | #N/A | #N/A | #N/A | #N/A | -0.41713 | #N/A | #N/A | -0.96772 | #N/A | -0.9205 | #N/A | 0.42662 | #N/A | 0.41011 | 0 | 2.93229 | 0.55262 | #N/A | 0.552617 | Not Significant | NS | #N/A |
| 1278 | Q9GJP9 | Skeletal muscle myosin heavy chain MyHC-EO/IIL C | MYH13 | RABBIT | 0.01166 | 1.58294 | #N/A | #N/A | #N/A | #N/A | #N/A | #N/A | -4.46478 | #N/A | -5.26208 | -3.867 | -5.04243 | 0.1611 | 0.0076 | 0.24012 | 0.08098 | 1 | 2.93229 | 0.55262 | 1.23446 | 0.726012 | Significant | Down mtub | MYH13 |
| 2076 | P9WNH3 | #N/A | #N/A | MT | #N/A | 0.79081 | #N/A | -1.42051 | 1.75486 | #N/A | -0.59299 | #N/A | #N/A | #N/A | -0.96804 | -0.51496 | -0.70771 | 0.65785 | 0.51115 | 0.47085 | 0.44921 | 2 | 2.93229 | 0.55262 | 2.65038 | 1.403243 | Not Significant | NS | #N/A |
| 1383 | A0A5F9CQB2 | Selenide, water dikinase 1 OS=Oryctolagus cunicul | SEPHS1 | RABBIT | -0.06913 | 2.42466 | #N/A | 0.91867 | -0.77382 | #N/A | -0.70335 | 0.01643 | #N/A | #N/A | -0.96855 | -0.91145 | -1.06512 | 0.41362 | 0.32251 | 0.37693 | 0.35646 | 4 | 2.93229 | 0.55262 | 1.50565 | 1.102524 | Not Significant | NS | #N/A |
| 1338 | A0A5F9D144 | Tudor domain-containing protein 7 OS=Oryctolagus | TDRD7 | RABBIT | 0.76587 | 0.1475 | 0.32178 | -0.08355 | 0.6181 | -2.6572 | 0.4073 | -0.03845 | -0.25182 | -0.53393 | -0.96876 | -1.74543 | -1.81556 | 0.11906 | 0.09694 | 0.20675 | 0.19688 | 8 | 2.93229 | 0.55262 | 0.77013 | 0.711791 | Not Significant | NS | #N/A |
| 2001 | A0A5F9D600 | #N/A | NSUN2 | RABBIT | #N/A | 0.55556 | 1.02134 | -0.41537 | #N/A | -0.58318 | #N/A | #N/A | #N/A | #N/A | -0.97036 | -1.14645 | -1.13685 | 0.37027 | 0.3078 | 0.35549 | 0.35174 | 2 | 2.93229 | 0.55262 | 0.5373 | 0.546406 | Not Significant | NS | #N/A |
| 5 | G1SN11 | Spectrin beta chain OS=Oryctolagus cuniculus OX= | SPTBN1 | RABBIT | 0.51353 | -0.44548 | 0.48302 | -0.23607 | 0.98049 | -3.59018 | 0.45702 | 0.11407 | -0.22706 | -0.31288 | -0.9709 | -1.24802 | -1.37009 | 0.24731 | 0.19814 | 0.29795 | 0.28262 | 8 | 2.93229 | 0.55262 | 1.51303 | 1.255429 | Not Significant | NS | #N/A |
| 2879 | G1T084 | #N/A | PELI3 | RABBIT | #N/A | #N/A | #N/A | #N/A | 0.48037 | #N/A | -0.49376 | #N/A | #N/A | #N/A | -0.97413 | #N/A | -0.92659 | #N/A | 0.42391 | #N/A | 0.40956 | 0 | 2.93229 | 0.55262 | #N/A | 0.552617 | Not Significant | NS | #N/A |
| 1461 | G1T1Z5 | Extracellular matrix protein 1 OS=Oryctolagus cunic | ECM1 | RABBIT | 0.01166 | #N/A | #N/A | 1.27543 | #N/A | #N/A | #N/A | #N/A | -0.33497 | #N/A | -0.97852 | -0.89407 | -1.01866 | 0.53557 | 0.36688 | 0.42838 | 0.38387 | 1 | 2.93229 | 0.55262 | 0.79855 | 0.61516 | Not Significant | NS | #N/A |
| 2795 | Q9BED4 | #N/A | kir6.2 | RABBIT | #N/A | #N/A | #N/A | #N/A | 0.7941 | #N/A | #N/A | #N/A | 0 | -0.37294 | -0.98058 | -3.03603 | -1.22129 | 0.20256 | 0.2901 | 0.27183 | 0.34045 | 1 | 2.93229 | 0.55262 | 0.06954 | 0.429769 | Not Significant | NS | #N/A |
| 2132 | A0A5F9D4Q1 | #N/A | OGA | RABBIT | #N/A | #N/A | 0.17065 | 0.29152 | #N/A | #N/A | #N/A | -0.74957 | #N/A | #N/A | -0.98066 | -9.36852 | -1.24452 | 0.0677 | 0.28233 | 0.1625 | 0.33546 | 1 | 2.93229 | 0.55262 | 0.0073 | 0.413941 | Not Significant | NS | #N/A |
| 433 | G1SIJ2 | Acetyl-CoA acetyltransferase, mitochondrial OS=O | ACAT1 | RABBIT | 0.40436 | 1.00308 | -0.52548 | 1.17923 | 0.39599 | -0.94908 | -1.71358 | 1.11279 | -0.51047 | -0.3927 | -0.98204 | -1.78225 | -1.85104 | 0.11256 | 0.09135 | 0.20012 | 0.19284 | 8 | 2.93229 | 0.55262 | 0.75904 | 0.703675 | Not Significant | NS | #N/A |
| 2472 | A5U0V3 | #N/A | #N/A | MT | #N/A | #N/A | 0.5766 | #N/A | #N/A | #N/A | #N/A | #N/A | -0.40595 | #N/A | -0.98254 | #N/A | -0.9346 | #N/A | 0.42038 | #N/A | 0.40859 | 0 | 2.93229 | 0.55262 | #N/A | 0.552617 | Not Significant | NS | #N/A |
| 1915 | A0A5F9D053 | #N/A | RASA1 | RABBIT | #N/A | #N/A | #N/A | 0.8138 | #N/A | -0.6874 | -0.44542 | 0.40958 | -0.58003 | 0.44542 | -0.98537 | -1.62456 | -1.40369 | 0.17958 | 0.20359 | 0.25448 | 0.28553 | 4 | 2.93229 | 0.55262 | 0.30658 | 0.410652 | Not Significant | NS | #N/A |
| 2800 | G1T042 | #N/A | SLC8A1 | RABBIT | #N/A | #N/A | #N/A | #N/A | 0.54206 | #N/A | 0.41756 | -1.61647 | 0 | -0.58054 | -0.98692 | -1.00105 | -1.08135 | 0.39057 | 0.32153 | 0.36581 | 0.35646 | 3 | 2.93229 | 0.55262 | 0.77758 | 0.666382 | Not Significant | NS | #N/A |
| 2497 | A0A5F9DPQ8 | #N/A | TP53BP1 | RABBIT | #N/A | #N/A | #N/A | 0.80663 | #N/A | #N/A | 0 | -0.36168 | #N/A | #N/A | -0.98748 | -3.15258 | -1.23139 | 0.19555 | 0.2867 | 0.26573 | 0.33784 | 1 | 2.93229 | 0.55262 | 0.06541 | 0.428717 | Not Significant | NS | #N/A |
| 798 | G1SLU7 | Guanylate cyclase soluble subunit alpha-1 OS=Ory | GUCY1A1 | RABBIT | 1.37678 | 0.22201 | -1.03634 | 1.45944 | 2.28744 | -0.498 | -1.29115 | -0.08855 | 1.66901 | -0.42012 | -0.98763 | -1.30398 | -1.4269 | 0.22851 | 0.18154 | 0.28853 | 0.27161 | 8 | 2.93229 | 0.55262 | 1.43413 | 1.197687 | Not Significant | NS | #N/A |
| 2295 | G1TIS9 | #N/A | #N/A | RABBIT | #N/A | #N/A | 1.20929 | #N/A | 0.40794 | 0 | -0.07383 | -0.47273 | #N/A | #N/A | -0.9908 | -2.80107 | -1.83702 | 0.06779 | 0.11642 | 0.1625 | 0.21451 | 3 | 2.93229 | 0.55262 | 0.15014 | 0.349083 | Not Significant | NS | #N/A |
| 2505 | G1SQ93 | #N/A | AGO2 | RABBIT | #N/A | #N/A | #N/A | 0.69972 | #N/A | -0.58324 | #N/A | #N/A | #N/A | 0 | -0.99134 | -1.96266 | -1.19953 | 0.29999 | 0.29757 | 0.32411 | 0.34543 | 1 | 2.93229 | 0.55262 | 0.17008 | 0.455337 | Not Significant | NS | #N/A |
| 2015 | B7NZR6 | #N/A | FUNDC2 | RABBIT | #N/A | 0.56264 | #N/A | #N/A | #N/A | #N/A | #N/A | -0.42918 | #N/A | #N/A | -0.99182 | #N/A | -0.94342 | #N/A | 0.41652 | #N/A | 0.40855 | 0 | 2.93229 | 0.55262 | #N/A | 0.552617 | Not Significant | NS | #N/A |
| 2757 | G1SU35 | #N/A | DNAH1 | RABBIT | #N/A | #N/A | #N/A | #N/A | 4.83473 | #N/A | #N/A | -0.5993 | 0.53493 | -1.15343 | -5.24067 | -5.27352 | -5.72314 | 0.03413 | 0.00238 | 0.13313 | 0.05138 | 2 | 2.93229 | 0.55262 | 0.74068 | 0.628876 | Significant | Down mtub | DNAH1 |
| 2864 | A0A5F9D0P9 | #N/A | #N/A | RABBIT | #N/A | #N/A | #N/A | #N/A | 0.49015 | #N/A | -0.50353 | #N/A | #N/A | #N/A | -0.99368 | #N/A | -0.94519 | #N/A | 0.41575 | #N/A | 0.40833 | 0 | 2.93229 | 0.55262 | #N/A | 0.552617 | Not Significant | NS | #N/A |
| 793 | G1TA80 | GRIP1 associated protein 1 OS=Oryctolagus cunicu | GRIPAP1 | RABBIT | #N/A | #N/A | #N/A | 0.43715 | #N/A | #N/A | #N/A | -0.55915 | #N/A | #N/A | -0.9963 | #N/A | -0.94768 | #N/A | 0.41467 | #N/A | 0.40833 | 0 | 2.93229 | 0.55262 | #N/A | 0.552617 | Not Significant | NS | #N/A |
| 1563 | G1U5N5 | Metallo-beta-lactamase domain-containing protein | #N/A | RABBIT | #N/A | 0.59757 | #N/A | #N/A | #N/A | #N/A | #N/A | #N/A | -0.39975 | #N/A | -0.99733 | #N/A | -0.94866 | #N/A | 0.41424 | #N/A | 0.40833 | 0 | 2.93229 | 0.55262 | #N/A | 0.552617 | Not Significant | NS | #N/A |
| 183 | G1SS79 | Myristoylated alanine-rich C-kinase substrate OS=C | MARCKS | RABBIT | 0.2254 | 0.91041 | -0.22258 | 0.15064 | 0.04325 | -2.27195 | -0.0479 | -0.37937 | -1.2289 | 0.0479 | -0.99747 | -2.09869 | -2.10476 | 0.06908 | 0.05926 | 0.16349 | 0.16154 | 8 | 2.93229 | 0.55262 | 0.56473 | 0.561484 | Not Significant | NS | #N/A |
| 1386 | A0A5F9CI79 | Microtubule crosslinking factor 1 OS=Oryctolagus c | MTCL1 | RABBIT | -0.18882 | -0.36541 | 2.95923 | 0.71866 | 0.1871 | #N/A | #N/A | #N/A | -0.33976 | #N/A | -1.00191 | -0.67771 | -0.80694 | 0.53513 | 0.4465 | 0.42838 | 0.4199 | 4 | 2.93229 | 0.55262 | 1.82137 | 1.284698 | Not Significant | NS | #N/A |
| 646 | G1U5C4 | Cell adhesion molecule 4 OS=Oryctolagus cuniculu | CADM4 | RABBIT | 1.14268 | 2.17496 | 0.17065 | 0.73783 | -0.02256 | #N/A | -0.29953 | 0.08572 | -0.0994 | -0.33665 | -1.00318 | -2.21495 | -2.14864 | 0.06234 | 0.05738 | 0.15764 | 0.15918 | 7 | 2.93229 | 0.55262 | 0.45584 | 0.484413 | Not Significant | NS | #N/A |
| 1454 | A0A5F9CU97 | Phosphatidylinositol-4-phosphate 3-kinase catalyti | PIK3C2B | RABBIT | 0.50771 | #N/A | #N/A | #N/A | #N/A | #N/A | #N/A | #N/A | -0.49605 | #N/A | -1.00377 | #N/A | -0.95479 | #N/A | 0.41159 | #N/A | 0.40698 | 0 | 2.93229 | 0.55262 | #N/A | 0.552617 | Not Significant | NS | #N/A |
| 2395 | A0A5F9CF50 | #N/A | CMTM5 | RABBIT | #N/A | #N/A | #N/A | #N/A | 0.91188 | #N/A | #N/A | #N/A | 0 | -0.18383 | -1.0038 | -6.30513 | -1.27015 | 0.10013 | 0.27398 | 0.1899 | 0.33128 | 1 | 2.93229 | 0.55262 | 0.0169 | 0.416381 | Not Significant | NS | #N/A |
| 1693 | G1SHS4 | #N/A | CUL1 | RABBIT | #N/A | 0.79189 | #N/A | 0.52657 | #N/A | #N/A | -0.37301 | -0.04992 | -0.01444 | -0.94113 | -1.00386 | -3.02412 | -2.05385 | 0.03901 | 0.07947 | 0.1391 | 0.18311 | 4 | 2.93229 | 0.55262 | 0.14692 | 0.318527 | Not Significant | NS | #N/A |
| 2029 | G1SZ83 | #N/A | NELFCD | RABBIT | #N/A | 0.91224 | #N/A | #N/A | #N/A | #N/A | #N/A | -0.06437 | #N/A | -0.12043 | -1.00464 | -20.6925 | -1.27721 | 0.03074 | 0.27173 | 0.12916 | 0.33018 | 1 | 2.93229 | 0.55262 | 0.00157 | 0.412483 | Not Significant | NS | #N/A |
| 2117 | G1U031 | #N/A | #N/A | RABBIT | #N/A | #N/A | 2.61137 | #N/A | #N/A | -2.44072 | #N/A | #N/A | #N/A | #N/A | -5.0521 | #N/A | -4.80557 | #N/A | 0.01807 | #N/A | 0.10478 | 0 | 2.93229 | 0.55262 | #N/A | 0.552617 | Significant | Down mtub | #N/A |
| 157 | G1T0Y9 | Dynactin subunit 2 OS=Oryctolagus cuniculus OX=! | DCTN2 | RABBIT | 0.4107 | #N/A | #N/A | #N/A | 0.64696 | #N/A | #N/A | #N/A | -0.39904 | -0.56988 | -1.01329 | -6.95099 | -1.7451 | 0.02008 | 0.14222 | 0.11473 | 0.24166 | 2 | 2.93229 | 0.55262 | 0.02125 | 0.337152 | Not Significant | NS | #N/A |
| 282 | G1T0R9 | Glutathione S-transferase OS=Oryctolagus cuniculu | GSTM3 | RABBIT | 0.11519 | 3.34179 | -0.0507 | -0.21596 | -0.11691 | -3.34892 | 0.25082 | 0.75798 | -0.21442 | 0.53802 | -1.01798 | -0.99991 | -1.12572 | 0.34663 | 0.28439 | 0.34582 | 0.33721 | 8 | 2.93229 | 0.55262 | 2.59118 | 2.044389 | Not Significant | NS | #N/A |
| 2167 | G1TH86 | #N/A | RAB11B | RABBIT | #N/A | #N/A | 4.34521 | 4.93055 | #N/A | -0.74501 | #N/A | -0.75663 | -0.64384 | 0.64384 | -5.01329 | -9.25721 | -8.54088 | 0.00076 | 6.3E-05 | 0.04295 | 0.01186 | 4 | 2.93229 | 0.55262 | 0.39104 | 0.459387 | Significant | Down mtub | RAB11B |
| 475 | G1SL60 | Splicing factor 3B subunit 3 OS=Oryctolagus cunicu | SF3B3 | RABBIT | -0.87434 | -0.24831 | 0.95104 | 0.5754 | 0.91662 | -3.5292 | 0.65795 | 0.38176 | -0.54223 | -0.74542 | -1.01951 | -1.2371 | -1.36691 | 0.25113 | 0.1991 | 0.29976 | 0.28306 | 8 | 2.93229 | 0.55262 | 1.69793 | 1.390728 | Not Significant | NS | #N/A |
| 1116 | G1SST9 | UV excision repair protein RAD23 OS=Oryctolagus c | RAD23B | RABBIT | #N/A | 2.37314 | #N/A | 2.19846 | #N/A | #N/A | -2.96877 | -2.23968 | #N/A | #N/A | -4.89002 | -13.0449 | -7.87572 | 0.00583 | 0.00056 | 0.07337 | 0.02813 | 2 | 2.93229 | 0.55262 | 0.14052 | 0.385515 | Significant | Down mtub | RAD23B |
| 2444 | A0A5F9CFK5 | #N/A | #N/A | RABBIT | #N/A | #N/A | 0.39175 | #N/A | -0.01338 | #N/A | #N/A | -0.83137 | #N/A | #N/A | -1.02055 | -2.90872 | -1.26639 | 0.21081 | 0.27519 | 0.27497 | 0.33157 | 1 | 2.93229 | 0.55262 | 0.08207 | 0.432954 | Not Significant | NS | #N/A |
| 237 | G1SJJ2 | Actin related protein 10 OS=Oryctolagus cuniculus | ACTR10 | RABBIT | 0.57626 | -0.13985 | 0.28708 | 0.03983 | 0.7226 | -2.48309 | -0.04753 | -0.01684 | -0.16882 | -0.9079 | -1.02202 | -2.06263 | -2.09076 | 0.07306 | 0.06071 | 0.16663 | 0.16242 | 8 | 2.93229 | 0.55262 | 0.61379 | 0.597381 | Not Significant | NS | #N/A |
| 2174 | P20647 | #N/A | ATP2A2 | RABBIT | #N/A | #N/A | 0.56104 | #N/A | #N/A | #N/A | #N/A | -1.39172 | 0.38853 | -0.38853 | -1.02494 | -0.99452 | -1.09966 | 0.42476 | 0.32223 | 0.38058 | 0.35646 | 2 | 2.93229 | 0.55262 | 0.79658 | 0.651543 | Not Significant | NS | #N/A |
| 1619 | A0A5F9CJ98 | Secretory carrier-associated membrane protein OS | SCAMP1 | RABBIT | -1.5354 | #N/A | 1.24851 | #N/A | 0.60047 | 0 | #N/A | 0.77719 | -1.07674 | -3.39262 | -1.02757 | -0.80091 | -0.95541 | 0.45953 | 0.36758 | 0.39628 | 0.38387 | 5 | 2.93229 | 0.55262 | 2.82185 | 1.982995 | Not Significant | NS | #N/A |
| 2380 | A0A5F9CLQ4 | #N/A | RBFOX2 | RABBIT | #N/A | #N/A | 0.17065 | 1.57141 | #N/A | -0.15787 | #N/A | #N/A | #N/A | #N/A | -1.0289 | -0.84817 | -1.03286 | 0.55218 | 0.36096 | 0.43315 | 0.38038 | 1 | 2.93229 | 0.55262 | 0.98106 | 0.661573 | Not Significant | NS | #N/A |
| 757 | G1SCL6 | Cytospin-A OS=Oryctolagus cuniculus OX=9986 GI | SPECC1L | RABBIT | 0.01166 | #N/A | #N/A | #N/A | 0.12843 | #N/A | #N/A | -4.77275 | #N/A | #N/A | -4.8428 | -47.8883 | -6.14676 | 0.01329 | 0.00376 | 0.09752 | 0.05982 | 1 | 2.93229 | 0.55262 | 0.00682 | 0.413817 | Significant | Down mtub | SPECC1L |
| 1870 | A0A5F9DUQ3 | #N/A | LCORL | RABBIT | #N/A | 0.58187 | #N/A | #N/A | #N/A | #N/A | #N/A | -0.44841 | #N/A | #N/A | -1.03028 | #N/A | -0.98001 | #N/A | 0.40086 | #N/A | 0.40169 | 0 | 2.93229 | 0.55262 | #N/A | 0.552617 | Not Significant | NS | #N/A |
| 1643 | G1TP34 | Protein kinase domain-containing protein OS=Oryct | #N/A | RABBIT | -1.13557 | 0.32982 | 1.28545 | #N/A | 0.48887 | -1.08777 | #N/A | 0.61878 | -2.55168 | -0.132 | -1.03031 | -1.21248 | -1.35786 | 0.2709 | 0.20782 | 0.30873 | 0.28797 | 6 | 2.93229 | 0.55262 | 1.44416 | 1.151483 | Not Significant | NS | #N/A |
| 2061 | G1T035 | #N/A | PRKCD | RABBIT | #N/A | 2.49287 | #N/A | #N/A | #N/A | #N/A | #N/A | #N/A | #N/A | -2.29505 | -4.78793 | #N/A | -4.55429 | #N/A | 0.02084 | #N/A | 0.10939 | 0 | 2.93229 | 0.55262 | #N/A | 0.552617 | Significant | Down mtub | PRKCD |
| 1829 | G1U6F6 | #N/A | GRIA1 | RABBIT | #N/A | 2.48358 | #N/A | #N/A | #N/A | #N/A | -2.28576 | #N/A | #N/A | #N/A | -4.76933 | #N/A | -4.5366 | #N/A | 0.02105 | #N/A | 0.10939 | 0 | 2.93229 | 0.55262 | #N/A | 0.552617 | Significant | Down mtub | GRIA1 |
| 2402 | A0A5F9CX20 | #N/A | GMPS | RABBIT | #N/A | #N/A | #N/A | 0.90019 | #N/A | -0.1618 | #N/A | -0.39982 | 0.1618 | #N/A | -1.03346 | -3.17495 | -1.49011 | 0.08652 | 0.19717 | 0.17578 | 0.28194 | 2 | 2.93229 | 0.55262 | 0.07947 | 0.360758 | Not Significant | NS | #N/A |
| 212 | G1T2V2 | PurE domain-containing protein OS=Oryctolagus cu | PAICS | RABBIT | 1.04055 | 0.51146 | -0.23169 | -0.05764 | 0.78802 | #N/A | 0.52853 | -0.63291 | -1.57673 | -0.82173 | -1.03585 | -2.19749 | -2.15983 | 0.06397 | 0.05631 | 0.15853 | 0.15773 | 7 | 2.93229 | 0.55262 | 0.49377 | 0.511145 | Not Significant | NS | #N/A |
| 22 | G1T652 | Fructose-bisphosphate aldolase OS=Oryctolagus c | ALDOC | RABBIT | 0.80321 | 1.56307 | -0.36759 | 0.03749 | 0.33683 | -2.5242 | 0.47429 | -0.23245 | -0.43087 | -0.09513 | -1.03627 | -1.69449 | -1.79585 | 0.12862 | 0.10018 | 0.21348 | 0.20018 | 8 | 2.93229 | 0.55262 | 0.935 | 0.832435 | Not Significant | NS | #N/A |
| 90 | A0A5F9DVP2 | Syntaxin-binding protein 1 OS=Oryctolagus cunicul | STXBP1 | RABBIT | -0.57639 | 1.09964 | -0.74384 | 0.22818 | 4.64569 | 0.13415 | -0.63846 | -0.19852 | 0.37203 | -0.22582 | -1.04198 | -1.04221 | -1.17177 | 0.32778 | 0.2662 | 0.33657 | 0.32835 | 8 | 2.93229 | 0.55262 | 2.49889 | 1.976856 | Not Significant | NS | #N/A |
| 2317 | G1SQQ7 | #N/A | TSNAXIP1 | RABBIT | #N/A | #N/A | 0.60681 | #N/A | #N/A | #N/A | #N/A | #N/A | #N/A | -0.43616 | -1.04297 | #N/A | -0.99208 | #N/A | 0.39582 | #N/A | 0.39935 | 0 | 2.93229 | 0.55262 | #N/A | 0.552617 | Not Significant | NS | #N/A |
| 1627 | G1TL77 | mitogen-activated protein kinase kinase OS=Orycto | MAP2K7 | RABBIT | 0.69217 | #N/A | #N/A | #N/A | #N/A | #N/A | #N/A | #N/A | 0 | -0.70391 | -1.04412 | -1.71279 | -1.23686 | 0.33643 | 0.28487 | 0.33996 | 0.33729 | 1 | 2.93229 | 0.55262 | 0.24774 | 0.475086 | Not Significant | NS | #N/A |
| 2103 | G1SII2 | #N/A | STOX1 | RABBIT | #N/A | 0.19782 | #N/A | 2.37794 | #N/A | #N/A | #N/A | -3.48123 | #N/A | #N/A | -4.76911 | -2.52596 | -3.86236 | 0.23998 | 0.0187 | 0.29437 | 0.106 | 1 | 2.93229 | 0.55262 | 2.37646 | 1.01643 | Significant | Down mtub | STOX1 |
| 1642 | G1TAQ4 | Anaphase-promoting complex subunit 4 OS=Oryctc | ANAPC4 | RABBIT | 1.81261 | #N/A | #N/A | #N/A | 0.02781 | #N/A | #N/A | -0.10556 | -0.14683 | #N/A | -1.0464 | -1.17226 | -1.29627 | 0.36183 | 0.25222 | 0.35245 | 0.3189 | 2 | 2.93229 | 0.55262 | 0.7968 | 0.651633 | Not Significant | NS | #N/A |
| 1068 | G1SN33 | Structural maintenance of chromosomes protein 1/ | #N/A | RABBIT | 4.61288 | #N/A | #N/A | #N/A | #N/A | #N/A | 0.21227 | -0.10834 | 0 | -0.54034 | -4.72198 | -13.3302 | -7.4208 | 0.00091 | 0.00032 | 0.04345 | 0.02295 | 3 | 2.93229 | 0.55262 | 0.10038 | 0.32392 | Significant | Down mtub | #N/A |
| 1855 | G1SZC0 | #N/A | #N/A | RABBIT | #N/A | 0.72705 | -0.35858 | 0.48041 | #N/A | #N/A | -0.768 | #N/A | #N/A | #N/A | -1.05096 | -1.59926 | -1.34215 | 0.25088 | 0.23801 | 0.29971 | 0.30799 | 2 | 2.93229 | 0.55262 | 0.32389 | 0.459871 | Not Significant | NS | #N/A |
| 248 | A0A5F9DQ03 | Calcium/calmodulin dependent serine protein kinas | CASK | RABBIT | 1.01298 | #N/A | -0.11081 | 0.24101 | 0.41682 | -1.86876 | -0.08351 | -0.06437 | 0.01549 | -1.33589 | -1.05741 | -2.16145 | -2.14909 | 0.06746 | 0.05733 | 0.1625 | 0.15918 | 7 | 2.93229 | 0.55262 | 0.53184 | 0.537975 | Not Significant | NS | #N/A |
| 1486 | G1SKE3 | Keratin 77 OS=Oryctolagus cuniculus OX=9986 GN | KRT77 | RABBIT | #N/A | 1.35619 | -0.15103 | -0.76684 | 1.41354 | 0.80182 | -2.63567 | -2.2268 | 0 | 1.08197 | -1.0587 | -1.05939 | -1.20085 | 0.3246 | 0.25767 | 0.33479 | 0.32317 | 7 | 2.93229 | 0.55262 | 2.21933 | 1.727268 | Not Significant | NS | #N/A |
| 2467 | G1SVG5 | #N/A | ERI3 | RABBIT | #N/A | #N/A | 0.61575 | #N/A | #N/A | -0.44509 | #N/A | #N/A | #N/A | #N/A | -1.06084 | #N/A | -1.00907 | #N/A | 0.38882 | #N/A | 0.39446 | 0 | 2.93229 | 0.55262 | #N/A | 0.552617 | Not Significant | NS | #N/A |
| 845 | A0A5F9C4F9 | Geranylgeranyl transferase type-2 subunit beta OS= | RABGGTB | RABBIT | 1.87376 | 0.46008 | #N/A | 0.08334 | -0.466 | #N/A | -0.84473 | #N/A | 0 | -0.87642 | -1.06151 | -1.66375 | -1.7317 | 0.15705 | 0.12189 | 0.23659 | 0.22013 | 5 | 2.93229 | 0.55262 | 0.69784 | 0.644155 | Not Significant | NS | #N/A |
| 325 | G1TI39 | Radixin OS=Oryctolagus cuniculus OX=9986 GN=RI | RDX | RABBIT | 0.01166 | 1.24732 | #N/A | 2.13254 | #N/A | #N/A | -2.26602 | -4.82888 | #N/A | #N/A | -4.67796 | -3.76633 | -4.65988 | 0.03275 | 0.00357 | 0.132 | 0.05832 | 3 | 2.93229 | 0.55262 | 1.85122 | 1.20933 | Significant | Down mtub | RDX |
| 1189 | P00919 | Carbonic anhydrase 2 OS=Oryctolagus cuniculus O | CA2 | RABBIT | 0.95302 | 4.89357 | -0.55579 | -1.48312 | #N/A | -0.74799 | 0.03793 | #N/A | -0.03793 | 0.2942 | -1.06536 | -0.7481 | -0.88383 | 0.48268 | 0.39995 | 0.40677 | 0.40151 | 6 | 2.93229 | 0.55262 | 4.05611 | 2.905982 | Not Significant | NS | #N/A |
| 765 | A0A5F9CTR2 | ArfGAP with GTPase domain, ankyrin repeat and PH | AGAP3 | RABBIT | 0.06804 | 0.14144 | #N/A | #N/A | 0.53749 | #N/A | -4.24626 | #N/A | #N/A | #N/A | -4.49526 | -15.4161 | -6.53946 | 0.00418 | 0.00132 | 0.06755 | 0.04508 | 2 | 2.93229 | 0.55262 | 0.06377 | 0.354394 | Significant | Down mtub | AGAP3 |
| 2210 | G1SEF8 | #N/A | NAPA | RABBIT | #N/A | #N/A | #N/A | #N/A | 0.49538 | #N/A | #N/A | -0.57312 | #N/A | #N/A | -1.0685 | #N/A | -1.01636 | #N/A | 0.38585 | #N/A | 0.39334 | 0 | 2.93229 | 0.55262 | #N/A | 0.552617 | Not Significant | NS | #N/A |
| 1669 | A0A5F9D8K0 | #N/A | KRT10 | RABBIT | #N/A | 1.79841 | -1.00141 | -0.05764 | 0.98332 | 1.27008 | -2.7592 | -2.05785 | -0.19169 | 0.53608 | -1.07119 | -1.04969 | -1.19216 | 0.32874 | 0.2609 | 0.33706 | 0.32441 | 7 | 2.93229 | 0.55262 | 2.31418 | 1.794119 | Not Significant | NS | #N/A |
| 2863 | G1T0S7 | #N/A | #N/A | RABBIT | #N/A | #N/A | #N/A | #N/A | 0.53022 | #N/A | -0.5436 | #N/A | #N/A | #N/A | -1.07382 | #N/A | -1.02142 | #N/A | 0.38381 | #N/A | 0.39263 | 0 | 2.93229 | 0.55262 | #N/A | 0.552617 | Not Significant | NS | #N/A |
| 650 | G1SI86 | Protein S100 OS=Oryctolagus cuniculus OX=9986 ( | S100A1 | RABBIT | 0.79018 | 2.47731 | 0.05201 | 0.99515 | -0.11583 | -1.94179 | -0.0063 | -0.1259 | 0.89378 | 0.0063 | -1.07454 | -1.64329 | -1.76135 | 0.13895 | 0.10608 | 0.22123 | 0.20505 | 8 | 2.93229 | 0.55262 | 1.06895 | 0.930459 | Not Significant | NS | #N/A |
| 2828 | G1SZG4 | #N/A | #N/A | RABBIT | #N/A | #N/A | #N/A | #N/A | 0.49967 | #N/A | #N/A | -0.57742 | #N/A | #N/A | -1.07708 | #N/A | -1.02452 | #N/A | 0.38256 | #N/A | 0.39182 | 0 | 2.93229 | 0.55262 | #N/A | 0.552617 | Not Significant | NS | #N/A |
| 1576 | A0A5F9CA83 | Mediator of RNA polymerase II transcription subunit | MED13 | RABBIT | 0.84912 | 1.88377 | #N/A | #N/A | #N/A | -5.40444 | -0.83746 | #N/A | #N/A | #N/A | -4.4874 | -1.91658 | -2.80934 | 0.19534 | 0.03815 | 0.2657 | 0.12996 | 2 | 2.93229 | 0.55262 | 5.48193 | 2.551413 | Significant | Down mtub | MED13 |
| 2775 | G1SX44 | #N/A | #N/A | RABBIT | #N/A | #N/A | #N/A | 2.15038 | #N/A | #N/A | #N/A | -2.27239 | #N/A | #N/A | -4.42277 | #N/A | -4.20695 | #N/A | 0.02565 | #N/A | 0.11574 | 0 | 2.93229 | 0.55262 | #N/A | 0.552617 | Significant | Down mtub | #N/A |
| 1796 | A0A5F9CF91 | #N/A | #N/A | RABBIT | #N/A | -0.87327 | 0.74885 | 0.07794 | 0.66329 | #N/A | -1.65299 | -0.19995 | #N/A | #N/A | -1.08067 | -1.51048 | -1.5752 | 0.20544 | 0.15963 | 0.27238 | 0.25503 | 4 | 2.93229 | 0.55262 | 0.68249 | 0.627556 | Not Significant | NS | #N/A |
| 1132 | G1STJ4 | NTR domain-containing protein OS=Oryctolagus cu | #N/A | RABBIT | -0.23093 | 1.09175 | #N/A | 0.18495 | 2.00927 | #N/A | 1.42643 | -0.6343 | -1.57094 | -0.48929 | -1.08079 | -1.34699 | -1.49425 | 0.22663 | 0.16957 | 0.2869 | 0.26263 | 6 | 2.93229 | 0.55262 | 1.28759 | 1.046316 | Not Significant | NS | #N/A |
| 1442 | G1TB74 | BCL2 like 13 OS=Oryctolagus cuniculus OX=9986 G | BCL2L13 | RABBIT | 0.7161 | 2.45812 | 0.4068 | 0.06194 | -0.13296 | #N/A | -0.13324 | -0.39935 | #N/A | -0.60589 | -1.08149 | -1.73119 | -1.80525 | 0.13413 | 0.10477 | 0.21787 | 0.2039 | 6 | 2.93229 | 0.55262 | 0.73174 | 0.672937 | Not Significant | NS | #N/A |
| 1059 | G1SX73 | Prefoldin subunit 1 OS=Oryctolagus cuniculus OX=! | PFDN1 | RABBIT | -0.6774 | -0.53998 | 0.17065 | 1.15778 | 0.87529 | #N/A | -1.78312 | 0.73815 | 0.93233 | -3.42498 | -1.08167 | -1.07033 | -1.21452 | 0.31998 | 0.25264 | 0.33396 | 0.31897 | 7 | 2.93229 | 0.55262 | 2.26957 | 1.762677 | Not Significant | NS | #N/A |
| 633 | O77819 | Rho-associated protein kinase 1 OS=Oryctolagus cu | ROCK1 | RABBIT | 0.30727 | #N/A | 0.48868 | -0.74623 | 0.52701 | -2.19769 | #N/A | 0.06593 | -0.1303 | -1.48812 | -1.08173 | -1.74065 | -1.82813 | 0.13239 | 0.10105 | 0.21631 | 0.20137 | 6 | 2.93229 | 0.55262 | 0.7724 | 0.700248 | Not Significant | NS | #N/A |
| 2135 | Q9TT37 | #N/A | ATP1B1 | RABBIT | #N/A | #N/A | 0.72515 | #N/A | 0.8241 | 0 | -1.25286 | 0.53674 | -0.19082 | -0.64583 | -1.08518 | -2.14164 | -1.96548 | 0.08514 | 0.08524 | 0.17453 | 0.1881 | 5 | 2.93229 | 0.55262 | 0.36679 | 0.435482 | Not Significant | NS | #N/A |
| 931 | A0A5F9C549 | Phosphoinositide phospholipase C OS=Oryctolagus | PLCG1 | RABBIT | 1.41147 | #N/A | 0.4846 | -0.47541 | 1.17375 | -0.31395 | -0.56618 | #N/A | #N/A | #N/A | -1.08867 | -1.70287 | -1.69783 | 0.1638 | 0.13377 | 0.24341 | 0.23331 | 4 | 2.93229 | 0.55262 | 0.54496 | 0.548201 | Not Significant | NS | #N/A |
| 299 | G1U7U3 | Nucleoside diphosphate kinase OS=Oryctolagus cu | NME1 | RABBIT | 1.11962 | 2.48876 | -0.00192 | 1.19148 | -2.36712 | -2.56655 | 0.45427 | -0.35776 | -0.73406 | 0.17257 | -1.09247 | -1.1226 | -1.25954 | 0.29417 | 0.23406 | 0.32123 | 0.3048 | 8 | 2.93229 | 0.55262 | 2.36759 | 1.880772 | Not Significant | NS | #N/A |
| 1033 | U3KNC6 | Glycylpeptide N-tetradecanoyltransferase OS=Oryc | NMT2 | RABBIT | 0.20389 | 0.5966 | #N/A | 0.18735 | #N/A | -0.19223 | #N/A | #N/A | -0.3266 | -1.77414 | -1.0936 | -2.08798 | -1.95124 | 0.10507 | 0.0924 | 0.19468 | 0.19439 | 4 | 2.93229 | 0.55262 | 0.41149 | 0.471185 | Not Significant | NS | #N/A |
| 1 | G1T346 | Spectrin alpha, non-erythrocytic 1 OS=Oryctolagus | SPTAN1 | RABBIT | 0.48421 | -0.28796 | 0.44533 | -0.20723 | 0.89186 | -3.87435 | 0.27856 | 0.08523 | -0.3433 | -0.28961 | -1.09394 | -1.36392 | -1.50259 | 0.20973 | 0.16127 | 0.27454 | 0.25578 | 8 | 2.93229 | 0.55262 | 1.60823 | 1.325087 | Not Significant | NS | #N/A |
| 335 | A0A5F9DJ34 | Thioredoxin reductase 1, cytoplasmic OS=Oryctola | TXNRD1 | RABBIT | 1.55906 | #N/A | -3.7928 | 1.21879 | 0.3386 | #N/A | 0.46879 | -3.328 | -1.84129 | -0.35198 | -1.09403 | -0.73304 | -0.86846 | 0.49117 | 0.40787 | 0.41059 | 0.40587 | 6 | 2.93229 | 0.55262 | 4.4549 | 3.173863 | Not Significant | NS | #N/A |
| 2201 | A0A5F9C697 | #N/A | TANGO2 | RABBIT | #N/A | #N/A | #N/A | #N/A | -0.01338 | #N/A | -2.4924 | 0.2745 | #N/A | #N/A | -1.09557 | -0.45721 | -0.75995 | 0.727 | 0.49029 | 0.49145 | 0.43948 | 1 | 2.93229 | 0.55262 | 3.82789 | 1.385534 | Not Significant | NS | #N/A |
| 503 | G1T9S9 | Tubulin alpha chain OS=Oryctolagus cuniculus OX= | TUBAL3 | RABBIT | 2.78826 | #N/A | #N/A | #N/A | -0.74095 | #N/A | 0.72757 | #N/A | #N/A | -0.87487 | -1.0973 | -0.56621 | -0.80643 | 0.62831 | 0.4571 | 0.46044 | 0.42409 | 2 | 2.93229 | 0.55262 | 3.75578 | 1.85147 | Not Significant | NS | #N/A |
| 163 | G1SF47 | Septin OS=Oryctolagus cuniculus OX=9986 GN=SE | SEPTIN11 | RABBIT | 0.3259 | -0.08471 | 0.29482 | -0.18181 | 0.54628 | -0.54824 | 0.63062 | 0.27415 | -4.68179 | -0.26128 | -1.09741 | -1.1282 | -1.26577 | 0.29193 | 0.23191 | 0.32071 | 0.30329 | 8 | 2.93229 | 0.55262 | 2.3654 | 1.87917 | Not Significant | NS | #N/A |

| | | | | | | | | | | | | | | | | | | | | | | | | | | | | | |
|---|---|---|---|---|---|---|---|---|---|---|---|---|---|---|---|---|---|---|---|---|---|---|---|---|---|---|---|---|---|
| 2668 | A0A5F9CCB6 | #N/A | UBP1 | RABBIT | #N/A | #N/A | #N/A | 0.49019 | #N/A | #N/A | #N/A | -0.61219 | #N/A | #N/A | -1.10238 | #N/A | -1.04859 | #N/A | 0.373 | #N/A | 0.3868 | 0 | 2.93229 | 0.55262 | #N/A | 0.552617 | Not Significant | NS | #N/A |
| 1166 | G1SPG2 | Thyroid hormone receptor interactor 11 OS=Oryctol | TRIP11 | RABBIT | -1.56286 | 0.26639 | 1.03261 | #N/A | 1.33337 | #N/A | -1.15918 | #N/A | 0 | -1.35308 | -1.1048 | -1.30509 | -1.46222 | 0.24869 | 0.18214 | 0.29904 | 0.27202 | 5 | 2.93229 | 0.55262 | 1.22848 | 0.978637 | Not Significant | NS | #N/A |
| 2043 | A0A5F9DPR8 | #N/A | #N/A | RABBIT | #N/A | 0.19782 | #N/A | 0.43702 | #N/A | #N/A | #N/A | #N/A | -0.79038 | #N/A | -1.1078 | -5.34766 | -1.39676 | 0.11769 | 0.23616 | 0.20536 | 0.30628 | 1 | 2.93229 | 0.55262 | 0.02861 | 0.419359 | Not Significant | NS | #N/A |
| 880 | A0A5F9DT15 | Nuclear receptor coactivator 7 OS=Oryctolagus cur | NCOA7 | RABBIT | 2.21902 | #N/A | #N/A | -1.57721 | 0.66071 | #N/A | #N/A | #N/A | -0.6741 | #N/A | -1.10827 | -0.50298 | -0.71438 | 0.6649 | 0.50736 | 0.47235 | 0.44748 | 2 | 2.93229 | 0.55262 | 3.64132 | 1.80506 | Not Significant | NS | #N/A |
| 1686 | A0A5F9DVI3 | #N/A | #N/A | RABBIT | #N/A | 0.43585 | #N/A | 0.7899 | #N/A | -0.75485 | #N/A | #N/A | -0.23803 | #N/A | -1.10932 | -3.54157 | -1.82787 | 0.07131 | 0.12792 | 0.16445 | 0.22682 | 2 | 2.93229 | 0.55262 | 0.09811 | 0.368319 | Not Significant | NS | #N/A |
| 964 | G1SWM1 | Small ribosomal subunit protein uS5 OS=Oryctolag | RPS2 | RABBIT | 0.32017 | 1.12987 | #N/A | -0.36614 | #N/A | -0.74824 | #N/A | #N/A | #N/A | #N/A | -1.10953 | -1.28314 | -1.28873 | 0.32805 | 0.25462 | 0.33661 | 0.32053 | 2 | 2.93229 | 0.55262 | 0.56078 | 0.555926 | Not Significant | NS | #N/A |
| 1876 | A0A5F9C2C1 | #N/A | ARAP2 | RABBIT | #N/A | 2.30038 | #N/A | #N/A | #N/A | #N/A | #N/A | #N/A | #N/A | -2.10256 | -4.40294 | #N/A | -4.18809 | #N/A | 0.02595 | #N/A | 0.11679 | 0 | 2.93229 | 0.55262 | #N/A | 0.552617 | Significant | Down mtub | ARAP2 |
| 2865 | G1SYV5 | #N/A | ABCC6 | RABBIT | #N/A | #N/A | #N/A | #N/A | 0.54892 | #N/A | #N/A | #N/A | -0.5623 | #N/A | -1.11123 | #N/A | -1.057 | #N/A | 0.36971 | #N/A | 0.38394 | 0 | 2.93229 | 0.55262 | #N/A | 0.552617 | Not Significant | NS | #N/A |
| 1340 | G1TZI2 | Serine/threonine-protein phosphatase PGAM5, mitc | PGAM5 | RABBIT | 0.79571 | 1.00152 | #N/A | -0.05764 | #N/A | -3.61508 | #N/A | #N/A | #N/A | -3.99284 | -4.38383 | -9.92638 | -7.67485 | 0.00217 | 0.00027 | 0.05406 | 0.02295 | 3 | 2.93229 | 0.55262 | 0.23405 | 0.391515 | Significant | Down mtub | PGAM5 |
| 1297 | G1TG30 | Creatine kinase M-type OS=Oryctolagus cuniculus ( | CKM | RABBIT | 0.01166 | 0.25406 | 0.41067 | -0.61075 | #N/A | -1.09797 | #N/A | #N/A | #N/A | #N/A | -1.11438 | -2.21905 | -1.62726 | 0.11314 | 0.15537 | 0.20012 | 0.25326 | 3 | 2.93229 | 0.55262 | 0.20176 | 0.375184 | Not Significant | NS | #N/A |
| 2244 | A0A5F9D7S9 | #N/A | NFIB | RABBIT | #N/A | #N/A | -0.00037 | #N/A | 2.22942 | #N/A | #N/A | #N/A | #N/A | 0 | -1.11452 | -0.57716 | -0.8905 | 0.66676 | 0.42433 | 0.47235 | 0.40956 | 1 | 2.93229 | 0.55262 | 2.48597 | 1.044278 | Not Significant | NS | #N/A |
| 1052 | Q9TQQ8 | Class MU glutathione S-transferase isozyme R11, cl | #N/A | RABBIT | 1.3921 | 2.37958 | #N/A | -0.05764 | -0.36158 | #N/A | 0.27553 | #N/A | -1.0065 | -0.1008 | -1.11537 | -1.35671 | -1.51058 | 0.23291 | 0.16966 | 0.29083 | 0.26263 | 5 | 2.93229 | 0.55262 | 1.15863 | 0.934611 | Not Significant | NS | #N/A |
| 893 | A0A5F9CAK9 | CCHC-type zinc finger nucleic acid binding protein ( | #N/A | RABBIT | 0.8478 | -0.39249 | 0.34563 | 0.75536 | 0.04697 | 0.23785 | -0.27155 | -0.12472 | -1.51744 | -2.32305 | -1.12043 | -2.09918 | -2.16526 | 0.06903 | 0.05336 | 0.16349 | 0.153 | 8 | 2.93229 | 0.55262 | 0.71222 | 0.669411 | Not Significant | NS | #N/A |
| 1558 | G1SZF6 | Putative malate dehydrogenase 1B OS=Oryctolagus | MDH1B | RABBIT | 0.65998 | #N/A | #N/A | -0.05764 | #N/A | #N/A | #N/A | #N/A | #N/A | -0.82539 | -1.12656 | -1.81272 | -1.33105 | 0.32093 | 0.2551 | 0.33441 | 0.3209 | 1 | 2.93229 | 0.55262 | 0.25749 | 0.477564 | Not Significant | NS | #N/A |
| 1690 | A0A5F9DDV8 | #N/A | KPNA4 | RABBIT | #N/A | 0.62412 | -0.25565 | #N/A | 0.96433 | -3.93074 | #N/A | #N/A | #N/A | #N/A | -4.37501 | -6.01825 | -5.41681 | 0.02652 | 0.00303 | 0.12384 | 0.05579 | 2 | 2.93229 | 0.55262 | 0.39635 | 0.489252 | Significant | Down mtub | KPNA4 |
| 748 | A0A5F9CNX7 | RB1 inducible coiled-coil 1 OS=Oryctolagus cunicul | RB1CC1 | RABBIT | 0.43196 | -1.20149 | 1.5233 | #N/A | 1.94005 | #N/A | -0.87178 | -0.49725 | 0 | #N/A | -1.1298 | -1.31963 | -1.48197 | 0.24415 | 0.17694 | 0.29685 | 0.26786 | 5 | 2.93229 | 0.55262 | 1.25655 | 0.996328 | Not Significant | NS | #N/A |
| 2109 | G1SN15 | #N/A | OSGEP | RABBIT | #N/A | 2.2319 | #N/A | #N/A | #N/A | -2.03408 | #N/A | #N/A | #N/A | #N/A | -4.26597 | #N/A | -4.0578 | #N/A | 0.02816 | #N/A | 0.11898 | 0 | 2.93229 | 0.55262 | #N/A | 0.552617 | Significant | Down mtub | OSGEP |
| 2820 | A0A5F9DJX9 | #N/A | MTPAP | RABBIT | #N/A | #N/A | #N/A | #N/A | 2.03689 | #N/A | #N/A | #N/A | #N/A | -2.05028 | -4.08717 | #N/A | -3.88772 | #N/A | 0.03143 | #N/A | 0.12426 | 0 | 2.93229 | 0.55262 | #N/A | 0.552617 | Significant | Down mtub | MTPAP |
| 2420 | G1TL88 | #N/A | #N/A | RABBIT | #N/A | #N/A | 2.11478 | #N/A | #N/A | -1.94413 | #N/A | #N/A | #N/A | #N/A | -4.05892 | #N/A | -3.86085 | #N/A | 0.03199 | #N/A | 0.12464 | 0 | 2.93229 | 0.55262 | #N/A | 0.552617 | Significant | Down mtub | #N/A |
| 1589 | G1SWD8 | 1-phosphatidylinositol 4-kinase OS=Oryctolagus cu | PI4KA | RABBIT | #N/A | #N/A | 3.72861 | -1.87356 | 2.21105 | -0.02841 | -0.55111 | -0.06437 | 1.48508 | #N/A | -1.14507 | -0.76653 | -0.92701 | 0.47797 | 0.38126 | 0.40468 | 0.39096 | 5 | 2.93229 | 0.55262 | 3.82554 | 2.615653 | Not Significant | NS | #N/A |
| 2734 | A0A5F9CJ56 | #N/A | OTOF | RABBIT | #N/A | #N/A | #N/A | 1.98092 | #N/A | #N/A | -2.03856 | #N/A | #N/A | #N/A | -4.01949 | #N/A | -3.82334 | #N/A | 0.03279 | #N/A | 0.12553 | 0 | 2.93229 | 0.55262 | #N/A | 0.552617 | Significant | Down mtub | OTOF |
| 2476 | G1T305 | #N/A | CASD1 | RABBIT | #N/A | #N/A | 0.62722 | #N/A | #N/A | #N/A | #N/A | -0.52093 | #N/A | #N/A | -1.14815 | #N/A | -1.09213 | #N/A | 0.35629 | #N/A | 0.37732 | 0 | 2.93229 | 0.55262 | #N/A | 0.552617 | Not Significant | NS | #N/A |
| 986 | A0A5F9DC28 | phosphoinositide 5-phosphatase OS=Oryctolagus ( | SYNJ2 | RABBIT | 0.01166 | #N/A | #N/A | #N/A | 0.32983 | #N/A | -0.97988 | #N/A | #N/A | #N/A | -1.15063 | -4.17583 | -1.44118 | 0.14964 | 0.22415 | 0.23045 | 0.29961 | 1 | 2.93229 | 0.55262 | 0.05062 | 0.424956 | Not Significant | NS | #N/A |
| 2019 | Q9Q8Q1 | #N/A | m045L | RABBIT | #N/A | 0.28524 | #N/A | -0.07039 | -0.00063 | #N/A | #N/A | -1.08049 | #N/A | #N/A | -1.1519 | -5.29374 | -1.70349 | 0.03388 | 0.15001 | 0.13313 | 0.24914 | 2 | 2.93229 | 0.55262 | 0.03551 | 0.342935 | Not Significant | NS | #N/A |
| 376 | G1TD99 | Propionyl-CoA carboxylase subunit alpha OS=Oryct | PCCA | RABBIT | 1.25764 | 1.0815 | 0.17065 | -0.5672 | 1.33007 | #N/A | -0.34088 | 0.55439 | -1.45037 | -0.76355 | -1.15464 | -2.07022 | -2.13438 | 0.07719 | 0.05877 | 0.17093 | 0.16071 | 7 | 2.93229 | 0.55262 | 0.69127 | 0.650335 | Not Significant | NS | #N/A |
| 398 | G1SX47 | Spermatid perinuclear RNA-binding protein OS=Ory | STRBP | RABBIT | -1.22562 | 0.25898 | 0.41486 | 0.58354 | 0.85156 | -2.67998 | -1.73977 | 0.09865 | -0.519 | -0.06116 | -1.15692 | -1.79174 | -1.91656 | 0.11094 | 0.08179 | 0.1981 | 0.18476 | 8 | 2.93229 | 0.55262 | 1.0423 | 0.910957 | Not Significant | NS | #N/A |
| 2134 | A0A5F9CV40 | #N/A | RDX | RABBIT | #N/A | #N/A | 2.78464 | #N/A | 1.64866 | #N/A | #N/A | #N/A | -1.66204 | -1.91246 | -4.0039 | -6.88393 | -5.86712 | 0.02046 | 0.00214 | 0.11586 | 0.05019 | 2 | 2.93229 | 0.55262 | 0.33829 | 0.465711 | Significant | Down mtub | RDX |
| 954 | A0A5F9CXA1 | Heparin binding growth factor OS=Oryctolagus cuni | HDGF | RABBIT | -1.00778 | 2.24415 | #N/A | 0.28317 | #N/A | #N/A | 0.59361 | -2.20855 | #N/A | -0.34081 | -1.15843 | -0.92386 | -1.12355 | 0.40785 | 0.2986 | 0.3744 | 0.34592 | 4 | 2.93229 | 0.55262 | 2.3584 | 1.594573 | Not Significant | NS | #N/A |
| 1818 | G1T102 | #N/A | #N/A | RABBIT | #N/A | -0.30398 | #N/A | 0.79282 | 0.92885 | -2.06915 | #N/A | #N/A | 0.21814 | -0.21814 | -1.16228 | -1.44848 | -1.60052 | 0.22106 | 0.15393 | 0.28316 | 0.25312 | 4 | 2.93229 | 0.55262 | 0.9658 | 0.791027 | Not Significant | NS | #N/A |
| 572 | G1T369 | DCN1-like protein OS=Oryctolagus cuniculus OX=9 | DCUN1D1 | RABBIT | 1.29403 | 1.09593 | -1.05677 | 0.27884 | -0.05461 | -4.01424 | -0.11301 | -0.02314 | 0.49447 | -0.60123 | -1.16291 | -1.27276 | -1.42051 | 0.23884 | 0.18335 | 0.29367 | 0.27335 | 8 | 2.93229 | 0.55262 | 2.08709 | 1.675509 | Not Significant | NS | #N/A |
| 527 | A0A5F9D4P7 | RUN and FYVE domain containing 3 OS=Oryctolagu | RUFY3 | RABBIT | -0.01566 | 0.4571 | 0.36082 | #N/A | -0.01338 | -0.9658 | #N/A | #N/A | #N/A | #N/A | -1.16302 | -4.20062 | -1.88615 | 0.02462 | 0.10878 | 0.11961 | 0.2077 | 3 | 2.93229 | 0.55262 | 0.06133 | 0.304167 | Not Significant | NS | #N/A |
| 723 | A0A5F9CGR4 | Nidogen 1 OS=Oryctolagus cuniculus OX=9986 PE= | #N/A | RABBIT | -0.39784 | #N/A | -0.35208 | 0.43335 | 1.1959 | -4.81966 | 1.40199 | -1.79718 | 0 | 0.4939 | -1.16403 | -0.90179 | -1.04211 | 0.39713 | 0.32206 | 0.36958 | 0.35646 | 7 | 2.93229 | 0.55262 | 3.70256 | 2.772607 | Not Significant | NS | #N/A |
| 97 | G1SQI4 | Neural cell adhesion molecule 1 OS=Oryctolagus c | NCAM1 | RABBIT | 0.58807 | #N/A | #N/A | #N/A | #N/A | #N/A | #N/A | #N/A | -0.57641 | #N/A | -1.16448 | #N/A | -1.10766 | #N/A | 0.35052 | #N/A | 0.3742 | 0 | 2.93229 | 0.55262 | #N/A | 0.552617 | Not Significant | NS | #N/A |
| 539 | B7NZU5 | Tyrosine 3-monooxygenase OS=Oryctolagus cunicu | TH | RABBIT | 2.14802 | 1.05931 | 1.23501 | -0.05764 | 1.16656 | -3.70013 | #N/A | -1.4898 | -2.36853 | -3.74171 | -3.93529 | -6.30518 | -6.67135 | 0.0004 | 5.7E-05 | 0.03578 | 0.01186 | 7 | 2.93229 | 0.55262 | 0.86566 | 0.77324 | Significant | Down mtub | TH |
| 111 | G1TBU8 | Rap1 GTPase-GDP dissociation stimulator 1 OS=Or | RAP1GDS1 | RABBIT | 0.48411 | 0.0831 | -0.52389 | 0.59962 | 0.10133 | -1.0229 | #N/A | #N/A | #N/A | #N/A | -1.17176 | -2.43051 | -1.81977 | 0.07194 | 0.11202 | 0.16462 | 0.21129 | 4 | 2.93229 | 0.55262 | 0.19369 | 0.34551 | Not Significant | NS | #N/A |
| 1330 | G1TBC0 | Spliceosome associated factor 3, U4/U6 recycling p | SART3 | RABBIT | #N/A | #N/A | 0.48771 | 3.11465 | -1.14275 | 0.41949 | -1.11019 | -0.38143 | #N/A | #N/A | -1.17725 | -0.89423 | -1.09499 | 0.42174 | 0.3101 | 0.3798 | 0.35209 | 4 | 2.93229 | 0.55262 | 2.59976 | 1.733841 | Not Significant | NS | #N/A |
| 2051 | B7NZR4 | #N/A | MPP1 | RABBIT | #N/A | 3.72916 | #N/A | #N/A | #N/A | 1.21362 | -0.31858 | -1.88525 | -0.23939 | 0.23939 | -3.92721 | -3.19157 | -3.65554 | 0.03317 | 0.00826 | 0.13286 | 0.08427 | 4 | 2.93229 | 0.55262 | 1.26176 | 0.961799 | Significant | Down mtub | MPP1 |
| 62 | U3KPF7 | protein-tyrosine-phosphatase OS=Oryctolagus cun | PTPRZ1 | RABBIT | 0.20437 | -0.4069 | 0.41553 | 0.29205 | 0.82749 | -3.16387 | 0.15293 | -0.2173 | -1.02261 | -0.31248 | -1.17918 | -1.88167 | -2.00274 | 0.09666 | 0.07063 | 0.1866 | 0.17326 | 8 | 2.93229 | 0.55262 | 0.98177 | 0.86666 | Not Significant | NS | #N/A |
| 262 | U3KMS5 | COP9 signalosome complex subunit 3 OS=Oryctola | COPS3 | RABBIT | 0.60118 | 0.29331 | 0.07516 | 0.18599 | 0.75446 | -3.01894 | 0.41821 | -0.34707 | -0.51085 | -0.53226 | -1.1802 | -1.98145 | -2.08991 | 0.08286 | 0.0608 | 0.17453 | 0.16242 | 8 | 2.93229 | 0.55262 | 0.88692 | 0.797254 | Not Significant | NS | #N/A |
| 2695 | G1TA52 | #N/A | #N/A | RABBIT | #N/A | #N/A | #N/A | 0.56159 | #N/A | -0.61923 | #N/A | #N/A | #N/A | #N/A | -1.18081 | #N/A | -1.12319 | #N/A | 0.34483 | #N/A | 0.37021 | 0 | 2.93229 | 0.55262 | #N/A | 0.552617 | Not Significant | NS | #N/A |
| 288 | G1T6Q8 | Ferritin OS=Oryctolagus cuniculus OX=9986 PE=3 S | #N/A | RABBIT | 0.79168 | 0.94573 | -0.02526 | -0.07673 | 0.77842 | -5.45779 | 2.33204 | -0.04527 | -0.13316 | -0.18997 | -1.1816 | -0.90881 | -1.03781 | 0.39001 | 0.32177 | 0.36581 | 0.35646 | 8 | 2.93229 | 0.55262 | 4.22603 | 3.240735 | Not Significant | NS | #N/A |
| 2118 | A0A5F9DDV5 | #N/A | DST | RABBIT | #N/A | #N/A | 0.16777 | #N/A | 1.19128 | #N/A | 0.00288 | #N/A | -1.0081 | #N/A | -1.18214 | -1.64343 | -1.61116 | 0.24201 | 0.16886 | 0.29614 | 0.26263 | 2 | 2.93229 | 0.55262 | 0.51741 | 0.53834 | Not Significant | NS | #N/A |
| 1145 | A5U318 | Polyketide synthase Pks7 OS=Mycobacterium tuber | #N/A | MT | 0.49361 | #N/A | 0.74014 | -0.05764 | #N/A | #N/A | -3.19709 | #N/A | #N/A | -3.65444 | -3.8178 | -10.9417 | -7.09932 | 0.00163 | 0.00041 | 0.05406 | 0.02526 | 3 | 2.93229 | 0.55262 | 0.14609 | 0.347036 | Significant | Down mtub | #N/A |
| 2552 | A0A5F9D139 | #N/A | KIAA1671 | RABBIT | #N/A | #N/A | #N/A | 0.53188 | #N/A | #N/A | #N/A | -0.65388 | #N/A | #N/A | -1.18576 | #N/A | -1.1279 | #N/A | 0.34312 | #N/A | 0.36928 | 0 | 2.93229 | 0.55262 | #N/A | 0.552617 | Not Significant | NS | #N/A |
| 135 | P25704 | Beta-enolase OS=Oryctolagus cuniculus OX=9986 ( | ENO3 | RABBIT | 1.69139 | 3.51213 | -2.7764 | 0.26426 | #N/A | #N/A | -0.3219 | -0.45849 | -1.79389 | 0.52094 | -1.18618 | -0.84029 | -0.9922 | 0.43295 | 0.34722 | 0.3832 | 0.37184 | 6 | 2.93229 | 0.55262 | 3.9854 | 2.858487 | Not Significant | NS | #N/A |
| 363 | A0A5F9CCM6 | Clathrin light chain OS=Oryctolagus cuniculus OX=! | CLTB | RABBIT | 0.3559 | -1.36733 | 4.55428 | -0.12176 | 1.25349 | -0.75153 | 0.05752 | 0.6629 | -0.05752 | -1.17077 | -1.1868 | -1.13111 | -1.27613 | 0.29078 | 0.22836 | 0.3201 | 0.30142 | 8 | 2.93229 | 0.55262 | 2.75222 | 2.162235 | Not Significant | NS | #N/A |
| 824 | G1TJR5 | HSPA (Hsp70) binding protein 1 OS=Oryctolagus cu | HSPBP1 | RABBIT | 0.48063 | 0.54282 | -0.30865 | 1.11683 | -0.01338 | #N/A | -0.69424 | 0.33454 | -2.45906 | -0.47384 | -1.1868 | -2.02136 | -2.11 | 0.08296 | 0.06123 | 0.17453 | 0.16275 | 7 | 2.93229 | 0.55262 | 0.76604 | 0.703033 | Not Significant | NS | #N/A |
| 1094 | A0A5F9CK42 | Formin binding protein 1 OS=Oryctolagus cuniculus | FNBP1 | RABBIT | 0.49875 | 1.73655 | -1.00593 | #N/A | 0.75104 | -1.98997 | #N/A | -0.09294 | #N/A | 0 | -1.18941 | -1.37838 | -1.55044 | 0.22657 | 0.15995 | 0.2869 | 0.25503 | 5 | 2.93229 | 0.55262 | 1.27645 | 1.008876 | Not Significant | NS | #N/A |
| 1536 | G1SJZ8 | coproporphyrinogen oxidase OS=Oryctolagus cunic | CPOX | RABBIT | #N/A | 0.89772 | #N/A | 3.4853 | -0.03803 | #N/A | -0.0894 | -0.67754 | 1.79243 | 0 | -1.19196 | -1.09922 | -1.28507 | 0.32176 | 0.23502 | 0.33441 | 0.30526 | 5 | 2.93229 | 0.55262 | 2.01573 | 1.474872 | Not Significant | NS | #N/A |
| 29 | A0A5F9C4N0 | Spectrin beta, non-erythrocytic 1 OS=Oryctolagus c | #N/A | RABBIT | 0.68122 | 0.01312 | 0.18388 | -0.07087 | 0.9413 | -3.21525 | 0.39487 | -0.04853 | -0.53545 | -0.81026 | -1.19266 | -1.81202 | -1.94422 | 0.10756 | 0.07804 | 0.1968 | 0.18205 | 8 | 2.93229 | 0.55262 | 1.08304 | 0.940766 | Not Significant | NS | #N/A |
| 2283 | G1TF42 | #N/A | NAA16 | RABBIT | #N/A | #N/A | 0.17065 | #N/A | 0.53452 | #N/A | #N/A | -0.84038 | #N/A | #N/A | -1.19297 | -3.78577 | -1.48729 | 0.16441 | 0.21234 | 0.24381 | 0.29139 | 1 | 2.93229 | 0.55262 | 0.0662 | 0.428919 | Not Significant | NS | #N/A |
| 2343 | A0A5F9CN32 | #N/A | #N/A | RABBIT | #N/A | #N/A | 0.17065 | #N/A | #N/A | #N/A | 0.46527 | #N/A | -2.51276 | #N/A | -1.1944 | -0.46312 | -0.78592 | 0.72389 | 0.47657 | 0.49085 | 0.43293 | 1 | 2.93229 | 0.55262 | 4.43432 | 1.539753 | Not Significant | NS | #N/A |
| 1986 | G1SIE2 | #N/A | TOP2A | RABBIT | #N/A | #N/A | #N/A | #N/A | 1.84221 | #N/A | #N/A | #N/A | #N/A | -1.8556 | -3.69781 | #N/A | -3.51737 | #N/A | 0.04042 | #N/A | 0.13328 | 0 | 2.93229 | 0.55262 | #N/A | 0.552617 | Significant | Down mtub | TOP2A |
| 749 | P06815 | Calpain-1 catalytic subunit (Fragment) OS=Oryctol: | CAPN1 | RABBIT | 1.46301 | 0.10858 | #N/A | 0.0316 | #N/A | -1.49045 | 0.32909 | #N/A | -0.83592 | #N/A | -1.20015 | -1.69862 | -1.80137 | 0.16462 | 0.11507 | 0.24388 | 0.21398 | 4 | 2.93229 | 0.55262 | 0.74881 | 0.665823 | Not Significant | NS | #N/A |
| 860 | G1SM01 | A-kinase anchoring protein 9 OS=Oryctolagus cunic | AKAP9 | RABBIT | -0.62726 | 3.36449 | 3.05829 | 3.23536 | 3.81408 | -1.40838 | 0 | -1.74896 | -1.26849 | #N/A | -3.67545 | -3.7634 | -4.25645 | 0.00704 | 0.0017 | 0.07875 | 0.04595 | 7 | 2.93229 | 0.55262 | 2.11957 | 1.656964 | Significant | Down mtub | AKAP9 |
| 394 | P84246 | Histone H3.3 OS=Oryctolagus cuniculus OX=9986 ( | H3-3A H3F | RABBIT | 6.11741 | 0.32032 | 6.87992 | 1.23239 | -0.93731 | -2.23113 | -1.89858 | 0.01349 | -0.07786 | -0.55877 | -3.67312 | -2.22352 | -2.56153 | 0.05687 | 0.02656 | 0.15449 | 0.11767 | 8 | 2.93229 | 0.55262 | 6.82222 | 5.140573 | Significant | Down mtub | H3-3A H3F3A |
| 2750 | G1ST17 | #N/A | PGLYRP1 | RABBIT | #N/A | #N/A | #N/A | 0.57169 | #N/A | #N/A | -0.62933 | #N/A | #N/A | #N/A | -1.20102 | #N/A | -1.14241 | #N/A | 0.33792 | #N/A | 0.36693 | 0 | 2.93229 | 0.55262 | #N/A | 0.552617 | Not Significant | NS | #N/A |
| 1234 | G1TXJ8 | Dematin actin binding protein OS=Oryctolagus cuni | DMTN | RABBIT | 1.57451 | 0.34752 | #N/A | -0.05764 | #N/A | #N/A | #N/A | -0.75897 | -0.40095 | #N/A | -1.20142 | -1.85592 | -1.81214 | 0.16048 | 0.12048 | 0.23968 | 0.21975 | 3 | 2.93229 | 0.55262 | 0.50287 | 0.527461 | Not Significant | NS | #N/A |
| 1279 | A0A5F9DJ89 | 2-iminobutanoate/2-iminopropanoate deaminase ( | #N/A | RABBIT | -0.32043 | 4.14181 | -0.22298 | 1.21643 | #N/A | #N/A | #N/A | #N/A | #N/A | 0 | -1.20371 | -0.51737 | -0.68601 | 0.64062 | 0.5186 | 0.4658 | 0.45158 | 3 | 2.93229 | 0.55262 | 4.33036 | 2.463048 | Not Significant | NS | #N/A |
| 2238 | G1SL16 | #N/A | #N/A | RABBIT | #N/A | #N/A | 0.17065 | #N/A | 1.94324 | -1.30542 | 0.09861 | -0.29674 | -0.56242 | 1.32726 | -1.20469 | -1.39306 | -1.53692 | 0.22237 | 0.16319 | 0.28347 | 0.25811 | 5 | 2.93229 | 0.55262 | 1.06835 | 0.877702 | Not Significant | NS | #N/A |
| 772 | U3KPA0 | Translin-associated protein X OS=Oryctolagus cuni | TSNAX | RABBIT | -0.51049 | 2.6567 | 0.52485 | -0.05764 | -0.65071 | #N/A | -0.82653 | 0.48467 | -3.65628 | 0.74285 | -1.20637 | -1.07915 | -1.23501 | 0.3163 | 0.24524 | 0.33209 | 0.31496 | 7 | 2.93229 | 0.55262 | 2.77707 | 2.120349 | Not Significant | NS | #N/A |
| 1557 | A0A5F9DKH6 | DS cell adhesion molecule like 1 OS=Oryctolagus c | DSCAML1 | RABBIT | -1.86046 | 0.51683 | 0.17065 | 1.11553 | 3.0606 | 0.81969 | -0.36812 | -1.68086 | -1.21217 | #N/A | -1.21099 | -1.18749 | -1.34858 | 0.27376 | 0.20742 | 0.31045 | 0.28797 | 7 | 2.93229 | 0.55262 | 2.31105 | 1.791909 | Not Significant | NS | #N/A |
| 2098 | A0A8J2MJT7 | #N/A | #N/A | RABBIT | #N/A | 0.67098 | #N/A | 0.24377 | -0.31479 | -1.01395 | #N/A | #N/A | #N/A | #N/A | -1.21393 | -2.12665 | -1.60765 | 0.16731 | 0.16962 | 0.24611 | 0.26263 | 2 | 2.93229 | 0.55262 | 0.24438 | 0.427628 | Not Significant | NS | #N/A |
| 124 | G1SKP2 | Importin 5 OS=Oryctolagus cuniculus OX=9986 GN | IPO5 | RABBIT | 0.35629 | 0.7538 | -0.11413 | 0.433 | -0.024 | -3.07083 | 0.1994 | -0.05375 | -0.80031 | -0.97088 | -1.22027 | -2.04144 | -2.15461 | 0.0755 | 0.05436 | 0.16901 | 0.15405 | 8 | 2.93229 | 0.55262 | 0.89325 | 0.801887 | Not Significant | NS | #N/A |
| 339 | G1U6B2 | Delta-aminolevulinic acid dehydratase OS=Oryctol: | ALAD | RABBIT | 0.91889 | 1.18903 | -0.81171 | -0.05764 | 0.27644 | #N/A | 0.14768 | -0.63975 | -2.04052 | -1.14221 | -1.22171 | -2.14183 | -2.22048 | 0.06945 | 0.05083 | 0.16349 | 0.15104 | 7 | 2.93229 | 0.55262 | 0.72302 | 0.672711 | Not Significant | NS | #N/A |
| 164 | G1U4Q4 | Phosphodiesterase OS=Oryctolagus cuniculus OX= | PDE2A | RABBIT | 0.87159 | 1.04045 | 0.22684 | -0.11383 | 0.79589 | -2.41449 | 1.45717 | -0.48511 | -0.36614 | -1.49613 | -1.22513 | -1.79419 | -1.93592 | 0.11053 | 0.07915 | 0.19806 | 0.18311 | 8 | 2.93229 | 0.55262 | 1.16565 | 1.001222 | Not Significant | NS | #N/A |
| 1864 | G1TJP0 | #N/A | UBQLN2 | RABBIT | #N/A | 1.14217 | #N/A | 0.25053 | #N/A | -1.25694 | -0.50742 | -0.06437 | -0.87036 | 0.05169 | -1.22583 | -2.59208 | -2.30035 | 0.04871 | 0.05071 | 0.14906 | 0.15095 | 5 | 2.93229 | 0.55262 | 0.3195 | 0.405673 | Not Significant | NS | #N/A |
| 2049 | G1SIH7 | #N/A | RNF31 | RABBIT | #N/A | 1.13024 | #N/A | #N/A | #N/A | 0.01366 | #N/A | -0.07803 | -0.23075 | #N/A | -1.22861 | -8.61737 | -1.83911 | 0.0132 | 0.12609 | 0.09752 | 0.2257 | 2 | 2.93229 | 0.55262 | 0.01525 | 0.334717 | Not Significant | NS | #N/A |
| 607 | G1SY68 | Splicing factor 3b subunit 2 OS=Oryctolagus cunicu | SF3B2 | RABBIT | -0.13911 | -0.17008 | 0.83509 | 1.34006 | 1.55451 | -3.04055 | -0.25914 | 0.59922 | 0.15077 | -0.17406 | -1.22885 | -1.66775 | -1.8186 | 0.13392 | 0.09645 | 0.21787 | 0.19688 | 8 | 2.93229 | 0.55262 | 1.3573 | 1.141465 | Not Significant | NS | #N/A |
| 529 | G1SW76 | Calcium-binding and coiled-coil domain-containing | CALCOCO | RABBIT | 0.07891 | #N/A | 0.10341 | #N/A | 1.68087 | #N/A | #N/A | #N/A | -3.02913 | #N/A | -3.65019 | -3.44389 | -3.86145 | 0.07496 | 0.01218 | 0.16836 | 0.09479 | 2 | 2.93229 | 0.55262 | 0.84255 | 0.670181 | Significant | Down mtub | CALCOCO1 |
| 2030 | G1SMB4 | #N/A | TENM4 | RABBIT | #N/A | #N/A | #N/A | -0.05764 | 1.12759 | #N/A | -0.701 | #N/A | #N/A | #N/A | -1.23598 | -1.20414 | -1.31305 | 0.44121 | 0.26055 | 0.38723 | 0.32441 | 1 | 2.93229 | 0.55262 | 0.70238 | 0.590703 | Not Significant | NS | #N/A |
| 2190 | G1SHJ1 | #N/A | SLC6A17 | RABBIT | #N/A | #N/A | 0.07791 | #N/A | 0.86811 | 0.09274 | -1.88984 | 0.83625 | #N/A | -2.09408 | -1.23674 | -1.10809 | -1.30794 | 0.32996 | 0.23259 | 0.33706 | 0.30372 | 4 | 2.93229 | 0.55262 | 1.66091 | 1.192112 | Not Significant | NS | #N/A |
| 816 | Q6IVV5 | PAPS synthase 1 OS=Oryctolagus cuniculus OX=99 | #N/A | RABBIT | -1.99089 | 0.25704 | 1.23746 | #N/A | 0.35841 | 0 | -2.26718 | -3.27219 | 0.08925 | -0.90867 | -1.23726 | -1.29139 | -1.45778 | 0.23757 | 0.17578 | 0.29265 | 0.26731 | 7 | 2.93229 | 0.55262 | 2.03984 | 1.600767 | Not Significant | NS | #N/A |
| 1764 | G1TEM4 | #N/A | STK39 | RABBIT | #N/A | 2.13658 | 1.80606 | 1.3607 | #N/A | -0.61221 | -6.31239 | -1.0735 | 0.61221 | -2.01591 | -3.64814 | -2.29461 | -2.72321 | 0.06155 | 0.02364 | 0.15739 | 0.11338 | 6 | 2.93229 | 0.55262 | 4.73944 | 3.364988 | Significant | Down mtub | STK39 |
| 2349 | A0A7G9P5T4 | #N/A | #N/A | RABBIT | #N/A | #N/A | 3.12216 | #N/A | #N/A | 0 | -0.72968 | #N/A | #N/A | #N/A | -3.487 | -5.51811 | -4.1104 | 0.11413 | 0.01525 | 0.20082 | 0.09934 | 1 | 2.93229 | 0.55262 | 0.26621 | 0.479783 | Significant | Down mtub | #N/A |
| 837 | G1SNT1 | Eukaryotic translation initiation factor 3 subunit J O: | EIF3J EIF3: | RABBIT | 0.18711 | 0.5022 | #N/A | -0.05764 | #N/A | #N/A | #N/A | -3.28389 | #N/A | -3.20258 | -3.45379 | -16.34 | -6.90451 | 0.0005 | 0.00048 | 0.03578 | 0.02567 | 3 | 2.93229 | 0.55262 | 0.05361 | 0.300267 | Significant | Down mtub | EIF3J EIF3S1 |
| 434 | Q28685 | Dystroglycan 1 OS=Oryctolagus cuniculus OX=998( | DAG1 | RABBIT | 1.13155 | 0.71458 | -0.61521 | -0.05764 | 0.53526 | #N/A | 0.36606 | -0.51816 | -3.0729 | -0.3977 | -1.24738 | -1.67782 | -1.83327 | 0.13728 | 0.09686 | 0.22058 | 0.19688 | 7 | 2.93229 | 0.55262 | 1.22827 | 1.028799 | Not Significant | NS | #N/A |
| 2801 | P09212 | #N/A | SOD1 | RABBIT | #N/A | #N/A | #N/A | #N/A | 0.61781 | -0.63119 | #N/A | #N/A | #N/A | #N/A | -1.249 | #N/A | -1.18805 | #N/A | 0.32207 | #N/A | 0.35646 | 0 | 2.93229 | 0.55262 | #N/A | 0.552617 | Not Significant | NS | #N/A |
| 2816 | G1SNV9 | #N/A | ESF1 | RABBIT | #N/A | #N/A | #N/A | #N/A | 1.05331 | #N/A | -0.60204 | #N/A | 0.18637 | -0.18637 | -1.25399 | -2.75354 | -1.7354 | 0.11046 | 0.144 | 0.19806 | 0.243 | 2 | 2.93229 | 0.55262 | 0.15555 | 0.391609 | Not Significant | NS | #N/A |
| 272 | G1TZS4 | Discs large MAGUK scaffold protein 4 OS=Oryctolag | DLG4 | RABBIT | 0.29777 | 0.14474 | -0.54184 | 0.29536 | 0.14514 | -1.1464 | -1.33835 | 0.00316 | 0.05308 | -3.5163 | -1.25719 | -1.88539 | -2.02704 | 0.09611 | 0.06775 | 0.1866 | 0.17137 | 8 | 2.93229 | 0.55262 | 1.11159 | 0.96166 | Not Significant | NS | #N/A |
| 1024 | G1SG06 | Platelet-derived growth factor receptor beta OS=Or | PDGFRB | RABBIT | 0.63457 | #N/A | #N/A | #N/A | #N/A | #N/A | #N/A | #N/A | #N/A | -0.62291 | -1.25748 | #N/A | -1.19612 | #N/A | 0.31935 | #N/A | 0.35646 | 0 | 2.93229 | 0.55262 | #N/A | 0.552617 | Not Significant | NS | #N/A |
| 556 | G1SI83 | S100 calcium binding protein A13 OS=Oryctolagus ( | S100A13 | RABBIT | 0.75128 | 1.65584 | -1.89047 | 0.67083 | 0.11969 | #N/A | 0 | -1.30295 | -2.09374 | -0.59201 | -1.25861 | -1.61419 | -1.77659 | 0.15052 | 0.10622 | 0.23143 | 0.20509 | 7 | 2.93229 | 0.55262 | 1.35103 | 1.115314 | Not Significant | NS | #N/A |
| 2396 | G1T2D1 | #N/A | MAPKBP1 | RABBIT | #N/A | #N/A | 0.17065 | #N/A | #N/A | -2.59136 | #N/A | #N/A | #N/A | 0.40767 | -1.2625 | -0.48609 | -0.82646 | 0.71195 | 0.45574 | 0.48851 | 0.42329 | 1 | 2.93229 | 0.55262 | 4.49707 | 1.555712 | Not Significant | NS | #N/A |
| 348 | G1TRY5 | Plastin-3 OS=Oryctolagus cuniculus OX=9986 GN= | PLS3 | RABBIT | 1.43634 | 1.99197 | -0.80649 | 0.67266 | -0.01338 | #N/A | -0.56092 | -0.47233 | -1.72301 | 0.32704 | -1.26352 | -1.86772 | -2.00797 | 0.10402 | 0.07261 | 0.19368 | 0.17535 | 7 | 2.93229 | 0.55262 | 1.01702 | 0.879914 | Not Significant | NS | #N/A |
| 727 | G1SRI8 | Hexokinase-1 OS=Oryctolagus cuniculus OX=9986 | HK1 | RABBIT | 0.39749 | 1.06691 | -0.55857 | 1.19732 | #N/A | #N/A | #N/A | -0.06437 | -2.02785 | -0.12148 | -1.26369 | -1.75705 | -1.89389 | 0.13925 | 0.09517 | 0.22132 | 0.19659 | 5 | 2.93229 | 0.55262 | 0.88673 | 0.763222 | Not Significant | NS | #N/A |
| 1968 | A0A5F9CR49 | #N/A | ITGA11 | RABBIT | #N/A | 2.44021 | -0.11285 | #N/A | #N/A | #N/A | -0.93007 | -0.06437 | #N/A | 0.69425 | -1.26374 | -1.12039 | -1.35408 | 0.34415 | 0.22502 | 0.34542 | 0.29961 | 3 | 2.93229 | 0.55262 | 1.52673 | 1.045231 | Not Significant | NS | #N/A |
| 2578 | A0A5F9CYF7 | #N/A | RAPGEF2 | RABBIT | #N/A | #N/A | #N/A | 1.43602 | #N/A | -0.0673 | #N/A | 0.87911 | -0.12445 | 0 | -1.26418 | -2.38422 | -1.81784 | 0.09725 | 0.11954 | 0.1871 | 0.21826 | 3 | 2.93229 | 0.55262 | 0.22491 | 0.386894 | Not Significant | NS | #N/A |
| 355 | G1T7V5 | Dihydrolipoyl dehydrogenase OS=Oryctolagus cuni | #N/A | RABBIT | 0.58295 | 0.21604 | 0.2332 | -0.09196 | 0.66263 | -2.66533 | -1.91567 | 0.79212 | -0.91381 | -0.01822 | -1.26476 | -1.98081 | -2.11491 | 0.08294 | 0.05823 | 0.17453 | 0.16044 | 8 | 2.93229 | 0.55262 | 1.01922 | 0.894067 | Not Significant | NS | #N/A |
| 521 | A0A5F9DQZ8 | NSFL1 cofactor OS=Oryctolagus cuniculus OX=998 | NSFL1C | RABBIT | 2.15091 | 0.91642 | -0.50443 | 2.22583 | -0.01338 | #N/A | 0.90456 | -0.45947 | -0.99077 | -0.69906 | -1.26625 | -1.74121 | -1.89576 | 0.12519 | 0.08743 | 0.21204 | 0.1893 | 7 | 2.93229 | 0.55262 | 1.17525 | 0.991428 | Not Significant | NS | #N/A |
| 2431 | G1SQY0 | #N/A | SLC6A7 | RABBIT | #N/A | #N/A | 0.60113 | #N/A | #N/A | #N/A | #N/A | -0.06437 | #N/A | -1.267 | -1.26682 | -1.21633 | -1.33983 | 0.43806 | 0.25249 | 0.38563 | 0.31897 | 1 | 2.93229 | 0.55262 | 0.72316 | 0.595987 | Not Significant | NS | #N/A |
| 2411 | O06625 | #N/A | #N/A | MT | #N/A | #N/A | 1.1833 | #N/A | #N/A | #N/A | #N/A | #N/A | -0.18502 | 0 | -1.27581 | -7.96239 | -1.61423 | 0.07954 | 0.183 | 0.17277 | 0.27307 | 1 | 2.93229 | 0.55262 | 0.01712 | 0.416436 | Not Significant | NS | #N/A |

| | | | | | | | | | | | | | | | | | | | | | | | | | | | | | |
|---|---|---|---|---|---|---|---|---|---|---|---|---|---|---|---|---|---|---|---|---|---|---|---|---|---|---|---|---|---|
| 28 | A0A5F9CTR4 | Glial fibrillary acidic protein OS=Oryctolagus cunicu | GFAP | RABBIT | -0.20674 | 0.41622 | 0.47804 | -0.27664 | #N/A | -3.06763 | #N/A | #N/A | #N/A | 0.72004 | -1.27651 | -1.0657 | -1.27448 | 0.34661 | 0.24355 | 0.34582 | 0.31373 | 4 | 2.93229 | 0.55262 | 1.91301 | 1.337577 | Not Significant | NS | #N/A |
| 231 | G1T0N4 | Nucleophosmin OS=Oryctolagus cuniculus OX=99 | #N/A | RABBIT | 0.04876 | 0.53273 | 0.01577 | -0.05356 | 0.17762 | -3.74881 | 0.01671 | -0.06844 | -1.28284 | -0.61893 | -1.28472 | -1.83524 | -1.98737 | 0.1038 | 0.07251 | 0.19361 | 0.17535 | 8 | 2.93229 | 0.55262 | 1.2251 | 1.044727 | Not Significant | NS | #N/A |
| 2374 | A0A5F9D2S1 | #N/A | SLC6A11 | RABBIT | #N/A | #N/A | #N/A | #N/A | 0.96248 | #N/A | 0 | #N/A | #N/A | -0.65392 | -1.28944 | -2.27692 | -1.54152 | 0.26345 | 0.19925 | 0.30573 | 0.28306 | 1 | 2.93229 | 0.55262 | 0.2138 | 0.466455 | Not Significant | NS | #N/A |
| 493 | A0A5F9CGI8 | Methylosome protein WDR77 OS=Oryctolagus cuni | WDR77 | RABBIT | 0.19171 | -0.65126 | 2.73832 | -0.27456 | 2.26275 | -3.71678 | 0.14699 | -0.21136 | -1.3915 | 2.98824 | -1.29028 | -0.99986 | -1.14139 | 0.34666 | 0.27809 | 0.34582 | 0.3326 | 8 | 2.93229 | 0.55262 | 4.16319 | 3.194755 | Not Significant | NS | #N/A |
| 1609 | A0A5F9CNP3 | BICD cargo adaptor 1 OS=Oryctolagus cuniculus O | BICD1 | RABBIT | 4.00773 | #N/A | 3.67751 | 1.90319 | #N/A | #N/A | -0.15286 | -0.06437 | -0.5783 | -0.03114 | -3.40281 | -6.003 | -5.99942 | 0.00184 | 0.00033 | 0.05406 | 0.02295 | 5 | 2.93229 | 0.55262 | 0.55084 | 0.551494 | Significant | Down mtub | BICD1 |
| 341 | P15253 | Calreticulin OS=Oryctolagus cuniculus OX=9986 G | CALR | RABBIT | 0.46289 | 2.09786 | -0.72472 | 0.47008 | -0.61176 | -4.20057 | 0.54829 | 0.35004 | -1.09039 | -0.4144 | -1.30027 | -1.30129 | -1.463 | 0.22938 | 0.17162 | 0.28914 | 0.26461 | 8 | 2.93229 | 0.55262 | 2.49609 | 1.974804 | Not Significant | NS | #N/A |
| 2551 | A0A5F9DFN4 | #N/A | EIF2B3 | RABBIT | #N/A | #N/A | #N/A | 0.31769 | #N/A | -4.83884 | 0.10964 | -0.17401 | 0.19759 | -0.20771 | -1.30036 | -0.54885 | -0.69315 | 0.6123 | 0.51076 | 0.45455 | 0.44921 | 4 | 2.93229 | 0.55262 | 4.67767 | 2.932817 | Not Significant | NS | #N/A |
| 2536 | A0A5F9DFM4 | #N/A | FMR1 | RABBIT | #N/A | #N/A | #N/A | 3.13391 | #N/A | -0.48435 | #N/A | #N/A | 0 | #N/A | -3.37608 | -8.04861 | -4.14666 | 0.07869 | 0.01481 | 0.17251 | 0.09934 | 1 | 2.93229 | 0.55262 | 0.1173 | 0.441913 | Significant | Down mtub | FMR1 |
| 1588 | G1SV17 | Cell division cycle 23 OS=Oryctolagus cuniculus O | CDC23 | RABBIT | 1.09725 | #N/A | #N/A | #N/A | 0.9984 | -3.50941 | #N/A | #N/A | #N/A | -1.01178 | -3.30842 | -2.64718 | -3.3733 | 0.11798 | 0.02024 | 0.20536 | 0.10939 | 2 | 2.93229 | 0.55262 | 1.56198 | 0.961906 | Significant | Down mtub | CDC23 |
| 683 | G1U7S4 | Phosphoglycerate mutase OS=Oryctolagus cunicul | PGAM2 | RABBIT | 0.40009 | -2.77693 | -0.37791 | 0.22825 | 0.21849 | -0.23187 | 0.27869 | #N/A | -5.34273 | #N/A | -1.3037 | -0.85143 | -1.00834 | 0.4272 | 0.33982 | 0.38091 | 0.3676 | 6 | 2.93229 | 0.55262 | 4.396 | 3.134294 | Not Significant | NS | #N/A |
| 668 | G1SXP3 | GST C-terminal domain-containing protein OS=Oryc | #N/A | RABBIT | 1.0115 | 2.4363 | #N/A | -0.05764 | -0.78876 | #N/A | 0.22107 | -4.7673 | #N/A | -3.22422 | -3.2405 | -2.18012 | -2.63552 | 0.0811 | 0.03014 | 0.1731 | 0.12246 | 5 | 2.93229 | 0.55262 | 3.78746 | 2.591652 | Significant | Down mtub | #N/A |
| 764 | G1T3I0 | m7GpppX diphosphatase OS=Oryctolagus cunicul | #N/A | RABBIT | 2.7647 | 2.17392 | -0.13155 | 1.02988 | -0.02743 | #N/A | -0.28403 | -0.05032 | #N/A | -0.10664 | -1.3089 | -1.69256 | -1.85391 | 0.14149 | 0.097 | 0.22438 | 0.19688 | 6 | 2.93229 | 0.55262 | 1.12131 | 0.934618 | Not Significant | NS | #N/A |
| 565 | A0A5F9CY32 | Protein phosphatase 1 regulatory subunit 9A OS=O | PPP1R9A | RABBIT | 0.60135 | 0.03979 | 0.10193 | 0.31569 | 0.0001 | #N/A | -2.45652 | -0.06437 | 0.6752 | -2.55038 | -1.31079 | -1.78199 | -1.94378 | 0.11795 | 0.08077 | 0.20536 | 0.18391 | 7 | 2.93229 | 0.55262 | 1.20238 | 1.010552 | Not Significant | NS | #N/A |
| 1193 | A5U052 | DNA-directed RNA polymerase subunit beta OS=My | #N/A | MT | -0.00805 | #N/A | 1.48749 | #N/A | #N/A | 0.01972 | #N/A | #N/A | -1.17444 | #N/A | -1.31708 | -1.3764 | -1.5744 | 0.30254 | 0.17701 | 0.32474 | 0.26786 | 2 | 2.93229 | 0.55262 | 0.91566 | 0.699828 | Not Significant | NS | #N/A |
| 1290 | G1SK97 | ArfGAP with SH3 domain, ankyrin repeat and PH do | ASAP2 | RABBIT | #N/A | #N/A | #N/A | 1.72979 | #N/A | -0.64672 | 2.916 | -0.13875 | -0.15489 | 0.07439 | -1.31978 | -0.8451 | -1.01589 | 0.44564 | 0.34382 | 0.38942 | 0.36959 | 4 | 2.93229 | 0.55262 | 2.0324 | 1.406465 | Not Significant | NS | #N/A |
| 218 | A0A5F9DVE1 | Tropomyosin alpha-4 chain OS=Oryctolagus cunicu | TPM4 | RABBIT | 0.45317 | 0.26045 | 0.54867 | -0.12257 | 0.52007 | -5.03352 | 0.67987 | -0.23078 | -0.06263 | -0.29464 | -1.32029 | -1.27736 | -1.43962 | 0.2373 | 0.17799 | 0.29256 | 0.26845 | 8 | 2.93229 | 0.55262 | 2.6709 | 2.10273 | Not Significant | NS | #N/A |
| 1020 | G1SU84 | Nischarin OS=Oryctolagus cuniculus OX=9986 GN= | NISCH | RABBIT | 0.4933 | #N/A | 1.9903 | 2.71067 | 1.54757 | -0.48164 | #N/A | -1.48588 | -1.14432 | -3.07659 | -3.23257 | -4.48825 | -4.87801 | 0.00416 | 0.00089 | 0.06755 | 0.036 | 6 | 2.93229 | 0.55262 | 1.03746 | 0.878297 | Significant | Down mtub | NISCH |
| 2150 | A0A5F9DDC5 | #N/A | NDRG2 | RABBIT | #N/A | #N/A | 0.94492 | #N/A | #N/A | -1.04952 | #N/A | 0.21856 | -0.67555 | 0 | -1.32155 | -2.00929 | -1.76566 | 0.13808 | 0.12845 | 0.22114 | 0.22682 | 3 | 2.93229 | 0.55262 | 0.34608 | 0.448168 | Not Significant | NS | #N/A |
| 1305 | A0A5F9C2M0 | ATP-dependent RNA helicase OS=Oryctolagus cuni | DDX31 | RABBIT | 0.63503 | #N/A | #N/A | #N/A | #N/A | #N/A | #N/A | -0.68773 | #N/A | #N/A | -1.32276 | #N/A | -1.25821 | #N/A | 0.29918 | #N/A | 0.34612 | 0 | 2.93229 | 0.55262 | #N/A | 0.552617 | Not Significant | NS | #N/A |
| 1111 | A0A5F9DRA4 | Haloacid dehalogenase-like hydrolase domain-con | HDHD2 | RABBIT | 1.23988 | 2.66206 | -0.57678 | 0.24069 | -0.20113 | #N/A | -0.50703 | -1.64613 | #N/A | 0.18775 | -1.32808 | -1.52743 | -1.70804 | 0.17751 | 0.12206 | 0.25448 | 0.22013 | 6 | 2.93229 | 0.55262 | 1.41752 | 1.133592 | Not Significant | NS | #N/A |
| 104 | G1T332 | Aspartate aminotransferase OS=Oryctolagus cunic | GOT1 | RABBIT | 2.30041 | 3.2821 | 0.24209 | 1.75398 | 0.49278 | -4.54249 | -0.07144 | -1.0876 | -1.79884 | -0.58918 | -3.23218 | -3.34001 | -3.74575 | 0.01023 | 0.00327 | 0.09529 | 0.05702 | 8 | 2.93229 | 0.55262 | 2.34119 | 1.861456 | Significant | Down mtub | GOT1 |
| 1013 | A0A5F9DIT0 | Alpha-ketoglutarate-dependent dioxygenase FTO O | FTO | RABBIT | -0.0745 | 0.28398 | #N/A | 0.07353 | 1.17131 | #N/A | #N/A | #N/A | -1.55541 | -0.3808 | -1.33169 | -2.41279 | -2.24748 | 0.07333 | 0.05978 | 0.16663 | 0.16242 | 4 | 2.93229 | 0.55262 | 0.40617 | 0.468113 | Not Significant | NS | #N/A |
| 2387 | A0A5F9CXV5 | #N/A | MAP4 | RABBIT | #N/A | #N/A | 3.13177 | #N/A | #N/A | #N/A | 0.06145 | -0.12581 | #N/A | -0.16873 | -3.20947 | -22.7085 | -4.80501 | 0.00193 | 0.00504 | 0.05406 | 0.06702 | 2 | 2.93229 | 0.55262 | 0.01498 | 0.33461 | Significant | Down mtub | MAP4 |
| 1307 | U3KMX6 | Transcription factor BTF3 OS=Oryctolagus cuniculu | BTF3 | RABBIT | 0.01166 | #N/A | 0.36845 | #N/A | #N/A | #N/A | #N/A | #N/A | #N/A | -2.96761 | -3.15767 | -10.2194 | -3.93969 | 0.0621 | 0.01753 | 0.15739 | 0.10298 | 1 | 2.93229 | 0.55262 | 0.06365 | 0.42827 | Significant | Down mtub | BTF3 |
| 2057 | G1T8R3 | #N/A | UTRN | RABBIT | #N/A | 1.42604 | 2.552 | -2.33304 | #N/A | -0.53686 | #N/A | 0.4725 | -2.29777 | #N/A | -1.33571 | -0.7931 | -0.99766 | 0.47211 | 0.35198 | 0.40181 | 0.37531 | 4 | 2.93229 | 0.55262 | 4.25468 | 2.688744 | Not Significant | NS | #N/A |
| 1765 | G1SUA4 | #N/A | #N/A | RABBIT | #N/A | 2.35879 | #N/A | -0.05764 | #N/A | #N/A | #N/A | #N/A | #N/A | -0.18789 | -1.33847 | -0.63959 | -1.01709 | 0.63775 | 0.36755 | 0.4644 | 0.38387 | 1 | 2.93229 | 0.55262 | 2.91955 | 1.154541 | Not Significant | NS | #N/A |
| 532 | G1SQY8 | Nuclear migration protein nudC OS=Oryctolagus cu | NUDC | RABBIT | 1.62925 | 1.889 | #N/A | 0.66877 | -2.51374 | -2.73403 | -0.01003 | -0.94951 | #N/A | 0.01003 | -1.33921 | -1.11621 | -1.30221 | 0.30703 | 0.22542 | 0.32551 | 0.29961 | 6 | 2.93229 | 0.55262 | 2.87892 | 2.115245 | Not Significant | NS | #N/A |
| 1756 | A0A5F9CRI5 | #N/A | STXBP5L | RABBIT | #N/A | 3.70078 | -1.1049 | #N/A | -0.01338 | #N/A | #N/A | 0.97027 | -1.9297 | #N/A | -1.34055 | -0.61874 | -0.83114 | 0.57989 | 0.43806 | 0.44328 | 0.41582 | 3 | 2.93229 | 0.55262 | 5.63284 | 3.121724 | Not Significant | NS | #N/A |
| 14 | A0A5F9DDB1 | Pyruvate kinase OS=Oryctolagus cuniculus OX=99 | #N/A | RABBIT | 1.1438 | 1.724 | -0.69894 | 0.12865 | 1.23001 | #N/A | -1.32511 | -0.39421 | #N/A | -0.18628 | -1.3407 | -2.10924 | -2.20964 | 0.07946 | 0.0547 | 0.17277 | 0.1545 | 6 | 2.93229 | 0.55262 | 0.75755 | 0.690278 | Not Significant | NS | #N/A |
| 95 | A0A5F9D937 | Neural cell adhesion molecule 1 OS=Oryctolagus c | NCAM1 | RABBIT | -4.33981 | 0.78306 | 0.32719 | 0.31915 | 0.71248 | -3.00188 | 0.25094 | -0.22091 | -5.72338 | -0.21454 | -1.34237 | -0.89247 | -1.02509 | 0.39819 | 0.32747 | 0.36967 | 0.36005 | 8 | 2.93229 | 0.55262 | 5.65586 | 4.287053 | Not Significant | NS | #N/A |
| 942 | A0A5F9C499 | SWI/SNF related, matrix associated, actin depende | SMARCC2 | RABBIT | -0.05825 | #N/A | 0.4442 | 0.17223 | 0.46613 | #N/A | 0.06991 | -1.0574 | -3.14555 | -0.21345 | -1.3427 | -1.82082 | -1.98842 | 0.11849 | 0.07824 | 0.206 | 0.18205 | 6 | 2.93229 | 0.55262 | 1.08756 | 0.91195 | Not Significant | NS | #N/A |
| 401 | A0A5F9DUX0 | Nardilysin convertase OS=Oryctolagus cuniculus O | NRDC | RABBIT | 2.10798 | 2.23256 | 0.17065 | 0.67927 | 1.88145 | #N/A | -1.26222 | -1.67112 | -1.4831 | -2.36681 | -3.11019 | -6.02879 | -6.08805 | 0.00053 | 0.00012 | 0.03578 | 0.01728 | 7 | 2.93229 | 0.55262 | 0.59143 | 0.57997 | Significant | Down mtub | NRDC |
| 2327 | A0A5F9D9V2 | #N/A | #N/A | RABBIT | #N/A | #N/A | 1.0056 | #N/A | #N/A | -0.67819 | #N/A | #N/A | 0 | #N/A | -1.3447 | -2.2895 | -1.60055 | 0.26216 | 0.18595 | 0.3052 | 0.27484 | 1 | 2.93229 | 0.55262 | 0.22997 | 0.470567 | Not Significant | NS | #N/A |
| 2226 | G1SW53 | #N/A | RPS6KA5 | RABBIT | #N/A | #N/A | 0.17065 | #N/A | #N/A | #N/A | #N/A | 1.01684 | #N/A | -3.3678 | -1.34613 | -0.3545 | -0.6503 | 0.78311 | 0.55153 | 0.50847 | 0.46295 | 1 | 2.93229 | 0.55262 | 9.61255 | 2.856603 | Not Significant | NS | #N/A |
| 36 | G1SK04 | phosphopyruvate hydratase OS=Oryctolagus cunic | ENO2 | RABBIT | 1.85368 | 2.81667 | 0.19923 | 1.20437 | 0.21278 | -5.88346 | -0.02858 | -0.91178 | -1.75884 | -0.41388 | -3.05665 | -2.60553 | -2.95995 | 0.03135 | 0.01306 | 0.12952 | 0.09653 | 8 | 2.93229 | 0.55262 | 3.44065 | 2.666016 | Significant | Down mtub | ENO2 |
| 1384 | A0A5F9D493 | Conserved oligomeric Golgi complex subunit 2 OS= | COG2 | RABBIT | 1.10569 | #N/A | #N/A | #N/A | #N/A | #N/A | #N/A | #N/A | -0.48752 | 0 | -1.34945 | -3.19618 | -1.65673 | 0.19304 | 0.17414 | 0.26379 | 0.26635 | 1 | 2.93229 | 0.55262 | 0.11884 | 0.442305 | Not Significant | NS | #N/A |
| 512 | G1SMI4 | Rabaptin, RAB GTPase binding effector protein 1 OS | RABEP1 | RABBIT | 2.30546 | #N/A | -0.43217 | #N/A | #N/A | #N/A | #N/A | 0.21303 | 0 | -1.47276 | -1.35656 | -1.10428 | -1.36285 | 0.35009 | 0.22239 | 0.34667 | 0.29903 | 3 | 2.93229 | 0.55262 | 1.81092 | 1.188948 | Not Significant | NS | #N/A |
| 1942 | G1SG66 | #N/A | PLCE1 | RABBIT | #N/A | 1.0606 | #N/A | #N/A | #N/A | #N/A | 0.23753 | -0.71637 | -0.71577 | 0 | -1.35925 | -2.47281 | -1.93344 | 0.08984 | 0.10191 | 0.1797 | 0.20137 | 3 | 2.93229 | 0.55262 | 0.24172 | 0.395393 | Not Significant | NS | #N/A |
| 2220 | G1U729 | #N/A | IGSF8 | RABBIT | #N/A | #N/A | 0.17065 | 0.16577 | 1.19715 | #N/A | 0.28462 | -0.74158 | -0.73457 | -2.20858 | -1.36122 | -2.02738 | -2.1434 | 0.09844 | 0.06472 | 0.18766 | 0.16687 | 5 | 2.93229 | 0.55262 | 0.7728 | 0.691407 | Not Significant | NS | #N/A |
| 178 | G1SL24 | Neurofilament heavy OS=Oryctolagus cuniculus O | NEFH | RABBIT | 0.37958 | 0.04985 | 1.14761 | -0.63839 | 1.13394 | -4.5185 | 0.14797 | 0.71194 | -0.33136 | -0.75612 | -1.36373 | -1.38395 | -1.55427 | 0.20376 | 0.14857 | 0.27183 | 0.24819 | 8 | 2.93229 | 0.55262 | 2.42749 | 1.924606 | Not Significant | NS | #N/A |
| 246 | A0A5F9CB43 | Kinectin 1 OS=Oryctolagus cuniculus OX=9986 GN | KTN1 | RABBIT | -0.10405 | -0.15013 | 0.40402 | 0.89598 | -0.82134 | -6.48068 | 0.95141 | 0.72087 | -1.93706 | 0.11571 | -1.37084 | -0.96857 | -1.11003 | 0.36112 | 0.29081 | 0.35226 | 0.34104 | 8 | 2.93229 | 0.55262 | 5.00785 | 3.812856 | Not Significant | NS | #N/A |
| 761 | A0A5F9DDQ8 | Stathmin OS=Oryctolagus cuniculus OX=9986 PE= | #N/A | RABBIT | 1.31803 | 3.23987 | 0.71078 | 0.65784 | -0.55352 | #N/A | #N/A | -2.44291 | -0.96942 | -2.43482 | -3.02365 | -3.35382 | -3.77154 | 0.01535 | 0.00447 | 0.10246 | 0.06435 | 6 | 2.93229 | 0.55262 | 1.524 | 1.205115 | Significant | Down mtub | #N/A |
| 2688 | A0A5F9CSM3 | #N/A | ZNF112 | RABBIT | #N/A | #N/A | #N/A | 1.24441 | #N/A | 0 | #N/A | #N/A | -0.25787 | #N/A | -1.37335 | -6.14964 | -1.72915 | 0.10262 | 0.16007 | 0.19228 | 0.25503 | 1 | 2.93229 | 0.55262 | 0.03325 | 0.420539 | Not Significant | NS | #N/A |
| 2618 | A0A5F9C6B1 | #N/A | UGGT1 | RABBIT | #N/A | #N/A | #N/A | -0.05764 | 1.94416 | #N/A | 1.5591 | -0.85311 | -1.9995 | #N/A | -1.37443 | -0.88912 | -1.14693 | 0.43943 | 0.29555 | 0.38598 | 0.34496 | 3 | 2.93229 | 0.55262 | 2.86749 | 1.723265 | Not Significant | NS | #N/A |
| 1629 | P34032 | Thymosin beta-4 OS=Oryctolagus cuniculus OX=99 | TMSB4 | RABBIT | #N/A | 2.60843 | #N/A | #N/A | #N/A | #N/A | 0 | -1.14824 | -0.71711 | 0.48154 | -2.95438 | -3.63447 | -3.59436 | 0.03588 | 0.01167 | 0.13507 | 0.09305 | 3 | 2.93229 | 0.55262 | 0.52862 | 0.540479 | Significant | Down mtub | TMSB4 |
| 945 | Q28717 | Serine/threonine-protein phosphatase 2A activator | PTPA PPP2 | RABBIT | 0.15973 | 2.91407 | -0.5292 | 0.49085 | #N/A | #N/A | -0.46801 | -1.38297 | #N/A | 0 | -1.37585 | -1.44933 | -1.65969 | 0.20693 | 0.13588 | 0.27258 | 0.23462 | 5 | 2.93229 | 0.55262 | 1.54488 | 1.178074 | Not Significant | NS | #N/A |
| 505 | Q2PPJ8 | Apolipoprotein A-I OS=Oryctolagus cuniculus OX=9 | apoA-I | RABBIT | 1.24023 | #N/A | #N/A | #N/A | #N/A | #N/A | 0 | #N/A | #N/A | -0.27474 | -1.3776 | -5.78995 | -1.73215 | 0.10888 | 0.15952 | 0.19697 | 0.25503 | 1 | 2.93229 | 0.55262 | 0.03774 | 0.421681 | Not Significant | NS | #N/A |
| 148 | G1SFV7 | DNA damage-binding protein 1 OS=Oryctolagus cu | DDB1 | RABBIT | 0.57794 | 1.08685 | -0.36446 | 0.49503 | 0.29865 | -3.66319 | 0.17614 | -0.38865 | -0.75593 | -0.17614 | -1.38036 | -1.88843 | -2.05709 | 0.09566 | 0.06434 | 0.1866 | 0.16683 | 8 | 2.93229 | 0.55262 | 1.33574 | 1.125686 | Not Significant | NS | #N/A |
| 204 | P13806 | Voltage-dependent calcium channel subunit alpha- | CACNA2D | RABBIT | 2.11327 | 1.76381 | 0.89917 | #N/A | 1.74251 | #N/A | -0.72852 | -1.15256 | -1.73644 | -1.66553 | -2.95045 | -8.43293 | -7.09497 | 0.00015 | 5.9E-05 | 0.03578 | 0.01186 | 6 | 2.93229 | 0.55262 | 0.24482 | 0.345865 | Significant | Down mtub | CACNA2D1 CACNL2A CCHL2A |
| 275 | G1T670 | Proteasome subunit alpha type OS=Oryctolagus cu | PSMA5 | RABBIT | 0.37239 | 0.10909 | 0.25938 | -0.22516 | 0.6489 | -5.16607 | 0.60843 | -0.36797 | -0.98375 | 0.0954 | -1.39571 | -1.33597 | -1.50681 | 0.21831 | 0.1602 | 0.28083 | 0.25503 | 8 | 2.93229 | 0.55262 | 2.72859 | 2.144944 | Not Significant | NS | #N/A |
| 578 | G1U1Q8 | Macrophage migration inhibitory factor OS=Oryctol | MIF | RABBIT | 1.92199 | 3.93366 | 0.17065 | 1.04246 | -3.81243 | #N/A | 0.55537 | -1.77488 | -1.52729 | -0.23824 | -1.39752 | -0.91462 | -1.06595 | 0.39081 | 0.31167 | 0.36581 | 0.35272 | 7 | 2.93229 | 0.55262 | 5.18827 | 3.819694 | Not Significant | NS | #N/A |
| 732 | G1TCA0 | Zinc finger RNA-binding protein OS=Oryctolagus cu | ZFR | RABBIT | 0.01166 | 1.23842 | #N/A | 0.73979 | #N/A | #N/A | #N/A | -2.0725 | -2.47744 | #N/A | -2.93826 | -6.07112 | -4.99461 | 0.00897 | 0.00255 | 0.09045 | 0.05283 | 3 | 2.93229 | 0.55262 | 0.28108 | 0.415297 | Significant | Down mtub | ZFR |
| 769 | G1SD46 | Ciliary neurotrophic factor receptor subunit alpha C | CNTFR | RABBIT | 1.40556 | 2.13199 | 2.12137 | 0.81023 | -0.01338 | -3.37848 | -1.13994 | -0.64166 | -1.39238 | #N/A | -2.92927 | -4.17085 | -4.51434 | 0.00418 | 0.00114 | 0.06755 | 0.04239 | 7 | 2.93229 | 0.55262 | 1.09611 | 0.935659 | Significant | Down mtub | CNTFR |
| 1684 | A0A5F9CRP6 | #N/A | ADAM2 | RABBIT | #N/A | 1.23296 | #N/A | #N/A | #N/A | #N/A | 0 | -0.34212 | #N/A | #N/A | -1.40402 | -4.73878 | -1.75441 | 0.1324 | 0.15545 | 0.21631 | 0.25326 | 1 | 2.93229 | 0.55262 | 0.05852 | 0.426966 | Not Significant | NS | #N/A |
| 1704 | G1TMM7 | #N/A | #N/A | RABBIT | #N/A | 3.17825 | #N/A | 1.05836 | #N/A | #N/A | #N/A | -0.12244 | -2.29846 | 0 | -2.92527 | -2.34726 | -2.90635 | 0.10056 | 0.02747 | 0.18996 | 0.11898 | 3 | 2.93229 | 0.55262 | 1.86376 | 1.215672 | Significant | Down mtub | #N/A |
| 1017 | G1SU01 | Nipsnap homolog 3A OS=Oryctolagus cuniculus O | NIPSNAP3 | RABBIT | 0.4611 | 2.43155 | -0.35161 | 0.85947 | -0.01338 | #N/A | -1.91001 | 1.20867 | -1.673 | -0.58502 | -1.41727 | -1.70456 | -1.89283 | 0.13205 | 0.08785 | 0.21622 | 0.1893 | 7 | 2.93229 | 0.55262 | 1.53626 | 1.245858 | Not Significant | NS | #N/A |
| 1434 | G1TW15 | Myotubularin related protein 10 OS=Oryctolagus cu | MTMR10 | RABBIT | 0.68253 | #N/A | #N/A | #N/A | #N/A | #N/A | #N/A | -0.73524 | #N/A | #N/A | -1.41777 | #N/A | -1.34859 | #N/A | 0.27217 | #N/A | 0.33034 | 0 | 2.93229 | 0.55262 | #N/A | 0.552617 | Not Significant | NS | #N/A |
| 533 | G1T0Z8 | Prefoldin subunit 5 OS=Oryctolagus cuniculus OX= | PFDN5 | RABBIT | 1.28368 | 1.26856 | -0.29678 | 0.73937 | -0.02016 | -3.80239 | 0.5083 | -0.05758 | -0.14279 | -0.62016 | -1.41786 | -1.70312 | -1.88385 | 0.12695 | 0.08644 | 0.2129 | 0.18856 | 8 | 2.93229 | 0.55262 | 1.73267 | 1.41615 | Not Significant | NS | #N/A |
| 347 | A0A5F9DGS3 | Importin subunit alpha OS=Oryctolagus cuniculus ( | KPNA4 | RABBIT | 0.36505 | #N/A | #N/A | 0.26058 | #N/A | #N/A | 0.35885 | -0.38258 | -1.38045 | -3.01812 | -1.41839 | -1.29419 | -1.52209 | 0.26526 | 0.17221 | 0.30603 | 0.26481 | 4 | 2.93229 | 0.55262 | 1.60152 | 1.157847 | Not Significant | NS | #N/A |
| 2804 | A5U2X3 | #N/A | #N/A | MT | #N/A | #N/A | #N/A | #N/A | 0.92561 | #N/A | #N/A | -0.06437 | #N/A | -0.92963 | -1.42261 | -1.89848 | -1.63086 | 0.30864 | 0.17948 | 0.32678 | 0.26969 | 1 | 2.93229 | 0.55262 | 0.37434 | 0.507281 | Not Significant | NS | #N/A |
| 1702 | O77708 | #N/A | CAMK2D | RABBIT | #N/A | 0.13863 | 0.85217 | #N/A | #N/A | -3.89758 | #N/A | 1.1108 | #N/A | 0 | -1.42433 | -0.71993 | -0.95875 | 0.52359 | 0.37511 | 0.4245 | 0.38769 | 3 | 2.93229 | 0.55262 | 4.69696 | 2.648441 | Not Significant | NS | #N/A |
| 1515 | A0A5F9D7C0 | Methyltransferase like 4 OS=Oryctolagus cuniculus | METTL4 | RABBIT | #N/A | #N/A | 0.79762 | #N/A | #N/A | -0.62697 | #N/A | #N/A | #N/A | #N/A | -1.42459 | #N/A | -1.35507 | #N/A | 0.27033 | #N/A | 0.32989 | 0 | 2.93229 | 0.55262 | #N/A | 0.552617 | Not Significant | NS | #N/A |
| 1139 | G1SIA6 | Armadillo like helical domain containing 3 OS=Oryc | ARMH3 | RABBIT | 1.14095 | 0.01781 | #N/A | 1.66975 | 0.16662 | 2.31631 | -0.5221 | #N/A | -1.81296 | -2.69115 | -1.42626 | -1.22729 | -1.42777 | 0.26568 | 0.18737 | 0.30603 | 0.27558 | 6 | 2.93229 | 0.55262 | 2.70106 | 1.995769 | Not Significant | NS | #N/A |
| 189 | G1SR16 | COP9 signalosome complex subunit 2 OS=Oryctola | COPS2 | RABBIT | 0.4175 | 0.17934 | 0.18913 | 0.01156 | 0.63242 | -3.89398 | 0.043 | -0.32569 | -0.53449 | -1.00637 | -1.42949 | -1.99536 | -2.16772 | 0.0811 | 0.05313 | 0.1731 | 0.1526 | 8 | 2.93229 | 0.55262 | 1.2831 | 1.087169 | Not Significant | NS | #N/A |
| 63 | G1SVB6 | Glutamate dehydrogenase OS=Oryctolagus cunicul | #N/A | RABBIT | 0.74238 | 0.84253 | 0.01899 | -0.52618 | 0.13828 | -4.69795 | 0.31023 | 0.11666 | -0.80182 | -0.88783 | -1.43534 | -1.52286 | -1.70419 | 0.16629 | 0.11657 | 0.24561 | 0.21451 | 8 | 2.93229 | 0.55262 | 2.22091 | 1.773439 | Not Significant | NS | #N/A |
| 1467 | G1TZR0 | ADAM metallopeptidase domain 20 OS=Oryctolagu | #N/A | RABBIT | 0.72351 | #N/A | #N/A | #N/A | #N/A | #N/A | #N/A | #N/A | -0.71185 | #N/A | -1.43536 | #N/A | -1.36532 | #N/A | 0.26746 | #N/A | 0.32868 | 0 | 2.93229 | 0.55262 | #N/A | 0.552617 | Not Significant | NS | #N/A |
| 446 | G1T9U6 | Calretinin OS=Oryctolagus cuniculus OX=9986 GN= | CALB2 | RABBIT | 1.56301 | 3.15567 | 0.02837 | 1.05796 | -0.18735 | #N/A | -0.61304 | -0.71214 | 0.0618 | 0 | -1.43938 | -2.04152 | -2.21114 | 0.08053 | 0.05164 | 0.1731 | 0.15166 | 7 | 2.93229 | 0.55262 | 1.10466 | 0.94168 | Not Significant | NS | #N/A |
| 105 | A0A5F9DHL3 | Neurofascin OS=Oryctolagus cuniculus OX=9986 G | NFASC | RABBIT | 1.15967 | 0.36917 | 1.07014 | 1.16079 | 1.17062 | -7.11066 | -0.70911 | -0.23572 | -0.80869 | -0.79431 | -2.91778 | -2.23031 | -2.5476 | 0.05627 | 0.02723 | 0.15432 | 0.11898 | 8 | 2.93229 | 0.55262 | 4.27874 | 3.279306 | Significant | Down mtub | NFASC |
| 1395 | A0A5F9D1X6 | Microtubule associated scaffold protein 1 OS=Oryc | MTUS1 | RABBIT | 0.25155 | #N/A | #N/A | #N/A | #N/A | -2.37863 | #N/A | #N/A | 0 | #N/A | -1.44086 | -0.69946 | -1.10599 | 0.61143 | 0.33176 | 0.45451 | 0.36184 | 1 | 2.93229 | 0.55262 | 2.82893 | 1.131494 | Not Significant | NS | #N/A |
| 1221 | G1TAV9 | Transforming growth factor beta receptor 3 OS=Ory | TGFBR3 | RABBIT | 0.73235 | #N/A | #N/A | #N/A | #N/A | #N/A | #N/A | #N/A | #N/A | -0.72069 | -1.45304 | #N/A | -1.38214 | #N/A | 0.26281 | #N/A | 0.32511 | 0 | 2.93229 | 0.55262 | #N/A | 0.552617 | Not Significant | NS | #N/A |
| 797 | G1T7S1 | NADP-dependent oxidoreductase domain-containi | #N/A | RABBIT | 1.0059 | 0.1108 | #N/A | 2.91285 | -0.01338 | -1.46022 | #N/A | 0.40184 | #N/A | -0.29151 | -1.45401 | -1.5812 | -1.80047 | 0.17467 | 0.10979 | 0.25338 | 0.20887 | 5 | 2.93229 | 0.55262 | 1.44957 | 1.118 | Not Significant | NS | #N/A |
| 2463 | G1SVV4 | #N/A | AUTS2 | RABBIT | #N/A | #N/A | 0.25922 | 1.98182 | #N/A | -0.58104 | #N/A | #N/A | -0.08857 | #N/A | -1.45532 | -1.62459 | -1.79967 | 0.24575 | 0.13262 | 0.29746 | 0.23225 | 2 | 2.93229 | 0.55262 | 0.80247 | 0.653932 | Not Significant | NS | #N/A |
| 2478 | G1SZN0 | #N/A | #N/A | RABBIT | #N/A | #N/A | #N/A | 1.5379 | #N/A | 0.44913 | -0.53181 | -0.51349 | -0.64817 | 1.63305 | -1.46016 | -1.36667 | -1.50672 | 0.24351 | 0.17602 | 0.29685 | 0.26731 | 4 | 2.93229 | 0.55262 | 0.95124 | 0.782627 | Not Significant | NS | #N/A |
| 1204 | A0A5F9C4X8 | 5'-3' exoribonuclease OS=Oryctolagus cuniculus O | XRN2 | RABBIT | -0.0742 | 0.66972 | #N/A | -0.05764 | #N/A | #N/A | #N/A | -2.66745 | 0.09289 | #N/A | -1.46657 | -1.36255 | -1.62602 | 0.26629 | 0.15563 | 0.30603 | 0.25326 | 3 | 2.93229 | 0.55262 | 1.39021 | 0.976196 | Not Significant | NS | #N/A |
| 79 | B8K132 | Alpha-hemoglobin (Predicted) OS=Oryctolagus cun | HBA | RABBIT | -0.94012 | -0.95887 | 0.47342 | #N/A | #N/A | 0.83229 | 0.6356 | 0.51696 | -11.4362 | -0.30277 | -1.47564 | -0.46242 | -0.56026 | 0.66008 | 0.58908 | 0.47104 | 0.4724 | 6 | 2.93229 | 0.55262 | 19.0939 | 13.00721 | Not Significant | NS | #N/A |
| 455 | G1SLD6 | Histidine--tRNA ligase, cytoplasmic OS=Oryctolagu | HARS1 | RABBIT | 2.29898 | 3.03478 | 0.39831 | 1.81498 | -0.40917 | #N/A | -0.22766 | -1.99131 | #N/A | -2.18287 | -2.89486 | -3.03295 | -3.43872 | 0.02301 | 0.00749 | 0.11776 | 0.08098 | 6 | 2.93229 | 0.55262 | 1.70815 | 1.328812 | Significant | Down mtub | HARS1 |
| 1745 | A0A5F9D4A4 | #N/A | HNRNPR | RABBIT | #N/A | #N/A | 0.66908 | 1.43989 | #N/A | 1.50488 | -1.97624 | -0.71881 | #N/A | -0.49843 | -1.47663 | -1.33556 | -1.57351 | 0.25262 | 0.16002 | 0.30043 | 0.25503 | 4 | 2.93229 | 0.55262 | 1.62988 | 1.174209 | Not Significant | NS | #N/A |
| 814 | G1SHQ6 | Beta-synuclein OS=Oryctolagus cuniculus OX=998 | #N/A | RABBIT | 0.16618 | 1.0419 | #N/A | -0.05764 | -2.20437 | #N/A | #N/A | #N/A | #N/A | -1.74025 | -1.47677 | -0.95842 | -1.18921 | 0.40856 | 0.27977 | 0.37454 | 0.33427 | 3 | 2.93229 | 0.55262 | 1.89935 | 1.233668 | Not Significant | NS | #N/A |
| 726 | G1T112 | CD166 antigen OS=Oryctolagus cuniculus OX=998 | ALCAM | RABBIT | 0.01561 | 1.38811 | 1.66578 | 1.54894 | -0.01733 | #N/A | -2.54762 | -2.36998 | -0.84659 | #N/A | -2.84162 | -4.43618 | -4.65672 | 0.00439 | 0.00122 | 0.0684 | 0.04432 | 6 | 2.93229 | 0.55262 | 0.76933 | 0.698189 | Significant | Down mtub | ALCAM |
| 802 | A0A5F9CG70 | Mono-ADP ribosylhydrolase 2 OS=Oryctolagus cuni | MACROD2 | RABBIT | 0.92904 | 1.71906 | -0.12883 | 1.37632 | 1.41161 | -4.16651 | 0.25981 | -2.73094 | -1.84035 | -0.25981 | -2.809 | -3.22656 | -3.58506 | 0.01212 | 0.00432 | 0.09752 | 0.06392 | 8 | 2.93229 | 0.55262 | 1.8948 | 1.534796 | Significant | Down mtub | MACROD2 |
| 652 | G1SDD0 | Enoyl-CoA hydratase, mitochondrial OS=Oryctolag | ECHS1 | RABBIT | 0.77035 | 2.38257 | #N/A | 0.44947 | -0.87417 | #N/A | -2.2398 | 0.41375 | -0.47812 | -0.88677 | -1.47979 | -1.70561 | -1.91627 | 0.13896 | 0.08782 | 0.22123 | 0.1893 | 6 | 2.93229 | 0.55262 | 1.50547 | 1.192665 | Not Significant | NS | #N/A |
| 882 | Q28619 | Na(+)/H(+) exchange regulatory cofactor NHE-RF1 ( | NHERF1 N | RABBIT | -0.78711 | 3.1678 | 0.34574 | 2.08448 | #N/A | #N/A | #N/A | -0.39265 | #N/A | -0.17509 | -1.4866 | -1.12237 | -1.36415 | 0.32452 | 0.21515 | 0.33479 | 0.29254 | 4 | 2.93229 | 0.55262 | 2.33913 | 1.583455 | Not Significant | NS | #N/A |
| 13 | G1T7R2 | 14-3-3 protein epsilon OS=Oryctolagus cuniculus C | YWHAE | RABBIT | 1.26206 | 1.95738 | 0.00807 | 0.7002 | 0.5929 | -4.58522 | 0.16258 | -0.76903 | -0.65243 | -3.67982 | -2.8089 | -2.83405 | -3.18426 | 0.02201 | 0.00876 | 0.11776 | 0.0849 | 8 | 2.93229 | 0.55262 | 2.45583 | 1.945345 | Significant | Down mtub | YWHAE |
| 366 | G1T894 | Bridging integrator 1 OS=Oryctolagus cuniculus OX | BIN1 | RABBIT | 2.10584 | 0.2982 | -1.18399 | 0.46617 | -0.09414 | -2.3612 | #N/A | 0.01639 | #N/A | -1.1961 | -1.49872 | -1.7271 | -1.93071 | 0.13489 | 0.08582 | 0.21795 | 0.18814 | 6 | 2.93229 | 0.55262 | 1.4119 | 1.129813 | Not Significant | NS | #N/A |
| 828 | O77706 | calcium/calmodulin-dependent protein kinase (Fra | #N/A | RABBIT | -0.3336 | #N/A | 2.07924 | #N/A | 1.35387 | -1.37288 | -1.92478 | 1.26163 | -0.63948 | 0.34526 | -1.49922 | -1.61552 | -1.82446 | 0.15733 | 0.10163 | 0.23659 | 0.20137 | 6 | 2.93229 | 0.55262 | 1.61477 | 1.266085 | Not Significant | NS | #N/A |
| 1021 | A0A5F9CTV6 | Cartilage acidic protein 1 OS=Oryctolagus cuniculu | CRTAC1 | RABBIT | 1.03245 | 1.86678 | -0.19343 | 4.14831 | 0.3507 | -1.53629 | #N/A | #N/A | -1.22305 | -1.2542 | -2.77881 | -2.73687 | -3.12796 | 0.03388 | 0.01227 | 0.13313 | 0.09479 | 6 | 2.93229 | 0.55262 | 1.9329 | 1.479779 | Significant | Down mtub | CRTAC1 |
| 273 | A0A5F9C1Q6 | Limbic system associated membrane protein OS=O | LSAMP | RABBIT | 2.32276 | 1.97456 | 0.3437 | 1.87892 | 1.01022 | -2.73141 | -0.49339 | -1.1459 | -0.17304 | -1.40995 | -2.69677 | -4.70582 | -4.92679 | 0.00153 | 0.00046 | 0.05271 | 0.02567 | 8 | 2.93229 | 0.55262 | 0.82103 | 0.749033 | Significant | Down mtub | LSAMP |
| 1150 | G1SMU8 | Prefoldin subunit 4 OS=Oryctolagus cuniculus OX= | #N/A | RABBIT | 0.49267 | 1.62305 | #N/A | -0.53864 | 2.0233 | #N/A | -1.31736 | -2.24984 | #N/A | #N/A | -2.68369 | -2.93636 | -3.31019 | 0.04254 | 0.01312 | 0.14534 | 0.09655 | 4 | 2.93229 | 0.55262 | 1.11374 | 0.876391 | Significant | Down mtub | #N/A |
| 1851 | A0A0B4J1Q4 | #N/A | TXLNA | RABBIT | #N/A | 0.5257 | -0.13704 | 1.06156 | #N/A | #N/A | #N/A | #N/A | -2.05296 | 0 | -1.50989 | -1.70346 | -1.90997 | 0.18703 | 0.10527 | 0.26045 | 0.20407 | 3 | 2.93229 | 0.55262 | 0.94277 | 0.749922 | Not Significant | NS | #N/A |
| 2802 | A0A5F9CVK2 | #N/A | HK2 | RABBIT | #N/A | #N/A | #N/A | #N/A | 1.16925 | 0.37944 | 1.11465 | -1.78815 | -0.37944 | -1.03091 | -1.51013 | -1.20795 | -1.38883 | 0.2936 | 0.20787 | 0.32101 | 0.28797 | 4 | 2.93229 | 0.55262 | 1.30241 | 0.985256 | Not Significant | NS | #N/A |
| 314 | G1TB17 | Adenylosuccinate lyase OS=Oryctolagus cuniculus | ADSL | RABBIT | 2.00153 | 2.16667 | -1.31531 | 1.09321 | 0.83593 | #N/A | 0 | -1.36671 | -0.58516 | -0.26778 | -1.51132 | -2.00921 | -2.1994 | 0.08446 | 0.05267 | 0.17453 | 0.15178 | 7 | 2.93229 | 0.55262 | 1.25732 | 1.049274 | Not Significant | NS | #N/A |

| | | | | | | | | | | | | | | | | | | | | | | | | | | | | | |
|---|---|---|---|---|---|---|---|---|---|---|---|---|---|---|---|---|---|---|---|---|---|---|---|---|---|---|---|---|---|
| 1936 | G1T3V0 | #N/A | HDLBP | RABBIT | #N/A | #N/A | 4.03118 | -0.2278 | #N/A | 0.28145 | -0.62046 | -0.06437 | -0.52613 | 2.87712 | -1.51217 | -0.97071 | -1.16922 | 0.37628 | 0.27625 | 0.35866 | 0.33229 | 5 | 2.93229 | 0.55262 | 3.46675 | 2.389497 | Not Significant | NS | #N/A |
| 2823 | G1SQS0 | #N/A | PDCD11 | RABBIT | #N/A | #N/A | #N/A | #N/A | 1.09197 | #N/A | 0 | -0.8512 | #N/A | #N/A | -1.51757 | -2.05866 | -1.74501 | 0.28787 | 0.15716 | 0.31864 | 0.25435 | 1 | 2.93229 | 0.55262 | 0.36227 | 0.504212 | Not Significant | NS | #N/A |
| 333 | G1ST69 | Lamin B1 OS=Oryctolagus cuniculus OX=9986 GN= | LMNB1 | RABBIT | -0.48428 | 1.16671 | 0.8819 | -0.83492 | 0.8854 | -5.20293 | -0.01595 | 0.49771 | -1.29837 | 0.01595 | -1.52368 | -1.35932 | -1.54015 | 0.21112 | 0.15195 | 0.27499 | 0.25099 | 8 | 2.93229 | 0.55262 | 3.14111 | 2.446816 | Not Significant | NS | #N/A |
| 2324 | A0A5F9CNJ0 | #N/A | PELI1 | RABBIT | #N/A | #N/A | 0.81514 | #N/A | #N/A | #N/A | #N/A | -0.70885 | #N/A | #N/A | -1.52399 | #N/A | -1.44962 | #N/A | 0.24503 | #N/A | 0.31493 | 0 | 2.93229 | 0.55262 | #N/A | 0.552617 | Not Significant | NS | #N/A |
| 1002 | G1U684 | Cell adhesion molecule 1 OS=Oryctolagus cuniculu | CADM1 | RABBIT | #N/A | 1.83698 | -0.6357 | 1.29778 | #N/A | #N/A | 0 | -1.38805 | #N/A | #N/A | -1.52705 | -1.39007 | -1.66822 | 0.25869 | 0.14688 | 0.30309 | 0.24654 | 3 | 2.93229 | 0.55262 | 1.44814 | 1.00549 | Not Significant | NS | #N/A |
| 629 | A0A5F9DDA3 | PTPRF interacting protein alpha 2 OS=Oryctolagus c | PPFIA2 | RABBIT | 0.61997 | #N/A | 0.52807 | #N/A | #N/A | -1.55991 | #N/A | #N/A | #N/A | -0.35742 | -1.53268 | -2.54175 | -2.22156 | 0.12615 | 0.07772 | 0.21251 | 0.18205 | 2 | 2.93229 | 0.55262 | 0.36361 | 0.475977 | Not Significant | NS | #N/A |
| 2466 | G1T6E1 | #N/A | CFAP54 | RABBIT | #N/A | #N/A | #N/A | #N/A | 2.32683 | #N/A | 0.30066 | #N/A | -0.96504 | -0.30066 | -2.64851 | -3.62286 | -3.27311 | 0.06846 | 0.02257 | 0.16349 | 0.11217 | 2 | 2.93229 | 0.55262 | 0.40083 | 0.491069 | Significant | Down mtub | CFAP54 |
| 2797 | A0A5F9CEQ5 | #N/A | RYR3 | RABBIT | #N/A | #N/A | #N/A | #N/A | 0.76264 | #N/A | -0.77603 | #N/A | #N/A | #N/A | -1.53867 | #N/A | -1.46359 | #N/A | 0.24152 | #N/A | 0.31205 | 0 | 2.93229 | 0.55262 | #N/A | 0.552617 | Not Significant | NS | #N/A |
| 450 | G1STP6 | Catenin alpha-1 OS=Oryctolagus cuniculus OX=998 | CTNNA1 | RABBIT | 0.19895 | #N/A | #N/A | -0.05764 | #N/A | #N/A | #N/A | #N/A | -2.57607 | #N/A | -2.64673 | -11.9108 | -3.33276 | 0.05332 | 0.02979 | 0.15348 | 0.12188 | 1 | 2.93229 | 0.55262 | 0.03292 | 0.420455 | Significant | Down mtub | CTNNA1 |
| 1871 | G1U3S9 | #N/A | GJA3 | RABBIT | #N/A | 1.79177 | #N/A | 0.40863 | -0.47965 | #N/A | -0.97731 | #N/A | #N/A | #N/A | -1.55089 | -1.17337 | -1.44846 | 0.36147 | 0.20793 | 0.35236 | 0.28797 | 2 | 2.93229 | 0.55262 | 1.31024 | 0.859826 | Not Significant | NS | #N/A |
| 2046 | G1SX12 | #N/A | ARAP3 | RABBIT | #N/A | 1.48751 | #N/A | #N/A | #N/A | #N/A | #N/A | #N/A | 0 | -0.13218 | -1.5536 | -13.5724 | -1.97076 | 0.04682 | 0.12128 | 0.14885 | 0.21983 | 1 | 2.93229 | 0.55262 | 0.00874 | 0.414305 | Not Significant | NS | #N/A |
| 1035 | G1SJ72 | Chondroitin sulfate proteoglycan 4 OS=Oryctolagus | CSPG4 | RABBIT | 0.40078 | #N/A | #N/A | 1.01417 | 1.72808 | #N/A | -3.93384 | #N/A | -0.38912 | -0.4639 | -2.64329 | -2.14797 | -2.60497 | 0.0982 | 0.03547 | 0.18746 | 0.12804 | 4 | 2.93229 | 0.55262 | 2.27156 | 1.544466 | Significant | Down mtub | CSPG4 |
| 1770 | G1SDF4 | #N/A | KRT82 | RABBIT | #N/A | 2.50502 | 2.43996 | #N/A | -0.31976 | 0.30638 | #N/A | -2.68169 | #N/A | -0.85829 | -2.61961 | -2.05641 | -2.50663 | 0.10889 | 0.04092 | 0.19697 | 0.13388 | 4 | 2.93229 | 0.55262 | 2.43412 | 1.638265 | Significant | Down mtub | KRT82 |
| 818 | G1SYC6 | HDGF like 3 OS=Oryctolagus cuniculus OX=9986 G | HDGFL3 | RABBIT | 0.43324 | -0.14027 | 0.44434 | 0.9271 | 0.14319 | #N/A | -3.00142 | -0.22094 | -3.44637 | #N/A | -2.58443 | -3.33842 | -3.65743 | 0.01564 | 0.00532 | 0.10396 | 0.06762 | 6 | 2.93229 | 0.55262 | 1.1237 | 0.936222 | Significant | Down mtub | HDGFL3 |
| 441 | G1SNI4 | Dimethylarginine dimethylaminohydrolase 1 OS=O | DDAH1 | RABBIT | 2.27402 | 3.89283 | 0.93635 | 1.33414 | 0.16645 | -0.26763 | -0.68261 | -2.34717 | -0.79856 | -0.17983 | -2.57592 | -3.43227 | -3.75153 | 0.00892 | 0.00324 | 0.09045 | 0.05702 | 8 | 2.93229 | 0.55262 | 1.40812 | 1.178657 | Significant | Down mtub | DDAH1 |
| 2021 | G1TA82 | #N/A | WASHC5 | RABBIT | #N/A | 0.85799 | #N/A | #N/A | #N/A | #N/A | #N/A | -0.72453 | #N/A | #N/A | -1.58252 | #N/A | -1.5053 | #N/A | 0.23135 | #N/A | 0.30329 | 0 | 2.93229 | 0.55262 | #N/A | 0.552617 | Not Significant | NS | #N/A |
| 1306 | A0A5F9CV02 | Nuclear factor 1 OS=Oryctolagus cuniculus OX=99 | NFIA | RABBIT | #N/A | #N/A | 1.55719 | #N/A | 0.79217 | #N/A | #N/A | -0.06437 | -1.8897 | -2.24071 | -2.57294 | -2.80771 | -3.1857 | 0.06742 | 0.01924 | 0.1625 | 0.10772 | 3 | 2.93229 | 0.55262 | 1.00771 | 0.782761 | Significant | Down mtub | NFIA |
| 345 | A0A5F9D7C9 | L1 cell adhesion molecule OS=Oryctolagus cunicul | L1CAM | RABBIT | 0.13939 | 0.12298 | 0.01967 | 1.43516 | #N/A | -4.93034 | -0.32778 | #N/A | 0.07484 | 0.54949 | | -1.20852 | -1.42005 | 0.27231 | 0.18954 | 0.30973 | 0.27623 | 6 | 2.93229 | 0.55262 | 3.45211 | 2.500268 | Not Significant | NS | #N/A |
| 399 | A0A5F9DH52 | Cullin 1 OS=Oryctolagus cuniculus OX=9986 GN=C | CUL1 | RABBIT | 1.53062 | #N/A | -0.16053 | #N/A | 0.3178 | -2.0055 | #N/A | #N/A | #N/A | #N/A | -2.56813 | -2.55125 | -2.7873 | 0.12538 | 0.03915 | 0.21204 | 0.13135 | 2 | 2.93229 | 0.55262 | 0.75996 | 0.636692 | Significant | Down mtub | CUL1 |
| 140 | G1T140 | Protein kinase C and casein kinase substrate in neu | PACSIN1 | RABBIT | 1.54167 | 0.55173 | 0.03963 | 0.54832 | 0.6426 | -3.92903 | -0.00805 | -0.05632 | -0.55159 | -0.0776 | -1.5893 | -1.99729 | -2.1984 | 0.08085 | 0.05037 | 0.1731 | 0.15072 | 8 | 2.93229 | 0.55262 | 1.58296 | 1.3066 | Not Significant | NS | #N/A |
| 364 | A0A5F9D3K0 | Cullin 4B OS=Oryctolagus cuniculus OX=9986 GN= | CUL4B | RABBIT | -0.16263 | 3.00594 | #N/A | -0.05764 | #N/A | -2.05033 | #N/A | 0.65126 | 0.46819 | -1.71581 | -1.59023 | -1.31585 | -1.55951 | 0.24532 | 0.15782 | 0.29719 | 0.25465 | 5 | 2.93229 | 0.55262 | 2.50376 | 1.782489 | Not Significant | NS | #N/A |
| 1720 | G1SQI2 | #N/A | PRPH | RABBIT | #N/A | 2.5048 | #N/A | #N/A | 0.16777 | #N/A | #N/A | -0.33223 | #N/A | -0.18115 | -1.59297 | -1.3604 | -1.69377 | 0.30674 | 0.15189 | 0.32551 | 0.25099 | 2 | 2.93229 | 0.55262 | 1.37114 | 0.88452 | Not Significant | NS | #N/A |
| 489 | A0A5F9CW89 | Capping protein regulator and myosin 1 linker 1 OS= | CARMIL1 | RABBIT | 0.71497 | 1.03402 | 1.7481 | 1.40542 | 1.17265 | -2.06653 | -1.99014 | -0.76768 | -0.82451 | -1.11519 | -2.56784 | -7.74485 | -6.8694 | 5.5E-05 | 2.8E-05 | 0.02014 | 0.01186 | 8 | 2.93229 | 0.55262 | 0.27482 | 0.349332 | Significant | Down mtub | CARMIL1 |
| 432 | G1SP54 | Leukotriene A(4) hydrolase OS=Oryctolagus cunicu | LTA4H | RABBIT | 0.35665 | 2.77873 | #N/A | -0.05764 | #N/A | #N/A | 0.59936 | -2.97098 | -2.31375 | -1.4344 | -2.55585 | -2.16657 | -2.56051 | 0.0825 | 0.03385 | 0.17453 | 0.1262 | 5 | 2.93229 | 0.55262 | 2.38567 | 1.708055 | Significant | Down mtub | LTA4H |
| 1459 | A0A5F9D285 | KIF-binding protein OS=Oryctolagus cuniculus OX= | KIFBP | RABBIT | 0.01166 | #N/A | #N/A | #N/A | 0.99841 | #N/A | #N/A | #N/A | #N/A | -2.03895 | -2.54399 | -2.97699 | -2.83748 | 0.20631 | 0.04793 | 0.27238 | 0.14727 | 1 | 2.93229 | 0.55262 | 0.48684 | 0.53589 | Significant | Down mtub | KIFBP |
| 170 | G1T2X7 | Dipeptidyl aminopeptidase-like protein 6 OS=Oryct | DPP6 | RABBIT | 1.66581 | 1.991 | -0.12451 | 0.68112 | 1.03582 | -5.54245 | 0.29516 | -0.65917 | -1.01646 | -0.34002 | -2.50243 | -2.25547 | -2.55391 | 0.0541 | 0.02692 | 0.15365 | 0.11897 | 8 | 2.93229 | 0.55262 | 3.07745 | 2.400232 | Significant | Down mtub | DPP6 |
| 2660 | A0A5F9CS82 | #N/A | #N/A | RABBIT | #N/A | #N/A | #N/A | 0.6193 | #N/A | -2.88968 | #N/A | 0.56022 | -0.62458 | #N/A | -1.60398 | -0.79244 | -1.10703 | 0.51117 | 0.31933 | 0.42044 | 0.35646 | 2 | 2.93229 | 0.55262 | 3.07271 | 1.574491 | Not Significant | NS | #N/A |
| 1228 | G1T7D9 | Protein farnesyltransferase subunit beta OS=Oryctc | #N/A | RABBIT | #N/A | 0.31655 | #N/A | -0.05764 | #N/A | #N/A | #N/A | #N/A | -2.37197 | #N/A | -2.50142 | -7.71902 | -3.11504 | 0.08202 | 0.03653 | 0.17426 | 0.12876 | 1 | 2.93229 | 0.55262 | 0.07001 | 0.429887 | Significant | Down mtub | #N/A |
| 134 | G1SMX2 | Internexin neuronal intermediate filament protein al | INA | RABBIT | 0.29997 | 0.03958 | 1.09774 | -0.17533 | 1.42248 | -4.81631 | -0.05673 | 0.21679 | 0.05673 | -0.75808 | -1.60841 | -1.60866 | -1.80865 | 0.14636 | 0.09806 | 0.22839 | 0.19757 | 8 | 2.93229 | 0.55262 | 2.49921 | 1.977093 | Not Significant | NS | #N/A |
| 2529 | G1SZ34 | #N/A | CDC42BPI | RABBIT | #N/A | #N/A | #N/A | 1.34496 | #N/A | #N/A | #N/A | -0.58295 | -0.73517 | 0.51858 | -1.61147 | -2.03984 | -1.93842 | 0.17819 | 0.11108 | 0.25448 | 0.21045 | 2 | 2.93229 | 0.55262 | 0.46807 | 0.518335 | Not Significant | NS | #N/A |
| 2502 | G1TIB3 | #N/A | #N/A | RABBIT | #N/A | #N/A | #N/A | 2.30887 | #N/A | -0.50005 | 0 | 0.14778 | #N/A | -0.39465 | -2.4956 | -7.20209 | -3.93526 | 0.00552 | 0.00784 | 0.07337 | 0.08248 | 3 | 2.93229 | 0.55262 | 0.09606 | 0.321731 | Significant | Down mtub | #N/A |
| 137 | G1SQG5 | Malate dehydrogenase OS=Oryctolagus cuniculus ( | MDH1 | RABBIT | 1.60515 | 2.69522 | 0.20175 | 1.04051 | 0.0481 | -3.94134 | -0.0311 | -1.17504 | -1.40981 | -0.21593 | -2.47279 | -2.90516 | -3.22078 | 0.01974 | 0.00821 | 0.1141 | 0.08427 | 8 | 2.93229 | 0.55262 | 1.81124 | 1.473646 | Significant | Down mtub | MDH1 |
| 1612 | G1TC15 | non-specific serine/threonine protein kinase OS=Or | SMG1 | RABBIT | #N/A | 0.57793 | #N/A | #N/A | 1.06943 | -3.13981 | 0.80194 | #N/A | -0.38011 | -0.46761 | -1.62008 | -1.2872 | -1.55222 | 0.26746 | 0.16497 | 0.30672 | 0.26016 | 4 | 2.93229 | 0.55262 | 2.11211 | 1.45246 | Not Significant | NS | #N/A |
| 603 | P41982 | Superoxide dismutase [Mn], mitochondrial (Fragme | SOD2 | RABBIT | 3.05804 | 4.75797 | 0.15913 | 1.02097 | -1.17916 | #N/A | -0.93134 | -0.26316 | 0 | 0.96633 | -1.62043 | -1.30228 | -1.50155 | 0.23403 | 0.16432 | 0.29142 | 0.25966 | 7 | 2.93229 | 0.55262 | 3.44064 | 2.588014 | Not Significant | NS | #N/A |
| 1445 | A0A5F9DIH9 | UEV and lactate/malate dehyrogenase domains OS | UEVLD | RABBIT | 0.81746 | #N/A | #N/A | #N/A | #N/A | -0.8058 | #N/A | #N/A | #N/A | #N/A | -1.62326 | #N/A | -1.54405 | #N/A | 0.22232 | #N/A | 0.29903 | 0 | 2.93229 | 0.55262 | #N/A | 0.552617 | Not Significant | NS | #N/A |
| 566 | A0A5F9CQN2 | Xaa-Pro dipeptidase OS=Oryctolagus cuniculus OX | PEPD | RABBIT | 0.93761 | 1.53689 | #N/A | 0.21072 | -0.48293 | #N/A | -0.26835 | -1.88223 | #N/A | #N/A | -1.62586 | -1.97659 | -2.16164 | 0.11927 | 0.06782 | 0.20687 | 0.17137 | 4 | 2.93229 | 0.55262 | 0.90214 | 0.754295 | Not Significant | NS | #N/A |
| 1715 | A0A5F9C0Z4 | #N/A | PSAP | RABBIT | #N/A | 1.57411 | #N/A | #N/A | #N/A | #N/A | -0.0469 | -0.06437 | #N/A | #N/A | -1.62975 | -107.776 | -2.07282 | 0.00591 | 0.10809 | 0.07337 | 0.20706 | 1 | 2.93229 | 0.55262 | 0.00015 | 0.412122 | Not Significant | NS | #N/A |
| 422 | A0A5F9DBZ1 | Propionyl-CoA carboxylase beta chain, mitochondri | PCCB | RABBIT | 3.38728 | 1.48208 | -0.18416 | -0.05764 | 2.36411 | #N/A | -0.15459 | #N/A | -1.9833 | #N/A | -2.46728 | -1.97107 | -2.32036 | 0.10578 | 0.04916 | 0.19504 | 0.1497 | 5 | 2.93229 | 0.55262 | 2.23838 | 1.61521 | Significant | Down mtub | PCCB |
| 1062 | G1T1S7 | BCL2 associated athanogene 3 OS=Oryctolagus cu | BAG3 | RABBIT | 1.25017 | 1.41558 | #N/A | -1.61847 | #N/A | #N/A | #N/A | -1.28213 | #N/A | #N/A | -1.63122 | -0.82808 | -1.15014 | 0.49471 | 0.30278 | 0.41217 | 0.34911 | 2 | 2.93229 | 0.55262 | 2.91033 | 1.508651 | Not Significant | NS | #N/A |
| 1291 | G1SPT6 | Aspartoacylase OS=Oryctolagus cuniculus OX=998 | ASPA | RABBIT | #N/A | 1.39118 | #N/A | 2.54183 | 0.0933 | #N/A | -1.21565 | -0.17105 | -1.92524 | #N/A | -2.44609 | -2.8063 | -3.1732 | 0.0485 | 0.01584 | 0.14898 | 0.09934 | 4 | 2.93229 | 0.55262 | 1.13964 | 0.891335 | Significant | Down mtub | ASPA |
| 1621 | G1SIL9 | BCL6 corepressor like 1 OS=Oryctolagus cuniculus | BCORL1 | RABBIT | 2.14616 | 0.48973 | #N/A | -0.34955 | #N/A | #N/A | #N/A | #N/A | -0.87484 | #N/A | -1.63696 | -1.1163 | -1.43021 | 0.38042 | 0.21282 | 0.36082 | 0.29168 | 2 | 2.93229 | 0.55262 | 1.61279 | 0.982509 | Not Significant | NS | #N/A |
| 235 | A0A5F9DDG6 | Glutathione S-transferase OS=Oryctolagus cunicul | GSTM2 | RABBIT | 4.17145 | 3.73794 | -0.04015 | 0.15316 | -0.70423 | -1.38193 | -0.49757 | 0.36975 | -0.22613 | 0.84992 | -1.64083 | -1.49495 | -1.69162 | 0.17329 | 0.11899 | 0.25187 | 0.21763 | 8 | 2.93229 | 0.55262 | 3.0117 | 2.352121 | Not Significant | NS | #N/A |
| 1505 | A0A5F9CDE9 | Ubiquitin conjugation factor E4 OS=Oryctolagus cu | UBE4B | RABBIT | 2.40129 | #N/A | #N/A | #N/A | #N/A | 0.51872 | #N/A | -0.06437 | -0.23381 | -0.37616 | -2.4402 | -5.55377 | -3.68263 | 0.01151 | 0.01051 | 0.09732 | 0.09154 | 3 | 2.93229 | 0.55262 | 0.15444 | 0.351257 | Significant | Down mtub | UBE4B |
| 1802 | G1TBW1 | #N/A | #N/A | RABBIT | #N/A | 3.11204 | #N/A | -0.05764 | #N/A | #N/A | #N/A | #N/A | #N/A | -0.12298 | -1.65018 | -0.60115 | -1.03657 | 0.65542 | 0.35942 | 0.4707 | 0.37925 | 1 | 2.93229 | 0.55262 | 5.02342 | 1.689564 | Not Significant | NS | #N/A |
| 658 | A0A5F9CAT8 | Cystathionine beta-synthase OS=Oryctolagus cunic | CBS | RABBIT | 1.84425 | 1.76248 | 0.17065 | 0.65839 | 1.10626 | #N/A | -1.13142 | -1.27908 | -1.25652 | -1.64004 | -2.43517 | -6.48313 | -5.85724 | 0.00034 | 0.00016 | 0.03578 | 0.0188 | 7 | 2.93229 | 0.55262 | 0.31353 | 0.384114 | Significant | Down mtub | CBS |
| 1680 | G1TAN9 | #N/A | ACO1 | RABBIT | #N/A | 2.22671 | #N/A | 0.39462 | #N/A | 0 | #N/A | -0.85464 | #N/A | -0.18329 | -1.65664 | -2.1778 | -2.29678 | 0.11759 | 0.06186 | 0.20536 | 0.16343 | 3 | 2.93229 | 0.55262 | 0.69439 | 0.624312 | Not Significant | NS | #N/A |
| 2686 | G1SG07 | #N/A | LRRC8A | RABBIT | #N/A | #N/A | #N/A | 0.16843 | -0.01338 | -1.57942 | #N/A | #N/A | #N/A | #N/A | -1.65695 | -10.5231 | -2.09685 | 0.06032 | 0.10522 | 0.15554 | 0.20407 | 1 | 2.93229 | 0.55262 | 0.01653 | 0.416287 | Not Significant | NS | #N/A |
| 420 | G1SZS0 | NIF3-like protein 1 OS=Oryctolagus cuniculus OX=9 | #N/A | RABBIT | 0.89913 | 0.72225 | 0.2783 | -2.1176 | 0.43408 | #N/A | -0.10765 | -0.63082 | #N/A | -4.10598 | -1.65805 | -1.41181 | -1.63913 | 0.2077 | 0.13586 | 0.27264 | 0.23462 | 6 | 2.93229 | 0.55262 | 2.58608 | 1.918537 | Not Significant | NS | #N/A |
| 1776 | Q6X973 | #N/A | #N/A | RABBIT | #N/A | 1.53821 | #N/A | #N/A | #N/A | 0.11314 | #N/A | #N/A | -0.36869 | -0.11314 | -1.6611 | -5.96758 | -2.42435 | 0.02695 | 0.06048 | 0.12392 | 0.16242 | 2 | 2.93229 | 0.55262 | 0.05811 | 0.352099 | Not Significant | NS | #N/A |
| 191 | A0A5F9D9E8 | Gephyrin OS=Oryctolagus cuniculus OX=9986 GN= | GPHN | RABBIT | 0.48239 | 0.4495 | -0.10505 | 0.47154 | 0.38966 | -4.74391 | -0.853 | 0.04063 | -0.10499 | -0.97888 | -1.66564 | -1.88386 | -2.09626 | 0.09633 | 0.06014 | 0.1866 | 0.16242 | 8 | 2.93229 | 0.55262 | 1.95436 | 1.578384 | Not Significant | NS | #N/A |
| 1889 | A0A5F9DRA0 | #N/A | #N/A | RABBIT | #N/A | 0.19782 | 0.23262 | #N/A | #N/A | #N/A | #N/A | -1.45339 | #N/A | #N/A | -1.66861 | -55.3635 | -2.12196 | 0.0115 | 0.10231 | 0.09732 | 0.20162 | 1 | 2.93229 | 0.55262 | 0.00061 | 0.412238 | Not Significant | NS | #N/A |
| 557 | A0A5F9CLJ3 | Rap guanine nucleotide exchange factor 4 OS=Oryc | RAPGEF4 | RABBIT | 2.94448 | 1.67054 | #N/A | -0.19865 | -1.39225 | -1.88886 | -2.3148 | #N/A | 0.14101 | 0.37537 | -1.67785 | -1.41635 | -1.65055 | 0.20643 | 0.13348 | 0.27238 | 0.2333 | 6 | 2.93229 | 0.55262 | 2.80668 | 2.066714 | Not Significant | NS | #N/A |
| 1981 | A0A5F9CV07 | #N/A | ADA | RABBIT | #N/A | 0.19782 | #N/A | #N/A | -0.00073 | #N/A | -2.33551 | #N/A | #N/A | #N/A | -2.43405 | -14.1557 | -3.07728 | 0.0449 | 0.03788 | 0.14686 | 0.12938 | 1 | 2.93229 | 0.55262 | 0.01971 | 0.417096 | Significant | Down mtub | ADA |
| 261 | P62139 | Serine/threonine-protein phosphatase PP1-alpha ca | PPP1CA PI | RABBIT | 0.0909 | 2.99141 | 2.53683 | 3.02258 | -0.77199 | 0.5434 | -0.07924 | -2.41644 | -0.17203 | -2.17181 | -2.43317 | -2.43722 | -2.73989 | 0.04074 | 0.01933 | 0.14369 | 0.10772 | 8 | 2.93229 | 0.55262 | 2.49169 | 1.971589 | Significant | Down mtub | PPP1CA PPP1A |
| 1251 | G1TWC5 | U6 snRNA-associated Sm-like protein LSm3 OS=Or | LSM3 | RABBIT | 1.35584 | 3.09194 | #N/A | -0.61924 | #N/A | #N/A | -0.74487 | -0.06437 | #N/A | #N/A | -1.6808 | -1.19454 | -1.51623 | 0.3181 | 0.1808 | 0.33256 | 0.27126 | 3 | 2.93229 | 0.55262 | 2.37583 | 1.474627 | Not Significant | NS | #N/A |
| 2555 | A0A5F9CFR2 | #N/A | TRMT1 | RABBIT | #N/A | #N/A | #N/A | 2.4072 | 0.2966 | -0.35243 | #N/A | #N/A | #N/A | -0.30999 | -1.68311 | -1.59459 | -1.90538 | 0.25185 | 0.11586 | 0.30012 | 0.21429 | 2 | 2.93229 | 0.55262 | 1.11411 | 0.780296 | Not Significant | NS | #N/A |
| 1701 | A0A5F9CIC6 | #N/A | AKR1A1 | RABBIT | #N/A | 0.19782 | #N/A | 1.94024 | 1.60992 | -1.32398 | -2.76945 | 1.63268 | -2.2057 | #N/A | -2.41594 | -1.94515 | -2.31241 | 0.10935 | 0.04977 | 0.19697 | 0.14997 | 5 | 2.93229 | 0.55262 | 2.64453 | 1.871224 | Significant | Down mtub | AKR1A1 |
| 277 | G1SD02 | COP9 signalosome complex subunit 6 OS=Oryctola | COPS6 | RABBIT | 0.34459 | -0.12208 | 0.49332 | 0.66797 | 0.30652 | -4.66803 | 0.47735 | -0.59889 | -1.14101 | -0.79602 | -1.68338 | -1.90552 | -2.12019 | 0.09317 | 0.0577 | 0.18402 | 0.15957 | 8 | 2.93229 | 0.55262 | 1.95111 | 1.576003 | Not Significant | NS | #N/A |
| 782 | A0A5F9DLJ2 | Homer scaffold protein 2 OS=Oryctolagus cuniculu | HOMER2 | RABBIT | 1.98846 | -0.68433 | -0.34046 | 2.83007 | 1.09333 | #N/A | 0.51111 | -1.19186 | -1.43898 | #N/A | -1.68399 | -1.68617 | -1.92317 | 0.14274 | 0.08686 | 0.22477 | 0.18876 | 6 | 2.93229 | 0.55262 | 1.87014 | 1.437626 | Not Significant | NS | #N/A |
| 20 | G1SDY5 | 14-3-3 protein zeta/delta OS=Oryctolagus cuniculu | YWHAZ | RABBIT | 2.06982 | 2.43122 | 0.03853 | 1.48702 | 0.35754 | -3.43324 | 0.13212 | -0.75939 | -1.06759 | -0.54294 | -2.41103 | -3.1432 | -3.44483 | 0.01374 | 0.00553 | 0.09752 | 0.06816 | 8 | 2.93229 | 0.55262 | 1.47097 | 1.224644 | Significant | Down mtub | YWHAZ |
| 2376 | A0A375YJK3 | #N/A | #N/A | MT | #N/A | #N/A | 0.93048 | #N/A | #N/A | -0.75983 | #N/A | #N/A | #N/A | #N/A | -1.6903 | #N/A | -1.60782 | #N/A | 0.20832 | #N/A | 0.28804 | 0 | 2.93229 | 0.55262 | #N/A | 0.552617 | Not Significant | NS | #N/A |
| 2817 | A0A5F9CUH0 | #N/A | CCDC174 | RABBIT | #N/A | #N/A | #N/A | #N/A | 0.83889 | -0.85227 | #N/A | #N/A | #N/A | #N/A | -1.69116 | #N/A | -1.60864 | #N/A | 0.20814 | #N/A | 0.28803 | 0 | 2.93229 | 0.55262 | #N/A | 0.552617 | Not Significant | NS | #N/A |
| 1830 | A0A5F9DQF6 | #N/A | RNF40 | RABBIT | #N/A | 0.97392 | #N/A | #N/A | #N/A | 0 | #N/A | -1.43746 | #N/A | #N/A | -1.69265 | -1.35969 | -1.68239 | 0.4037 | 0.16901 | 0.37287 | 0.26263 | 1 | 2.93229 | 0.55262 | 1.03314 | 0.674817 | Not Significant | NS | #N/A |
| 1101 | A0A5F9D6J6 | Myosin phosphatase Rho interacting protein OS=Or | MPRIP | RABBIT | #N/A | 1.52875 | #N/A | #N/A | 0.91925 | #N/A | #N/A | -1.37626 | #N/A | -0.93264 | -2.37845 | -6.3101 | -3.82753 | 0.02421 | 0.0126 | 0.11931 | 0.09521 | 2 | 2.93229 | 0.55262 | 0.14207 | 0.386145 | Significant | Down mtub | MPRIP |
| 1115 | A0A5F9DN73 | Filensin OS=Oryctolagus cuniculus OX=9986 GN=B | BFSP1 | RABBIT | 2.12269 | #N/A | #N/A | #N/A | #N/A | 0.43229 | -0.75862 | #N/A | #N/A | -0.43229 | -2.37557 | -3.34308 | -2.96299 | 0.07902 | 0.03194 | 0.17277 | 0.12464 | 2 | 2.93229 | 0.55262 | 0.37871 | 0.482097 | Significant | Down mtub | BFSP1 |
| 1134 | A5TZI0 | HNH endonuclease OS=Mycobacterium tuberculos | #N/A | MT | 0.8238 | #N/A | #N/A | #N/A | #N/A | #N/A | #N/A | -0.8765 | #N/A | #N/A | -1.7003 | #N/A | -1.61733 | #N/A | 0.20631 | #N/A | 0.28712 | 0 | 2.93229 | 0.55262 | #N/A | 0.552617 | Not Significant | NS | #N/A |
| 2423 | P9WPS3 | #N/A | #N/A | MT | #N/A | #N/A | 0.93987 | #N/A | #N/A | -0.76922 | #N/A | #N/A | #N/A | #N/A | -1.7091 | #N/A | -1.6257 | #N/A | 0.20457 | #N/A | 0.28585 | 0 | 2.93229 | 0.55262 | #N/A | 0.552617 | Not Significant | NS | #N/A |
| 1811 | G1SH53 | #N/A | GGT7 | RABBIT | #N/A | 1.22856 | 0.17065 | #N/A | 2.59055 | -2.01325 | -2.35022 | -0.76015 | #N/A | 1.06699 | -2.34408 | -2.15704 | -2.52248 | 0.0835 | 0.03591 | 0.17453 | 0.12868 | 5 | 2.93229 | 0.55262 | 2.02446 | 1.480372 | Significant | Down mtub | GGT7 |
| 1781 | A0A5F9DLT0 | #N/A | #N/A | RABBIT | #N/A | 2.98369 | 0.12365 | #N/A | #N/A | #N/A | #N/A | -0.36389 | #N/A | 0.047 | -1.71212 | -1.1851 | -1.57958 | 0.35771 | 0.17584 | 0.35078 | 0.26731 | 2 | 2.93229 | 0.55262 | 2.08716 | 1.17486 | Not Significant | NS | #N/A |
| 1362 | A0A5F9D217 | Serpin family E member 2 OS=Oryctolagus cuniculu | SERPINE2 | RABBIT | 0.01166 | #N/A | #N/A | 2.40054 | #N/A | #N/A | #N/A | -0.51063 | #N/A | #N/A | -1.71673 | -0.82981 | -1.31414 | 0.55904 | 0.26021 | 0.43643 | 0.32441 | 1 | 2.93229 | 0.55262 | 2.85337 | 1.137709 | Not Significant | NS | #N/A |
| 609 | A0A5F9C6I2 | E3 ubiquitin-protein ligase RNF123 OS=Oryctolagus | RNF123 KI | RABBIT | 0.33866 | 1.39018 | 2.39537 | 0.6517 | 0.59855 | -1.25045 | -2.66618 | -1.05062 | -0.32699 | -1.02696 | -2.33913 | -4.3674 | -4.50831 | 0.00239 | 0.0009 | 0.05406 | 0.036 | 8 | 2.93229 | 0.55262 | 0.71714 | 0.67301 | Significant | Down mtub | RNF123 KPC1 |
| 1176 | G1TB49 | NAD(P)H-hydrate epimerase OS=Oryctolagus cunic | APOA1BP / | RABBIT | #N/A | 2.33073 | #N/A | #N/A | #N/A | #N/A | -0.01553 | #N/A | #N/A | 0 | -2.33849 | -173.909 | -2.97428 | 0.00366 | 0.04186 | 0.06691 | 0.13567 | 1 | 2.93229 | 0.55262 | 0.00012 | 0.412114 | Significant | Down mtub | APOA1BP AIBP NAXE |
| 827 | U3KN73 | Vacuolar protein sorting-associated protein 29 OS= | VPS29 | RABBIT | 1.6379 | 1.43551 | 2.36586 | -0.70712 | 2.26077 | 0.22698 | -3.12686 | -0.29135 | -0.84641 | -0.61259 | -2.32863 | -2.90489 | -3.20003 | 0.01974 | 0.00852 | 0.1141 | 0.08441 | 8 | 2.93229 | 0.55262 | 1.6065 | 1.323826 | Significant | Down mtub | VPS29 |
| 464 | G1SNZ0 | protein-serine/threonine phosphatase OS=Oryctola | PPM1B | RABBIT | 1.44804 | 2.54145 | #N/A | 0.98297 | #N/A | -0.33669 | #N/A | -1.10391 | -1.21981 | 0 | -2.32259 | -4.45405 | -4.30875 | 0.00668 | 0.00264 | 0.07628 | 0.05319 | 5 | 2.93229 | 0.55262 | 0.46614 | 0.498108 | Significant | Down mtub | PPM1B |
| 2782 | A0A5F9C750 | #N/A | AMPH | RABBIT | #N/A | #N/A | #N/A | #N/A | 0.86143 | -0.87481 | #N/A | #N/A | #N/A | #N/A | -1.73624 | #N/A | -1.65151 | #N/A | 0.19929 | #N/A | 0.28306 | 0 | 2.93229 | 0.55262 | #N/A | 0.552617 | Not Significant | NS | #N/A |
| 841 | A0A5F9D1V0 | phosphoribosylformylglycinamidine synthase OS=( | PFAS | RABBIT | 1.88279 | 3.03055 | 0.66217 | 2.20979 | -0.71213 | -1.48048 | -2.57234 | -0.55589 | -0.68535 | 0.9163 | -2.29019 | -2.6317 | -2.92399 | 0.0301 | 0.01392 | 0.12757 | 0.09782 | 8 | 2.93229 | 0.55262 | 1.89326 | 1.533669 | Significant | Down mtub | PFAS |
| 2106 | G1SF64 | #N/A | CELF1 | RABBIT | #N/A | #N/A | #N/A | 1.45817 | #N/A | 0.0215 | #N/A | #N/A | -0.85334 | -0.0215 | -1.74262 | -3.06027 | -2.30911 | 0.09224 | 0.0697 | 0.18325 | 0.17249 | 2 | 2.93229 | 0.55262 | 0.24319 | 0.427147 | Not Significant | NS | #N/A |
| 153 | Q8HZM9 | Serine/threonine-protein phosphatase OS=Oryctola | #N/A | RABBIT | 2.80965 | 2.6079 | 0.05238 | 0.50956 | 0.18788 | -3.48159 | -0.58008 | -0.44459 | 0.11827 | -0.87872 | -2.28681 | -2.61629 | -2.9081 | 0.03083 | 0.01432 | 0.12916 | 0.09913 | 8 | 2.93229 | 0.55262 | 1.90998 | 1.545904 | Significant | Down mtub | #N/A |
| 1559 | G1TD26 | Transmembrane protein 33 OS=Oryctolagus cunicu | TMEM33 | RABBIT | 1.878 | 0.5139 | #N/A | #N/A | #N/A | -0.78729 | #N/A | #N/A | #N/A | -0.31608 | -1.74764 | -2.4219 | -2.37894 | 0.13644 | 0.06395 | 0.21984 | 0.16637 | 2 | 2.93229 | 0.55262 | 0.5207 | 0.539677 | Not Significant | NS | #N/A |
| 1957 | A0A5F9C7G5 | #N/A | TRA2B | RABBIT | #N/A | #N/A | #N/A | 1.35918 | #N/A | #N/A | -0.49938 | -1.18685 | #N/A | 0.49938 | -1.7548 | -1.79233 | -1.92994 | 0.21495 | 0.11229 | 0.27765 | 0.21133 | 2 | 2.93229 | 0.55262 | 0.71892 | 0.62005 | Not Significant | NS | #N/A |
| 1023 | A0A5F9CNF8 | Transmembrane protein 132B OS=Oryctolagus cun | TMEM132I | RABBIT | 1.88369 | 1.95976 | -0.15229 | 2.82384 | 0.99218 | #N/A | 0 | -1.21258 | -0.60899 | -1.30934 | -2.28416 | -3.61688 | -3.83636 | 0.00855 | 0.00333 | 0.08862 | 0.05702 | 7 | 2.93229 | 0.55262 | 0.88629 | 0.787779 | Significant | Down mtub | TMEM132B |
| 121 | A0A5F9CAH6 | Alpha-2-macroglobulin OS=Oryctolagus cuniculus ( | A2M | RABBIT | 0.05653 | 0.15295 | 0.44995 | 0.44782 | #N/A | -2.25086 | #N/A | #N/A | -1.74965 | #N/A | -2.27707 | -10.545 | -5.06367 | 0.00046 | 0.0015 | 0.03578 | 0.04508 | 4 | 2.93229 | 0.55262 | 0.06217 | 0.269626 | Significant | Down mtub | A2M |
| 2793 | A0A5F9C6K7 | #N/A | MOG | RABBIT | #N/A | #N/A | #N/A | #N/A | 2.14739 | #N/A | -0.76381 | -0.06437 | 0.37425 | -0.04309 | -2.27164 | -4.30677 | -3.27156 | 0.02304 | 0.01728 | 0.11776 | 0.10217 | 3 | 2.93229 | 0.55262 | 0.22257 | 0.38571 | Significant | Down mtub | MOG |
| 2277 | A0A5F9CNM8 | #N/A | MAX | RABBIT | #N/A | #N/A | 0.97755 | #N/A | #N/A | -0.80689 | #N/A | #N/A | #N/A | #N/A | -1.78444 | #N/A | -1.69736 | #N/A | 0.1903 | #N/A | 0.27623 | 0 | 2.93229 | 0.55262 | #N/A | 0.552617 | Not Significant | NS | #N/A |
| 2581 | A0A5F9C1V4 | #N/A | USP2 | RABBIT | #N/A | #N/A | #N/A | #N/A | 0.8268 | -0.01338 | #N/A | -1.37898 | #N/A | #N/A | -1.78569 | -2.45415 | -2.05815 | 0.24633 | 0.10988 | 0.29787 | 0.20887 | 1 | 2.93229 | 0.55262 | 0.35295 | 0.501841 | Not Significant | NS | #N/A |
| 108 | A0A5F9CQS0 | Catenin beta-1 OS=Oryctolagus cuniculus OX=998 | CTNNB1 | RABBIT | 1.49564 | 0.66758 | 0.15862 | -0.02756 | 0.5673 | -4.71531 | -0.08687 | -0.06437 | #N/A | -1.90517 | -2.26524 | -2.24736 | -2.54948 | 0.05943 | 0.02903 | 0.15524 | 0.12009 | 7 | 2.93229 | 0.55262 | 2.25773 | 1.754334 | Significant | Down mtub | CTNNB1 |
| 1022 | A0A5F9DS66 | WD repeat-containing protein 37 OS=Oryctolagus c | WDR37 | RABBIT | 1.67553 | #N/A | #N/A | #N/A | #N/A | #N/A | 0 | -0.24 | #N/A | #N/A | -1.79553 | -8.63862 | -2.26375 | 0.07337 | 0.08747 | 0.16663 | 0.1893 | 1 | 2.93229 | 0.55262 | 0.0288 | 0.419408 | Not Significant | NS | #N/A |
| 2388 | G1TE30 | #N/A | ECD | RABBIT | #N/A | #N/A | 0.98564 | #N/A | #N/A | -0.81498 | #N/A | #N/A | #N/A | #N/A | -1.80062 | #N/A | -1.71275 | #N/A | 0.18738 | #N/A | 0.27558 | 0 | 2.93229 | 0.55262 | #N/A | 0.552617 | Not Significant | NS | #N/A |
| 799 | P41150 | Calcyphosin OS=Oryctolagus cuniculus OX=9986 ( | CAPS | RABBIT | 3.94439 | 3.18088 | -1.00264 | #N/A | #N/A | #N/A | 0 | -0.08771 | -0.99158 | 2.02366 | -1.80479 | -1.21064 | -1.46386 | 0.28013 | 0.1817 | 0.31436 | 0.27161 | 5 | 2.93229 | 0.55262 | 3.80983 | 2.605752 | Not Significant | NS | #N/A |
| 2207 | A0A5F9CMY1 | #N/A | #N/A | RABBIT | #N/A | #N/A | 2.3379 | #N/A | #N/A | 0 | #N/A | 1.17251 | -0.72662 | -0.10945 | -2.25379 | -2.54213 | -2.62186 | 0.08452 | 0.03991 | 0.17453 | 0.13235 | 3 | 2.93229 | 0.55262 | 0.62881 | 0.591149 | Significant | Down mtub | #N/A |
| 2530 | A0A5F9DN04 | #N/A | NAA15 | RABBIT | #N/A | #N/A | #N/A | 0.8787 | #N/A | -0.93634 | #N/A | #N/A | #N/A | #N/A | -1.81504 | #N/A | -1.72647 | #N/A | 0.18483 | #N/A | 0.27413 | 0 | 2.93229 | 0.55262 | #N/A | 0.552617 | Not Significant | NS | #N/A |

| | | | | | | | | | | | | | | | | | | | | | | | | | | | | | |
|---|---|---|---|---|---|---|---|---|---|---|---|---|---|---|---|---|---|---|---|---|---|---|---|---|---|---|---|---|---|
| 2720 | G1T1U6 | #N/A | #N/A | RABBIT | #N/A | #N/A | #N/A | 0.87923 | #N/A | -0.93687 | #N/A | #N/A | #N/A | #N/A | -1.8161 | #N/A | -1.72748 | #N/A | 0.18464 | #N/A | 0.27413 | 0 | 2.93229 | 0.55262 | #N/A | 0.552617 | Not Significant | NS | #N/A |
| 30 | G1TZP0 | 14-3-3 protein gamma OS=Oryctolagus cuniculus C | YWHAG | RABBIT | 1.6391 | 2.38616 | 0.08229 | 1.1182 | 0.24352 | -3.81039 | 0.08836 | -0.57759 | -1.18009 | -0.30491 | -2.25078 | -2.75305 | -3.03935 | 0.02494 | 0.01133 | 0.11996 | 0.09305 | 8 | 2.93229 | 0.55262 | 1.671 | 1.371023 | Significant | Down mtub | YWHAG |
| 1674 | O19049 | #N/A | HNRNPK H | RABBIT | #N/A | #N/A | 0.57212 | 1.18796 | -0.41485 | #N/A | #N/A | -1.3729 | #N/A | #N/A | -1.82131 | -1.95081 | -2.0472 | 0.19037 | 0.09676 | 0.26255 | 0.19688 | 2 | 2.93229 | 0.55262 | 0.65373 | 0.593619 | Not Significant | NS | #N/A |
| 642 | A0A5F9CWY9 | Aminotransferase class I/classII domain-containing | #N/A | RABBIT | 0.42617 | 0.75236 | #N/A | 0.21027 | -0.6159 | #N/A | #N/A | #N/A | -0.2679 | -2.99323 | -1.82379 | -1.93548 | -2.19933 | 0.12502 | 0.06417 | 0.21204 | 0.16669 | 4 | 2.93229 | 0.55262 | 1.18389 | 0.916869 | Not Significant | NS | #N/A |
| 786 | G1SHF3 | Nitrilase 1 OS=Oryctolagus cuniculus OX=9986 GN | NIT1 | RABBIT | 1.72137 | 2.68073 | #N/A | 0.43729 | -0.70947 | #N/A | -0.49493 | #N/A | #N/A | -1.09888 | -1.82939 | -1.62464 | -1.92097 | 0.17956 | 0.0966 | 0.25448 | 0.19688 | 4 | 2.93229 | 0.55262 | 1.69058 | 1.209231 | Not Significant | NS | #N/A |
| 249 | A0A5F9C295 | Proteasome subunit alpha type OS=Oryctolagus cu | PSMA6 | RABBIT | 0.48891 | 0.12828 | 0.249 | -0.00381 | 0.89268 | #N/A | -1.40373 | -2.89647 | -3.26805 | 0 | -2.24308 | -3.29269 | -3.54448 | 0.01325 | 0.00537 | 0.09752 | 0.06774 | 7 | 2.93229 | 0.55262 | 1.03127 | 0.889959 | Significant | Down mtub | PSMA6 |
| 266 | G1T0T5 | COP9 signalosome subunit 7A OS=Oryctolagus cur | COPS7A | RABBIT | 0.49588 | 0.60316 | 0.0883 | 0.10687 | 0.47618 | -5.87333 | 0.08235 | -1.18452 | -1.08854 | -1.3724 | -2.24136 | -2.16712 | -2.44253 | 0.0621 | 0.0328 | 0.15739 | 0.12553 | 8 | 2.93229 | 0.55262 | 2.67423 | 2.105163 | Significant | Down mtub | COPS7A |
| 549 | G1TLL9 | non-specific serine/threonine protein kinase OS=Or | PAK1 | RABBIT | 2.1824 | 3.48682 | -0.3756 | 1.12358 | -0.01338 | -0.11425 | #N/A | -2.13405 | 0.10944 | -1.56937 | -2.20782 | -2.35027 | -2.64767 | 0.05107 | 0.02454 | 0.15026 | 0.11483 | 7 | 2.93229 | 0.55262 | 1.96101 | 1.545211 | Significant | Down mtub | PAK1 |
| 756 | G1TL92 | Cadherin 13 OS=Oryctolagus cuniculus OX=9986 G | CDH13 | RABBIT | 1.61535 | 1.72084 | -0.03481 | 1.56816 | 0.82124 | #N/A | 0 | -2.14878 | -0.37675 | -1.6946 | -2.19319 | -3.72 | -3.88686 | 0.00746 | 0.00306 | 0.0819 | 0.05588 | 7 | 2.93229 | 0.55262 | 0.77242 | 0.707527 | Significant | Down mtub | CDH13 |
| 1644 | A0A5F9CLN0 | Myotubularin related protein 9 OS=Oryctolagus cun | MTMR9 | RABBIT | #N/A | #N/A | #N/A | 2.36127 | 0.38766 | -0.57376 | #N/A | #N/A | -0.40104 | #N/A | -1.86186 | -1.87958 | -2.18451 | 0.20093 | 0.0814 | 0.27128 | 0.18476 | 2 | 2.93229 | 0.55262 | 0.98124 | 0.72642 | Not Significant | NS | #N/A |
| 2882 | A0A375YUT8 | #N/A | #N/A | MT | #N/A | #N/A | #N/A | #N/A | 0.92433 | #N/A | #N/A | #N/A | -0.93771 | #N/A | -1.86204 | #N/A | -1.77118 | #N/A | 0.17678 | #N/A | 0.26786 | 0 | 2.93229 | 0.55262 | #N/A | 0.552617 | Not Significant | NS | #N/A |
| 760 | A0A5F9C7I9 | Methionine adenosyltransferase 2 subunit beta OS= | MAT2B | RABBIT | 1.08224 | 2.32229 | -1.02681 | 3.63566 | #N/A | -2.32861 | -1.21021 | -0.12489 | 0.26431 | 0 | -2.18323 | -2.12254 | -2.41235 | 0.07145 | 0.03669 | 0.16449 | 0.12876 | 7 | 2.93229 | 0.55262 | 2.35111 | 1.820144 | Significant | Down mtub | MAT2B |
| 691 | A0A5F9DLT8 | AP complex mu/sigma subunit domain-containing p | #N/A | RABBIT | 1.16587 | 1.22863 | -0.31187 | 1.27188 | -0.01338 | #N/A | 0.08205 | -0.38936 | -2.05421 | -3.69364 | -2.18201 | -2.57082 | -2.86234 | 0.03696 | 0.017 | 0.13648 | 0.10198 | 7 | 2.93229 | 0.55262 | 1.60087 | 1.291399 | Significant | Down mtub | #N/A |
| 1341 | A0A5F9DCA9 | non-specific serine/threonine protein kinase OS=Or | TNIK | RABBIT | #N/A | -0.68069 | 6.1991 | 1.02362 | -0.01338 | #N/A | -0.24367 | #N/A | #N/A | #N/A | -1.87583 | -0.53701 | -0.73508 | 0.62854 | 0.49032 | 0.46044 | 0.43948 | 3 | 2.93229 | 0.55262 | 9.76146 | 5.209596 | Not Significant | NS | #N/A |
| 1951 | A0A5F9DDE0 | #N/A | ACSL4 | RABBIT | #N/A | 2.00458 | #N/A | -0.05764 | #N/A | #N/A | -0.9047 | #N/A | #N/A | #N/A | -1.87817 | -1.05165 | -1.57102 | 0.48398 | 0.19248 | 0.40702 | 0.27803 | 1 | 2.93229 | 0.55262 | 2.12637 | 0.95283 | Not Significant | NS | #N/A |
| 1220 | G1SSL0 | Endoplasmic reticulum resident protein 29 OS=Ory | ERP29 | RABBIT | #N/A | 1.7385 | #N/A | #N/A | #N/A | #N/A | #N/A | -0.28145 | #N/A | 0 | -1.87923 | -7.70973 | -2.36155 | 0.08212 | 0.07865 | 0.17426 | 0.18248 | 1 | 2.93229 | 0.55262 | 0.03961 | 0.422156 | Not Significant | NS | #N/A |
| 2484 | A0A5F9CHZ1 | #N/A | PDE1B | RABBIT | #N/A | #N/A | #N/A | 0.87964 | #N/A | #N/A | #N/A | -1.00164 | #N/A | #N/A | -1.88128 | #N/A | -1.78948 | #N/A | 0.1736 | #N/A | 0.26575 | 0 | 2.93229 | 0.55262 | #N/A | 0.552617 | Not Significant | NS | #N/A |
| 1624 | G1SN27 | WASH complex subunit 4 OS=Oryctolagus cuniculu | WASHC4 | RABBIT | #N/A | #N/A | #N/A | 0.91465 | #N/A | #N/A | #N/A | #N/A | -0.97229 | #N/A | -1.88693 | #N/A | -1.79485 | #N/A | 0.17267 | #N/A | 0.26481 | 0 | 2.93229 | 0.55262 | #N/A | 0.552617 | Not Significant | NS | #N/A |
| 305 | U3KNZ4 | Nucleoside diphosphate kinase OS=Oryctolagus cu | #N/A | RABBIT | 0.6426 | 1.62342 | -1.99518 | 0.33161 | 2.70964 | #N/A | 0 | -1.10892 | -2.82998 | -0.96787 | -1.88911 | -1.8374 | -2.08819 | 0.10876 | 0.06351 | 0.19697 | 0.16581 | 7 | 2.93229 | 0.55262 | 2.34907 | 1.818704 | Not Significant | NS | #N/A |
| 634 | G1TDQ2 | VHL binding protein 1 OS=Oryctolagus cuniculus O) | #N/A | RABBIT | 1.37938 | 2.30983 | -0.00704 | 1.14304 | 0.61177 | -2.43127 | -0.76284 | -0.66673 | 0.17769 | -1.74689 | -2.17341 | -3.63998 | -3.84098 | 0.00659 | 0.00277 | 0.07586 | 0.05341 | 8 | 2.93229 | 0.55262 | 0.8913 | 0.800456 | Significant | Down mtub | #N/A |
| 1897 | A0A5F9DH82 | #N/A | INPP4B | RABBIT | #N/A | 2.10279 | #N/A | #N/A | #N/A | #N/A | #N/A | -0.19857 | -0.11535 | 0.11535 | -2.16898 | -11.55 | -3.22493 | 0.00741 | 0.0238 | 0.0819 | 0.11365 | 2 | 2.93229 | 0.55262 | 0.02645 | 0.33926 | Significant | Down mtub | INPP4B |
| 2120 | G1SDT0 | #N/A | ACTBL2 | RABBIT | #N/A | #N/A | -0.05994 | 1.96604 | #N/A | 2.56253 | -6.36188 | #N/A | 0.1239 | -0.1239 | -1.90289 | -0.65139 | -0.84266 | 0.55033 | 0.42753 | 0.43252 | 0.41011 | 4 | 2.93229 | 0.55262 | 11.3786 | 6.799302 | Not Significant | NS | #N/A |
| 379 | G1T0S1 | Opioid binding protein/cell adhesion molecule like ( | OPCML | RABBIT | 1.86013 | 1.98414 | 0.30968 | 1.80922 | 0.76885 | -2.59924 | -0.35379 | -0.55528 | -0.13903 | -0.3567 | -2.14721 | -3.78901 | -3.95807 | 0.00532 | 0.00227 | 0.07293 | 0.05019 | 8 | 2.93229 | 0.55262 | 0.80286 | 0.735736 | Significant | Down mtub | OPCML |
| 2164 | G1TCU5 | #N/A | SLC30A3 | RABBIT | #N/A | #N/A | 0.9759 | #N/A | 1.18963 | 0 | -1.75054 | 0.51538 | -1.26696 | -2.76207 | -2.13561 | -2.15043 | -2.44221 | 0.0842 | 0.04067 | 0.17453 | 0.13359 | 5 | 2.93229 | 0.55262 | 1.40895 | 1.092393 | Significant | Down mtub | SLC30A3 |
| 351 | G1SZH9 | Discs large MAGUK scaffold protein 2 OS=Oryctolag | DLG2 | RABBIT | 1.59545 | 3.5956 | #N/A | 1.29344 | -2.11293 | -2.59236 | 0.95509 | -0.26921 | -2.17786 | 0 | -1.90976 | -1.47874 | -1.70888 | 0.18274 | 0.11848 | 0.25742 | 0.21702 | 7 | 2.93229 | 0.55262 | 3.70647 | 2.775366 | Not Significant | NS | #N/A |
| 340 | G1U2B5 | Pyridoxal kinase OS=Oryctolagus cuniculus OX=998 | PDXK | RABBIT | 1.34134 | 2.49791 | -0.46992 | 1.19393 | 0.80592 | -3.72001 | 0.33052 | -0.7222 | -0.33052 | -0.84328 | -2.13093 | -2.52263 | -2.79454 | 0.03566 | 0.01754 | 0.13507 | 0.10298 | 8 | 2.93229 | 0.55262 | 1.78391 | 1.453649 | Significant | Down mtub | PDXK |
| 1937 | A0A5F9CX52 | #N/A | LARP1B | RABBIT | #N/A | 0.19782 | #N/A | 0.35528 | #N/A | #N/A | -1.65129 | #N/A | #N/A | #N/A | -1.92784 | -14.1378 | -2.44274 | 0.04495 | 0.07209 | 0.14686 | 0.17493 | 1 | 2.93229 | 0.55262 | 0.0124 | 0.415236 | Not Significant | NS | #N/A |
| 695 | G1TD43 | Discs large MAGUK scaffold protein 3 OS=Oryctolag | DLG3 | RABBIT | 3.40148 | 1.13542 | -0.08285 | 1.21358 | 0.55286 | -1.94485 | -0.90492 | -0.62655 | 0 | #N/A | -2.11318 | -2.79713 | -3.06398 | 0.02663 | 0.01205 | 0.12384 | 0.09479 | 7 | 2.93229 | 0.55262 | 1.26833 | 1.057034 | Significant | Down mtub | DLG3 |
| 908 | G1SEK2 | Protein phosphatase, Mg2+/Mn2+ dependent 1F OS | PPM1F | RABBIT | 0.21947 | 2.81028 | #N/A | -0.26545 | 0.86227 | #N/A | #N/A | #N/A | -1.41696 | -0.98966 | -2.10995 | -2.06592 | -2.39338 | 0.10773 | 0.04827 | 0.1968 | 0.14779 | 4 | 2.93229 | 0.55262 | 1.39077 | 1.03624 | Significant | Down mtub | PPM1F |
| 1458 | A0A5F9CMY6 | Pirin OS=Oryctolagus cuniculus OX=9986 GN=PIR F | PIR | RABBIT | 0.97282 | #N/A | #N/A | #N/A | #N/A | #N/A | #N/A | #N/A | -0.96115 | #N/A | -1.93397 | #N/A | -1.8396 | #N/A | 0.16522 | #N/A | 0.26016 | 0 | 2.93229 | 0.55262 | #N/A | 0.552617 | Not Significant | NS | #N/A |
| 114 | A0A5F9CV98 | UTP--glucose-1-phosphate uridylyltransferase OS=( | #N/A | RABBIT | 1.15839 | 1.13298 | #N/A | -6.1866 | 0.95559 | -8.11366 | -5.13414 | -0.06437 | -0.1306 | 0.06719 | -1.9402 | -0.78065 | -0.92215 | 0.46059 | 0.37831 | 0.39639 | 0.38956 | 7 | 2.93229 | 0.55262 | 13.7268 | 9.837427 | Not Significant | NS | #N/A |
| 653 | G1TE61 | Importin subunit alpha OS=Oryctolagus cuniculus ( | KPNA6 | RABBIT | 0.04268 | 0.45267 | 2.74871 | 2.28768 | -0.0444 | -2.32341 | 1.00068 | -0.13834 | -2.01251 | -1.50047 | -2.09228 | -2.43655 | -2.70364 | 0.04078 | 0.02062 | 0.14369 | 0.10939 | 8 | 2.93229 | 0.55262 | 1.84344 | 1.497211 | Significant | Down mtub | KPNA6 |
| 2392 | A0A5F9C124 | #N/A | FNDC1 | RABBIT | #N/A | #N/A | 1.92418 | #N/A | #N/A | -0.42092 | #N/A | 0.02015 | #N/A | -0.08452 | -2.08595 | -7.8387 | -3.0532 | 0.01589 | 0.02883 | 0.10512 | 0.12009 | 2 | 2.93229 | 0.55262 | 0.05311 | 0.350071 | Significant | Down mtub | FNDC1 |
| 1335 | A0A5F9DPI8 | non-specific serine/threonine protein kinase OS=Or | CDC42BP/ | RABBIT | 0.01166 | #N/A | #N/A | #N/A | 0.70723 | #N/A | #N/A | -1.58717 | #N/A | #N/A | -1.94662 | -3.23153 | -2.30955 | 0.19105 | 0.08321 | 0.26255 | 0.18576 | 1 | 2.93229 | 0.55262 | 0.24191 | 0.473602 | Not Significant | NS | #N/A |
| 1820 | G1U110 | #N/A | LINS1 | RABBIT | #N/A | 0.89709 | #N/A | -0.05764 | #N/A | #N/A | #N/A | #N/A | #N/A | -1.53366 | -1.95338 | -2.36252 | -2.19498 | 0.25491 | 0.09434 | 0.30166 | 0.19616 | 1 | 2.93229 | 0.55262 | 0.45576 | 0.527985 | Not Significant | NS | #N/A |
| 17 | G1SX46 | Contactin-1 OS=Oryctolagus cuniculus OX=9986 G | CNTN1 | RABBIT | 0.757 | 0.03503 | 0.64696 | 0.66582 | 1.10901 | -5.64746 | 0.03762 | -0.10198 | -0.51606 | -0.34534 | -1.95741 | -1.77763 | -2.01189 | 0.11336 | 0.06953 | 0.20019 | 0.17249 | 8 | 2.93229 | 0.55262 | 3.03126 | 2.366433 | Not Significant | NS | #N/A |
| 11 | G1SR03 | Transitional endoplasmic reticulum ATPase OS=Ory | VCP | RABBIT | 0.47516 | 0.05153 | 0.32362 | 0.03124 | 0.86953 | -7.0754 | 0.39565 | -0.15324 | -0.72506 | -0.47855 | -1.95754 | -1.40988 | -1.61455 | 0.19625 | 0.13487 | 0.26644 | 0.23421 | 8 | 2.93229 | 0.55262 | 4.81945 | 3.67499 | Not Significant | NS | #N/A |
| 2546 | G1SUX5 | #N/A | PLCL2 | RABBIT | #N/A | #N/A | #N/A | 1.96938 | #N/A | 0.74066 | 0 | #N/A | -0.45556 | -0.67321 | -2.06641 | -2.95747 | -2.69406 | 0.05967 | 0.03626 | 0.15524 | 0.12876 | 3 | 2.93229 | 0.55262 | 0.39055 | 0.470661 | Significant | Down mtub | PLCL2 |
| 779 | G1TKH3 | Superoxide dismutase [Cu-Zn] OS=Oryctolagus cun | SOD1 | RABBIT | 0.19463 | 3.66674 | -0.82325 | 0.92501 | #N/A | #N/A | 0.11801 | -0.96215 | -2.9538 | -0.11801 | -1.96977 | -1.65813 | -1.93278 | 0.14836 | 0.08554 | 0.22981 | 0.18812 | 6 | 2.93229 | 0.55262 | 2.82242 | 2.077287 | Not Significant | NS | #N/A |
| 626 | G1SPY4 | Isoaspartyl peptidase/L-asparaginase OS=Oryctola | ASRGL1 | RABBIT | 0.89054 | 2.01084 | -0.20958 | 0.55163 | 0.64805 | -3.79173 | 0.13512 | -1.45262 | -0.13512 | -1.18533 | -2.06423 | -2.63902 | -2.899 | 0.02976 | 0.01456 | 0.12757 | 0.09934 | 8 | 2.93229 | 0.55262 | 1.52958 | 1.267539 | Significant | Down mtub | ASRGL1 |
| 152 | G1SLS3 | AP-1 complex subunit gamma OS=Oryctolagus cun | AP1G1 | RABBIT | 1.22967 | 1.13666 | 0.00552 | 0.6829 | 0.53719 | -4.44216 | 0.05628 | -0.12065 | -1.33369 | -0.84312 | -2.05506 | -2.43161 | -2.69384 | 0.0411 | 0.02099 | 0.14411 | 0.10939 | 8 | 2.93229 | 0.55262 | 1.78567 | 1.454935 | Significant | Down mtub | AP1G1 |
| 2089 | G1SIY7 | #N/A | SV2B | RABBIT | #N/A | #N/A | 1.18616 | 0.11785 | 0.87363 | -0.17549 | -0.63214 | 1.45588 | -3.13 | -3.78258 | -1.97874 | -1.50645 | -1.76583 | 0.18267 | 0.11149 | 0.25742 | 0.21075 | 6 | 2.93229 | 0.55262 | 3.23497 | 2.354411 | Not Significant | NS | #N/A |
| 1687 | A0A5F9CID2 | #N/A | OGDH | RABBIT | #N/A | -0.03012 | #N/A | 0.1703 | 1.5841 | -1.40529 | #N/A | #N/A | #N/A | #N/A | -1.98005 | -1.94897 | -2.13941 | 0.19063 | 0.08614 | 0.26255 | 0.18814 | 2 | 2.93229 | 0.55262 | 0.77412 | 0.642433 | Not Significant | NS | #N/A |
| 2564 | A0A5F9DB17 | #N/A | GMPR2 GN | RABBIT | #N/A | #N/A | #N/A | 1.75112 | #N/A | 0 | #N/A | #N/A | #N/A | -0.4627 | -1.98247 | -4.94739 | -2.44218 | 0.12697 | 0.07213 | 0.2129 | 0.17493 | 1 | 2.93229 | 0.55262 | 0.10705 | 0.439306 | Not Significant | NS | #N/A |
| 2328 | A0A5F9DTN2 | #N/A | #N/A | RABBIT | #N/A | #N/A | #N/A | 0.93255 | #N/A | #N/A | #N/A | -1.05456 | #N/A | #N/A | -1.98711 | #N/A | -1.89015 | #N/A | 0.15725 | #N/A | 0.25435 | 0 | 2.93229 | 0.55262 | #N/A | 0.552617 | Not Significant | NS | #N/A |
| 682 | A0A5F9CLZ9 | Phosphorylase b kinase regulatory subunit OS=Ory( | PHKB | RABBIT | 2.70331 | 1.36307 | 1.27455 | -0.64458 | -0.0843 | -2.20123 | -0.95922 | 0.08481 | 0.07091 | -2.62327 | -2.04801 | -2.51303 | -2.7734 | 0.0362 | 0.01821 | 0.13507 | 0.10478 | 8 | 2.93229 | 0.55262 | 1.66039 | 1.363258 | Significant | Down mtub | PHKB |
| 675 | G1SFW1 | Triokinase/FMN cyclase OS=Oryctolagus cuniculus | TKFC | RABBIT | 3.22535 | 2.6117 | 0.17065 | 1.51457 | #N/A | #N/A | -0.04454 | -0.15187 | -0.27846 | #N/A | -2.03885 | -2.56204 | -2.83188 | 0.05052 | 0.02228 | 0.15026 | 0.11168 | 5 | 2.93229 | 0.55262 | 1.08564 | 0.888598 | Significant | Down mtub | TKFC |
| 2833 | G1T9A1 | #N/A | ITCH | RABBIT | #N/A | #N/A | #N/A | #N/A | 1.79625 | -0.43599 | #N/A | #N/A | 0 | #N/A | -2.01425 | -5.3346 | -2.48999 | 0.11797 | 0.06856 | 0.20536 | 0.17188 | 1 | 2.93229 | 0.55262 | 0.09505 | 0.436254 | Not Significant | NS | #N/A |
| 2253 | G1TCP6 | #N/A | ITIH5 | RABBIT | #N/A | #N/A | #N/A | 0.46391 | 2.15864 | #N/A | #N/A | -0.58591 | #N/A | -0.82435 | -2.0164 | -2.3564 | -2.54965 | 0.14257 | 0.05194 | 0.22477 | 0.15166 | 2 | 2.93229 | 0.55262 | 0.73224 | 0.625454 | Not Significant | NS | #N/A |
| 1382 | A0A5F9CME8 | Adenylate cyclase type 10-like OS=Oryctolagus cur | #N/A | RABBIT | 1.16812 | #N/A | 2.3607 | -0.0066 | -0.06442 | 1.01699 | #N/A | -1.87254 | -1.57059 | -2.22029 | -2.02606 | -2.16674 | -2.46052 | 0.07339 | 0.03632 | 0.16663 | 0.12876 | 6 | 2.93229 | 0.55262 | 1.74872 | 1.356061 | Significant | Down mtub | #N/A |
| 228 | G1SJX5 | Ankyrin 3 OS=Oryctolagus cuniculus OX=9986 GN= | ANK3 | RABBIT | 0.63391 | -1.45611 | 0.58669 | 1.6435 | 1.60807 | -2.77545 | -1.07553 | #N/A | -0.41604 | #N/A | -2.02556 | -2.22808 | -2.50871 | 0.06744 | 0.03357 | 0.1625 | 0.1262 | 6 | 2.93229 | 0.55262 | 1.54963 | 1.22233 | Significant | Down mtub | ANK3 |
| 2837 | A0A5F9C422 | #N/A | SMARCA5 | RABBIT | #N/A | #N/A | #N/A | #N/A | 0.98022 | #N/A | #N/A | -1.05796 | #N/A | #N/A | -2.03818 | #N/A | -1.93872 | #N/A | 0.15001 | #N/A | 0.24914 | 0 | 2.93229 | 0.55262 | #N/A | 0.552617 | Not Significant | NS | #N/A |
| 1121 | A0A5F9CCR1 | Tubby-like protein OS=Oryctolagus cuniculus OX=9 | TUB | RABBIT | 0.41676 | -0.20728 | #N/A | 0.34849 | 0.80621 | #N/A | -1.82839 | #N/A | -1.50965 | #N/A | -2.01007 | -6.1291 | -4.12567 | 0.00359 | 0.00452 | 0.06691 | 0.06461 | 4 | 2.93229 | 0.55262 | 0.14341 | 0.316498 | Significant | Down mtub | TUB |
| 280 | G1T013 | Malic enzyme OS=Oryctolagus cuniculus OX=9986 | ME1 | RABBIT | 1.51037 | 1.82801 | 0.17065 | 0.80007 | 0.72997 | #N/A | -0.11042 | -1.43137 | -1.65645 | -0.73492 | -1.99111 | -4.37362 | -4.25003 | 0.00326 | 0.00172 | 0.06421 | 0.04595 | 7 | 2.93229 | 0.55262 | 0.46057 | 0.487744 | Significant | Down mtub | ME1 |
| 1006 | A0A5F9D3Z0 | HMG box domain-containing protein OS=Oryctolagu | #N/A | RABBIT | 0.99792 | #N/A | #N/A | #N/A | #N/A | #N/A | #N/A | -1.05063 | #N/A | #N/A | -2.04855 | #N/A | -1.94859 | #N/A | 0.14859 | #N/A | 0.24819 | 0 | 2.93229 | 0.55262 | #N/A | 0.552617 | Not Significant | NS | #N/A |
| 279 | G1ST56 | Importin 9 OS=Oryctolagus cuniculus OX=9986 GN | IPO9 | RABBIT | 1.1655 | 3.06948 | -1.38268 | 1.94367 | 1.43137 | -2.09014 | 0.24415 | -0.30852 | -0.27147 | -1.22608 | -1.97588 | -2.34531 | -2.5974 | 0.04702 | 0.02492 | 0.14885 | 0.11547 | 8 | 2.93229 | 0.55262 | 1.77445 | 1.446724 | Significant | Down mtub | IPO9 |
| 2350 | A0A5F9CCI4 | #N/A | OSBPL6 | RABBIT | #N/A | #N/A | #N/A | #N/A | 1.99188 | #N/A | #N/A | #N/A | 0 | -0.13011 | -2.05693 | -18.2555 | -2.60946 | 0.03484 | 0.06048 | 0.13441 | 0.16242 | 1 | 2.93229 | 0.55262 | 0.00846 | 0.414236 | Not Significant | NS | #N/A |
| 477 | A0A5F9CU35 | Cell cycle associated protein 1 OS=Oryctolagus cur | CAPRIN1 | RABBIT | 2.1829 | 1.78636 | 0.17065 | 1.58094 | 0.14739 | #N/A | -0.44521 | -0.90244 | -1.09215 | -0.75961 | -1.9735 | -3.97077 | -3.96684 | 0.00539 | 0.00269 | 0.07293 | 0.05341 | 7 | 2.93229 | 0.55262 | 0.54892 | 0.550014 | Significant | Down mtub | CAPRIN1 |
| 1160 | A0A5F9CT29 | Cysteine and glycine rich protein 3 OS=Oryctolagus | CSRP3 | RABBIT | 0.39205 | 0.96492 | #N/A | -0.05764 | #N/A | #N/A | -1.19739 | -1.87372 | #N/A | #N/A | -1.96866 | -4.30151 | -3.40869 | 0.02312 | 0.0146 | 0.11776 | 0.09934 | 3 | 2.93229 | 0.55262 | 0.25135 | 0.400265 | Significant | Down mtub | CSRP3 |
| 2587 | G1SIJ8 | #N/A | IPO4 | RABBIT | #N/A | #N/A | #N/A | 1.56107 | #N/A | 0.9728 | #N/A | -1.52135 | -0.9728 | #N/A | -2.06818 | -1.36656 | -1.76903 | 0.30511 | 0.13792 | 0.32493 | 0.23706 | 2 | 2.93229 | 0.55262 | 1.71784 | 1.025107 | Not Significant | NS | #N/A |
| 53 | G1SD01 | Spectrin beta chain OS=Oryctolagus cuniculus OX= | SPTB | RABBIT | 0.58409 | -0.29978 | 0.81015 | -1.02827 | 1.29918 | -8.73208 | 0.68514 | 0.43323 | -0.7277 | -0.65935 | -2.07323 | -1.14889 | -1.32664 | 0.28379 | 0.21167 | 0.31659 | 0.29127 | 8 | 2.93229 | 0.55262 | 8.14095 | 6.105585 | Not Significant | NS | #N/A |
| 1844 | O97669 | #N/A | #N/A | RABBIT | #N/A | 1.13911 | #N/A | #N/A | #N/A | #N/A | #N/A | #N/A | -0.94129 | #N/A | -2.08041 | #N/A | -1.97889 | #N/A | 0.14432 | #N/A | 0.24331 | 0 | 2.93229 | 0.55262 | #N/A | 0.552617 | Not Significant | NS | #N/A |
| 752 | G1SDD6 | Angiotensinogen OS=Oryctolagus cuniculus OX=99 | AGT | RABBIT | 0.01166 | 1.28602 | #N/A | 2.1563 | 2.19765 | #N/A | -0.47362 | -0.41867 | -0.6987 | #N/A | -1.94324 | -3.18431 | -3.26648 | 0.02442 | 0.01155 | 0.11931 | 0.09305 | 5 | 2.93229 | 0.55262 | 0.63841 | 0.606698 | Significant | Down mtub | AGT |
| 1498 | A0A5F9CAE1 | Interactor of little elongation complex ELL subunit 1 | ICE1 | RABBIT | 0.89755 | 0.19782 | #N/A | #N/A | #N/A | #N/A | #N/A | -1.54216 | #N/A | #N/A | -2.08985 | -3.4487 | -2.47756 | 0.17967 | 0.06947 | 0.25448 | 0.17249 | 1 | 2.93229 | 0.55262 | 0.24481 | 0.47434 | Not Significant | NS | #N/A |
| 941 | A0A5F9C4Q6 | Spermine synthase OS=Oryctolagus cuniculus OX= | SMS | RABBIT | 1.05163 | #N/A | #N/A | #N/A | #N/A | #N/A | #N/A | #N/A | #N/A | -1.03996 | -2.09159 | #N/A | -1.98953 | #N/A | 0.14286 | #N/A | 0.24228 | 0 | 2.93229 | 0.55262 | #N/A | 0.552617 | Not Significant | NS | #N/A |
| 16 | P00883 | Fructose-bisphosphate aldolase A OS=Oryctolagus | ALDOA | RABBIT | 1.45987 | 1.7151 | 0.04382 | 0.7988 | 0.40612 | -3.23022 | 0.12683 | -0.68254 | -1.03309 | -0.46232 | -1.94101 | -2.96299 | -3.17678 | 0.01806 | 0.00888 | 0.11098 | 0.08502 | 8 | 2.93229 | 0.55262 | 1.07283 | 0.9333 | Significant | Down mtub | ALDOA |
| 1281 | G1U6C8 | Protein phosphatase 1 regulatory subunit 1B OS=Or | PPP1R1B | RABBIT | -0.37892 | 0.5884 | #N/A | 4.29026 | #N/A | #N/A | #N/A | -0.5942 | #N/A | #N/A | -2.09412 | -0.73589 | -1.0855 | 0.5384 | 0.32788 | 0.42845 | 0.36006 | 2 | 2.93229 | 0.55262 | 6.07346 | 2.79127 | Not Significant | NS | #N/A |
| 317 | A0A5F9CEA3 | Nucleosome assembly protein 1 like 4 OS=Oryctola | NAP1L4 | RABBIT | 1.69509 | 1.30048 | 0.20493 | 0.55002 | 0.8286 | -3.47754 | -0.68537 | -0.09864 | -0.60217 | -0.21973 | -1.93252 | -2.84666 | -3.06887 | 0.02159 | 0.01075 | 0.11776 | 0.09154 | 8 | 2.93229 | 0.55262 | 1.15217 | 0.991353 | Significant | Down mtub | NAP1L4 |
| 497 | A0A5F9DRI0 | Neurotrimin OS=Oryctolagus cuniculus OX=9986 G | NTM | RABBIT | 1.66555 | 0.85047 | 0.17065 | 0.67527 | 1.03984 | #N/A | -0.208 | -0.15841 | -1.94439 | -1.89465 | -1.93172 | -3.71501 | -3.75982 | 0.00751 | 0.00377 | 0.0819 | 0.05982 | 7 | 2.93229 | 0.55262 | 0.60084 | 0.5866 | Significant | Down mtub | NTM |
| 574 | G1T7L5 | Inositol monophosphatase 1 OS=Oryctolagus cunic | IMPA1 | RABBIT | 1.98332 | 3.66937 | -0.30638 | 1.92681 | -0.89339 | #N/A | 0 | -1.6775 | #N/A | #N/A | -2.1147 | -1.44636 | -1.73216 | 0.20772 | 0.1218 | 0.27264 | 0.22013 | 5 | 2.93229 | 0.55262 | 3.05385 | 2.129235 | Not Significant | NS | #N/A |
| 1681 | G1SZV5 | #N/A | APOA1 | RABBIT | #N/A | 0.1172 | 0.25127 | #N/A | 1.10461 | -1.86826 | #N/A | #N/A | -3.43971 | 0.4169 | -2.12138 | -1.82633 | -2.19465 | 0.14183 | 0.06461 | 0.22469 | 0.16683 | 4 | 2.93229 | 0.55262 | 2.02382 | 1.401519 | Not Significant | NS | #N/A |
| 762 | A0A5F9DA16 | Nuclear distribution protein nudE-like 1 OS=Oryctol | NDEL1 | RABBIT | 0.02463 | 0.19782 | #N/A | 0.11794 | 0.98016 | #N/A | -1.26701 | -1.5035 | -1.99096 | #N/A | -1.91729 | -6.08506 | -4.49739 | 0.00173 | 0.00205 | 0.05406 | 0.05019 | 5 | 2.93229 | 0.55262 | 0.17019 | 0.311559 | Significant | Down mtub | NDEL1 |
| 1553 | G1U4G4 | Coiled-coil domain containing 70 OS=Oryctolagus ( | CCDC70 | RABBIT | 0.1552 | 0.19782 | -0.12093 | 0.24361 | 0.20296 | -1.5234 | #N/A | #N/A | -2.0181 | #N/A | -1.90648 | -11.1553 | -4.74536 | 0.0001 | 0.00149 | 0.02953 | 0.04508 | 5 | 2.93229 | 0.55262 | 0.04173 | 0.230584 | Significant | Down mtub | CCDC70 |
| 225 | G1TVT0 | Nucleolin OS=Oryctolagus cuniculus OX=9986 GN= | NCL | RABBIT | -0.40203 | 1.00559 | 0.47571 | 0.18494 | 0.84821 | -6.83777 | -1.20513 | -0.04965 | -0.51163 | -0.01471 | -2.14626 | -1.62532 | -1.85733 | 0.14275 | 0.09039 | 0.22477 | 0.19156 | 8 | 2.93229 | 0.55262 | 4.3594 | 3.338334 | Not Significant | NS | #N/A |
| 588 | A0A5F9C403 | Neogenin 1 OS=Oryctolagus cuniculus OX=9986 GI | NEO1 | RABBIT | 1.02468 | 0.19782 | -0.76556 | 1.93263 | 0.36079 | #N/A | 0.50212 | -0.76946 | -2.99542 | -2.16089 | -1.90599 | -2.25028 | -2.50479 | 0.05917 | 0.03134 | 0.15506 | 0.12426 | 7 | 2.93229 | 0.55262 | 1.59424 | 1.286726 | Significant | Down mtub | NEO1 |
| 332 | U3KLW8 | Trafficking from ER to golgi regulator OS=Oryctolagu | TFG | RABBIT | 1.0217 | 0.61763 | 0.30708 | 0.51668 | 0.70237 | -5.91953 | -0.22137 | -0.20079 | -0.68904 | -0.57946 | -2.15513 | -1.94189 | -2.19888 | 0.08809 | 0.05033 | 0.17722 | 0.15072 | 8 | 2.93229 | 0.55262 | 3.0792 | 2.401516 | Not Significant | NS | #N/A |
| 1929 | G1U1X1 | #N/A | APIP | RABBIT | #N/A | #N/A | #N/A | 1.05006 | #N/A | -1.1077 | #N/A | #N/A | #N/A | #N/A | -2.15776 | #N/A | -2.05247 | #N/A | 0.13455 | #N/A | 0.23397 | 0 | 2.93229 | 0.55262 | #N/A | 0.552617 | Not Significant | NS | #N/A |
| 2069 | A0A5F9C6D0 | #N/A | DHX40 | RABBIT | #N/A | 2.1569 | #N/A | #N/A | #N/A | #N/A | #N/A | #N/A | -0.00387 | 0 | -2.15883 | -643.716 | -2.74587 | 0.00099 | 0.05257 | 0.04382 | 0.15178 | 1 | 2.93229 | 0.55262 | 7.5E-06 | 0.412086 | Not Significant | NS | #N/A |
| 1494 | P9WHE5 | Large ribosomal subunit protein uL11 OS=Mycobac | #N/A | MT | 1.08693 | #N/A | #N/A | #N/A | #N/A | #N/A | #N/A | #N/A | #N/A | -1.07526 | -2.16219 | #N/A | -2.05668 | #N/A | 0.13402 | #N/A | 0.23351 | 0 | 2.93229 | 0.55262 | #N/A | 0.552617 | Not Significant | NS | #N/A |
| 223 | G1SI20 | Isocitrate dehydrogenase [NADP] OS=Oryctolagus ( | IDH1 | RABBIT | 2.38789 | 2.40598 | -1.10674 | 1.71146 | 0.06881 | -1.85873 | 0.6937 | -1.85502 | -0.93065 | -0.0822 | -1.90006 | -2.21868 | -2.46122 | 0.0573 | 0.03173 | 0.15458 | 0.1243 | 8 | 2.93229 | 0.55262 | 1.83352 | 1.489951 | Significant | Down mtub | IDH1 |
| 1262 | G1T4X2 | Contactin 3 OS=Oryctolagus cuniculus OX=9986 G | CNTN3 | RABBIT | 0.01166 | #N/A | #N/A | 0.29721 | #N/A | #N/A | -2.01669 | #N/A | #N/A | #N/A | -2.17113 | -8.77956 | -2.72741 | 0.0722 | 0.05357 | 0.16495 | 0.15308 | 1 | 2.93229 | 0.55262 | 0.04077 | 0.422451 | Not Significant | NS | #N/A |
| 728 | A0A5F9D988 | Leucine-rich repeat transmembrane neuronal prote | LRRTM1 | RABBIT | 1.43442 | -0.18225 | 0.69852 | 0.88335 | 1.11766 | #N/A | 0 | -1.43331 | -1.43411 | -1.56767 | -1.89911 | -4.22958 | -4.09094 | 0.00389 | 0.00221 | 0.06755 | 0.05019 | 7 | 2.93229 | 0.55262 | 0.44802 | 0.478898 | Significant | Down mtub | LRRTM1 |
| 396 | P12345 | Aspartate aminotransferase, mitochondrial OS=Ory | GOT2 | RABBIT | 2.29561 | 4.13637 | -1.18064 | 2.00812 | -1.20549 | #N/A | -1.39597 | -2.70271 | #N/A | 1.19211 | -2.17965 | -1.33951 | -1.59166 | 0.22891 | 0.14618 | 0.28879 | 0.24572 | 6 | 2.93229 | 0.55262 | 4.96456 | 3.516212 | Not Significant | NS | #N/A |
| 1823 | G1SYH9 | #N/A | DNAH6 | RABBIT | #N/A | 0.94709 | #N/A | #N/A | 1.13282 | #N/A | #N/A | -1.63255 | 0 | -0.86818 | -1.87354 | -3.0576 | -2.89957 | 0.0551 | 0.02771 | 0.15427 | 0.11898 | 3 | 2.93229 | 0.55262 | 0.45055 | 0.501003 | Significant | Down mtub | DNAH6 |
| 447 | G1SUV6 | Neuronal growth regulator 1 OS=Oryctolagus cunic | NEGR1 | RABBIT | 1.86308 | 1.33054 | -0.48056 | 2.25549 | 0.21391 | #N/A | 0 | -1.16024 | -1.12427 | -1.04603 | -1.86912 | -2.96904 | -3.14714 | 0.02084 | 0.01047 | 0.11673 | 0.09154 | 7 | 2.93229 | 0.55262 | 0.88071 | 0.783846 | Significant | Down mtub | NEGR1 |
| 190 | A0A5F9CXV7 | Tropomyosin alpha-1 chain OS=Oryctolagus cunicu | #N/A | RABBIT | 1.06548 | #N/A | 0.61143 | #N/A | #N/A | -1.59642 | #N/A | #N/A | #N/A | -0.44078 | -1.85706 | -2.99131 | -2.66708 | 0.09594 | 0.04512 | 0.1866 | 0.143 | 2 | 2.93229 | 0.55262 | 0.38541 | 0.484817 | Significant | Down mtub | #N/A |
| 961 | G1T2S8 | Neural cell adhesion molecule 2 OS=Oryctolagus cu | NCAM2 | RABBIT | 0.27836 | 1.91957 | -0.22066 | 1.409 | 1.15682 | #N/A | -0.8007 | -0.06437 | -1.38301 | -1.54531 | -1.85696 | -3.50955 | -3.56892 | 0.00987 | 0.00516 | 0.09414 | 0.06702 | 7 | 2.93229 | 0.55262 | 0.62215 | 0.601619 | Significant | Down mtub | NCAM2 |
| 2553 | A0A5F9DPK3 | #N/A | ATP2A2 | RABBIT | #N/A | #N/A | #N/A | 1.07726 | #N/A | -1.1349 | #N/A | #N/A | #N/A | #N/A | -2.21215 | #N/A | -2.1042 | #N/A | 0.12815 | #N/A | 0.22682 | 0 | 2.93229 | 0.55262 | #N/A | 0.552617 | Not Significant | NS | #N/A |
| 1718 | A0A5F9DBK0 | #N/A | OXCT1 | RABBIT | #N/A | 1.96876 | #N/A | #N/A | #N/A | #N/A | #N/A | -0.06437 | -0.44924 | #N/A | -2.22556 | -6.67713 | -2.76819 | 0.09464 | 0.05139 | 0.18575 | 0.15166 | 1 | 2.93229 | 0.55262 | 0.07406 | 0.430919 | Not Significant | NS | #N/A |
| 2886 | A0A5F9C0S8 | #N/A | KLHL1 | RABBIT | #N/A | #N/A | #N/A | #N/A | 1.10773 | #N/A | #N/A | #N/A | -1.12111 | #N/A | -2.22884 | #N/A | -2.12008 | #N/A | 0.12626 | #N/A | 0.2257 | 0 | 2.93229 | 0.55262 | #N/A | 0.552617 | Not Significant | NS | #N/A |
| 1788 | A0A5F9CRG3 | #N/A | MAPK9 | RABBIT | #N/A | 0.23992 | #N/A | 2.20157 | -0.01338 | -1.71375 | #N/A | 0.30201 | -1.31024 | -1.40366 | -1.84078 | -2.31931 | -2.56201 | 0.06811 | 0.03377 | 0.163 | 0.1262 | 5 | 2.93229 | 0.55262 | 1.07986 | 0.884959 | Significant | Down mtub | MAPK9 |

| | | | | | | | | | | | | | | | | | | | | | | | | | | | | | |
|---|---|---|---|---|---|---|---|---|---|---|---|---|---|---|---|---|---|---|---|---|---|---|---|---|---|---|---|---|---|
| 703 | A0A5F9C5D0 | MHD domain-containing protein OS=Oryctolagus cu | #N/A | RABBIT | 0.76558 | 1.8314 | 0.0534 | 0.88509 | -0.03803 | -3.22246 | 0.02464 | 0.08204 | -1.52773 | -1.05589 | -1.83937 | -2.65141 | -2.86748 | 0.02919 | 0.0154 | 0.12708 | 0.09934 | 8 | 2.93229 | 0.55262 | 1.20316 | 1.028667 | Significant | Down mtub | #N/A |
| 294 | A0A5F9DI27 | Dipeptidyl peptidase like 10 OS=Oryctolagus cunicu | DPP10 | RABBIT | 1.97982 | 2.12069 | -0.27353 | 0.98275 | 0.90169 | #N/A | 0 | -0.58506 | -1.45405 | -0.66436 | -1.81815 | -3.26713 | -3.3665 | 0.01373 | 0.00723 | 0.09752 | 0.07947 | 7 | 2.93229 | 0.55262 | 0.6882 | 0.648171 | Significant | Down mtub | DPP10 |
| 2839 | G1TJ27 | #N/A | #N/A | RABBIT | #N/A | #N/A | #N/A | #N/A | 1.11914 | #N/A | -1.13253 | #N/A | #N/A | #N/A | -2.25167 | #N/A | -2.14179 | #N/A | 0.12373 | #N/A | 0.22213 | 0 | 2.93229 | 0.55262 | #N/A | 0.552617 | Not Significant | NS | #N/A |
| 1084 | G1SM91 | Fumarylacetoacetase OS=Oryctolagus cuniculus O | FAH | RABBIT | 0.9097 | 0.80618 | #N/A | -0.05764 | #N/A | -2.44084 | 0.18489 | #N/A | -1.90648 | -0.88663 | -1.81501 | -2.47659 | -2.68276 | 0.05607 | 0.02802 | 0.15432 | 0.11898 | 5 | 2.93229 | 0.55262 | 0.92073 | 0.784652 | Significant | Down mtub | FAH |
| 1587 | G1U6K9 | Gap junction protein OS=Oryctolagus cuniculus OX | #N/A | RABBIT | 1.13496 | #N/A | #N/A | #N/A | #N/A | #N/A | #N/A | #N/A | -1.1233 | #N/A | -2.25826 | #N/A | -2.14807 | #N/A | 0.12301 | #N/A | 0.22107 | 0 | 2.93229 | 0.55262 | #N/A | 0.552617 | Not Significant | NS | #N/A |
| 439 | G1SXI6 | Neurexin 2 OS=Oryctolagus cuniculus OX=9986 GN | NRXN2 | RABBIT | 1.05859 | 0.769 | 0.40964 | -0.05764 | 1.19051 | #N/A | -0.62761 | -1.28007 | -1.61101 | -0.93826 | -1.78826 | -5.62719 | -4.70283 | 0.00079 | 0.00085 | 0.04295 | 0.036 | 7 | 2.93229 | 0.55262 | 0.22442 | 0.321314 | Significant | Down mtub | NRXN2 |
| 1926 | G1SCK8 | #N/A | RBBP5 | RABBIT | #N/A | #N/A | #N/A | 0.7515 | #N/A | -3.03363 | 0 | #N/A | #N/A | #N/A | -2.26831 | -0.8634 | -1.47238 | 0.54659 | 0.21609 | 0.43084 | 0.29349 | 1 | 2.93229 | 0.55262 | 4.60146 | 1.582257 | Not Significant | NS | #N/A |
| 50 | G1SW00 | Glutamine synthetase OS=Oryctolagus cuniculus C | GLUL | RABBIT | 0.83648 | 0.81093 | 0.15002 | 0.65058 | 1.10111 | -3.21764 | -0.05498 | -0.04374 | -0.32513 | -1.73039 | -1.7842 | -2.78936 | -2.97908 | 0.02358 | 0.01262 | 0.11849 | 0.09521 | 8 | 2.93229 | 0.55262 | 1.02286 | 0.896734 | Significant | Down mtub | GLUL |
| 690 | G1U7C1 | EMAP like 2 OS=Oryctolagus cuniculus OX=9986 GI | EML2 | RABBIT | 1.19831 | 2.24408 | 0.38799 | -0.27498 | 0.55987 | -1.59731 | -0.4064 | #N/A | -0.85969 | #N/A | -1.77752 | -2.85955 | -2.97786 | 0.02882 | 0.01563 | 0.12702 | 0.09934 | 6 | 2.93229 | 0.55262 | 0.72449 | 0.668069 | Significant | Down mtub | EML2 |
| 542 | A0A5F9C1X1 | 10 kDa heat shock protein, mitochondrial OS=Oryct | #N/A | RABBIT | 1.72534 | 2.79348 | -1.01416 | 1.09226 | -0.01338 | -2.22727 | #N/A | -0.42286 | -0.99691 | 0.23816 | -1.76893 | -2.00689 | -2.2448 | 0.08475 | 0.04878 | 0.17453 | 0.14882 | 7 | 2.93229 | 0.55262 | 1.72647 | 1.379916 | Significant | Down mtub | #N/A |
| 2254 | G1TRS0 | #N/A | DKC1 | RABBIT | #N/A | #N/A | 1.79379 | #N/A | 1.47721 | -0.00523 | #N/A | -0.06437 | #N/A | -0.26146 | -1.74585 | -11.2884 | -3.56575 | 0.00149 | 0.01207 | 0.05271 | 0.09479 | 3 | 2.93229 | 0.55262 | 0.0287 | 0.28767 | Significant | Down mtub | DKC1 |
| 899 | A0A5F9C6K4 | Syntaxin binding protein 5 OS=Oryctolagus cuniculu | STXBP5 | RABBIT | 0.23339 | 1.01884 | 1.01458 | 1.07407 | -0.23512 | -1.36704 | -1.60422 | -0.94318 | 0.73177 | -2.39449 | -1.73659 | -2.98225 | -3.13205 | 0.01754 | 0.00961 | 0.1103 | 0.09004 | 8 | 2.93229 | 0.55262 | 0.8477 | 0.768554 | Significant | Down mtub | STXBP5 |
| 389 | A0A5F9CDY2 | Importin subunit alpha OS=Oryctolagus cuniculus ( | KPNA3 | RABBIT | 1.32198 | 1.38477 | -0.1195 | 0.58957 | -0.23111 | -2.2293 | 0.21773 | 0.24169 | -6.24886 | -0.57736 | -2.30836 | -1.82425 | -2.08054 | 0.10556 | 0.0618 | 0.19489 | 0.16343 | 8 | 2.93229 | 0.55262 | 4.00295 | 3.077494 | Not Significant | NS | #N/A |
| 502 | G1TBW5 | Contactin associated protein like 5 OS=Oryctolagus | CNTNAP5 | RABBIT | 1.57791 | 0.63144 | #N/A | 0.69919 | -1.303 | #N/A | -3.38451 | #N/A | #N/A | -0.43362 | -2.31045 | -1.80025 | -2.17761 | 0.1462 | 0.06625 | 0.22839 | 0.16941 | 4 | 2.93229 | 0.55262 | 2.19616 | 1.500956 | Not Significant | NS | #N/A |
| 388 | G1SIN7 | Geranylgeranyl transferase type-2 subunit alpha OS | RABGGTA | RABBIT | 1.19879 | 2.0714 | -1.0628 | 1.64051 | 1.50508 | -0.02508 | 0.02508 | -1.52661 | -0.52478 | -1.25625 | -1.73212 | -2.72509 | -2.90739 | 0.02604 | 0.01434 | 0.12244 | 0.09913 | 8 | 2.93229 | 0.55262 | 1.01003 | 0.887343 | Significant | Down mtub | RABGGTA |
| 1440 | G1T4V7 | Desmoplakin OS=Oryctolagus cuniculus OX=9986 | DSP | RABBIT | #N/A | #N/A | #N/A | #N/A | 1.94956 | #N/A | 0.64231 | #N/A | -1.12916 | -0.64231 | -2.32595 | -2.20102 | -2.46429 | 0.1587 | 0.0576 | 0.23799 | 0.15955 | 2 | 2.93229 | 0.55262 | 0.83756 | 0.668157 | Not Significant | NS | #N/A |
| 421 | Q28730 | Intercellular adhesion molecule 5 OS=Oryctolagus | ICAM5 TLC | RABBIT | 0.75014 | 0.13461 | -0.47395 | 2.23368 | 0.31661 | -3.05672 | 0.39981 | -0.3434 | 0.06321 | -2.7381 | -1.72725 | -2.00638 | -2.22687 | 0.07972 | 0.04793 | 0.17277 | 0.14727 | 8 | 2.93229 | 0.55262 | 1.85279 | 1.504053 | Significant | Down mtub | ICAM5 TLCN |
| 476 | A0A5F9CFR3 | Neuroligin 3 OS=Oryctolagus cuniculus OX=9986 G | NLGN3 | RABBIT | 1.49803 | 1.96236 | -0.3482 | 0.9654 | 0.00782 | #N/A | -1.30725 | #N/A | -1.37798 | -0.02121 | -1.71923 | -2.58487 | -2.73929 | 0.0415 | 0.02303 | 0.14427 | 0.11217 | 6 | 2.93229 | 0.55262 | 0.82945 | 0.738573 | Significant | Down mtub | NLGN3 |
| 160 | G1TJK2 | Neurofilament light polypeptide OS=Oryctolagus cu | NEFL | RABBIT | 0.44195 | 0.63453 | 0.99303 | -0.30587 | 1.23824 | -4.17974 | -0.22585 | 0.10687 | -0.17124 | -1.12175 | -1.71872 | -2.05606 | -2.27523 | 0.07381 | 0.04404 | 0.16683 | 0.14088 | 8 | 2.93229 | 0.55262 | 1.74693 | 1.426588 | Significant | Down mtub | NEFL |
| 69 | A0A5F9DPL1 | L-lactate dehydrogenase OS=Oryctolagus cuniculu | LDHB | RABBIT | 1.37317 | 1.87297 | -0.0804 | 0.69594 | 0.58524 | -2.88909 | 0.18146 | -0.37638 | -0.83596 | -0.18146 | -1.70967 | -2.67647 | -2.85787 | 0.02808 | 0.01566 | 0.12583 | 0.09934 | 8 | 2.93229 | 0.55262 | 1.02009 | 0.894705 | Significant | Down mtub | LDHB |
| 1102 | A0A5F9DNV3 | Serine/arginine repetitive matrix 2 OS=Oryctolagus | SRRM2 | RABBIT | 3.90852 | #N/A | 0.17065 | #N/A | #N/A | #N/A | #N/A | -0.31008 | #N/A | #N/A | -2.34967 | -0.72586 | -1.29681 | 0.60029 | 0.26556 | 0.45128 | 0.3278 | 1 | 2.93229 | 0.55262 | 6.98583 | 2.188616 | Not Significant | NS | #N/A |
| 2847 | A0A5F9C6Q6 | #N/A | MYT1L | RABBIT | #N/A | #N/A | #N/A | #N/A | 1.16815 | #N/A | -1.18153 | #N/A | #N/A | #N/A | -2.34968 | #N/A | -2.23502 | #N/A | 0.11353 | #N/A | 0.21273 | 0 | 2.93229 | 0.55262 | #N/A | 0.552617 | Not Significant | NS | #N/A |
| 1659 | G1SNP7 | #N/A | MAP1A | RABBIT | #N/A | 0.37988 | 0.17065 | 1.14713 | 0.84458 | -0.535 | -8.05505 | 0.42201 | -0.16244 | -0.27601 | -2.35686 | -1.2988 | -1.52319 | 0.23516 | 0.15889 | 0.29167 | 0.25503 | 7 | 2.93229 | 0.55262 | 7.31761 | 5.320396 | Not Significant | NS | #N/A |
| 2789 | G1SFP2 | #N/A | NHERF1 | RABBIT | #N/A | #N/A | #N/A | #N/A | 1.17982 | #N/A | #N/A | #N/A | -1.1932 | #N/A | -2.37301 | #N/A | -2.25722 | #N/A | 0.11125 | #N/A | 0.21054 | 0 | 2.93229 | 0.55262 | #N/A | 0.552617 | Not Significant | NS | #N/A |
| 413 | G1U7Q6 | Translin OS=Oryctolagus cuniculus OX=9986 GN=T | TSN | RABBIT | 1.0399 | 1.33415 | -0.6092 | 0.63398 | 0.32088 | -3.22674 | 0.25437 | -0.51375 | -2.02689 | -0.25437 | -1.69742 | -2.34055 | -2.54693 | 0.04738 | 0.02726 | 0.14885 | 0.11898 | 8 | 2.93229 | 0.55262 | 1.31487 | 1.110417 | Significant | Down mtub | TSN |
| 2556 | G1TMC6 | #N/A | ITPKA | RABBIT | #N/A | #N/A | #N/A | 2.84263 | #N/A | 2.41014 | -0.16827 | -0.37675 | #N/A | 0 | -2.37635 | -1.62867 | -1.99556 | 0.20187 | 0.09354 | 0.2713 | 0.19582 | 3 | 2.93229 | 0.55262 | 1.70312 | 1.134433 | Not Significant | NS | #N/A |
| 1136 | G1U0I5 | Ig-like domain-containing protein OS=Oryctolagus c | #N/A | RABBIT | #N/A | 2.4468 | #N/A | 2.73398 | -0.31235 | -1.02128 | -0.23817 | -0.10659 | 0.04223 | 0.97274 | -1.69303 | -2.04736 | -2.27035 | 0.08656 | 0.04955 | 0.17578 | 0.14997 | 6 | 2.93229 | 0.55262 | 1.28216 | 1.042665 | Significant | Down mtub | #N/A |
| 935 | G1U2E9 | D-dopachrome decarboxylase OS=Oryctolagus cun | #N/A | RABBIT | 1.67907 | 4.14637 | -0.86535 | 0.80444 | #N/A | #N/A | -1.01617 | #N/A | #N/A | -0.86208 | -2.38026 | -1.51806 | -1.88536 | 0.20361 | 0.10177 | 0.27183 | 0.20137 | 4 | 2.93229 | 0.55262 | 3.27801 | 2.125198 | Not Significant | NS | #N/A |
| 2876 | P25950 | #N/A | D3R s076F | RABBIT | #N/A | #N/A | #N/A | #N/A | 1.01205 | -4.14519 | #N/A | #N/A | -0.26865 | 0.26865 | -2.39377 | -0.86082 | -1.26627 | 0.48006 | 0.26193 | 0.40598 | 0.32448 | 2 | 2.93229 | 0.55262 | 5.7997 | 2.680263 | Not Significant | NS | #N/A |
| 1409 | A0A5F9D7R1 | BCL9 transcription coactivator OS=Oryctolagus cur | BCL9 | RABBIT | 1.17429 | #N/A | #N/A | #N/A | #N/A | #N/A | #N/A | -1.22699 | #N/A | #N/A | -2.40128 | #N/A | -2.2841 | #N/A | 0.10857 | #N/A | 0.20752 | 0 | 2.93229 | 0.55262 | #N/A | 0.552617 | Not Significant | NS | #N/A |
| 1484 | G1SJQ9 | HECT domain E3 ubiquitin protein ligase 3 OS=Oryc | HECTD3 | RABBIT | 2.10318 | #N/A | 0.06408 | -0.04997 | 2.05726 | #N/A | 0.21384 | -1.02484 | -0.00767 | -1.74661 | -1.68496 | -2.23717 | -2.45321 | 0.06661 | 0.03675 | 0.16176 | 0.12876 | 6 | 2.93229 | 0.55262 | 1.13452 | 0.943493 | Significant | Down mtub | HECTD3 |
| 230 | A0A5F9CMI1 | Neurexin-1 OS=Oryctolagus cuniculus OX=9986 GN | NRXN1 | RABBIT | 0.7149 | 0.49382 | 0.17065 | 0.92352 | 1.28867 | #N/A | -0.0799 | -0.61963 | -1.23644 | -1.92358 | -1.6832 | -4.10686 | -3.84334 | 0.00453 | 0.00329 | 0.06905 | 0.05702 | 7 | 2.93229 | 0.55262 | 0.37328 | 0.426228 | Significant | Down mtub | NRXN1 |
| 2449 | G1T902 | #N/A | #N/A | RABBIT | #N/A | #N/A | 1.84509 | #N/A | #N/A | #N/A | #N/A | -0.06437 | -1.09569 | #N/A | -2.42512 | -2.71524 | -2.67648 | 0.22465 | 0.05644 | 0.28563 | 0.15773 | 1 | 2.93229 | 0.55262 | 0.53181 | 0.547326 | Not Significant | NS | #N/A |
| 901 | A0A5F9CYH7 | protein-tyrosine-phosphatase OS=Oryctolagus cun | PTPRG | RABBIT | 0.66351 | 1.56212 | #N/A | 1.4707 | -0.42675 | #N/A | 0.41337 | -1.42799 | -1.01746 | -1.4156 | -1.67931 | -2.64682 | -2.79458 | 0.03819 | 0.02105 | 0.1391 | 0.10939 | 6 | 2.93229 | 0.55262 | 0.80509 | 0.722208 | Significant | Down mtub | PTPRG |
| 437 | G1T2A3 | glycerophosphocholine cholinephosphodiesterase | ENPP6 | RABBIT | 0.0062 | 0.20328 | 0.71101 | 1.00808 | 0.55474 | -2.43192 | -1.54123 | 0.08795 | -1.08414 | -0.9402 | -1.67857 | -3.74887 | -3.69834 | 0.00563 | 0.00355 | 0.07337 | 0.05832 | 8 | 2.93229 | 0.55262 | 0.50121 | 0.514997 | Significant | Down mtub | ENPP6 |
| 589 | G1TAM8 | Proteasome activator complex subunit 3 OS=Oryctc | PSME3 | RABBIT | 1.37188 | 1.54814 | -1.01409 | 1.67888 | 0.68737 | #N/A | -0.70628 | -0.799 | -1.69248 | 0 | -1.65387 | -2.57978 | -2.74476 | 0.03648 | 0.02078 | 0.13574 | 0.10939 | 7 | 2.93229 | 0.55262 | 0.91333 | 0.806835 | Significant | Down mtub | PSME3 |
| 41 | G1TPK8 | Neurofilament medium polypeptide OS=Oryctolagu | #N/A | RABBIT | 0.80442 | 0.814 | 0.82542 | -0.23412 | 0.99246 | -3.80026 | -0.27587 | 0.11211 | -0.60852 | -0.43637 | -1.64222 | -2.209 | -2.41158 | 0.05817 | 0.03465 | 0.15468 | 0.1262 | 8 | 2.93229 | 0.55262 | 1.38169 | 1.159313 | Significant | Down mtub | #N/A |
| 2796 | A0A5F9DRN4 | #N/A | PNKD | RABBIT | #N/A | #N/A | #N/A | #N/A | 1.1841 | #N/A | #N/A | -1.26185 | #N/A | #N/A | -2.44594 | #N/A | -2.32659 | #N/A | 0.10449 | #N/A | 0.20358 | 0 | 2.93229 | 0.55262 | #N/A | 0.552617 | Not Significant | NS | #N/A |
| 499 | G1TD98 | glutathione-disulfide reductase OS=Oryctolagus cu | GSR | RABBIT | 1.9251 | 2.86987 | -0.6627 | 1.81919 | -0.01338 | #N/A | -0.75076 | 0.26194 | -0.23697 | -1.05352 | -1.63244 | -2.07308 | -2.28492 | 0.07687 | 0.04557 | 0.17055 | 0.14364 | 7 | 2.93229 | 0.55262 | 1.37795 | 1.13429 | Significant | Down mtub | GSR |
| 666 | G1U982 | Immunoglobin superfamily member 21 OS=Oryctol | IGSF21 | RABBIT | 0.94417 | 1.048 | -1.13116 | 1.27426 | 1.20998 | #N/A | 0 | -1.44491 | -1.24768 | -1.1542 | -1.63075 | -2.76877 | -2.89229 | 0.02774 | 0.01615 | 0.12548 | 0.10049 | 7 | 2.93229 | 0.55262 | 0.77088 | 0.706442 | Significant | Down mtub | IGSF21 |
| 198 | Q6R8J5 | Chaperonin-containing TCP-1 subunit gamma (Frag | CCT3 | RABBIT | 1.1254 | 0.20289 | 0.68623 | 0.31609 | 0.75974 | -1.30577 | -0.01234 | -0.06944 | -1.69077 | -1.92492 | -1.61872 | -3.70715 | -3.63037 | 0.00598 | 0.00399 | 0.07337 | 0.06113 | 8 | 2.93229 | 0.55262 | 0.47665 | 0.497028 | Significant | Down mtub | CCT3 |
| 31 | B7NZM8 | Tyrosine 3-monooxygenase/tryptophan 5-monooxy | YWHAH | RABBIT | 0.88928 | 2.45902 | -0.19927 | 0.64341 | 0.23147 | -2.52386 | -0.24485 | -0.80792 | -0.79444 | 0.30627 | -1.61774 | -2.46568 | -2.64423 | 0.03897 | 0.02293 | 0.1391 | 0.11217 | 8 | 2.93229 | 0.55262 | 1.07618 | 0.93575 | Significant | Down mtub | YWHAH |
| 1206 | G1T616 | Phosphoglucomutase 2 OS=Oryctolagus cuniculus | PGM2 | RABBIT | #N/A | 1.09543 | #N/A | 0.00722 | #N/A | #N/A | -0.06486 | #N/A | #N/A | -3.82854 | -2.49802 | -1.27521 | -1.81967 | 0.33034 | 0.12927 | 0.33706 | 0.22731 | 2 | 2.93229 | 0.55262 | 3.83737 | 1.884554 | Not Significant | NS | #N/A |
| 409 | A0A5F9DQK8 | Dynactin subunit 3 OS=Oryctolagus cuniculus OX=9 | DCTN3 | RABBIT | 1.31211 | 0.13197 | 1.11433 | #N/A | 3.1033 | -1.14949 | 0.41035 | -0.06594 | 0 | -0.15939 | -1.60832 | -2.60467 | -2.74965 | 0.03519 | 0.02061 | 0.13498 | 0.10939 | 7 | 2.93229 | 0.55262 | 0.84728 | 0.760285 | Significant | Down mtub | DCTN3 |
| 934 | G1TN86 | CutA divalent cation tolerance homolog OS=Orycto | CUTA | RABBIT | 0.01166 | 2.00832 | #N/A | #N/A | #N/A | #N/A | -1.49195 | #N/A | #N/A | #N/A | -2.50194 | -1.44691 | -2.13095 | 0.38499 | 0.10129 | 0.36278 | 0.20137 | 1 | 2.93229 | 0.55262 | 1.99333 | 0.918996 | Not Significant | NS | #N/A |
| 967 | A0A5F9CHJ2 | SWI/SNF related, matrix associated, actin depender | SMARCC1 | RABBIT | 1.07499 | 0.73517 | 0.17065 | 1.15155 | 0.99284 | #N/A | -0.59468 | -0.52856 | -1.45594 | -0.53154 | -1.60272 | -5.65453 | -4.44452 | 0.00077 | 0.00127 | 0.04295 | 0.04432 | 7 | 2.93229 | 0.55262 | 0.17853 | 0.288971 | Significant | Down mtub | SMARCC1 |
| 1719 | A0A5F9DLN0 | #N/A | #N/A | RABBIT | #N/A | 1.32018 | #N/A | #N/A | #N/A | #N/A | #N/A | -0.06437 | #N/A | -2.31038 | -2.50756 | -1.28916 | -1.99473 | 0.42001 | 0.11803 | 0.37909 | 0.21667 | 1 | 2.93229 | 0.55262 | 2.5223 | 1.053517 | Not Significant | NS | #N/A |
| 1932 | A0A5F9CLJ8 | #N/A | ERO1A | RABBIT | #N/A | 1.89361 | #N/A | -0.05764 | #N/A | #N/A | #N/A | #N/A | -1.593 | #N/A | -2.51099 | -1.48594 | -2.16569 | 0.37711 | 0.09745 | 0.35899 | 0.19688 | 1 | 2.93229 | 0.55262 | 1.90368 | 0.896199 | Not Significant | NS | #N/A |
| 711 | A0A5F9CED7 | Glutamate--cysteine ligase OS=Oryctolagus cunicu | GCLC | RABBIT | 1.75925 | 3.36595 | -1.10269 | 1.90526 | -0.01338 | #N/A | -0.74341 | -1.95044 | #N/A | #N/A | -2.5298 | -1.87498 | -2.22694 | 0.11964 | 0.05684 | 0.20728 | 0.15828 | 5 | 2.93229 | 0.55262 | 2.60064 | 1.843558 | Not Significant | NS | #N/A |
| 2635 | Q28739 | #N/A | BPI | RABBIT | #N/A | #N/A | #N/A | 1.54217 | #N/A | -1.29037 | 1.1515 | -4.93621 | 1.28286 | -1.1515 | -2.53091 | -0.91612 | -1.16937 | 0.41144 | 0.28089 | 0.37603 | 0.3349 | 4 | 2.93229 | 0.55262 | 6.36019 | 3.903644 | Not Significant | NS | #N/A |
| 71 | Q9N1E2 | Glucose-6-phosphate isomerase OS=Oryctolagus c | GPI | RABBIT | 1.15739 | 2.17459 | -0.40781 | 1.16943 | 0.04329 | -2.82971 | 0.04113 | -0.51484 | -0.04113 | -0.51057 | -1.5984 | -2.28982 | -2.47863 | 0.05128 | 0.03077 | 0.15026 | 0.12309 | 8 | 2.93229 | 0.55262 | 1.21817 | 1.039653 | Significant | Down mtub | GPI |
| 2807 | A0A5F9CL79 | #N/A | MAPK12 | RABBIT | #N/A | #N/A | #N/A | #N/A | 1.26616 | -1.27955 | #N/A | #N/A | #N/A | #N/A | -2.54571 | #N/A | -2.42149 | #N/A | 0.09603 | #N/A | 0.19688 | 0 | 2.93229 | 0.55262 | #N/A | 0.552617 | Not Significant | NS | #N/A |
| 195 | G1STQ6 | Phosphoserine aminotransferase OS=Oryctolagus c | PSAT1 | RABBIT | 2.21226 | 2.82112 | -0.1384 | 1.29337 | 0.25983 | -0.70813 | 0.50295 | -0.33758 | -0.65477 | -0.34043 | -1.59723 | -2.65535 | -2.80579 | 0.02901 | 0.01719 | 0.12702 | 0.10198 | 8 | 2.93229 | 0.55262 | 0.90455 | 0.810153 | Significant | Down mtub | PSAT1 |
| 526 | G1TZV3 | Adaptor related protein complex 1 subunit mu 1 OS | #N/A | RABBIT | 1.21146 | 0.74213 | 0.131 | 1.00886 | 0.59397 | -3.40126 | 0.03965 | -0.18354 | -0.46922 | -0.27189 | -1.59474 | -2.38923 | -2.56913 | 0.04391 | 0.0262 | 0.14581 | 0.117 | 8 | 2.93229 | 0.55262 | 1.11378 | 0.963265 | Significant | Down mtub | #N/A |
| 177 | A0A5F9CAA0 | Neurexin 3 OS=Oryctolagus cuniculus OX=9986 GN | NRXN3 | RABBIT | 1.63818 | 0.24833 | 0.1377 | 1.02948 | #N/A | #N/A | 0 | -1.42355 | -1.0513 | #N/A | -1.58837 | -2.89349 | -2.8569 | 0.03405 | 0.02144 | 0.13313 | 0.11031 | 5 | 2.93229 | 0.55262 | 0.51659 | 0.529905 | Significant | Down mtub | NRXN3 |
| 306 | G1SJY8 | Hydroxymethylglutaryl-CoA synthase OS=Oryctolag | HMGCS1 | RABBIT | 1.13714 | 1.41402 | -0.12683 | 1.4526 | 0.78435 | -2.20557 | 0.18348 | -0.63163 | -0.42974 | -0.18348 | -1.58564 | -3.14953 | -3.20499 | 0.01361 | 0.00844 | 0.09752 | 0.08441 | 8 | 2.93229 | 0.55262 | 0.63366 | 0.611924 | Significant | Down mtub | HMGCS1 |
| 319 | G1SQM2 | Disintegrin and metalloproteinase domain-containi | ADAM2 | RABBIT | 0.8811 | #N/A | -0.01907 | 0.8645 | 0.17634 | #N/A | #N/A | #N/A | -0.88533 | -1.31716 | -1.57696 | -4.22452 | -3.11847 | 0.01343 | 0.01709 | 0.09752 | 0.10198 | 4 | 2.93229 | 0.55262 | 0.18579 | 0.340955 | Significant | Down mtub | ADAM2 |
| 1082 | G1T0N8 | Zinc finger SWIM-type containing 2 OS=Oryctolagus | ZSWIM2 | RABBIT | 1.18119 | 1.37761 | 0.03416 | 0.57802 | 0.1231 | #N/A | -0.60598 | -0.95772 | #N/A | -1.18648 | -1.57555 | -4.12119 | -3.56855 | 0.00621 | 0.00611 | 0.07337 | 0.07277 | 6 | 2.93229 | 0.55262 | 0.27404 | 0.365493 | Significant | Down mtub | ZSWIM2 |
| 286 | A0A5F9CFU6 | ubiquitinyl hydrolase 1 OS=Oryctolagus cuniculus ( | USP9X | RABBIT | -0.66057 | -1.27594 | 0.17065 | 0.02793 | 0.12071 | 0.0855 | -5.92571 | #N/A | #N/A | #N/A | -2.59666 | -1.56544 | -1.89516 | 0.17826 | 0.09499 | 0.25448 | 0.19659 | 5 | 2.93229 | 0.55262 | 3.93063 | 2.681895 | Not Significant | NS | #N/A |
| 200 | P25154 | Acylamino-acid-releasing enzyme OS=Oryctolagus | APEH | RABBIT | 0.97353 | 0.85085 | -0.7381 | 0.22676 | 0.10799 | #N/A | 0 | -1.56365 | -2.34173 | -1.2062 | -1.5621 | -2.83365 | -2.91286 | 0.02527 | 0.01559 | 0.12061 | 0.09934 | 7 | 2.93229 | 0.55262 | 0.67533 | 0.639099 | Significant | Down mtub | APEH |
| 2787 | G1T0C1 | #N/A | RPL15 | RABBIT | #N/A | #N/A | #N/A | #N/A | 3.06527 | #N/A | 2.42634 | -0.06437 | -0.43183 | -0.15346 | -2.6211 | -1.76179 | -2.16867 | 0.17632 | 0.07371 | 0.25448 | 0.17651 | 3 | 2.93229 | 0.55262 | 1.77071 | 1.168615 | Not Significant | NS | #N/A |
| 2404 | A0A5F9CLN3 | #N/A | TM9SF2 | RABBIT | #N/A | #N/A | 1.39823 | #N/A | #N/A | #N/A | -1.22758 | #N/A | #N/A | #N/A | -2.62581 | #N/A | -2.49768 | #N/A | 0.08983 | #N/A | 0.1911 | 0 | 2.93229 | 0.55262 | #N/A | 0.552617 | Not Significant | NS | #N/A |
| 2547 | G1T9H0 | #N/A | FAM98A | RABBIT | #N/A | #N/A | #N/A | 0.17258 | -0.02147 | -5.01446 | #N/A | -0.05628 | 0.33665 | -5.49901 | -2.63383 | -1.12294 | -1.4391 | 0.32431 | 0.1937 | 0.33479 | 0.27908 | 4 | 2.93229 | 0.55262 | 7.33504 | 4.466143 | Not Significant | NS | #N/A |
| 161 | G1T7F5 | Adducin 2 OS=Oryctolagus cuniculus OX=9986 GN | ADD2 | RABBIT | 1.03454 | 0.62061 | -0.05158 | 0.21434 | 0.82156 | -3.90393 | 0.20677 | -0.27114 | -0.35325 | -0.8264 | -1.55748 | -2.04068 | -2.23514 | 0.07559 | 0.04724 | 0.16901 | 0.14603 | 8 | 2.93229 | 0.55262 | 1.45625 | 1.213877 | Significant | Down mtub | ADD2 |
| 1194 | A0A5F9D9A1 | Cytokine induced apoptosis inhibitor 1 OS=Oryctola | CIAPIN1 | RABBIT | 0.07577 | 2.91264 | 0.77344 | 2.3405 | -0.01338 | -0.85063 | #N/A | -0.10456 | -0.17446 | -0.16747 | -1.54208 | -2.21748 | -2.39593 | 0.06211 | 0.03773 | 0.15739 | 0.12938 | 7 | 2.93229 | 0.55262 | 1.07468 | 0.920556 | Significant | Down mtub | CIAPIN1 |
| 181 | G1T671 | acetyl-CoA C-acetyltransferase OS=Oryctolagus cu | ACAT2 | RABBIT | 1.41848 | 2.08422 | 0.21666 | 1.14935 | 0.72405 | -0.93753 | -0.04601 | -0.37344 | -0.16633 | -0.56579 | -1.53637 | -4.35606 | -3.96267 | 0.00243 | 0.00225 | 0.05406 | 0.05019 | 8 | 2.93229 | 0.55262 | 0.31099 | 0.375799 | Significant | Down mtub | ACAT2 |
| 113 | G1SMM9 | Neuromodulin OS=Oryctolagus cuniculus OX=9986 | #N/A | RABBIT | 1.1336 | 1.95502 | #N/A | #N/A | #N/A | -3.84143 | 0.18611 | -0.71166 | #N/A | -0.18611 | -2.68258 | -1.91305 | -2.34387 | 0.12829 | 0.0519 | 0.21348 | 0.15166 | 4 | 2.93229 | 0.55262 | 2.62176 | 1.746535 | Not Significant | NS | #N/A |
| 1672 | A0A5F9D6P7 | #N/A | CHL1 | RABBIT | #N/A | 0.86915 | 0.41642 | -0.11083 | 0.84167 | #N/A | -0.398 | -0.01117 | -2.08521 | -1.50412 | -1.50372 | -2.82291 | -2.83515 | 0.03024 | 0.0197 | 0.12779 | 0.10842 | 6 | 2.93229 | 0.55262 | 0.56751 | 0.56262 | Significant | Down mtub | CHL1 |
| 1042 | A0A5F9CTH8 | Farnesyl pyrophosphate synthase OS=Oryctolagus | FDPS | RABBIT | 1.23582 | 2.67385 | -0.25805 | 0.92531 | 0.41141 | #N/A | -0.00246 | -1.39526 | -0.61631 | 0 | -1.50117 | -2.39633 | -2.53746 | 0.04772 | 0.02963 | 0.14885 | 0.12155 | 7 | 2.93229 | 0.55262 | 0.87208 | 0.777767 | Significant | Down mtub | FDPS |
| 2375 | A5U7L2 | #N/A | #N/A | MT | #N/A | #N/A | 1.43577 | #N/A | #N/A | #N/A | -1.26512 | #N/A | #N/A | #N/A | -2.70089 | #N/A | -2.5691 | #N/A | 0.08447 | #N/A | 0.18737 | 0 | 2.93229 | 0.55262 | #N/A | 0.552617 | Not Significant | NS | #N/A |
| 308 | P00949 | Phosphoglucomutase-1 OS=Oryctolagus cuniculus | PGM1 | RABBIT | 0.17621 | 1.76191 | #N/A | 0.06602 | #N/A | #N/A | 0 | -1.28491 | -1.43661 | -0.61056 | -1.50107 | -2.49086 | -2.54425 | 0.0551 | 0.03471 | 0.15427 | 0.1262 | 5 | 2.93229 | 0.55262 | 0.62257 | 0.596708 | Significant | Down mtub | PGM1 |
| 2499 | G1T489 | #N/A | #N/A | RABBIT | #N/A | #N/A | #N/A | 3.43356 | #N/A | -0.57388 | #N/A | 3.25979 | 0 | -0.08385 | -2.78305 | -1.41608 | -1.8372 | 0.25172 | 0.11639 | 0.30012 | 0.21451 | 3 | 2.93229 | 0.55262 | 3.08997 | 1.835775 | Not Significant | NS | #N/A |
| 2419 | A0A5F9CV69 | #N/A | #N/A | RABBIT | #N/A | #N/A | 1.68726 | #N/A | #N/A | -2.19433 | #N/A | #N/A | #N/A | 0 | -2.78442 | -1.46522 | -2.2463 | 0.38126 | 0.08916 | 0.36106 | 0.1902 | 1 | 2.93229 | 0.55262 | 2.40754 | 1.024333 | Not Significant | NS | #N/A |
| 2082 | G1U467 | #N/A | SV2C | RABBIT | #N/A | 1.46304 | #N/A | #N/A | #N/A | #N/A | #N/A | -1.32959 | #N/A | #N/A | -2.79263 | #N/A | -2.65636 | #N/A | 0.07845 | #N/A | 0.18226 | 0 | 2.93229 | 0.55262 | #N/A | 0.552617 | Not Significant | NS | #N/A |
| 1812 | G1SRN0 | #N/A | DNAH7 | RABBIT | #N/A | 1.49913 | #N/A | #N/A | #N/A | #N/A | -1.30131 | #N/A | #N/A | #N/A | -2.80043 | #N/A | -2.66378 | #N/A | 0.07796 | #N/A | 0.18205 | 0 | 2.93229 | 0.55262 | #N/A | 0.552617 | Not Significant | NS | #N/A |
| 773 | G1SI22 | A-kinase anchoring protein 12 OS=Oryctolagus cun | AKAP12 | RABBIT | 0.03806 | 2.32983 | #N/A | 0.70182 | 0.02068 | #N/A | -1.47428 | -0.09077 | -0.65029 | -0.65181 | -1.48938 | -2.42966 | -2.54263 | 0.05119 | 0.03175 | 0.15026 | 0.1243 | 6 | 2.93229 | 0.55262 | 0.75154 | 0.686239 | Significant | Down mtub | AKAP12 |
| 921 | Q9N2C2 | Prostaglandin-H2 D-isomerase OS=Oryctolagus cui | #N/A | RABBIT | 0.01166 | 2.09553 | -0.1152 | 0.36841 | 0.15453 | #N/A | 0.00605 | -1.94907 | -1.59392 | -0.37001 | -1.47972 | -2.39064 | -2.52501 | 0.04812 | 0.03027 | 0.14885 | 0.1227 | 7 | 2.93229 | 0.55262 | 0.85137 | 0.763173 | Significant | Down mtub | #N/A |
| 1509 | G1SN31 | Carbonic anhydrase OS=Oryctolagus cuniculus OX | #N/A | RABBIT | #N/A | 1.50443 | #N/A | #N/A | #N/A | #N/A | #N/A | #N/A | #N/A | -1.30661 | -2.81104 | #N/A | -2.67387 | #N/A | 0.07731 | #N/A | 0.18205 | 0 | 2.93229 | 0.55262 | #N/A | 0.552617 | Not Significant | NS | #N/A |
| 2280 | G1T4C5 | #N/A | SIK2 | RABBIT | #N/A | #N/A | 1.50592 | #N/A | #N/A | #N/A | -1.33527 | #N/A | #N/A | #N/A | -2.84119 | #N/A | -2.70255 | #N/A | 0.07548 | #N/A | 0.17973 | 0 | 2.93229 | 0.55262 | #N/A | 0.552617 | Not Significant | NS | #N/A |
| 253 | G1T221 | Chromosome 1 open reading frame 43 OS=Oryctola | C1orf43 | RABBIT | 0.34789 | 0.18301 | 0.17065 | 1.6758 | 1.08025 | #N/A | 0.35779 | -0.53603 | -1.32552 | -1.63992 | -1.47744 | -2.85735 | -2.88728 | 0.02443 | 0.01629 | 0.11931 | 0.10083 | 7 | 2.93229 | 0.55262 | 0.59412 | 0.581871 | Significant | Down mtub | C1orf43 |
| 1117 | G1SFV1 | Protein disulfide-isomerase OS=Oryctolagus cunicu | PDIA4 | RABBIT | 0.01166 | 2.72758 | #N/A | #N/A | #N/A | #N/A | -1.49694 | #N/A | #N/A | #N/A | -2.86656 | -1.21875 | -2.01442 | 0.43744 | 0.11543 | 0.3855 | 0.21416 | 1 | 2.93229 | 0.55262 | 3.68811 | 1.349988 | Not Significant | NS | #N/A |
| 2154 | A0A5F9D0C4 | #N/A | #N/A | RABBIT | #N/A | #N/A | 1.34495 | #N/A | #N/A | -3.04998 | #N/A | #N/A | #N/A | 0 | -2.86994 | -1.08654 | -1.8555 | 0.47361 | 0.13834 | 0.40262 | 0.2372 | 1 | 2.93229 | 0.55262 | 4.65118 | 1.594901 | Not Significant | NS | #N/A |
| 89 | A0A5F9D8X8 | Catenin alpha-2 OS=Oryctolagus cuniculus OX=998 | CTNNA2 | RABBIT | 1.34698 | 0.54576 | 0.06247 | -0.01668 | 0.86915 | -2.61812 | 0.03139 | -0.09576 | -0.04563 | -1.84412 | -1.47598 | -2.42951 | -2.57178 | 0.04123 | 0.02608 | 0.14424 | 0.117 | 8 | 2.93229 | 0.55262 | 0.92271 | 0.823444 | Significant | Down mtub | CTNNA2 |
| 1414 | A0A5F9CN46 | Phosphate regulating endopeptidase homolog X-lin | PHEX | RABBIT | 1.46311 | #N/A | #N/A | #N/A | #N/A | #N/A | -1.45145 | #N/A | #N/A | #N/A | -2.91455 | #N/A | -2.77233 | #N/A | 0.07125 | #N/A | 0.17448 | 0 | 2.93229 | 0.55262 | #N/A | 0.552617 | Not Significant | NS | #N/A |
| 1233 | G1TN79 | Myosin VIIA OS=Oryctolagus cuniculus OX=9986 GI | MYO7A | RABBIT | 0.5514 | #N/A | #N/A | -0.05764 | 0.63294 | #N/A | #N/A | -1.24928 | #N/A | -0.87935 | -1.43988 | -4.5966 | -2.73457 | 0.01935 | 0.03437 | 0.11273 | 0.1262 | 3 | 2.93229 | 0.55262 | 0.11775 | 0.332701 | Significant | Down mtub | MYO7A |
| 2361 | A0A5F9DSB9 | #N/A | CNTLN | RABBIT | #N/A | #N/A | 0.8278 | 1.14492 | #N/A | #N/A | #N/A | -0.06437 | -0.85915 | -0.35988 | -1.41416 | -4.39306 | -2.67235 | 0.02186 | 0.03732 | 0.11776 | 0.12876 | 3 | 2.93229 | 0.55262 | 0.12435 | 0.336039 | Significant | Down mtub | CNTLN |
| 1215 | G1SCE2 | inositol-1,4-bisphosphate 1-phosphatase OS=Oryc | INPP1 | RABBIT | 0.62497 | 2.72793 | #N/A | 0.93689 | -0.31262 | -1.36315 | 0 | -0.56802 | -0.50689 | 0.41575 | -1.39875 | -2.13997 | -2.28562 | 0.06964 | 0.04552 | 0.16349 | 0.14364 | 7 | 2.93229 | 0.55262 | 0.94941 | 0.832267 | Significant | Down mtub | INPP1 |
| 1975 | G1TB37 | #N/A | #N/A | RABBIT | #N/A | #N/A | #N/A | 1.40379 | #N/A | #N/A | #N/A | -1.5258 | #N/A | #N/A | -2.92959 | #N/A | -2.78663 | #N/A | 0.07042 | #N/A | 0.17322 | 0 | 2.93229 | 0.55262 | #N/A | 0.552617 | Not Significant | NS | #N/A |
| 24 | A0A5F9DCV7 | Dihydropyrimidinase like 3 OS=Oryctolagus cunicul | DPYSL3 | RABBIT | 0.66253 | 0.77995 | 0.21756 | 0.37249 | 0.5139 | -2.12012 | -0.04691 | -0.58733 | -0.59943 | -1.0897 | -1.39798 | -3.84747 | -3.54059 | 0.0049 | 0.00467 | 0.07293 | 0.06565 | 8 | 2.93229 | 0.55262 | 0.33006 | 0.389755 | Significant | Down mtub | DPYSL3 |
| 2869 | A0A5F9DP72 | #N/A | TANC2 | RABBIT | #N/A | #N/A | #N/A | 1.43233 | #N/A | #N/A | #N/A | -1.51008 | #N/A | #N/A | -2.94242 | #N/A | -2.79884 | #N/A | 0.06972 | #N/A | 0.17249 | 0 | 2.93229 | 0.55262 | #N/A | 0.552617 | Not Significant | NS | #N/A |
| 884 | G1SZJ5 | Prefoldin subunit 6 OS=Oryctolagus cuniculus OX=9 | PFDN6 | RABBIT | 1.28158 | 1.05973 | -0.15707 | 0.23048 | 0.1944 | -2.80634 | 0.39626 | -0.8331 | -0.20778 | -0.8538 | -1.38278 | -2.28689 | -2.41874 | 0.05151 | 0.03421 | 0.15041 | 0.1262 | 8 | 2.93229 | 0.55262 | 0.91402 | 0.817082 | Significant | Down mtub | PFDN6 |

| | | | | | | | | | | | | | | | | | | | | | | | | | | | | | |
|---|---|---|---|---|---|---|---|---|---|---|---|---|---|---|---|---|---|---|---|---|---|---|---|---|---|---|---|---|---|
| 2318 | G1U891 | #N/A | #N/A | RABBIT | #N/A | #N/A | 1.56222 | #N/A | #N/A | -1.39157 | #N/A | #N/A | #N/A | #N/A | -2.95379 | #N/A | -2.80966 | #N/A | 0.06911 | #N/A | 0.17249 | 0 | 2.93229 | 0.55262 | #N/A | 0.552617 | Not Significant | NS | #N/A |
| 156 | A0A5F9CLU9 | Tropomyosin alpha-1 chain OS=Oryctolagus cunicu | #N/A | RABBIT | 0.85468 | 0.15064 | 0.67577 | 0.16234 | 0.94376 | -3.1542 | -0.13154 | -0.01718 | -0.41644 | -0.36777 | -1.37486 | -2.24402 | -2.37902 | 0.05508 | 0.03669 | 0.15427 | 0.12876 | 8 | 2.93229 | 0.55262 | 0.93844 | 0.834955 | Significant | Down mtub | #N/A |
| 2104 | G1SN28 | #N/A | #N/A | RABBIT | #N/A | 1.58369 | #N/A | #N/A | #N/A | #N/A | #N/A | #N/A | -1.38587 | #N/A | -2.96956 | #N/A | -2.82465 | #N/A | 0.06827 | #N/A | 0.17163 | 0 | 2.93229 | 0.55262 | #N/A | 0.552617 | Not Significant | NS | #N/A |
| 2881 | A0A5F9C753 | #N/A | SQSTM1 | RABBIT | #N/A | #N/A | #N/A | #N/A | 1.48766 | #N/A | #N/A | #N/A | #N/A | -1.50104 | -2.9887 | #N/A | -2.84286 | #N/A | 0.06727 | #N/A | 0.17112 | 0 | 2.93229 | 0.55262 | #N/A | 0.552617 | Not Significant | NS | #N/A |
| 985 | A0A5F9CDR9 | PH domain and leucine rich repeat protein phospha | PHLPP1 | RABBIT | 1.50988 | #N/A | #N/A | #N/A | #N/A | -1.49822 | #N/A | #N/A | #N/A | #N/A | -3.00811 | #N/A | -2.86132 | #N/A | 0.06628 | #N/A | 0.16941 | 0 | 2.93229 | 0.55262 | #N/A | 0.552617 | Not Significant | NS | #N/A |
| 1983 | G1SEX6 | #N/A | DHX36 | RABBIT | #N/A | 1.60432 | #N/A | #N/A | #N/A | #N/A | #N/A | #N/A | -1.4065 | #N/A | -3.01082 | #N/A | -2.8639 | #N/A | 0.06614 | #N/A | 0.16941 | 0 | 2.93229 | 0.55262 | #N/A | 0.552617 | Not Significant | NS | #N/A |
| 520 | A0A5F9CKY0 | Amyloid-beta A4 protein OS=Oryctolagus cuniculus | APP | RABBIT | 0.87174 | 0.42176 | #N/A | 1.29246 | -0.26274 | #N/A | -0.17288 | 0.10851 | -1.63435 | -1.46576 | -1.37192 | -2.47549 | -2.51731 | 0.0481 | 0.0331 | 0.14885 | 0.12595 | 6 | 2.93229 | 0.55262 | 0.61428 | 0.59404 | Significant | Down mtub | APP |
| 73 | G1U2R2 | Aminopeptidase OS=Oryctolagus cuniculus OX=99 | NPEPPS | RABBIT | 1.11196 | 1.76379 | 0.11093 | 1.09198 | 0.46271 | -1.85468 | -0.15855 | -0.18547 | 0.02115 | -0.02115 | -1.34801 | -2.95046 | -2.9274 | 0.01841 | 0.01384 | 0.11171 | 0.09774 | 8 | 2.93229 | 0.55262 | 0.52186 | 0.530107 | Significant | Down mtub | NPEPPS |
| 937 | A0A5F9D9C3 | Growth factor receptor bound protein 2 OS=Oryctol | GRB2 | RABBIT | 1.53756 | #N/A | #N/A | #N/A | #N/A | -1.5259 | #N/A | #N/A | #N/A | #N/A | -3.06346 | #N/A | -2.91397 | #N/A | 0.06354 | #N/A | 0.16581 | 0 | 2.93229 | 0.55262 | #N/A | 0.552617 | Not Significant | NS | #N/A |
| 2616 | G1U577 | #N/A | ARMC6 | RABBIT | #N/A | #N/A | #N/A | 1.51118 | #N/A | #N/A | #N/A | #N/A | -1.56882 | #N/A | -3.08 | #N/A | -2.92971 | #N/A | 0.06275 | #N/A | 0.1645 | 0 | 2.93229 | 0.55262 | #N/A | 0.552617 | Not Significant | NS | #N/A |
| 2056 | A5U8N1 | #N/A | #N/A | MT | #N/A | 1.60903 | #N/A | #N/A | #N/A | #N/A | #N/A | -1.47558 | #N/A | #N/A | -3.08461 | #N/A | -2.93409 | #N/A | 0.06253 | #N/A | 0.16443 | 0 | 2.93229 | 0.55262 | #N/A | 0.552617 | Not Significant | NS | #N/A |
| 1663 | A0A5F9C974 | #N/A | NCAM1 | RABBIT | #N/A | 0.67702 | 0.05985 | 0.75798 | 1.30739 | #N/A | 0 | -0.23721 | #N/A | -1.69178 | -1.34356 | -2.50759 | -2.4525 | 0.05399 | 0.04003 | 0.15365 | 0.13248 | 5 | 2.93229 | 0.55262 | 0.49213 | 0.514491 | Significant | Down mtub | NCAM1 |
| 269 | G1U3G0 | Ubiquitin conjugating enzyme E2 O OS=Oryctolagus | UBE2O | RABBIT | 0.27817 | -0.02348 | 0.17065 | -0.00382 | 0.40077 | -1.47302 | -0.86607 | #N/A | #N/A | #N/A | -1.334 | -6.33581 | -3.22651 | 0.00144 | 0.01226 | 0.05271 | 0.09479 | 5 | 2.93229 | 0.55262 | 0.06333 | 0.244202 | Significant | Down mtub | UBE2O |
| 1105 | G1T364 | Aminopeptidase like 1 OS=Oryctolagus cuniculus C | NPEPL1 | RABBIT | 1.45361 | 1.47788 | #N/A | #N/A | #N/A | #N/A | -4.51847 | -0.5631 | #N/A | 0 | -3.1596 | -1.72153 | -2.27369 | 0.18363 | 0.06384 | 0.25793 | 0.16636 | 3 | 2.93229 | 0.55262 | 4.04218 | 2.317312 | Not Significant | NS | #N/A |
| 869 | G1TPT3 | IMD domain-containing protein OS=Oryctolagus cu | #N/A | RABBIT | 1.47135 | 0.94412 | 0.05566 | 0.0555 | 1.2972 | #N/A | -0.4538 | -0.17751 | -1.07484 | #N/A | -1.33348 | -2.98626 | -2.77633 | 0.02444 | 0.02168 | 0.11931 | 0.11035 | 6 | 2.93229 | 0.55262 | 0.37387 | 0.432548 | Significant | Down mtub | #N/A |
| 34 | G1T883 | Dihydropyrimidinase like 5 OS=Oryctolagus cunicul | DPYSL5 | RABBIT | 1.52097 | 1.6002 | -0.11918 | 0.7797 | 0.64287 | -1.3714 | -0.31442 | -0.02348 | -0.04089 | -0.47651 | -1.33025 | -3.31977 | -3.16379 | 0.01054 | 0.00908 | 0.09648 | 0.08604 | 8 | 2.93229 | 0.55262 | 0.40141 | 0.441969 | Significant | Down mtub | DPYSL5 |
| 369 | G1SN56 | Casein kinase II subunit alpha OS=Oryctolagus cun | CSNK2A2 | RABBIT | 0.93036 | 0.0043 | 1.36122 | -0.03083 | 1.71816 | -1.90834 | -0.02681 | 0.40251 | -0.61043 | -0.48119 | -1.32149 | -2.51322 | -2.58365 | 0.03619 | 0.02554 | 0.13507 | 0.11552 | 8 | 2.93229 | 0.55262 | 0.69121 | 0.654033 | Significant | Down mtub | CSNK2A2 |
| 35 | A0A5F9CN85 | 14-3-3 protein beta/alpha OS=Oryctolagus cuniculu | YWHAB | RABBIT | 1.1448 | 1.65546 | -0.61389 | 0.72748 | 0.29503 | -2.35979 | 0.15852 | -0.76484 | -0.20904 | -0.15852 | -1.30851 | -2.21025 | -2.32859 | 0.05806 | 0.0401 | 0.15468 | 0.13248 | 8 | 2.93229 | 0.55262 | 0.87621 | 0.789418 | Significant | Down mtub | YWHAB |
| 632 | G1SJS1 | Histone H2B OS=Oryctolagus cuniculus OX=9986 G | H2BC21 | RABBIT | -1.06671 | 5.57071 | 5.79104 | -0.12508 | 5.40419 | #N/A | 1.25331 | -0.06437 | -1.3572 | -0.44292 | -3.26762 | -1.82375 | -2.13799 | 0.11096 | 0.05841 | 0.1981 | 0.1605 | 7 | 2.93229 | 0.55262 | 7.13381 | 5.190857 | Not Significant | NS | #N/A |
| 370 | G1SPF5 | phosphoglycerate mutase (2,3-diphosphoglycerate | #N/A | RABBIT | 1.46139 | 2.54672 | 0.09172 | 1.80022 | -0.25671 | -0.25274 | 0.07893 | -0.58609 | -0.26383 | 0.15962 | -1.30149 | -2.39089 | -2.47676 | 0.04379 | 0.03087 | 0.14581 | 0.12309 | 8 | 2.93229 | 0.55262 | 0.74081 | 0.690329 | Significant | Down mtub | #N/A |
| 1616 | P9WJU9 | Probable transport protein MmpL6 OS=Mycobacter | #N/A | MT | 1.66704 | #N/A | #N/A | #N/A | #N/A | -1.65538 | #N/A | #N/A | #N/A | #N/A | -3.32242 | #N/A | -3.16029 | #N/A | 0.05247 | #N/A | 0.15178 | 0 | 2.93229 | 0.55262 | #N/A | 0.552617 | Not Significant | NS | #N/A |
| 323 | A0A5F9CLG9 | ELKS/RAB6-interacting/CAST family member 2 OS=( | ERC2 | RABBIT | 2.03284 | #N/A | #N/A | #N/A | #N/A | #N/A | #N/A | -0.06437 | #N/A | -2.59144 | -3.36075 | -1.53563 | -2.48018 | 0.36747 | 0.06928 | 0.35413 | 0.17249 | 1 | 2.93229 | 0.55262 | 3.19305 | 1.224091 | Not Significant | NS | #N/A |
| 107 | G1T386 | Guanine deaminase OS=Oryctolagus cuniculus OX= | GDA | RABBIT | 1.31879 | 1.18182 | -0.05785 | 1.03591 | 0.68412 | -0.84409 | -0.38473 | -0.50733 | 0.2285 | -0.83141 | -1.30037 | -4.13104 | -3.58188 | 0.00329 | 0.00435 | 0.06421 | 0.06392 | 8 | 2.93229 | 0.55262 | 0.24772 | 0.329498 | Significant | Down mtub | GDA |
| 1315 | A0A5F9DNC3 | Arginyl-tRNA--protein transferase 1 OS=Oryctolagus | ATE1 | RABBIT | 0.58675 | #N/A | #N/A | #N/A | #N/A | #N/A | #N/A | #N/A | -5.58462 | 0 | -3.37906 | -0.69867 | -1.31864 | 0.61177 | 0.25884 | 0.45453 | 0.32394 | 1 | 2.93229 | 0.55262 | 15.594 | 4.377715 | Not Significant | NS | #N/A |
| 811 | G1TVR9 | Lys-63-specific deubiquitinase OS=Oryctolagus cur | BRCC3 | RABBIT | 1.02247 | -0.30424 | 0.60758 | 0.31644 | 1.30968 | -1.53912 | 0.37659 | -0.43845 | -0.44564 | -1.4547 | -1.29065 | -2.83484 | -2.80992 | 0.02199 | 0.01706 | 0.11776 | 0.10198 | 8 | 2.93229 | 0.55262 | 0.5182 | 0.527434 | Significant | Down mtub | BRCC3 |
| 1067 | G1TD10 | Kazrin, periplakin interacting protein OS=Oryctolagu | #N/A | RABBIT | 0.83941 | 0.74049 | 0.10689 | -0.05764 | 0.87532 | #N/A | -0.89533 | -0.59446 | #N/A | #N/A | -1.24579 | -3.66644 | -2.68193 | 0.0145 | 0.02805 | 0.09905 | 0.11898 | 5 | 2.93229 | 0.55262 | 0.16493 | 0.308244 | Significant | Down mtub | #N/A |
| 1977 | G1T0Q6 | #N/A | SMC3 | RABBIT | #N/A | 0.19782 | 0.11785 | 6.42408 | #N/A | 0.91517 | #N/A | #N/A | #N/A | -3.35104 | -3.46452 | -1.10669 | -1.52169 | 0.34919 | 0.17947 | 0.34656 | 0.26969 | 3 | 2.93229 | 0.55262 | 11.7602 | 6.220383 | Not Significant | NS | #N/A |
| 185 | M1ZMP8 | Aldehyde oxidase 3 OS=Oryctolagus cuniculus OX= | #N/A | RABBIT | 1.32378 | #N/A | 0.19519 | 1.14513 | #N/A | #N/A | #N/A | -0.08891 | -0.28144 | -0.69451 | -1.24299 | -3.16147 | -2.51107 | 0.03414 | 0.04065 | 0.13313 | 0.13359 | 4 | 2.93229 | 0.55262 | 0.23187 | 0.367543 | Significant | Down mtub | #N/A |
| 1666 | G1U9T2 | #N/A | PPP3CA | RABBIT | #N/A | 7.15745 | -0.37861 | 7.71135 | -0.34516 | #N/A | #N/A | #N/A | 0 | #N/A | -3.53626 | -0.7018 | -0.97401 | 0.53335 | 0.36808 | 0.42779 | 0.38387 | 3 | 2.93229 | 0.55262 | 20.312 | 10.54509 | Not Significant | NS | #N/A |
| 1835 | A0A5F9DSK5 | #N/A | MECP2 | RABBIT | #N/A | 0.19782 | #N/A | 2.16998 | #N/A | -4.06598 | -4.34816 | 1.20669 | #N/A | #N/A | -3.58638 | -1.46673 | -1.98902 | 0.23872 | 0.09439 | 0.29367 | 0.19616 | 3 | 2.93229 | 0.55262 | 7.17456 | 3.901382 | Not Significant | NS | #N/A |
| 203 | G1TQJ4 | AMP deaminase OS=Oryctolagus cuniculus OX=998 | AMPD2 | RABBIT | 0.74196 | 0.40411 | 0.17065 | 0.54724 | 0.94521 | #N/A | -0.22641 | -0.58491 | -0.65881 | -1.2523 | -1.24244 | -5.16447 | -3.67647 | 0.0013 | 0.00432 | 0.04885 | 0.06392 | 7 | 2.93229 | 0.55262 | 0.12861 | 0.253791 | Significant | Down mtub | AMPD2 |
| 101 | G1TQI2 | Dihydropyrimidinase like 4 OS=Oryctolagus cunicul | DPYSL4 | RABBIT | 1.2427 | 1.24258 | 0.05692 | 0.69119 | 0.43109 | -1.5979 | 0.11373 | -0.30943 | -0.2461 | -0.44271 | -1.22938 | -3.31209 | -3.0724 | 0.01066 | 0.01069 | 0.09686 | 0.09154 | 8 | 2.93229 | 0.55262 | 0.34444 | 0.400274 | Significant | Down mtub | DPYSL4 |
| 80 | G1SV05 | Heat shock protein family A (Hsp70) member 4 OS= | HSPA4 | RABBIT | 0.755 | 0.8164 | -0.10833 | 0.22135 | 0.91594 | -2.21469 | 0.30811 | -0.3527 | -0.54406 | -0.60034 | -1.20081 | -2.6057 | -2.59154 | 0.03134 | 0.02518 | 0.12952 | 0.11552 | 8 | 2.93229 | 0.55262 | 0.53093 | 0.536748 | Significant | Down mtub | HSPA4 |
| 318 | G1SZ76 | Small RNA binding exonuclease protection factor La | #N/A | RABBIT | 0.11216 | 1.0606 | 0.18503 | -0.05764 | 0.60163 | #N/A | -0.82601 | -0.11345 | -1.8527 | -0.48915 | -1.20069 | -3.00177 | -2.78271 | 0.01989 | 0.01948 | 0.11451 | 0.10808 | 7 | 2.93229 | 0.55262 | 0.35554 | 0.413725 | Significant | Down mtub | #N/A |
| 165 | G1TQ31 | Casein kinase II subunit alpha OS=Oryctolagus cun | #N/A | RABBIT | 0.41811 | 0.01544 | 0.50485 | -0.03392 | 1.58388 | -2.30242 | -0.02372 | -0.01071 | -0.35764 | -0.81214 | -1.199 | -2.32228 | -2.37736 | 0.04875 | 0.0368 | 0.14906 | 0.12876 | 8 | 2.93229 | 0.55262 | 0.66642 | 0.635894 | Significant | Down mtub | #N/A |
| 48 | G1SN14 | Cullin-associated NEDD8-dissociated protein 1 OS= | CAND1 | RABBIT | 0.79074 | 0.51534 | 0.20482 | 0.38051 | 0.85606 | -2.13876 | -0.03417 | -0.21588 | -0.44753 | -0.32784 | -1.18233 | -2.94291 | -2.80709 | 0.01863 | 0.01715 | 0.11219 | 0.10198 | 8 | 2.93229 | 0.55262 | 0.40352 | 0.44351 | Significant | Down mtub | CAND1 |
| 438 | G1SWK8 | Proteasome subunit beta OS=Oryctolagus cuniculu | PSMB7 | RABBIT | 0.30765 | 0.25136 | -0.05543 | 0.28478 | 1.05958 | #N/A | 0 | -0.48109 | -2.16988 | -0.58159 | -1.17773 | -2.53881 | -2.48243 | 0.03873 | 0.03256 | 0.1391 | 0.12544 | 7 | 2.93229 | 0.55262 | 0.47821 | 0.500174 | Significant | Down mtub | PSMB7 |
| 74 | A0A5F9CT82 | 1-phosphatidylinositol 4,5-bisphosphate phosphoc | PLCB1 | RABBIT | 0.9608 | 1.02705 | -0.32587 | 1.06326 | 0.57893 | #N/A | -0.2686 | -0.06437 | -0.94968 | -0.75207 | -1.16951 | -3.36848 | -2.93877 | 0.01195 | 0.01492 | 0.09752 | 0.09934 | 7 | 2.93229 | 0.55262 | 0.26787 | 0.351937 | Significant | Down mtub | PLCB1 |
| 698 | G1TT38 | Rabphilin 3A OS=Oryctolagus cuniculus OX=9986 G | RPH3A | RABBIT | 0.72409 | 0.31281 | 0.05566 | 0.76092 | 0.53455 | -0.14255 | -1.78146 | 0.7374 | -0.8042 | -1.40946 | -1.15766 | -2.46587 | -2.46491 | 0.03896 | 0.03153 | 0.1391 | 0.12426 | 8 | 2.93229 | 0.55262 | 0.55101 | 0.551441 | Significant | Down mtub | RPH3A |
| 311 | G1T0N5 | Purine rich element binding protein A OS=Oryctolag | PURA | RABBIT | 0.54549 | 0.19392 | 0.38365 | 0.80182 | 0.51199 | -1.8892 | 0.0039 | -0.29094 | -0.48883 | -0.63577 | -1.14754 | -3.3711 | -3.02314 | 0.00977 | 0.01167 | 0.09414 | 0.09305 | 8 | 2.93229 | 0.55262 | 0.28969 | 0.360212 | Significant | Down mtub | PURA |
| 149 | A0A5F9DBB4 | Interleukin enhancer binding factor 2 OS=Oryctolag | ILF2 | RABBIT | -0.12259 | 0.33721 | 0.3049 | 0.3186 | 0.26959 | -2.83619 | -0.58324 | 0.12008 | -1.10924 | -0.20439 | -1.14414 | -2.16895 | -2.23135 | 0.06192 | 0.04756 | 0.15739 | 0.14665 | 8 | 2.93229 | 0.55262 | 0.69566 | 0.657295 | Significant | Down mtub | ILF2 |
| 1083 | A0A5F9CEN7 | Pyridoxal phosphatase OS=Oryctolagus cuniculus ( | #N/A | RABBIT | 0.31113 | 1.46748 | -0.39636 | 1.12829 | -0.01338 | #N/A | -0.96994 | -0.81014 | 0.45796 | -1.2437 | -1.14089 | -2.21116 | -2.23299 | 0.06269 | 0.04976 | 0.15764 | 0.14997 | 7 | 2.93229 | 0.55262 | 0.59161 | 0.580095 | Significant | Down mtub | #N/A |
| 93 | G1T4P7 | Talin 2 OS=Oryctolagus cuniculus OX=9986 GN=TLI | TLN2 | RABBIT | 0.52284 | 0.18587 | 0.48611 | 0.09221 | 0.78009 | -2.08078 | -0.0928 | -0.21482 | 0.01195 | -1.20644 | -1.13 | -2.67114 | -2.59069 | 0.02831 | 0.02522 | 0.12583 | 0.11552 | 8 | 2.93229 | 0.55262 | 0.44741 | 0.475629 | Significant | Down mtub | TLN2 |
| 1368 | A0A5F9DLN2 | Y-box-binding protein 1 OS=Oryctolagus cuniculus ( | #N/A | RABBIT | 0.74512 | #N/A | 1.23238 | -0.05815 | 1.24804 | #N/A | -0.36541 | -0.06385 | -0.45522 | -0.45725 | -1.12728 | -3.52003 | -2.82176 | 0.01252 | 0.02014 | 0.09752 | 0.10938 | 6 | 2.93229 | 0.55262 | 0.20512 | 0.319194 | Significant | Down mtub | #N/A |
| 673 | G1SHF1 | Prefoldin subunit 2 OS=Oryctolagus cuniculus OX=9 | PFDN2 | RABBIT | 1.06231 | 1.54358 | 0.14233 | -0.05764 | 0.94729 | #N/A | 0.01628 | -0.60293 | -0.83847 | -0.11296 | -1.1121 | -2.90901 | -2.64772 | 0.02269 | 0.02454 | 0.11776 | 0.11483 | 7 | 2.93229 | 0.55262 | 0.32477 | 0.39204 | Significant | Down mtub | PFDN2 |
| 472 | A0A5F9CDW9 | SET nuclear proto-oncogene OS=Oryctolagus cunic | #N/A | RABBIT | 0.61053 | 1.14767 | 0.10715 | 0.72916 | 0.45086 | #N/A | 0 | -0.45082 | -1.44547 | -0.10743 | -1.11 | -3.1913 | -2.78653 | 0.01525 | 0.01935 | 0.10228 | 0.10772 | 7 | 2.93229 | 0.55262 | 0.26884 | 0.352622 | Significant | Down mtub | #N/A |
| 312 | A7X8X3 | Hypoxanthine phosphoribosyltransferase OS=Oryct | HPRT HPRT | RABBIT | 0.93626 | 1.19606 | -0.25883 | 0.56866 | 0.52754 | #N/A | 0 | -0.676 | -0.12625 | -1.14052 | -1.07963 | -2.97975 | -2.65035 | 0.02052 | 0.02443 | 0.11586 | 0.11483 | 7 | 2.93229 | 0.55262 | 0.29173 | 0.368749 | Significant | Down mtub | HPRT HPRT1 |
| 184 | G1T3E6 | Aspartyl aminopeptidase OS=Oryctolagus cuniculu | DNPEP | RABBIT | 0.27171 | 0.63137 | 0.17065 | 0.0618 | 0.66729 | #N/A | -0.18541 | -0.92902 | -1.15295 | -0.60495 | -1.07865 | -4.66745 | -3.23674 | 0.00229 | 0.009 | 0.05406 | 0.08569 | 7 | 2.93229 | 0.55262 | 0.11868 | 0.246792 | Significant | Down mtub | DNPEP |
| 392 | G1TDF1 | Syntaxin-1A OS=Oryctolagus cuniculus OX=9986 G | STX1A | RABBIT | 0.3799 | 1.18467 | 0.45572 | -0.0395 | 0.73489 | -1.43591 | -0.20017 | -0.0825 | -0.0458 | -0.84884 | -1.06578 | -3.15128 | -2.81845 | 0.01357 | 0.01681 | 0.09752 | 0.10198 | 8 | 2.93229 | 0.55262 | 0.28595 | 0.35748 | Significant | Down mtub | STX1A |
| 37 | G1SZD6 | 14-3-3 protein theta OS=Oryctolagus cuniculus OX= | YWHAQ | RABBIT | 0.23889 | 1.17714 | 0.2324 | 0.04907 | 0.43949 | -0.75579 | -0.06175 | -0.42408 | -0.36677 | -1.57562 | -1.0642 | -3.26608 | -2.87536 | 0.01142 | 0.01518 | 0.09732 | 0.09934 | 8 | 2.93229 | 0.55262 | 0.26542 | 0.342451 | Significant | Down mtub | YWHAQ |
| 32 | A0A5F9DCZ1 | Neuronal cell adhesion molecule OS=Oryctolagus c | NRCAM | RABBIT | 0.47647 | 0.19782 | 0.32839 | 0.8686 | 0.75153 | #N/A | -0.25915 | -0.5011 | -0.86331 | -0.4711 | -1.04823 | -5.8118 | -3.37712 | 0.00066 | 0.0071 | 0.0401 | 0.07947 | 7 | 2.93229 | 0.55262 | 0.07229 | 0.214096 | Significant | Down mtub | NRCAM |
| 10 | P11974 | Pyruvate kinase PKM OS=Oryctolagus cuniculus OX | PKM | RABBIT | 1.05778 | 1.43524 | -0.16785 | 0.54071 | 0.47383 | -1.07956 | 0.25619 | -0.32055 | -0.45629 | -0.28439 | -1.04486 | -3.01267 | -2.72232 | 0.01675 | 0.01995 | 0.10856 | 0.10892 | 8 | 2.93229 | 0.55262 | 0.30071 | 0.36828 | Significant | Down mtub | PKM |
| 42 | A0A5F9DI35 | Microtubule associated protein 1B OS=Oryctolagus | #N/A | RABBIT | 0.67946 | 0.55655 | 0.00528 | 0.65273 | 0.5955 | -1.74393 | -0.15931 | 0.09494 | -0.47661 | -0.38441 | -1.03177 | -3.0176 | -2.71106 | 0.01662 | 0.02035 | 0.10849 | 0.10939 | 8 | 2.93229 | 0.55262 | 0.29227 | 0.362099 | Significant | Down mtub | #N/A |
| 397 | G1SFC5 | Hook microtubule tethering protein 3 OS=Oryctolag | HOOK3 | RABBIT | 0.0011 | #N/A | 1.18577 | 0.60919 | 0.55158 | -0.59631 | #N/A | -0.73791 | #N/A | 0 | -1.03165 | -3.00498 | -2.34552 | 0.02993 | 0.04727 | 0.12757 | 0.14603 | 5 | 2.93229 | 0.55262 | 0.20205 | 0.331644 | Significant | Down mtub | HOOK3 |
| 45 | G1TEM1 | Actinin alpha 2 OS=Oryctolagus cuniculus OX=998 | ACTN2 | RABBIT | 0.76384 | 0.19782 | -0.05028 | 0.62588 | 0.84412 | #N/A | -0.28852 | -0.51525 | 0.0909 | -1.49805 | -1.029 | -2.88839 | -2.54979 | 0.02337 | 0.02902 | 0.11783 | 0.12009 | 7 | 2.93229 | 0.55262 | 0.28204 | 0.361922 | Significant | Down mtub | ACTN2 |
| 180 | G1T810 | Cullin 2 OS=Oryctolagus cuniculus OX=9986 GN=C | CUL2 | RABBIT | 0.89751 | 0.42683 | 0.19214 | 0.66163 | 0.40213 | -1.3969 | -0.44629 | -0.08585 | -0.09125 | -0.49931 | -1.01997 | -3.80334 | -3.04885 | 0.00521 | 0.01114 | 0.07293 | 0.09296 | 8 | 2.93229 | 0.55262 | 0.1798 | 0.279797 | Significant | Down mtub | CUL2 |
| 598 | G1SYZ0 | Syntaxin-1B OS=Oryctolagus cuniculus OX=9986 G | STX1B | RABBIT | 0.36398 | 1.44967 | 0.32649 | -0.21337 | 0.27675 | -1.1609 | -0.16302 | 0.09137 | -1.18371 | -0.4757 | -1.01909 | -2.70963 | -2.52572 | 0.02667 | 0.0283 | 0.12384 | 0.11898 | 8 | 2.93229 | 0.55262 | 0.35363 | 0.407003 | Significant | Down mtub | STX1B |
| 1541 | A0A5F9CAP9 | Guanylate cyclase 1 soluble subunit alpha 1 OS=Or | GUCY1A1 | RABBIT | -0.42613 | 0.34883 | #N/A | 0.82898 | 0.89334 | -1.04739 | 0.2159 | -0.87787 | 0 | -1.26041 | -1.00521 | -2.348 | -2.23332 | 0.05124 | 0.04974 | 0.15026 | 0.14997 | 7 | 2.93229 | 0.55262 | 0.40729 | 0.450195 | Significant | Down mtub | GUCY1A1 |
| 1485 | G1TMQ4 | Golgi associated PDZ and coiled-coil motif containi | GOPC | RABBIT | 0.7223 | 0.25789 | 0.31378 | 0.26531 | 0.47824 | -1.27947 | -0.50365 | -0.12443 | -0.30752 | -0.71165 | -0.99285 | -4.55657 | -3.23775 | 0.00186 | 0.00797 | 0.05406 | 0.08328 | 8 | 2.93229 | 0.55262 | 0.11869 | 0.235082 | Significant | Down mtub | GOPC |
| 78 | O77622 | T-complex protein 1 subunit zeta OS=Oryctolagus c | CCT6 | RABBIT | 1.17703 | 0.0159 | 0.55644 | 0.17633 | 0.71368 | -1.88757 | -0.16893 | -0.14348 | -0.0496 | 0.0496 | -0.96787 | -2.3165 | -2.23797 | 0.04919 | 0.04701 | 0.14906 | 0.14585 | 8 | 2.93229 | 0.55262 | 0.43643 | 0.467593 | Significant | Down mtub | CCT6 |
| 453 | Q95J97 | cAMP-dependent protein kinase catalytic subunit al | #N/A | RABBIT | 0.82828 | 0.81475 | -0.13404 | 0.71153 | 1.01267 | -1.1677 | 0.04677 | -0.20913 | -0.04677 | -0.19169 | -0.96034 | -3.2325 | -2.72853 | 0.01201 | 0.01973 | 0.09752 | 0.10842 | 8 | 2.93229 | 0.55262 | 0.22066 | 0.309695 | Significant | Down mtub | #N/A |
| 43 | G1TMS5 | T-complex protein 1 subunit epsilon OS=Oryctolagu | CCT5 | RABBIT | 0.61127 | -0.20699 | 0.3978 | 0.08181 | 0.91133 | -1.9099 | -0.13945 | 0.08792 | -0.46704 | -0.44188 | -0.93312 | -2.32991 | -2.22005 | 0.04817 | 0.04851 | 0.14885 | 0.14824 | 8 | 2.93229 | 0.55262 | 0.40099 | 0.441658 | Significant | Down mtub | CCT5 |
| 898 | G1TSM0 | AP-1 complex subunit mu-2 OS=Oryctolagus cunic | AP1M2 | RABBIT | 0.80964 | 0.61293 | 0.17065 | 0.38638 | 0.35414 | #N/A | -0.15141 | -0.32642 | -0.78268 | -0.47643 | -0.90098 | -5.23613 | -2.93426 | 0.0012 | 0.01503 | 0.04885 | 0.09934 | 7 | 2.93229 | 0.55262 | 0.0658 | 0.209519 | Significant | Down mtub | AP1M2 |
| 336 | O77768 | Heterogeneous nuclear ribonucleoprotein C OS=Or | HNRNPC | RABBIT | 0.26603 | 0.17146 | 0.3632 | 0.11534 | 0.59411 | -1.89682 | -0.2717 | -0.25322 | -0.53942 | 0.02636 | -0.88899 | -2.54143 | -2.30437 | 0.03463 | 0.04185 | 0.13411 | 0.13567 | 8 | 2.93229 | 0.55262 | 0.3059 | 0.372074 | Significant | Down mtub | HNRNPC |
| 8 | A0A5F9C503 | Heat shock protein HSP 90-alpha OS=Oryctolagus c | HSP90AA1 | RABBIT | 0.94866 | 0.80016 | 0.17338 | 0.59113 | -0.01611 | -1.15096 | 0.07445 | -0.25425 | -0.2747 | -0.29926 | -0.88039 | -3.20718 | -2.60256 | 0.01248 | 0.02469 | 0.09752 | 0.11504 | 8 | 2.93229 | 0.55262 | 0.18838 | 0.286079 | Significant | Down mtub | HSP90AA1 |
| 215 | G1SJQ5 | Spectrin beta, non-erythrocytic 4 OS=Oryctolagus c | SPTBN4 | RABBIT | 0.33254 | -0.09189 | 0.33645 | 0.16991 | 0.36103 | #N/A | 0 | -0.66262 | -0.48185 | -1.44693 | -0.86946 | -3.08962 | -2.41862 | 0.01758 | 0.0363 | 0.1103 | 0.12876 | 7 | 2.93229 | 0.55262 | 0.17599 | 0.287178 | Significant | Down mtub | SPTBN4 |
| 1313 | A0A5F9CU21 | Rho GTPase activating protein 18 OS=Oryctolagus c | ARHGAP18 | RABBIT | 0.28574 | 0.29805 | 0.07043 | 0.46694 | 0.77827 | #N/A | -0.67536 | #N/A | -0.53114 | -0.243 | -0.86305 | -4.73006 | -2.50063 | 0.00322 | 0.03401 | 0.06421 | 0.1262 | 6 | 2.93229 | 0.55262 | 0.06242 | 0.223343 | Significant | Down mtub | ARHGAP18 |
| 186 | G1SZR6 | Cullin 3 OS=Oryctolagus cuniculus OX=9986 GN=C | CUL3 | RABBIT | 0.95899 | 0.46662 | 0.07755 | 0.17965 | 0.35882 | #N/A | -0.12119 | -0.06437 | -0.79411 | -0.57009 | -0.79577 | -3.41473 | -2.3811 | 0.01122 | 0.0387 | 0.09732 | 0.13043 | 7 | 2.93229 | 0.55262 | 0.12068 | 0.248202 | Significant | Down mtub | CUL3 |
| 342 | G1SXP5 | Homer scaffold protein 1 OS=Oryctolagus cuniculu | HOMER1 | RABBIT | 0.96793 | 0.30971 | 0.17065 | 0.48125 | 0.05917 | #N/A | -0.26306 | -0.1284 | -0.21437 | -0.85682 | -0.7634 | -3.2911 | -2.28789 | 0.01328 | 0.04534 | 0.09752 | 0.14344 | 7 | 2.93229 | 0.55262 | 0.11957 | 0.247416 | Significant | Down mtub | HOMER1 |
