# Supplementary material for: Tuberculous Meningitis Alters the Proteomic Landscape of Brain-Derived Extracellular Vesicles
Source: Cells. 2026 Sep 25;15(19):1749. doi: 10.3390/cells15191749 (PMC13664519; doi:10.3390/cells15191749)
Supplement: Supplementary file 1 [file cells-15-01749-s001.zip › Supplementary Table S2.pdf]

Rabbit gene symbol	Rabbit ensembl gene ID	rabbit gene description	human gene symbol	human ensembl gene ID	orthology type	Human orthology confidence [0 low, 1 high]	percent identity human to rabbit	percent identity rabbit to human	included in primary enrichment	exclusion or flag reason
ARF5	ENSOCUG00000024365	ARF GTPase 5 [Source:UniProtKB Gene Name;Acc:G1U4K3]	ARF5	ENSG00000004059	ortholog_one2one	1	100.0000	100.0000	Yes	Confident one-to-one ortholog
ANXA5	ENSOCUG00000017760	annexin A5 [Source:UniProtKB Gene Name;Acc:G1TED6]	ANXA5	ENSG00000164111	ortholog_one2one	1	93.1464	93.4375	Yes	Confident one-to-one ortholog
IGHM	ENSOCUG00000013533	immunoglobulin heavy constant mu [Source:UniProtKB Gene Name;Acc:A0A5F9C411]	IGHM	ENSG00000211899	ortholog_one2one	1	61.1314	70.6751	Yes	Confident one-to-one ortholog
CSRP1	ENSOCUG00000010299	cysteine and glycine rich protein 1 [Source:UniProtKB Gene Name;Acc:A0A5F9DQI6]	CSRP1	ENSG00000159176	ortholog_one2one	1	80.5085	98.4456	Yes	Confident one-to-one ortholog
LGALS3BP	ENSOCUG00000024112	galectin 3 binding protein [Source:UniProtKB Gene Name;Acc:A0A5F9DTN1]	LGALS3BP	ENSG00000108679	ortholog_one2one	1	66.9304	72.3077	Yes	Confident one-to-one ortholog
RAB1A	ENSOCUG00000017003	RAB1A, member RAS onco family [Source:UniProtKB Gene Name;Acc:A0A5F9CL06]	RAB1A	ENSG00000138069	ortholog_one2one	1	100.0000	100.0000	Yes	Confident one-to-one ortholog
PTPA	ENSOCUG00000003042	protein phosphatase 2 phosphatase activator [Source:NCBI gene (formerly Entrezgene);Acc:100009404]	PTPA	ENSG00000119383	ortholog_one2one		45.0586	83.2817	No	Flag: low or missing orthology confidence
RANGAP1	ENSOCUG00000002704	Ran GTPase activating protein 1 [Source:UniProtKB Gene Name;Acc:G1TP30]	RANGAP1	ENSG00000100401	ortholog_one2one	1	84.7619	90.9710	Yes	Confident one-to-one ortholog
PSME2	ENSOCUG00000002857	proteasome activator subunit 2 [Source:UniProtKB Gene Name;Acc:A0A5F9CRD7]	PSME2	ENSG00000100911	ortholog_one2one	1	95.3975	95.3975	Yes	Confident one-to-one ortholog
USP5	ENSOCUG00000003582	ubiquitin specific peptidase 5 [Source:UniProtKB Gene Name;Acc:G1SK00]	USP5	ENSG00000111667	ortholog_one2one	1	81.9444	89.3939	Yes	Confident one-to-one ortholog
RRM2	ENSOCUG00000005820	ribonucleotide reductase regulatory subunit M2 [Source:UniProtKB Gene Name;Acc:G1SPQ7]	RRM2	ENSG00000171848	ortholog_one2one	1	84.1962	79.4344	Yes	Confident one-to-one ortholog
CHORDC1	ENSOCUG00000014147	cysteine and histidine rich domain containing 1 [Source:UniProtKB Gene Name;Acc:G1T6T5]	CHORDC1	ENSG00000110172	ortholog_one2many	1	94.5619	94.2771	No	Flag: ortholog_one2many
CFL2	ENSOCUG00000009383	cofilin 2 [Source:UniProtKB Gene Name;Acc:A0A5F9CKA3]	CFL2	ENSG00000165410	ortholog_one2one	1	84.6154	99.3976	Yes	Confident one-to-one ortholog
HSPA5	ENSOCUG00000011636	heat shock protein family A (Hsp70) member 5 [Source:UniProtKB Gene Name;Acc:A0A5F9C804]	HSPA5	ENSG00000044574	ortholog_one2one	1	99.5363	98.4709	Yes	Confident one-to-one ortholog
LMO7	ENSOCUG00000006793	LIM domain 7 [Source:UniProtKB Gene Name;Acc:A0A5F9CCE3]	LMO7	ENSG00000136153	ortholog_one2one	1	68.0457	76.6401	Yes	Confident one-to-one ortholog
HNRNPC	ENSOCUG00000015029	heterous nuclear ribonucleoprotein C [Source:NCBI gene (formerly Entrezgene);Acc:100008686]	HNRNPCL1	ENSG00000179172	ortholog_many2many		86.2745	90.1024	No	Flag: ortholog_many2many
HNRNPC	ENSOCUG00000015029	heterous nuclear ribonucleoprotein C [Source:NCBI gene (formerly Entrezgene);Acc:100008686]	HNRNPCL2	ENSG00000275774	ortholog_many2many		83.9869	87.7133	No	Flag: ortholog_many2many
HNRNPC	ENSOCUG00000015029	heterous nuclear ribonucleoprotein C [Source:NCBI gene (formerly Entrezgene);Acc:100008686]	HNRNPCL3	ENSG00000277058	ortholog_many2many		84.3137	88.0546	No	Flag: ortholog_many2many
HNRNPC	ENSOCUG00000015029	heterous nuclear ribonucleoprotein C [Source:NCBI gene (formerly Entrezgene);Acc:100008686]	HNRNPCL4	ENSG00000179412	ortholog_many2many		84.3137	88.0546	No	Flag: ortholog_many2many
HNRNPC	ENSOCUG00000015029	heterous nuclear ribonucleoprotein C [Source:NCBI gene (formerly Entrezgene);Acc:100008686]	HNRNPC	ENSG00000092199	ortholog_many2many	1	94.4444	98.6348	No	Flag: ortholog_many2many
RIC8A	ENSOCUG00000014493	RIC8 guanine nucleotide exchange factor A [Source:UniProtKB Gene Name;Acc:G1T7I8]	RIC8A	ENSG00000177963	ortholog_one2one	1	85.2590	80.6026	Yes	Confident one-to-one ortholog
PDIA6	ENSOCUG00000012991	protein disulfide isomerase family A member 6 [Source:UniProtKB Gene Name;Acc:G1T4H3]	PDIA6	ENSG00000143870	ortholog_one2one	1	92.5000	92.5000	Yes	Confident one-to-one ortholog
CTSB	ENSOCUG00000016580	cathepsin B [Source:UniProtKB Gene Name;Acc:A0A5F9C4V2]	CTSB	ENSG00000164733	ortholog_one2one	1	56.1404	75.5162	Yes	Confident one-to-one ortholog
LDHA	ENSOCUG00000015939	lactate dehydrogenase A [Source:UniProtKB Gene Name;Acc:A0A5F9D4H9]							No	No human ortholog returned
RUVBL2	ENSOCUG00000006427	RuvB like AAA ATPase 2 [Source:UniProtKB Gene Name;Acc:G1SQZ4]	RUVBL2	ENSG00000183207	ortholog_one2one	1	99.3521	99.3521	Yes	Confident one-to-one ortholog
MTHFD1	ENSOCUG00000011720	ethylenetetrahydrofolate dehydrogenase, cyclohydrolase and formyltetrahydrofolate synthetase 1 [Source:UniProtKB Gene Name;Acc:G1T1U	MTHFD1	ENSG00000100714	ortholog_one2one	1	92.8342	92.8342	Yes	Confident one-to-one ortholog
TIMP1	ENSOCUG00000010718	TIMP metallopeptidase inhibitor 1 [Source:UniProtKB Gene Name;Acc:A0A5F9CLN4]	TIMP1	ENSG00000102265	ortholog_one2one	1	65.2344	80.6763	Yes	Confident one-to-one ortholog
FIS1	ENSOCUG00000023720	fission, mitochondrial 1 [Source:UniProtKB Gene Name;Acc:G1TM35]	FIS1	ENSG00000214253	ortholog_one2one	1	63.2850	86.1842	Yes	Confident one-to-one ortholog
LCP1	ENSOCUG00000017943	lymphocyte cytosolic protein 1 [Source:UniProtKB Gene Name;Acc:A0A5F9DLF3]	LCP1	ENSG00000136167	ortholog_one2one	1	96.6981	98.0861	Yes	Confident one-to-one ortholog
EIF4A1	ENSOCUG00000010375	eukaryotic translation initiation factor 4A1 [Source:UniProtKB Gene Name;Acc:A0A5F9DM90]							No	No human ortholog returned
VPS51	ENSOCUG00000000816	VPS51 subunit of GARP complex [Source:UniProtKB Gene Name;Acc:G1SE49]	VPS51	ENSG00000149823	ortholog_one2one	1	89.9058	85.4220	Yes	Confident one-to-one ortholog
HINT1	ENSOCUG00000006607	histidine triad nucleotide binding protein 1 [Source:NCBI gene (formerly Entrezgene);Acc:100009302]	HINT1	ENSG00000169567	ortholog_one2many	1	96.0317	96.0317	No	Flag: ortholog_one2many
VPS26A	ENSOCUG00000001513	VPS26 retromer complex component A [Source:UniProtKB Gene Name;Acc:G1TM00]	VPS26A	ENSG00000122958	ortholog_one2one	1	99.3884	99.3884	Yes	Confident one-to-one ortholog
GSN	ENSOCUG00000013676	gelsolin [Source:UniProtKB Gene Name;Acc:A0A5F9C0T5]	GSN	ENSG00000148180	ortholog_one2one	1	81.7633	92.6129	Yes	Confident one-to-one ortholog
EIF3B	ENSOCUG00000010289	eukaryotic translation initiation factor 3 subunit B [Source:UniProtKB Gene Name;Acc:G1SZ03]	EIF3B	ENSG00000106263	ortholog_one2one	1	97.6436	81.4496	Yes	Confident one-to-one ortholog
CAPN1	ENSOCUG00000003122	calpain 1 [Source:UniProtKB Gene Name;Acc:G1SJ23]	CAPN1	ENSG00000014216	ortholog_one2one	1	96.0061	87.5350	Yes	Confident one-to-one ortholog
ACLY	ENSOCUG00000013212	ATP citrate lyase [Source:UniProtKB Gene Name;Acc:A0A5F9D719]	ACLY	ENSG00000131473	ortholog_one2one	1	94.0760	96.6394	Yes	Confident one-to-one ortholog
RAB14	ENSOCUG00000013654	RAB14, member RAS onco family [Source:UniProtKB Gene Name;Acc:G1T5V3]	RAB14	ENSG00000119396	ortholog_one2one	1	100.0000	100.0000	Yes	Confident one-to-one ortholog
ZDHHC6	ENSOCUG00000003602	zinc finger DHHC-type palmitoyltransferase 6 [Source:UniProtKB Gene Name;Acc:A0A5F9DRV9]	ZDHHC6	ENSG00000023041	ortholog_one2one	1	96.1259	96.1259	Yes	Confident one-to-one ortholog
SPATA2	ENSOCUG00000021907	spermatosis associated 2 [Source:UniProtKB Gene Name;Acc:G1TGG4]	SPATA2	ENSG00000158480	ortholog_one2one	1	80.7466	79.0385	Yes	Confident one-to-one ortholog
MVP	ENSOCUG00000008668	major vault protein [Source:UniProtKB Gene Name;Acc:G1SVM1]	MVP	ENSG00000013364	ortholog_one2one	1	90.7865	90.4815	Yes	Confident one-to-one ortholog
RIPOR2	ENSOCUG00000001481	RHO family interacting cell polarization regulator 2 [Source:UniProtKB Gene Name;Acc:G1SFI8]	RIPOR2	ENSG00000111913	ortholog_one2one	1	82.2523	87.2015	Yes	Confident one-to-one ortholog
SERPINA1	ENSOCUG00000026257	serpin family A member 1 [Source:UniProtKB Gene Name;Acc:A0A5F9CCV3]	SERPINA1	ENSG00000197249	ortholog_one2one	1	58.7336	64.3541	Yes	Confident one-to-one ortholog
PFN1	ENSOCUG00000001106	profilin 1 [Source:HGNC Symbol;Acc:HGNC:8881]	PFN1	ENSG00000108518	ortholog_one2one	1	91.3462	67.8571	Yes	Confident one-to-one ortholog
NUDT21	ENSOCUG00000017224	nudix hydrolase 21 [Source:UniProtKB Gene Name;Acc:G1TD91]	NUDT21	ENSG00000167005	ortholog_one2one	1	99.5595	99.5595	Yes	Confident one-to-one ortholog
RAB5C	ENSOCUG00000013307	RAB5C, member RAS onco family [Source:UniProtKB Gene Name;Acc:G1T550]	RAB5C	ENSG00000108774	ortholog_one2one	1	99.0741	99.0741	Yes	Confident one-to-one ortholog
IQGAP1	ENSOCUG00000000146	IQ motif containing GTPase activating protein 1 [Source:UniProtKB Gene Name;Acc:G1SCP7]	IQGAP1	ENSG00000140575	ortholog_one2one	1	92.8199	96.7411	Yes	Confident one-to-one ortholog
ITGB1	ENSOCUG00000003623	integrin subunit beta 1 [Source:UniProtKB Gene Name;Acc:A0A5F9DJW7]	ITGB1	ENSG00000150093	ortholog_one2one	1	90.1205	93.7343	Yes	Confident one-to-one ortholog
PRKAR2A	ENSOCUG00000012426	protein kinase cAMP-dependent type II regulatory subunit alpha [Source:UniProtKB Gene Name;Acc:G1U522]	PRKAR2A	ENSG00000114302	ortholog_one2one	1	87.5312	86.8812	Yes	Confident one-to-one ortholog
YWHAB	ENSOCUG00000003002	tyrosine 3-monooxygenase/tryptophan 5-monooxygenase activation protein beta [Source:UniProtKB Gene Name;Acc:A0A5F9CN85]	YWHAB	ENSG00000166913	ortholog_one2one	1	65.4494	94.7154	Yes	Confident one-to-one ortholog
ALDOA	ENSOCUG00000006329	aldolase, fructose-bisphosphate A [Source:NCBI gene (formerly Entrezgene);Acc:100009055]	ALDOA	ENSG00000149925	ortholog_one2one	1	95.9427	96.1722	Yes	Confident one-to-one ortholog
COMMD5	ENSOCUG00000006483	COMM domain containing 5 [Source:UniProtKB Gene Name;Acc:G1SR33]	COMMD5	ENSG00000170619	ortholog_one2one	1	83.5616	81.6964	Yes	Confident one-to-one ortholog
ARHGAP35	ENSOCUG00000017126	Rho GTPase activating protein 35 [Source:UniProtKB Gene Name;Acc:G1TD24]	ARHGAP35	ENSG00000160007	ortholog_one2one	1	94.4170	89.1261	Yes	Confident one-to-one ortholog
EML4	ENSOCUG00000015526	EMAP like 4 [Source:UniProtKB Gene Name;Acc:A0A5F9CH75]	EML4	ENSG00000143924	ortholog_one2one	1	85.8365	92.0489	Yes	Confident one-to-one ortholog
VPS13A	ENSOCUG00000001328	vacuolar protein sorting 13 homolog A [Source:UniProtKB Gene Name;Acc:A0A5F9CU68]	VPS13A	ENSG00000197969	ortholog_one2one	1	89.3375	89.2250	Yes	Confident one-to-one ortholog
GIGYF2	ENSOCUG00000001982	GRB10 interacting GYF protein 2 [Source:UniProtKB Gene Name;Acc:G1SGK1]	GIGYF2	ENSG00000204120	ortholog_one2one	1	93.9394	93.0716	Yes	Confident one-to-one ortholog
CTSA	ENSOCUG00000000740	cathepsin A [Source:UniProtKB Gene Name;Acc:A0A5F9C3V2]	CTSA	ENSG00000064601	ortholog_one2one	1	78.4836	79.7917	Yes	Confident one-to-one ortholog
BZW2	ENSOCUG00000016419	basic leucine zipper and W2 domains 2 [Source:UniProtKB Gene Name;Acc:A0A5F9CNB5]	BZW2	ENSG00000136261	ortholog_one2one	1	89.0380	94.9881	Yes	Confident one-to-one ortholog
PLTP	ENSOCUG00000000744	phospholipid transfer protein [Source:UniProtKB Gene Name;Acc:A0A5F9C720]	PLTP	ENSG00000100979	ortholog_one2one	1	67.6101	87.2211	Yes	Confident one-to-one ortholog
PTPRA	ENSOCUG00000021376	protein tyrosine phosphatase receptor type A [Source:UniProtKB Gene Name;Acc:G1TXJ3]	PTPRA	ENSG00000132670	ortholog_one2one	1	94.1176	95.7606	Yes	Confident one-to-one ortholog
ACTA1	ENSOCUG00000007566	actin alpha 1, skeletal muscle [Source:UniProtKB Gene Name;Acc:G1SL06]	ACTA1	ENSG00000143632	ortholog_one2one	1	99.7290	97.6127	Yes	Confident one-to-one ortholog
OAT	ENSOCUG00000013547	ornithine aminotransferase [Source:UniProtKB Gene Name;Acc:G1T5M0]	OAT	ENSG00000065154	ortholog_one2one	1	86.1233	89.0661	Yes	Confident one-to-one ortholog
HTT	ENSOCUG00000009805	huntingtin [Source:UniProtKB Gene Name;Acc:U3KPH8]	HTT	ENSG00000197386	ortholog_one2one	1	91.3601	88.5105	Yes	Confident one-to-one ortholog
HSPB6	ENSOCUG00000021287	heat shock protein family B (small) member 6 [Source:UniProtKB Gene Name;Acc:G1U1F6]	HSPB6	ENSG00000004776	ortholog_one2one	1	63.0208	75.6250	Yes	Confident one-to-one ortholog
ABCA3	ENSOCUG00000005137	ATP binding cassette subfamily A member 3 [Source:UniProtKB Gene Name;Acc:A0A5F9DGT8]	ABCA3	ENSG00000167972	ortholog_one2one	1	81.9074	88.2042	Yes	Confident one-to-one ortholog
ACTN4	ENSOCUG00000022448	actinin alpha 4 [Source:UniProtKB Gene Name;Acc:A0A5F9D4S1]	ACTN4	ENSG00000130402	ortholog_one2one	1	93.7213	67.1789	Yes	Confident one-to-one ortholog
MRI1	ENSOCUG00000024840	methylthioribose-1-phosphate isomerase 1 [Source:UniProtKB Gene Name;Acc:G1TPW2]	MRI1	ENSG00000037757	ortholog_one2one	1	85.7534	84.8238	Yes	Confident one-to-one ortholog
BLVRB	ENSOCUG00000002791	biliverdin reductase B [Source:UniProtKB Gene Name;Acc:A0A5F9DBL7]	BLVRB	ENSG00000090013	ortholog_one2one	1	51.4377	78.1553	Yes	Confident one-to-one ortholog
ARPC3	ENSOCUG00000011908	actin related protein 2/3 complex subunit 3 [Source:UniProtKB Gene Name;Acc:G1T277]	ARPC3	ENSG00000111229	ortholog_one2one	1	100.0000	100.0000	Yes	Confident one-to-one ortholog
IDH2	ENSOCUG00000010508	isocitrate dehydrogenase (NADP(+)) 2 [Source:UniProtKB Gene Name;Acc:G1SZF7]	IDH2	ENSG00000182054	ortholog_one2one	1	87.0213	90.4867	Yes	Confident one-to-one ortholog
FBL	ENSOCUG00000009027	fibrillarin [Source:UniProtKB Gene Name;Acc:G1SWD1]	FBL	ENSG00000105202	ortholog_one2one	1	90.3125	90.0312	Yes	Confident one-to-one ortholog
EIF3L	ENSOCUG00000000941	eukaryotic translation initiation factor 3 subunit L [Source:UniProtKB Gene Name;Acc:A0A5F9D9H1]	EIF3L	ENSG00000100129	ortholog_one2one	1	92.0923	99.1135	Yes	Confident one-to-one ortholog
ITGB4	ENSOCUG00000014951	integrin subunit beta 4 [Source:UniProtKB Gene Name;Acc:G1T8G1]	ITGB4	ENSG00000132470	ortholog_one2one	1	86.7682	81.3392	Yes	Confident one-to-one ortholog
PRKAR1A	ENSOCUG00000017419	protein kinase cAMP-dependent type I regulatory subunit alpha [Source:UniProtKB Gene Name;Acc:G1TDN4]	PRKAR1A	ENSG00000108946	ortholog_one2one	1	98.9501	98.9501	Yes	Confident one-to-one ortholog
HNRNPC	ENSOCUG00000000339	heterous nuclear ribonucleoprotein C [Source:UniProtKB Gene Name;Acc:O77768]	HNRNPCL1	ENSG00000179172	ortholog_many2many	1	86.2745	90.1024	No	Flag: ortholog_many2many

HNRNPC	ENSOCUG0000000339	heterous nuclear ribonucleoprotein C [Source:UniProtKB Gene Name;Acc:O77768]	HNRNPCL2	ENSG00000275774	ortholog_many2many	1	83.9869	87.7133	No	Flag: ortholog_many2many
HNRNPC	ENSOCUG0000000339	heterous nuclear ribonucleoprotein C [Source:UniProtKB Gene Name;Acc:O77768]	HNRNPCL3	ENSG00000277058	ortholog_many2many	1	84.3137	88.0546	No	Flag: ortholog_many2many
HNRNPC	ENSOCUG0000000339	heterous nuclear ribonucleoprotein C [Source:UniProtKB Gene Name;Acc:O77768]	HNRNPCL4	ENSG00000179412	ortholog_many2many	1	84.3137	88.0546	No	Flag: ortholog_many2many
HNRNPC	ENSOCUG0000000339	heterous nuclear ribonucleoprotein C [Source:UniProtKB Gene Name;Acc:O77768]	HNRNPC	ENSG00000092199	ortholog_many2many		94.4444	98.6348	No	Flag: ortholog_many2many
EHD1	ENSOCUG0000029532	EH domain containing 1 [Source:UniProtKB Gene Name;Acc:U3KMU7]	EHD1	ENSG00000110047	ortholog_one2one	1	96.3504	98.8764	Yes	Confident one-to-one ortholog
PDIA3	ENSOCUG0000001155	protein disulfide isomerase family A member 3 [Source:NCBI gene (formerly Entrezgene);Acc:100328685]	PDIA3	ENSG00000167004	ortholog_one2one	1	96.4143	95.8416	Yes	Confident one-to-one ortholog
LRRFIP1	ENSOCUG0000027499	LRR binding FLII interacting protein 1 [Source:HGNC Symbol;Acc:HGNC:6702]	LRRFIP1	ENSG00000124831	ortholog_one2one	1	50.8639	73.5938	Yes	Confident one-to-one ortholog
CSE1L	ENSOCUG0000005459	chromosome segregation 1 like [Source:UniProtKB Gene Name;Acc:A0A5F9D376]	CSE1L	ENSG00000124207	ortholog_one2one	1	92.9672	99.3821	Yes	Confident one-to-one ortholog
PAFAH1B3	ENSOCUG0000006470	platelet activating factor acetylhydrolase 1b catalytic subunit 3 [Source:UniProtKB Gene Name;Acc:G1SR28]	PAFAH1B3	ENSG00000079462	ortholog_one2one	1	96.9697	96.9697	Yes	Confident one-to-one ortholog
PSMG1	ENSOCUG0000008866	proteasome assembly chaperone 1 [Source:NCBI gene (formerly Entrezgene);Acc:100328650]	PSMG1	ENSG00000183527	ortholog_one2one	1	85.6557	72.5694	Yes	Confident one-to-one ortholog
HSPH1	ENSOCUG0000014773	heat shock protein family H (Hsp110) member 1 [Source:UniProtKB Gene Name;Acc:A0A5F9CER0]	HSPH1	ENSG00000120694	ortholog_one2one	1	94.3613	95.5711	Yes	Confident one-to-one ortholog
VPS35	ENSOCUG0000010484	VPS35 retromer complex component [Source:UniProtKB Gene Name;Acc:G1SZE0]	VPS35	ENSG00000069329	ortholog_one2one	1	99.7487	99.7487	Yes	Confident one-to-one ortholog
CORO7	ENSOCUG0000000193	coronin 7 [Source:UniProtKB Gene Name;Acc:A0A5F9CC05]	CORO7	ENSG00000262246	ortholog_one2one	1	76.2857	86.5946	Yes	Confident one-to-one ortholog
HNRNPK	ENSOCUG0000010364	heterous nuclear ribonucleoprotein K [Source:NCBI gene (formerly Entrezgene);Acc:100008849]							No	No human ortholog returned
TUBB2A	ENSOCUG0000009827	tubulin beta 2A class IIa [Source:UniProtKB Gene Name;Acc:G1T4J4]	TUBB2A	ENSG00000137267	ortholog_one2one	1	65.4450	84.2697	Yes	Confident one-to-one ortholog
IDH3A	ENSOCUG0000015765	isocitrate dehydrogenase (NAD(+)) 3 catalytic subunit alpha [Source:UniProtKB Gene Name;Acc:A0A5F9DEX9]	IDH3A	ENSG00000166411	ortholog_one2one	1	73.2759	92.8962	Yes	Confident one-to-one ortholog
SERPINB9	ENSOCUG0000005091	serpin family B member 9 [Source:UniProtKB Gene Name;Acc:G1SN70]	SERPINB9	ENSG00000170542	ortholog_one2one	1	77.1277	77.1277	Yes	Confident one-to-one ortholog
CRABP1	ENSOCUG0000000431	cellular retinoic acid binding protein 1 [Source:UniProtKB Gene Name;Acc:G1SDA2]	CRABP1	ENSG00000166426	ortholog_one2one	1	99.2701	99.2701	Yes	Confident one-to-one ortholog
FBXO6	ENSOCUG0000024728	F-box protein 6 [Source:UniProtKB Gene Name;Acc:A0A5F9C5U2]	FBXO6	ENSG00000116663	ortholog_one2one	1	61.6959	72.0136	Yes	Confident one-to-one ortholog
SFTPD	ENSOCUG0000027260	surfactant protein D [Source:UniProtKB Gene Name;Acc:G1TK26]	SFTPD	ENSG00000133661	ortholog_one2one	1	71.1429	66.4000	Yes	Confident one-to-one ortholog
CANX	ENSOCUG0000015091	calnexin [Source:UniProtKB Gene Name;Acc:G1U6H0]	CANX	ENSG00000127022	ortholog_one2one	1	90.5145	95.1013	Yes	Confident one-to-one ortholog
SEPTIN2	ENSOCUG0000015500	septin 2 [Source:UniProtKB Gene Name;Acc:G1U0Q7]	SEPTIN2	ENSG00000168385	ortholog_one2one	1	96.7213	98.0609	Yes	Confident one-to-one ortholog
PSMA6	ENSOCUG0000015635	proteasome 20S subunit alpha 6 [Source:UniProtKB Gene Name;Acc:G1T9V4]	PSMA6	ENSG00000100902	ortholog_one2one	1	84.0909	90.2439	Yes	Confident one-to-one ortholog
ACTG1	ENSOCUG0000010443	actin gamma 1 [Source:UniProtKB Gene Name;Acc:A0A5F9DGZ0]	ACTB	ENSG00000075624	ortholog_one2one		70.8978	61.0667	No	Flag: low or missing orthology confidence
ATP5F1B	ENSOCUG0000006090	ATP synthase F1 subunit beta [Source:UniProtKB Gene Name;Acc:G1SQA8]	ATP5F1B	ENSG00000110955	ortholog_one2one	1	97.3485	97.1645	Yes	Confident one-to-one ortholog
PTGES3	ENSOCUG0000035555	prostaglandin E synthase 3 [Source:UniProtKB Gene Name;Acc:G1TGF1]							No	No human ortholog returned
PSMD6	ENSOCUG0000025313	proteasome 26S subunit, non-ATPase 6 [Source:UniProtKB Gene Name;Acc:G1U115]	PSMD6	ENSG00000163636	ortholog_one2one	1	98.7147	98.7147	Yes	Confident one-to-one ortholog
WDR13	ENSOCUG0000014470	WD repeat domain 13 [Source:UniProtKB Gene Name;Acc:G1U7G7]	WDR13	ENSG00000101940	ortholog_one2one	1	95.6067	94.2268	Yes	Confident one-to-one ortholog
NDRG1	ENSOCUG0000016394	N-myc downstream regulated 1 [Source:UniProtKB Gene Name;Acc:A0A5F9DPK1]	NDRG1	ENSG00000104419	ortholog_one2one	1	86.5297	96.1929	Yes	Confident one-to-one ortholog
SLC3A2	ENSOCUG0000001334	solute carrier family 3 member 2 [Source:NCBI gene (formerly Entrezgene);Acc:100009363]	SLC3A2	ENSG00000168003	ortholog_one2one	1	76.8827	82.9868	Yes	Confident one-to-one ortholog
LANCL2	ENSOCUG0000002094	LanC like glutathione S-transferase 2 [Source:UniProtKB Gene Name;Acc:G1SGT0]	LANCL2	ENSG00000132434	ortholog_one2one	1	82.0619	88.4444	Yes	Confident one-to-one ortholog
ADAR	ENSOCUG0000008544	adenosine deaminase RNA specific [Source:UniProtKB Gene Name;Acc:G1SVD5]	ADAR	ENSG00000160710	ortholog_one2one	1	84.0948	81.0767	Yes	Confident one-to-one ortholog
PSMD3	ENSOCUG0000009691	proteasome 26S subunit, non-ATPase 3 [Source:UniProtKB Gene Name;Acc:G1TP15]	PSMD3	ENSG00000108344	ortholog_one2one	1	76.8657	77.1536	Yes	Confident one-to-one ortholog
ENO1	ENSOCUG0000010086	enolase 1 [Source:HGNC Symbol;Acc:HGNC:3350]	ENO1	ENSG00000074800	ortholog_one2one	1	67.3190	79.2627	Yes	Confident one-to-one ortholog
CDH2	ENSOCUG0000003656	cadherin 2 [Source:UniProtKB Gene Name;Acc:G1TPS9]	CDH2	ENSG00000170558	ortholog_one2one	1	96.4871	90.9492	Yes	Confident one-to-one ortholog
TUFM	ENSOCUG0000002683	Tu translation elongation factor, mitochondrial [Source:UniProtKB Gene Name;Acc:A0A5F9CQB4]	TUFM	ENSG00000178952	ortholog_one2one	1	73.0526	76.2637	Yes	Confident one-to-one ortholog
PGRMC1	ENSOCUG0000010280	progesterone receptor membrane component 1 [Source:UniProtKB Gene Name;Acc:G1TT75]	PGRMC1	ENSG00000101856	ortholog_one2one	1	93.8462	93.8462	Yes	Confident one-to-one ortholog
RRBP1	ENSOCUG0000021320	ribosome binding protein 1 [Source:UniProtKB Gene Name;Acc:A0A5F9C7H8]	RRBP1	ENSG00000125844	ortholog_one2one	1	78.8075	77.8014	Yes	Confident one-to-one ortholog
ITGA6	ENSOCUG0000000403	integrin subunit alpha 6 [Source:UniProtKB Gene Name;Acc:G1SD83]	ITGA6	ENSG00000091409	ortholog_one2one	1	89.1099	79.3103	Yes	Confident one-to-one ortholog
GLS	ENSOCUG0000002448	glutaminase [Source:HGNC Symbol;Acc:HGNC:4331]	GLS	ENSG00000115419	ortholog_one2one	1	83.2908	97.6084	Yes	Confident one-to-one ortholog
CCDC93	ENSOCUG0000005932	coiled-coil domain containing 93 [Source:UniProtKB Gene Name;Acc:G1SPZ5]	CCDC93	ENSG00000125633	ortholog_one2one	1	85.2857	94.6117	Yes	Confident one-to-one ortholog
RAPGEFL1	ENSOCUG0000023409	Rap guanine nucleotide exchange factor like 1 [Source:UniProtKB Gene Name;Acc:A0A5F9CTY3]	RAPGEFL1	ENSG00000108352	ortholog_one2one	1	91.3793	88.0665	Yes	Confident one-to-one ortholog
SFPQ	ENSOCUG0000001877	splicing factor proline and glutamine rich [Source:UniProtKB Gene Name;Acc:G1SGB5]	SFPQ	ENSG00000116560	ortholog_one2one	1	90.2752	69.5898	Yes	Confident one-to-one ortholog
CLU	ENSOCUG0000005985	clusterin [Source:NCBI gene (formerly Entrezgene);Acc:100008713]	CLU	ENSG00000120885	ortholog_one2one	1	66.6023	76.8374	Yes	Confident one-to-one ortholog
SRPK1	ENSOCUG0000008660	SRSF protein kinase 1 [Source:UniProtKB Gene Name;Acc:A0A5F9D9S2]	SRPK1	ENSG00000096063	ortholog_one2one	1	76.3571	96.6412	Yes	Confident one-to-one ortholog
PPP1CB	ENSOCUG0000001023	protein phosphatase 1 catalytic subunit beta [Source:UniProtKB Gene Name;Acc:A0A5F9D840]	PPP1CB	ENSG00000213639	ortholog_one2one	1	87.1508	95.4128	Yes	Confident one-to-one ortholog
HSPB1	ENSOCUG0000012690	heat shock protein family B (small) member 1 [Source:UniProtKB Gene Name;Acc:G1T3V2]	HSPB1	ENSG00000106211	ortholog_one2one	1	91.6667	91.2195	Yes	Confident one-to-one ortholog
MAP2K1	ENSOCUG0000008782	mitogen-activated protein kinase kinase 1 [Source:UniProtKB Gene Name;Acc:G1SVV6]	MAP2K1	ENSG00000169032	ortholog_one2one	1	92.6768	93.3842	Yes	Confident one-to-one ortholog
MMS19	ENSOCUG0000007776	MMS19 homolog, cytosolic iron-sulfur assembly component [Source:UniProtKB Gene Name;Acc:G1STS3]	MMS19	ENSG00000155229	ortholog_one2one	1	92.8155	92.8155	Yes	Confident one-to-one ortholog
CAST	ENSOCUG0000007802	calpastatin [Source:NCBI gene (formerly Entrezgene);Acc:100009098]	CAST	ENSG00000310517	ortholog_one2one	1	71.3244	74.2099	Yes	Confident one-to-one ortholog
RAB11A	ENSOCUG0000014755	RAB11A, member RAS onco family [Source:NCBI gene (formerly Entrezgene);Acc:100009072]	RAB11A	ENSG00000103769	ortholog_one2one	1	63.0435	67.1296	Yes	Confident one-to-one ortholog
OGT	ENSOCUG0000016965	O-linked N-acetylglucosamine (GlcNAc) transferase [Source:UniProtKB Gene Name;Acc:P81436]	OGT	ENSG00000147162	ortholog_one2one	1	98.6654	98.9484	Yes	Confident one-to-one ortholog
CCT2	ENSOCUG0000017199	chaperonin containing TCP1 subunit 2 [Source:NCBI gene (formerly Entrezgene);Acc:100216334]	CCT2	ENSG00000166226	ortholog_one2one	1	91.6667	98.6916	Yes	Confident one-to-one ortholog
QARS1	ENSOCUG0000005082	glutaminyl-tRNA synthetase 1 [Source:UniProtKB Gene Name;Acc:G1SN68]	QARS1	ENSG00000172053	ortholog_one2one	1	93.2903	93.2903	Yes	Confident one-to-one ortholog
NAP1L1	ENSOCUG0000013857	nucleosome assembly protein 1 like 1 [Source:UniProtKB Gene Name;Acc:A0A5F9DPT9]	NAP1L1	ENSG00000187109	ortholog_one2one	1	85.1685	96.9309	Yes	Confident one-to-one ortholog
MYO1E	ENSOCUG0000015835	myosin IE [Source:UniProtKB Gene Name;Acc:G1TAB8]	MYO1E	ENSG00000157483	ortholog_one2one	1	97.4706	97.3827	Yes	Confident one-to-one ortholog
LAMTOR3	ENSOCUG0000001152	late endosomal/lysosomal adaptor, MAPK and MTOR activator 3 [Source:UniProtKB Gene Name;Acc:G1SEU9]	LAMTOR3	ENSG00000109270	ortholog_one2one	1	97.5806	97.5806	Yes	Confident one-to-one ortholog
MYH9	ENSOCUG0000004142	myosin heavy chain 9 [Source:UniProtKB Gene Name;Acc:A0A5F9CQZ3]	MYH9	ENSG00000100345	ortholog_one2one	1	97.9167	95.9184	Yes	Confident one-to-one ortholog
DES	ENSOCUG0000000968	desmin [Source:UniProtKB Gene Name;Acc:G1SEF9]	DES	ENSG00000175084	ortholog_one2one	1	90.2534	98.5106	Yes	Confident one-to-one ortholog
PSMA3	ENSOCUG0000010316	proteasome 20S subunit alpha 3 [Source:UniProtKB Gene Name;Acc:G1SZ14]	PSMA3	ENSG00000100567	ortholog_one2one	1	77.3006	98.8235	Yes	Confident one-to-one ortholog
CAPZB	ENSOCUG0000014711	capping actin protein of muscle Z-line subunit beta [Source:HGNC Symbol;Acc:HGNC:1491]	CAPZB	ENSG00000077549	ortholog_one2one	1	89.7010	99.2647	Yes	Confident one-to-one ortholog
HSPA9	ENSOCUG0000006655	heat shock protein family A (Hsp70) member 9 [Source:UniProtKB Gene Name;Acc:G1SRF7]	HSPA9	ENSG00000113013	ortholog_one2one	1	98.5272	98.5272	Yes	Confident one-to-one ortholog
KIF21A	ENSOCUG0000016630	kinesin family member 21A [Source:UniProtKB Gene Name;Acc:A0A5F9DEY4]	KIF21A	ENSG00000139116	ortholog_one2one	1	95.1057	94.0263	Yes	Confident one-to-one ortholog
SDCBP	ENSOCUG0000016206	syndecan binding protein [Source:UniProtKB Gene Name;Acc:G1TB50]	SDCBP	ENSG00000137575	ortholog_one2one	1	84.9359	88.9262	Yes	Confident one-to-one ortholog
DYNC1I1	ENSOCUG0000012213	dynein cytoplasmic 1 intermediate chain 1 [Source:UniProtKB Gene Name;Acc:A0A5F9DA29]	DYNC1I1	ENSG00000158560	ortholog_one2one	1	94.3338	98.0892	Yes	Confident one-to-one ortholog
RAB5A	ENSOCUG0000012200	RAB5A, member RAS onco family [Source:UniProtKB Gene Name;Acc:G1T2T9]	RAB5A	ENSG00000144566	ortholog_one2one	1	98.6047	98.6047	Yes	Confident one-to-one ortholog
PDK4	ENSOCUG0000017120	pyruvate dehydrogenase kinase 4 [Source:UniProtKB Gene Name;Acc:G1TD19]	PDK4	ENSG00000004799	ortholog_one2one	1	95.6311	95.8637	Yes	Confident one-to-one ortholog
RPS6	ENSOCUG0000003336	ribosomal protein S6 [Source:UniProtKB Gene Name;Acc:G1TM55]	RPS6	ENSG00000137154	ortholog_one2one	1	100.0000	100.0000	Yes	Confident one-to-one ortholog
ATL1	ENSOCUG0000017706	atlastin GTPase 1 [Source:UniProtKB Gene Name;Acc:G1TE90]	ATL1	ENSG00000198513	ortholog_one2one	1	88.5852	98.7455	Yes	Confident one-to-one ortholog
GRN	ENSOCUG0000013567	granulin [Source:UniProtKB Gene Name;Acc:A0A5F9C723]	GRN	ENSG00000030582	ortholog_one2one	1	73.6422	77.7403	Yes	Confident one-to-one ortholog
RTN4	ENSOCUG0000012050	reticulon 4 [Source:UniProtKB Gene Name;Acc:A0A5F9C4A3]	RTN4	ENSG00000115310	ortholog_one2one	1	78.7387	73.3222	Yes	Confident one-to-one ortholog
PON1	ENSOCUG0000005136	paraoxonase 1 [Source:NCBI gene (formerly Entrezgene);Acc:100009133]	PON1	ENSG00000005421	ortholog_one2one	1	84.6797	85.6338	Yes	Confident one-to-one ortholog
S100A10	ENSOCUG0000005198	S100 calcium binding protein A10 [Source:NCBI gene (formerly Entrezgene);Acc:100008922]	S100A10	ENSG00000197747	ortholog_one2one		48.9899	100.0000	No	Flag: low or missing orthology confidence
CTNNA1	ENSOCUG0000007744	catenin alpha 1 [Source:NCBI gene (formerly Entrezgene);Acc:100328952]	CTNNA1	ENSG00000044115	ortholog_one2one	1	90.5337	99.2274	Yes	Confident one-to-one ortholog
HSP90B1	ENSOCUG0000016288	heat shock protein 90 beta family member 1 [Source:UniProtKB Gene Name;Acc:G1TBC1]	HSP90B1	ENSG00000166598	ortholog_one2one	1	97.6309	97.5093	Yes	Confident one-to-one ortholog
CCT5	ENSOCUG0000021231	chaperonin containing TCP1 subunit 5 [Source:UniProtKB Gene Name;Acc:G1TMS5]	CCT5	ENSG00000150753	ortholog_one2one	1	98.5213	98.5213	Yes	Confident one-to-one ortholog
EIF2S1	ENSOCUG0000012023	eukaryotic translation initiation factor 2 subunit alpha [Source:UniProtKB Gene Name;Acc:A0A5F9CHW6]	EIF2S1	ENSG00000134001	ortholog_one2one	1	99.3151	92.0635	Yes	Confident one-to-one ortholog
ARHGAP1	ENSOCUG0000016160	Rho GTPase activating protein 1 [Source:UniProtKB Gene Name;Acc:G1TB18]	ARHGAP1	ENSG00000175220	ortholog_one2one	1	94.7727	94.9886	Yes	Confident one-to-one ortholog
MADD	ENSOCUG0000002099	MAP kinase activating death domain [Source:UniProtKB Gene Name;Acc:A0A5F9DQD6]	MADD	ENSG00000110514	ortholog_one2one	1	96.4763	96.2424	Yes	Confident one-to-one ortholog
GC	ENSOCUG0000007986	GC vitamin D binding protein [Source:NCBI gene (formerly Entrezgene);Acc:100008994]	GC	ENSG00000145321	ortholog_one2one	1	64.7260	79.7468	Yes	Confident one-to-one ortholog
CLIC1	ENSOCUG0000022545	chloride intracellular channel 1 [Source:NCBI gene (formerly Entrezgene);Acc:100008817]	CLIC1	ENSG00000213719	ortholog_one2many	1	98.3402	98.3402	No	Flag: ortholog_one2many
NDRG2	ENSOCUG0000000653	NDRG family member 2 [Source:UniProtKB Gene Name;Acc:A0A5F9DDC5]	NDRG2	ENSG00000165795	ortholog_one2one	1	81.5909	96.7655	Yes	Confident one-to-one ortholog

PSMC3	ENSOCUG00000026980	proteasome 26S subunit, ATPase 3 [Source:UniProtKB Gene Name;Acc:A0A5F9C3R7]	PSMC3	ENSG00000165916	ortholog_one2one	1	99.3166	99.3166	Yes	Confident one-to-one ortholog
TOM1	ENSOCUG00000010626	target of myb1 membrane trafficking protein [Source:UniProtKB Gene Name;Acc:U3KM53]	TOM1	ENSG00000100284	ortholog_one2one	1	85.3229	88.6179	Yes	Confident one-to-one ortholog
MAPK10	ENSOCUG00000010663	mitogen-activated protein kinase 10 [Source:UniProtKB Gene Name;Acc:A0A5F9CM49]	MAPK10	ENSG00000109339	ortholog_one2one	1	98.0728	98.7069	Yes	Confident one-to-one ortholog
EIF3A	ENSOCUG00000004996	eukaryotic translation initiation factor 3 subunit A [Source:UniProtKB Gene Name;Acc:A0A5F9CMG0]	EIF3A	ENSG00000107581	ortholog_one2one	1	95.4479	94.0666	Yes	Confident one-to-one ortholog
UQCRC1	ENSOCUG00000002041	ubiquinol-cytochrome c reductase core protein 1 [Source:UniProtKB Gene Name;Acc:A0A5F9DIE5]	UQCRC1	ENSG00000010256	ortholog_one2one	1	80.3340	90.2083	Yes	Confident one-to-one ortholog
RAP1GDS1	ENSOCUG00000016535	Rap1 GTPase-GDP dissociation stimulator 1 [Source:UniProtKB Gene Name;Acc:G1TBU8]	RAP1GDS1	ENSG00000138698	ortholog_one2one	1	97.5207	97.1993	Yes	Confident one-to-one ortholog
VTA1	ENSOCUG00000002554	vesicle trafficking 1 [Source:UniProtKB Gene Name;Acc:A0A5F9C6U9]	VTA1	ENSG00000009844	ortholog_one2one	1	74.7851	85.0163	Yes	Confident one-to-one ortholog
EPB41L2	ENSOCUG00000001799	erythrocyte membrane protein band 4.1 like 2 [Source:UniProtKB Gene Name;Acc:A0A5F9CFP4]	EPB41L2	ENSG00000079819	ortholog_one2one	1	82.7149	90.9453	Yes	Confident one-to-one ortholog
CLIC4	ENSOCUG00000003196	chloride intracellular channel 4 [Source:UniProtKB Gene Name;Acc:A0A5F9DNN9]	CLIC4	ENSG00000169504	ortholog_one2one	1	72.3602	92.0949	Yes	Confident one-to-one ortholog
GCC2	ENSOCUG00000017849	GRIP and coiled-coil domain containing 2 [Source:UniProtKB Gene Name;Acc:G1TEK3]	GCC2	ENSG00000135968	ortholog_one2one	1	86.2233	86.2233	Yes	Confident one-to-one ortholog
AARSD1	ENSOCUG00000009259	alanyl-tRNA synthetase domain containing 1 [Source:UniProtKB Gene Name;Acc:G1TF82]	AARSD1	ENSG00000266967	ortholog_one2one	1	89.3204	89.3204	Yes	Confident one-to-one ortholog
YWHAE	ENSOCUG00000014607	tyrosine 3-monooxygenase/tryptophan 5-monooxygenase activation protein epsilon [Source:UniProtKB Gene Name;Acc:G1SIG2]	YWHAE	ENSG00000108953	ortholog_one2one	1	55.5046	94.9020	Yes	Confident one-to-one ortholog
COPS5	ENSOCUG00000011490	COP9 signalosome subunit 5 [Source:UniProtKB Gene Name;Acc:G1T1D9]	COPS5	ENSG00000121022	ortholog_one2one	1	100.0000	100.0000	Yes	Confident one-to-one ortholog
TWF2	ENSOCUG00000021993	twinfilin actin binding protein 2 [Source:UniProtKB Gene Name;Acc:A0A5F9CGX5]	TWF2	ENSG00000247596	ortholog_one2one	1	95.1289	95.1289	Yes	Confident one-to-one ortholog
ANKFY1	ENSOCUG00000014843	ankyrin repeat and FYVE domain containing 1 [Source:UniProtKB Gene Name;Acc:G1T888]	ANKFY1	ENSG00000185722	ortholog_one2one	1	92.8201	91.7878	Yes	Confident one-to-one ortholog
ATG7	ENSOCUG00000001908	autophagy related 7 [Source:UniProtKB Gene Name;Acc:A0A5F9DD12]	ATG7	ENSG00000197548	ortholog_one2one	1	76.5046	94.0256	Yes	Confident one-to-one ortholog
BRCA1	ENSOCUG00000003883	BRCA1 DNA repair associated [Source:UniProtKB Gene Name;Acc:A0A5F9DIV5]	BRCA1	ENSG00000012048	ortholog_one2one	1	69.8704	69.4579	Yes	Confident one-to-one ortholog
CKAP5	ENSOCUG00000016200	cytoskeleton associated protein 5 [Source:UniProtKB Gene Name;Acc:A0A5F9CT68]	CKAP5	ENSG00000175216	ortholog_one2one	1	97.0501	97.1457	Yes	Confident one-to-one ortholog
NAA15	ENSOCUG00000017331	N-alpha-acetyltransferase 15, NatA auxiliary subunit [Source:UniProtKB Gene Name;Acc:A0A5F9C965]	NAA15	ENSG00000164134	ortholog_one2one	1	96.9001	97.4596	Yes	Confident one-to-one ortholog
VARS1	ENSOCUG00000022463	valyl-tRNA synthetase 1 [Source:UniProtKB Gene Name;Acc:G1TWP4]	VARS1	ENSG00000204394	ortholog_one2one	1	94.5990	91.4557	Yes	Confident one-to-one ortholog
BROX	ENSOCUG00000016381	BRO1 domain and CAAX motif containing [Source:UniProtKB Gene Name;Acc:G1TBI4]	BROX	ENSG00000162819	ortholog_one2one	1	88.3408	95.8637	Yes	Confident one-to-one ortholog
GPX1	ENSOCUG00000005938	glutathione peroxidase 1 [Source:NCBI gene (formerly Entrezgene);Acc:100009258]	GPX1	ENSG00000233276	ortholog_one2many	1	91.5000	90.1478	No	Flag: ortholog_one2many
RHOA	ENSOCUG00000013323	ras homolog family member A [Source:UniProtKB Gene Name;Acc:G1T567]	RHOA	ENSG00000067560	ortholog_one2one	1	100.0000	100.0000	Yes	Confident one-to-one ortholog
VWF	ENSOCUG00000006783	von Willebrand factor [Source:UniProtKB Gene Name;Acc:G1U334]	VWF	ENSG00000110799	ortholog_one2one	1	82.3171	81.5855	Yes	Confident one-to-one ortholog
NPM1	ENSOCUG00000021499	nucleophosmin 1 [Source:UniProtKB Gene Name;Acc:B7NZF9]							No	No human ortholog returned
TUFT1	ENSOCUG00000012659	tuftelin 1 [Source:UniProtKB Gene Name;Acc:B7NZE0]	TUFT1	ENSG00000143367	ortholog_one2one	1	86.9231	86.9231	Yes	Confident one-to-one ortholog
MTARC1	ENSOCUG00000013162	mitochondrial amidoxime reducing component 1 [Source:UniProtKB Gene Name;Acc:A0A5F9D6B1]	MTARC1	ENSG00000186205	ortholog_one2one	1	76.3158	77.4481	Yes	Confident one-to-one ortholog
IQGAP3	ENSOCUG00000012177	IQ motif containing GTPase activating protein 3 [Source:UniProtKB Gene Name;Acc:A0A5F9CYM5]	IQGAP3	ENSG00000183856	ortholog_one2one	1	86.2791	90.9871	Yes	Confident one-to-one ortholog
ILK	ENSOCUG00000006593	integrin linked kinase [Source:UniProtKB Gene Name;Acc:G1SRA9]	ILK	ENSG00000166333	ortholog_one2one	1	86.4706	97.5664	Yes	Confident one-to-one ortholog
PSMC2	ENSOCUG00000010217	proteasome 26S subunit, ATPase 2 [Source:UniProtKB Gene Name;Acc:G1SYV0]	PSMC2	ENSG00000161057	ortholog_one2one	1	100.0000	100.0000	Yes	Confident one-to-one ortholog
VIM	ENSOCUG00000009222	vimentin [Source:UniProtKB Gene Name;Acc:A0A5F9DS35]	VIM	ENSG00000026025	ortholog_one2one	1	96.7811	96.7811	Yes	Confident one-to-one ortholog
KIF5B	ENSOCUG00000017493	kinesin family member 5B [Source:UniProtKB Gene Name;Acc:A0A5F9DS06]	KIF5B	ENSG00000170759	ortholog_one2one	1	98.9142	94.6002	Yes	Confident one-to-one ortholog
DDX3X	ENSOCUG00000012338	DEAD-box helicase 3 X-linked [Source:UniProtKB Gene Name;Acc:G1T336]	DDX3X	ENSG00000215301	ortholog_one2one	1	99.2447	99.2447	Yes	Confident one-to-one ortholog
TKT	ENSOCUG00000015904	transketolase [Source:UniProtKB Gene Name;Acc:G1TAH7]	TKT	ENSG00000163931	ortholog_one2one	1	93.5795	93.5795	Yes	Confident one-to-one ortholog
RRAS2	ENSOCUG00000011362	RAS related 2 [Source:UniProtKB Gene Name;Acc:A0A5F9D8W5]	RRAS2	ENSG00000133818	ortholog_one2one	1	72.4444	79.9020	Yes	Confident one-to-one ortholog
PSME1	ENSOCUG00000002846	proteasome activator subunit 1 [Source:UniProtKB Gene Name;Acc:G1U8C4]	PSME1	ENSG00000092010	ortholog_one2one	1	93.5743	93.5743	Yes	Confident one-to-one ortholog
COPB1	ENSOCUG00000000432	COPI coat complex subunit beta 1 [Source:UniProtKB Gene Name;Acc:A0A5F9DSP2]	COPB1	ENSG00000129083	ortholog_one2one	1	99.2655	99.2655	Yes	Confident one-to-one ortholog
CNDP2	ENSOCUG00000004001	carnosine dipeptidase 2 [Source:UniProtKB Gene Name;Acc:G1SKV7]	CNDP2	ENSG00000133313	ortholog_one2one	1	90.3158	90.3158	Yes	Confident one-to-one ortholog
EEF1G	ENSOCUG00000021109	eukaryotic translation elongation factor 1 gamma [Source:NCBI gene (formerly Entrezgene);Acc:100009282]	EEF1G	ENSG00000254772	ortholog_one2one		97.6247	94.0503	No	Flag: low or missing orthology confidence
DNM1L	ENSOCUG00000000021	dynamin 1 like [Source:UniProtKB Gene Name;Acc:A0A5F9D7H7]	DNM1L	ENSG00000087470	ortholog_one2one	1	71.1991	90.3533	Yes	Confident one-to-one ortholog
SMAP1	ENSOCUG00000017134	small ArfGAP 1 [Source:UniProtKB Gene Name;Acc:A0A5F9CWY3]	SMAP1	ENSG00000112305	ortholog_one2one	1	84.8671	88.8651	Yes	Confident one-to-one ortholog
ITIH2	ENSOCUG00000004620	inter-alpha-trypsin inhibitor heavy chain 2 [Source:UniProtKB Gene Name;Acc:A0A5F9DNT2]	ITIH2	ENSG00000151655	ortholog_one2one	1	78.8350	85.8351	Yes	Confident one-to-one ortholog
CD59	ENSOCUG00000014601	CD59 molecule (CD59 blood group) [Source:NCBI gene (formerly Entrezgene);Acc:100009459]	CD59	ENSG00000085063	ortholog_one2one		20.2749	46.0938	No	Flag: low or missing orthology confidence
EEF1A1	ENSOCUG00000008481	eukaryotic translation elongation factor 1 alpha 1 [Source:NCBI gene (formerly Entrezgene);Acc:100009189]							No	No human ortholog returned
HSP90AB1	ENSOCUG00000012842	heat shock protein 90 alpha family class B member 1 [Source:UniProtKB Gene Name;Acc:P30947]	HSP90AB1	ENSG00000096384	ortholog_one2one	1	98.8981	99.1713	Yes	Confident one-to-one ortholog
DAG1	ENSOCUG00000003439	dystroglycan 1 [Source:NCBI gene (formerly Entrezgene);Acc:100009278]	DAG1	ENSG00000173402	ortholog_one2one	1	93.7430	93.7430	Yes	Confident one-to-one ortholog
TBCE	ENSOCUG00000017490	tubulin folding cofactor E [Source:UniProtKB Gene Name;Acc:A0A5F9CZR3]	TBCE	ENSG00000284770	ortholog_one2one	1	82.4859	83.1120	Yes	Confident one-to-one ortholog
RUVBL1	ENSOCUG00000016510	RuvB like AAA ATPase 1 [Source:UniProtKB Gene Name;Acc:G1TBS2]	RUVBL1	ENSG00000175792	ortholog_one2one	1	94.0206	100.0000	Yes	Confident one-to-one ortholog
CCT4	ENSOCUG00000024386	chaperonin containing TCP1 subunit 4 [Source:UniProtKB Gene Name;Acc:A0A5F9C9R5]	CCT4	ENSG00000115484	ortholog_one2one	1	96.3702	98.5158	Yes	Confident one-to-one ortholog
GAPDH	ENSOCUG00000029891	glyceraldehyde-3-phosphate dehydrogenase [Source:NCBI gene (formerly Entrezgene);Acc:100009074]	GAPDH	ENSG00000111640	ortholog_one2many	1	94.8949	94.3284	No	Flag: ortholog_one2many
SAR1B	ENSOCUG00000008250	secretion associated Ras related GTPase 1B [Source:UniProtKB Gene Name;Acc:A0A5F9DAK5]	SAR1B	ENSG00000152700	ortholog_one2one	1	57.6271	85.8586	Yes	Confident one-to-one ortholog
PLAA	ENSOCUG00000015471	phospholipase A2 activating protein [Source:UniProtKB Gene Name;Acc:G1T9I3]	PLAA	ENSG00000137055	ortholog_one2one	1	95.8491	95.8491	Yes	Confident one-to-one ortholog
PSMA1	ENSOCUG00000000439	proteasome 20S subunit alpha 1 [Source:HGNC Symbol;Acc:HGNC:9530]	PSMA1	ENSG00000129084	ortholog_one2one	1	100.0000	100.0000	Yes	Confident one-to-one ortholog
ANXA1	ENSOCUG00000015493	annexin A1 [Source:UniProtKB Gene Name;Acc:G1TIS5]	ANXA1	ENSG00000135046	ortholog_one2one	1	91.0180	87.8613	Yes	Confident one-to-one ortholog
ATP5F1C	ENSOCUG00000004642	ATP synthase F1 subunit gamma [Source:UniProtKB Gene Name;Acc:G1SM77]	ATP5F1C	ENSG00000165629	ortholog_one2one	1	94.2953	94.2953	Yes	Confident one-to-one ortholog
FGF1	ENSOCUG00000013987	fibroblast growth factor 1 [Source:NCBI gene (formerly Entrezgene);Acc:100328898]	FGF1	ENSG00000113578	ortholog_one2one	1	96.7742	96.7742	Yes	Confident one-to-one ortholog
EPRS1	ENSOCUG00000012039	glutamyl-prolyl-tRNA synthetase 1 [Source:UniProtKB Gene Name;Acc:G1T2I4]	EPRS1	ENSG00000136628	ortholog_one2one	1	92.3290	88.3598	Yes	Confident one-to-one ortholog
UBA6	ENSOCUG00000012827	ubiquitin like modifier activating enzyme 6 [Source:UniProtKB Gene Name;Acc:G1T466]	UBA6	ENSG00000033178	ortholog_one2one	1	93.0608	93.0608	Yes	Confident one-to-one ortholog
HMGB1	ENSOCUG00000024061	high mobility group box 1 [Source:UniProtKB Gene Name;Acc:G1U2Q5]	HMGB1	ENSG00000189403	ortholog_many2many		100.0000	100.0000	No	Flag: ortholog_many2many
HMGB1	ENSOCUG00000024061	high mobility group box 1 [Source:UniProtKB Gene Name;Acc:G1U2Q5]	HMGB1P1	ENSG00000124097	ortholog_many2many	1	93.0233	94.7867	No	Flag: ortholog_many2many
RNF17	ENSOCUG00000007384	ring finger protein 17 [Source:UniProtKB Gene Name;Acc:A0A5F9C3V8]	RNF17	ENSG00000132972	ortholog_one2one	1	80.3837	80.0370	Yes	Confident one-to-one ortholog
COPG1	ENSOCUG00000005970	COPI coat complex subunit gamma 1 [Source:UniProtKB Gene Name;Acc:A0A5F9D277]	COPG1	ENSG00000181789	ortholog_one2one	1	99.0847	99.0847	Yes	Confident one-to-one ortholog
PSMG2	ENSOCUG00000005086	proteasome assembly chaperone 2 [Source:UniProtKB Gene Name;Acc:G1SN64]	PSMG2	ENSG00000128789	ortholog_one2one	1	55.1980	84.4697	Yes	Confident one-to-one ortholog
LAP3	ENSOCUG00000001571	leucine aminopeptidase 3 [Source:UniProtKB Gene Name;Acc:A0A5F9C6Y6]	LAP3	ENSG00000002549	ortholog_one2one	1	91.7148	91.7148	Yes	Confident one-to-one ortholog
CCT3	ENSOCUG00000000137	chaperonin containing TCP1 subunit 3 [Source:UniProtKB Gene Name;Acc:A0A5F9CJQ3]	CCT3	ENSG00000163468	ortholog_one2one	1	98.1651	98.1651	Yes	Confident one-to-one ortholog
CALD1	ENSOCUG00000008127	caldesmon 1 [Source:NCBI gene (formerly Entrezgene);Acc:100009077]	CALD1	ENSG00000122786	ortholog_one2one	1	85.9964	60.4035	Yes	Confident one-to-one ortholog
TAGLN2	ENSOCUG00000022296	transgelin 2 [Source:UniProtKB Gene Name;Acc:G1TKE3]	TAGLN2	ENSG00000158710	ortholog_one2one	1	80.0000	74.3719	Yes	Confident one-to-one ortholog
HMGB1	ENSOCUG00000022718	high mobility group box 1 [Source:UniProtKB Gene Name;Acc:G1U2Q5]							No	No human ortholog returned
RNF103	ENSOCUG00000029277	ring finger protein 103 [Source:UniProtKB Gene Name;Acc:A0A5F9DQV7]	RNF103	ENSG00000239305	ortholog_one2one	1	93.5673	93.4307	Yes	Confident one-to-one ortholog
ARPC2	ENSOCUG00000005516	actin related protein 2/3 complex subunit 2 [Source:UniProtKB Gene Name;Acc:A0A5F9CUC6]	ARPC2	ENSG00000163466	ortholog_one2one	1	91.3043	98.0000	Yes	Confident one-to-one ortholog
ANXA4	ENSOCUG00000015796	annexin A4 [Source:UniProtKB Gene Name;Acc:G1TA83]	ANXA4	ENSG00000196975	ortholog_one2one	1	93.7695	93.7695	Yes	Confident one-to-one ortholog
PRKCI	ENSOCUG00000014563	protein kinase C iota [Source:UniProtKB Gene Name;Acc:A0A5F9CT72]	PRKCI	ENSG00000163558	ortholog_one2one	1	99.1611	99.1611	Yes	Confident one-to-one ortholog
AAK1	ENSOCUG00000009550	AP2 associated kinase 1 [Source:UniProtKB Gene Name;Acc:A0A5F9D9C1]	AAK1	ENSG00000115977	ortholog_one2one	1	69.2720	94.0687	Yes	Confident one-to-one ortholog
VCP	ENSOCUG00000006433	valosin containing protein [Source:UniProtKB Gene Name;Acc:G1SR03]	VCP	ENSG00000165280	ortholog_one2one	1	100.0000	100.0000	Yes	Confident one-to-one ortholog
HSPA8	ENSOCUG00000015533	heat shock protein family A (Hsp70) member 8 [Source:HGNC Symbol;Acc:HGNC:5241]	HSPA8	ENSG00000109971	ortholog_one2one	1	70.4471	100.0000	Yes	Confident one-to-one ortholog
TUBB	ENSOCUG00000002187	tubulin beta class I [Source:UniProtKB Gene Name;Acc:G1SH05]	TUBB	ENSG00000196230	ortholog_one2one	1	99.5496	99.5496	Yes	Confident one-to-one ortholog
RAB10	ENSOCUG00000029308	RAB10, member RAS onco family [Source:UniProtKB Gene Name;Acc:U3KMP1]	RAB10	ENSG00000084733	ortholog_one2one	1	82.2511	95.0000	Yes	Confident one-to-one ortholog
PACS1	ENSOCUG00000025097	phosphofurin acidic cluster sorting protein 1 [Source:UniProtKB Gene Name;Acc:A0A5F9DF76]	PACS1	ENSG00000175115	ortholog_one2one	1	87.5817	83.4891	Yes	Confident one-to-one ortholog
G6PD	ENSOCUG00000007866	glucose-6-phosphate dehydrogenase [Source:UniProtKB Gene Name;Acc:A0A5F9DG82]	G6PD	ENSG00000160211	ortholog_one2one	1	79.6902	89.9029	Yes	Confident one-to-one ortholog
SUCLG1	ENSOCUG00000003780	succinate-CoA ligase GDP/ADP-forming subunit alpha [Source:UniProtKB Gene Name;Acc:A0A5F9CBK9]	SUCLG1	ENSG00000163541	ortholog_one2one	1	89.7507	93.6416	Yes	Confident one-to-one ortholog
WDR1	ENSOCUG00000002549	WD repeat domain 1 [Source:UniProtKB Gene Name;Acc:A0A5F9DBZ9]	WDR1	ENSG00000071127	ortholog_one2one	1	95.7627	93.2343	Yes	Confident one-to-one ortholog
LAMB1	ENSOCUG00000014676	laminin subunit beta 1 [Source:UniProtKB Gene Name;Acc:G1T7X6]	LAMB1	ENSG00000091136	ortholog_one2one	1	93.1452	90.5375	Yes	Confident one-to-one ortholog

BCAP31	ENSOCUG00000017921	B cell receptor associated protein 31 [Source:UniProtKB Gene Name;Acc:G1TER3]	BCAP31	ENSG00000185825	ortholog_one2one	1	92.6829	92.6829	Yes	Confident one-to-one ortholog
HYOU1	ENSOCUG00000004239	hypoxia up-regulated 1 [Source:UniProtKB Gene Name;Acc:G1SLD5]	HYOU1	ENSG00000149428	ortholog_one2one	1	90.7908	90.7908	Yes	Confident one-to-one ortholog
